# Supplementary material for: Direct nucleophilic and electrophilic activation of alcohols using a unified boron-based organocatalyst scaffold
Source: Nat Commun. 2023 May 4;14:2563. doi: 10.1038/s41467-023-38228-8 (PMC10160031; doi:10.1038/s41467-023-38228-8)
Supplement: Supplementary file 1 — Supplementary Information [file 41467_2023_38228_MOESM1_ESM.pdf]

Supplementary Information

Direct nucleophilic and electrophilic activation of alcohols using a unified boron-based organocatalyst scaffold

Jason P. G. Rygus, Dennis G. Hall*

Department of Chemistry, Centennial Centre for Interdisciplinary Science, University of Alberta, Edmonton, Alberta Canada T6G 2G2

dennis.hall@ualberta.ca

Table of Contents

Supplementary Methods 4

1. General Information 4

Supplementary Discussion 5

2. Synthesis and Characterization of Boron Heterocycles 5

 2.1 Synthesis of Boron Heterocycles 5

 2.2 pK_a Measurements for Boron Heterocycles 5

3. Synthesis and Characterization of 1-Aryl-1,2-Diols 6

4. Monophophorylation – Initial Stoichiometric Study, Reaction Optimization and Diol Complexation Studies by ^{11}B NMR 9

 4.1 Initial Stoichiometric Studies (Figure 2a) 9

 4.2 Cation-Dependent Conformational Equilibration of Tetravalent Borate **2-II** 12

 4.3 Optimization of Catalytic Monophosphorylation (Figure 2b) 15

 4.4 Diol Complexation Studies by ^{11}B NMR (Figure 3a) 17

5. Monophosphorylation – Substrate Scope (*cf.* Figure 3b) 22

6. Synthesis and Characterization of Benzylic Alcohols and Ketones 26

 6.1 Synthesis and Characterization of Benzylic Alcohols 26

 6.2 Synthesis and Characterization of Benzylic Ketones 34

7. Reductive Deoxygenation – Optimization 37

 7.1 Optimization of Reductive Deoxygenation of Benzylic Alcohols (Table 1) 37

 7.2 Optimization of Reductive Deoxygenation of Benzylic Ketones 37

8. Reductive Deoxygenation – Substrate Scope 38

 8.1 Reductive Deoxygenation of Benzylic Alcohols (Figure 5) 38

 8.2 Reductive Deoxygenation of Benzylic Ketones (Figure 6) 46

 8.3 Comparison of Deoxygenation Methods for Alcohol **6h** 50

 8.4 Unsuccessful Deoxygenation Substrates 51

9. Deoxygenation of 1-Adamantol, Two-Step Ethylation of Trimethoxybenzene and Comparison of Catalysts in Model Reactions 52

 9.1 Deoxygenation of 1-Adamantol (Figure 7a) 52

 9.2 Two-Step Ethylation of Trimethoxybenzene (Figure 7a) 53

 9.3 Comparison of Catalysts in Model Monophosphorylation and Alcohol Deoxygenation Reactions (Figure 7b) 53

10. Mechanistic and Kinetic Studies of Reductive Deoxygenation 54

 10.1 Ketone Deoxygenation Kinetics (Figure 8a) 54

 10.2 Evidence for Formation and Hydrolysis of Silyl Ether Intermediates (Figure 8b) 55

10.3 Kinetics of Ketone Consumption in Deoxygenation Reactions (Figure 8c) 61
10.4 ^{11}B NMR Analysis of Alcohol Deoxygenation (Figure 9) 61
10.5 Crystallization of Bis(hexafluoroisopropoxy)boronate Zwitterion **3-II** and NMR Study of Boron Speciation 69
Supplementary Notes 77
11. NMR Spectra 77
12. Crystallographic Data 170
Supplementary References 179

Supplementary Methods

1. General Information

The following supporting information contains representative experimental procedures and details for the isolation and characterization of compounds. Full characterization of all novel compounds and partial characterization of known compounds are described. All reactions were performed in regular glassware with no exclusion of air or moisture unless otherwise noted. 2-formylphenylboronic acid was purchased from Combi-Blocks and recrystallized from hot H_2O prior to use. Hydroxylamine (50 wt. % solution in water) was purchased from Sigma Aldrich and used as received. *N*-methylhydroxylamine hydrochloride was purchased from Combi-Blocks and used as received. 1,1,1,3,3,3-hexafluoroisopropanol (HFIP) was purchased from Oakwood Chemical and used as received. All other solvents were purchased as ACS reagent grade and used as received, and other chemicals were purchased from commercial suppliers and used as received. Thin layer chromatography was performed on Silicycle silica gel 60 F254 plates, which were visualized under UV light and with $KMnO_4$ or phosphomolybdic acid (PMA) stains. Column chromatographic separations were performed on silica gel 60 using ACS grade hexanes, ethyl acetate and dichloromethane as eluents.

NMR spectra were recorded at ambient temperature using Varian DD2 MR two-channel 400 MHz, Varian INOVA two-channel 400MHz, Varian INOVA four-channel 500 MHz, Varian VNMRS two-channel 500 MHz, Varian VNMRS four-channel 600 MHz and Agilent VNMRS four-channel, dual receiver 700 MHz spectrometers operating at the indicated frequency for ^{1}H NMR. All NMR chemical shifts are reported in ppm (δ) units with residual solvent peaks ($CDCl_3$, CD_3CN or D_2O) as the internal reference. NMR data is reported using the following abbreviations: s, singlet; br s, broad singlet; d, doublet; t, triplet; q, quartet; p, pentet; h, hextet; dd, doublet of doublets; dt, doublet of triplets; td, triplet of doublets; ddd, doublet of doublet of doublets; dddd, doublet of doublet of doublet of doublets; app, apparent; m, multiplet. The error of coupling constants from ^{1}H NMR spectra is estimated to be approximately 0.3 Hz. The quaternary carbon bound to boron is often not observed due to the quadrupolar relaxation of boron, which was the case for all boron-containing compounds herein.

An OHAUS ST2100 pH meter with ST350 pH probe was used for pH measurements. High-resolution mass spectra were recorded by the University of Alberta Mass Spectrometry Services Laboratory using either electron impact (EI) or electrospray (ESI) techniques. Melting points were determined in a capillary tube using a melting point apparatus and are uncorrected. Fourier-transform infrared (FTIR) spectra were obtained on a Nicolet Magna-IR instrument.

Supplementary Discussion

2. Synthesis and Characterization of Boron Heterocycles

2.1 Synthesis of Boron Heterocycles

Boron heterocycles **1a**, **1b**, **1c** and **2** were synthesized as described previously.[1]

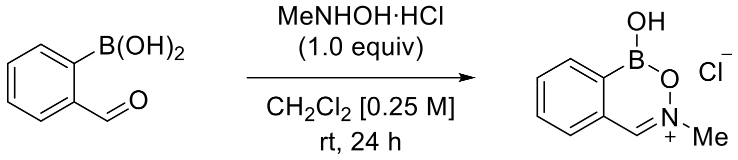

S1 **3**

1-Hydroxy-3-methyl-1H-benzo[d][1,2,6]oxazaborinin-3-ium chloride (3): In a round bottom flask under air, 2-formylphenylboronic acid (1.20 g, 8.00 mmol) was dissolved in CH_2Cl_2 (32 mL) and stirred for 10 minutes, after which *N*-methylhydroxylamine hydrochloride (668 mg, 8.00 mmol, 1.00 equiv) was added in one portion. The mixture was stirred at room temperature for 24 hours. Upon completion, the precipitate was collected by vacuum filtration, washed with CH_2Cl_2 (6 × 15 mL), and dried under vacuum to afford the title compound as a white solid (1.48 g, 94%). **mp** = 234 – 237 °C (sweating and change in morphology observed at 145.6 – 148.1 °C); **^{1}H NMR** (500 MHz, D_2O): δ 8.59 (s, 1 H), 7.78 (t, *J* = 7.4 Hz, 1 H), 7.70 (d, *J* = 7.4 Hz, 1 H), 7.62 (d, *J* = 7.7 Hz, 1 H), 7.55 (t, *J* = 7.6 Hz, 1 H), 3.94 (s, 3 H); **^{13}C NMR** (126 MHz, D_2O): δ 148.9, 136.8, 131.0, 130.9, 129.5, 127.6, 49.6; **^{11}B NMR** (128 MHz, D_2O): δ 4.5; **FTIR** (microscope, cm^{-1}): 3362 (br, s), 3324 (m), 3062 (w), 2980 (m), 1648 (m), 1560 (m), 1392 (m), 1158 (m), 1101 (m), 998 (w), 769 (m), 708 (w); **HRMS** (ESI Positive Mode) for $C_8H_9BNO_2^+$: Calculated: 162.0721; Found: 162.0722.

2.2 pK_a Measurements for Boron Heterocycles

The pK_a values of boron heterocycles **1a**, **1b**, **1c** and **2** were determined previously.[1] The following procedure was used for the determination of the pK_a of heterocycle **3** by ^{11}B NMR.

Boron heterocycle **3** (49.4 mg, 0.250 mmol) was dissolved in 5.0 mL D_2O. The solution was then diluted to a total volume of 25 mL using a phosphate buffer solution, which was prepared by dissolving 690 mg sodium phosphate monobasic monohydrate in 50.0 mL deionized water. Aliquots of 2.0 mL from the boron heterocycle stock solution were transferred to 3-dram vials, where the pH of the solutions was then adjusted using 1.0 M HCl, 0.1 M NaOH or 1.0 M NaOH and measured using a pH meter. After a stable pH reading was observed, approximately 700 μL of each aliquot was transferred to an NMR tube and analyzed by ^{11}B NMR spectroscopy, using D_2O as the solvent for locking and shimming.

No significant change in ^{11}B NMR chemical shift was observed between pH 0.81–13.04. Across this entire range, a chemical shift between 4.1–4.6 ppm was observed corresponding to a

tetravalent boron environment. Thus, the pK_a of heterocycle **3** in H_2O can be estimated to be less than 1.

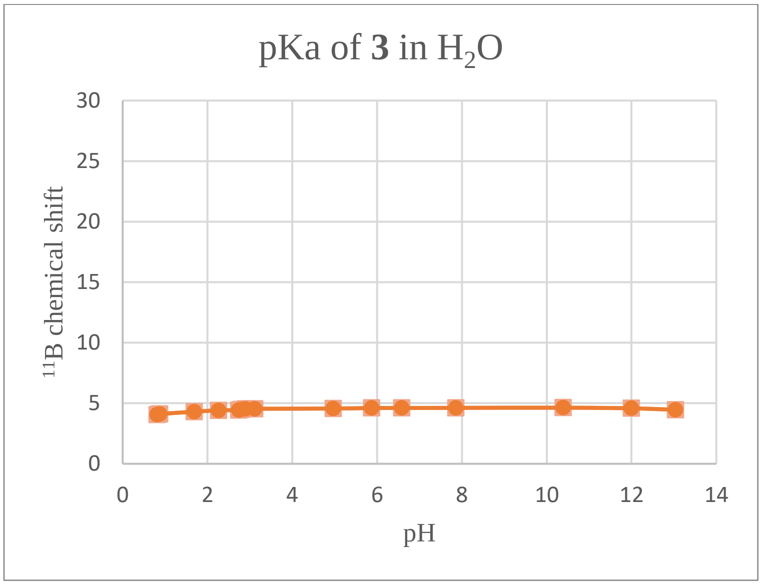

Supplementary Figure 1. Attempted pK_a determination of heterocycle **3** in H_2O.

3. Synthesis and Characterization of 1-Aryl-1,2-Diols

General Procedure for the synthesis of 1-aryl-1,2-diols from acetophenones (GP1)

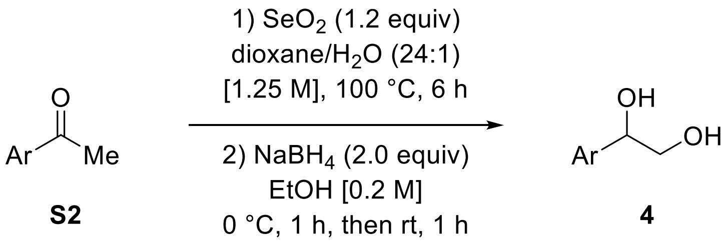

S2 **4**

A 10 mL round bottom flask was charged with SeO_2 (1.2 equiv), dioxane and H_2O (24:1 ratio, 1.25 M in limiting reagent). The mixture was heated to 55 °C until fully soluble (approximately 5 minutes), after which it was allowed to cool to room temperature. The corresponding acetophenone **S2** (1.0 equiv) was then added in a single portion. The mixture was heated to reflux at 100 °C for 6 hours. A dark red solution was observed soon after the heating began, followed by a dark green solution over time along with precipitation of a black solid. After 6 hours, the reaction was allowed to cool to room temperature and filtered through fluted filter paper (caution: the filter paper contains selenium waste and must be disposed of accordingly) to yield a pale yellow/green solution, which was concentrated by rotary evaporation. The crude mixture was passed through a short column of silica gel using hexanes/ethyl acetate and concentrated by rotary evaporation, after which it was dissolved in ethanol (0.2 M) and cooled to 0 °C. $NaBH_4$ (2.0 equiv) was added in two

portions approximately 5 minutes apart. The reaction was stirred at 0 °C for 1 hour, and then stirred for an additional 1 hour after removing the ice bath. The reaction was quenched with 1M $HCl_{(aq)}$ and concentrated by rotary evaporation to remove EtOH. The resulting mixture was extracted with ethyl acetate (3 × 25 mL). The combined organic phases were washed with water (30 mL) and brine (30 mL), dried over Na_2SO_4 and filtered. After removal of the solvent by rotary evaporation, purification by column chromatography afforded the diol **4**.

Diols **4a** and **4f** were purchased from commercial suppliers and used as received.

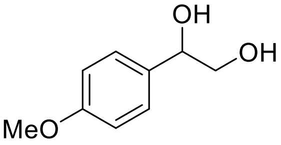

1-(4-Methoxyphenyl)ethane-1,2-diol (4b): Prepared according to **GP1** from 4-methoxyacetophenone (751 mg, 5.00 mmol). Purification by column chromatograph (1:2 hexane/EtOAc) afforded the title compound as a white solid (145 mg, 17%). **^{1}H NMR** (500 MHz, $CDCl_3$): δ 7.29 (d, *J* = 8.7 Hz, 2 H), 6.90 (d, *J* = 8.6 Hz, 2 H), 4.77 (dd, *J* = 8.2, 3.7 Hz, 1 H), 3.80 (s, 3 H), 3.72 (dd, *J* = 11.3, 3.7 Hz, 1 H), 3.65 (dd, *J* = 11.3, 8.2 Hz, 1 H), 2.53 (br s, 1 H), 2.15 (br s, 1 H); **^{13}C NMR** (126 MHz, $CDCl_3$): δ 159.6, 132.8, 127.5, 114.1, 74.4, 68.2, 55.5. Spectral data were in agreement with the literature.[2]

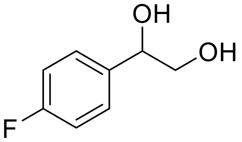

1-(4-Fluorophenyl)ethane-1,2-diol (4c): Prepared according to **GP1** from 4-fluoroacetophenone (607 µL, 5.00 mmol) and obtained as a white solid (390 mg, 50%) without further purification. **^{1}H NMR** (500 MHz, $CDCl_3$): δ 7.37 (dd, *J* = 8.6, 5.5 Hz, 2 H), 7.08 (app t, *J* = 8.7 Hz, 2 H), 4.84 (dd, *J* = 8.3, 3.5 Hz, 1 H), 3.77 (dd, *J* = 11.3, 3.5 Hz, 1 H), 3.66 (dd, *J* = 11.3, 8.2 Hz, 1 H), 2.78 (s, 1 H), 2.29 (s, 1 H); **^{13}C NMR** (126 MHz, $CDCl_3$): δ 162.6 (d, *J* = 246.2 Hz), 136.3 (d, *J* = 3.1 Hz), 127.9 (d, *J* = 8.3 Hz), 115.6 (d, *J* = 21.4 Hz), 74.2, 68.2; **^{19}F NMR** (376 MHz, $CDCl_3$): δ –114.3 (app tt, *J* = 8.8, 5.2 Hz). Spectral data were in agreement with the literature.[2]

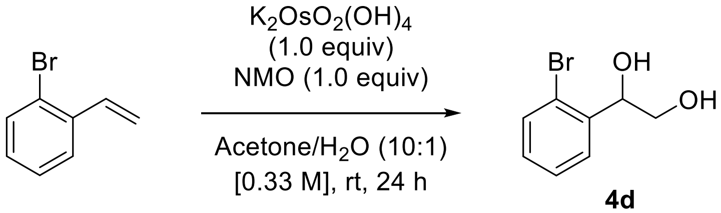

4d

1-(2-Bromophenyl)ethane-1,2-diol (4d): Under nitrogen, a 25 mL round bottom flask was charged with N-methylmorpholine N-oxide (352 mg, 3.00 mmol, 1.00 equiv) and $K_2OsO_2(OH)_4$ (12.1 mg, 0.03 mmol, 1 mol%). The solids were suspended in acetone (8.20 mL) and H_2O (0.820 mL). 4-bromostyrene (376 µL, 3.00 mmol, 1.00 equiv) was added via syringe and the mixture was stirred at room temperature for 24 hours. The reaction was then diluted with H_2O (25 mL) and

extracted with ethyl acetate (3 × 25 mL). The combined organic phases were washed with brine (25 mL), dried over Na_2SO_4 and filtered. The solvent was removed by rotary evaporation, and purification by column chromatography (2:1 hexane/EtOAc) afforded the title compound as a white solid (169 mg, 26%). **^{1}H NMR** (500 MHz, $CDCl_3$): δ 7.63 (dd, *J* = 7.8, 1.7 Hz, 1 H), 7.57 (dd, *J* = 8.0, 1.2 Hz, 1 H), 7.39 (td, *J* = 7.6, 1.2 Hz, 1H), 7.20 (td, *J* = 7.7, 1.8 Hz, 1 H), 5.24 (dt, *J* = 8.1, 3.0 Hz, 1 H), 3.96 (ddd, *J* = 10.7, 6.9, 3.1 Hz, 1 H), 3.61 (ddd, *J* = 11.6, 7.8, 4.2 Hz, 1 H), 2.67 (d, *J* = 3.3 Hz, 1 H), 2.08 – 2.02 (m, 1 H); **^{13}C NMR** (126 MHz, $CDCl_3$): δ 139.5, 132.9, 129.5, 128.0, 127.9, 122.1, 73.7, 66.4. Spectral data were in agreement with the literature.[3]

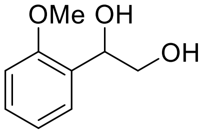

1-(2-Methoxyphenyl)ethane-1,2-diol (4e): Prepared according to **GP1** from 2-methoxyacetophenone (827 µL, 6.00 mmol). Purification by column chromatography (1:1 hexane/EtOAc) afforded the title compound as a white solid (403 mg, 40%). **^{1}H NMR** (500 MHz, $CDCl_3$): δ 7.38 (dd, *J* = 7.5, 1.7 Hz, 1 H), 7.31 – 7.25 (m, 1 H), 6.98 (td, *J* = 7.5, 1.0 Hz, 1 H), 6.89 (dd, *J* = 8.3, 1.0 Hz, 1 H), 5.05 (dd, *J* = 8.0, 3.6 Hz, 1 H), 3.85 (s, 3 H), 3.81 (dd, *J* = 11.2, 3.6 Hz, 1 H), 3.69 (dd, *J* = 11.2, 8.0 Hz, 1 H), 2.92 (br s, 1 H), 2.20 (br s, 1 H); **^{13}C NMR** (126 MHz, $CDCl_3$): δ 156.7, 129.0, 128.5, 127.4, 121.0, 110.6, 71.4, 66.6, 55.4. Spectral data were in agreement with the literature.[4]

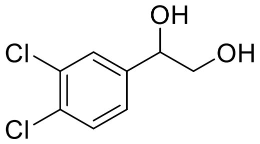

1-(3,4-Dichlorophenyl)ethane-1,2-diol (4g): Prepared according to **GP1** from 3,4-dichloroacetophenone (1.13 g, 6.00 mmol). Purification by column chromatography (gradient 1:1 to 1:2 hexane/EtOAc) afforded the title compound as a clear oil (1.04 g, 83%). **^{1}H NMR** (500 MHz, $CDCl_3$): δ 7.48 (d, *J* = 2.0 Hz, 1 H), 7.42 (d, *J* = 8.2 Hz, 1 H), 7.19 (dd, *J* = 8.3, 2.1 Hz, 1 H), 4.78 (dd, *J* = 8.1, 3.5 Hz, 1 H), 3.76 (dd, *J* = 11.3, 3.4 Hz, 1 H), 3.60 (dd, *J* = 11.3, 8.0 Hz, 1 H), 2.82 (br s, 1 H), 2.20 (br s, 1 H); **^{13}C NMR** (126 MHz): δ 140.8, 132.9, 132.1, 130.6, 128.3, 125.5, 73.6, 67.9. Spectral data were in agreement with the literature.[5]

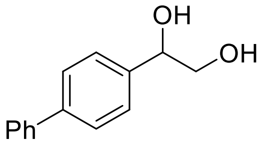

1-([1,1'-Biphenyl]-4-yl)ethane-1,2-diol (4h): Prepared according to **GP1** from 4-acetylbiphenyl (1.17 g, 6.00 mmol) and obtained as a white solid (583 mg, 45%) without further purification. **^{1}H NMR** (500 MHz, $CDCl_3$): δ 7.62 – 7.56 (m, 4 H), 7.50 – 7.39 (m, 4 H), 7.39 – 7.32 (m, 1 H), 4.89 (dd, *J* = 8.1, 3.6 Hz, 1 H), 3.83 (dd, *J* = 11.3, 3.6 Hz, 1 H), 3.73 (dd, *J* = 11.3, 8.1 Hz, 1 H), 1.58 (br s, 2 H); **^{13}C NMR** (126 MHz, $CDCl_3$): δ 141.2, 140.9, 139.6, 129.0, 127.6, 127.5, 127.2, 126.7, 74.6, 68.2. Spectral data were in agreement with the literature.[5]

4. Monophophorylation – Initial Stoichiometric Study, Reaction Optimization and Diol Complexation Studies by ^{11}B NMR

4.1 Initial Stoichiometric Studies (Figure 2a)

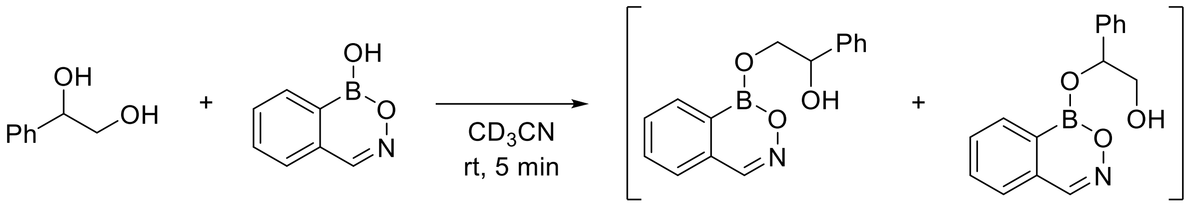

4a **2** (1.0 equiv) **2 - I**

A half-dram vial was charged with diol **4a** (10.4 mg, 0.0750 mmol) and boron heterocycle **2** (10.9 mg, 0.0750 mmol, 1.00 equiv). The solids were dissolved in CD_3CN to give a homogeneous solution, which was transferred to an NMR tube for analysis. Two new multiplets were observed in the 4.0 – 5.5 ppm region downfield from the corresponding resonances in free **4a**, consistent with formation of hemiboronic ester **2 – I** through boranol exchange. The broadness of these new resonances suggests a dynamic exchange process and may indicate that both regioisomers of **2 – I** exist in equilibrium. The ^{11}B NMR spectrum displayed a new resonance slightly upfield relative to the starting heterocycle, also consistent with boranol exchange. The formation of ester **2 - I** was also supported by HRMS of the reaction mixture ($C_{15}H_{14}NO_3{}^{11}BNa$ $[M+Na]^+$: Calculated: 290.0964; Found: 290.0960).

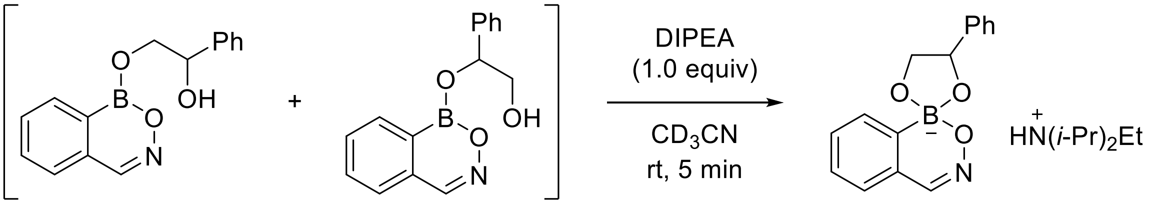

2 - I **2 - II**

N,N-diisopropylethylamine (13.1 μL, 0.0750 mmol, 1.0 equiv) was then added to the NMR tube and the solution mixed thoroughly. ^{11}B NMR displayed greater than 95% conversion to a tetravalent boronate (7.2 ppm), consistent with formation of adduct **2 – II.** The formation of adduct **2 - II** was also supported by HRMS of the reaction mixture in negative mode ($C_{15}H_{13}NO_3{}^{11}B^-$: Calculated: 266.0994; Found: 266.090).

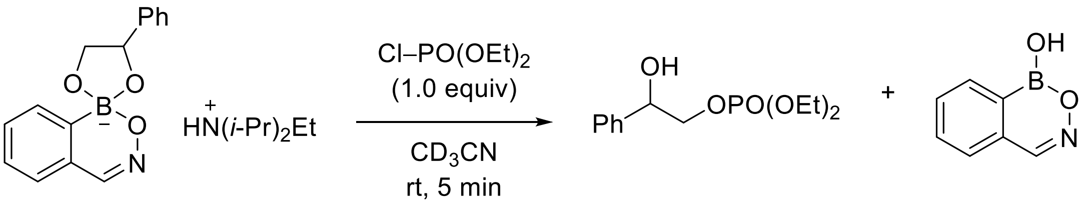

2 - II **5a** **2**

Diethyl chlorophosphate (10.8 μL, 1.00 equiv) was then added to the NMR tube and the solution mixed thoroughly. ^{11}B NMR showed complete consumption of tetravalent adduct **2 – II** and regeneration of heterocycle **2**, suggesting that after electrophile trapping of **2 – II**, the trivalent hemiester formed between **2** and **5a** undergoes hydrolysis from the water released in initial adduct formation. ^{1}H NMR also demonstrated formation of a multiplet (4.87 ppm, t, J = 5.4 Hz) consistent with an authentic sample of **5a** isolated from subsequent catalytic experiments.

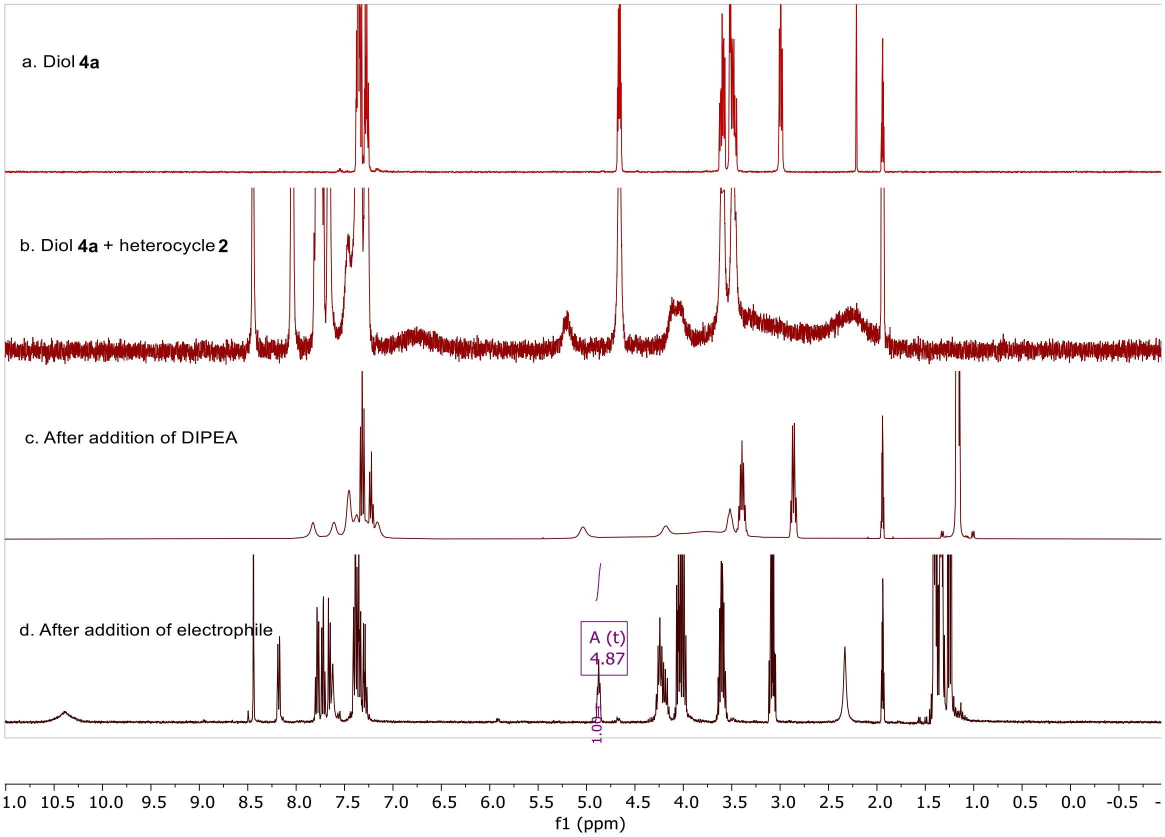

Supplementary Figure 2. ^{1}H NMR monitoring of stoichiometric monophosphorylation of diol **4a** mediated by heterocycle **2**.

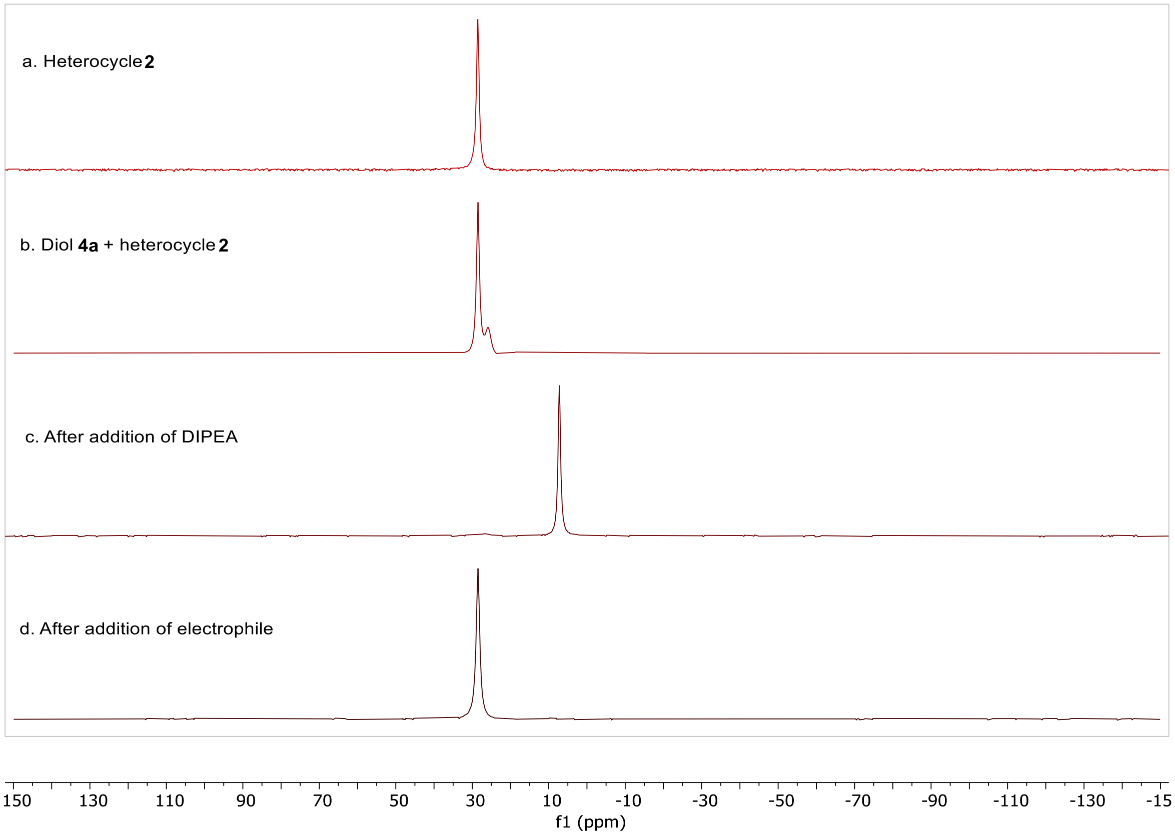

Supplementary Figure 3. ^{11}B NMR monitoring of stoichiometric monophosphorylation of diol **4a** mediated by heterocycle **2**.

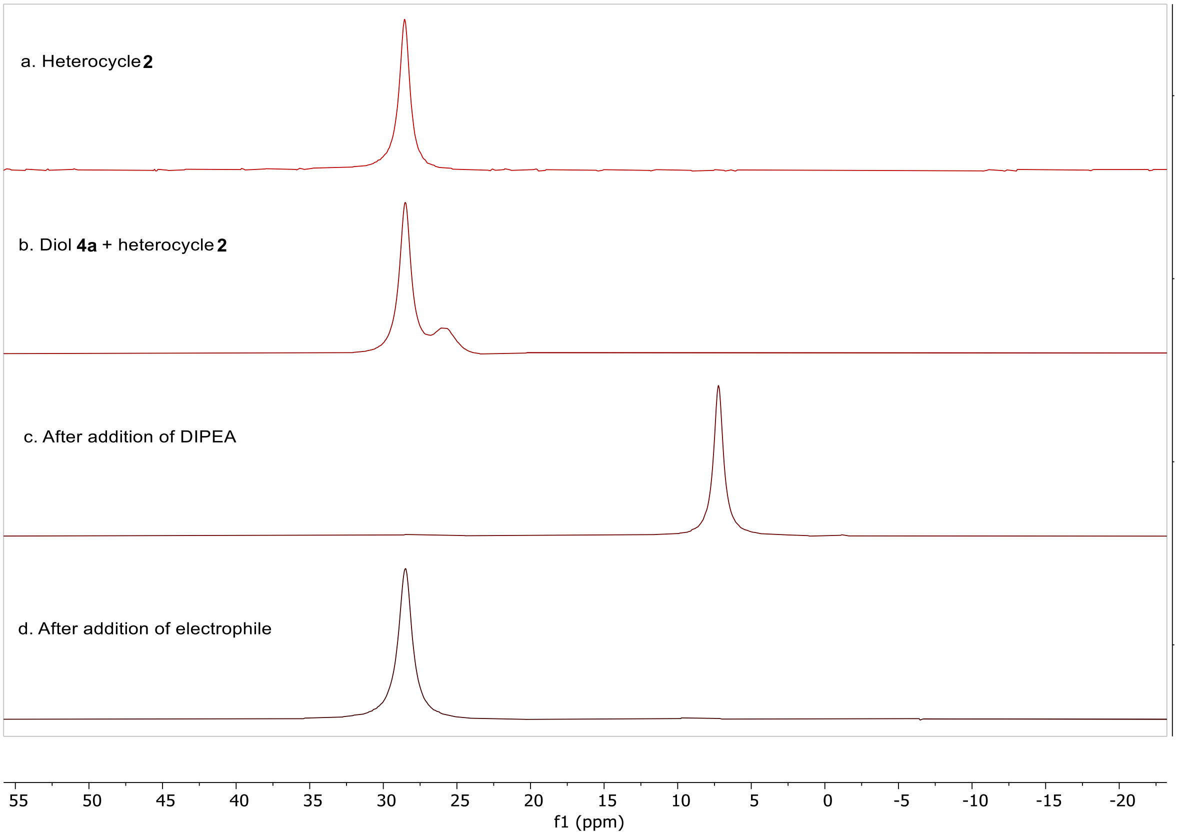

Supplementary Figure 4. Expansion of ^{11}B NMR monitoring of stoichiometric monophosphorylation of diol **4a** mediated by heterocycle **2**.

4.2 Cation-Dependent Conformational Equilibration of Tetravalent Borate 2-II

Upon formation of tetravalent borate **2-II** using DIPEA as a base as described in Section 4.1, the resulting ^{1}H NMR spectrum (CD_3CN, 400 MHz) displayed notably broad resonances, particularly those corresponding to the diol fragment (signals corresponding to sp^3 C–H resonances from the diol are marked with an * below).

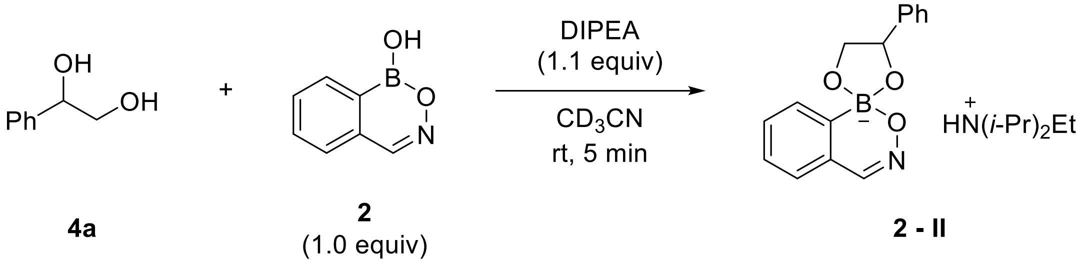

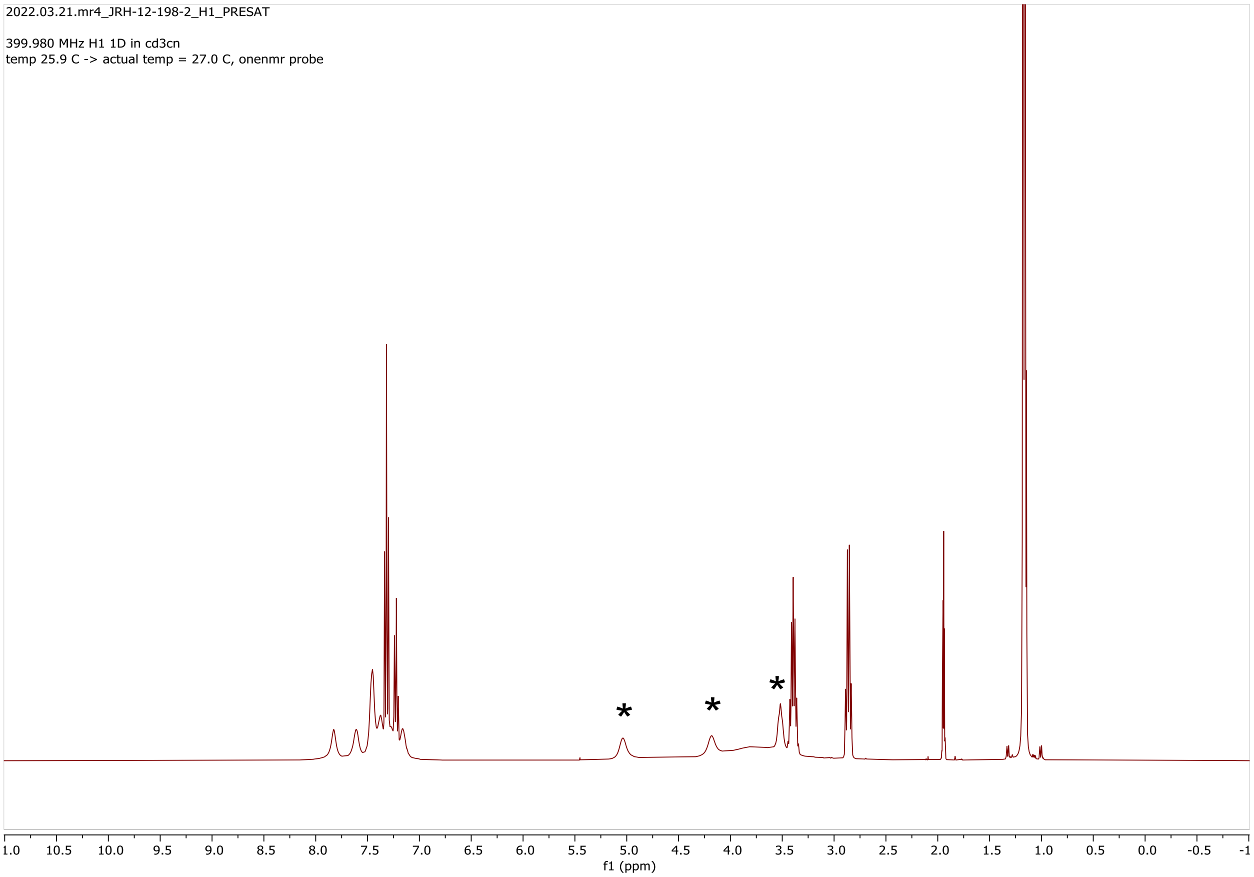

Supplementary Figure 5. ^{1}H NMR for formation of tetravalent borate **2-II** using DIPEA as a base.

In contrast, the use of tetra-*n*-butylammonium hydroxide as a base for tetravalent borate formation afforded two species by ^{1}H NMR (d_6-acetone, 700 MHz) in a ratio of approximately 60 : 40. The resonances did not demonstrate the broadness that was evident when DIPEA was used as a base.

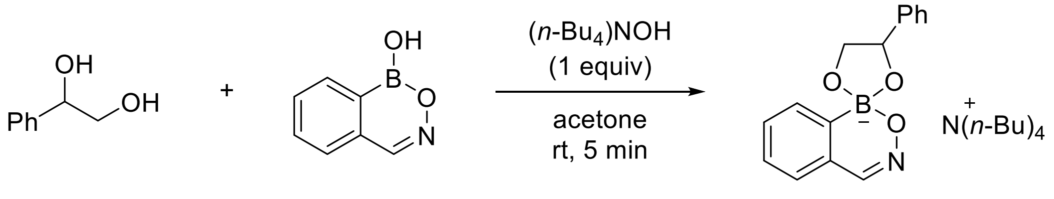

4a **2** (1.0 equiv) **2 - II**

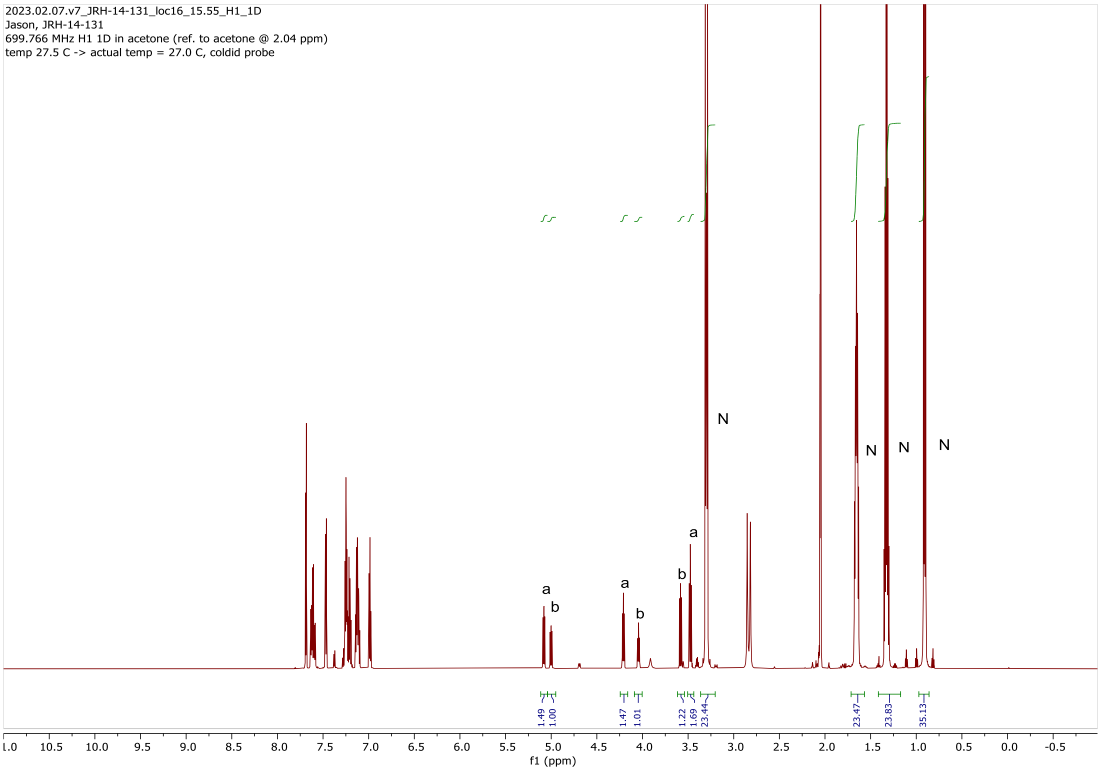

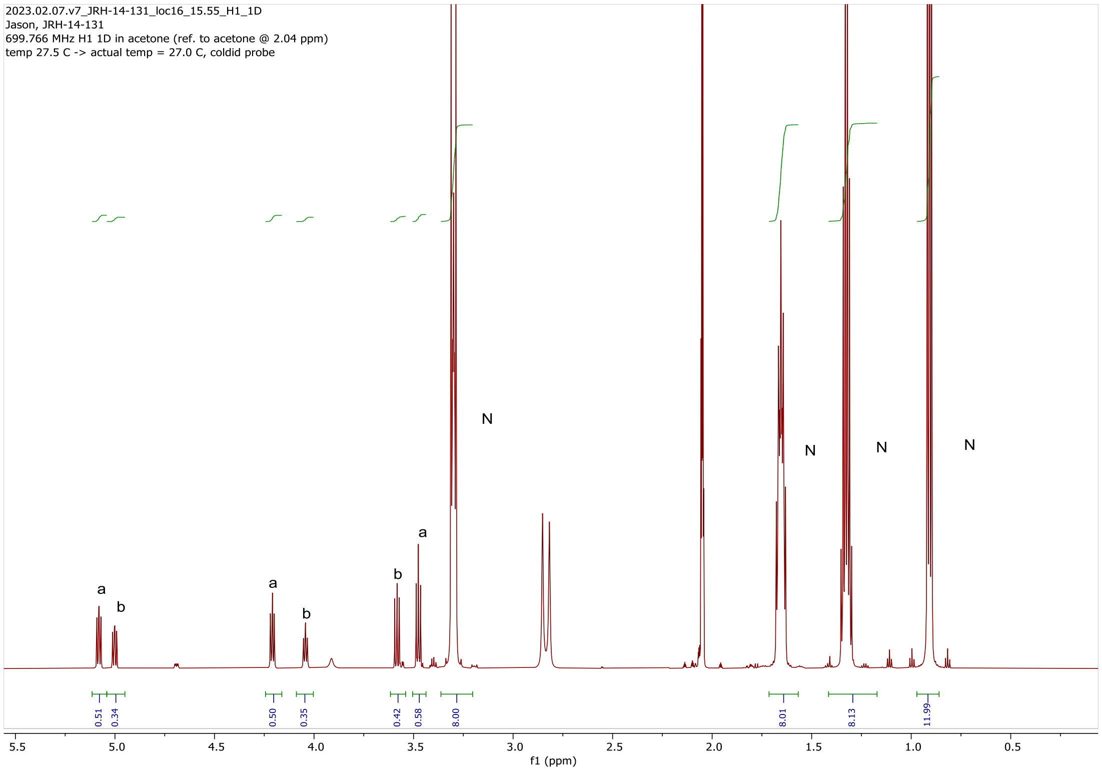

Supplementary Figure 6. ^{1}H NMR demonstrating formation of tetravalent borate **2-II** using tetra-*n*-butylammonium hydroxide as a base. An expansion of the region from 0.5–5.5 ppm is shown. Resonances are assigned the following labels: (a = diastereomer A, b = diastereomer B, N = tetra-*n*-butylammonium).

These observations suggest that tetravalent species **2-II** exists as a mixture of two diastereomers as shown below (only one enantiomer of each diastereomer is drawn), with a rate of interconversion that is dependent on the nature of the base.

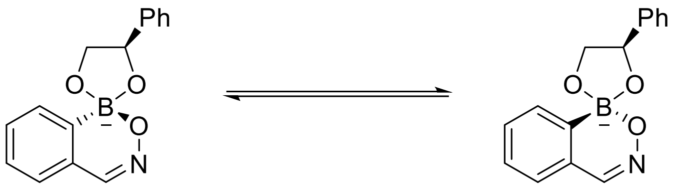

When using DIPEA as a base, as is employed under the optimized conditions for catalytic monophosphorylation, interconversion occurs at a moderate rate relative to the NMR timescale, and broad resonances are observed accordingly. Conversely, interconversion appears to be sufficiently slow using the tetraalkylammonium hydroxide base that the two species are both observed with well-defined resonances. The Brønsted acidity of the corresponding conjugate acid ($[HN(i\text{-}Pr)_2Et]^+$ vs H_2O) may play a significant role in the rate of conformational exchange.

4.3 Optimization of Catalytic Monophosphorylation (Figure 2b)

General Procedure for the monophosphorylation of diols using NMR yields (GP2)

A vial equipped with a stir bar was charged with diol **4a** (13.8 mg, 0.100 mmol), catalyst (10 mol%), MeCN (0.5 mL), base (X equiv) and diethyl chlorophosphate (Y equiv). The vial was then capped and stirred at room temperature for the indicated time, after which it was diluted with $CHCl_3$ and filtered through a small pipette of silica (approximately 1 cm high) with $CHCl_3$ washings to remove insoluble components. The mixture was concentrated by rotary evaporation, and yields were obtained by ^1H NMR relative to 1,3,5-trimethoxybenzene as an internal standard.

Supplementary Table 1. Optimization of catalytic monophosphorylation.

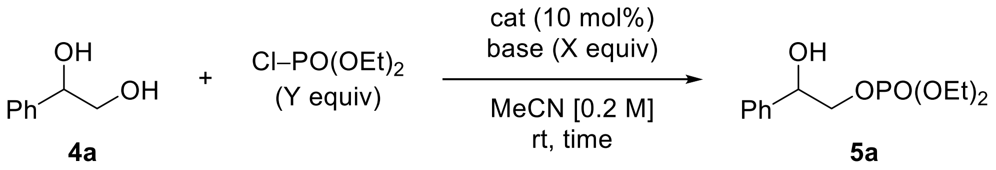

Entry	Catalyst	Base (X equiv)	Cl–PO(OEt)$_2$ (Y equiv)	Time	Yield 5a
1	**2**	NEt_3 (1.5 equiv)	1.5	18 h	70%
2	**2**	Pyridine (1.5 equiv)	1.5	18 h	43%
3	**2**	DBU (1.5 equiv)	1.5	18 h	12%
4	**2**	DIPEA (1.5 equiv)	1.5	18 h	73%
5	**2**	Na_2CO_3 (1.5 equiv)	1.5	18 h	64%
6	**2**	K_2HPO_4 (1.5 equiv)	1.5	18 h	34%
7	**2**	K_2CO_3 (1.5 equiv)	1.5	18 h	66%
8	**2**	Proton Sponge (1.5 equiv)	1.5	18 h	71%
9	**2**	DIPEA (1.5 equiv)	1.5	1 h	92%
10	**2**	DIPEA (1.5 equiv)	1.0	1 h	85%
11	**2**	DIPEA (1.0 equiv)	1.0	1 h	89%
12	**2**	DIPEA (1.1 equiv)	1.1	1.5 h	93%
13	**1a**	DIPEA (1.1 equiv)	1.1	1.5 h	13%
14	**1b**	DIPEA (1.1 equiv)	1.1	1.5 h	24%
15	**1c**	DIPEA (1.1 equiv)	1.1	1.5 h	6%
16	**3**	DIPEA (1.1 equiv)	1.1	1.5 h	11%
17	-	DIPEA (1.1 equiv)	1.1	1.5 h	4%

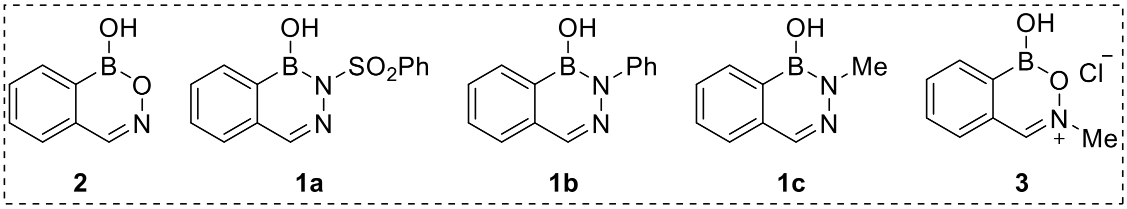

A control reaction using 4-bromobenzyl alcohol (**14b**) under the optimized conditions of Entry 12 led to significantly decreased yield of phosphorylated product relative to reaction of diol **4a**, highlighting the enhanced reactivity of chelated boronate **2 - II**.

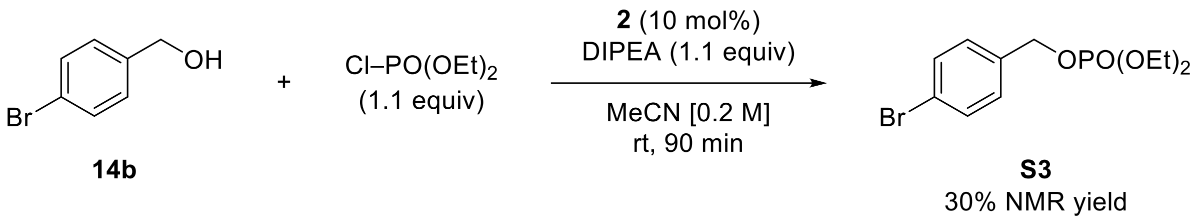

4.4 Diol Complexation Studies by ^{11}B NMR (Figure 3a)

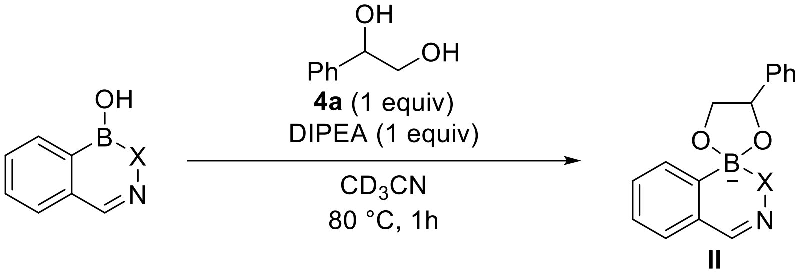

A vial was charged with diol **4a** (6.9, 0.050 mmol), boron heterocycle (1.0 equiv), DIPEA (9.6 µL, 1.0 equiv) and CD_3CN (0.70 mL). The vial was capped and heated to 80 °C in an aluminum heating block for one hour. After cooling to room temperature, the solution was transferred to a quartz NMR tube for ^{11}B NMR analysis. Conversion to the corresponding tetravalent adduct **II** was determined using relative integrations.

Heterocycle **1a** showed >99% conversion to the tetravalent adduct (6.1 ppm).

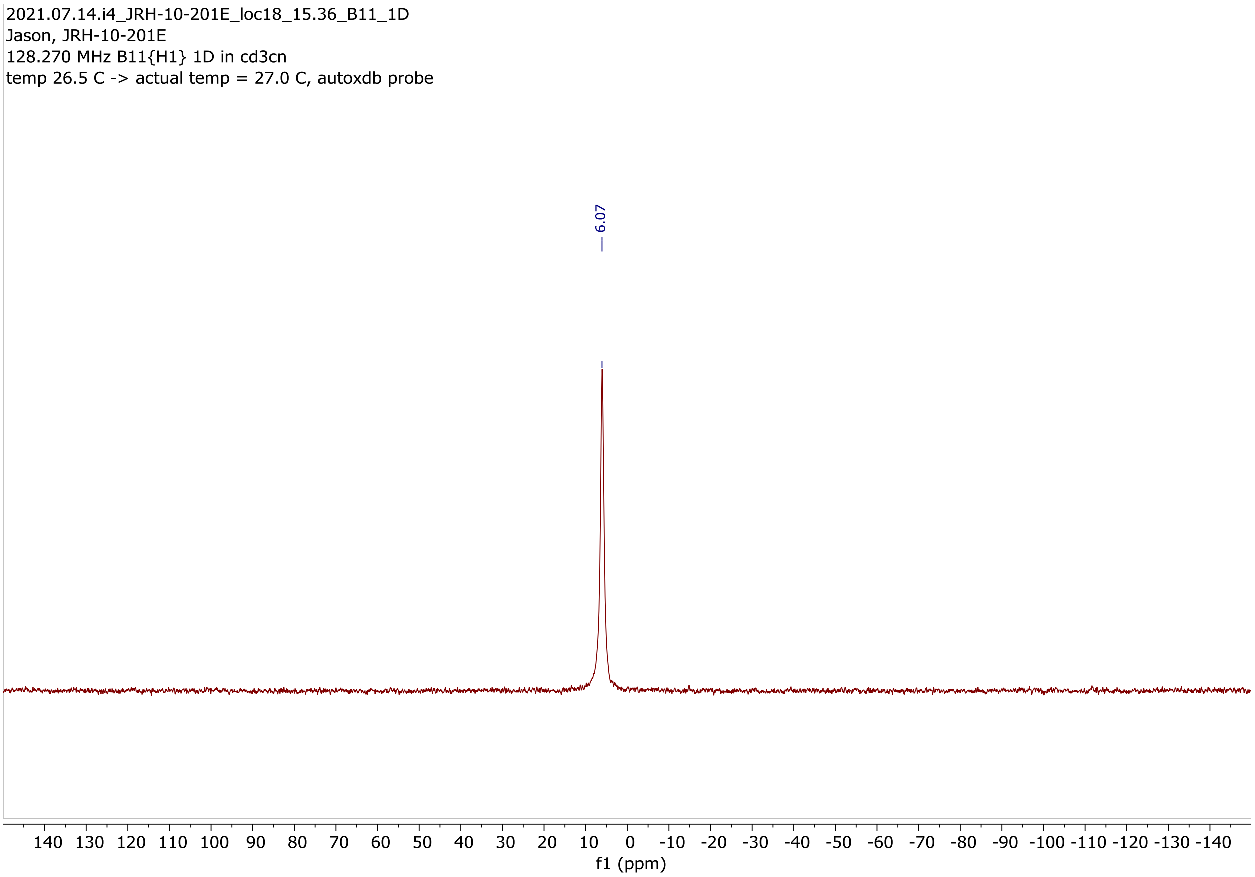

Supplementary Figure 7. ^{11}B NMR for tetravalent diol adduct formation from heterocycle **1a**. Heterocycle **1b** showed 13% conversion to the tetravalent adduct (6.9 ppm).

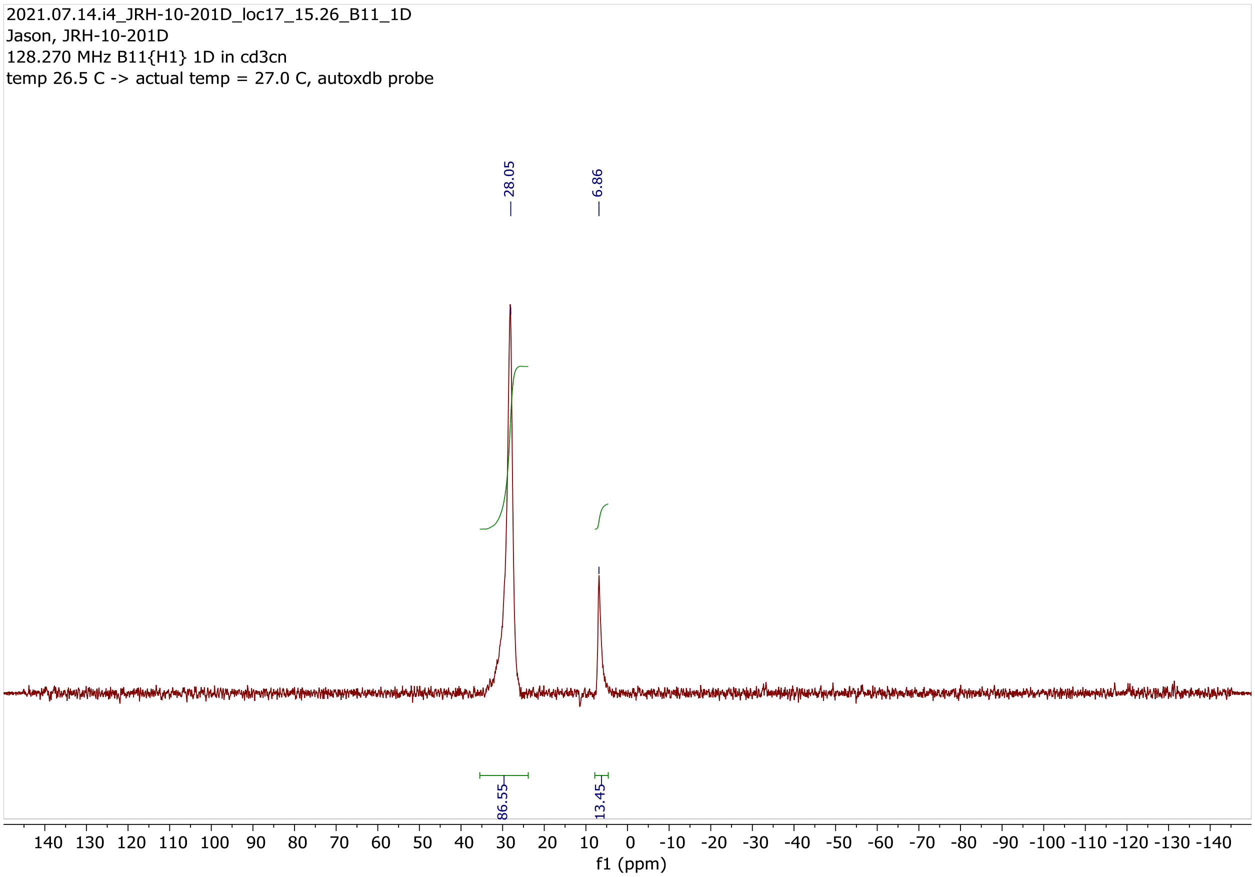

Supplementary Figure 8. ^{11}B NMR for tetravalent diol adduct formation from heterocycle **1b**. Heterocycle **1c** showed only trace conversion to the tetravalent adduct.

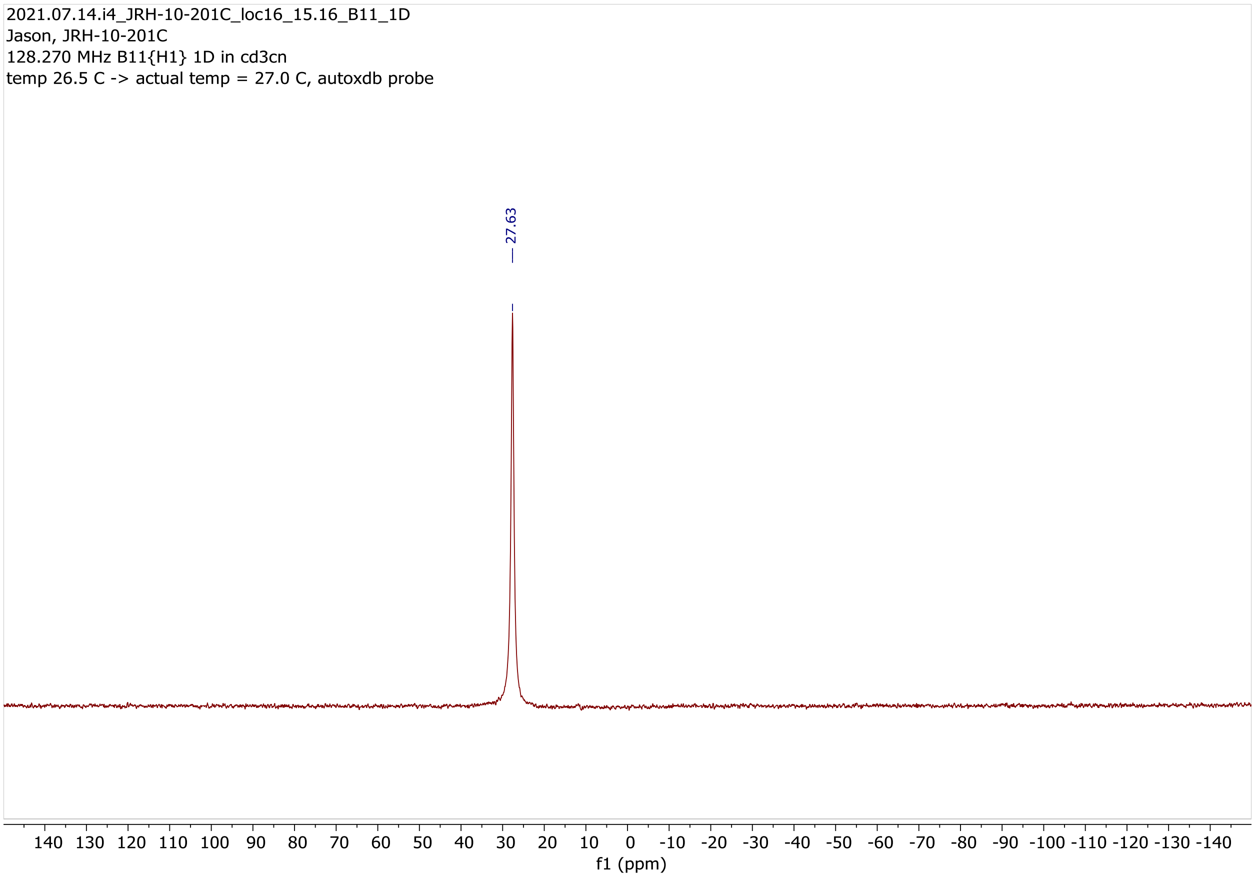

Supplementary Figure 9. ^{11}B NMR for tetravalent diol adduct formation from heterocycle **1c**. Heterocycle **2** showed 98% conversion to the tetravalent adduct (7.2 ppm).

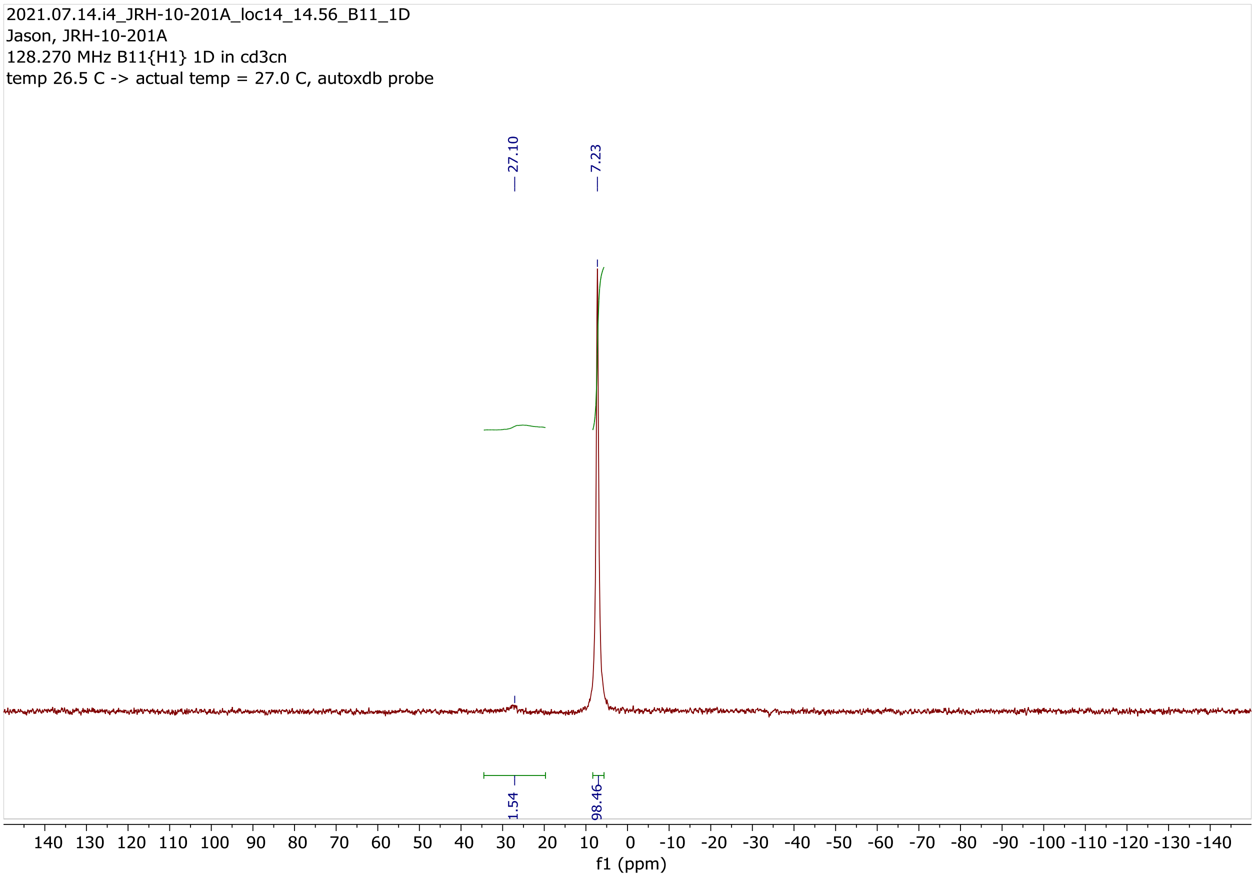

Supplementary Figure 10. ^{11}B NMR for tetravalent diol adduct formation from heterocycle **2**.

A clear correlation was observed between the pK_a of the parent hemiboronic acid[1] and conversion to the diol complex, where heterocycles with a lower pK_a (more acidic) showed increased conversion. As described in Section 4.1, the tetravalent adduct derived from heterocycle **2** (pK_a 7.1) was found to react rapidly with diethyl chlorophosphate and was fully consumed with 10 minutes at room temperature. In contrast, the tetravalent adduct derived from heterocycle **1a** (pK_a 5.5) reacted much slower with the electrophile. This suggests that the acidity of the boron heterocycle appears to be inversely correlated to the nucleophilicity of the corresponding tetravalent adduct.

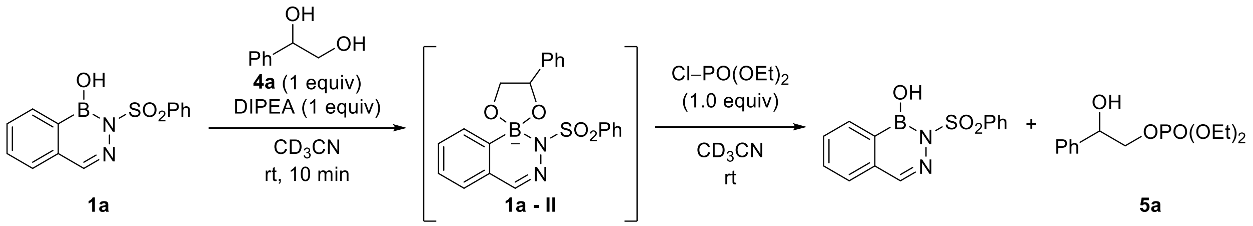

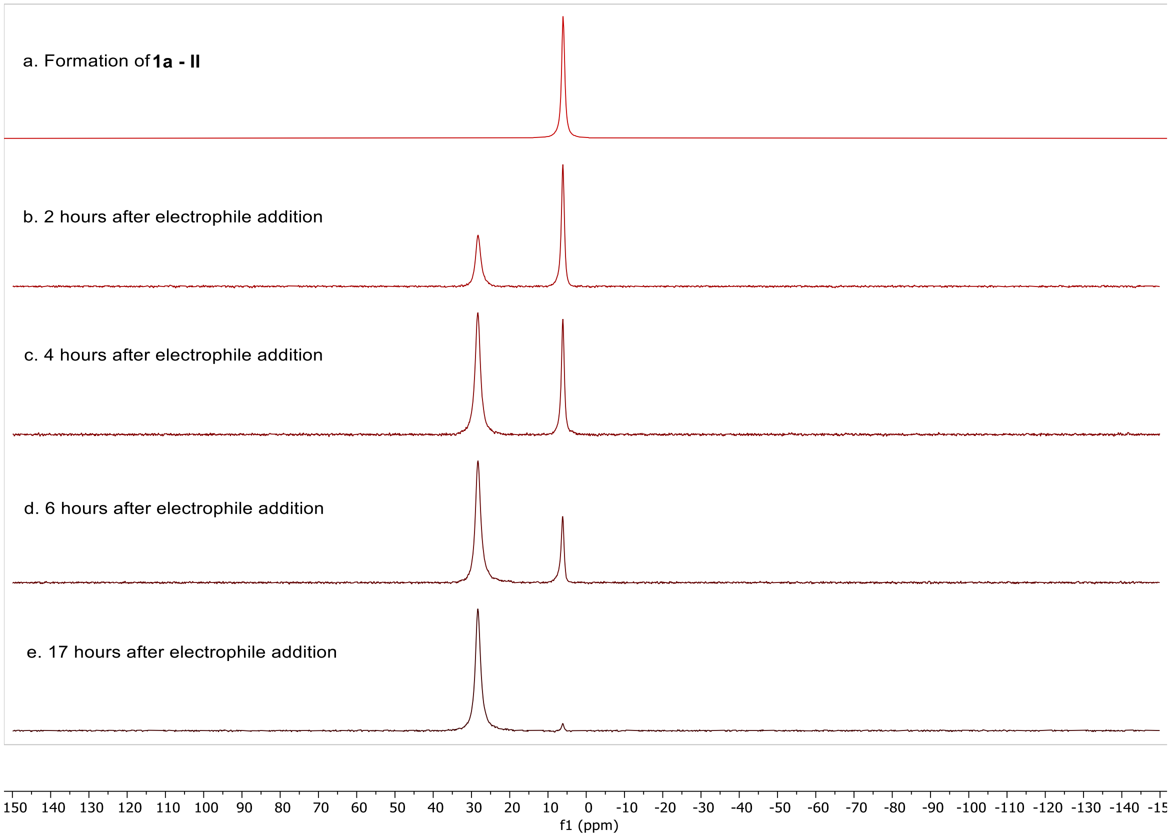

Supplementary Figure 11. Reaction of proposed adduct **1a – II** with $ClPO(OEt)_2$ as monitored by ^{11}B NMR.

The uniquely high catalytic activity of heterocycle **2** compared to heterocycles **1a**–**1c** likely originates from a balancing of being sufficiently Lewis acidic to rapidly generate the tetravalent adduct, while maintaining sufficient adduct nucleophilicity to rapidly react with the electrophile. Catalysts **1b** and **1c** are not acidic enough to form appreciable amounts of the active diol adduct, while the catalyst-substrate complex derived from heterocycle **1a** reacts too slowly with the electrophile.

5. Monophosphorylation – Substrate Scope (*cf.* Figure 3b)

General Procedure for the monophosphorylation of vicinal diols (GP3)

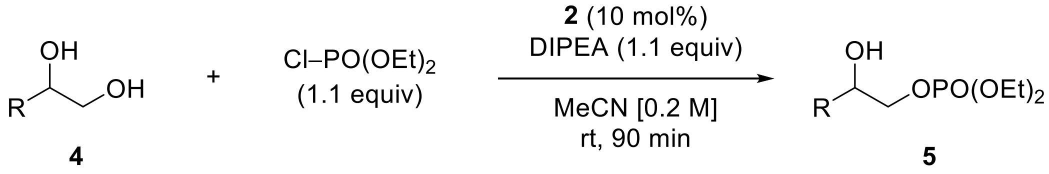

A two-dram vial with a stir bar was charged with diol **4** (1.0 equiv), catalyst **2** (10 mol%) and MeCN (0.2 M). The reaction was stirred for thirty seconds until fully dissolved, followed by

addition of DIPEA (1.1 equiv) and $ClPO(OEt)_2$ (1.1 equiv) (caution: addition of the electrophile is mildly exothermic). The vial was capped and stirred at room temperature for 1.5 hours. Upon completion, the reaction mixture was diluted with ethyl acetate (20 mL) and washed successively with 1 M $HCl_{(aq)}$ (10 mL), saturated $NaHCO_{3(aq)}$ (10 mL) and brine (10 mL). The organic layer was dried over Na_2SO_4, filtered, and concentrated by rotary evaporation. Purification by column chromatography afforded the desired product.

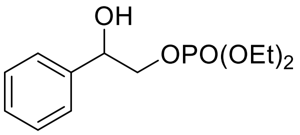

Diethyl (2-hydroxy-2-phenylethyl) phosphate (5a): Prepared according to **GP3** from 1-phenyl-1,2-ethanediol (83.0 mg, 0.600 mmol), catalyst **2** (8.8 mg, 0.060 mmol, 10 mol%), MeCN (3.0 mL), DIPEA (115 µL, 0.660 mmol, 1.10 equiv) and $ClPO(OEt)_2$ (96 µL, 0.66 mmol, 1.1 equiv). Purification by column chromatography (1:2 hexane/EtOAc) afforded the title compound as a clear oil (118 mg, 72%). ^{1}**H NMR** (500 MHz, $CDCl_3$): δ 7.39 (dd, *J* = 8.4, 1.7 Hz, 2 H), 7.36 (td, *J* = 7.6 Hz, 1.4 Hz, 2 H), 7.30 (tt, *J* = 7.4, 1.6 Hz, 1 H), 4.98 (dd, *J* = 8.3, 3.0 Hz, 1 H), 4.21 – 4.05 (m 6 H), 3.55 (br s, 1 H), 1.33 (tdd, *J* = 7.1, 6.1, 1.1 Hz, 6 H); ^{13}**C NMR** (126 MHz, $CDCl_3$): δ 139.3, 128.6, 128.3, 126.4, 73.2 (d, *J* = 5.4 Hz), 72.8 (d, *J* = 6.0 Hz), 64.3 (d, *J* = 5.9 Hz), 16.2 (d, *J* = 6.6 Hz); ^{31}**P NMR** (161 MHz, $CDCl_3$): δ – 0.1 (s); **FTIR** (Cast film, cm^{-1}): 3365 (br, m), 3031 (w), 2985 (w), 1454 (w), 1258 (m), 1030 (s), 984 (m), 702 (m); **HRMS** (ESI) for $C_{12}H_{19}PO_5Na$ $[M+Na]^+$: Calculated: 297.0868; Found: 297.0861.

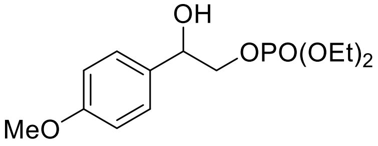

Diethyl (2-hydroxy-2-(4-methoxyphenyl)ethyl) phosphate (5b): Prepared according to **GP3** from diol **4b** (84.0 mg, 0.500 mmol), catalyst **2** (7.3 mg, 0.050 mmol, 10 mol%), MeCN (2.5 mL), DIPEA (96 µL, 0.55 mmol, 1.1 equiv) and $ClPO(OEt)_2$ (80 µL, 0.55 mmol, 1.1 equiv). Purification by column chromatography (1:2 hexane/EtOAc) afforded the title compound as a clear oil (97 mg, 64%). ^{1}**H NMR** (500 MHz, $CDCl_3$): δ 7.31 (d, *J* = 8.5 Hz, 2 H), 6.89 (d, *J* = 8.7 Hz, 2 H), 4.93 (dd, *J* = 8.4, 3.2 Hz, 1 H), 4.16 – 4.03 (m, 6 H), 3.80 (s, 3 H), 3.40 (br s, 1 H), 1.34 (tdd, *J* = 7.1, 4.3, 1.0 Hz, 6 H); ^{13}**C NMR** (126 MHz, $CDCl_3$): δ 159.7, 131.3, 127.6, 114.1, 72.8 (d, *J* = 5.7 Hz), 72.7 (d, *J* = 6.5 Hz), 64.3 (d, *J* = 5.9 Hz), 55.4, 16.2 (d, *J* = 6.7 Hz); ^{31}**P NMR** (162 MHz, $CDCl_3$): δ – 0.1 (s) (note: sample contains approximately 4% excess $ClPO(OEt)_2$, observed at –13.2 ppm); **FTIR** (Cast film, cm^{-1}): 3367 (br, m), 2985 (w), 2946 (w), 1613 (w), 1515 (m), 1250 (s), 1031 (s), 833 (w); **HRMS** (ESI) for $C_{13}H_{21}PO_6Na$ $[M+Na]^+$: Calculated: 327.0973; Found: 327.0969.

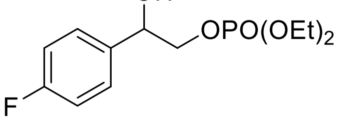

Diethyl (2-(4-fluorophenyl)-2-hydroxyethyl) phosphate (5c): Prepared according to **GP3** from diol **4c** (93.8 mg, 0.600 mmol), catalyst **2** (8.8 mg, 0.060 mmol, 10 mol%), MeCN (3.0 mL), DIPEA (116 µL, 0.660 mmol, 1.10 equiv) and $ClPO(OEt)_2$ (96 µL, 0.66 mmol, 1.1 equiv). Purification by column chromatography (1:2 hexane/EtOAc) afforded the title compound as a clear oil (134 mg, 77%). **^{1}H NMR** (500 MHz, $CDCl_3$): δ 7.40 – 7.34 (dd, *J* = 8.2, 5.3 Hz, 2 H), 7.04 (app t, *J* = 8.7 Hz, 2 H), 4.97 (dt, *J* = 8.1, 3.1 Hz, 1 H), 4.20 – 4.03 (m, 6 H), 3.69 (d, *J* = 3.3 Hz, 1 H), 1.34 (tdd, *J* = 7.2, 6.2, 1.0 Hz, 6 H); **^{13}C NMR** (126 MHz, $CDCl_3$): δ 162.7 (d, *J* = 246.0 Hz), 135.1 (d, *J* = 3.0 Hz), 128.1 (d, *J* = 8.1 Hz), 115.5 (d, *J* = 21.5 Hz), 72.6 (d, *J* = 6.3 Hz), 72.58 (d, *J* = 5.0 Hz), 64.4 (d, *J* = 5.9 Hz), 16.2 (d, *J* = 6.5 Hz); **^{31}P NMR** (202 MHz, $CDCl_3$): δ – 0.02 (s) (contains trace excess $ClPO(OEt)_2$ at –13.2 ppm); **^{19}F NMR** (376 MHz, $CDCl_3$): δ – 114.2 (tt, *J* = 11.6, 5.7 Hz) ; **FTIR** (Cast film, cm^{-1}): 3357 (br, m), 3066 (w), 2985 (w), 1442 (w), 1256 (s), 1033 (s), 757 (w) ; **HRMS** (ESI) for $C_{12}H_{18}PO_5FNa$ $[M+Na]^+$: Calculated: 315.0774; Found: 315.0767.

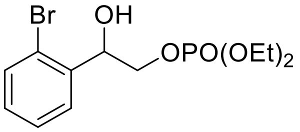

2-(2-Bromophenyl)-2-hydroxyethyl diethyl phosphate (5d): Prepared according to **GP3** from diol **4d** (109 mg, 0.500 mmol), catalyst **2** (7.3 mg, 0.050 mmol, 10 mol%), MeCN (2.5 mL), DIPEA (96 µL, 0.55 mmol, 1.1 equiv) and $ClPO(OEt)_2$ (80 µL, 0.55 mmol, 1.1 equiv). Purification by column chromatography (1:2 hexane/EtOAc) afforded the title compound as a clear oil (142 mg, 80%). **^{1}H NMR** (500 MHz, $CDCl_3$): δ 7.66 (dd, *J* = 7.8, 1.7 Hz, 1 H), 7.52 (dd, *J* = 8.0, 1.2 Hz, 1 H), 7.35 (td, *J* = 7.6, 1.3 Hz, 1 H), 7.17 (td, *J* = 7.7, 1.8 Hz, 1 H), 5.33 (dt, *J* = 8.0, 3.0 Hz, 1 H), 4.29 (td, *J* = 11.0, 2.5 Hz, 1 H), 4.22 – 4.08 (m, 4 H), 4.01 (ddd, *J* = 11.4, 9.2, 8.0 Hz, 1 H), 3.97 (d, *J* = 3.5 Hz, 1 H), 1.35 (ddd, *J* = 14.8, 7.6, 6.6 Hz, 6 H); **^{13}C NMR** (126 MHz, $CDCl_3$): δ 138.3, 132.8, 129.7, 128.5, 127.9, 122.1, 72.4 (d, *J* = 4.7 Hz), 71.2 (d, *J* = 6.0 Hz), 64.5 (d, *J* = 3.9 Hz), 64.4 (d, *J* = 4.0 Hz), 16.3 (d, *J* = 2.5 Hz), 16.2 (d, *J* = 2.6 Hz); **^{31}P NMR** (162 MHz, $CDCl_3$): δ 0.3 (s); **FTIR** (Cast film, cm^{-1}): 3363 (br, m), 2985 (m), 1605 (m), 1510 (s), 1395 (w), 1253 (s), 1221 (s), 1022 (s), 836 (m), 739 (w) ; **HRMS** (ESI) for $C_{12}H_{18}PO_5BrNa$ $[M+Na]^+$: Calculated: 374.9973; Found: 374.9969.

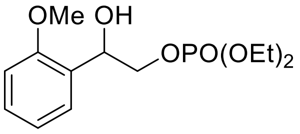

Diethyl (2-hydroxy-2-(2-methoxyphenyl)ethyl) phosphate (5e): Prepared according to **GP3** from diol **4e** (101 mg, 0.600 mmol), catalyst **2** (8.8 mg, 0.060 mmol, 10 mol%), MeCN (3.0 mL), DIPEA (116 µL, 0.660 mmol, 1.10 equiv) and $ClPO(OEt)_2$ (96 µL, 0.66 mmol, 1.1 equiv). Purification by column chromatography (1:2 hexane/EtOAc) afforded the title compound as a clear oil (133 mg, 73%). **^{1}H NMR** (500 MHz, $CDCl_3$): δ 7.48 (dd, *J* = 7.6, 1.7 Hz, 1 H), 7.27 (td, *J* = 7.8 Hz, 1 H (integral inflated by overlap with $CHCl_3$ residual signal), 6.98 (td, *J* = 7.5, 1.1 Hz, 1 H), 6.86 (dd, *J* = 8.3, 1.0 Hz, 1 H), 5.23 (dd, *J* = 7.9, 2.9 Hz, 1 H), 4.25 (ddd, *J* = 10.9, 9.5, 3.0 Hz, 1 H), 4.16 – 4.05 (m, 5 H), 3.84 (s, 3 H), 3.61 (br s, 1 H), 1.33 (dtd, *J* = 11.8, 7.1, 1.0 Hz, 7 H); **^{13}C NMR** (126 MHz, $CDCl_3$): δ 156.3, 129.1, 127.6, 127.3, 120.9, 110.3, 71.4 (d, *J* = 6.0 Hz),

69.2 (d, J = 5.6 Hz), 64.21 (d, J = 2.4 Hz), 64.16 (d, J = 2.5 Hz), 55.4, 16.3 (d, J = 1.6 Hz), 16.2 (d, J = 1.6 Hz); **^{31}P NMR** (162 MHz, $CDCl_3$): δ 0.03 (s) ; **FTIR** (Cast film, cm^{-1}): 3375 (br, m), 2985 (m), 1602 (w), 1491 (m), 1241 (s), 1026 (s), 985 (m), 756 (w); **HRMS** (ESI) for $C_{13}H_{21}PO_6Na$ $[M+Na]^+$: Calculated: 327.0973; Found: 327.0970.

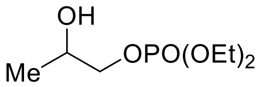

Diethyl (2-hydroxypropyl) phosphate (5f): Prepared according to **GP3** from diol **4f** (114 mg, 1.50 mmol), catalyst **2** (21.8 mg, 0.150 mmol, 10 mol%), MeCN (7.5 mL), DIPEA (288 µL, 1.65 mmol, 1.10 equiv) and $ClPO(OEt)_2$ (240 µL, 1.65 mmol, 1.10 equiv) with a reaction time of 3 hours. Purification by column chromatography (EtOAc) afforded the title compound as a clear oil (155 mg, 49%). **^{1}H NMR** (500 MHz, $CDCl_3$): δ 4.17 – 4.11 (m, 4 H), 4.06 – 3.99 (m, 2 H), 3.87 (ddd, J = 10.7, 8.8, 7.5 Hz, 1 H), 2.90 (d, J = 3.7 Hz, 1 H), 1.35 (tt, J = 7.1, 1.1 Hz, 6 H), 1.19 (d, J = 6.4 Hz, 3 H) ; **^{13}C NMR** (126 MHz, $CDCl_3$): δ 73.0 (d, J = 6.0 Hz), 66.8 (d, J = 5.6 Hz), 64.2 (d, J = 5.9 Hz), 18.5, 16.3 (d, J = 6.6 Hz); **^{31}P NMR** (202 MHz, $CDCl_3$): δ – 0.1 (s) (trace contamination at – 0.4 ppm); **FTIR** (Cast film, cm^{-1}): 3410 (br, w), 2983 (w), 1262 (m), 1165 (w), 1035 (s), 980 (w), 820 (w); **HRMS** (ESI) for $C_7H_{17}PO_5Na$ $[M+Na]^+$: Calculated: 235.0711; Found: 235.0708.

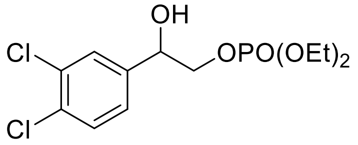

2-(3,4-Dichlorophenyl)-2-hydroxyethyl diethyl phosphate (5g): Prepared according to **GP3** from diol **4g** (123 mg, 0.600 mmol), catalyst **2** (8.8 mg, 0.060 mmol, 10 mol%), MeCN (3.0 mL), DIPEA (116 µL, 0.660 mmol, 1.10 equiv) and $ClPO(OEt)_2$ (96 µL, 0.66 mmol, 1.1 equiv). Purification by column chromatography (1:2 hexane/EtOAc) afforded the title compound as a clear oil (159 mg, 77%). **^{1}H NMR** (500 MHz, $CDCl_3$): δ 7.53 (d, J = 2.0 Hz, 1 H), 7.43 (d, J = 8.3 Hz, 1 H), 7.23 (dd, J = 8.2, 2.0 Hz, 1 H), 4.95 (dt, J = 7.1, 3.3 Hz, 1H), 4.18 – 4.02 (m, 6 H), 3.97 (d, J = 3.6 Hz, 1 H), 1.34 (dtd, J = 8.2, 7.1, 1.0 Hz, 6 H); **^{13}C NMR** (126 MHz, $CDCl_3$): δ 139.7, 132.9, 132.2, 130.6, 128.5, 125.7, 72.3 (d, J = 5.9 Hz), 72.1 (d, J = 4.6 Hz), 64.6 (d, J = 6.0 Hz), 16.3 (d, J = 1.6 Hz), 16.2 (d, J = 1.7 Hz); **^{31}P NMR** (202 MHz, $CDCl_3$): δ 0.2 (s); **FTIR** (Cast film, cm^{-1}): 3349 (br, m), 2984 (w), 1565 (w), 1469 (m), 1393 (m), 1249 (m), 1024 (s), 819 (w); **HRMS** (ESI) for $C_{12}H_{17}PO_5Cl_2Na$ $[M+Na]^+$: Calculated: 365.0088; Found: 365.0081.

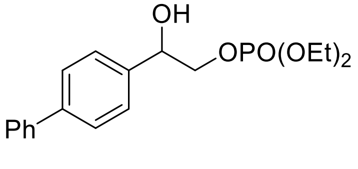

2-([1,1'-Biphenyl]-4-yl)-2-hydroxyethyl diethyl phosphate (5h): Prepared according to **GP3** from diol **4h** (107 mg, 0.500 mmol), catalyst **2** (7.3 mg, 0.050 mmol, 10 mol%), MeCN (2.5 mL), DIPEA (96 µL, 0.55 mmol, 1.1 equiv) and $ClPO(OEt)_2$ (80 µL, 0.55 mmol, 1.1 equiv). Purification by column chromatography afforded the title compound as a viscous clear oil (105 mg, 60%). **^{1}H**

NMR (500 MHz, $CDCl_3$): δ 7.61 – 7.57 (t, *J* = 8.0 Hz, 4 H), 7.48 (d, *J* = 8.2 Hz, 2 H), 7.44 (t, *J* = 7.7 Hz, 2 H), 7.35 (tt, *J* = 7.5, 1.2 Hz, 1 H), 5.04 (dt, *J* = 8.1, 3.2 Hz, 1 H), 4.22 (ddd, *J* = 11.1, 9.7, 3.1 Hz, 1 H), 4.18 – 4.09 (m, 5 H), 3.63 (d, *J* = 3.3 Hz, 1 H), 1.35 (qd, *J* = 7.0, 1.0 Hz, 6 H); ^{13}**C NMR** (126 MHz, $CDCl_3$): δ 141.2, 140.8, 138.3, 128.9, 127.5, 127.4, 127.2, 126.8, 73.0 (d, *J* = 5.2 Hz), 72.7 (d, *J* = 6.0 Hz), 64.4 (d, *J* = 5.9 Hz), 16.2 (d, *J* = 6.6 Hz); ^{31}**P NMR** (202 MHz, $CDCl_3$): δ – 0.02 (s); **FTIR** (Cast film, cm^{-1}): 3377 (br, m), 3029 (w), 2984 (w), 1601 (w), 1487 (w), 1444 (w), 1258 (m), 1029 (s), 767 (w); **HRMS** (ESI) for $C_{18}H_{23}PO_5Na$ $[M+Na]^+$: Calculated: 373.1181; Found: 373.1174.

6. Synthesis and Characterization of Benzylic Alcohols and Ketones

6.1 Synthesis and Characterization of Benzylic Alcohols

General Procedure for the synthesis of benzylic alcohols from ketones via reduction (GP4)

A round bottom flask equipped with a stir bar was charged with ketone (1.0 equiv) and MeOH (0.2 M) and cooled in an ice bath to 0 °C, after which $NaBH_4$ (1.5 equiv) was added as a solid in two portions. The reaction mixture was stirred at 0 °C for 30 minutes before removing the ice bath and stirring for an additional 3 hours. The reaction was quenched by addition of saturated $NH_4Cl_{(aq)}$, and methanol was removed by rotary evaporation. The mixture was extracted with EtOAc (3 × 25 mL). The combined organic phases were washed with H_2O (35 mL) and brine (35 mL), dried over Na_2SO_4, filtered, and concentrated by rotary evaporation. If necessary, purification by column chromatography was conducted to afford the desired alcohol.

General Procedure for the synthesis of benzylic alcohols via Grignard addition of PhMgBr (GP5)

Under nitrogen, a flame-dried round bottom flask equipped with stir bar was charged with carbonyl compound (1.0 equiv) and THF (0.5 M). The solution was cooled in an ice bath to 0 °C, after which PhMgBr (1.5 equiv, 3.0 M solution in Et_2O) was added dropwise. The reaction was stirred at 0 °C for 30 minutes, at which point the cooling bath was removed and stirring continued for an additional 4 hours. The reaction was quenched by addition of saturated $NH_4Cl_{(aq)}$. The mixture was extracted with EtOAc (3 × 25 mL). The combined organic phases were washed with H_2O (35 mL) and brine (35 mL), dried over Na_2SO_4, filtered, and concentrated by rotary evaporation. Purification by column chromatography was conducted to afford the desired alcohol.

Benzylic alcohols **6a**, **6p** and **6r** were purchased from commercial suppliers and used as received.

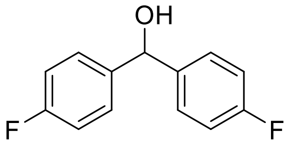

Bis(4-Fluorophenyl)methanol (6b): Prepared according to **GP4** from 4,4'-difluorobenzophenone (873 mg, 4.00 mmol). Purification by column chromatography (8:1 hexane/EtOAc) afforded the title compound as a white solid (865 mg, 99%). ^{1}**H NMR** (500 MHz, $CDCl_3$): δ 7.32 (dd, *J* = 8.6,

5.5 Hz, 4 H), 7.03 (app t, *J* = 8.7 Hz, 4 H), 5.82 (d, *J* = 3.5 Hz, 1 H), 2.20 (d, *J* = 3.5 Hz, 1 H); **^{13}C NMR** (126 MHz, $CDCl_3$): δ 162.4 (d, *J* = 246.4 Hz), 139.6 (d, *J* = 3.1 Hz), 128.3 (d, *J* = 8.3 Hz), 115.6 (d, *J* = 21.4 Hz), 75.1; **^{19}F NMR** (469 MHz, $CDCl_3$): δ – 114.7 (ddd, *J* = 13.8, 8.9, 5.3 Hz). Spectral data were in agreement with the literature.[6]

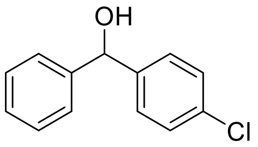

(4-Chlorophenyl)(phenyl)methanol (6c): Prepared according to **GP4** from 4-chlorobenzophenone (867 mg, 4.00 mmol). Purification by column chromatography (5:1 hexane/EtOAc) afforded the title compound as a white solid (756 mg, 86%). **^{1}H NMR** (500 MHz, $CDCl_3$): δ 7.35 (d, *J* = 4.4 Hz, 4 H), 7.33 – 7.27 (m, 5 H), 5.81 (d, *J* = 3.5 Hz, 1 H), 2.25 (d, *J* = 3.5 Hz, 1 H); **^{13}C NMR** (126 MHz, $CDCl_3$): δ 143.6, 142.4, 133.4, 128.80, 128.75, 128.0 (according to literature data, there are two overlapping carbon resonances here separated by 0.01 ppm, but separation was not observed here), 126.7, 75.8. Spectral data were in agreement with the literature.[7]

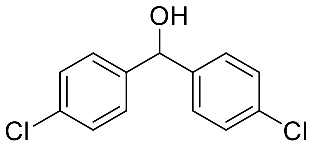

Bis(4-Chlorophenyl)methanol (6d): Prepared according to **GP4** from 4,4'-dichlorobenzophenone (1.00 g, 4.00 mmol). Purification by column chromatography (8:1 hexane/EtOAc) afforded the title compound as a white solid (820 mg, 81%). **^{1}H NMR** (600 MHz, $CDCl_3$): δ 7.32 (d, *J* = 8.6 Hz, 4 H), 7.29 (d, *J* = 8.6 Hz, 4 H), 5.79 (d, *J* = 2.9 Hz, 1 H), 2.24 (d, *J* = 3.1 Hz, 1 H); **^{13}C NMR** (151 MHz): δ 142.0, 133.8, 128.9, 128.0, 75.1. Spectral data were in agreement with the literature.[8]

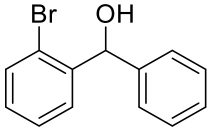

(2-Bromophenyl)(phenyl)methanol (6e): Prepared according to **GP4** from 2-bromobenzophenone (1.04 g, 4.00 mmol). Purification by column chromatography (5:1 hexane/EtOAc) afforded the title compound as a white solid (838 mg, 80%). **^{1}H NMR** (600 MHz, $CDCl_3$): δ 7.59 (dd, *J* = 7.8, 1.7 Hz, 1 H), 7.54 (dd, *J* = 8.0, 1.3 Hz, 1 H), 7.42 – 7.40 (m, 2 H), 7.36 – 7.33 (m, 3 H), 7.29 (tt, *J* = 7.3, 1.2 Hz 1 H), 7.15 (td, *J* = 7.7, 1.7 Hz, 1 H), 6.21 (d, *J* = 3.8 Hz, 1 H), 2.38 (d, *J* = 3.9 Hz, 1 H); **^{13}C NMR** (126 MHz, $CDCl_3$): δ 142.7, 142.3, 133.0, 129.2, 128.64, 128.62, 127.91, 127.86, 127.2, 123.0, 74.9. Spectral data were in agreement with the literature.[9]

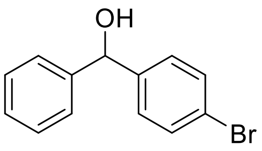

(4-Bromophenyl)(phenyl)methanol (6f): Prepared according to **GP4** from 4-bromobenzophenone (2.61 g, 10.0 mmol). Purification by column chromatography (5:1 hexane/EtOAc) afforded the title compound as a white solid (2.21 g, 84%). **^{1}H NMR** (600 MHz, $CDCl_3$): δ 7.46 (d, *J* = 8.5 Hz, 2 H), 7.36 – 7.33 (m, 4 H), 7.31 – 7.28 (m, 1 H), 7.25 (d, *J* = 8.3 Hz, 2 H), 5.76 (d, *J* = 3.2 Hz, 1 H), 2.42 (d, *J* = 3.4 Hz, 1 H); **^{13}C NMR** (151 MHz, $CDCl_3$): δ 143.5, 142.8, 131.7, 128.8, 128.3, 128.0, 126.6, 121.5, 75.7. Spectral data were in agreement with the literature.[10]

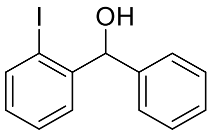

(2-Iodophenyl)(phenyl)methanol (6g): Prepared according to **GP4** from 2-iodobenzophenone (2.46 g, 8.00 mmol) afforded the title compound as a yellow oil (2.13 g, 86%). **^{1}H NMR** (600 MHz, $CDCl_3$): δ 7.84 (dd, *J* = 7.9, 1.3 Hz, 1 H), 7.53 (dd, *J* = 7.8, 1.7 Hz, 1 H), 7.42 – 7.34 (m, 5 H), 7.29 (tt, *J* = 7.4, 1.4 Hz, 1 H), 7.00 (td, *J* = 7.6, 1.7 Hz, 1 H), 6.06 (d, *J* = 3.7 Hz, 1H), 2.47 (br s, 1 H); **^{13}C NMR** (151 MHz, $CDCl_3$): δ 145.5, 142.2, 139.7, 129.6, 128.7, 128.6, 128.5, 127.9, 127.3, 98.8, 79.1. Spectral data were in agreement with the literature.[11]

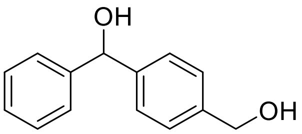

(4-(Hydroxymethyl)phenyl)(phenyl)methanol (6h): Under nitrogen, a flame-dried round bottom flask equipped with stir bar was charged with 4-bromobenzyl alcohol (1.12 g, 6.00 mmol, 1.00 equiv) and THF (18 mL). The flask was cooled to – 78 °C in a dry ice/acetone bath, at which point *n*-butyllithium (4.9 mL, 2.6 M in hexanes, 13 mmol, 2.1 equiv) was added dropwise. The reaction was stirred at – 78 °C for an additional one hour, at which point a solution of benzaldehyde (735 μL, 7.20 mmol, 1.20 equiv) in THF (15 mL) was added dropwise. The reaction was stirred at – 78 °C for an additional one hour, after which the cooling bath was removed and the reaction was stirred for an additional 2 hours while warming to room temperature. The reaction was then cooled to 0 °C and quenched with saturated $NH_4Cl_{(aq)}$ (30 mL) and extracted with EtOAc (3 × 25 mL). The combined organic layers were washed with brine (35 mL), dried over Na_2SO_4, filtered, and concentrated by rotary evaporation. Recrystallization using CH_2Cl_2/hexane afforded the title compound as a white solid (530 mg, 68%). **^{1}H NMR** (600 MHz, $CDCl_3$): δ 7.37 – 7.36 (m, 4 H), 7.34 – 7.30 (m, 4 H), 7.26 (tt, *J* = 6.5, 1.5 Hz, 1 H), 5.83 (s, 1 H), 4.64 (s, 2 H), 1.76 (br s, 2 H); **^{13}C NMR** (151 MHz): δ 143.9, 143.4, 140.3, 128.7, 127.8, 127.3, 126.9, 126.6, 76.2, 65.2. Spectral data were in agreement with the literature.[7]

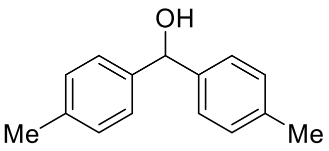

Di-p-tolylmethanol (6i): Prepared according to **GP4** from 4,4'-dimethylbenzophenone (841 mg, 4.00 mmol). Purification by column chromatography (4:1 hexane/EtOAc) afforded the title compound as a white solid (504 mg, 59%). **^{1}H NMR** (500 MHz, $CDCl_3$): δ 7.26 (d, *J* = 8.0 Hz, 4 H), 7.14 (d, *J* = 7.8 Hz, 4 H), 5.79 (d, *J* = 3.0 Hz, 1 H), 2.33 (s, 6 H), 2.14 (d, *J* = 3.3 Hz, 1 H); **^{13}C NMR** (126 MHz, $CDCl_3$): δ 141.3, 137.3, 129.3, 126.6, 76.1, 21.2. Spectral data were in agreement with the literature.[12]

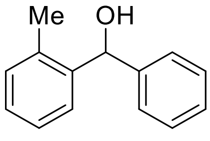

Phenyl(o-tolyl)methanol (6j): Prepared according to **GP4** from 2-methylbenzophenone (1.81 mL, 10.0 mmol). Purification by column chromatography (8:1 hexane/EtOAc) afforded the title compound as a white solid (1.53 g, 77%). **^{1}H NMR** (500 MHz, $CDCl_3$): δ 7.53 (dd, *J* = 7.6, 1.6 Hz, 1 H), 7.34 (d, *J* = 4.4 Hz, 4 H), 7.30 – 7.24 (m, 2 H), 7.22 (td, *J* = 7.4, 1.6 Hz, 1 H), 7.16 (d, *J* = 6.8 Hz, 1 H), 6.02 (d, *J* = 3.3 Hz, 1 H), 2.27 (s, 3 H), 2.15 (d, *J* = 3.6 Hz, 1 H); **^{13}C NMR** (126 MHz, $CDCl_3$): δ 143.0, 141.6, 135.5, 130.7, 128.6, 127.71, 127.68, 127.2, 126.4, 126.3. Spectral data were in agreement with the literature.[7]

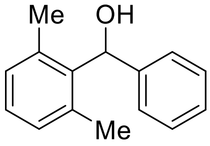

(2,6-Dimethylphenyl)(phenyl)methanol (6k): Prepared according to **GP5** from 2,6-dimethylbenzaldehyde (537 mg, 4.00 mmol). Purification by column chromatography (10:1 hexane/EtOAc) afforded the title compound as a white solid (734 mg, 86%). **^{1}H NMR** (500 MHz, $CDCl_3$): δ 7.33 – 7.22 (m, 5 H), 7.14 (dd, *J* = 8.1, 6.9 Hz, 1H), 7.05 (d, *J* = 7.5 Hz, 2 H), 6.38 (d, *J* = 4.2 Hz, 1 H), 2.29 (s, 6 H), 2.19 (d, *J* = 4.2 Hz, 1 H); **^{13}C NMR** (126 MHz, $CDCl_3$): δ 143.0, 139.5, 137.3, 129.5, 128.3, 127.9, 126.7, 125.6, 71.4, 20.8. Spectral data were in agreement with the literature.[13]

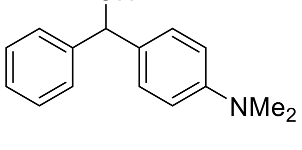

(4-(Dimethylamino)phenyl)(phenyl)methanol (6l): Prepared according to **GP5** from 4-(dimethylamino)benzaldehyde (597 mg, 4.00 mmol). Purification by column chromatography (gradient 7:1 to 4:1 hexane/EtOAc) afforded the title compound as a yellow oil that solidifies in the freezer to a white solid (795 mg, 88%). **^{1}H NMR** (600 MHz, $CDCl_3$): δ 7.40 (d, *J* = 7.7 Hz, 2

H), 7.33 (td, J = 7.6, 1.6 Hz, 2 H), 7.26 – 7.20 (m, 3 H), 6.70 (d, J = 8.8 Hz, 2 H), 5.78 (s, 1 H), 2.93 (s, 2 H), 2.10 (br s, 1 H); **^{13}C NMR** (151 MHz, $CDCl_3$): δ 150.3, 144.4, 132.1, 128.4, 127.9, 127.3, 126.5, 112.6, 76.1, 40.7. Spectral data were in agreement with the literature.[14]

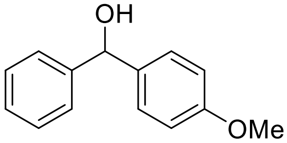

(4-Methoxyphenyl)(phenyl)methanol (6m): Prepared according to **GP4** from 4-methoxybenzophenone (1.27 g, 6.0 mmol). Purification by column chromatography (2:1 hexane/EtOAc) afforded the title compound as a white solid (1.02 g, 78%). **^{1}H NMR** (500 MHz, $CDCl_3$): δ 7.39 – 7.32 (m, 4 H), 7.31 – 7.25 (m, 3 H), 6.87 (d, J = 8.7 Hz, 2 H), 5.81 (d, J = 3.5 Hz, 1 H), 3.80 (s, 3 H), 2.17 (d, J = 3.5 Hz, 1 H); **^{13}C NMR** (126 MHz): δ 159.2, 144.2, 136.3, 128.6, 128.0, 127.6, 126.5, 114.0, 76.0, 55.4. Spectral data were in agreement with the literature.[7]

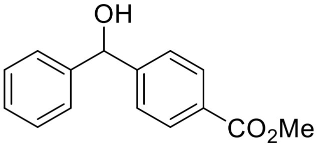

Methyl 4-(hydroxy(phenyl)methyl)benzoate (6n): Prepared according to **GP5** from methyl 4-formylbenzoate (657 mg, 4.00 mmol). Purification by column chromatography (4:1 hexane/EtOAc) afforded the title compound as a white solid (727 mg, 75%). **^{1}H NMR** (500 MHz, $CDCl_3$): δ 8.00 (d, J = 8.3 Hz, 2 H), 7.46 (d, J = 8.2 Hz, 2 H), 7.37 – 7.27 (m, 5 H), 5.87 (d, J = 3.1 Hz, 1 H), 3.89 (d, J = 0.5 Hz, 3 H), 2.42 (d, J = 3.3 Hz, 1 H); **^{13}C NMR** (126 MHz, $CDCl_3$): δ 167.0, 148.8, 143.4, 129.9, 129.4, 128.8, 128.1, 126.8, 126.5, 76.1, 52.2. Spectral data were in agreement with the literature.[15]

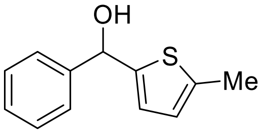

(5-Methylthiophen-2-yl)(phenyl)methanol (6o): Prepared according to **GP5** from 5-methyl-2-thiophenecarboxaldehyde (430 μL, 4.00 mmol). Purification by column chromatography (9:1 hexane/EtOAc) afforded the title compound as a white solid (693 mg, 85%). **^{1}H NMR** (500 MHz, $CDCl_3$): δ 7.46 – 7.44 (m, 2 H), 7.39 – 7.35 (m, 2 H), 7.30 (tt, J = 7.3, 1.3 Hz, 1 H), 6.68 (dd, J = 3.5, 0.9 Hz, 1 H), 6.58 (dq, J = 3.4, 1.1 Hz, 1 H), 5.97 (d, J = 3.7 Hz, 1 H), 2.44 (d, J = 1.0 Hz, 3 H), 2.32 (d, J = 3.9 Hz, 1 H); **^{13}C NMR** (126 MHz, $CDCl_3$): δ 145.8, 143.3, 140.4, 128.6, 128.0, 126.3, 125.1, 124.8, 72.6, 15.5. Spectral data were in agreement with the literature.[16]

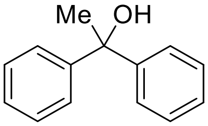

1,1-Diphenylethanol (6q): Under nitrogen, a flame-dried 50 mL round bottom flask equipped with stir bar was charged with benzophenone (728 mg, 4.00 mmol) and THF (12 mL). The solution was cooled in an ice bath to 0 °C, after which MeLi (5.0 mL, 1.6 M solution in Et_2O, 8.0 mmol, 2.0 equiv) was added dropwise. The resulting orange solution was stirred at 0 °C for 2 hours before removing the ice bath and stirring for an additional 18 hours. The reaction was cooled back to 0 °C and quenched by slow addition of saturated $NH_4Cl_{(aq)}$ (30 mL). The mixture was extracted with EtOAc (3 × 25 mL). The combined organic phases were washed with H_2O (35 mL) and brine (35 mL), dried over Na_2SO_4, filtered, and concentrated by rotary evaporation. Purification by column chromatography (7:1 hexane/EtOAc) afforded the title compound as a white solid (620 mg, 78%). **^{1}H NMR** (500 MHz, $CDCl_3$): δ 7.45 – 7.43 (m, 4 H), 7.34 (t, *J* = 7.6 Hz, 4 H), 7.26 (tt, *J* = 6.7, 1.3 Hz, 2 H), 2.19 (s, 1 H), 1.98 (s, 3 H); **^{13}C NMR** (126 MHz, $CDCl_3$): δ 148.1, 128.3, 127.1, 126.0, 76.4, 31.0. Spectral data were in agreement with the literature.[17]

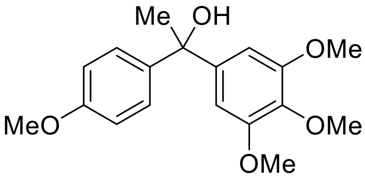

1-(4-Methoxyphenyl)-1-(3,4,5-trimethoxyphenyl)ethanol (6s): Under nitrogen, a flame-dried 50 mL round bottom flask equipped with stir bar was charged with 4-iodoanisole (1.12 g, 4.80 mmol, 1.20 equiv) and THF (8 mL). To this solution was added *i*-PrMgCl (2.4 mL, 2.0 M solution in THF, 4.8 mmol, 1.2 equiv) dropwise, which caused a color change from purple to yellow. The mixture was stirred at room temperature for 1 hour, at which point a solution of 3,4,5-trimethoxyacetophenone (841 mg, 4.00 mmol, 1.00 equiv) in THF (5 mL) was added dropwise. The resulting solution was stirred at room temperature for 14 hours, during which time it turned orange in color. The reaction was then cooled to 0 °C and quenched by slow addition of saturated $NH_4Cl_{(aq)}$ (30 mL). The mixture was extracted with EtOAc (3 × 25 mL). The combined organic phases were washed with H_2O (35 mL) and brine (35 mL), dried over Na_2SO_4, filtered, and concentrated by rotary evaporation. Purification by column chromatography (2:1 hexane/EtOAc) afforded the title compound as a clear oil (337 mg, 27%); **^{1}H NMR** (600 MHz, $CDCl_3$): δ 7.33 (d, *J* = 8.9 Hz, 2 H), 6.85 (d, *J* = 8.9 Hz, 2 H), 6.62 (s, 2 H), 3.83 (s, 3 H), 3.80 (s, 6 H), 3.80 (s, 3 H), 2.18 (s, 1 H), 1.91 (s, 3 H); **^{13}C NMR** (151 MHz, $CDCl_3$): δ 158.7, 152.9, 144.2, 140.2, 137.0, 127.2, 113.6, 103.4, 76.2, 60.9, 56.3, 55.4, 31.3; **FTIR** (cast film, cm^{-1}): 3474 (br, m), 2936 (m), 1590 (m), 1510 (s), 1413 (m), 1247 (m), 1125 (s), 1031 (w), 835 (w); **HRMS** (ESI) for $C_{18}H_{22}O_5Na$ $[M+Na]^+$: Calculated 341.1365; Found: 341.1363.

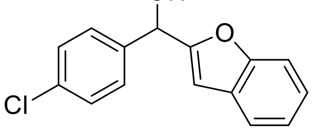

Benzofuran-2-yl(4-chlorophenyl)methanol (cloridarol) (6t): Under nitrogen, a flame-dried 50 mL round bottom flask equipped with stir bar was charged with 1-chloro-4-iodobenzene (1.33 g, 5.60 mmol, 1.40 equiv) and THF (20 mL). The solution was cooled in a dry ice/acetone bath to –

78 °C, after which *n*-BuLi (2.1 mL, 1.3 equiv, 2.5 M solution in hexanes) was added dropwise. The reaction was stirred at –78 °C for 1 hour, at which point a solution of 2-benzofurancarboxaldehyde (585 mg, 4.00 mmol, 1.00 equiv) in THF (6.6 mL) was added. The reaction was stirred at –78 °C for 15 minutes, at which point the cooling bath was removed and stirring continued for an additional 3 hours. The reaction was then cooled to 0 °C and quenched by slow addition of saturated $NH_4Cl_{(aq)}$ (30 mL). The mixture was extracted with EtOAc (3 × 25 mL). The combined organic phases were washed with H_2O (35 mL) and brine (35 mL), dried over Na_2SO_4, filtered, and concentrated by rotary evaporation. Purification by column chromatography (5:1 hexane/EtOAc) afforded the title compound as a thick yellow oil (696 mg, 67%). **^{1}H NMR** (700 MHz, $CDCl_3$): δ 7.52 (app d, *J* = 8.5 Hz, 3 H), 7.44 (d, *J* = 8.5 Hz, 2 H), 7.27 (td, *J* = 7.2, 1.3 Hz, 1 H), 7.21 (td, *J* = 7.5, 1.1 Hz, 1 H), 6.53 (s, 1 H), 5.94 (d, *J* = 4.5 Hz, 1 H), 2.50 (d, *J* = 4.5 Jz, 1 H); **^{13}C NMR** (151 MHz, $CDCl_3$): δ 158.1, 155.2, 138.8, 134.3, 128.9, 128.3, 128.0, 124.7, 123.1, 121.4, 111.5, 104.4, 70.1. Spectral data were in agreement with the literature.[18]

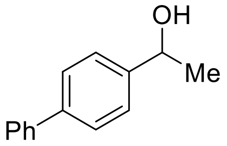

1-([1,1'-Biphenyl]-4-yl)ethanol (6u): Prepared according to **GP4** from 4-acetylbiphenyl (1.96 g, 10.0 mmol). Purification by column chromatography (6:1 hexane/EtOAc) afforded the title compound as a white solid (1.61 g, 81%). **^{1}H NMR** (500 MHz, $CDCl_3$): δ 7.60 – 7.57 (m, 4 H), 7.47 – 7.43 (m, 4 H), 7.35 (tt, *J* = 7.4, 1.2 Hz, 1 H), 4.96 (q, *J* = 6.5 Hz, 1 H), 1.80 (br s, 1 H), 1.55 (d, *J* = 6.4 Hz, 3 H); ^{13}C NMR (126 MHz, $CDCl_3$): δ 145.0, 141.0, 140.6, 128.9, 127.4, 127.2, 126.0, 70.3, 25.3. Spectral data were in agreement with the literature, which also observed only seven aromatic ^{13}C resonances due to overlap.[19]

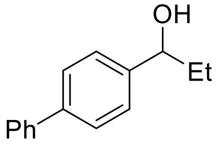

1-([1,1'-Biphenyl]-4-yl)propan-1-ol (6v): Under nitrogen, a flame-dried 50 mL round bottom flask equipped with stir bar was charged with 4-biphenylcarboxaldehyde (728 mg, 4.00 mmol) and THF (8 mL). The solution was cooled in an ice bath to 0 °C, after which EtMgBr (2.0 mL, 1.5 equiv, 3.0 M solution in Et_2O) was added dropwise. The reaction was stirred at 0 °C for 30 minutes, at which point the cooling bath was removed and stirring continued for an additional 3 hours. The reaction was quenched by addition of saturated $NH_4Cl_{(aq)}$. The mixture was extracted with EtOAc (3 × 25 mL). The combined organic phases were washed with H_2O (35 mL) and brine (35 mL), dried over Na_2SO_4, filtered, and concentrated by rotary evaporation. Purification by column chromatography (9:1 hexane/EtOAc) afforded the title compound as a white solid (619 mg, 73%). **^{1}H NMR** (500 MHz, $CDCl_3$): δ 7.61 – 7.58 (m, 4 H), 7.46 – 7.41 (m, 4 H), 7.35 (tt, *J* = 7.4, 1.3 Hz, 1 H), 4.66 (td, *J* = 6.6, 3.3 Hz, 1 H), 1.92 – 1.76 (m, 3 H), 0.97 (t, 7.4 Hz, 3 H); **^{13}C NMR** (126 MHz, $CDCl_3$): δ 143.8, 141.0, 140.6, 128.9, 127.4, 127.3, 127.2, 126.6, 75.9, 32.0, 10.3. Spectral data were in agreement with the literature.[20]

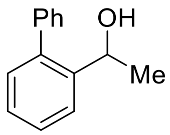

1-([1,1'-Biphenyl]-2-yl)ethanol (6w): Under nitrogen, a flame-dried 50 mL round bottom flask equipped with stir bar was charged with 2-biphenylcarboxaldehyde (645 µL, 4.00 mmol) and THF (8 mL). The solution was cooled in an ice bath to 0 °C, after which MeMgCl (2.0 mL, 1.5 equiv, 3.0 M solution in THF) was added dropwise. The reaction was stirred at 0 °C for 30 minutes, at which point the cooling bath was removed and stirring continued for an additional 3 hours. The reaction was quenched by addition of saturated $NH_4Cl_{(aq)}$. The mixture was extracted with EtOAc (3 × 25 mL). The combined organic phases were washed with H_2O (35 mL) and brine (35 mL), dried over Na_2SO_4, filtered, and concentrated by rotary evaporation. Purification by column chromatography (10:1 hexane/EtOAc) afforded the title compound as am off-white solid (571 mg, 72%). **^{1}H NMR** (500 MHz, $CDCl_3$): δ 7.68 (dd, *J* = 7.9, 1.4 Hz, 1 H), 7.44 – 7.41 (m, 3 H), 7.39 – 7.35 (m, 1 H),7.34 – 7.30 (m, 3 H), 7.22 (dd, *J* = 7.6, 1.4 Hz, 1 H), 4.99 (q, *J* = 6.4 Hz, 1 H), 1.70 (s, 1 H), 1.42 (d, *J* = 6.4 Hz, 3 H); **^{13}C NMR** (126 MHz, $CDCl_3$): δ 143.2, 141.1, 140.5, 130.1, 129.4, 128.3, 127.3, 127.2, 125.5, 66.6, 25.0. Spectral data were in agreement with the literature.[21]

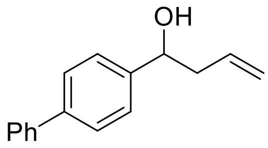

1-([1,1'-Biphenyl]-4-yl)but-3-en-1-ol (6x): Under nitrogen, a flame-dried 50 mL round bottom flask equipped with stir bar was charged with 4-biphenylcarboxaldehyde (728 mg, 4.00 mmol) and THF (12 mL). The solution was cooled in an ice bath to 0 °C, after which allylmagnesium chloride (3.0 mL, 1.5 equiv, 2.0 M solution in THF) was added dropwise. The reaction was stirred at 0 °C for 30 minutes, at which point the cooling bath was removed and stirring continued for an additional 3 hours. The reaction was quenched by addition of saturated $NH_4Cl_{(aq)}$. The mixture was extracted with EtOAc (3 × 25 mL). The combined organic phases were washed with H_2O (35 mL) and brine (35 mL), dried over Na_2SO_4, filtered, and concentrated by rotary evaporation. Purification by column chromatography (9:1 hexane/EtOAc) afforded the title compound as a white solid (592 mg, 66%). **^{1}H NMR** (500 MHz, $CDCl_3$): δ 7.61 – 7.58 (m, 4 H), 7.46 – 7.43 (m, 4 H), 7.35 (tt, *J* = 6.8, 1.2 Hz, 1 H), 5.86 (dddd, *J* = 16.9, 10.2, 7.6, 6.5 Hz, 1 H), 5.23 – 5.16 (m, 2 H), 4.80 (ddd, *J* = 8.1, 5.0, 3.2 Hz, 1 H), 2.62 – 2.51 (m, 2 H), 2.07 (d, *J* = 3.2 Hz, 1 H); **^{13}C NMR** (126 MHz, $CDCl_3$): δ 143.0, 141.0, 140.6, 134.6, 128.9, 127.4, 127.3, 127.2, 126.4, 118.7, 73.2, 44.0. Spectral data were in agreement with the literature.[22]

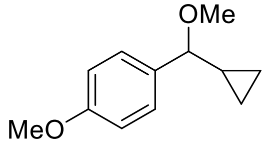

1-(Cyclopropyl(methoxy)methyl)-4-methoxybenzene (6y): Prepared according to **GP4** from Cyclopropyl 4-methoxyphenyl ketone (705 mg, 4.0 mmol), where 1M $HCl_{(aq)}$ was used to quench

the reaction rather than $NH_4Cl_{(aq)}$. Purification by column chromatography (7:1 hexane/EtOAc) afforded the title compound as a clear oil (613 mg, 80%). **^{1}H NMR** (500 MHz, $CDCl_3$): δ 7.24 (d, *J* = 8.4 Hz, 2 H), 6.89 (d, *J* = 8.7 Hz, 2 H), 3.81 (s, 3 H), 3.50 (d, 7.9 Hz, 1 H), 3.23 (s, 3 H), 1.19 – 1.12 (m, 1 H), 0.66 – 0.61 (m, 1 H), 0.46 – 0.40 (m, 2 H), 0.25 – 0.19 (m, 1 H); **^{13}C NMR** (126 MHz, $CDCl_3$): δ 159.2, 134.0, 128.1, 113.8, 87.4, 56.0, 55.4, 17.7, 4.4, 1.9. Spectral data were in agreement with the literature.[23]

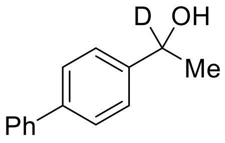

(92% D incorporation)

1-([1,1'-Biphenyl]-4-yl)-1-deuteroethanol (6z): Prepared according to **GP4** from 4-acetylbiphenyl (785 mg, 4.00 mmol) and $NaBD_4$ (252 mg, 6.00 mmol, 1.50 equiv). Purification by column chromatography (3:1 hexane/EtOAc) afforded the title compound as a white solid (669 mg, 84%). **^{1}H NMR** (500 MHz, $CDCl_3$): δ 7.61 – 7.57 (m, 4 H), 7.47 – 7.43 (m, 4 H), 7.35 (tt, *J* = 6.8, 1.2 Hz, 1 H), 1.80 (s, 1 H), 1.54 (s, 3 H); **^{13}C NMR** (126 MHz, $CDCl_3$): δ 144.9, 141.0, 140.6, 128.9, 127.4, 127.3, 127.2, 126.0, 69.9 (1:1:1 t, *J* = 21.9 Hz), 25.2. Additional resonances are observed from the 8% of non-deuterium labelled analog **6u** in ^{1}H NMR (4.96 ppm, q, *J* = 6.5 Hz, 0.09 H corresponding to the benzylic proton) and ^{13}C NMR (25.3 ppm and 70.3 ppm, corresponding to the methyl and benzylic carbons respectively) and are marked with an * in the spectra found in Section 12. Spectral data were in agreement with the literature.[24]

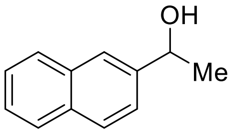

1-(Naphthalen-2-yl)ethanol (6aa): Prepared according to **GP4** from 2-acetylnaphthalene (851 mg, 5.00 mmol). Purification by column chromatography (5:1 hexane/EtOAc) afforded the title compound as a white solid (759 mg, 88%). **^{1}H NMR** (500 MHz, $CDCl_3$): δ 7.85 – 7.81 (m, 4 H), 7.51 (dd, *J* = 8.7, 1.8 Hz, 1 H), 7.49 – 7.45 (m, 2 H), 5.07 (qd, *J* = 6.5, 2.3 Hz, 1 H), 1.94 (d, *J* = 2.4 Hz, 1 H), 1.59 (d, *J* = 6.5 Hz, 3 H); **^{13}C NMR** (126 MHz, $CDCl_3$): δ 143.3, 133.5, 133.1, 128.5, 128.1, 127.8, 126.3, 125.9, 123.96, 123.95, 70.7, 25.3. Spectral data were in agreement with the literature.[14]

6.2 Synthesis and Characterization of Benzylic Ketones

Ketones **8a, 8b, 8e, 8u, 8aa, 8ae,8af, 8ag, 8ah, 8ai** (fenofibrate) and **8aj** (ketoprofen) were purchased from commercial suppliers and used as received.

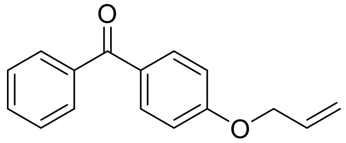

(4-(Allyloxy)phenyl)(phenyl)methanone (8ab): Under nitrogen, a flame-dried 50 mL round bottom flask equipped with stir bar was charged with 4-hydroxybenzophenone (991 mg, 5.00 mmol), K_2CO_3 (1.38 g, 10.0 mmol, 2.00 equiv), KI (41 mg, 0.25 mmol, 5 mol%) and acetone (20 mL). The resulting suspension was stirred at room temperature for 5 minutes, at which point allyl bromide (866 µL, 10.0 mmol, 2.00 equiv) was added via syringe. The mixture was heated to reflux (66 °C) for 4 hours. The reaction was then cooled to room temperature and concentrated by rotary evaporation. The mixture was taken up in 30 mL CH_2Cl_2 and washed with H_2O (15 mL), 1 M $NaOH_{(aq)}$ (3 × 10 mL) and brine (20 mL). The organic phase was dried over Na_2SO_4, filtered, and concentrated by rotary evaporation. Purification by column chromatography (gradient 1:3 to 1:10 hexane/CH_2Cl_2) afforded the title compound as a white solid (862 mg, 72%). **^{1}H NMR** (500 MHz, $CDCl_3$): δ 7.82 (d, *J* = 9.0 Hz, 2 H), 7.77 – 7.75 (m, 2 H), 7.56 (tt, *J* = 6.7, 1.3 Hz, 1H), 7.49 – 7.46 (m, 2 H), 6.98 (d, *J* = 8.9 Hz, 2 H), 6.07 (ddt, *J* = 17.2, 10.5, 5.3 Hz, 1 H), 5.44 (dq, *J* = 17.3, 1.6 Hz, 1 H), 5.33 (dq, *J* = 10.5, 1.4 Hz, 1 H), 4.62 (dt, *J* = 5.3, 1.5 Hz, 2 H); **^{13}C NMR** (126 MHz): δ 195.6, 162.4, 138.4, 132.68. 132.66, 132.0, 130.4, 129.9, 128.3, 118.3, 114.4, 69.1. Spectral data were in agreement with the literature.[25]

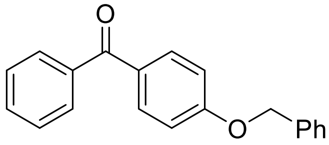

(4-(Benzyloxy)phenyl)(phenyl)methanone (8ac): Under nitrogen, a flame-dried 50 mL round bottom flask equipped with stir bar was charged with 4-hydroxybenzophenone (793 mg, 4.00 mmol), K_2CO_3 (1.11 g, 8.0 mmol, 2.00 equiv), KI (33 mg, 0.20 mmol, 5 mol%) and acetone (16 mL). The resulting suspension was stirred at room temperature for 5 minutes, at which point benzyl bromide (950 µL, 8.0 mmol, 2.00 equiv) was added via syringe. The mixture was heated to reflux (66 °C) for 4 hours. The reaction was then cooled to room temperature and concentrated by rotary evaporation. The mixture was taken up in 30 mL CH_2Cl_2 and washed with H_2O (15 mL), 1 M $NaOH_{(aq)}$ (3 × 10 mL) and brine (20 mL). The organic phase was dried over Na_2SO_4, filtered, and concentrated by rotary evaporation. Purification by column chromatography (gradient 1:3 to 1:10 hexane/CH_2Cl_2) afforded the title compound as a white solid (667 mg, 58%). **^{1}H NMR** (500 MHz, $CDCl_3$): δ 7.83 (d, *J* = 9.0 Hz, 2 H), 7.77 – 7.75 (m, 2 H), 7.57 (tt, *J* = 7.5, 1.3 Hz, 1 H), 7.49 – 7.39 (m, 6 H), 7.35 (tt, *J* = 7.2, 1.6 Hz, 1 H), 7.04 (d, *J* = 8.9 Hz, 2 H), 5.16 (s, 2 H); **^{13}C NMR** (126 MHz, $CDCl_3$) δ 195.7, 162.5, 138.4, 136.4, 132.7, 132.0, 130.5, 129.9, 128.9, 128.4, 128.3, 127.6, 114.6, 70.3. Spectral data were in agreement with the literature.[26]

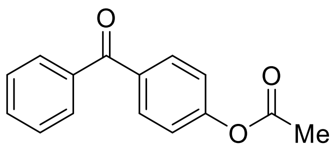

4-Benzoylphenyl acetate (8ad): Under nitrogen, a flame-dried 100 mL round bottom flask equipped with stir bar was charged with 4-hydroxyphenol (968 mg, 4.80 mmol), CH_2Cl_2 (25 mL), triethylamine (1.4 mL, 9.6 mmol, 2.0 equiv) and acetyl chloride (512 µL, 7.2 mmol, 1.5 equiv). The mixture was heated to reflux (39 °C) for 12 hours. The reaction was then cooled to room temperature and diluted with CH_2Cl_2 (20 mL). The reaction was washed with H_2O (2 × 20 mL), saturated $NaHCO_{3(aq)}$ (2 × 25 mL) and brine (25 mL). The organic phase was dried over Na_2SO_4, filtered, and concentrated by rotary evaporation. Purification by column chromatography (5:1 hexane/EtOAc) afforded the title compound as a white solid (976 mg, 85%). **^{1}H NMR** (500 MHz, $CDCl_3$): δ 7.86 (d, *J* = 8.9 Hz, 2 H), 7.81 – 7.79 (dd, *J* = 8.3, 1.4 Hz, 2 H), 7.59 (tt, *J* = 7.5, 1.84 Hz, 1 H), 7.49 (t, *J* = 7.7 Hz, 2 H), 7.22 (d, *J* = 8.7 Hz, 2 H), 2.34 (s, 3 H); **^{13}C NMR** (126 MHz, $CDCl_3$): δ 195.7, 169.1, 154.0, 137.7, 135.2, 132.6, 131.8, 130.1, 128.5, 121.7, 21.3. Spectral data were in agreement with the literature.[27]

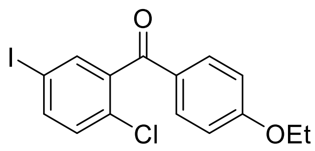

(2-Chloro-5-iodophenyl)(4-ethoxyphenyl)methanone (8ai): Under nitrogen, a flame-dried 25 mL round bottom flask equipped with a stir bar was charged with 2-chloro-5-iodobenzoic acid (1.13 g, 4.00 mmol), CH_2Cl_2 (8.0 mL) and DMF (one drop). The suspension was stirred at room temperature, during which time oxalyl chloride (412 µL, 4.80 mmol, 1.20 equiv) was added dropwise via syringe. The reaction was stirred at room temperature for an additional two hours, during which time a homogeneous solution was formed. This solution was subsequently transferred dropwise via syringe to a solution of ethoxybenzene (581 µL, 4.60 mmol, 1.15 equiv) and anhydrous $AlCl_3$ (667 mg, 5.00 mmol, 1.25 equiv) in CH_2Cl_2 (8.0 mL) under nitrogen at 0 °C. The resulting dark red solution was stirred at 0 °C for an additional two hours, after which time the ice bath was removed and stirring continued for an additional two hours at room temperature. The mixture was diluted with additional CH_2Cl_2 (25 mL), and the organic layer washed with 1 M $HCl_{(aq)}$ (15 mL), saturated $NaHCO_{3(aq)}$ (2 × 15 mL), and brine (20 mL). The organic phase was dried over Na_2SO_4, filtered, and concentrated by rotary evaporation. Crude NMR analysis revealed a mixture of *para*- and *ortho*- isomers in a 6.3 : 1.0 ratio. Purification by column chromatography (25:1 hexane/ethyl acetate) afforded the title compound as a white solid (412 mg, 27%). The *para*- to *ortho*- ratio of purified material was >40:1, and characterization data is given for the major product only. **^{1}H NMR** (500 MHz, $CDCl_3$): δ 7.76 (d, *J* = 8.9 Hz, 2 H), 7.72 (dd, *J* = 8.4, 2.1 Hz, 1 H), 7.65 (d, *J* = 2.1 Hz, 1 H), 7.18 (d, *J* = 8.4 Hz, 1 H), 6.93 (d, *J* = 8.9 Hz, 2 H), 4.12 (q, *J* = 7.0 Hz, 2 H), 1.45 (t, *J* = 7.0 Hz, 3 H) **^{13}C NMR** (126 MHz, $CDCl_3$) δ 192.1, 164.0, 141.1, 139.8, 137.4, 132.7, 131.8, 131.2, 128.8, 114.6, 91.4, 64.1, 14.8. Spectral data were in agreement with the literature.[28]

7. Reductive Deoxygenation – Optimization

7.1 Optimization of Reductive Deoxygenation of Benzylic Alcohols (Table 1)

General Procedure for the reductive deoxygenation of alcohols using NMR yields (GP6)

A vial equipped with a stir bar was charged with alcohol **6a** (27.6 mg, 0.150 mmol), catalyst **3** (X mol%), triethylsilane (26 µL, 0.16 mmol, 1.1 equiv) and solvent. The vial was then capped and stirred at room temperature for the indicated time, after which it was diluted with $CHCl_3$ and filtered through a small pipette of silica (approximately 1 cm high) with $CHCl_3$ washings to remove insoluble components. The mixture was concentrated by rotary evaporation, and yields were obtained by ^{1}H NMR relative to 1,3,5-trimethoxybenzene as an internal standard.

Supplementary Table 2. Optimization of catalytic alcohol reductive deoxygenation.

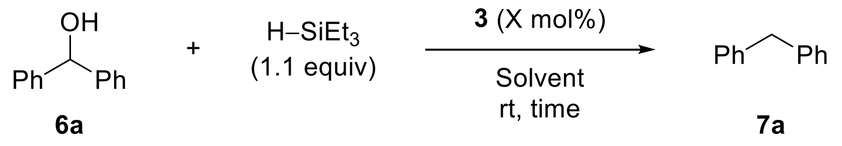

Entry	Catalyst (X mol%)	Solvent	Concentration	Time	Yield 7a
1	10 mol%	HFIP/$MeNO_2$ 4:1	0.5 M	18 h	96%
2	5 mol%	HFIP/$MeNO_2$ 4:1	0.5 M	18 h	98%
3	1 mol%	HFIP/$MeNO_2$ 4:1	0.5 M	18 h	97%
4	1 mol%	HFIP/$MeNO_2$ 4:1	0.5 M	1.5 h	98%
5	1 mol%	HFIP/$MeNO_2$ 1:4	0.5 M	1.5 h	68%
6	1 mol%	$MeNO_2$	0.5 M	1.5 h	0%
7	1 mol%	MeCN	0.5 M	1.5 h	0%
8	1 mol%	CH_2Cl_2	0.5 M	1.5 h	0%
9	1 mol%	PhMe	0.5 M	1.5 M	0%
10	1 mol%	TFE/$MeNO_2$ 4:1	0.5 M	1.5 h	38%
11	1 mol%	HFIP/$MeNO_2$ 4:1	1.0 M	1.5 h	95%
12	1 mol%	HFIP/$MeNO_2$ 4:1	1.5 M	1.5 h	96%
13	1 mol%	HFIP/$MeNO_2$ 4:1	2.0 M	1.5 h	98%
14	1 mol%	HFIP	2.0 M	1.5 h	90%
15	-	HFIP/$MeNO_2$ 4:1	2.0 M	1.5 h	0%

7.2 Optimization of Reductive Deoxygenation of Benzylic Ketones

General Procedure for the reductive deoxygenation of ketones using NMR yields (GP7)

A vial equipped with a stir bar was charged with ketone **8a** (27.6 mg, 0.150 mmol), catalyst **3** (X mol%), silane (Y equiv) and solvent. The vial was then capped and stirred at room temperature for the indicated time, after which it was diluted with $CHCl_3$ and filtered through a small pipette of silica (approximately 1 cm high) with $CHCl_3$ washings to remove insoluble components. The mixture was concentrated by rotary evaporation, and yields were obtained by ^{1}H NMR relative to 1,3,5-trimethoxybenzene as an internal standard.

Supplementary Table 3. Optimization of catalytic ketone reductive deoxygenation.

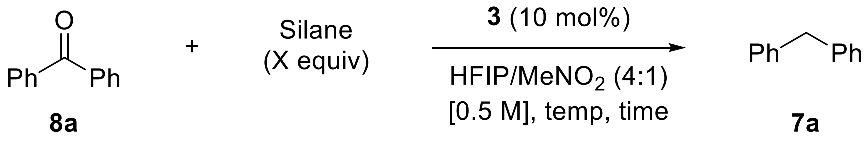

Entry	Silane (equiv)	Time	Temp	Yield 7a
1	$HSiEt_3$ (3.0 equiv)	18 h	rt	54%
2	$HSiEt_3$ (5.0 equiv)	18 h	80 °C	84%
3	$HSiEt_3$ (5.0 equiv)	18 h	rt	43%
4	$HSiEt_3$ (5.0 equiv)	6 h	rt	20%
5	PMHS (5.0 equiv)	6 h	rt	3%
6	$HSi(OEt)_3$ (5.0 equiv)	6 h	rt	11%
7	$PhSiH_3$ (5.0 equiv)	6 h	rt	7%
8	$HSiMe_2Ph$ (3.0 equiv)	6 h	rt	88%
9	$HSiMe_2Ph$ (5.0 equiv)	18 h	rt	94%
10	$HSiMe_2Ph$ (3.0 equiv)	18 h	rt	90%
11	$HSiMe_2Ph$ (2.0 equiv)	18 h	rt	70%
12	$HSiMe_2Ph$ (2.0 equiv)	48 h	rt	83%
13	$(HSiMe_2)_2O$ (2.2 equiv)	24 h	rt	95%[a]
14	$(HSiMe_2)_2O$ (2.2 equiv)	24 h	rt	97%[a,b]
15	$(HSiMe_2)_2O$ (1.1 equiv)	24 h	rt	56%[a,b]
16	$(HSiMe_2)_2O$ (2.0 equiv)	24 h	rt	84%[a,b]

[a]5 mol% catalyst. [b]HFIP used as solvent without $MeNO_2$

8. Reductive Deoxygenation – Substrate Scope

8.1 Reductive Deoxygenation of Benzylic Alcohols (Figure 5)

General Procedure for the reductive deoxygenation of benzylic alcohols (GP8)

A vial equipped with a stir bar was charged with alcohol **6**, catalyst **3** (0.1 – 5 mol%), triethylsilane (1.1 equiv), HFIP and $MeNO_2$ (4:1 ratio, 2.0 M in alcohol **6**). The reaction was stirred at room temperature for the indicated reaction time, after which it was concentrated by rotary evaporation. Purification by column chromatography afforded the reduction product **7**.

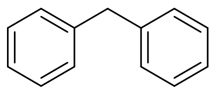

Diphenylmethane (7a): Prepared according to **GP8** from alcohol **6a** (2.02 g, 11.0 mmol), catalyst **3** (22.0 mg, 0.110 mmol, 1 mol%) and triethylsilane (1.94 mL, 12.1 mmol, 1.10 equiv) for 90 minutes. Purification by column chromatography (hexane) afforded the title compound as a clear oil (1.73 g, 94%). **^{1}H NMR** (500 MHz, $CDCl_3$): δ 7.32 – 7.28 (m, 4 H), 7.23 – 7.20 (m, 6 H), 4.00 (s, 2 H); **^{13}C NMR** (126 MHz, $CDCl_3$): δ 141.3, 129.1, 128.6, 126.2, 42.1. Spectral data were in agreement with the literature.[29]

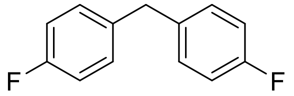

Bis(4-fluorophenyl)methane (7b): Prepared according to **GP8** from alcohol **6b** (220 mg, 1.00 mmol), catalyst **3** (2.0 mg, 0.010 mmol, 1 mol%) and triethylsilane (176 µL, 1.10 mmol, 1.10 equiv) for 90 minutes. Purification by column chromatography (hexane) afforded the title compound as a clear oil (138 mg, 68%). **^{1}H NMR** (500 MHz, $CDCl_3$): δ 7.12 (dd, *J* = 8.8, 5.4 Hz, 4 H), 6.98 (app t, *J* = 8.7 Hz, 4 H), 3.93 (s, 2 H); **^{13}C NMR** (126 MHz, $CDCl_3$): δ 161.6 (d, *J*= 244.2 Hz), 136.7 (d, *J* = 3.5 Hz), 130.3 (d, *J* = 8.1 Hz), 115.4 (d, *J* = 21.0 Hz), 40.4; **^{19}F NMR** (376 MHz, $CDCl_3$): δ – 117.1 (tt, *J* = 9.1, 5.3 Hz). Spectral data were in agreement with the literature.[30]

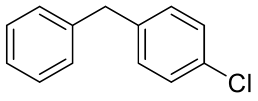

1-Benzyl-4-chlorobenzene (7c): Prepared according to **GP8** from alcohol **6c** (218 mg, 1.00 mmol), catalyst **3** (2.0 mg, 0.010 mmol, 1 mol%) and triethylsilane (176 µL, 1.10 mmol, 1.10 equiv) for 90 minutes. Purification by column chromatography (hexane) afforded the title compound as a clear oil (164 mg, 81%). **^{1}H NMR** (500 MHz, $CDCl_3$): δ 7.33 (t, *J* = 7.2 Hz, 2 H), 7.29 (d, *J* = 8.6 Hz, 2 H), 7.25 (tt, *J* = 7.5, 1.4 Hz, 1 H), 7.20 (dd, *J* = 7.6, 0.7 Hz, 2 H), 7.16 (d, *J* = 8.4 Hz, 2 H), 3.99 (s, 2 H); **^{13}C NMR** (126 MHz, $CDCl_3$): δ 140.7, 139.7, 132.0, 130.4, 129.0, 128.7, 126.4, 41.4. Spectral data were in agreement with the literature.[29]

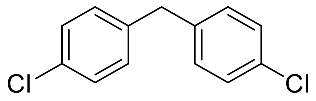

Bis(4-chlorophenyl)methane (7d): Prepared according to **GP8** from alcohol **6d** (253 mg, 1.00 mmol), catalyst **3** (2.0 mg, 0.010 mmol, 1 mol%) and triethylsilane (176 µL, 1.10 mmol, 1.10 equiv) for 90 minutes. Purification by column chromatography (hexane) afforded the title compound as a white solid (206 mg, 87%). **^{1}H NMR** (500 MHz, $CDCl_3$): δ 7.29 (d, *J* = 8.5 Hz, 4

H), 7.12 (d, J = 8.6 Hz, 4 H), 3.94 (s, 2 H); **^{13}C NMR** (126 MHz, $CDCl_3$): δ 139.2, 132.3, 130.3, 128.8. Spectral data were in agreement with the literature.[30]

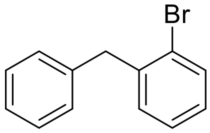

1-Benzyl-2-bromobenzene (7e): Prepared according to **GP8** from alcohol **6e** (263 mg, 1.00 mmol), catalyst **3** (2.0 mg, 0.010 mmol, 1 mol%) and triethylsilane (176 µL, 1.10 mmol, 1.10 equiv) for 90 minutes. Purification by column chromatography (hexane) afforded the title compound as a clear oil (174 mg, 71%). **^{1}H NMR** (500 MHz, $CDCl_3$): δ 7.58 (dd, J = 8.0, 1.4 Hz, 1 H), 7.30 (t, J = 7.3 Hz, 2 H), 7.25 – 7.19 (m, 4 H), 7.14 (dd, J = 7.8, 1.8 Hz, 1 H), 7.09 (td, J = 7.8, 1.8 Hz, 1 H), 4.13 (s, 2 H); **^{13}C NMR** (126 MHz, $CDCl_3$): δ 140.5, 139.6, 133.0, 131.2, 129.2, 128.6, 128.0, 127.6, 126.4, 125.0, 41.9. Spectral data were in agreement with the literature.[31]

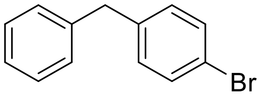

1-Benzyl-4-bromobenzene (7f): Prepared according to **GP8** from alcohol **6f** (263 mg, 1.00 mmol), catalyst **3** (2.0 mg, 0.010 mmol, 1 mol%) and triethylsilane (176 µL, 1.10 mmol, 1.10 equiv) for 90 minutes. Purification by column chromatography (hexane) afforded the title compound as a clear oil (180 mg, 73%). **^{1}H NMR** (500 MHz, $CDCl_3$): δ 7.41 (d, J = 8.4 Hz, 2 H), 7.30 (t, J = 7.2 Hz, 2 H), 7.22 (tt, J = 7.4, 2.2 Hz, 1 H), 7.17 (d, J = 7.5 Hz, 2 H), 7.07 (d, J = 8.7 Hz, 2 H), 3.94 (s, 2 H); **^{13}C NMR** (126 MHz, $CDCl_3$): δ 140.6, 140.2, 131.7, 130.8, 129.0, 128.7, 126.4, 120.1, 41.5. Spectral data were in agreement with the literature.[29]

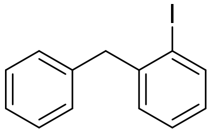

1-Benzyl-2-iodobenzene (7g): Prepared according to **GP8** from alcohol **6g** (310 mg, 1.00 mmol), catalyst **3** (2.0 mg, 0.010 mmol, 1 mol%) and triethylsilane (176 µL, 1.10 mmol, 1.10 equiv) for 90 minutes. Purification by column chromatography (hexane) afforded the title compound as a clear oil (244 mg, 83%). **^{1}H NMR** (500 MHz, $CDCl_3$): δ 7.87 (dd, J = 7.9, 1.3 Hz, 1 H), 7.32 – 7.28 (m, 2 H), 7.27 – 7.21 (m, 2 H), 7.20 – 7.18 (m, 2 H), 7.12 (d, J = 7.7, 1.7 Hz, 1 H), 6.92 (td, J = 7.6, 1.7 Hz, 1 H), 4.12 (s, 2 H); **^{13}C NMR** (126 MHz, $CDCl_3$): δ 143.8, 139.7, 130.5, 129.2, 128.6, 128.5, 128.2, 126.4, 101.4, 46.6. Spectral data were in agreement with the literature.[32]

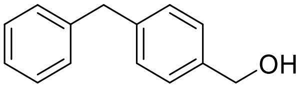

(4-Benzylphenyl)methanol (7h): Prepared according to **GP8** from alcohol **6h** (193 mg, 0.900 mmol), catalyst **3** (1.8 mg, 0.0090 mmol, 1 mol%) and triethylsilane (159 µL, 0.990 mmol, 1.10 equiv) for 90 minutes. Purification by column chromatography (7:1 hexane/EtOAc) afforded the title compound as a white solid (138 mg, 78%). **^{1}H NMR** (500 MHz, $CDCl_3$): δ 7.30 – 7.28 (m, 4

H), 7.22 – 7.18 (m 5 H), 4.66 (s, 2 H), 3.99 (s, 2 H), 1.61 (br s, 1 H); **^{13}C NMR** (126 MHz, $CDCl_3$): δ 141.2, 140.8, 138.8, 129.3, 129.0, 128.6, 127.4, 126.2, 65.4, 41.8. Spectral data were in agreement with the literature.[33]

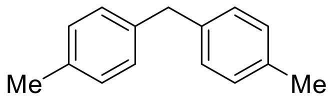

Di-p-tolylmethane (7i): Prepared according to **GP8** from alcohol **6i** (212 mg, 1.00 mmol), catalyst **3** (2.0 mg, 0.010 mmol, 1 mol%) and triethylsilane (176 µL, 1.10 mmol, 1.10 equiv) for 90 minutes. Purification by column chromatography (hexane) afforded the title compound as a clear oil (184 mg, 94%). **^{1}H NMR** (500 MHz, $CDCl_3$): δ 7.12 – 7.08 (m, 8 H), 3.93 (s, 2 H), 2.33 (s, 6 H); **^{13}C NMR** (126 MHz, $CDCl_3$): δ 138.5, 135.6, 129.3, 128.9, 41.2, 21.1. Spectral data were in agreement with the literature.[30]

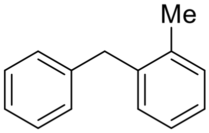

1-Benzyl-2-methylbenzene (7j): Prepared according to **GP8** from alcohol **6j** (198 mg, 1.00 mmol), catalyst **3** (2.0 mg, 0.010 mmol, 1 mol%) and triethylsilane (176 µL, 1.10 mmol, 1.10 equiv) for 90 minutes. Purification by column chromatography (hexane) afforded the title compound as a clear oil (154 mg, 84%). **^{1}H NMR** (500 MHz, $CDCl_3$): δ 7.33 – 7.30 (m, 2 H), 7.25 – 7.14 (m, 7 H), 4.04 (s, 2 H), 2.29 (s, 3 H); **^{13}C NMR** (126 MHz, $CDCl_3$): δ 140.5, 139.1, 136.8, 130.4, 130.1, 128.9, 128.5, 126.6, 126.13, 126.06, 39.6, 19.8. Spectral data were in agreement with the literature.[29]

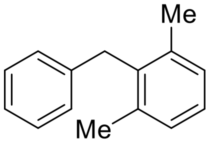

2-Benzyl-1,3-dimethylbenzene (7k): Prepared according to **GP8** from alcohol **6k** (159 mg, 0.750 mmol), catalyst **3** (7.5 mg, 0.0375 mmol, 5 mol%) and triethylsilane (135 µL, 0.825 mmol, 1.10 equiv) for 90 minutes. Purification by column chromatography (hexane) afforded the title compound as a clear oil (117 mg, 80%). **^{1}H NMR** (500 MHz, $CDCl_3$): δ 7.29 (t, J = 7.2 Hz, 2 H), 7.21 (t, J = 7.5 Hz, 1 H), 7.17 – 7.11 (m, 3 H), 7.07 (d, J = 7.8 Hz, 2 H), 4.12 (s, 2 H), 2.30 (s, 6 H); **^{13}C NMR** (126 MHz, $CDCl_3$): δ 139.9, 137.3, 137.0, 128.5, 128.3, 128.0, 126.5, 125.9, 35.2, 20.4. Spectral data were in agreement with the literature.[34]

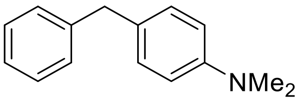

4-Benzyl-*N,N*-dimethylaniline (7l): Prepared according to **GP8** from alcohol **6l** (227 mg, 1.00 mmol), catalyst **3** (10.0 mg, 0.050 mmol, 5 mol%) and triethylsilane (176 µL, 1.10 mmol, 1.10 equiv) with a reaction time of 18 hours. Purification by column chromatography (20:1

hexane/EtOAc) afforded the title compound as a clear oil (158 mg, 75%). ^{1}**H NMR** (500 MHz, $CDCl_3$): δ 7.31 – 7.27 (m, 2 H), 7.22 – 7.18 (m, 3 H), 7.09 (d, *J* = 8.8 Hz, 2 H), 6.72 (d, *J* = 8.8 Hz, 2 H), 3.92 (s, 2 H), 2.94 (s, 6 H); ^{13}**C NMR** (126 MHz, $CDCl_3$): δ 149.3, 142.2, 129.7, 129.4, 129.0, 128.5, 125.9, 113.1, 41.1, 41.0. Spectral data were in agreement with the literature.[35]

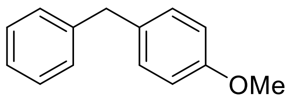

1-Benzyl-4-methoxybenzene (7m): Prepared according to **GP8** from alcohol **6m** (214 mg, 1.00 mmol), catalyst **3** (2.0 mg, 0.010 mmol, 1 mol%) and triethylsilane (176 μL, 1.10 mmol, 1.10 equiv) for 90 minutes. Purification by column chromatography (200:1 hexane/EtOAc) afforded the title compound as a clear oil (181 mg, 91%). ^{1}**H NMR** (600 MHz, $CDCl_3$): δ 7.29 (t, *J* = 7.1 Hz, 2 H), 7.21 – 7.18 (m, 3 H), 7.12 (d, *J* = 8.6 Hz, 2 H), 6.84 (d, *J* = 8.8 Hz, 2 H), 3.94 (s, 2 H), 3.79 (s, 3 H); ^{13}**C NMR** (151 MHz, $CDCl_3$): δ 158.1, 141.7, 133.4, 130.0, 128.9, 128.6, 126.1, 114.0, 55.4, 41.2. Spectral data were in agreement with the literature.[29]

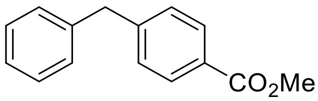

Methyl 4-benzylbenzoate (7n): Prepared according to **GP8** from alcohol **6n** (182 mg, 0.750 mmol), catalyst **3** (7.5 mg, 0.038 mmol, 5 mol%) and triethylsilane (134 μL, 0.825 mmol, 1.10 equiv) for 12 hours. Purification by column chromatography (gradient 19:1 to 9:1 hexane/EtOAc) afforded the title compound as a clear oil (144 mg, 85%). ^{1}**H NMR** (500 MHz, $CDCl_3$): δ 8.00 (dd, *J* = 8.4, 2.5 Hz, 2 H), 7.34 (td, *J* = 8.0, 1.6 Hz, 2 H), 7.31 – 7.24 (m, 3 H), 7.22 (d, *J* = 6.8 Hz, 2 H), 4.07 (s, 2 H), 3.94 (s, 3 H); ^{13}**C NMR** (126 MHz, $CDCl_3$): δ 167.2, 146.7, 140.3, 130.0, 129.1, 128.8, 128.3, 126.5, 52.1, 42.1. Spectral data were in agreement with the literature.[36]

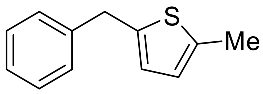

2-Benzyl-5-methylthiophene (7o): Prepared according to **GP8** from alcohol **6o** (204 mg, 1.00 mmol), catalyst **3** (2.0 mg, 0.010 mmol, 1 mol%) and triethylsilane (176 μL, 1.10 mmol, 1.10 equiv) for 90 minutes. Purification by column chromatography (hexane) afforded the title compound as a clear oil (142 mg, 75%). ^{1}**H NMR** (600 MHz, $CDCl_3$): δ 7.35 (tt, *J* = 6.1, 1.6 Hz, 2 H), 7.30 – 7.25 (m, 3 H), 6.63 – 6.62 (m, 1 H), 6.60 (dq, *J* = 3.4, 1.2 Hz, 1 H), 4.12 (s, 2 H), 2.46 (s, 3 H); ^{13}**C NMR** (176 MHz, $CDCl_3$): δ 141.9, 140.7, 138.5, 128.7, 126.5, 125.0, 124.9, 36.4, 15.4. Spectral data were in agreement with the literature.[37]

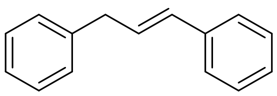

(E)-Prop-1-ene-1,3-diyldibenzene (7p): Prepared according to **GP8** from alcohol **6p** (210 mg, 1.00 mmol), catalyst **3** (10.0 mg, 0.050 mmol, 5 mol%) and triethylsilane (176 μL, 1.10 mmol, 1.10 equiv) for 90 minutes. Purification by column chromatography (hexane) afforded the title compound as a clear oil (158 mg, 82%). ^{1}**H NMR** (600 MHz, $CDCl_3$): δ 7.41 (d, *J* = 7.3 Hz, 2 H),

7.38 – 7.33 (m, 4 H), 7.31 – 7.24 (m, 4 H), 6.51 (dt, *J* = 15.8, 1.6 Hz, 1 H), 6.41 (dt, *J* = 15.8, 6.8 Hz, 1 H), 3.61 (d, 6.6 Hz, 2 H); **^{13}C NMR** (151 MHz, $CDCl_3$): δ 140.3, 137.6, 131.2, 129.4, 128.8, 128.64, 128.63, 127.2, 126.32, 126.27, 39.5. Spectral data were in agreement with the literature.[38]

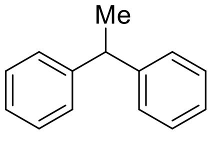

Ethane-1,1-diyldibenzene (7q): Prepared according to **GP8** from alcohol **6q** (198 mg, 1.00 mmol), catalyst **3** (2.0 mg, 0.010 mmol, 1 mol%) and triethylsilane (176 μL, 1.10 mmol, 1.10 equiv) for 90 minutes. Purification by column chromatography (hexane) afforded the title compound as a clear oil (154 mg, 85%). **^{1}H NMR** (500 MHz, $CDCl_3$): δ 7.30 (t, *J* = 7.7 Hz, 4 H), 7.25 – 7.23 (m, 4 H), 7.20 (tt, *J* = 7.2, 1.5 Hz, 2 H), 4.17 (q, *J* = 7.2 Hz, 1 H), 1.66 (d, *J* = 7.3 Hz, 3 H); **^{13}C NMR** (125 MHz, $CDCl_3$): δ 146.5, 128.5, 127.8, 126.2, 44.9, 22.0. Spectral data were in agreement with the literature.[29]

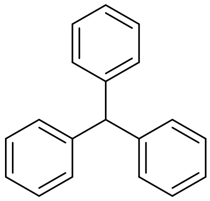

Triphenylmethane (7r): Prepared according to **GP8** from alcohol **6r** (5.21 g, 20.0 mmol), catalyst **3** (4.0 mg, 0.020 mmol, 0.1 mol%) and triethylsilane (3.50 mL, 22.0 mmol, 1.10 equiv) for 90 minutes. Purification by column chromatography (hexane) afforded the title compound as a white solid (4.44 g, 91%). **^{1}H NMR** (500 MHz, $CDCl_3$): δ 7.29 (t, *J* = 7.4 Hz, 6 H), 7.22 (tt, *J* = 7.5, 2.2 Hz, 3 H), 7.13 (d, *J* = 7.2 Hz, 6 H), 5.57 (s, 1 H); **^{13}C NMR** (125 MHz, $CDCl_3$): δ 144.1, 129.6, 128.4, 126.4, 57.0. Spectral data were in agreement with the literature.[39]

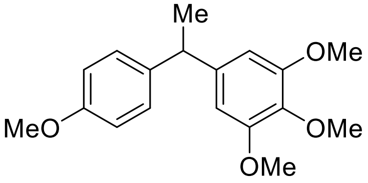

1,2,3-Trimethoxy-5-(1-(4-methoxyphenyl)ethyl)benzene (7s): Prepared according to **GP8** from alcohol **6s** (136 mg, 0.430 mmol), catalyst **3** (0.9 mg, 0.043 mmol, 1 mol%) and triethylsilane (84 μL, 0.47 mmol, 1.10 equiv) for 90 minutes. Purification by column chromatography (6:1 hexane/EtOAc) afforded the title compound as a clear oil (89 mg, 69%). **^{1}H NMR** (600 MHz, $CDCl_3$): δ 7.15 (d, *J* = 8.8 Hz, 2 H), 6.84 (d, *J* = 8.8 Hz, 2 H), 6.42 (s, 2 H), 4.04 (q, *J* = 7.2 Hz, 1 H), 3.82 (s, 3 H), 3.81 (s, 6 H), 3.79 (s, 3 H), 1.60 (d, *J* = 7.2 Hz, 3 H); **^{13}C NMR** (151 MHz, $CDCl_3$): δ 158.0, 153.2, 142.6, 138.5, 136.4, 128.5, 113.9, 104.8, 60.9, 56.2, 55.4, 44.3, 22.3; **FTIR** (Cast film, cm^{-1}): 2962 (w), 2835 (w), 1589 (m), 1511 (m), 1244 (m), 1128 (s), 1009 (m), 832 (w); **HRMS** (ESI) for $C_{18}H_{22}O_4Na$ $[M+Na]^+$: Calculated: 325.1416; Found: 325.1412. Spectral data were in agreement with the literature.[29]

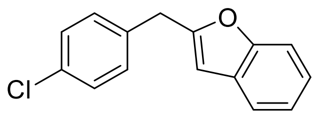

2-(4-Chlorobenzyl)benzofuran (7t): Prepared according to **GP8** from alcohol **6t** (161 mg, 0.62 mmol), catalyst **3** (1.2 mg, 0.0062 mmol, 1 mol%) and triethylsilane (109 µL, 0.682 mmol, 1.10 equiv) for 90 minutes. Purification by column chromatography (hexane) afforded the title compound as white solid (106 mg, 70%). **mp** = 68.6 – 70.1 °C; **^{1}H NMR** (500 MHz, $CDCl_3$): δ 7.48 (dd, *J* = 7.2, 1.8 Hz, 1 H), 7.41 (d, *J* = 7.7 Hz, 1 H), 7.30 (d, *J* = 8.6 Hz, 2 H), 7.25 – 7.21 (m, 3 H), 7.19 (td, *J* = 7.2, 1.2 Hz, 1 H), 6.38 (dd, *J* = 2.0, 1.0 Hz, 1 H), 4.08 (s, 2 H); **^{13}C NMR** (126 MHz, $CDCl_3$): δ 157.2, 155.1, 135.8, 132.8, 130.4, 128.9, 128.8, 123.8, 122.8, 120.6, 111.1, 103.7, 34.5; **FTIR** (Cast film, cm^{-1}): 3054 (w), 2919 (w), 1587 (w), 1491 (s), 1454 (s), 1253 (m), 1105 (m), 1016 (m), 796 (s), 751 (s); **HRMS** (ESI) for $C_{15}H_{11}OCl$: Calculated: 242.0498; Found: 242.0492. Spectral data were in agreement with the literature.[40]

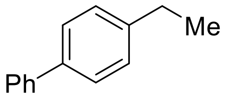

4-Ethyl-1,1'-biphenyl (7u): Prepared according to **GP8** from alcohol **6u** (198 mg, 1.00 mmol), catalyst **3** (10.0 mg, 0.050 mmol, 5 mol%) and triethylsilane (176 µL, 1.10 mmol, 1.10 equiv) with a reaction time of 3 hours. Purification by column chromatography (hexane) afforded the title compound as a white solid (147 mg, 81%). **^{1}H NMR** (500 MHz, $CDCl_3$): δ 7.59 (dd, *J* = 7.2, 1.2 Hz, 2 H), 7.53 (d, *J* = 8.4 Hz, 2 H), 7.44 (t, *J* = 7.7 Hz, 2 H), 7.33 (tt, *J* = 7.4, 1.3 Hz, 1 H), 7.29 (d, *J* = 8.4 Hz, 2 H), 2.71 (q, *J* = 7.6 Hz, 1 H), 1.29 (t, *J* = 7.6 Hz, 3 H); **^{13}C NMR** (126 MHz, $CDCl_3$): δ 143.5, 141.3, 138.8, 128.8, 128.4, 127.23, 127.16, 127.11, 28.7, 15.7. Spectral data were in agreement with the literature.[41]

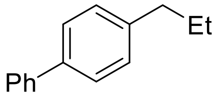

4-Propyl-1,1'-biphenyl (7v): Prepared according to **GP8** from alcohol **6v** (212 mg, 1.00 mmol), catalyst **3** (10.0 mg, 0.050 mmol, 5 mol%) and triethylsilane (176 µL, 1.10 mmol, 1.10 equiv) with a reaction time of 3 hours. Purification by column chromatography (hexane) afforded the title compound as a clear oil (142 mg, 68%). **^{1}H NMR** (500 MHz, $CDCl_3$): δ 7.63 (dd, *J* = 8.4, 1.1 Hz, 2 H), 7.56 (d, *J* = 8.4 Hz, 2 H), 7.47 (t, *J* = 7.4 Hz, 2 H), 7.37 (tt, *J* = 7.4, 1.3 Hz, 1 H), 7.30 (d, *J* = 7.6 Hz, 2 H), 2.68 (t, *J* = 7.7 Hz, 2 H), 1.73 (h, *J* = 7.4 Hz, 2 H), 1.03 (t, *J* = 7.3 Hz, 3 H) ; **^{13}C NMR** (126 MHz, $CDCl_3$): δ 142.0, 141.4, 138.7, 129.0, 128.8, 127.14, 127.11, 127.09, 37.8, 24.7, 14.0. Spectral data were in agreement with the literature.[42]

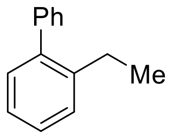

2-Ethyl-1,1'-biphenyl (7w): Prepared according to **GP8** from alcohol **6w** (198 mg, 1.00 mmol), catalyst **3** (10.0 mg, 0.050 mmol, 5 mol%) and triethylsilane (176 µL, 1.10 mmol, 1.10 equiv) with

a reaction time of 3 hours. Purification by column chromatography (hexane) afforded the title compound as a clear oil (107 mg, 59%). **^{1}H NMR** (500 MHz, $CDCl_3$): δ 7.42 (tt, *J* = 6.9 Hz, 2.8 Hz, 2 H), 7.37 – 7.2 (m, 5 H), 7.25 – 7.20 (m, 2 H), 2.61 (q, *J* = 7.6 Hz, 2 H), 1.11 (t, *J* = 7.6 Hz, 3 H); **^{13}C NMR** (126 MHz, $CDCl_3$): δ 142.1, 141.79, 141.76, 130.1, 129.4, 128.7, 128.1, 127.6, 126.9, 125.7, 26.3, 15.8. Spectral data were in agreement with the literature.[41]

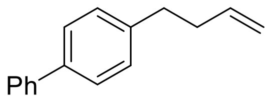

4-(But-3-en-1-yl)-1,1'-biphenyl (7x): Prepared according to **GP8** from alcohol **6x** (224 mg, 1.00 mmol), catalyst **3** (10.0 mg, 0.050 mmol, 5 mol%) and triethylsilane (176 µL, 1.10 mmol, 1.10 equiv) with a reaction time of 18 hours. Purification by column chromatography (hexane) afforded the title compound as a clear oil (145 mg, 70%). **^{1}H NMR** (500 MHz, $CDCl_3$): δ 7.60 (dd, *J* = 8.4, 1.2 Hz, 2 H), 7.54 (d, *J* = 8.4 Hz, 2 H), 7.44 (t, *J* = 7.7 Hz, 2 H), 7.34 (tt, *J* = 7.3, 1.3 Hz, 1 H), 7.28 (d, *J* = 8.4 Hz, 2 H), 5.91 (ddt, *J* = 16.9, 10.3, 6.6 Hz, 1 H), 5.09 (dq, *J* = 17.1, 1.7 Hz, 1 H), 5.04 – 5.01 (m, 1 H), 2.78 (t, *J* = 7.8 Hz, 2 H), 2.44 (tdt, *J* = 7.9, 6.6, 1.5 Hz, 2 H); **^{13}C NMR** (126 MHz, $CDCl_3$): δ 141.2, 141.1, 138.9, 138.2, 129.0, 128.9, 127.19, 127.15 (x 2), 115.2, 35.6, 35.2. Spectral data were in agreement with the literature.[43]

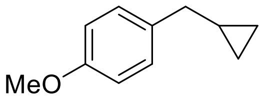

1-(Cyclopropylmethyl)-4-methoxybenzene (7y): Prepared according to **GP8** from ether **6y** (192 mg, 1.00 mmol), catalyst **3** (10.0 mg, 0.050 mmol, 5 mol%) and triethylsilane (176 µL, 1.10 mmol, 1.10 equiv) with a reaction time of 3 hours. Purification by column chromatography (hexane) afforded the title compound as a clear oil (114 mg, 70%). **^{1}H NMR** (700 MHz, $CDCl_3$): δ 7.19 (d, *J* = 8.8 Hz, 2 H), 6.85 (d, *J* = 8.7 Hz, 2 H), 3.80 (s, 3 H), 2.50 (d, *J* = 6.9 Hz, 2 H), 0.99 – 0.92 (m, 1 H), 0.53 – 0.50 (m, 2 H), 0.20 – 0.18 (m, 2 H) ; **^{13}C NMR** (125 MHz, $CDCl_3$): δ 158.0, 134.4, 129.4, 113.8, 55.4, 39.6, 12.2, 4.7. Spectral data were in agreement with the literature.[44]

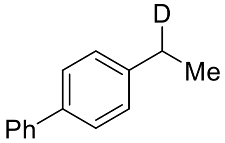

4-(1-Deuteroethyl)-1,1'-biphenyl (7z): Prepared according to **GP8** from alcohol **6z** (199 mg, 1.00 mmol), catalyst **3** (10.0 mg, 0.050 mmol, 5 mol%) and triethylsilane (176 µL, 1.10 mmol, 1.10 equiv) with a reaction time of 3 hours. Purification by column chromatography (hexane) afforded the title compound as a white solid (141 mg, 77%). **^{1}H NMR** (500 MHz, $CDCl_3$): δ 7.61 (dd, *J* = 8.4, 1.4 Hz, 2 H), 7.54 (d, *J* = 8.3 Hz, 2 H), 7.45 (t, *J* = 7.8 Hz, 2 H), 7.34 (tt, *J* = 7.4, 1.3 Hz, 1 H), 7.30 (d, *J* = 8.0 Hz, 2 H), 2.74 – 2.67 (m, 1 H), 1.29 (d, *J* = 7.6 Hz, 3 H); **^{13}C NMR** (126 MHz, $CDCl_3$): δ 143.5, 141.4, 138.8, 128.8, 128.4, 127.22, 127.16, 127.1, 28.31 (1:1:1 triplet, *J* = 19.3 Hz), 15.6. Traces of the non-deuterated product are observed (15.7 ppm, 28.7 ppm) and are denoted with an * in the spectra found in Section 12. Spectral data were in agreement with the literature.[45]

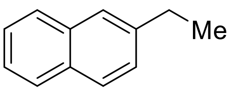

2-Ethylnaphthalene (7aa): Prepared according to **GP8** from alcohol **6aa** (172 mg, 1.00 mmol), catalyst **3** (10.0 mg, 0.050 mmol, 5 mol%) and triethylsilane (176 μL, 1.10 mmol, 1.10 equiv) with a reaction time of 3 hours. Purification by column chromatography (100:1 hexane/EtOAc) afforded the title compound as a clear oil (111 mg, 71%). **^{1}H NMR** (600 MHz, $CDCl_3$): δ 7.82 – 7.78 (m, 3 H), 7.64 (s, 1 H), 7.46 (td, *J* = 7.4, 1.3 Hz, 1 H), 7.43 (td, *J* = 7.4, 1.5 Hz, 1 H), 7.37 (dd, *J* = 8.3, 1.7 Hz, 1 H), 2.34 (q, *J* = 7.6 Hz, 2 H), 1.35 (t, *J* = 7.6 Hz, 3 H); **^{13}C NMR** (151 MHz, $CDCl_3$): δ 141.9, 133.9, 132.1, 127.9, 127.7, 127.6, 127.2, 126.0, 125.7, 125.2, 29.2, 15.7. Spectral data were in agreement with the literature.[46]

8.2 Reductive Deoxygenation of Benzylic Ketones (Figure 6)

General Procedure for the reductive deoxygenation of benzylic ketones (GP9)

A vial equipped with a stir bar was charged with ketone **8**, catalyst **3** (5 mol%), 1,1,3,3-tetramethyldisiloxane (TMDSO) (2.2 equiv) and HFIP (0.5 M in ketone **8**). The reaction was stirred at room temperature for 24 hours, after which it was concentrated by rotary evaporation. Purification by column chromatography afforded the reduction product **7**.

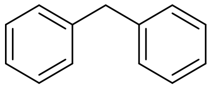

Diphenylmethane (7a): Prepared according to **GP9** from ketone **6a** (164 mg, 0.900 mmol), catalyst **3** (9.0 mg, 0.045 mmol, 5 mol%) and TMDSO (348 μL, 1.98 mmol, 2.20 equiv). Purification by column chromatography (hexane) afforded the title compound as a clear oil (135 mg, 89%). Spectral data match that reported in section 8.1.

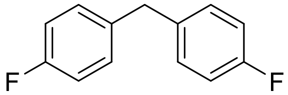

Bis(4-fluorophenyl)methane (7b): Prepared according to **GP9** from ketone **8b** (165 mg, 0.750 mmol), catalyst **3** (7.5 mg, 0.038 mmol, 5 mol%) and TMDSO (292 μL, 1.65 mmol, 2.20 equiv). Purification by column chromatography (hexane) afforded the title compound as a clear oil (101 mg, 65%). Spectral data match that reported in section 8.1.

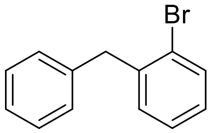

1-Benzyl-2-bromobenzene (7e): Prepared according to **GP9** from ketone **8e** (196 mg, 0.750 mmol), catalyst **3** (7.5 mg, 0.038 mmol, 5 mol%) and TMDSO (292 μL, 1.65 mmol, 2.20 equiv). Purification by column chromatography (hexane) afforded the title compound as a clear oil (142 mg, 77%). Spectral data match that reported in section 8.1.

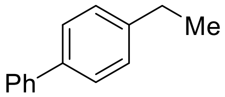

4-Ethyl-1,1'-biphenyl (7u): Prepared according to **GP9** from ketone **8u** (195 mg, 1.00 mmol), catalyst **3** (10.0 mg, 0.050 mmol, 5 mol%) and TMDSO (388 µL, 2.20 mmol, 2.20 equiv). Purification by column chromatography (hexane) afforded the title compound as a white solid (145 mg, 95%). Spectral data match that reported in section 8.1.

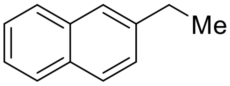

2-Ethylnaphthalene (7aa): Prepared according to **GP9** from ketone **8aa** (170 mg, 1.00 mmol), catalyst **3** (10.0 mg, 0.050 mmol, 5 mol%) and TMDSO (388 µL, 2.20 mmol, 2.20 equiv). Purification by column chromatography (hexane) afforded the title compound as a clear oil (121 mg, 77%). Spectral data match that reported in section 8.1.

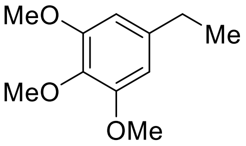

5-Ethyl-1,2,3-trimethoxybenzene (7ab): Prepared according to **GP9** from ketone **8ah** (158 mg, 0.750 mmol), catalyst **3** (7.5 mg, 0.038 mmol, 5 mol%) and TMDSO (292 µL, 1.65 mmol, 2.20 equiv). Purification by column chromatography (6:1 hexane/EtOAc) afforded the title compound as a clear oil (142 mg, 96%). **^{1}H NMR** (600 MHz, $CDCl_3$): δ 6.42 (s, 2 H), 3.86 (s, 6 H), 3.82 (s, 3 H), 2.60 (q, *J* = 7.6 Hz, 2 H), 1.24 (t, *J* = 7.6 Hz, 3 H); **^{13}C NMR** (151 MHz, $CDCl_3$): 153.2, 140.2, 136.1, 104.9, 61.0, 56.2, 29.4, 15.8. Spectral data were in agreement with the literature.[47]

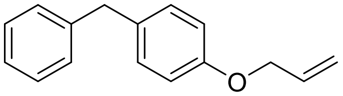

1-(Allyloxy)-4-benzylbenzene (7ac): Prepared according to **GP9** from ketone **8ab** (178 mg, 0.750 mmol), catalyst **3** (7.5 mg, 0.038 mmol, 5 mol%) and TMDSO (292 µL, 1.65 mmol, 2.20 equiv). Purification by column chromatography (gradient 200:1 to 50:1 hexane/EtOAc) afforded the title compound as a clear oil (157 mg, 94%). **^{1}H NMR** (600 MHz, $CDCl_3$): δ 7.29 (t, *J* = 7.4 Hz, 2 H), 7.22 – 7.18 (m, 3 H), 7.11 (d, *J* = 8.9 Hz, 2 H), 6.86 (d, *J* = 8.8 Hz, 2 H), 6.06 (ddt, *J* = 17.2, 10.5, 5.3 Hz, 1 H), 5.42 (dq, *J* = 17.2, 1.6 Hz, 1 H), 5.29 (dq, *J* = 10.5, 1.4 Hz, 1 H), 4.52 (dt, *J* = 5.3, 1.6 Hz, 2 H), 3.94 (s, 2 H); **^{13}C NMR** (151 MHz, $CDCl_3$): δ 157.2, 141.7, 133.60, 133.58, 130.0, 129.0, 128.6, 126.1, 117.7, 114.9, 69.0, 41.2. Spectral data were in agreement with the literature.[48]

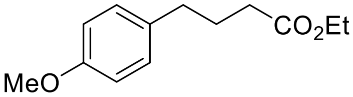

Ethyl 4-(4-methoxyphenyl)butanoate (7ad): Prepared according to **GP9** from ketone **8ae** (177 mg, 0.750 mmol), catalyst **3** (7.5 mg, 0.038 mmol, 5 mol%) and TMDSO (292 µL, 1.65 mmol,

2.20 equiv). Purification by column chromatography (40:1 hexane/EtOAc) afforded the title compound as a clear oil (155 mg, 93%). **^{1}H NMR** (500 MHz, $CDCl_3$): δ 7.09 (d, *J* = 8.7 Hz, 2 H), 6.83 (d, *J* = 8.7 Hz, 2 H), 4.12 (q, *J* = 7.2 Hz, 2 H), 3.79 (s, 3 H), 2.59 (t, *J* = 7.6 Hz, 2 H), 2.30 (t, *J* = 7.5 Hz, 2 H), 1.92 (p, *J* = 7.6 Hz, 2 H), 1.25 (t, *J* = 7.2 Hz, 3 H); **^{13}C NMR** (126 MHz, $CDCl_3$): δ 173.7, 158.0, 133.7, 129.5, 113.9, 60.4, 55.4, 34.4, 33.8, 26.9, 14.4. Spectral data were in agreement with the literature.[49]

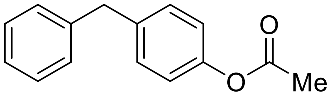

4-Benzylphenyl acetate (7ae): Prepared according to **GP9** from ketone **8ad** (180 mg, 0.750 mmol), catalyst **3** (7.5 mg, 0.038 mmol, 5 mol%) and TMDSO (292 µL, 1.65 mmol, 2.20 equiv). Purification by column chromatography (40:1 hexane/EtOAc) afforded the title compound as a clear oil (145 mg, 85%). **^{1}H NMR** (500 MHz, $CDCl_3$): δ 7.30 (t, *J* = 7.6 Hz, 2 H), 7.23 – 7.19 (m, 5 H), 7.01 (d, *J* = 8.5 Hz, 2 H), 3.98 (s, 2 H), 2.29 (s, 3 H); **^{13}C NMR** (126 MHz): δ 169.7, 149.1, 140.9, 138.8, 130.0, 129.1, 128.7, 126.3, 121.6, 41.4, 21.3. Spectral data were in agreement with the literature.[50]

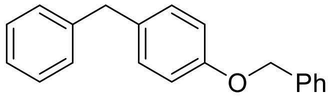

1-Benzyl-4-(benzyloxy)benzene (7af): Prepared according to **GP9** from ketone **8ac** (216 mg, 0.750 mmol), catalyst **3** (7.5 mg, 0.038 mmol, 5 mol%) and TMDSO (292 µL, 1.65 mmol, 2.20 equiv). Purification by column chromatography (gradient 100:1 to 20:1 hexane/EtOAc) afforded the title compound as a white solid (144 mg, 70%). **^{1}H NMR** (600 MHz, $CDCl_3$): δ 7.45 (d, *J* = 6.7 Hz, 2 H), 7.40 (t, *J* = 7.5 Hz, 2 H), 7.35 (tt, *J* = 7.4, 1.4 Hz, 1 H), 7.30 (t, *J* = 7.5 Hz, 2 H), 7.22 – 7.19 (m, 3 H), 7.12 (d, *J* = 8.8 Hz, 2 H), 6.93 (d, *J* = 8.8 Hz, 2 H), 5.06 (s, 2 H), 3.95 (s, 2 H); **^{13}C NMR** (151 MHz, $CDCl_3$): δ 157.4, 141.7, 137.3, 133.7, 130.0, 129.0, 128.7, 128.6, 128.0, 127.6, 126.1, 115.0, 70.2, 41.2. Spectral data were in agreement with the literature.[51]

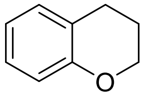

Chromane (7ag): Prepared according to **GP9** from ketone **8af** (111 mg, 0.750 mmol), catalyst **3** (7.5 mg, 0.038 mmol, 5 mol%) and TMDSO (292 µL, 1.65 mmol, 2.20 equiv). Purification by column chromatography (35:1 hexane/EtOAc) afforded the title compound as a clear oil (45 mg, 45%). **^{1}H NMR** (500 MHz, $CDCl_3$): δ 7.09 (t, *J* = 8.2 Hz, 1 H), 7.04 (d, *J* = 7.8 Hz, 1 H), 6.84 (td, *J* = 7.1, 1.0 Hz, 1 H), 6.81 (d, *J* = 8.2 Hz, 1 H), 4.19 (t, *J* = 5.2 Hz, 2 H), 2.80 (t, *J* = 6.6 Hz, 2 H), 2.04 – 2.00 (m, 2 H); **^{13}C NMR** (126 MHz, $CDCl_3$): δ 155.1, 129.9, 127.3, 122.4, 120.2, 116.8, 66.6, 25.0, 22.5. Spectral data were in agreement with the literature.[52]

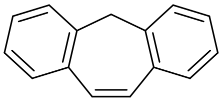

5H-Dibenzo[a,d][7]annulene (7ah): Prepared according to **GP9** from ketone **8ag** (1.65 g, 8.00 mmol), catalyst **3** (16.0 mg, 0.400 mmol, 5 mol%) and TMDSO (3.1 mL, 17.6 mmol, 2.20 equiv). Purification by column chromatography (hexane) afforded the title compound as a white solid (1.41 g, 92%). **^{1}H NMR** (600 MHz, $CDCl_3$): δ 7.34 – 7.29 (m, 6 H), 7.22 (td, J = 6.6, 1.7 Hz, 2 H), 3.76 (s, 2 H); **^{13}C NMR** (151 MHz, $CDCl_3$): δ 138.3, 135.3, 131.7, 128.6, 128.2, 128.0, 126.2, 41.8. Spectral data were in agreement with the literature.[53]

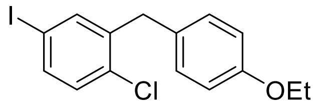

1-Chloro-2-(4-ethoxybenzyl)-4-iodobenzene (7ai): Prepared according to **GP9** from ketone **8ai** (105 mg, 0.27 mmol), catalyst **3** (2.7 mg, 0.014 mmol, 5 mol%) and TMDSO (104 µL, 0.594 mmol, 2.20 equiv). Purification by column chromatography (50:1 hexane/EtOAc) afforded the title compound as a white solid (87 mg, 87%). **^{1}H NMR** (500 MHz, $CDCl_3$): δ 7.47 – 7.45 (m, 2 H), 7.10 – 7.07 (m, 3 H), 6.84 (d, J = 8.8 Hz, 2 H), 4.02 (q, J = 7.0 Hz, 2 H), 3.97 (s, 2 H), 1.41 (t, J = 7.0 Hz, 3 H); **^{13}C NMR** (126 MHz, $CDCl_3$): δ 157.8, 141.7, 139.7, 136.7, 134.4, 131.3, 130.6, 130.0, 114.8, 91.8, 63.6, 38.2, 15.0. Spectral data were in agreement with the literature.[28]

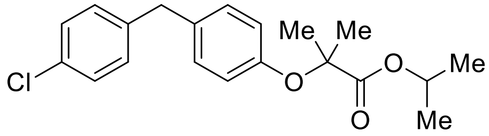

Isopropyl 2-(4-(4-chlorobenzyl)phenoxy)-2-methylpropanoate (7aj): Prepared according to **GP9** from ketone **8ai** (271 mg, 0.750 mmol), catalyst **3** (7.5 mg, 0.038 mmol, 5 mol%) and TMDSO (292 µL, 1.65 mmol, 2.20 equiv). Purification by column chromatography (hexane to 25:1 hexane/EtOAc) afforded the title compound as a clear oil (219 mg, 84%). **^{1}H NMR** (600 MHz, $CDCl_3$): δ 7.24 (d, J = 8.4 Hz, 2 H), 7.08 (d, J = 8.5 Hz, 2 H), 7.00 (d, J = 8.7 Hz, 2 H), 6.78 (d, J = 8.7 Hz, 2 H), 5.07 (hept, J = 6.3 Hz, 1 H), 3.87 (s, 2 H), 1.56 (s, 6 H), 1.21 (d, J = 6.2 Hz, 6 H); **^{13}C NMR** (151 MHz, $CDCl_3$): δ 173.9, 154.2, 140.0, 134.2, 132.0, 130.3, 129.6, 128.6, 119.4, 79.2, 69.0, 40.5, 25.5, 21.7; **FTIR** (Cast film, cm^{-1}): 2983 (w), 2939 (w), 1728 (s), 1610 (w), 1508 (s), 1383 (w), 1238 (m), 1179 (m), 1153 (m), 1104 (s), 1015 (w), 812 (w); **HRMS** (ESI) for $C_{20}H_{23}O_3ClNa$ $[M+Na]^+$: Calculated: 369.1233; Found: 369.1227.

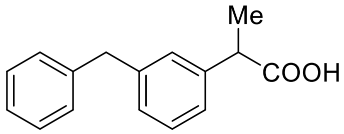

2-(3-Benzylphenyl)propanoic acid (7ak): Prepared according to **GP9** from ketone **8aj** (127 mg, 0.500 mmol), catalyst **3** (5.0 mg, 0.025 mmol, 5 mol%) and TMDSO (194 µL, 2.20 mmol, 2.20 equiv). Purification by column chromatography (2:1 hexane/EtOAc) afforded the title compound as a clear oil (99 mg, 83%). **^{1}H NMR** (500 MHz, $CDCl_3$): δ 7.29 (tt, J = 7.0, 1.8 Hz, 2.0 H), 7.24 – 7.16 (m, 6 H), 7.08 (d, J = 7.8 Hz, 1 H), 3.98 (s, 2 H), 3.71 (q, J = 7.2 Hz, 1 H), 1.50 (d, J = 7.2 Hz, 3 H); **^{13}C NMR** (126 MHz, $CDCl_3$): δ 180.2, 141.7, 140.1, 129.1, 128.9, 128.6, 128.5, 128.2, 126.3, 125.4, 45.4, 42.0, 18.3; **FTIR** (Cast film, cm^{-1}): 3063 (m), 3027 (m), 3000 (br, m), 2631

(w), 1708 (s), 1601 (w), 1453 (w), 1240 (w), 1074 (w), 935 (w), 699 (m); **HRMS** (ESI) for $C_{16}H_{15}O_2$ (M−H)$^-$: Calculated: 239.1078; Found: 239.1079.

8.3 Comparison of Deoxygenation Methods for Alcohol 6h

The reduction of alcohol **6h** was attempted using pTsOH·H_2O as a catalyst in place of boron heterocycle **3** under otherwise standard conditions following **GP6** to assess the mildness of catalyst **3**. Deoxygenation was also attempted using a conventional Lewis acid, Ga(OTf)$_3$, under conditions reported previously in the literature.[54] Crude ^{1}H NMR spectra for the three reactions are shown below, along with a comparison to a purified sample of diarylmethane **7h**.

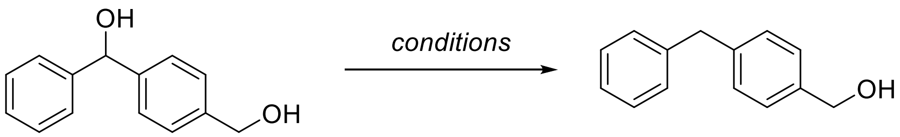

6h **7h**

Conditions A: pTsOH·H_2O (5 mol%), $HSiEt_3$ (1.1 equiv), HFIP/$MeNO_2$ (4:1) [0.5 M], rt, 90 min
Conditions B: Ga(OTf)$_3$ (5 mol%), $HSiMe_2Cl$ (2.5 equiv), CH_2Cl_2 [0.5 M], rt, 1 h
Conditions C: catalyst **3** (1 mol%), $HSiEt_3$ (1.1 equiv), HFIP/$MeNO_2$ (4:1) [0.5 M], rt, 90 min

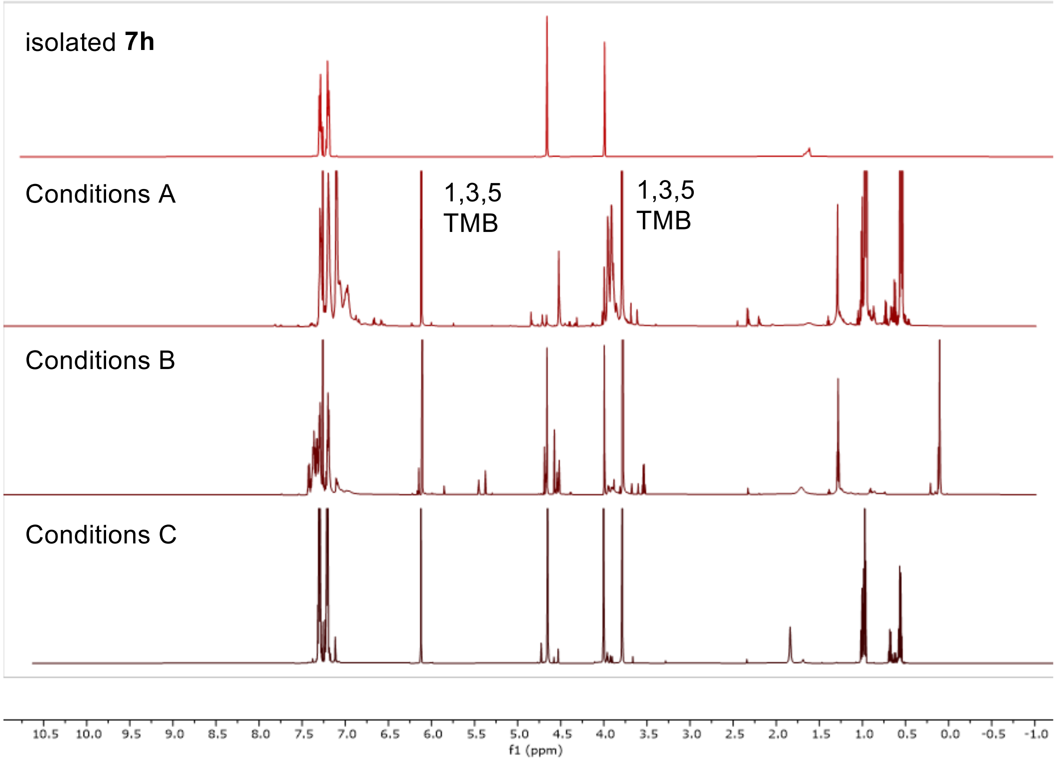

Supplementary Figure 12. Comparison of crude ^{1}H NMR spectra for the reduction of alcohol **6h** under three different conditions.

An expansion of the region from 3.0–6.0 ppm demonstrates that deoxygenation using heterocycle **3** gives a significantly cleaner crude sample.

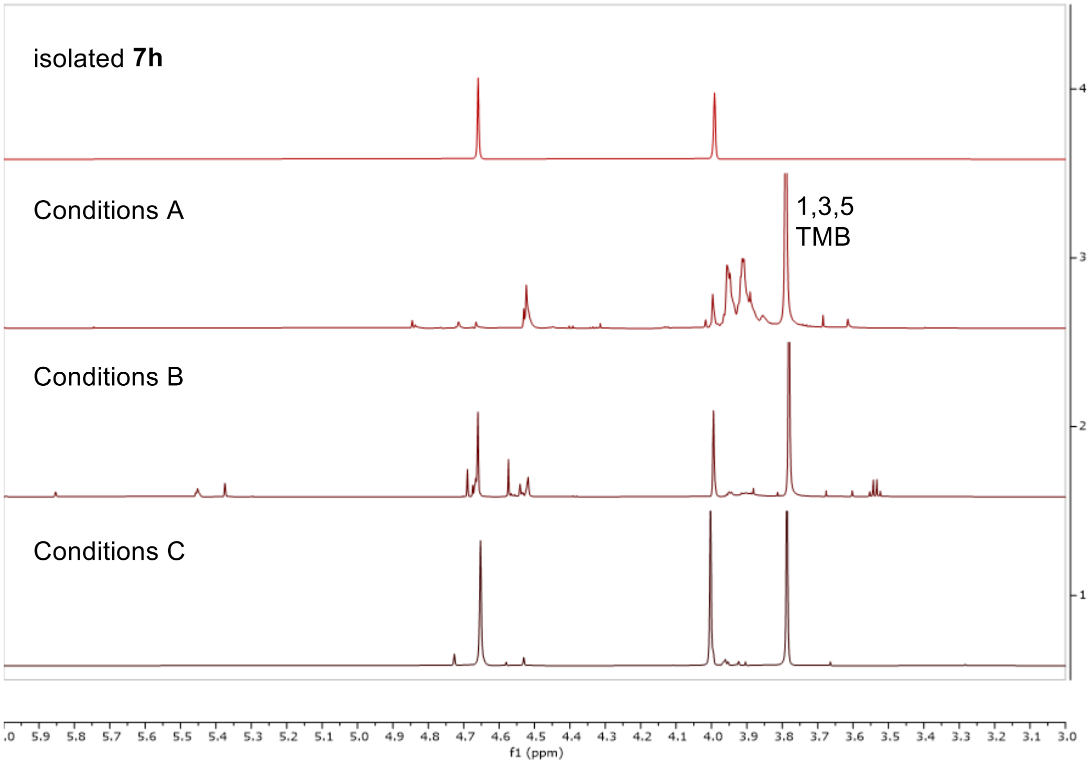

Supplementary Figure 13. Expansion of Supplementary Figure 12 between 3.0–6.0 ppm.

8.4 Unsuccessful Deoxygenation Substrates

The following substrates were unsuccessful in reductive deoxygenation following **GP6** or **GP7** for alcohols/ethers or ketones respectively. Highly electron-deficient alcohols were poor substrates in this methodology, likely due to a decreased stability of the proposed carbocation intermediate and corresponding increase in the barrier for C–O activation. Allylic or propargylic alcohols or ethers with a terminal π-system appeared to decompose to intractable by-products under the reaction conditions. A rapid color change was often observed in reactions of these substrates. A highly activated 1,2-diol was found to undergo competing pinacol rearrangement rather than deoxygenation reduction. Additionally, certain heteroaromatic substrates were unsuccessful.

Decomposition:

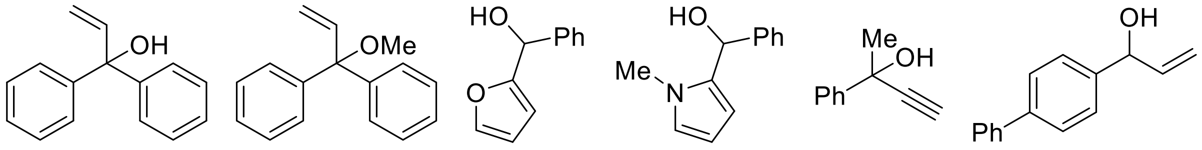

Pinacol rearrangement rather than reduction:

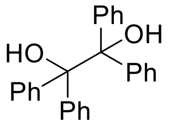

Very low conversion (electron-deficient or heteroatoms):

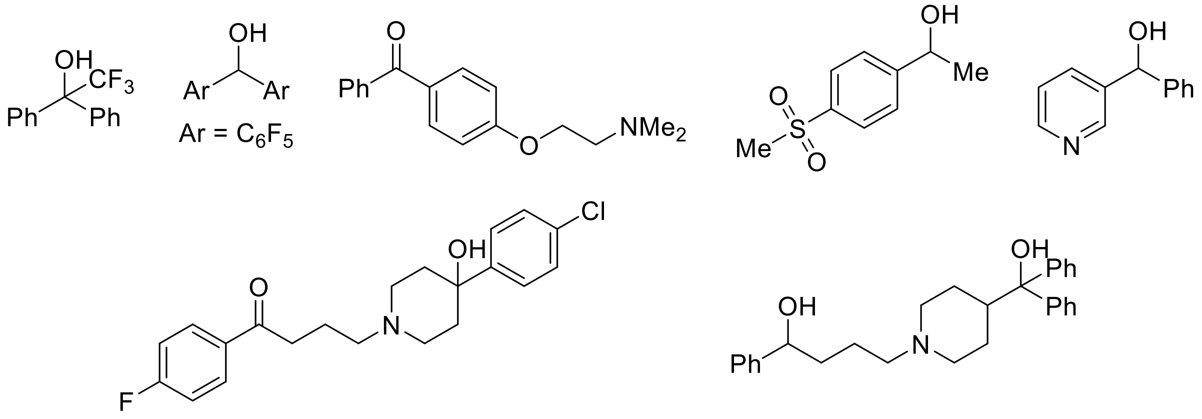

Supplementary Figure 14. Substrates which were unsuccessful in reductive deoxygenation catalyzed by **3**.

9. Deoxygenation of 1-Adamantol, Two-Step Ethylation of Trimethoxybenzene and Comparison of Catalysts in Model Reactions

9.1 Deoxygenation of 1-Adamantol (Figure 7a)

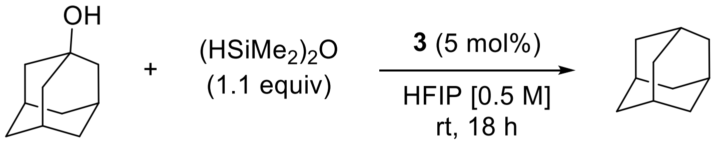

9 **10** 73%

Adamantane (10): A vial equipped with a stir bar was charged with 1-adamantol **9** (114 mg, 0.750 mmol), catalyst **3** (7.5 mg, 0.038 mmol, 5 mol%), TMDSO (146 µL, 0.825 mmol, 1.10 equiv) and HFIP (1.5 mL). The reaction was stirred at room temperature for 18 hours, after which it was concentrated by rotary evaporation. Purification by column chromatography (hexane) afforded the

title compound as a white solid (75 mg, 73%). **^{1}H NMR** (500 MHz, $CDCl_3$): δ 1.89 – 1.86 (m, 4 H), 1.76 (t, *J* = 3.2 Hz, 12 H); **^{13}C NMR** (126 MHz, $CDCl_3$): δ 37.9, 28.5 Spectral data were in agreement with the literature.[55]

9.2 Two-Step Ethylation of Trimethoxybenzene (Figure 7a)

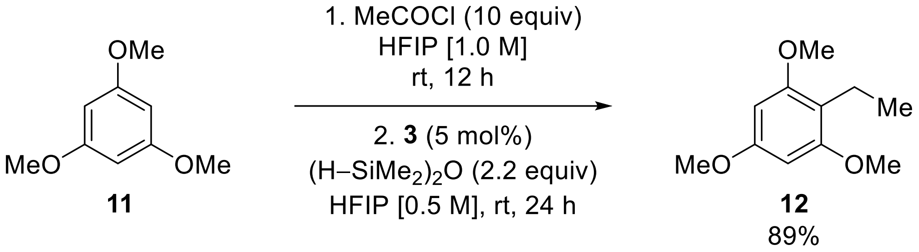

2-Ethyl-1,3,5-trimethoxybenzene (12): A vial equipped with a stir bar was charged with trimethoxybenzene **11** (126 mg, 0.750 mmol), acetyl chloride (535 µL, 7.50 mmol, 10.0 equiv) and HFIP (0.75 mL). The reaction was stirred at room temperature for 12 hours, during which time it turned dark red in color. The reaction was then concentrated by rotary evaporation, after which the vial was charged with catalyst **3** (7.5 mg, 0.038 mmol, 5 mol%), TMDSO (292 µL, 1.65 mmol, 2.20 equiv) and HFIP (1.5 mL). The reaction was stirred at room temperature for 24 hours, after which it was concentrated by rotary evaporation. Purification by column chromatography (10:1 hexane/EtOAc) afforded the title compound as a clear oil (131 mg, 89%). **^{1}H NMR** (500 MHz, $CDCl_3$): δ 6.14 (s, 2 H), 3.81 (s, 3 H), 3.80 (s, 6 H), 2.59 (q, *J* = 7.5 Hz, 2 H), 1.05 (t, *J* = 7.4 Hz, 3 H); **^{13}C NMR** (126 MHz, $CDCl_3$): δ 159.2, 158.8, 113.6, 90.8, 55.9, 55.5, 16.1, 14.3; **FTIR** (Cast film, cm^{-1}): 2999 (w), 2996 (w), 2837 (w), 1611 (m), 1437 (w), 1226 (m), 1140 (m), 1058 (w), 811 (w); **HRMS** (ESI) for $C_{11}H_{16}O_3$: Calculated: 196.1099; Found: 196.1099.

9.3 Comparison of Catalysts in Model Monophosphorylation and Alcohol Deoxygenation Reactions (Figure 7b)

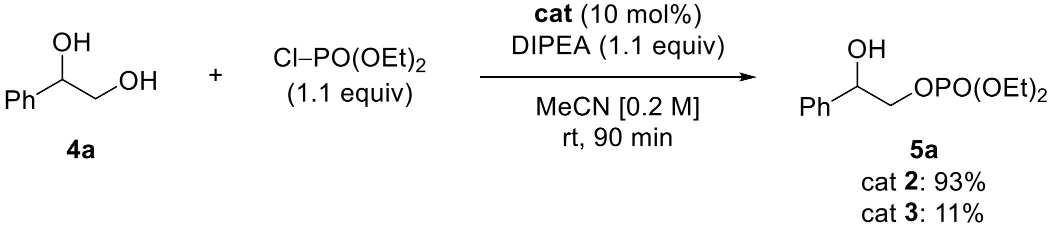

Following **GP2** with diol **4a** (13.8 mg, 0.100 mmol), catalyst (**2**: 1.5 mg or **3**: 2.0 mg, 0.010 mmol 10 mol%), MeCN (0.5 mL), DIPEA (19 µL, 0.11 mmol, 1.1 equiv) and diethyl chlorophosphate (16 µL, 0.11 mmol, 1.1 equiv). Yields were obtained by ^{1}H NMR relative to 1,3,5-trimethoxybenzene as an internal standard (catalyst **2** 93%, catalyst **3** 11%).

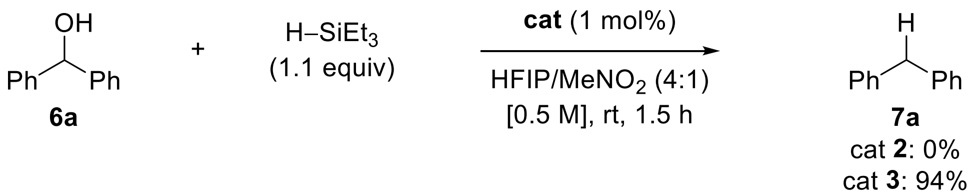

Following **GP6** with alcohol **6a** (27.6 mg, 0.150 mmol), catalyst (**2**: 0.2 mg or **3**: 0.3 mg, 0.002 mmol, 1 mol%), triethylsilane (26 µL, 0.16 mmol, 1.1 equiv), HFIP (240 µL) and $MeNO_2$ (60 µL). Yields were obtained by ^1H NMR relative to 1,3,5-trimethoxybenzene as an internal standard (catalyst **2** 0%, catalyst **3** 94%).

10. Mechanistic and Kinetic Studies of Reductive Deoxygenation

10.1 Ketone Deoxygenation Kinetics (Figure 8a)

A vial equipped with a stir bar was charged with ketone **8a** (1.00 mmol), catalyst **3** (10.0 mg, 0.050 mmol, 5 mol%), TMDSO (388 µL, 2.20 mmol, 2.20 equiv), HFIP (2.0 mL) and 1,4-dinitrobenzene (21.8 mg, 0.130 mmol) as an internal standard. The reaction was stirred at room temperature, and aliquots of 160 µL were taken periodically. These aliquots were diluted with $CHCl_3$ (1.0 mL), concentrated by rotary evaporation, and analyzed by ^1H NMR in $CDCl_3$. Clean conversion of ketone **8a** to diphenylmethane **7a** was observed, and intermediates **6a** or **6aSi** were not detected. An example spectrum is shown below.

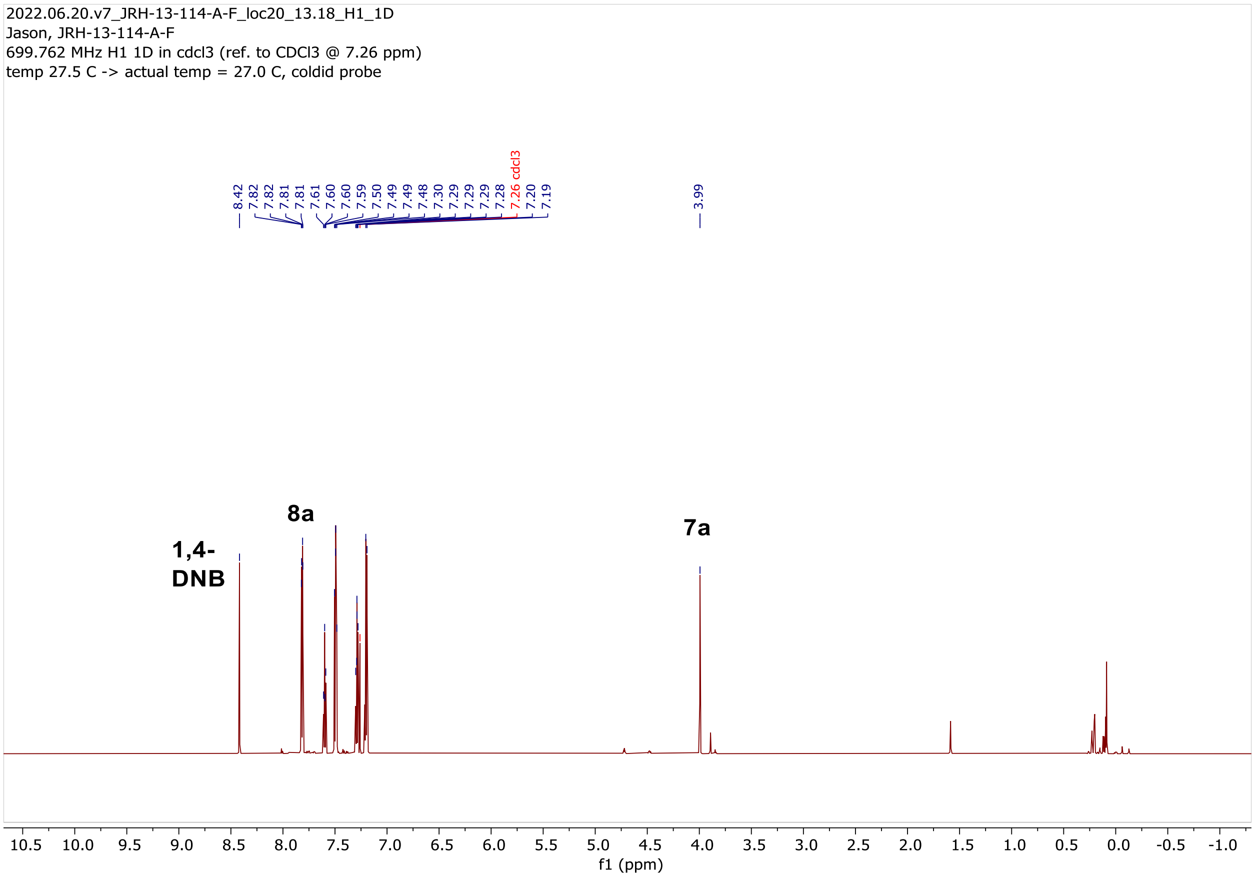

Supplementary Figure 15. Representative ^{1}H NMR spectrum from a reaction aliquot of kinetic monitoring experiments for the reduction of ketone **8a** to diphenylmethane **7a**.

10.2 Evidence for Formation and Hydrolysis of Silyl Ether Intermediates (Figure 8b)

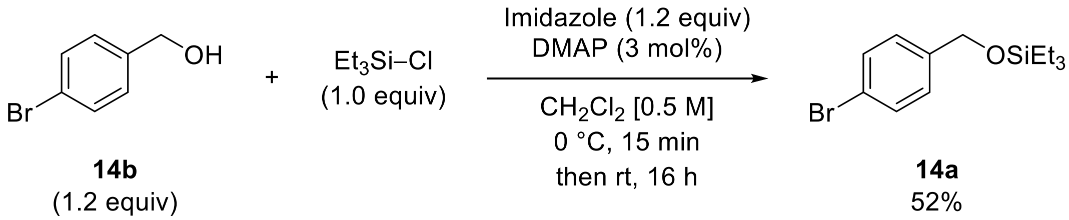

((4-Bromobenzyl)oxy)triethylsilane (14a): Under nitrogen, a flame-dried 50 mL round bottom flask equipped with a stir bar was charged with alcohol **14b** (673 mg, 3.60 mmol, 1.20 equiv), imidazole (245 mg, 3.60 mmol, 1.20 equiv), DMAP (10.0 mg, 0.090 mmol, 3 mol%) and CH_2Cl_2 (10 mL). The flask was cooled to 0 °C in an ice bath, at which point chlorotriethylsilane (508 µL, 3.00 mmol, 1.00 equiv) was added dropwise. The reaction was stirred at 0 °C for an additional 15 minutes, at which point the ice bath was removed and the reaction was stirred for an additional 16 hours. The mixture was then diluted with additional CH_2Cl_2 (15 mL) and H_2O (15 mL). The phases were separated, and the aqueous phase was extracted with an additional 10 mL CH_2Cl_2. The combined organic phases were washed with H_2O (15 mL) and brine (15 mL). The organic layer

was dried over Na_2SO_4, filtered, and concentrated by rotary evaporation. Purification by column chromatography (15:1 hexane/EtOAc) afforded the desired product as a clear oil (465 mg, 1.56 mmol, 52%). **^{1}H NMR** (600 MHz, $CDCl_3$): δ 7.45 (d, J = 8.4 Hz, 2 H), 7.21 (d, J = 8.7 Hz, 2 H), 4.68 (s, 2 H), 0.97 (t, J = 7.9 Hz, 9 H), 0.65 (q, J = 7.9 Hz, 6 H); **^{13}C NMR** (151 MHz, $CDCl_3$): δ 140.5, 131.4, 128.0, 120.8, 64.2, 6.9, 4.6. Spectral data were in agreement with the literature.[56]

Reduction of 4-bromobenzaldehyde using triethylsilane as a reductant was analyzed after 1 hour where both silyl ether **14a** and alcohol **14b** were observed based on crude ^{1}H NMR analysis.

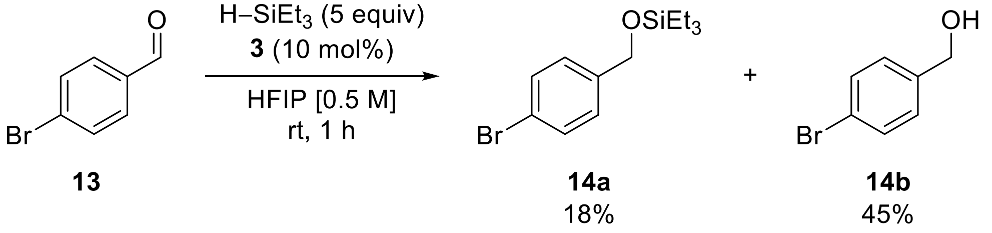

A vial equipped with a stir bar was charged with 4-bromobenzaldehyde **13** (27.8 mg, 0.150 mmol), catalyst **3** (3.0 mg, 0.015 mmol, 10 mol%), triethylsilane (120 µL, 0.750 mmol, 5.0 equiv) and HFIP (300 µL). The vial was then capped and stirred at room temperature for 1 hour, after which it was diluted with $CHCl_3$ and filtered through a small pipette of silica (approximately 1 cm high) with $CHCl_3$ washings to remove insoluble components. The mixture was concentrated by rotary evaporation, and yields were obtained by ^{1}H NMR relative to 1,3,5-trimethoxybenzene as an internal standard (18% silyl ether **14a**, 45% free alcohol **14b**).

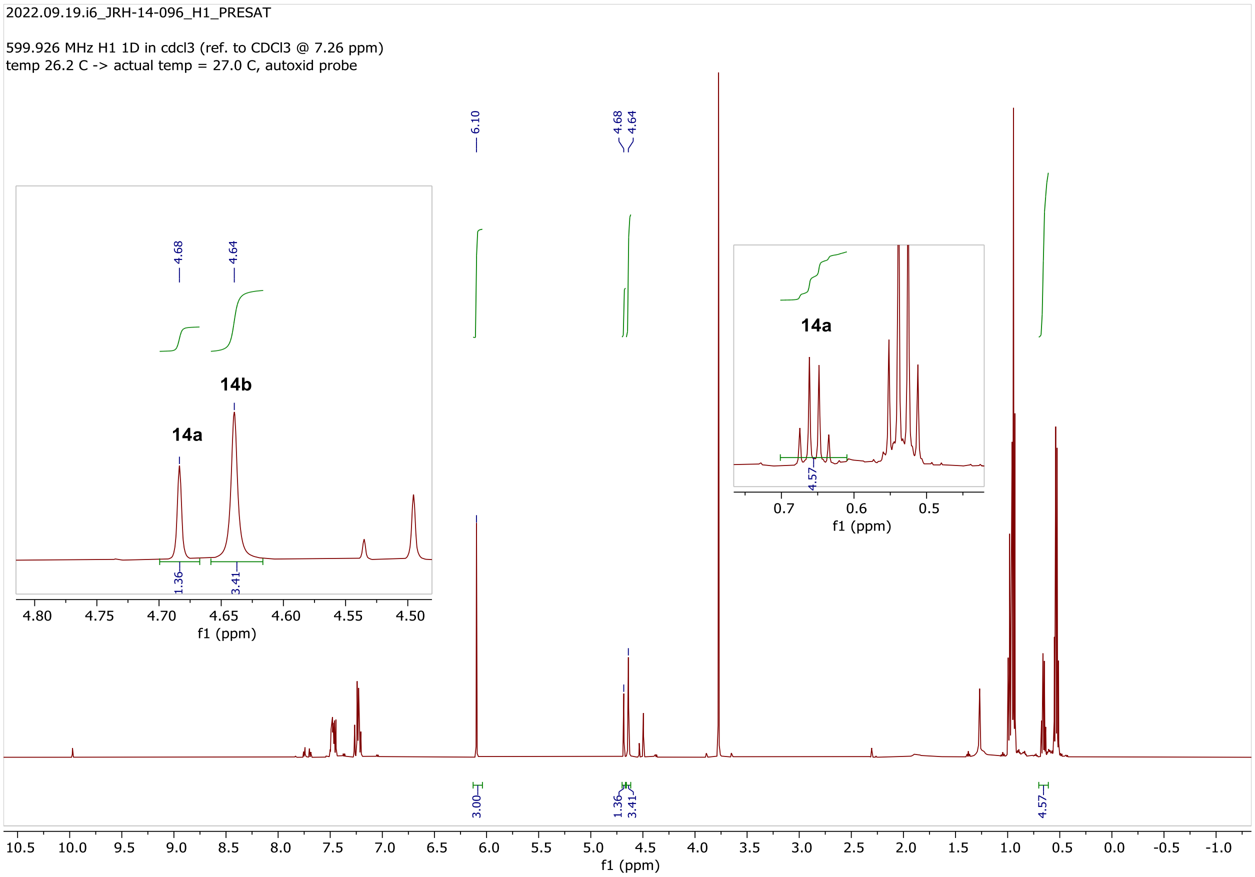

Supplementary Figure 16. ^{1}H NMR for reduction of 4-bromobenzaldehyde with triethylsilane showing the formation of silyl ether **14a** and alcohol **14b**.

When silyl ether **14a** was isolated and subjected to catalyst **3** in HFIP, 87% conversion to free alcohol **14b** was observed.

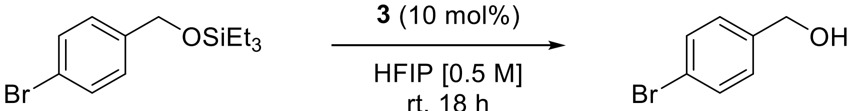

14a **14b** 87%

A vial equipped with a stir bar was charged with silyl ether **14a** (20.0 mg, 0.066 mmol), catalyst **3** (1.3 mg, 0.0066 mmol, 10 mol%) and HFIP (240 μL). The vial was then capped and stirred at room temperature for 18 hours, after which it was diluted with $CHCl_3$ and filtered through a small pipette of silica (approximately 1 cm high) with $CHCl_3$ washings to remove insoluble components. The mixture was concentrated by rotary evaporation, and yields were obtained by ^{1}H NMR relative to 1,3,5-trimethoxybenzene as an internal standard (12% silyl ether **14a**, 87% free alcohol **14b**).

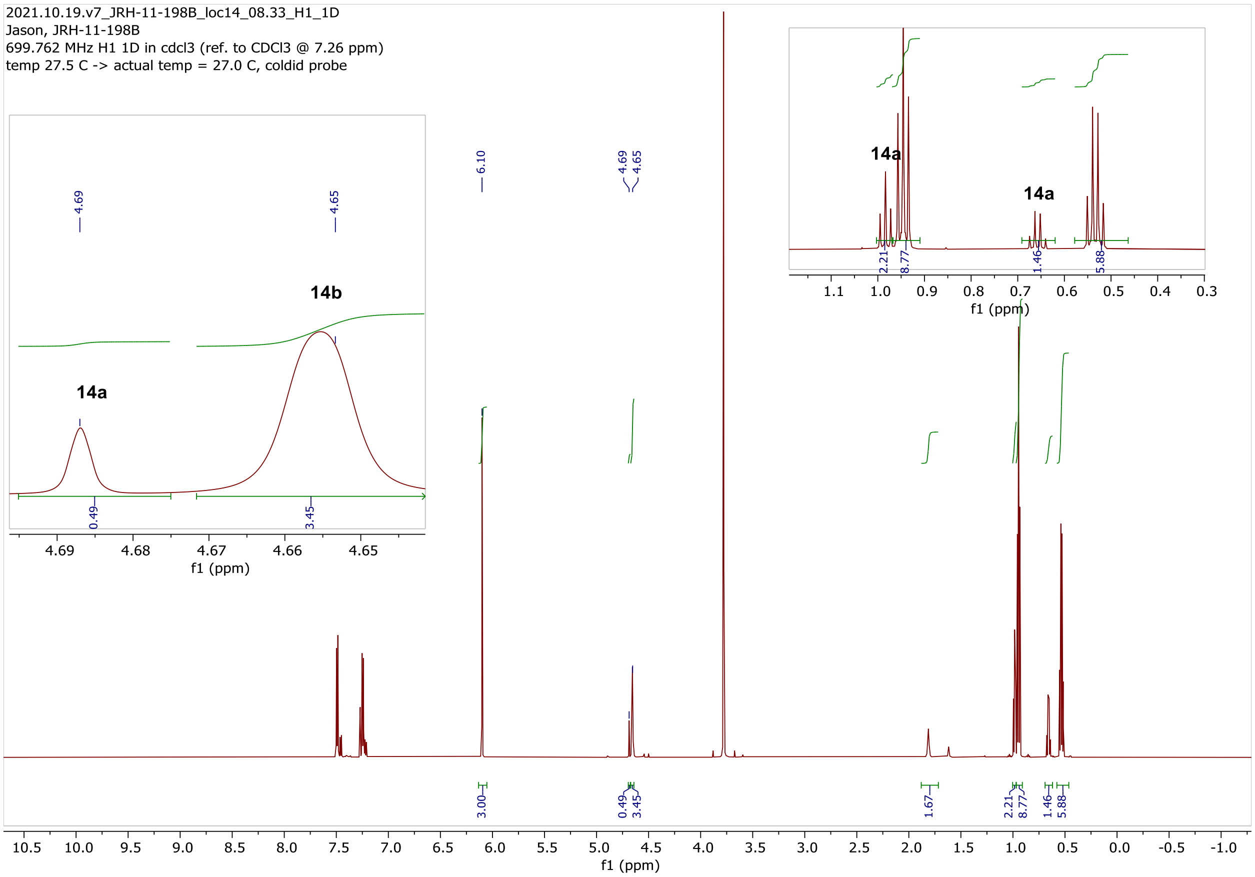

Supplementary Figure 17. ^{1}H NMR demonstrating desilylation of silyl ether **14a** in HFIP catalyzed by boron heterocycle **3**.

When the above experiment was repeated without catalyst **3** (ie. stirring silyl ether **14a** in HFIP alone), alcohol **14b** was not detected.

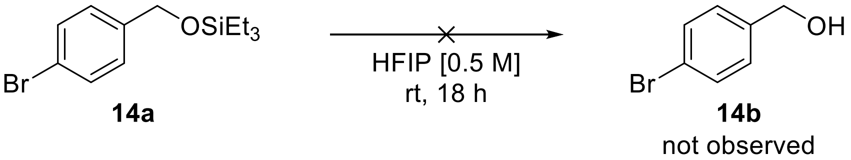

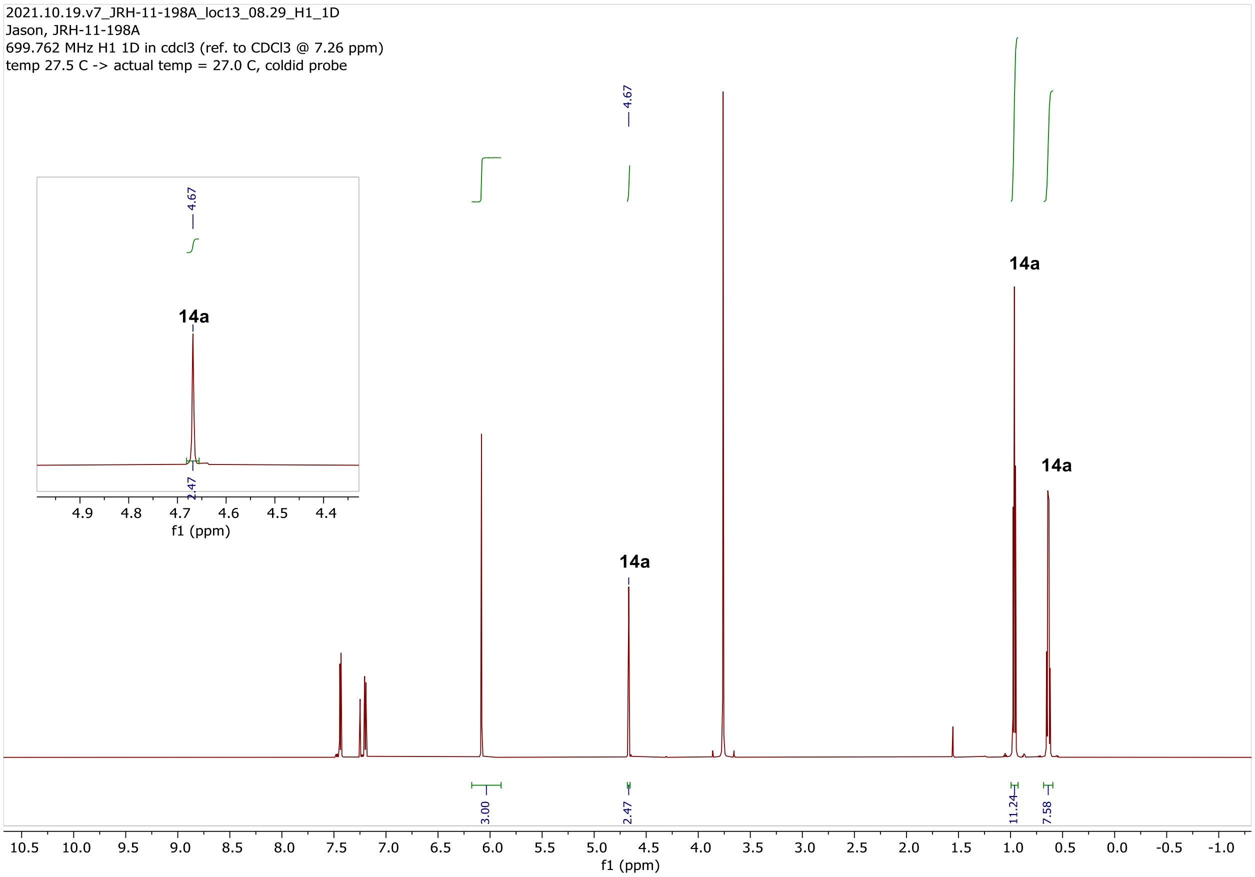

Supplementary Figure 18. ^{1}H NMR after reaction of silyl ether **14a** in HFIP with no added catalyst.

Ether **15** containing both a primary and secondary benzylic ether was prepared and subjected to alcohol deoxygenation conditions. Selective reduction of the secondary benzylic alcohol was observed to afford diarylmethane **7a** in good yield. The liberated primary benzyl alcohol was observed as a mixture of triethylsilyl ether **14a** and free alcohol **14b** based on crude ^{1}H NMR analysis.

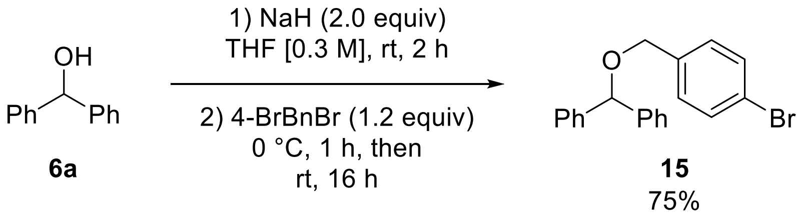

(((4-Bromobenzyl)oxy)methylene)dibenzene (15): Under nitrogen, a flame-dried 100 mL round bottom flask was charged with NaH (240 mg, 60% dispersion in mineral oil, 6.00 mmol, 2.00 equiv) and THF (4 mL). A solution of diphenylmethanol **6a** (552 mg, 3.00 mmol, 1.00 equiv) in THF (6 mL) was added dropwise, after which the reaction was stirred for 2 hours. The flask was then cooled to 0 °C in an ice bath, after which a solution of 4-bromobenzyl bromide (900 mg, 3.60 mmol, 1.20 equiv) in THF (6 mL) was added dropwise. The reaction was stirred at 0 °C for 1 hour,

after which the cooling bath was removed, and the reaction was stirred overnight. The reaction was quenched with saturated $NH_4Cl_{(aq)}$ (25 mL) and extracted with EtOAc (3 × 30 mL). The combined organic phases were washed with water (3 × 25 mL), brine (2 × 25 mL), dried over Na_2SO_4 and filtered. After removal of the solvent by rotary evaporation, purification by column chromatography (gradient 15:1 to 3:1 hexane/CH_2Cl_2) afforded the title compound as a white solid (791 mg, 75%). **^{1}H NMR** (500 MHz, $CDCl_3$): δ 7.50 (d, J = 8.4 Hz, 2 H), 7.41 – 7.35 (m, 8 H), 7.31 – 7.27 (m, 4 H), 5.45 (s, 1 H), 4.52 (s, 2 H); **^{13}C NMR** (126 MHz, $CDCl_3$): δ 142.0, 137.6, 131.6, 129.5, 128.6, 127.7, 127.2, 121.5, 82.8, 69.9. Spectral data were in agreement with the literature.[57]

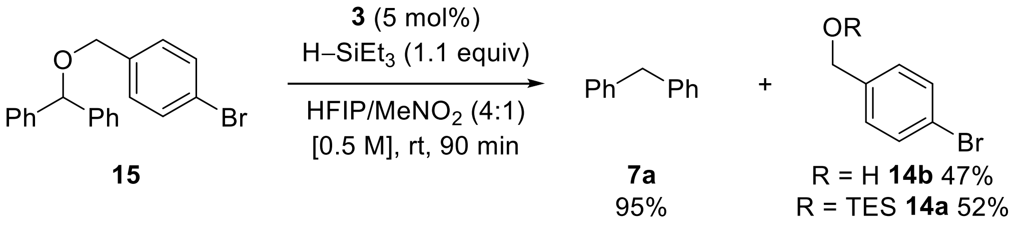

Following **GP6** with ether **15** (35.2 mg, 0.100 mmol), catalyst **3** (1.0 mg, 0.05 mmol, 5 mol%), triethylsilane (18 µL, 0.11 mmol, 1.1 equiv), HFIP (160 µL) and $MeNO_2$ (40 µL). Yields were obtained by ^{1}H NMR relative to 1,3,5-trimethoxybenzene as an internal standard (95% **7a**, 47% **14b**, 52% **14a**).

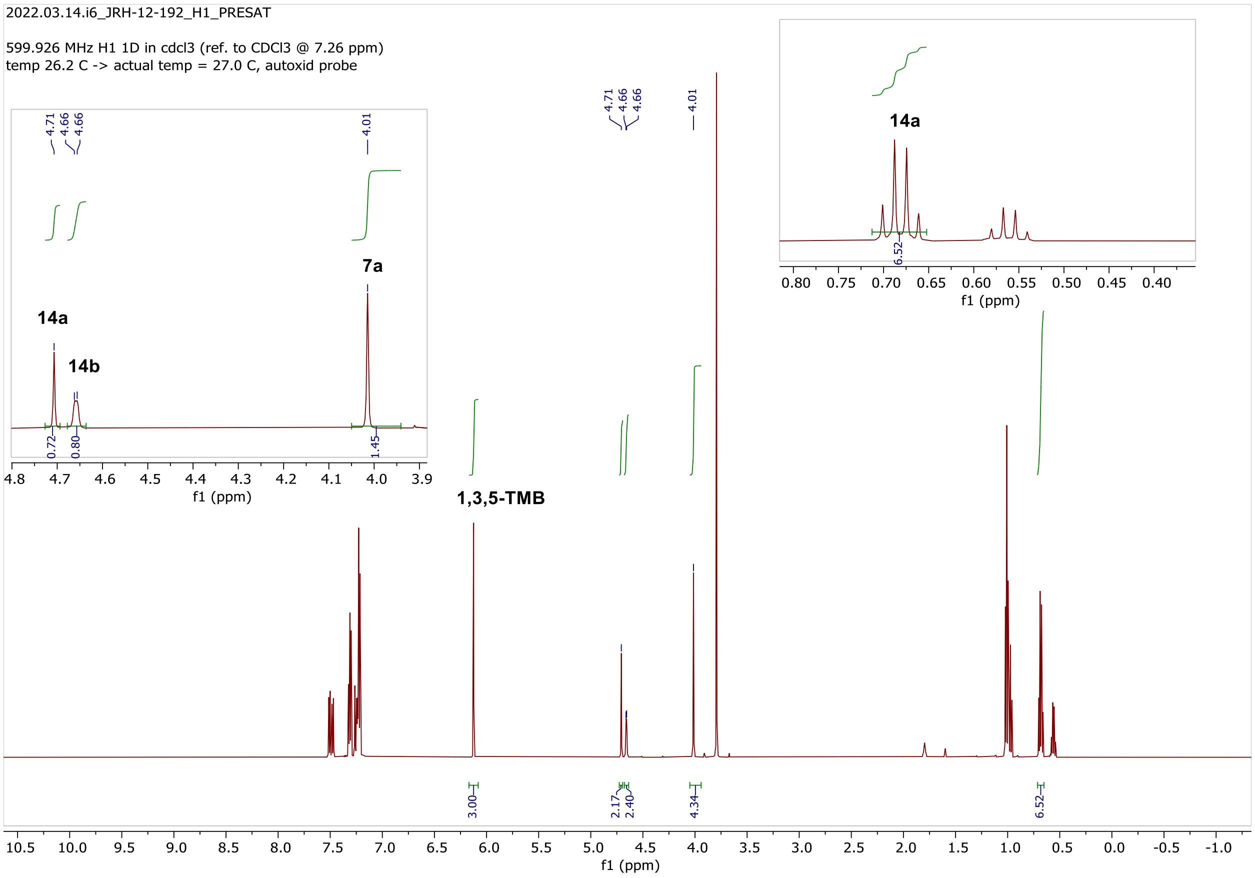

Supplementary Figure 19. ^{1}H NMR for reduction of ether **15** showing the formation of both silyl ether **14a** and alcohol **14b**.

10.3 Kinetics of Ketone Consumption in Deoxygenation Reactions (Figure 8c)

A vial equipped with a stir bar was charged with ketone **8** (1.00 mmol), catalyst **3** (10.0 mg, 0.050 mmol, 5 mol%), TMDSO (388 µL, 2.20 mmol, 2.20 equiv), HFIP (2.0 mL) and 1,4-dinitrobenzene (21.8 mg, 0.130 mmol) as an internal standard. The reaction was stirred at room temperature, and aliquots of 160 µL were taken periodically. These aliquots were diluted with $CHCl_3$ (1.0 mL), concentrated by rotary evaporation, and analyzed by ^{1}H NMR in $CDCl_3$.

Data for ketone **8al** was not collected after the first hour as the reaction mixture became heterogeneous, and aliquots may no longer be representative of reaction progress. The rate of ketone consumption was found to increase in the order of **8al** (slowest) < **8a** < **8u** < **8ah** (fastest) Ketone **8ah** was fully consumed at the time of the first aliquot (15 minutes), an over 10-fold increase in reaction rate relative to **8al**. The formation of product **7al** (9H-fluorene) was assigned in accordance with literature spectral data.[58]

10.4 ^{11}B NMR Analysis of Alcohol Deoxygenation (Figure 9)

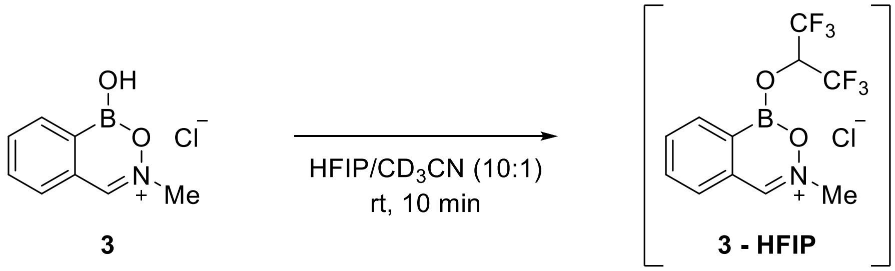

A vial was charged with heterocycle **3** (5.0 mg, 0.025 mmol), HFIP (600 µL) and CD_3CN (60 µL). The solution was transferred to an NMR tube for analysis. In the ^{1}H NMR, a single compound was observed rather than a mixture of compounds, suggesting that a single species forms in solution.

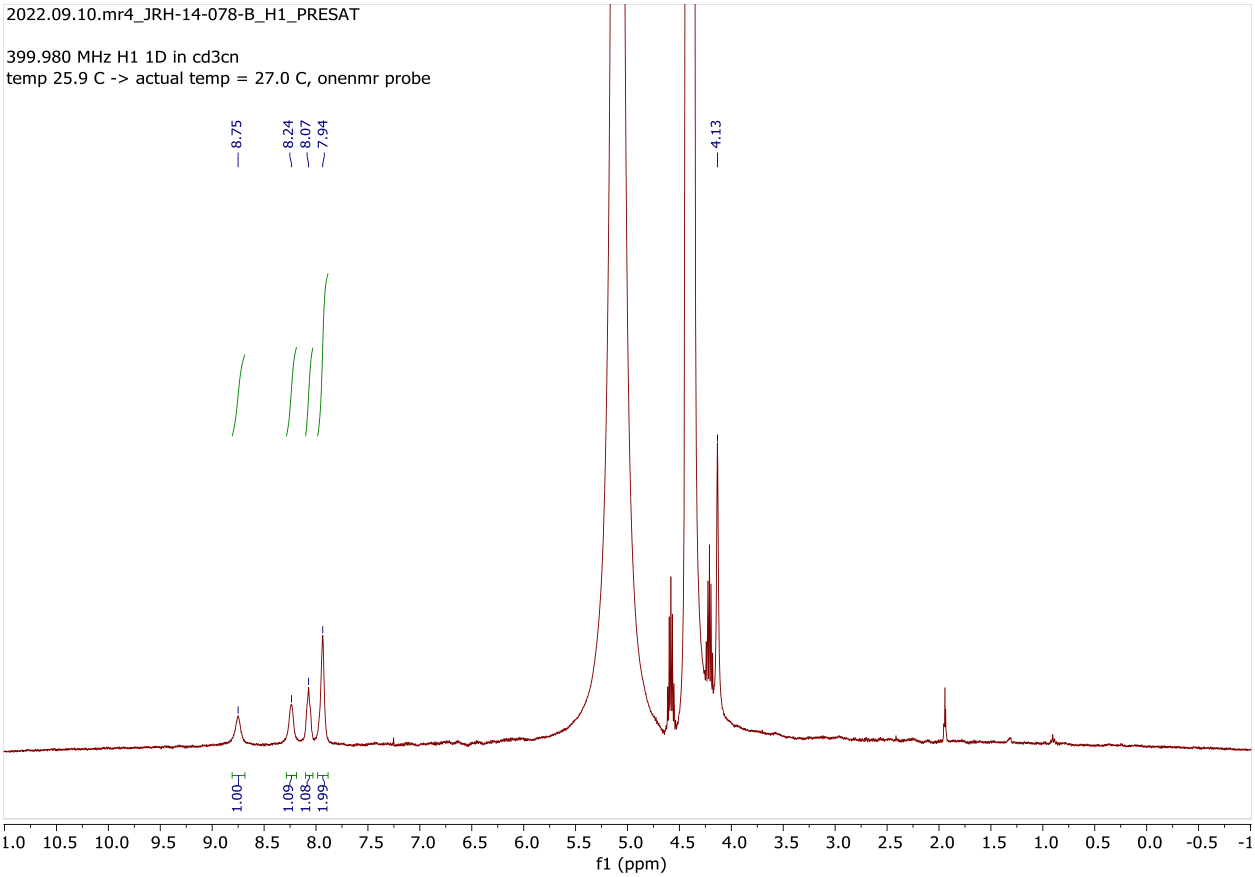

Supplementary Figure 20. ^{1}H NMR of heterocycle **3** in 10:1 HFIP/CD_3CN.

^{11}B NMR showed a single resonance at 26.2 ppm, consistent with a trivalent boron environment.

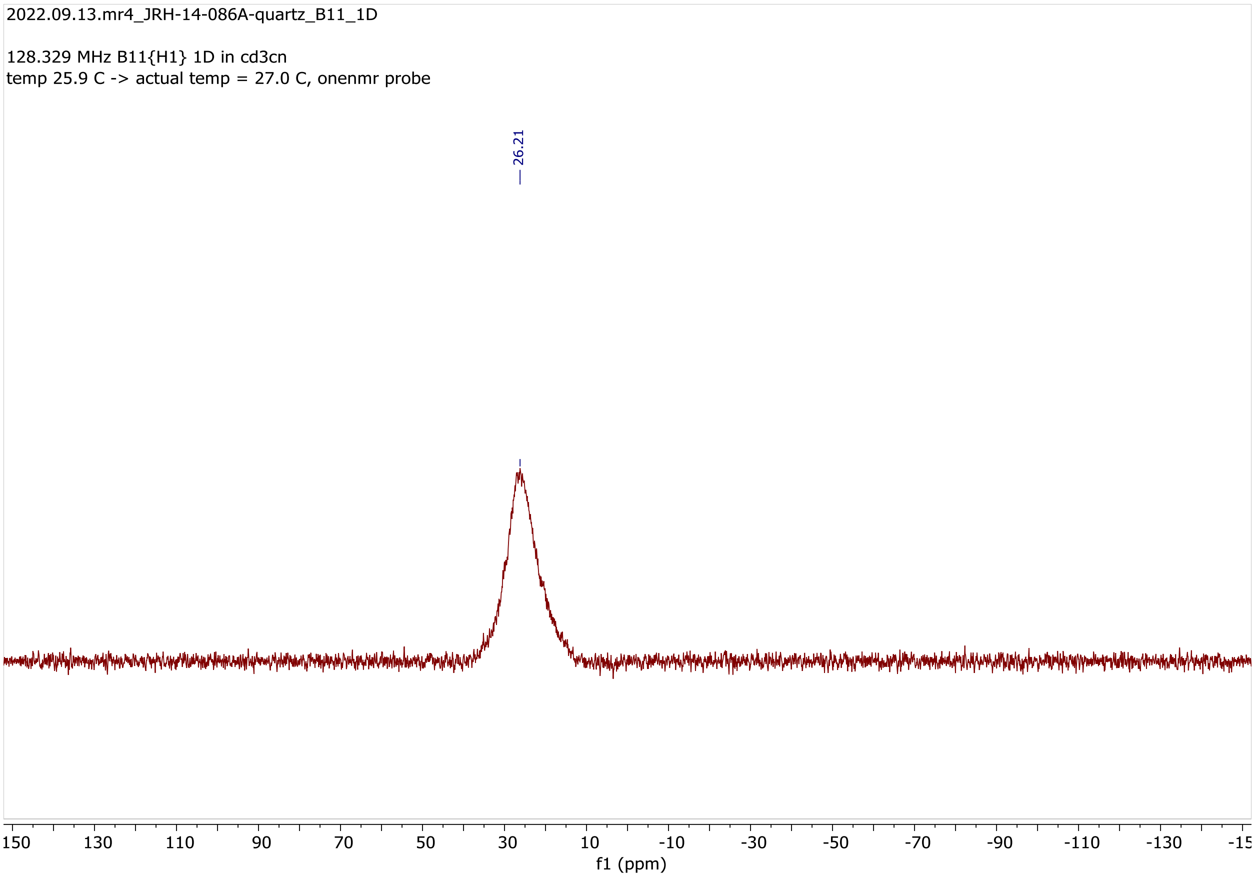

Supplementary Figure 21. ^{11}B NMR of heterocycle **3** in 10:1 HFIP/CD_3CN.

HRMS analysis (ESI, positive mode) was consistent with formation of boranol exchange ester **3 – HFIP**. $C_{11}H_9BF_6NO_2^+$: Calculated: 312.0625; Found: 312.0626.

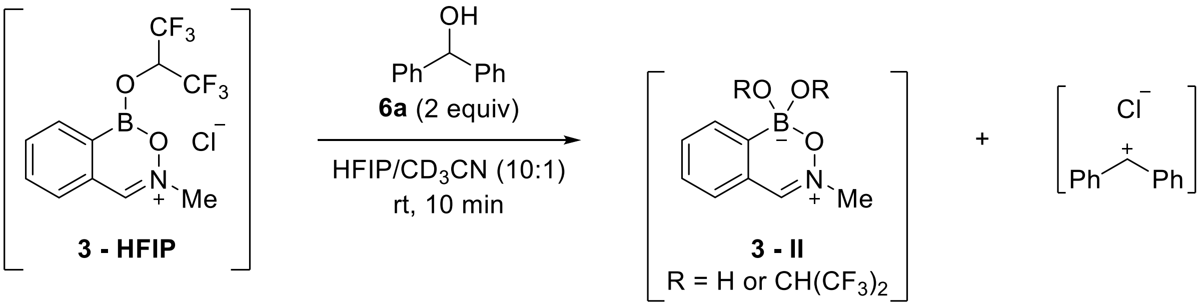

To the previous solution was added alcohol **6a** (9.2 mg, 0.050 mmol, 2.0 equiv). The solution turned pale yellow in color and was subsequently analyzed by ^{11}B NMR. A single resonance was observed at 6.0 ppm, suggesting a tetravalent boron environment that is consistent with formation of zwitterionic boronate **3 - II** which may be formed by C–O ionization of **6a** by reaction with **3** or **3 – HFIP**.

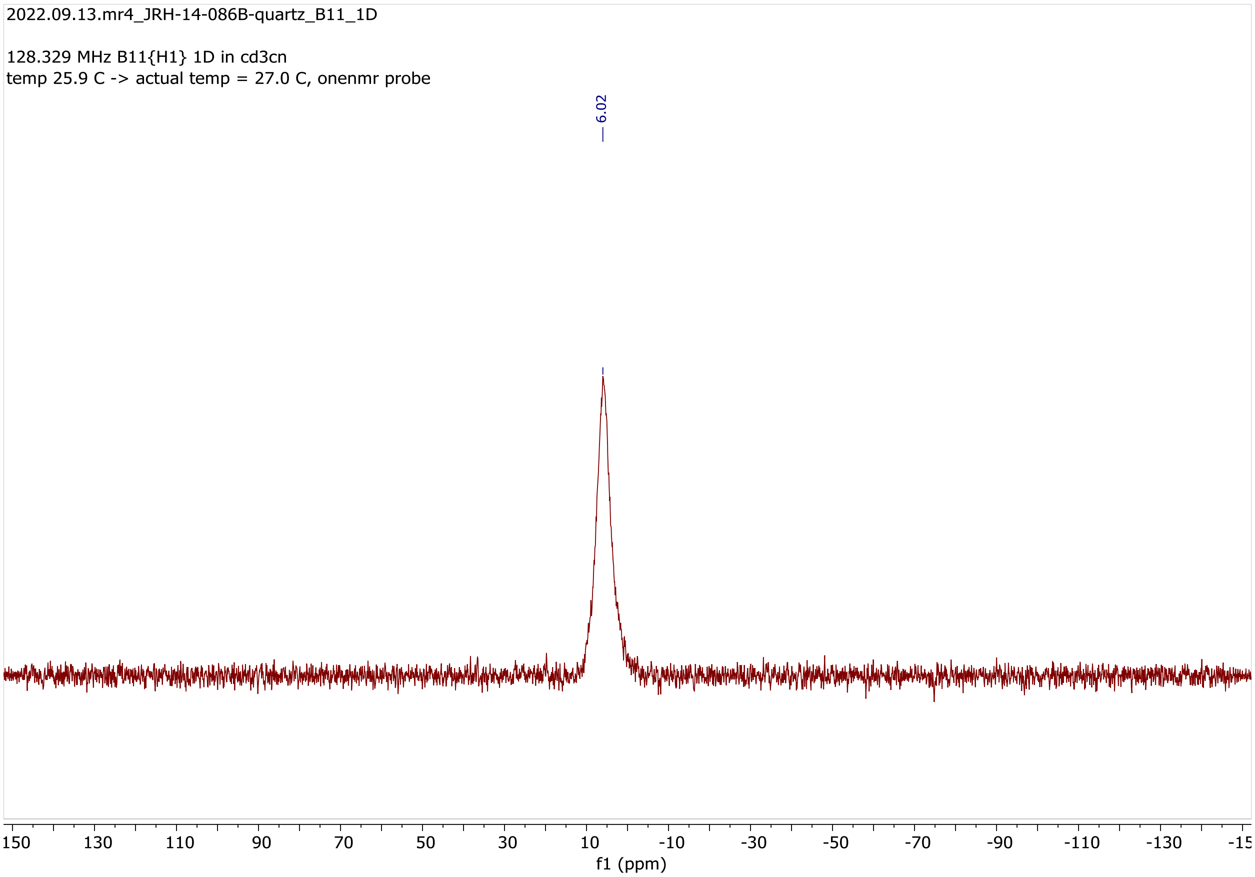

Supplementary Figure 22. ^{11}B NMR of reaction between heterocycle **3** and alcohol **6a** in 10:1 HFIP/CD_3CN.

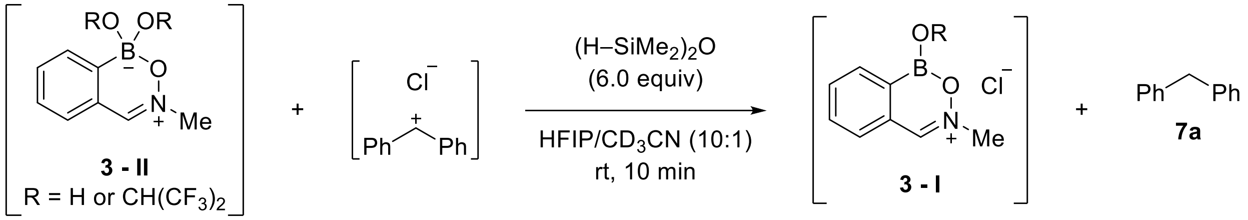

To the above solution was added TMDSO (53 µL, 0.30 mol, 6.0 equiv). The solution was mixed well and analyzed by ^{11}B NMR, where full consumption of the tetravalent boronate was observed to restore a trivalent boron environment with a resonance at 24.9 ppm. Additionally, reduction product **7a** was observed by ^{1}H NMR.

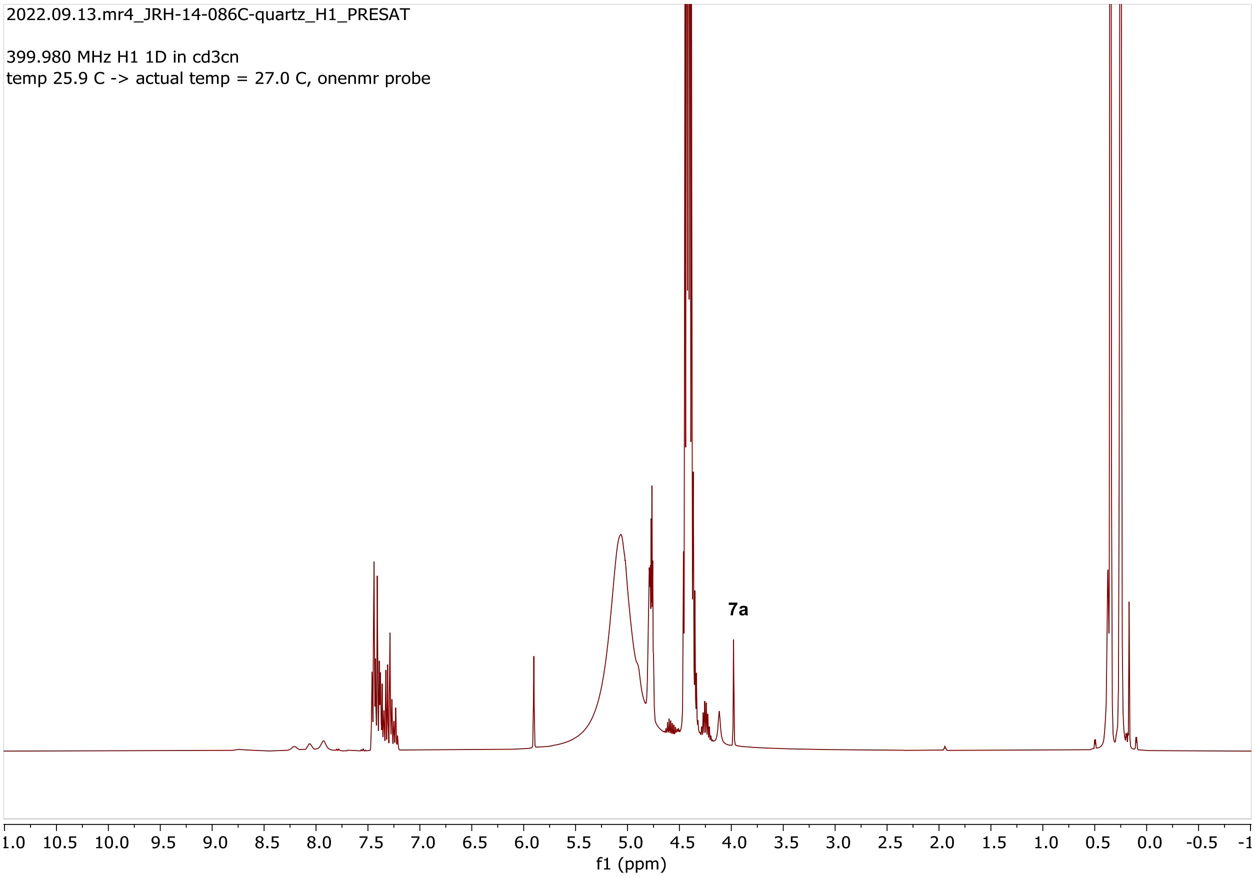

Supplementary Figure 23. ^{1}H NMR after silane addition in 10:1 HFIP/CD_3CN.

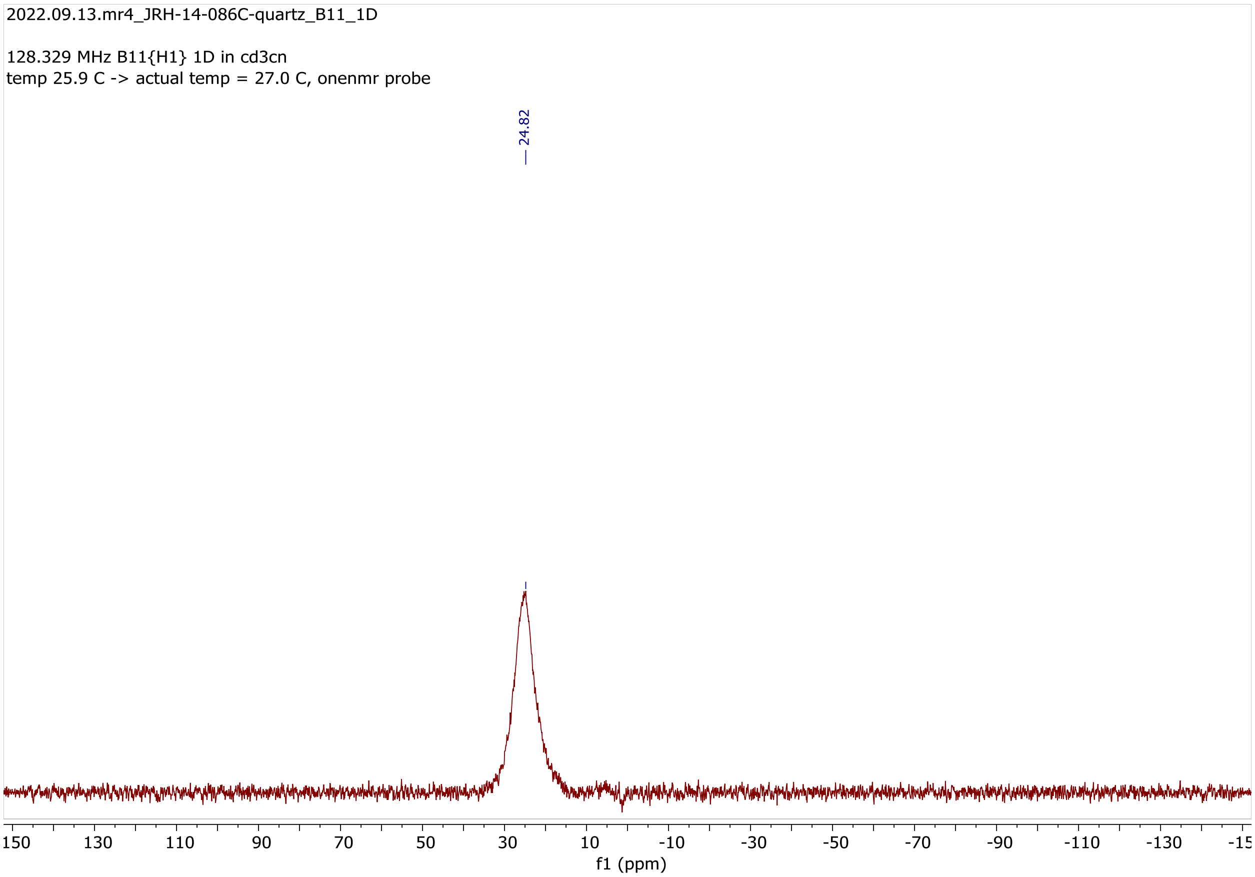

Supplementary Figure 24. ^{11}B NMR after silane addition in 10:1 HFIP/CD_3CN.

Heterocycle **3** was dissolved in HFIP/CD_3CN (10:1) and reacted with two equivalents of unactivated alcohol **S4**. Analysis by ^{11}B NMR showed that no tetravalent boron species were formed based on the lack of an upfield resonance. This contrasts with the reaction with alcohol **6a** described previously and suggests that the upfield ^{11}B resonance observed with the benzylic alcohol (Supplementary Figure 22) arises from C–O ionization rather than covalent exchange alone. Analysis of the ^{1}H NMR showed significant broadening of the methylene protons next to oxygen on **S4**, consistent with a dynamic exchange process.

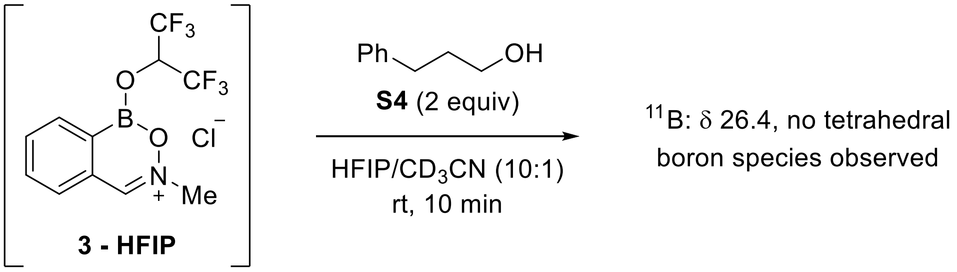

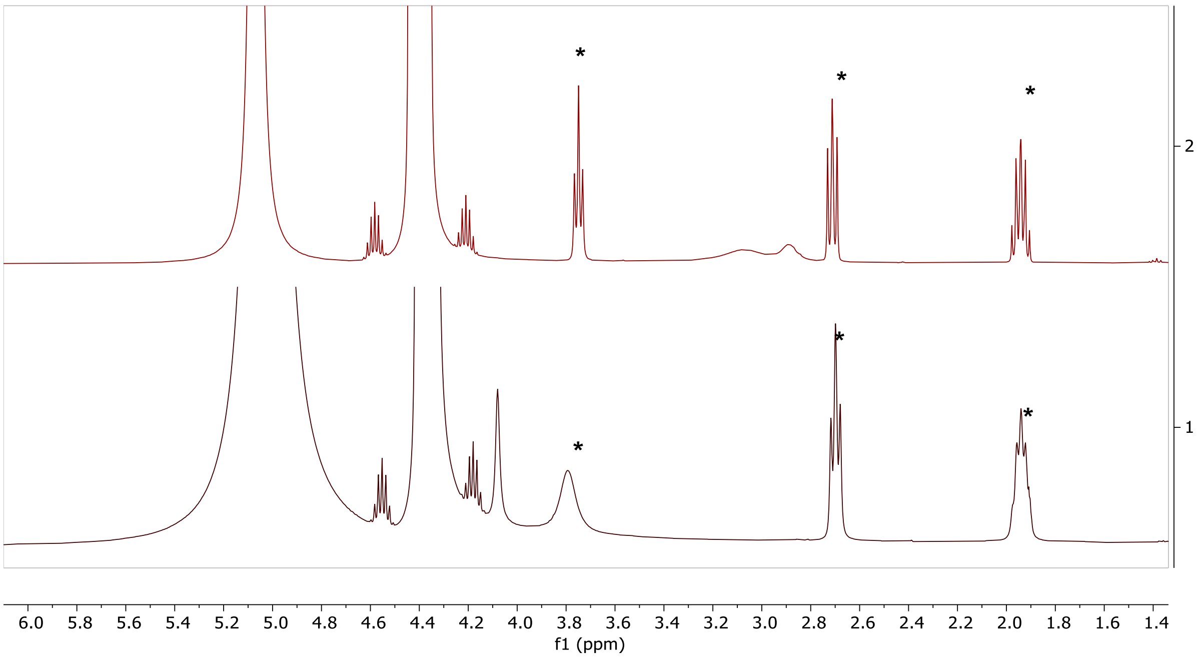

Supplementary Figure 25. ^{1}H NMR of alcohol **S4** in 10:1 HFIP/CD_3CN (top) and upon addition of heterocycle **3** (bottom).

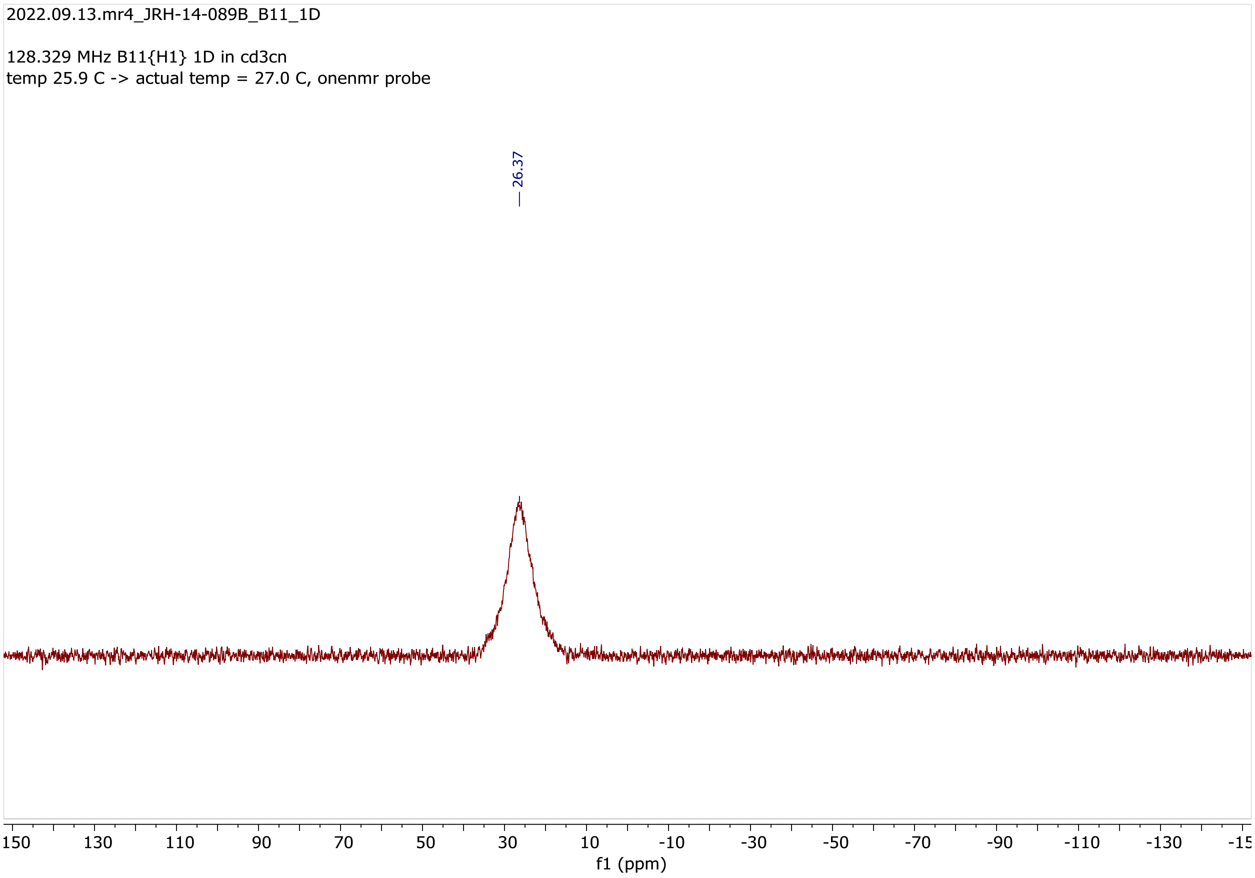

Supplementary Figure 26. ^{11}B NMR for reaction of alcohol **S4** with boron heterocycle **3** in 10:1 HFIP/CD_3CN.

Reaction between heterocycle **3** and TMDSO (5 equiv) in HFIP/CD_3CN (10:1) showed no evidence for borohydride formation by ^{11}B NMR.

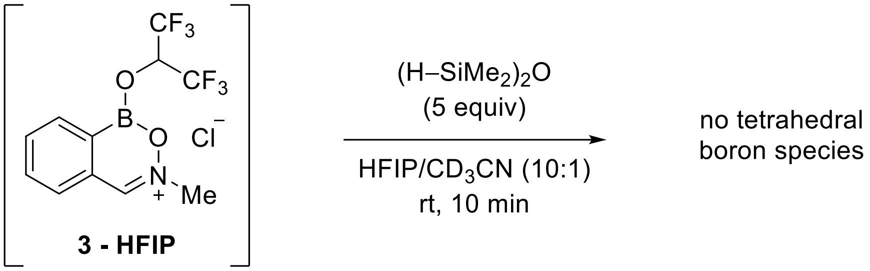

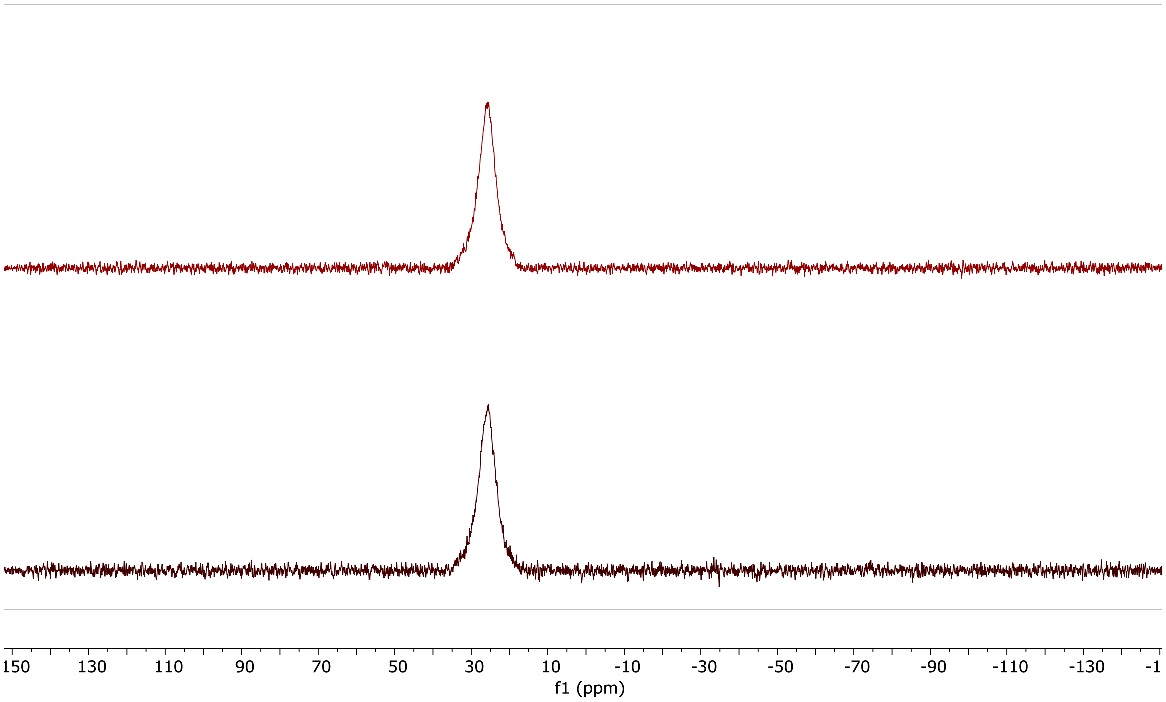

Supplementary Figure 27. ^{11}B NMR analysis (top = ^{1}H decoupled, bottom = not decoupled) of reaction between boron heterocycle **3** and TMDSO in HFIP/CD_3CN (10:1).

Reaction of heterocycle **3** with 2 equivalents of unactivated alcohol **S4** and excess TMDSO also showed no discernable change by ^{11}B NMR, suggesting that substrate-assisted Si–H bond activation to form a borohydride does not occur under these conditions. Taken together, these NMR studies do not provide evidence to support the formation of borohydride intermediates under the conditions for ketone deoxygenation using TMDSO.

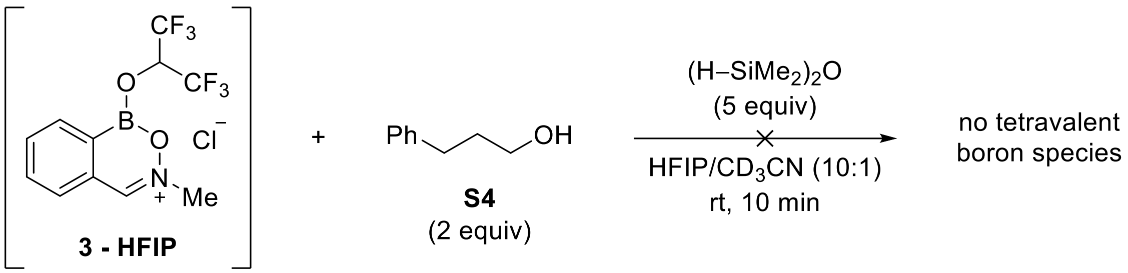

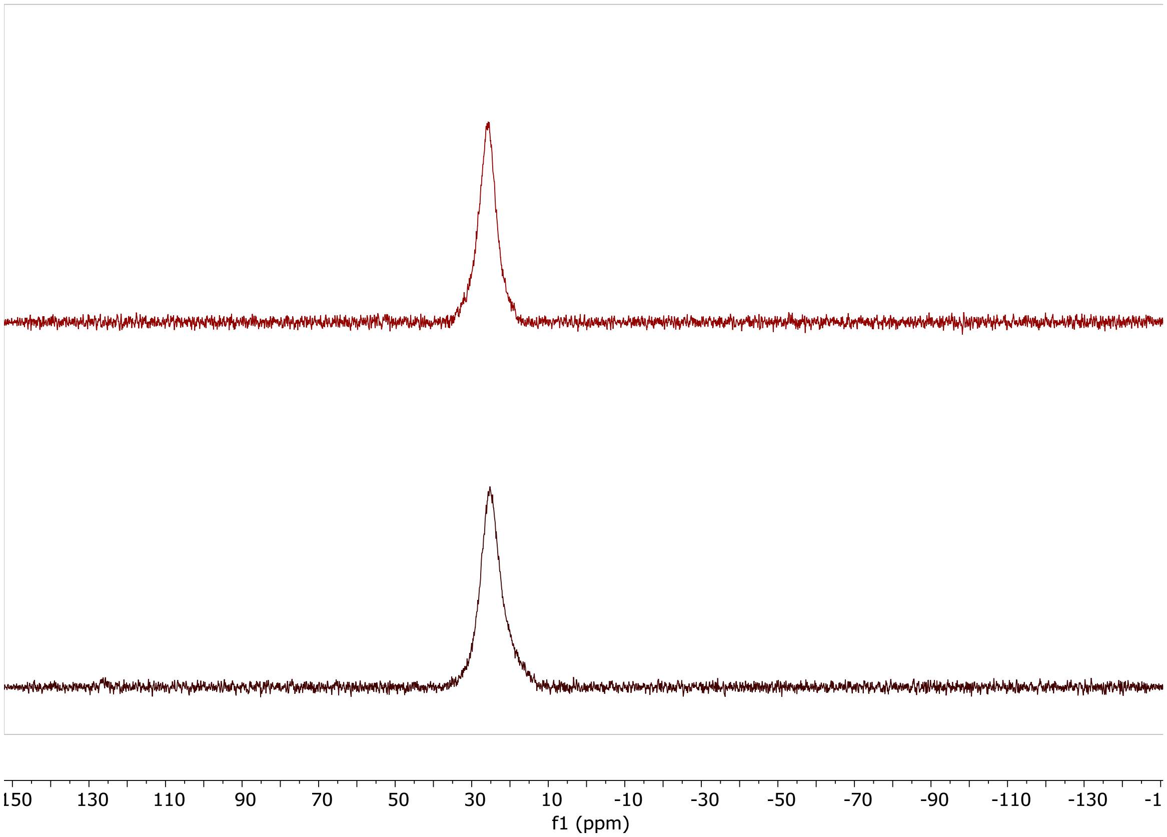

Supplemntary Figure 28. ^{11}B NMR analysis (10:1 HFIP/CD_3CN) of boron heterocycle **3** (top) and of reaction between boron heterocycle **3**, alcohol **S4** and TMDSO (bottom).

10.5 Crystallization of Bis(hexafluoroisopropoxy)boronate Zwitterion 3-II and NMR Study of Boron Speciation

Crystallization was performed as follows: Heterocycle **3** (7.0 mg) was dissolved in a mixture of HFIP (600 µL) and MeCN (60 µL). The mixture was allowed to stand at room temperature for 10 minutes, after which it was concentrated by rotary evaporation (water bath temperature 30 °C). Upon evaporation, white plate shaped crystals were obtained and used for X-ray crystallography, which revealed the zwitterionic structure **3-II**. See Section 13 for full X-ray crystallography details.

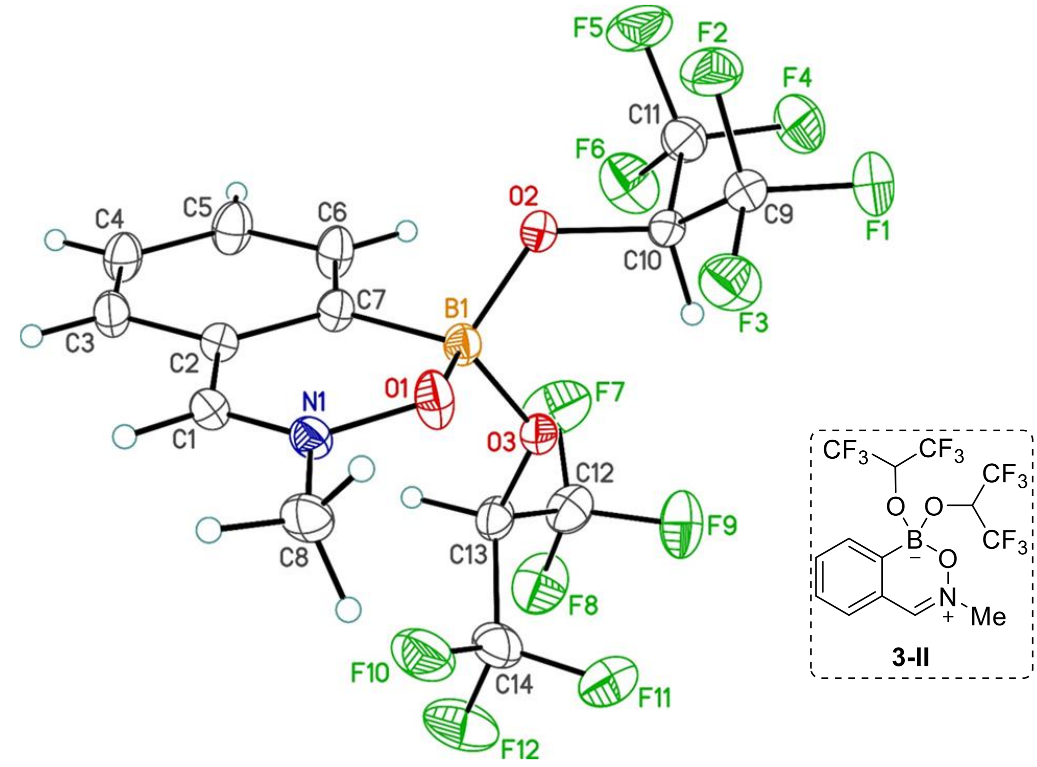

Supplementary Figure 29. X-ray structure of bis(hexafluoroisopropoxy)boronate zwitterion **3-II** obtained from heterocycle **3** after evaporation of an HFIP/MeCN (10:1) solution.

The crystals were subsequently dissolved in CD_3CN, where they were fully soluble (in contrast to starting heterocycle **3**, which is insoluble). Characterization data for zwitterionic boronate **3-II** is provided below.

1,1-Bis[(1,1,1,3,3,3-hexafluoropropan-2-yl)oxy]-3-methyl-1*H*-2,3,1-benzoxazaborinin-1-uide (3-II): Prepared as described above to afford the title compound as a white solid. **mp** = 95.3 – 99.0 °C; **^{1}H NMR** (500 MHz, CD_3CN): δ 8.41 (m, 1 H), 7.72 (td, *J* = 7.4, 1.3 Hz, 1 H), 7.64 (d, *J* = 7.2 Hz, 1 H), 7.59 (d, *J* = 7.7 Hz, 1 H), 7.53 (td, *J* = 7.6, 1.3 Hz, 1 H), 4.65 (heptet, *J* = 6.5 Hz, 2 H), 3.85 (d, *J* = 0.7 Hz, 3 H); **^{13}C NMR** (126 MHz, CD_3CN): δ 147.8, 136.3, 132.6, 130.5, 129.6, 128.3, 123.7 (q, *J* = 283.2 Hz), 70.1 (heptet, *J* = 32.0 Hz), 49.5; **^{11}B NMR** (128 MHz, CD_3CN): δ 4.9; **^{19}F NMR** (376 MHz, CD_3CN): δ – 75.2 (dq, *J* = 9.5, 6.6 Hz, 6 F), – 75.4 (dq, *J* = 8.9, 6.5 Hz, 6 F) (sample contains 2% free HFIP, – 76.4 (d, *J* = 6.5 Hz)); **FTIR** (microscope, cm^{-1}): 2956 (w), 2911 (w), 1375 (m), 1233 (s), 1187 (s), 1149 (m), 1105 (m), 1007 (w), 894 (w), 672 (w); **HRMS** (ESI) for $C_{14}H_{10}{}^{11}BF_{12}NO_3Na$ $[M+Na]^+$: Calculated: 502.0454; Found: 502.0450.

As described in Section 10.4, solution NMR of heterocycle **3** dissolved in a mixture of HFIP/CD_3CN (10:1) showed an ^{11}B NMR resonance at 26.2 ppm, consistent with a trivalent boron compound. This contrasts with the X-ray structure shown above for zwitterion **3-II** which contains a tetravalent boron. It is noteworthy that the sample was concentrated by rotary evaporation prior to crystallographic analysis. Accordingly, supplementary experiments were performed to elucidate the effect of rotary evaporation on boron speciation and to probe whether zwitterion **3-II** was a viable catalyst or pre-catalyst in deoxygenation.

Boron heterocycle **3** (5.0 mg) was dissolved in HFIP (600 µL) and CD_3CN (60 µL). The solution was analyzed directly by ^{11}B NMR in a quartz tube, where a single broad resonance was observed at 26.2 ppm.

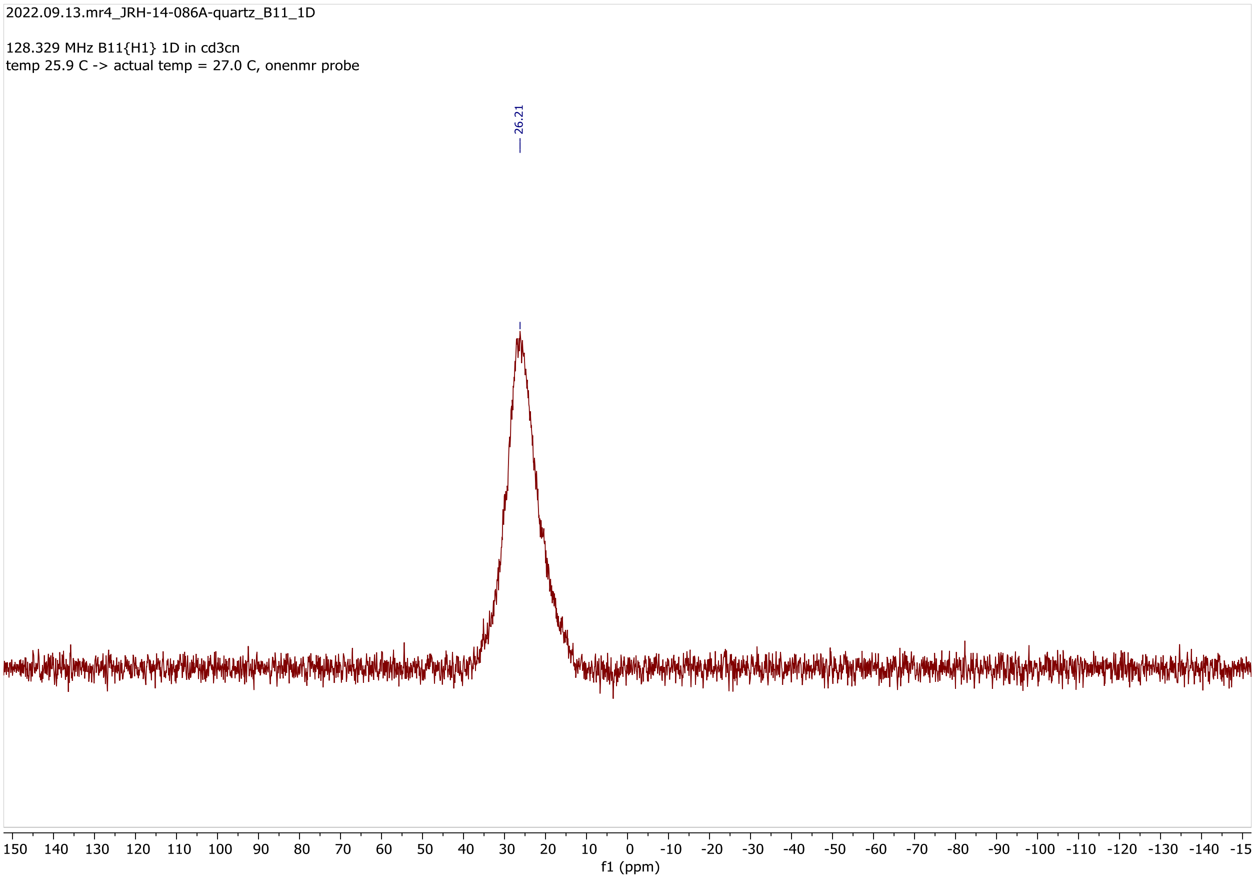

Supplementary Figure 30. ^{11}B NMR of heterocycle **3** in HFIP/CD_3CN (10:1).

Boron heterocycle **3** (5.0 mg) was dissolved in HFIP (600 µL) and CD_3CN (60 µL). The mixture was allowed to stand for 10 minutes at room temperature, after which it was concentrated by rotary evaporation. The resulting solid was dissolved in HFIP (600 µL) and CD_3CN (60 µL) and analyzed by ^{11}B NMR in a quartz tube, where a single resonance was observed at 5.9 ppm. This is consistent with the tetravalent boron compound characterized under these conditions by X-ray crystallography.

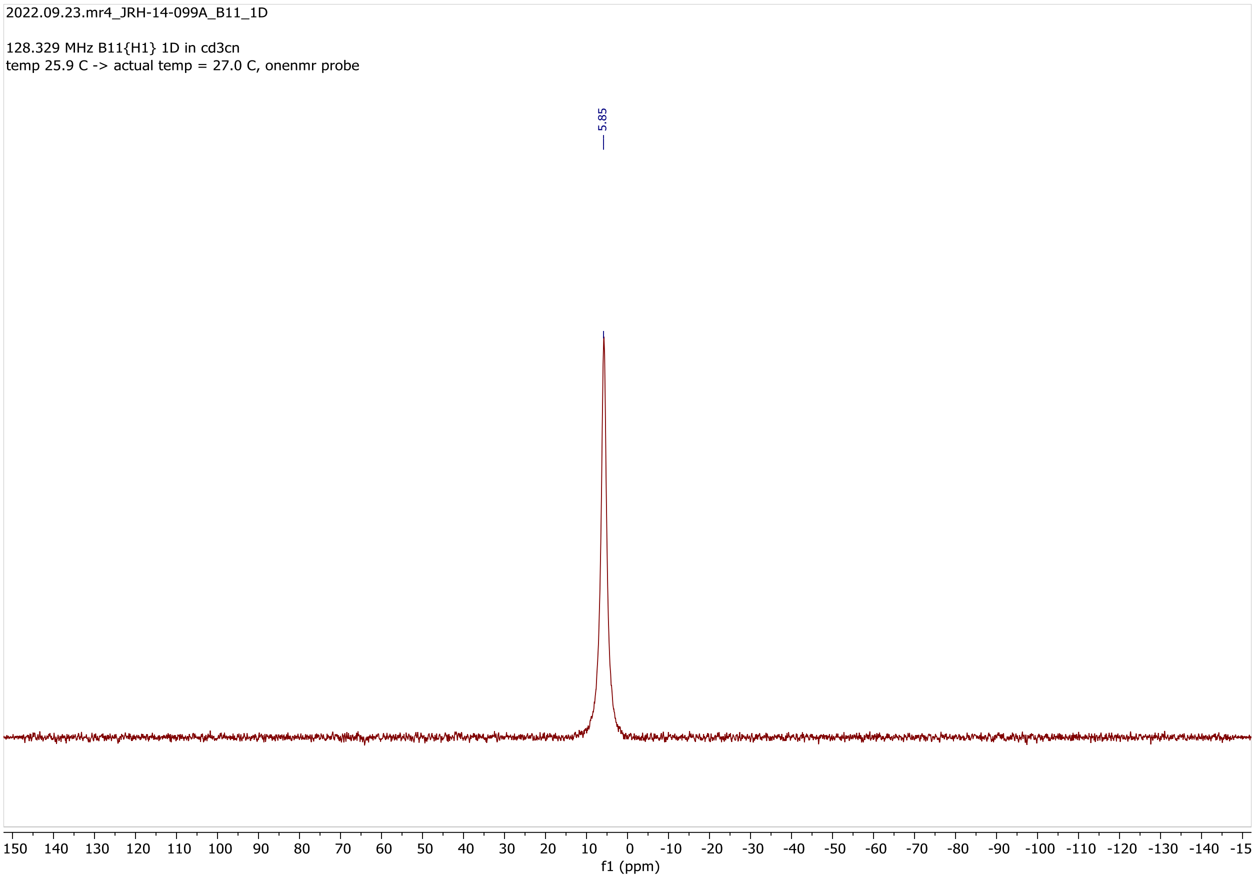

Supplementary Figure 31. ^{11}B NMR after heterocycle **3** was dissolved in HFIP/CD_3CN (10:1), concentrated by rotary evaporation, and re-dissolved in HFIP/CD_3CN (10:1) for NMR analysis.

Boron heterocycle **3** (5.0 mg) was dissolved in HFIP (600 µL). The mixture was allowed to stand for 10 minutes at room temperature, after which it was concentrated by rotary evaporation. The resulting solid was dissolved in HFIP (600 µL) and CD_3CN (60 µL) and analyzed by ^{11}B NMR in a quartz tube, where two resonances were observed at 27.8 ppm (15.2%) and 6.5 ppm (84.8%). This observation suggests that a mixture of both trivalent and tetravalent boron compounds was present under these conditions.

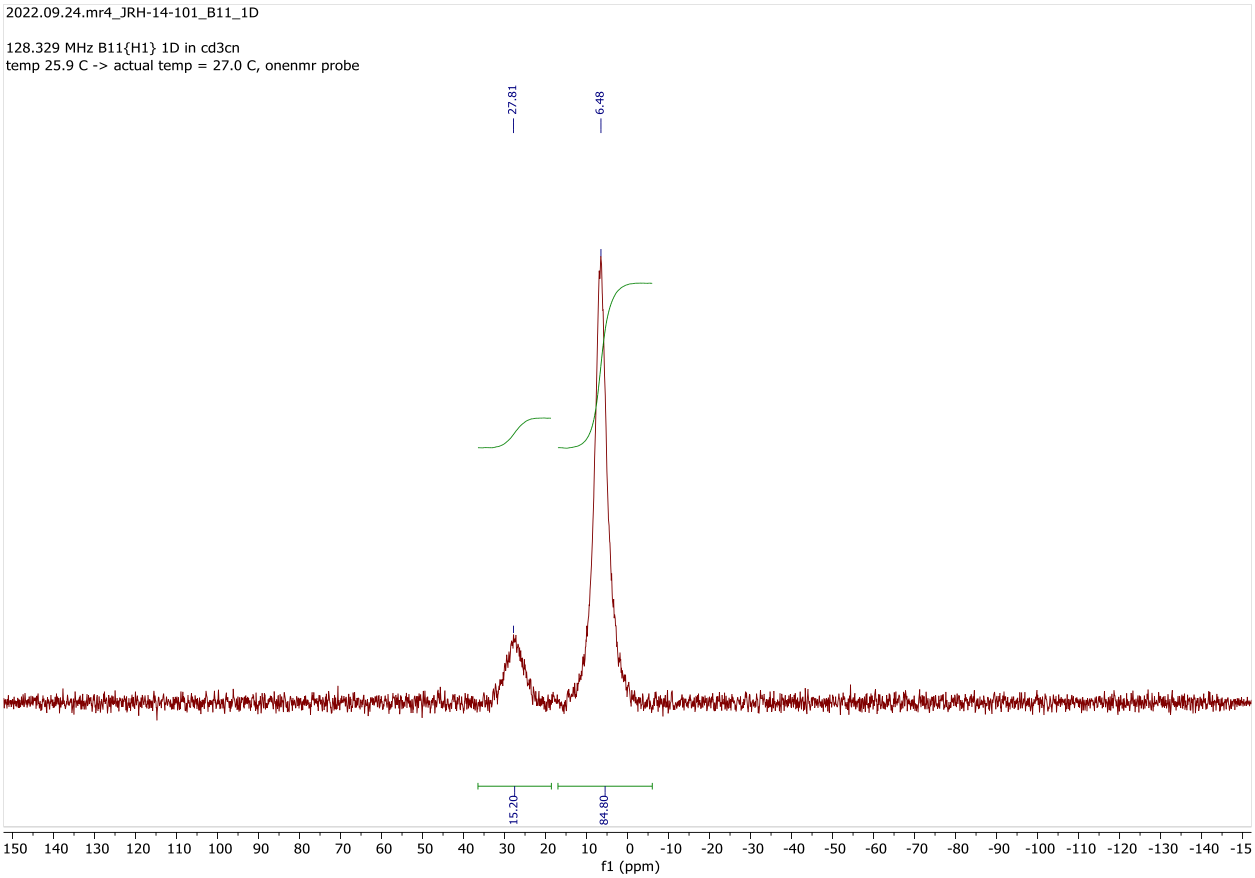

Supplementary Figure 32. ^{11}B NMR after heterocycle **3** was dissolved in HFIP, concentrated by rotary evaporation, and re-dissolved in HFIP/CD_3CN (10:1) for NMR analysis.

Boron heterocycle **3** (5.0 mg) was suspended in CD_3CN (600 μL). The resulting heterogeneous mixture was allowed to stand for 10 minutes at room temperature, after which it was concentrated by rotary evaporation. The resulting solid was dissolved in HFIP (600 μL) and CD_3CN (60 μL) and analyzed by ^{11}B NMR in a quartz tube, where a broad resonance was observed at 27.8 ppm. This observation suggests that no tetravalent boron compounds were formed under these conditions.

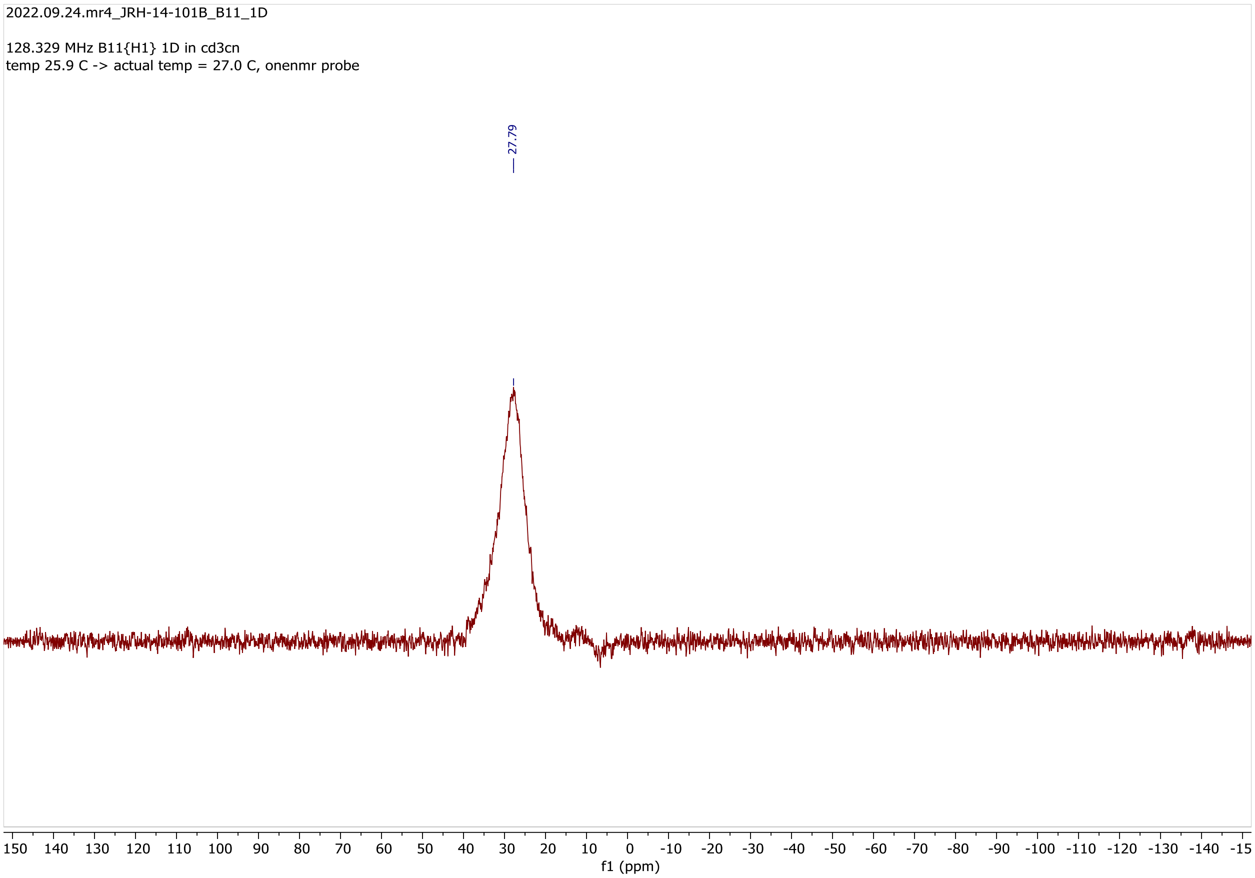

Supplementary Figure 33. ^{11}B NMR after heterocycle **3** was suspended in CD_3CN, concentrated by rotary evaporation, and re-dissolved in HFIP/CD_3CN (10:1) for NMR analysis.

A stack plot of these ^{11}B NMR experiments is shown below.

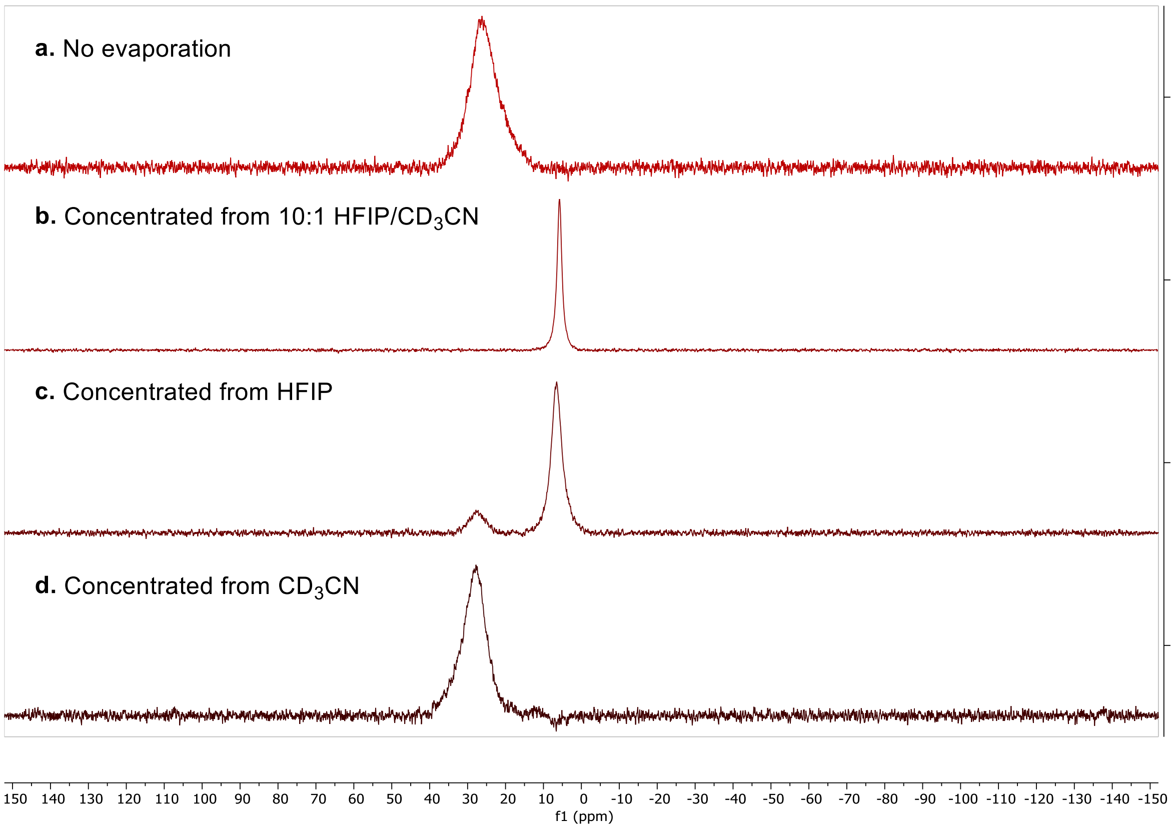

Supplementary Figure 34. Stack plot of ^{11}B NMR experiments to study the effect of prior rotary evaporation in different solvents on the speciation of heterocycle **3** in HFIP/CD_3CN (10:1).

HFIP (boiling point 59 °C) is more volatile than CD_3CN (boiling point 81 °C). Accordingly, when a solution containing a mixture of HFIP/CD_3CN is concentrated by rotary evaporation, the fraction of CD_3CN should increase over time. As the polarity of the solvent mixture decreases with the loss of HFIP, we believe that zwitterion **3-II** may be more effectively solubilized due to internal charge neutralization, whereas solvent-separated ion pair **3-HFIP** may be ineffectively stabilized by CD_3CN.

A solution of heterocycle **3** in HFIP with no deuterated solvent was analyzed by ^{11}B NMR, where a broad resonance was observed with the major signal at 28.1 ppm and a shoulder at 19.6 ppm. It is difficult to ascertain the exact nature of the mixture that is present, but the lack of upfield resonances (5–8 ppm) suggests that the boron atom is largely three-coordinate in nature in line with the preliminary mechanistic proposal for alcohol deoxygenation in Figure 9.

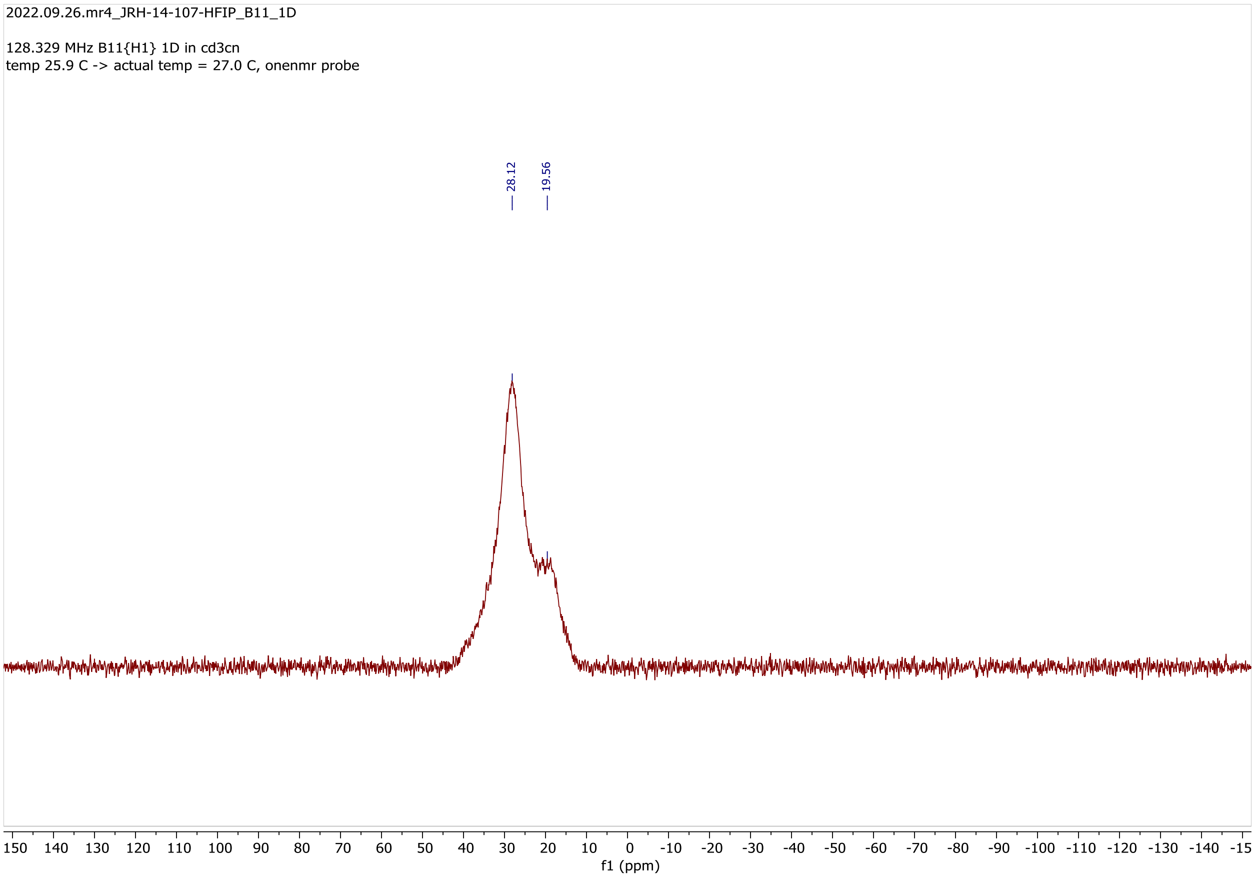

Supplementary Figure 35. ^{11}B NMR of heterocycle **3** in HFIP.

Additionally, zwitterion **3-II** was used as a catalyst in the reductive deoxygenation of model alcohol and ketone substrates **6u** and **8a** respectively. In both cases, nearly quantitative recovery of the starting material was observed. This further supports the proposal that a trivalent boron compound such as **3-HFIP** is the active catalyst in solution rather than a tetravalent species. Furthermore, it suggests that tetravalent **3-II** and trivalent **3-HFIP** do not appreciably interconvert in solution in the absence of a proposed carbocation formed upon C–O bond activation of the substrate.

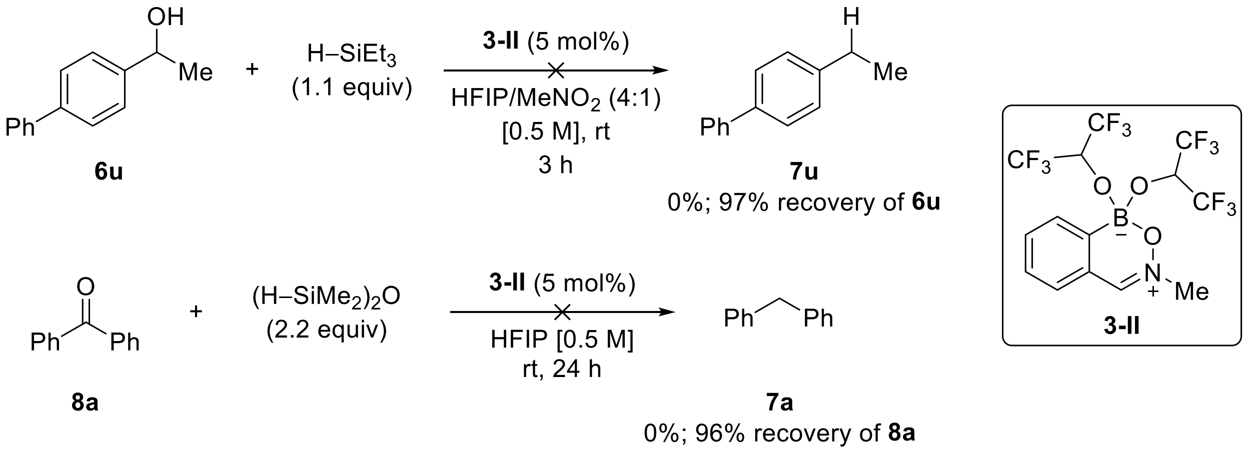

Supplementary Notes

11. NMR Spectra

^{1}H (500 MHz), ^{13}C (126 MHz) and ^{11}B NMR (128 MHz) spectra of compound 3 (D_2O)

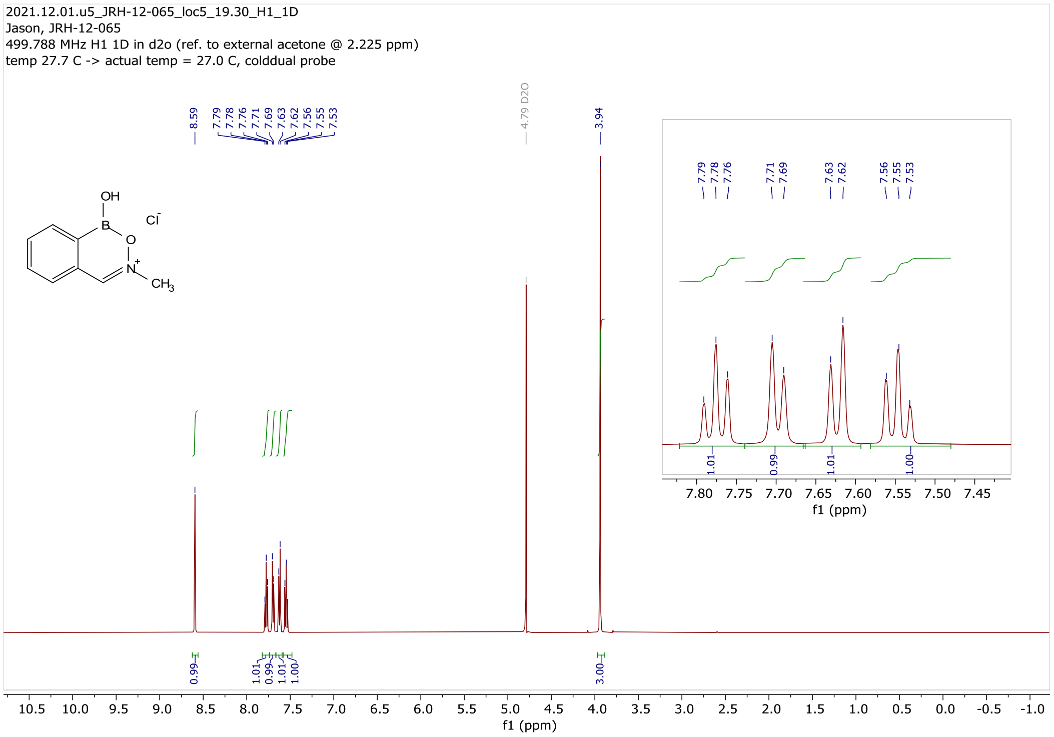

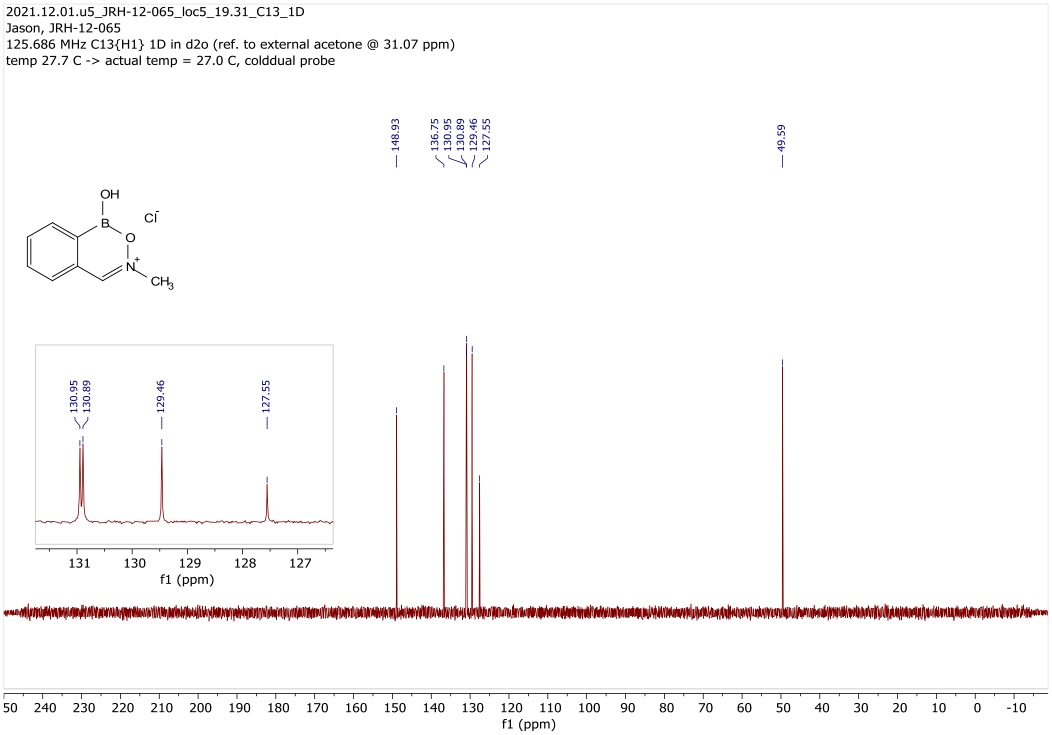

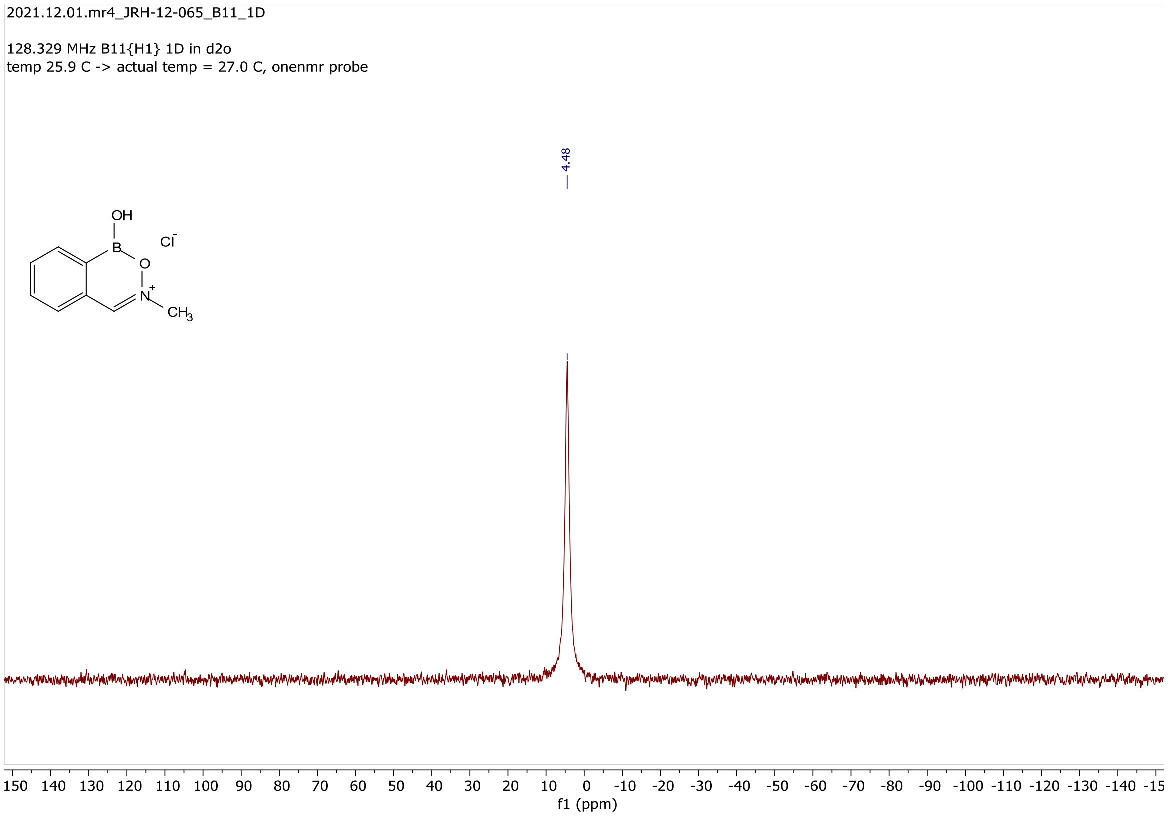

^{1}H (500 MHz) and ^{13}C (126 MHz) NMR of compound 4b ($CDCl_3$)

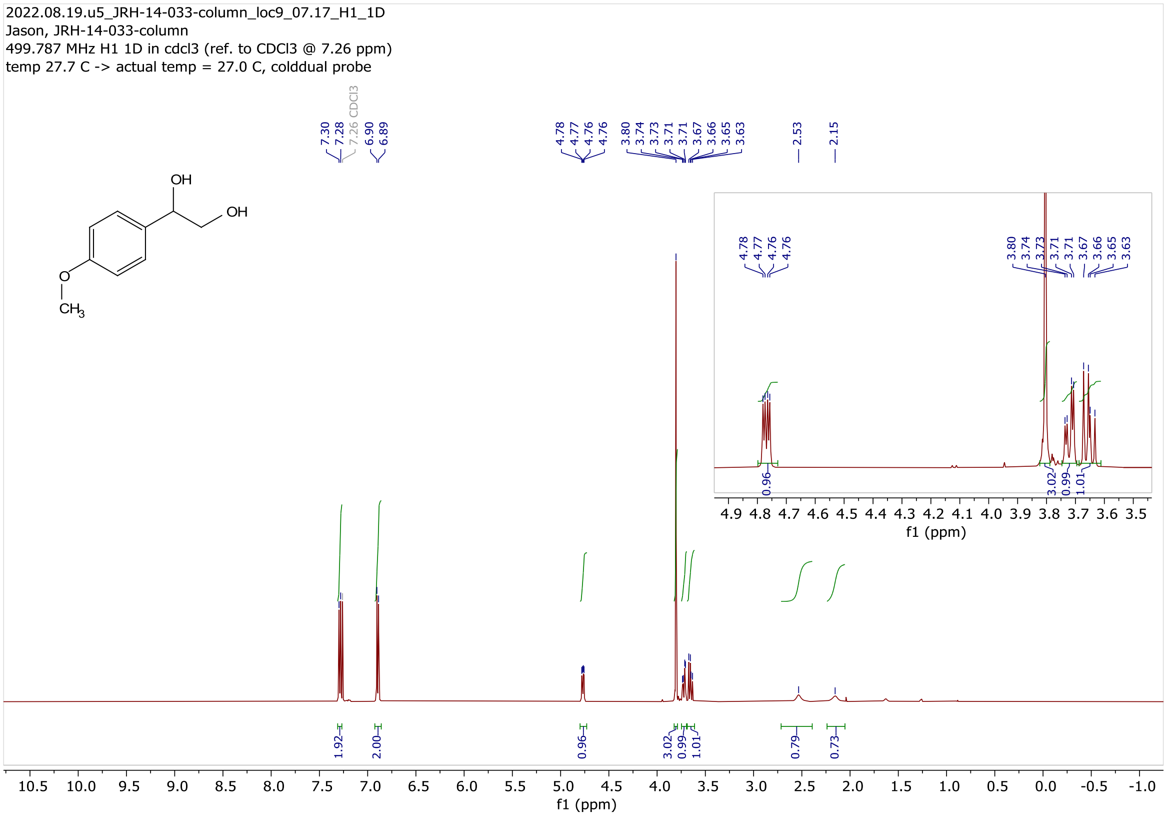

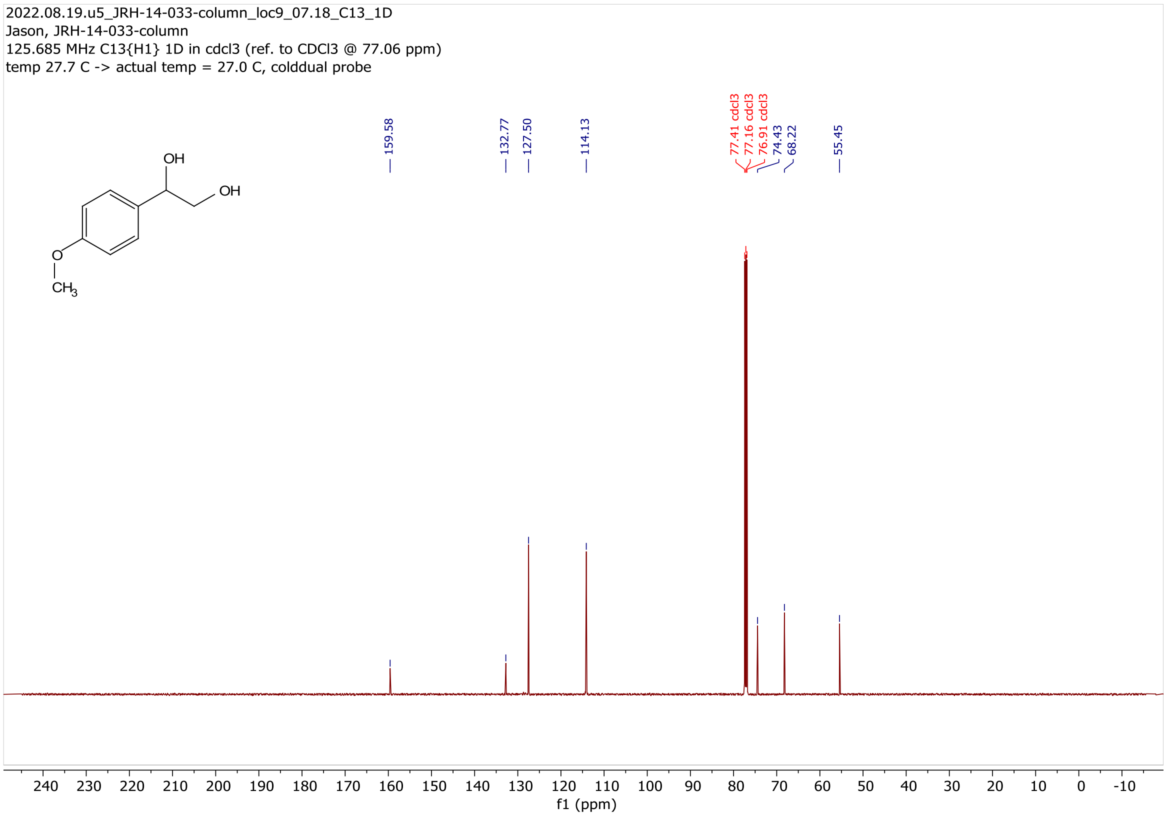

^{1}H (500 MHz), ^{13}C (126 MHz) and ^{19}F (376 MHz) NMR of compound 4c (CDCl$_3$)

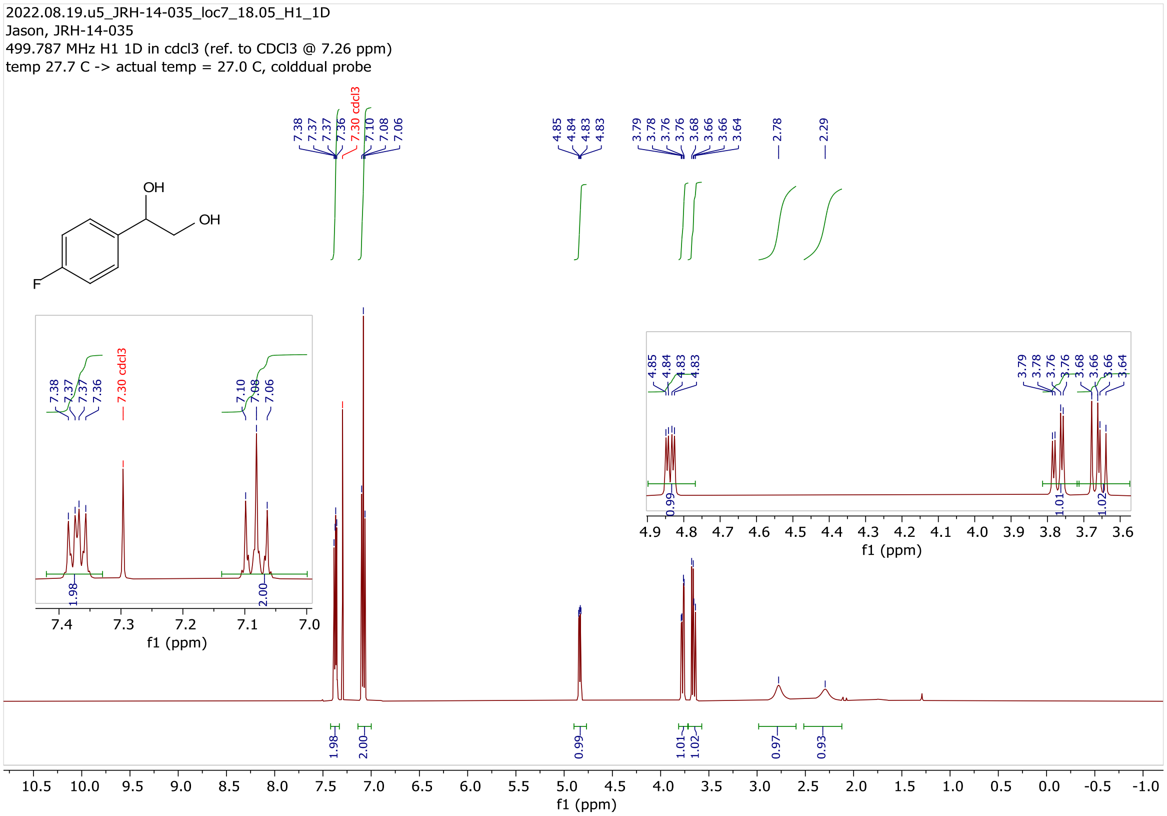

2022.08.19.u5_JRH-14-035_loc7_18.06_C13_1D
Jason, JRH-14-035
125.685 MHz C13{H1} 1D in cdcl3 (ref. to CDCl3 @ 77.06 ppm)
temp 27.7 C -> actual temp = 27.0 C, colddual probe

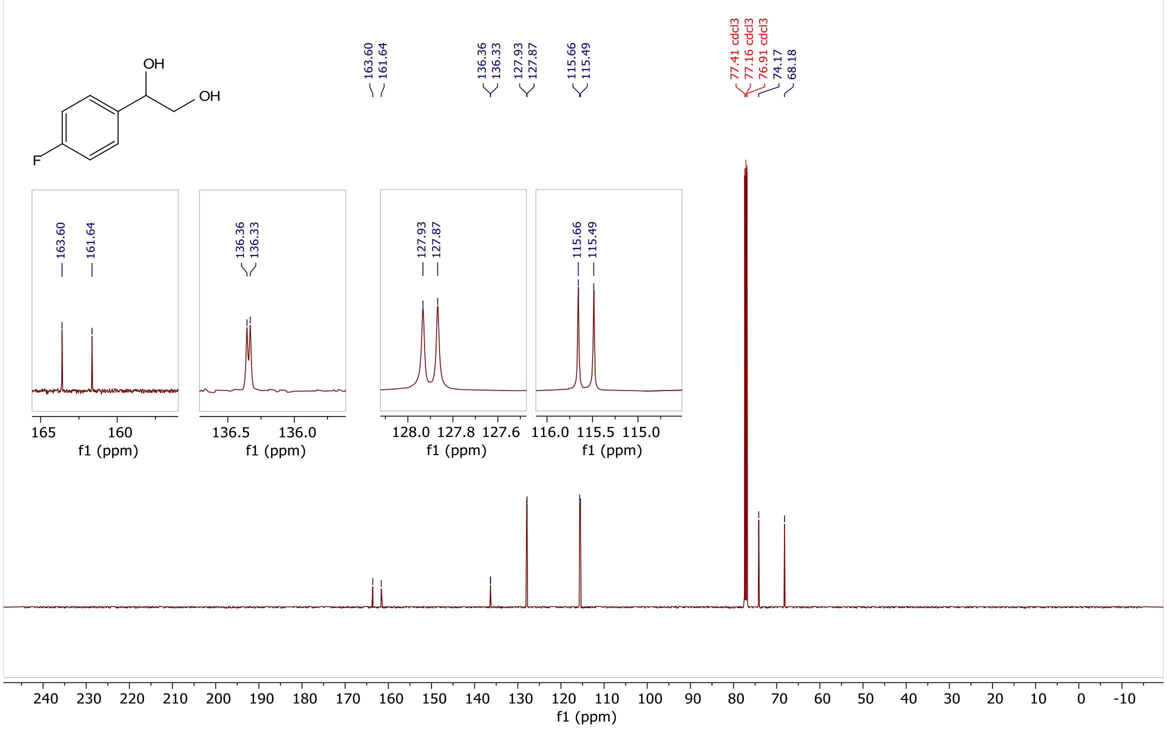

2022.08.19.mr4_JRH-14-035_F19_1D

376.306 MHz F19 1D in cdcl3
temp 25.9 C -> actual temp = 27.0 C, onenmr probe

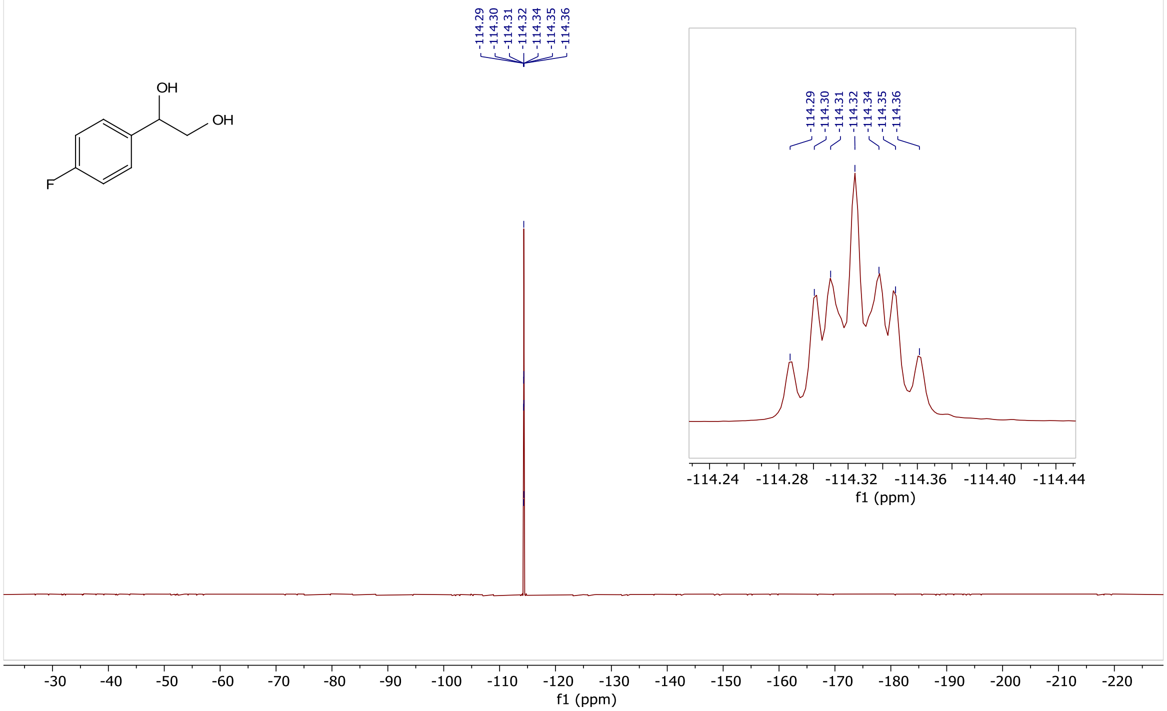

^{1}H (500 MHz) and ^{13}C (126 MHz) NMR of compound 4d ($CDCl_3$)

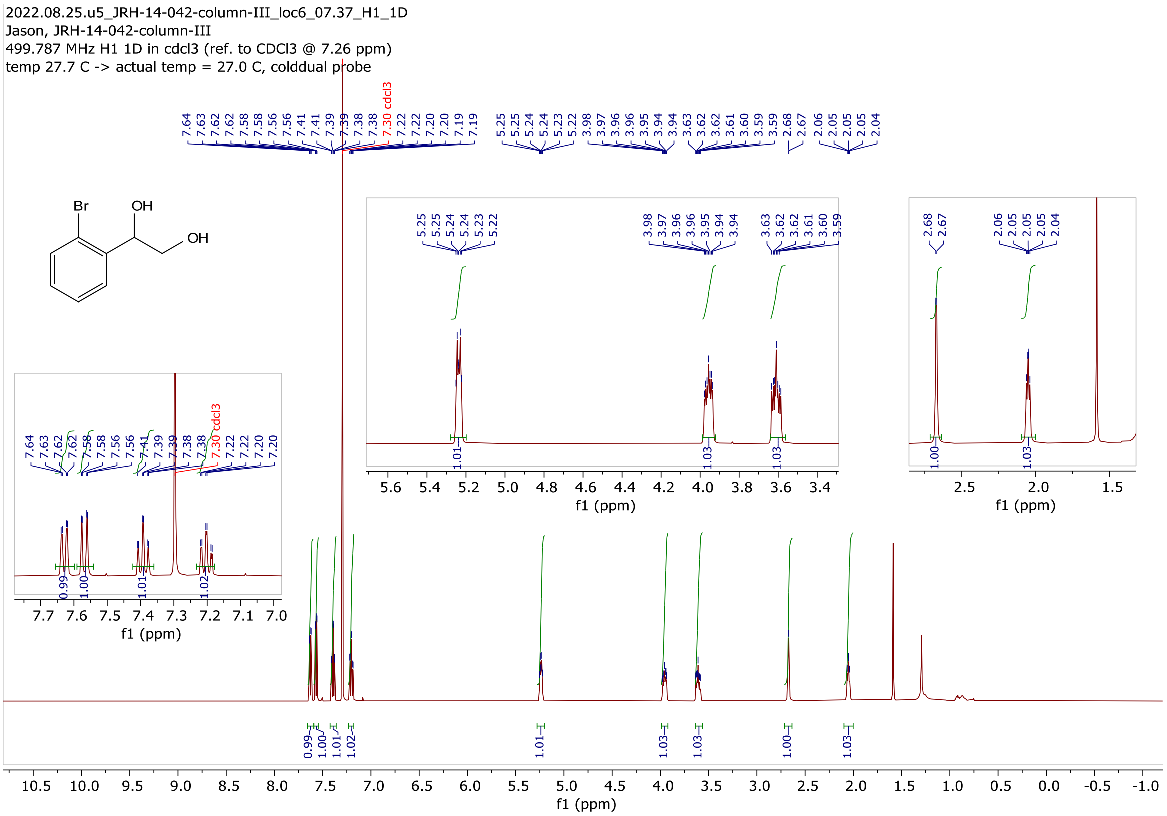

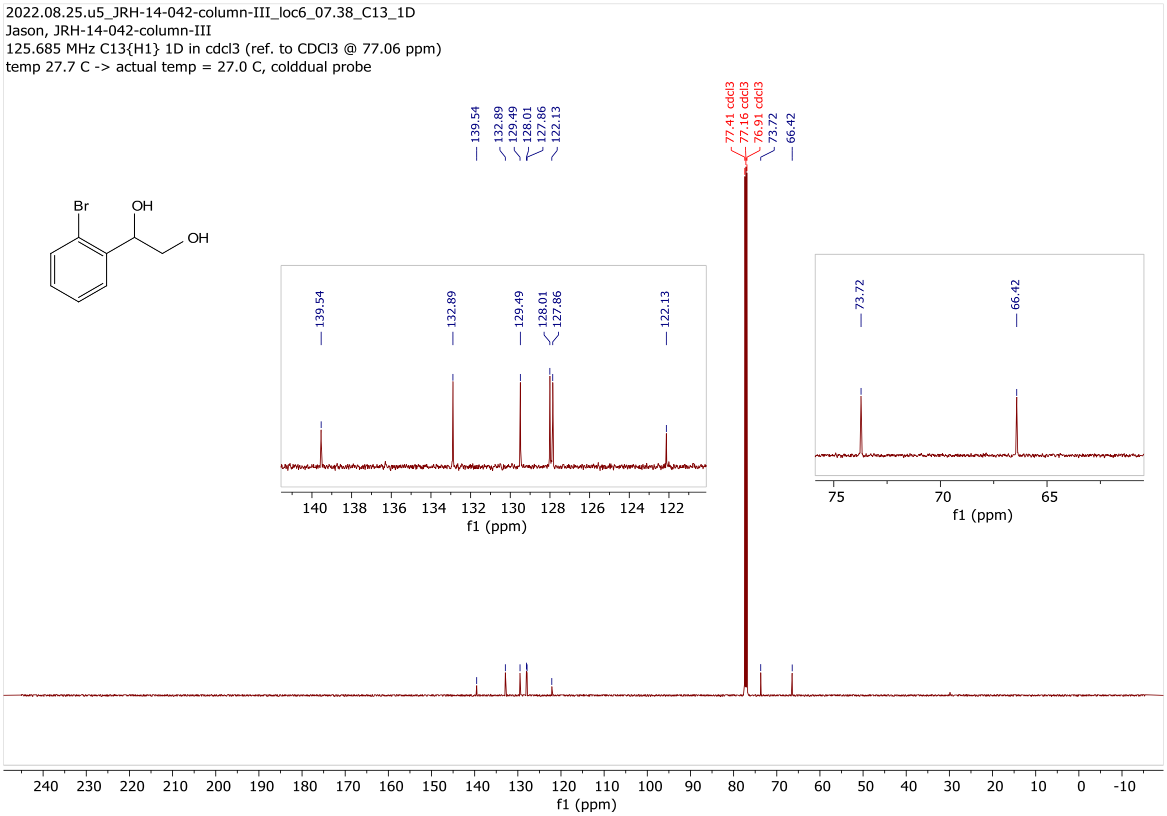

^{1}H (500 MHz) and ^{13}C (126 MHz) NMR of compound 4e ($CDCl_3$)

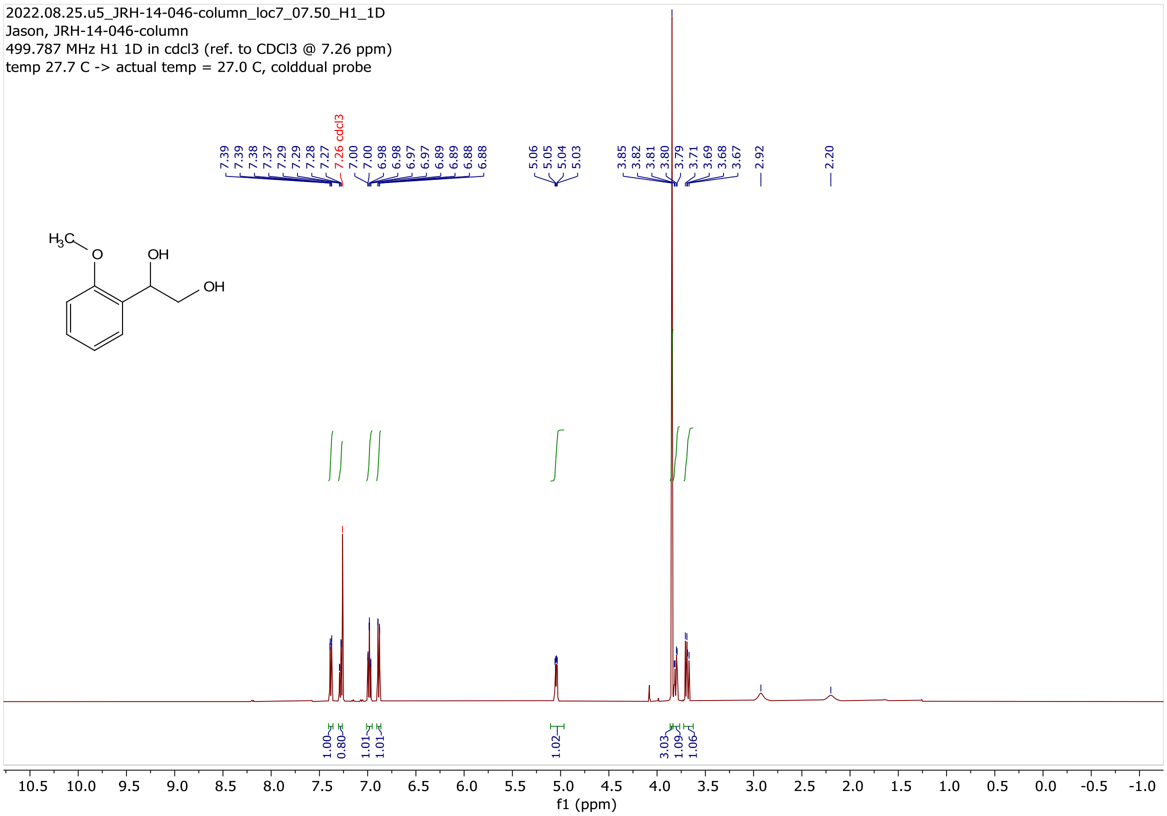

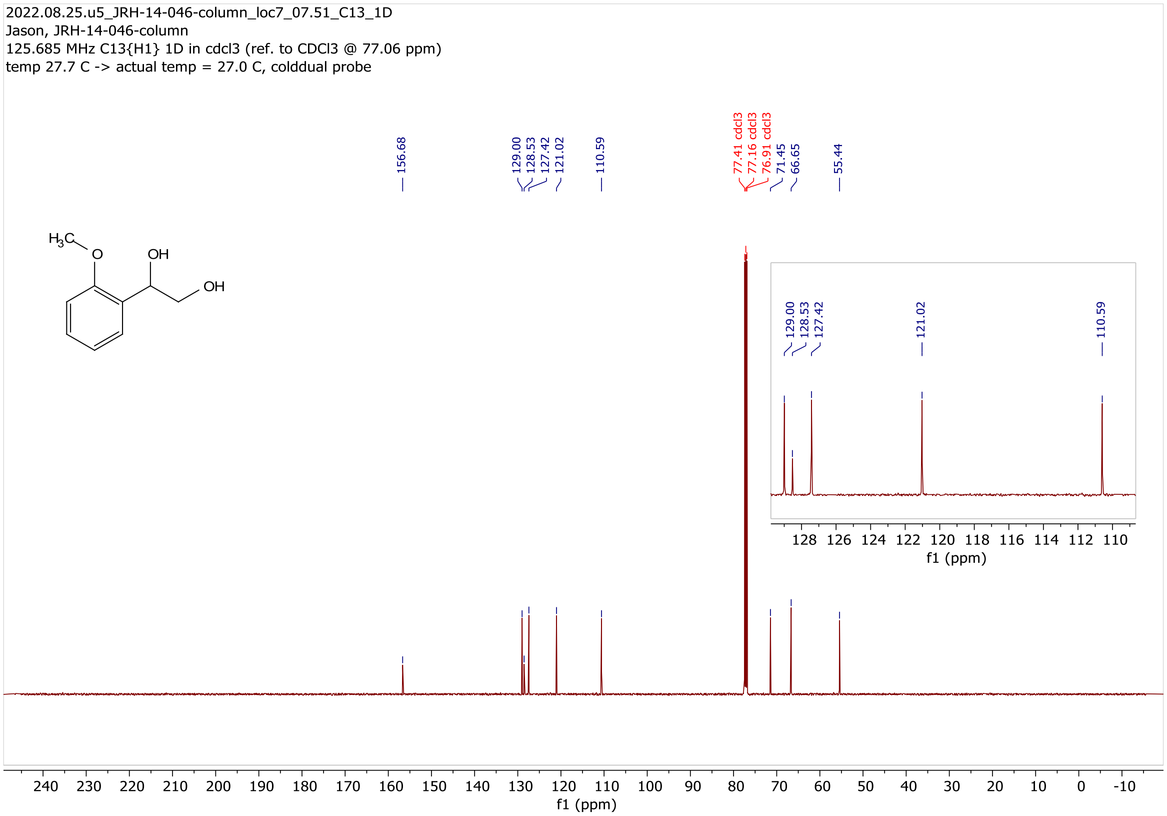

^{1}H (500 MHz) and ^{13}C (126 MHz) NMR of compound 4g ($CDCl_3$)

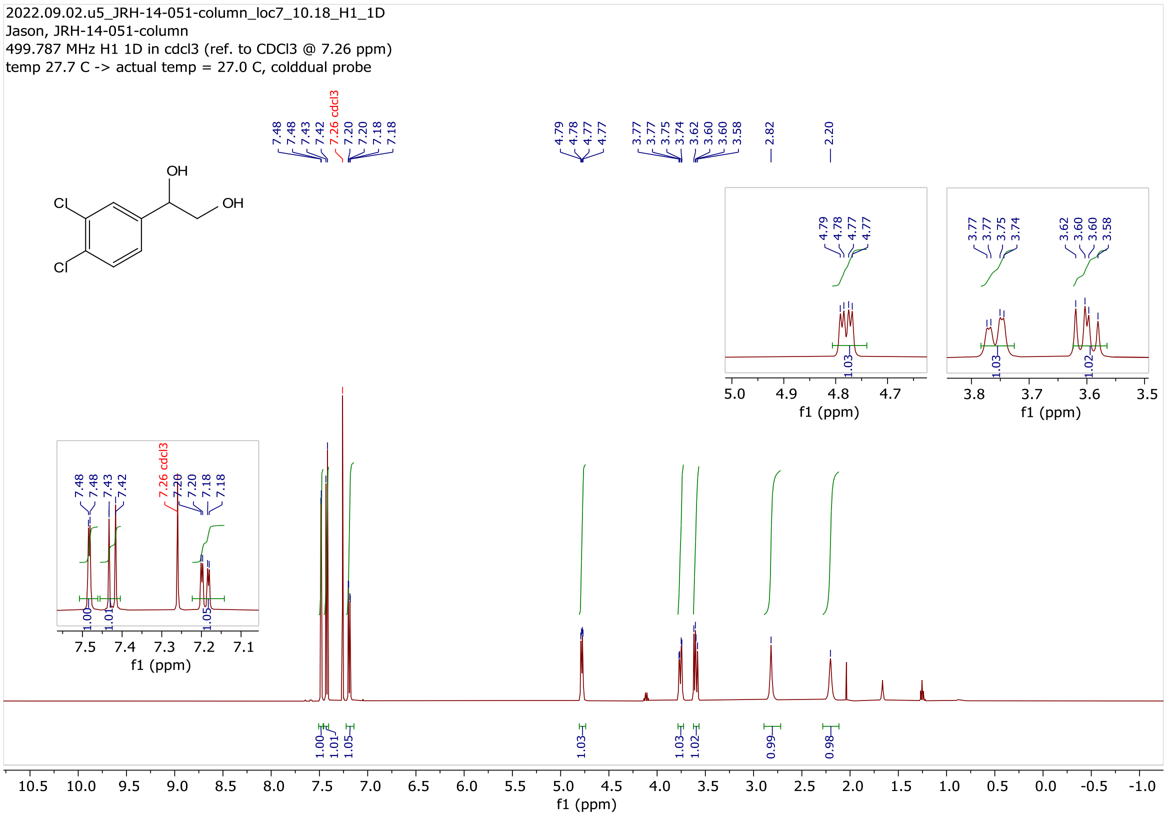

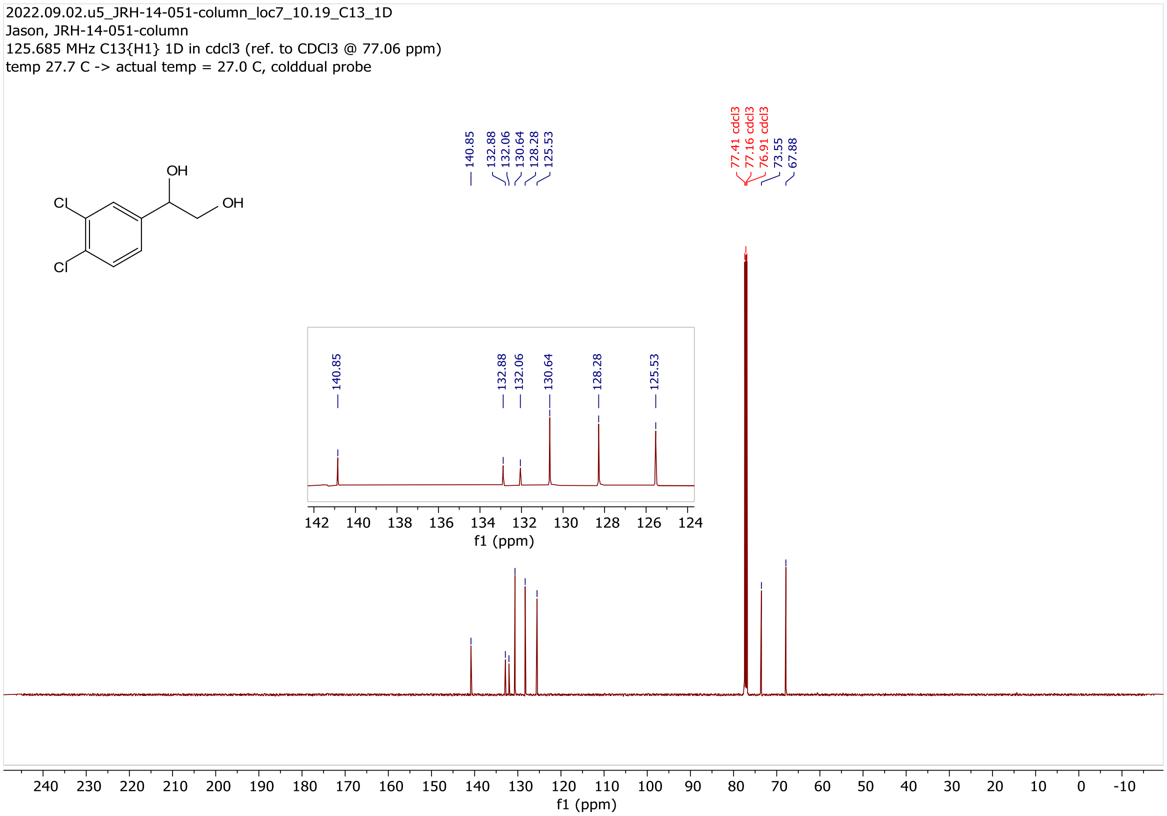

^{1}H (500 MHz) and ^{13}C (126 MHz) NMR of compound 4h ($CDCl_3$)

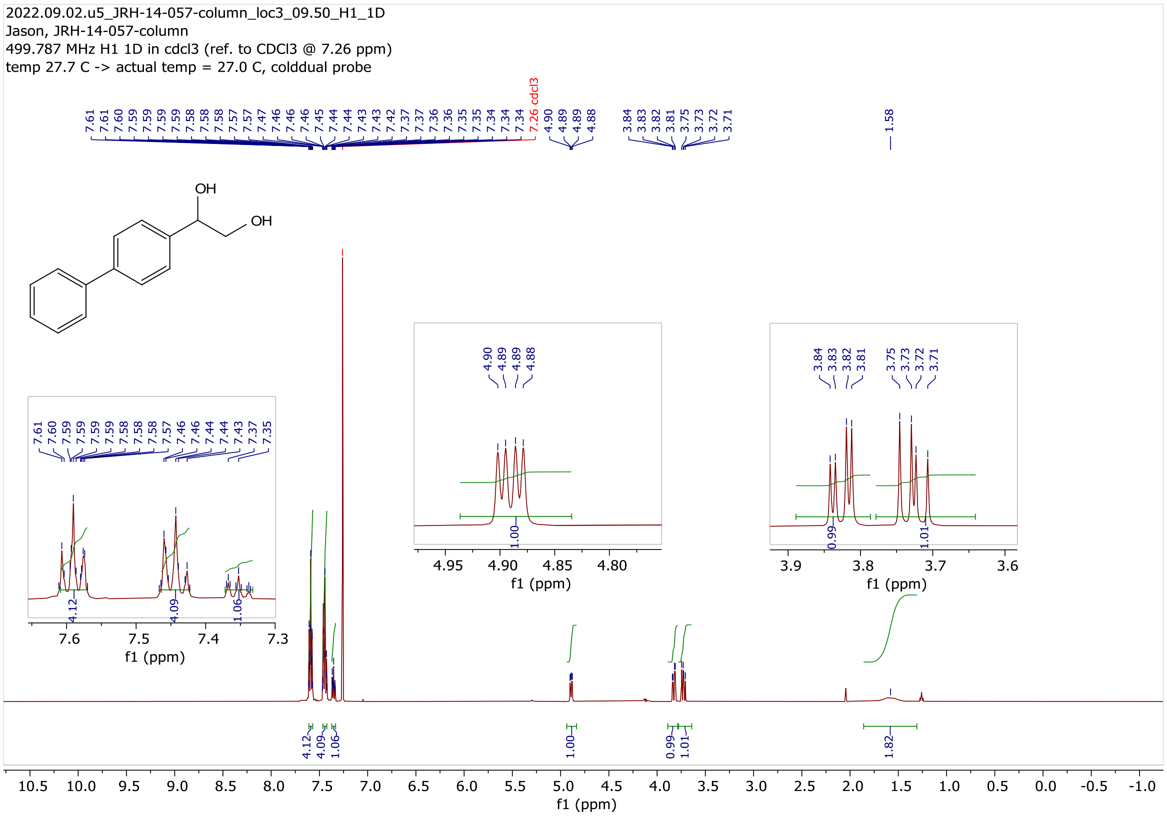

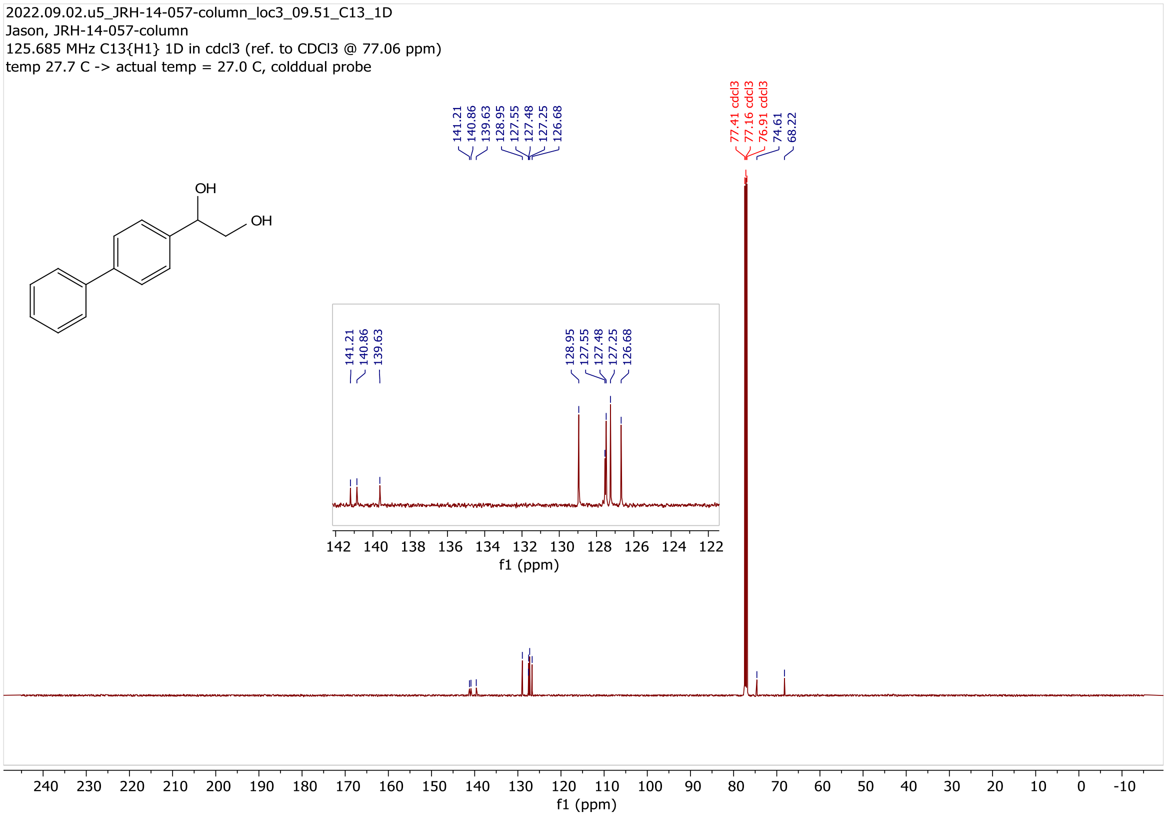

^{1}H (500 MHz), ^{13}C (126 MHz) and ^{31}P NMR (162 MHz) of compound 5a ($CDCl_3$)

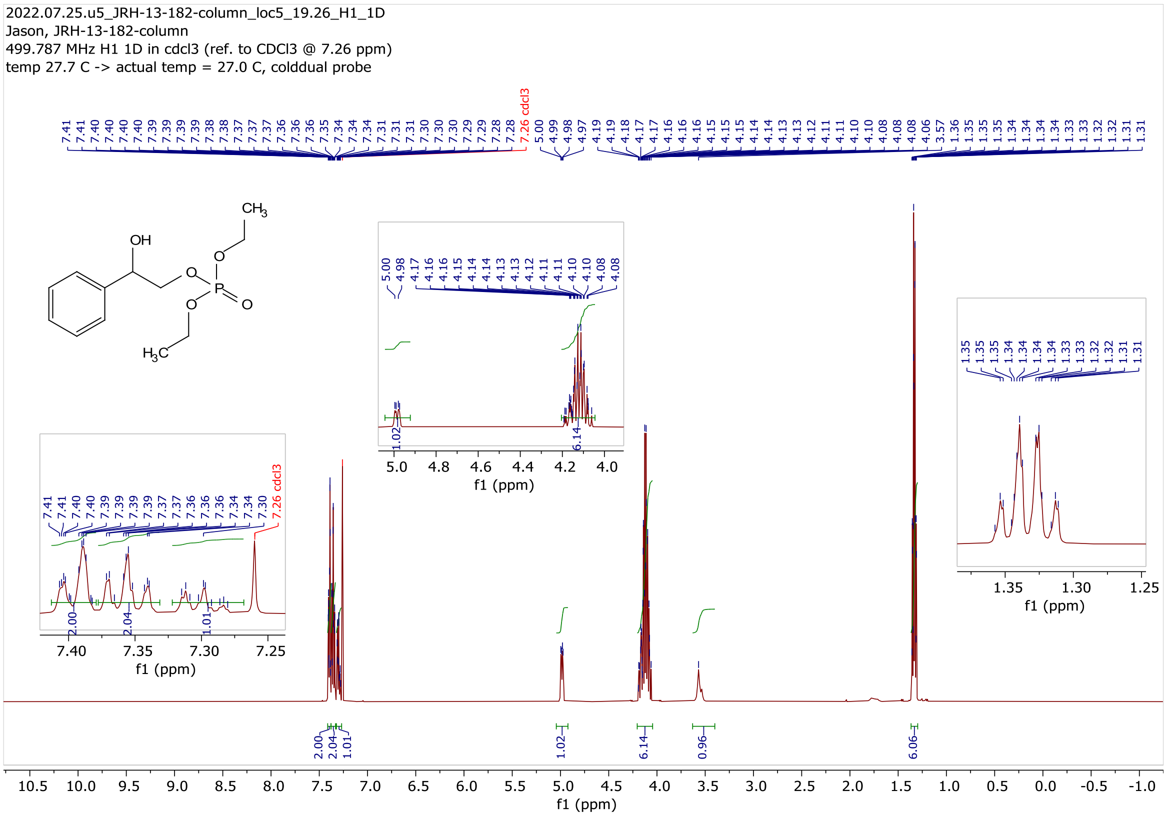

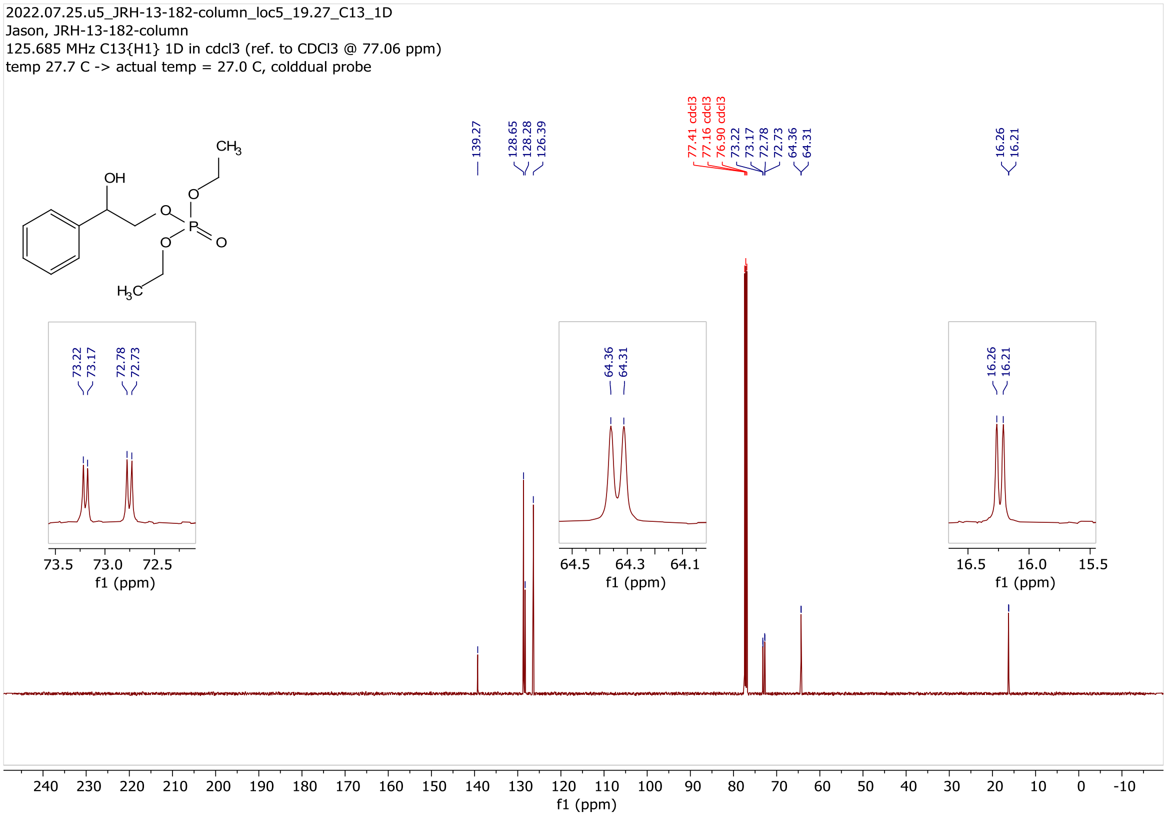

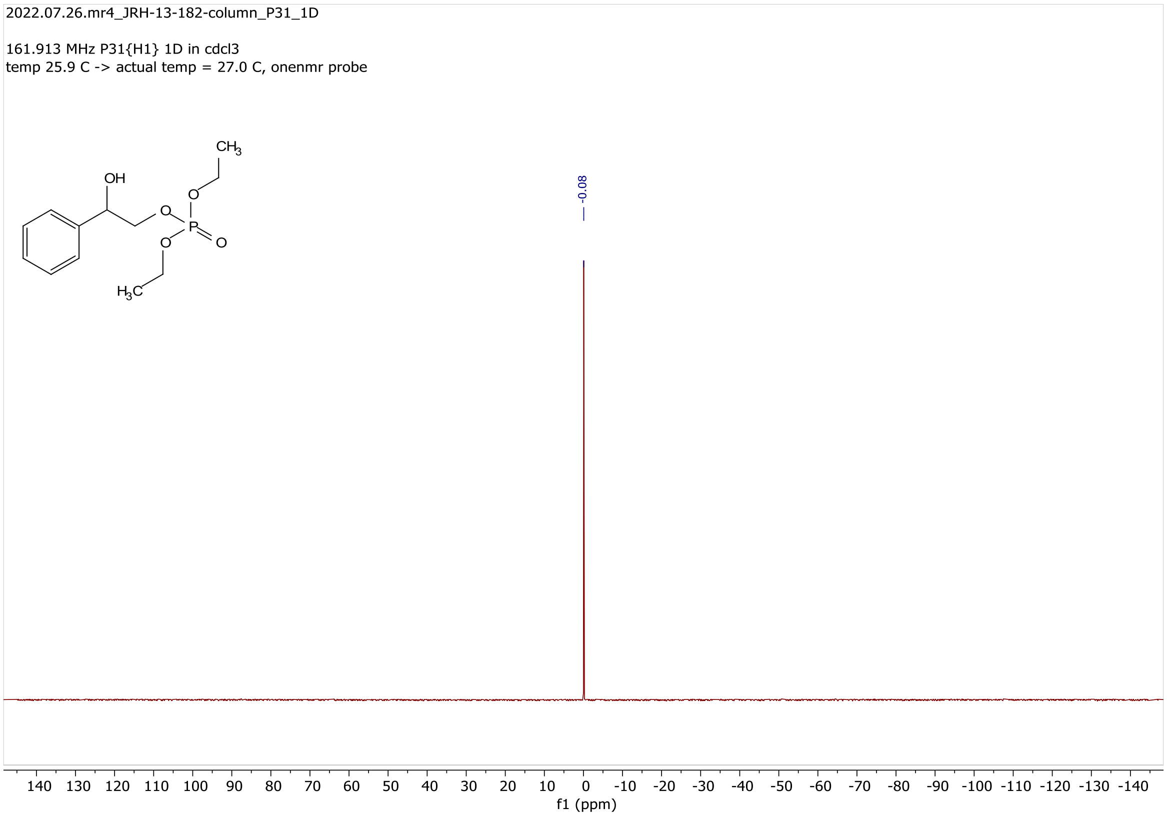

^{1}H (500 MHz), ^{13}C (126 MHz) and ^{31}P (162 MHz) NMR of compound 5b ($CDCl_3$)

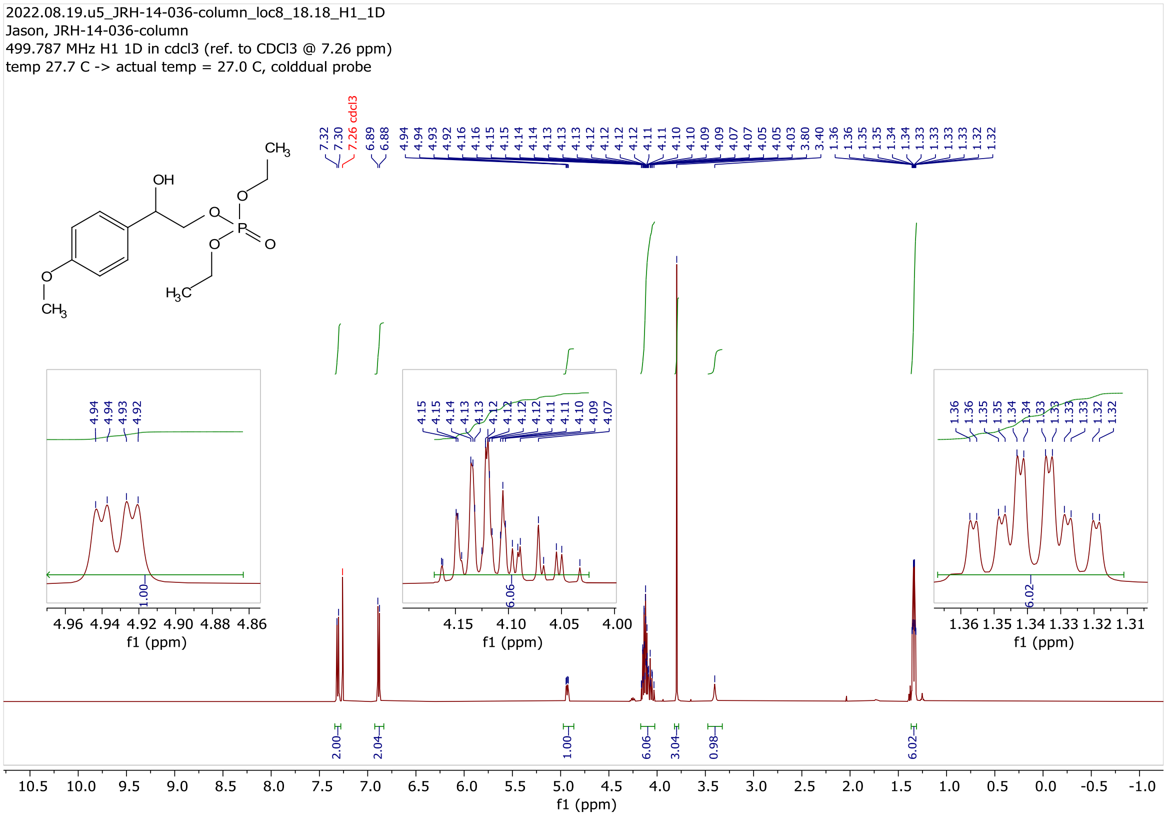

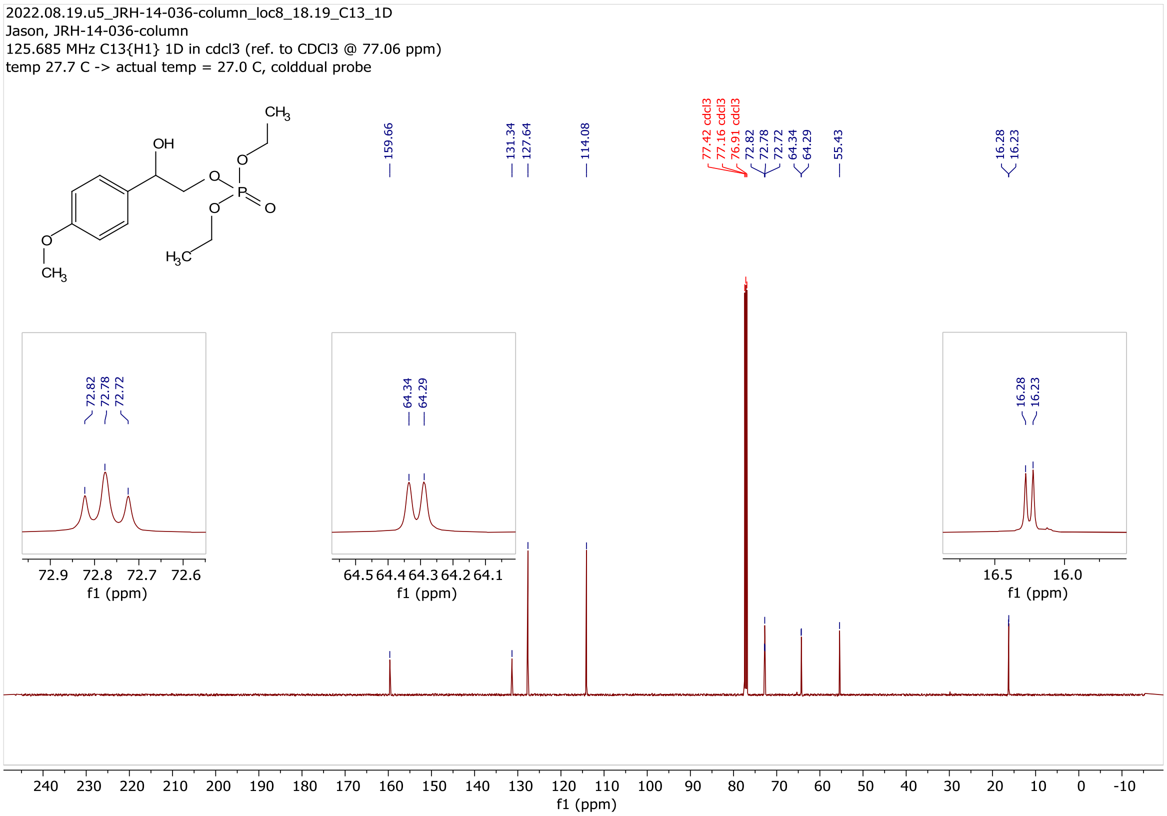
2022.08.19.u5_JRH-14-036-column_loc8_18.19_C13_1D
Jason, JRH-14-036-column
125.685 MHz C13{H1} 1D in cdcl3 (ref. to CDCl3 @ 77.06 ppm)
temp 27.7 C -> actual temp = 27.0 C, colddual probe
159.66
131.34
127.64
114.08
77.42 cdcl3
77.16 cdcl3
76.91 cdcl3
72.82
72.78
72.72
64.34
64.29
55.43
16.28
16.23
f1 (ppm)

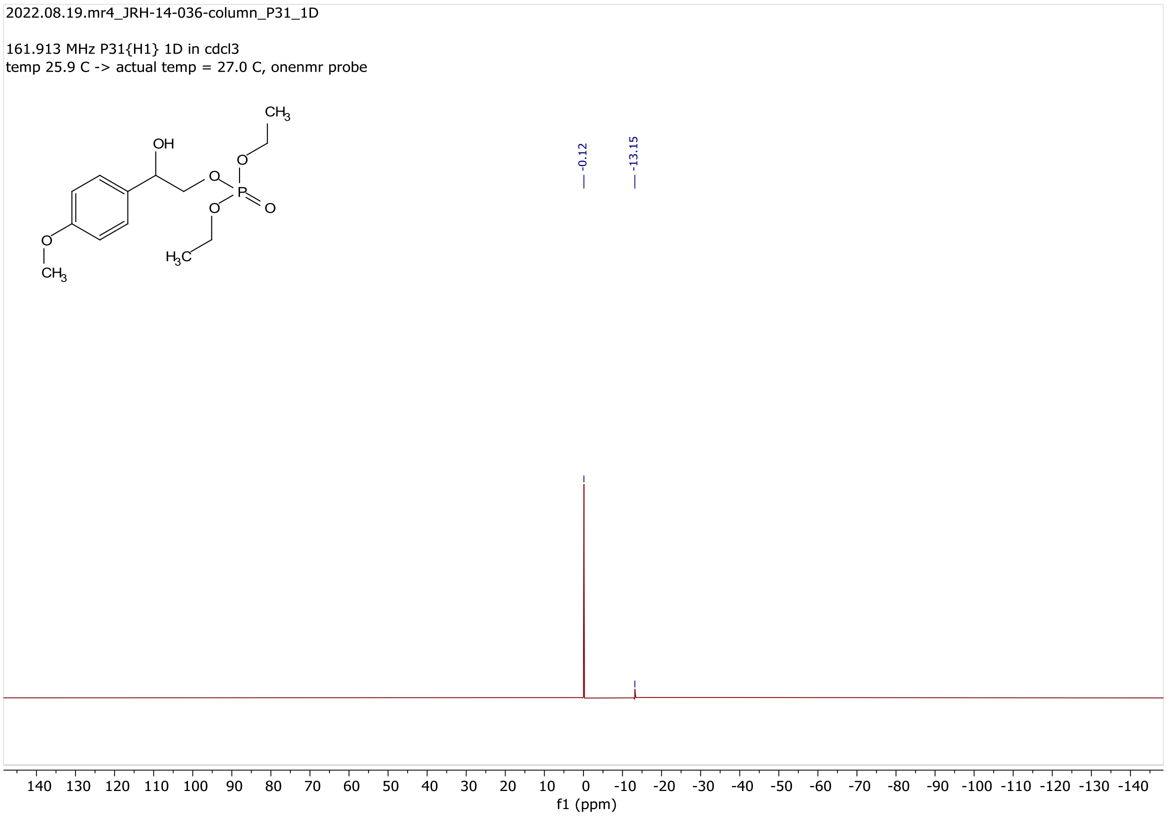
2022.08.19.mr4_JRH-14-036-column_P31_1D
161.913 MHz P31{H1} 1D in cdcl3
temp 25.9 C -> actual temp = 27.0 C, onenmr probe
-0.12
-13.15
f1 (ppm)

^{1}H (500 MHz), ^{13}C (126 MHz), ^{19}F (376 MHz) and ^{31}P (202 MHz) NMR of compound 5c ($CDCl_3$)

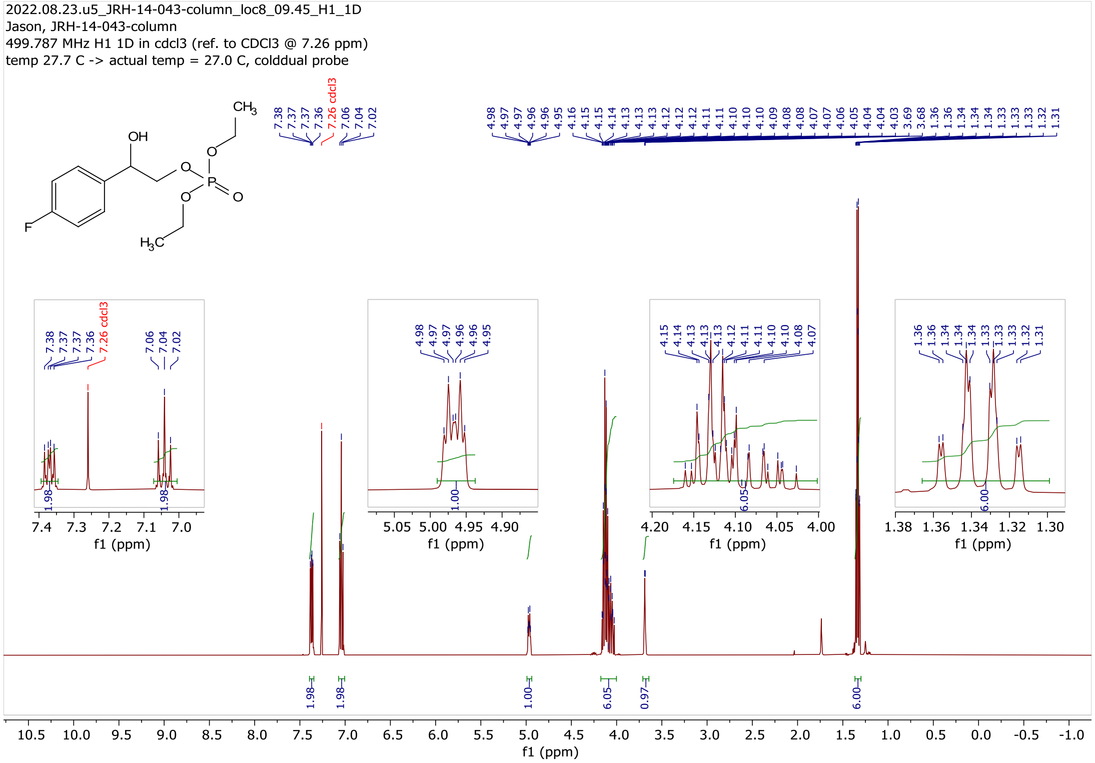

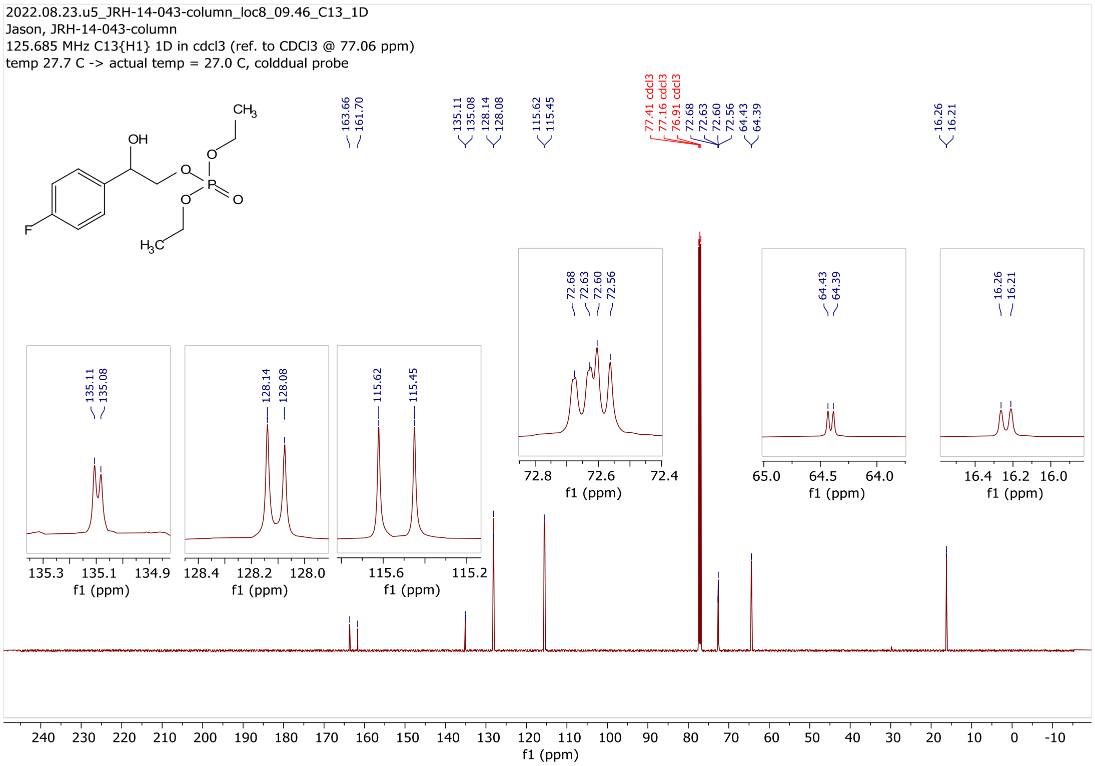

2022.09.20.mr4_JRH-14-043-column_F19_1D

376.306 MHz F19 1D in cdcl3
temp 25.9 C -> actual temp = 27.0 C, onenmr probe

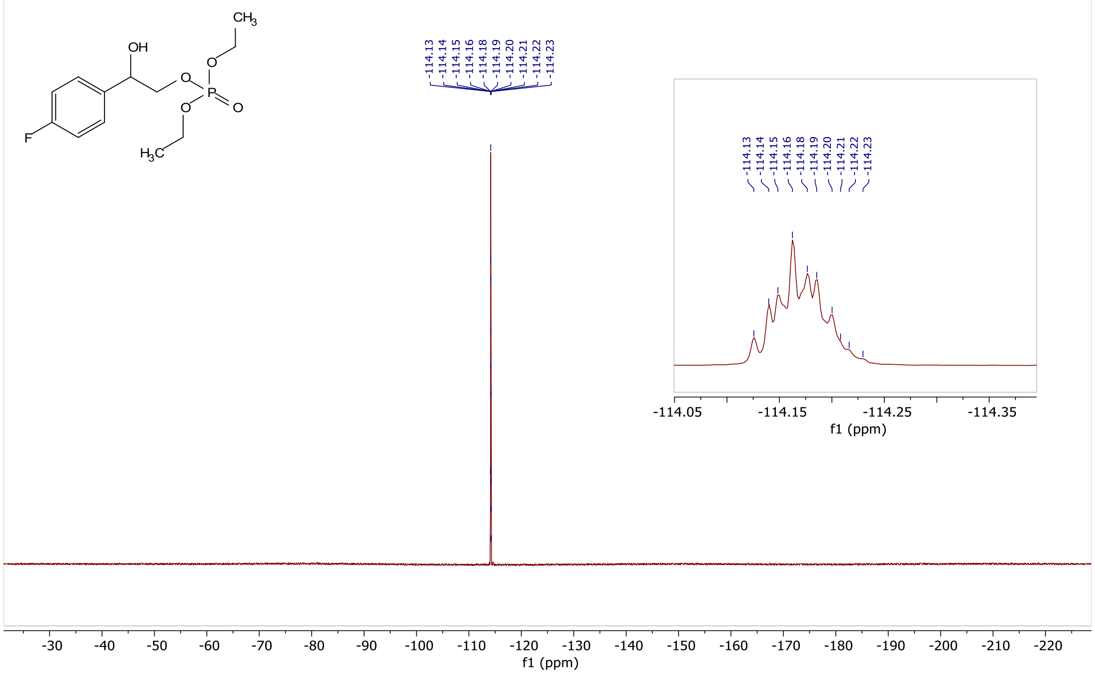

2022.09.20.i5_JRH-14-043-column_P31_1D

201.641 MHz P31{H1} 1D in cdcl3
temp 26.9 C -> actual temp = 27.0 C, autoxdb probe

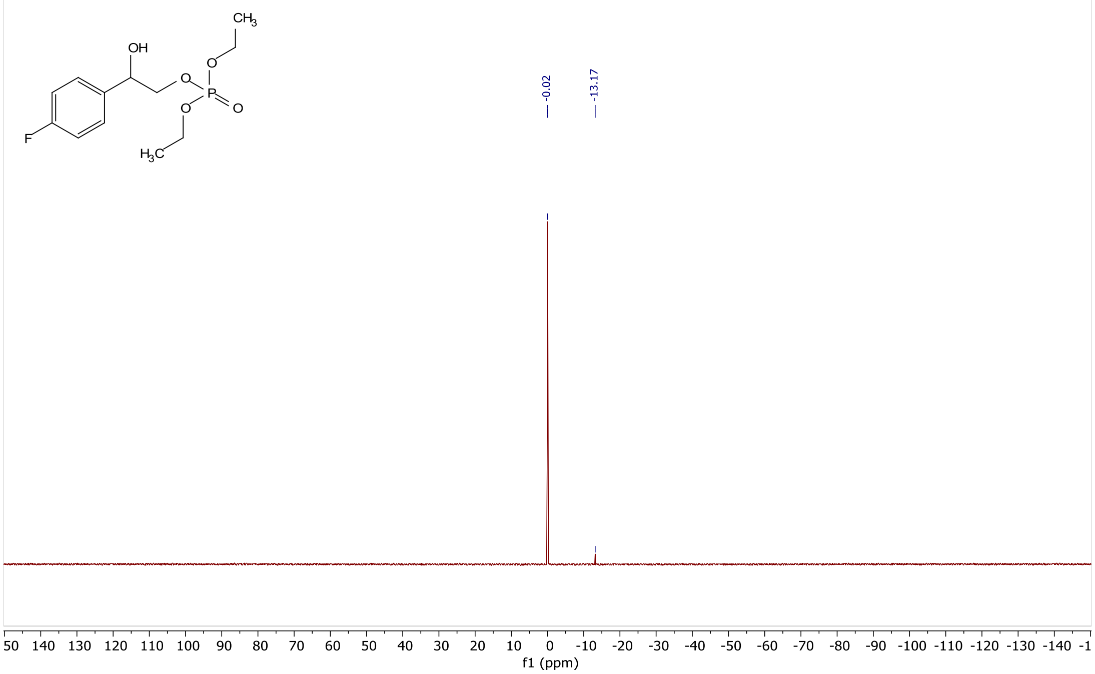

^{1}H (500 MHz), ^{13}C (126 MHz) and ^{31}P (162 MHz) NMR of compound 5d ($CDCl_3$)

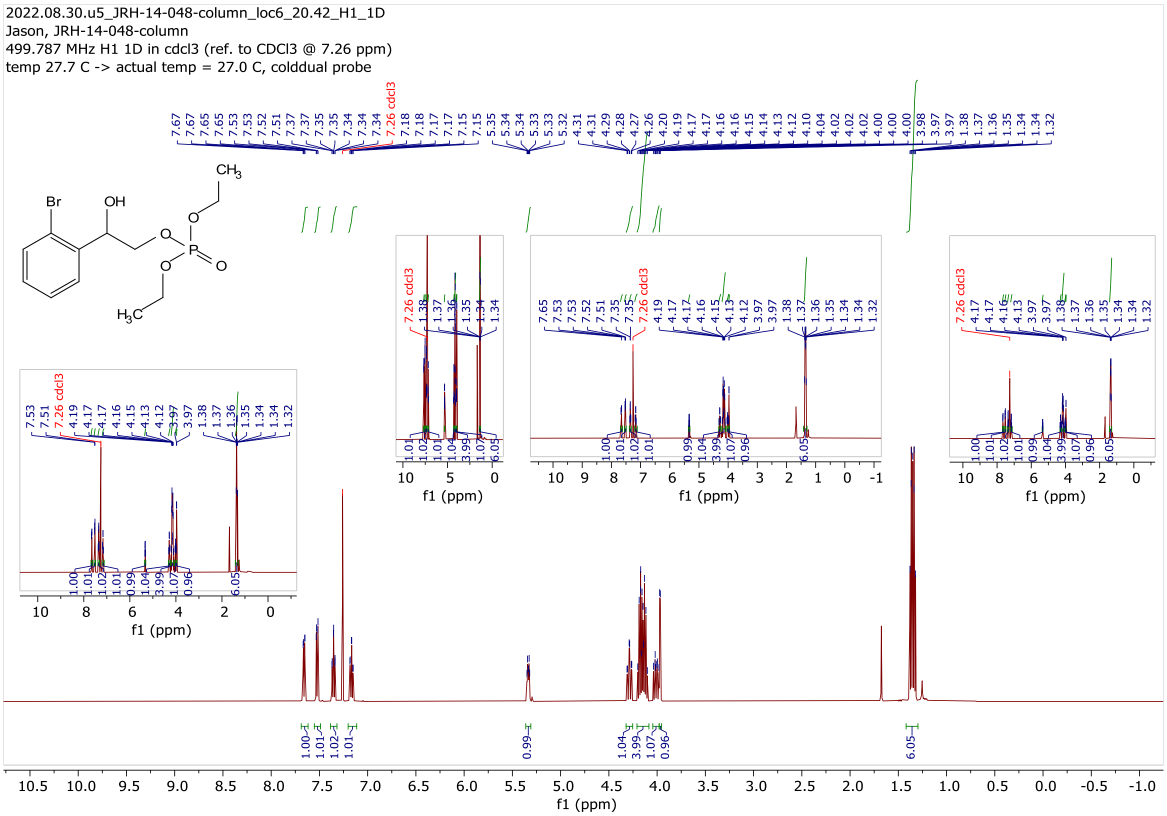

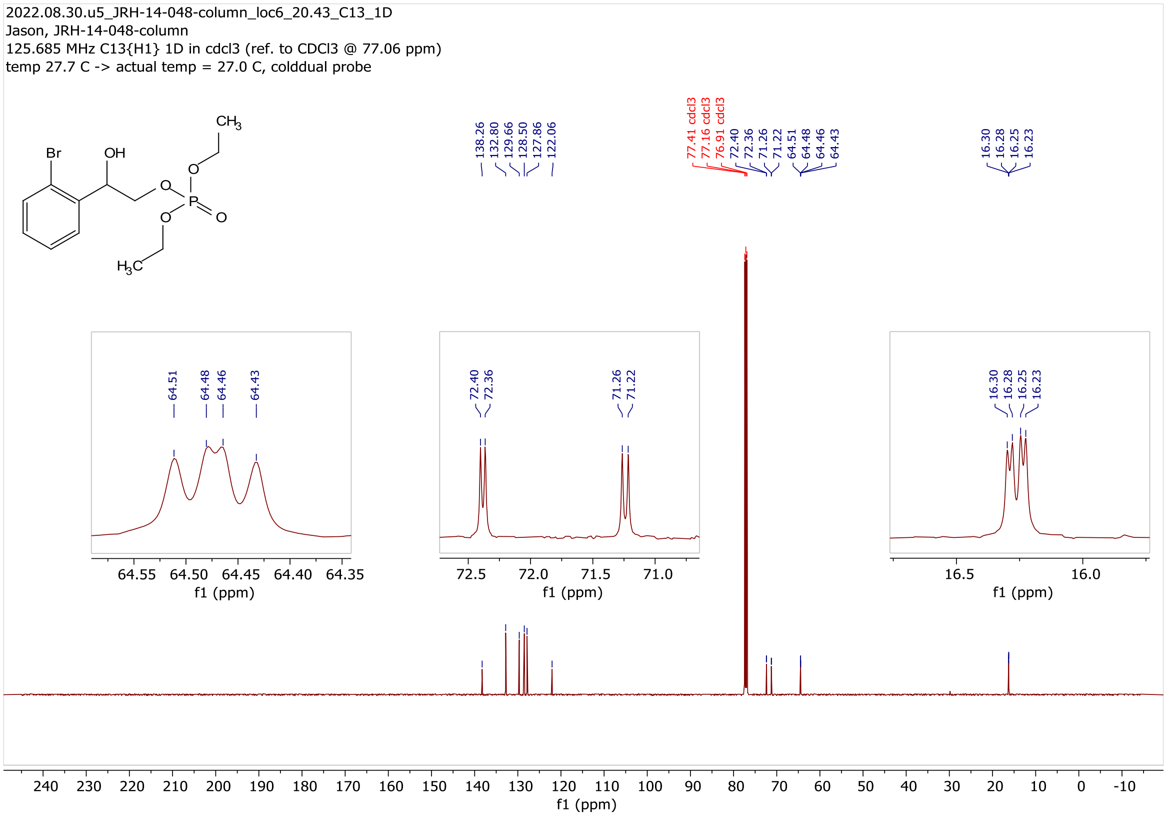

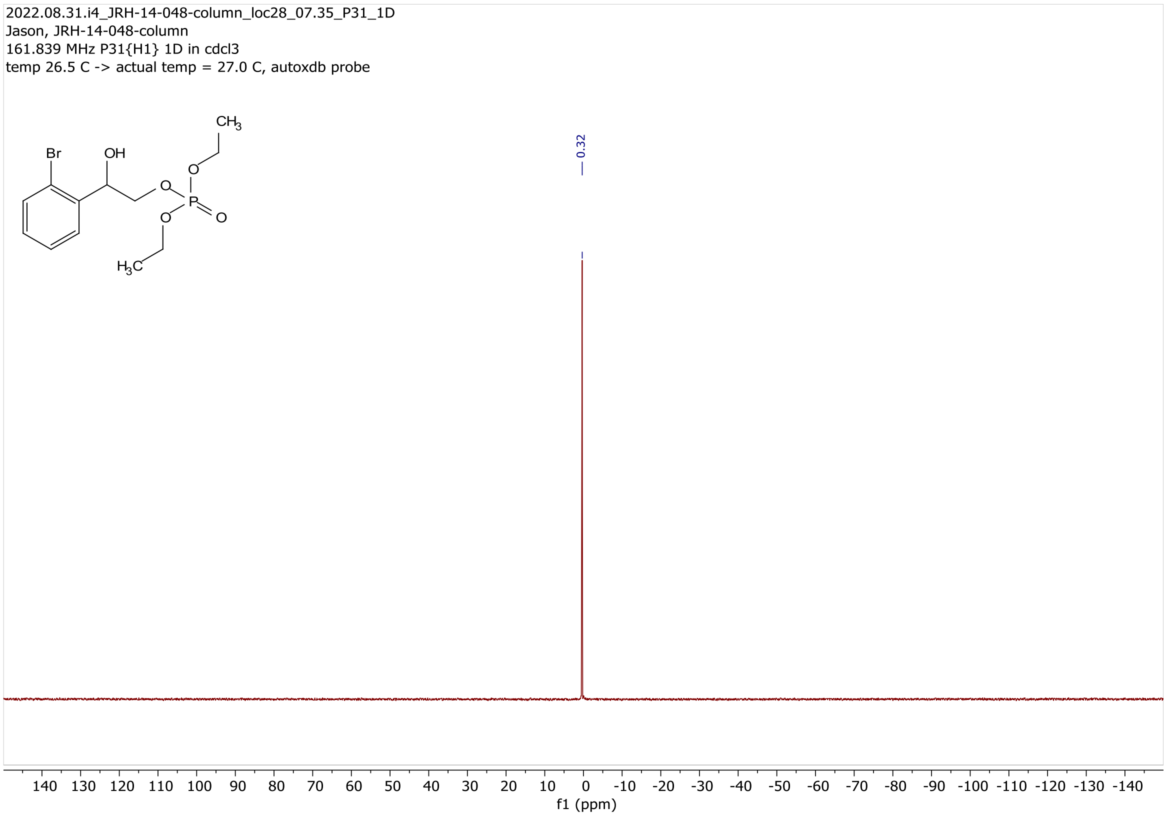

^{1}H (500 MHz), ^{13}C (126 MHz) and ^{31}P (202 MHz) NMR of compound 5e ($CDCl_3$)

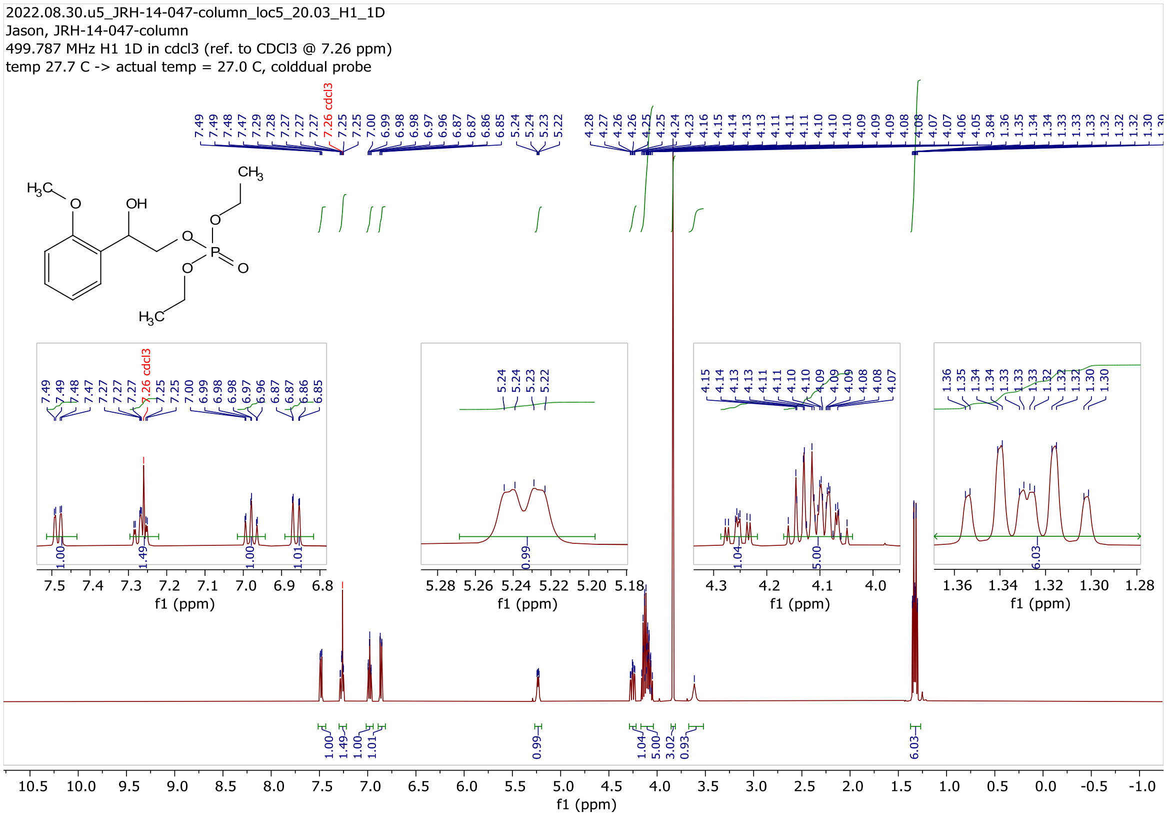

2022.08.30.u5_JRH-14-047-column_loc5_20.04_C13_1D
Jason, JRH-14-047-column
125.685 MHz C13{H1} 1D in cdcl3 (ref. to CDCl3 @ 77.06 ppm)
temp 27.7 C -> actual temp = 27.0 C, colddual probe

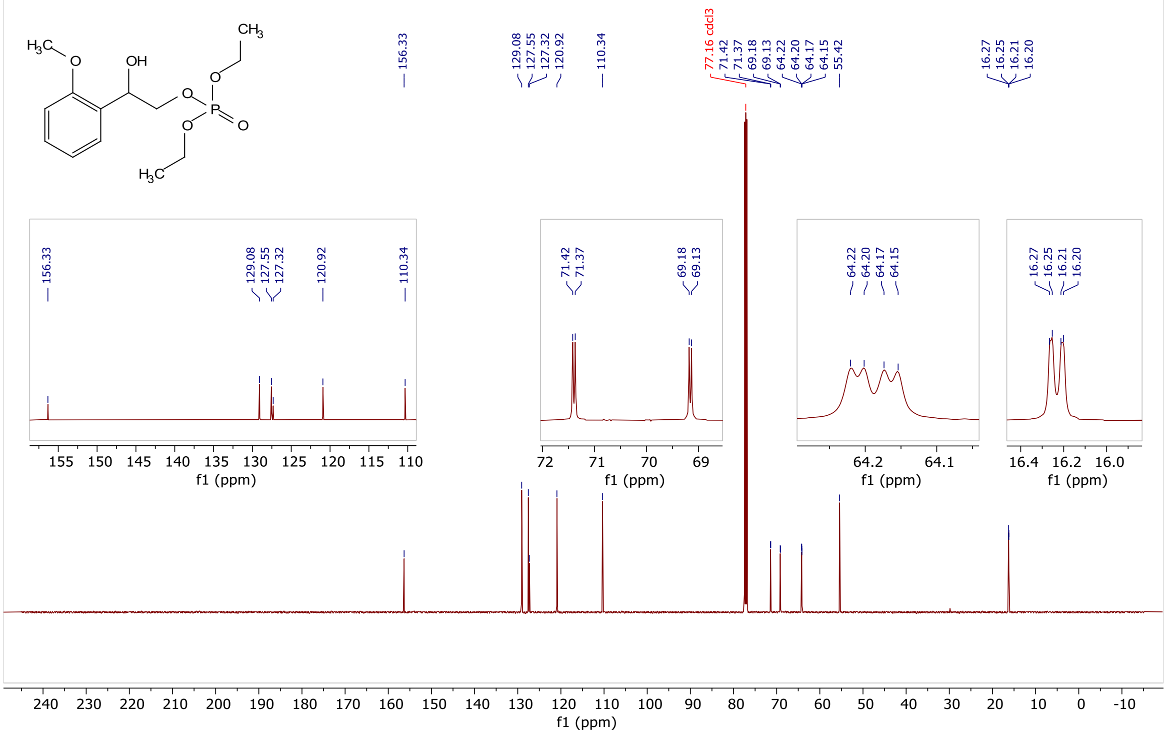

2022.08.31.i5_JRH-14-047-column_P31_1D

201.641 MHz P31{H1} 1D in cdcl3
temp 26.9 C -> actual temp = 27.0 C, autoxdb probe

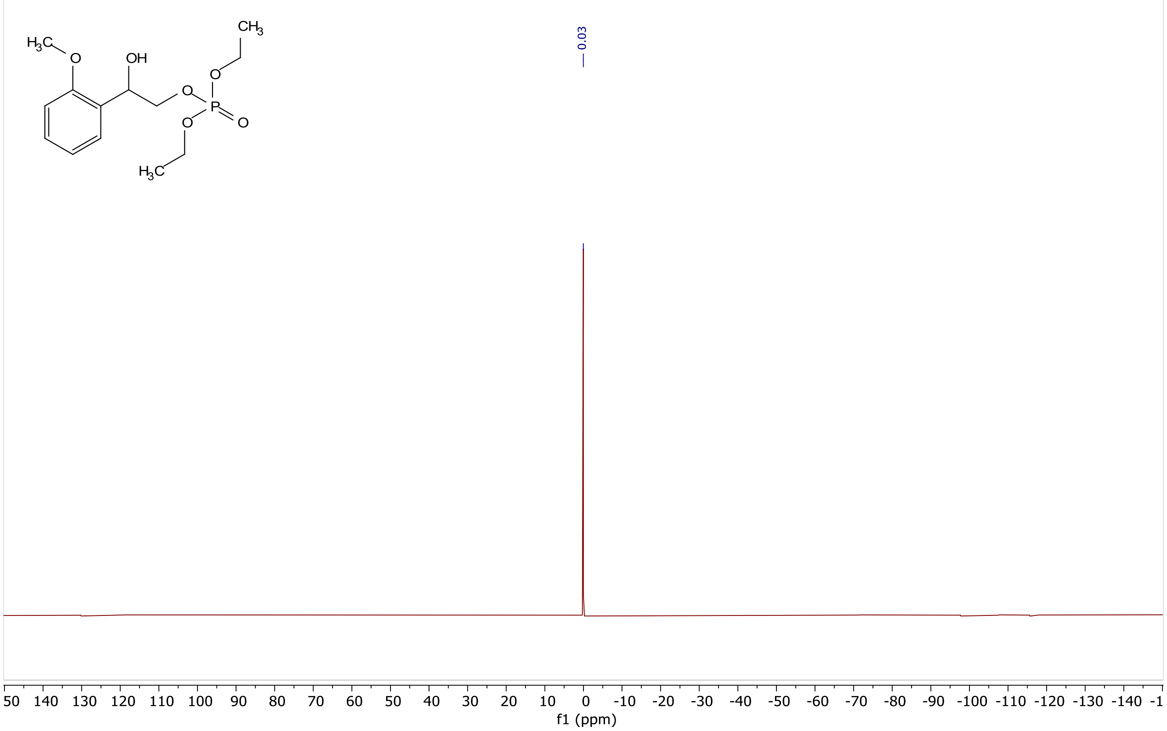

^{1}H (500 MHz), ^{13}C (126 MHz) and ^{31}P (202 MHz) NMR of compound 5f ($CDCl_3$)

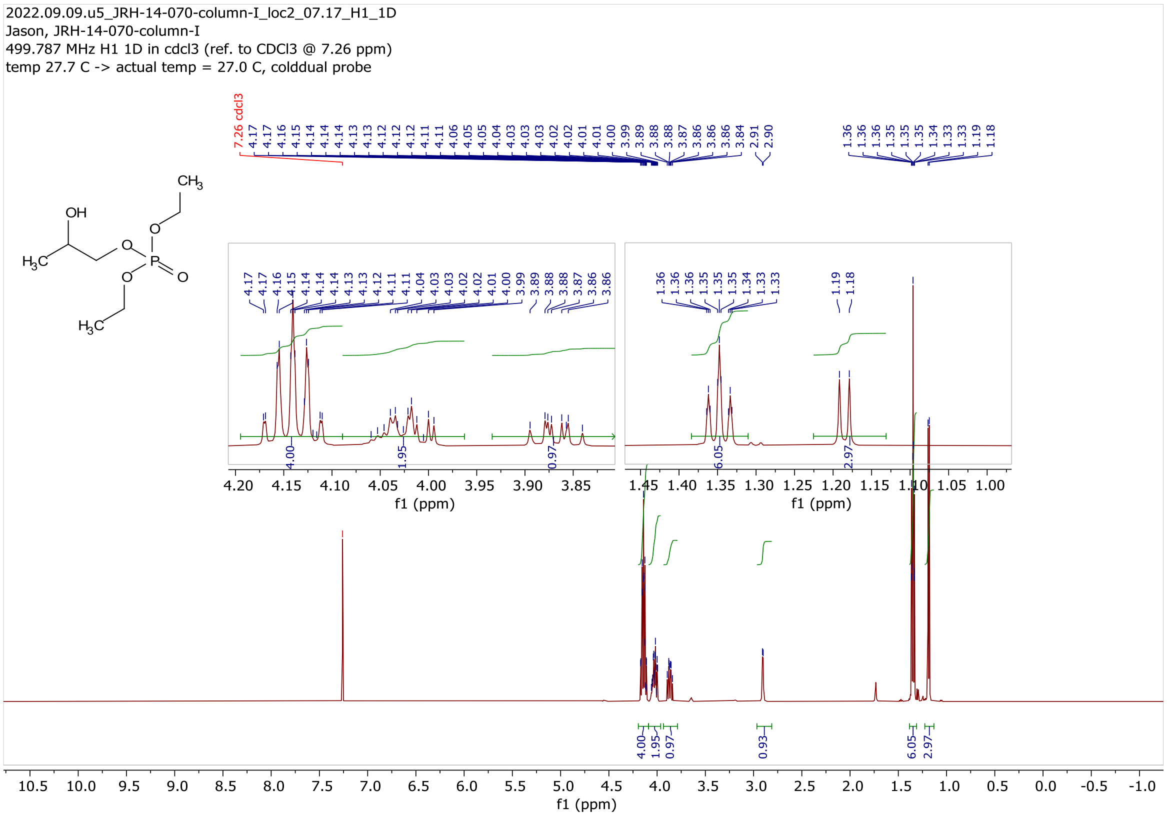

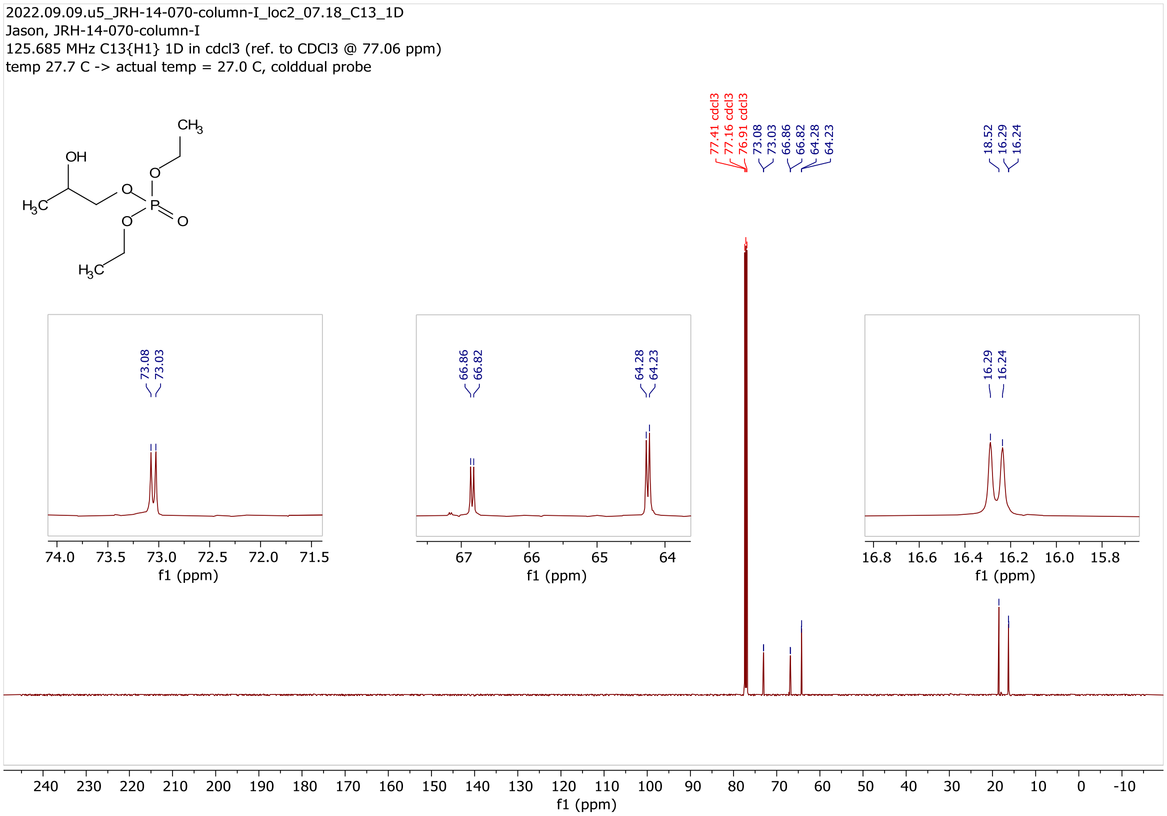

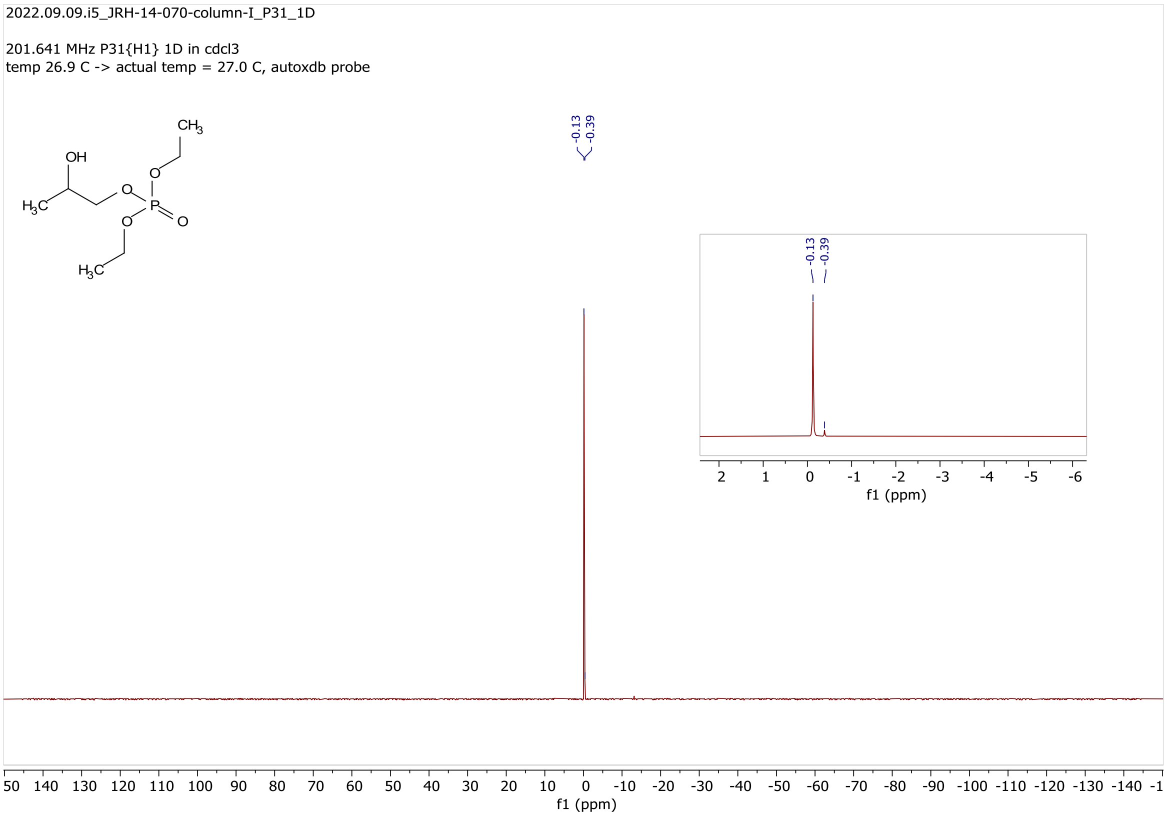

^{1}H (500 MHz), ^{13}C (126 MHz) and ^{31}P (202 MHz) NMR of compound 5g ($CDCl_3$)

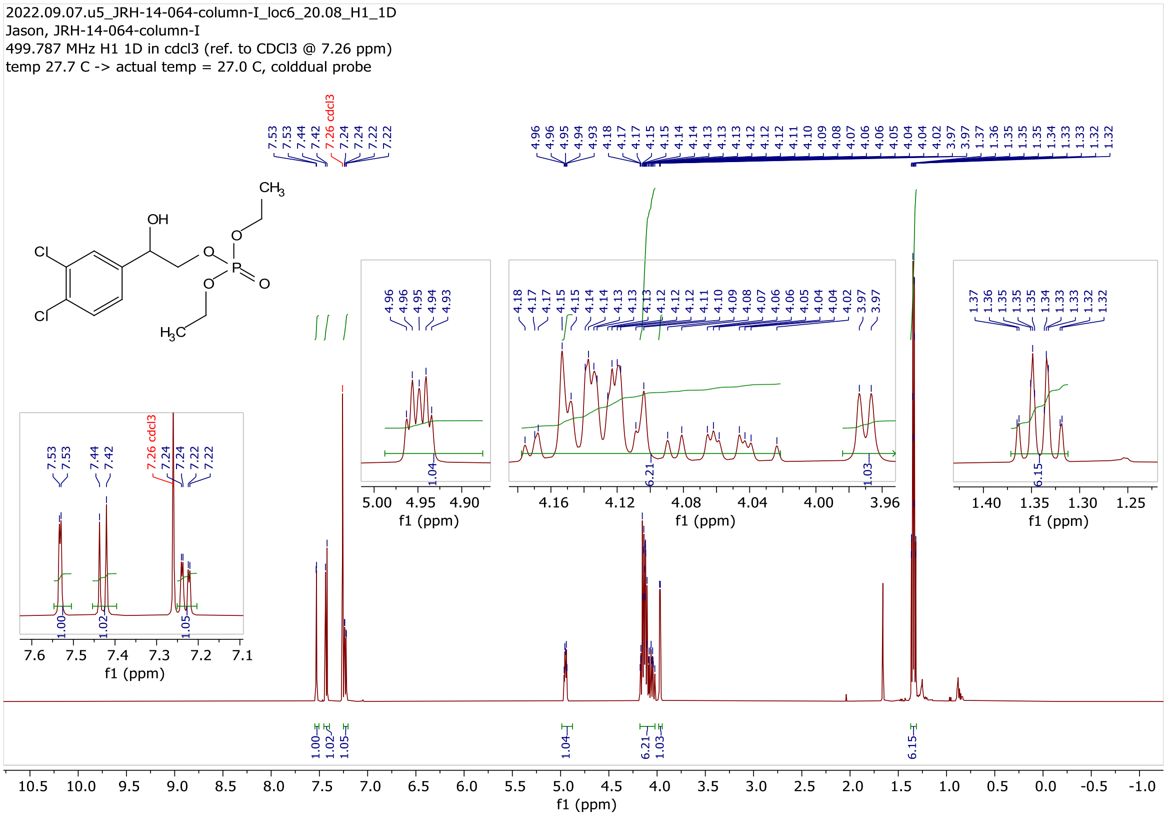

2022.09.07.u5_JRH-14-064-column-I_loc6_20.09_C13_1D
Jason, JRH-14-064-column-I
125.685 MHz C13{H1} 1D in cdcl3 (ref. to CDCl3 @ 77.06 ppm)
temp 27.7 C -> actual temp = 27.0 C, colddual probe

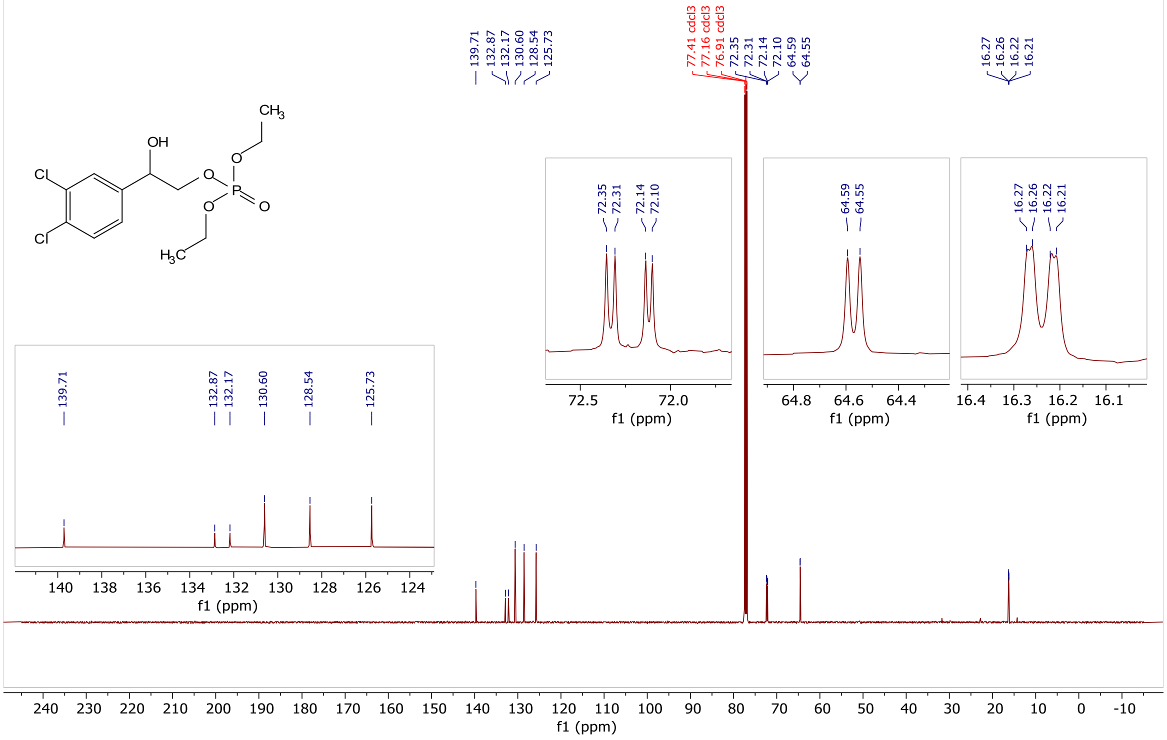

2022.09.07.i5_JRH-14-064-column-II_P31_1D

201.641 MHz P31{H1} 1D in cdcl3
temp 26.9 C -> actual temp = 27.0 C, autoxdb probe

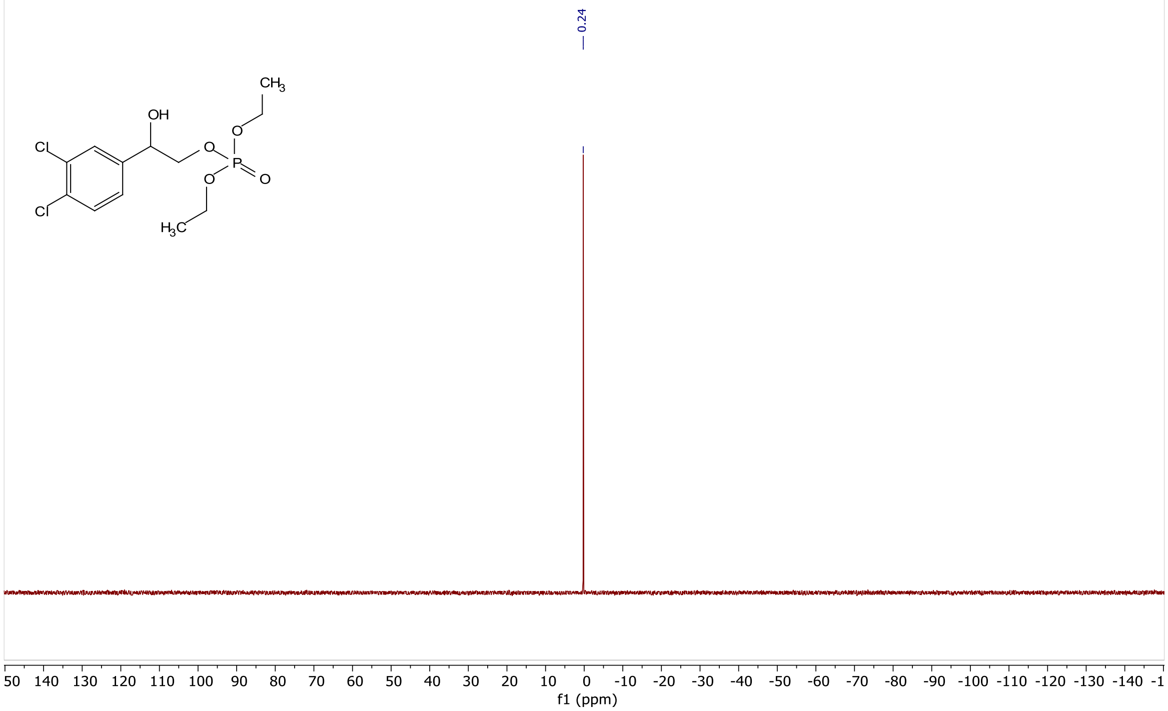

^{1}H (500 MHz), ^{13}C (126 MHz) and ^{31}P (202 MHz) NMR of compound 5h ($CDCl_3$)

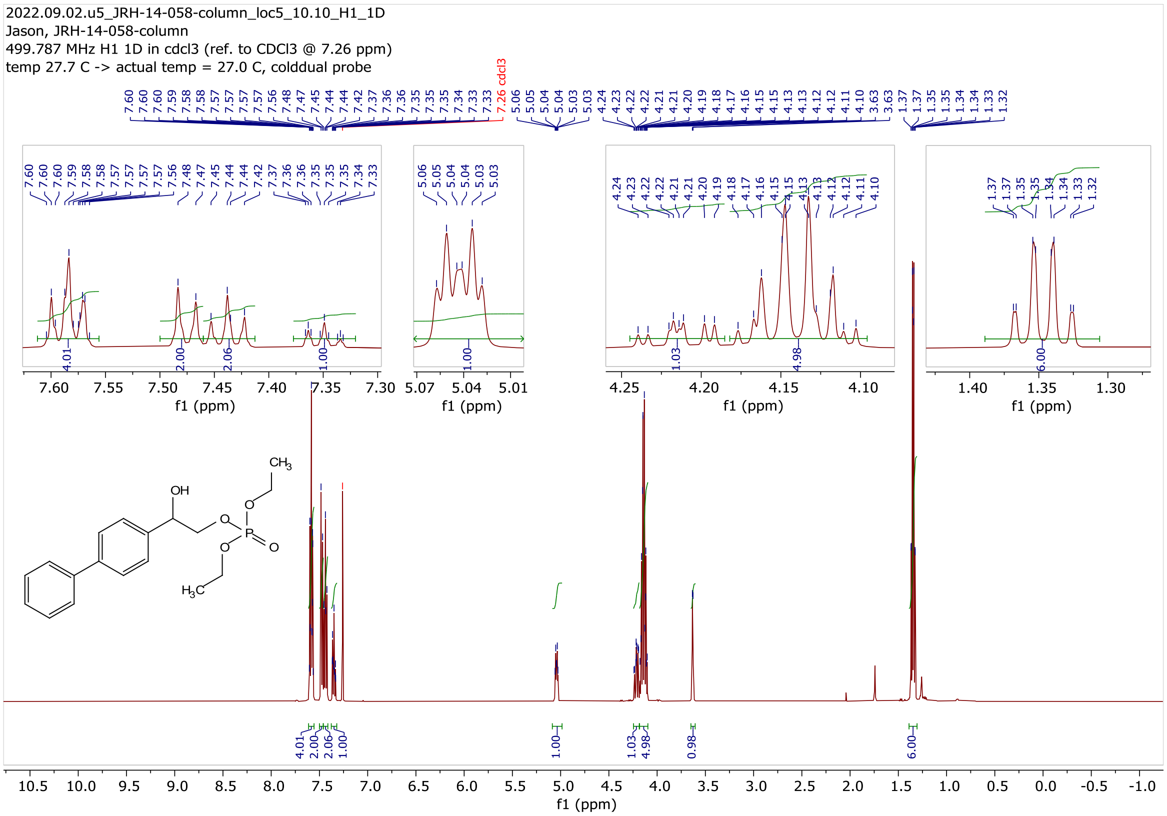

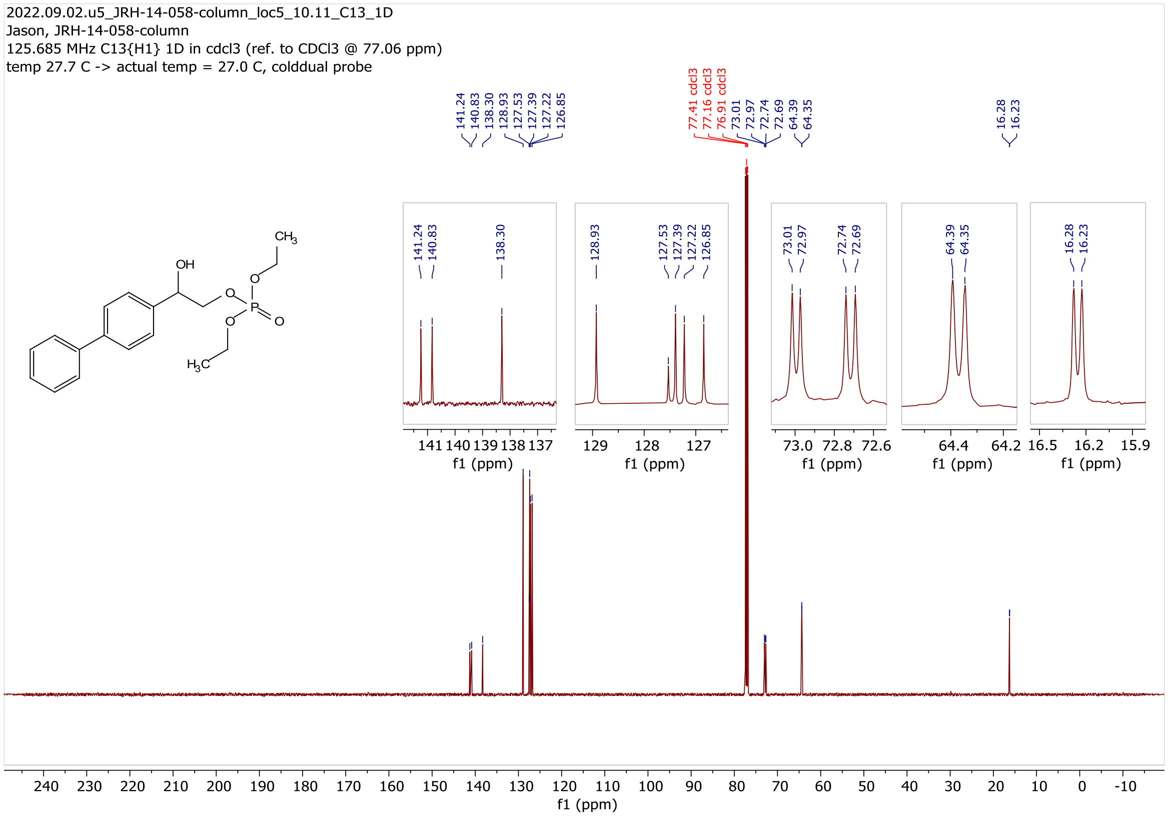

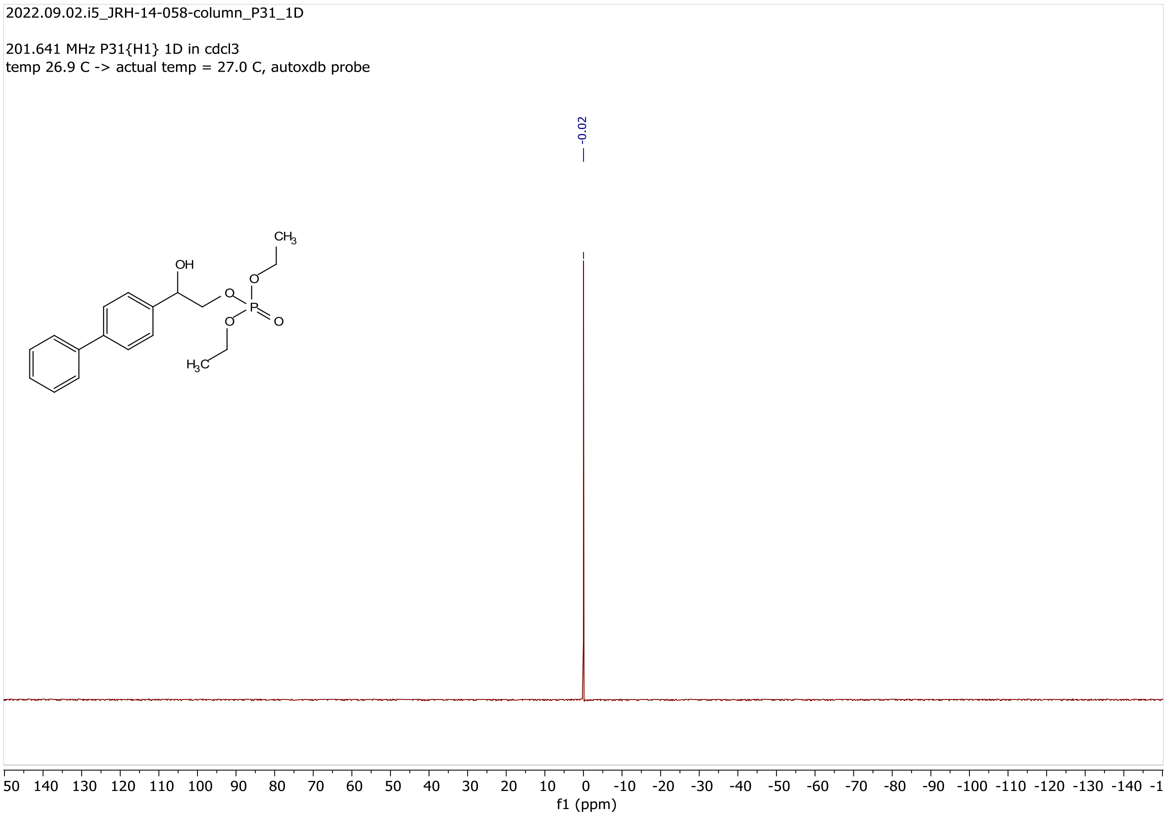

^{1}H (500 MHz), ^{13}C (126 MHz) and ^{19}F (469 MHz) NMR of compound 6b ($CDCl_3$)

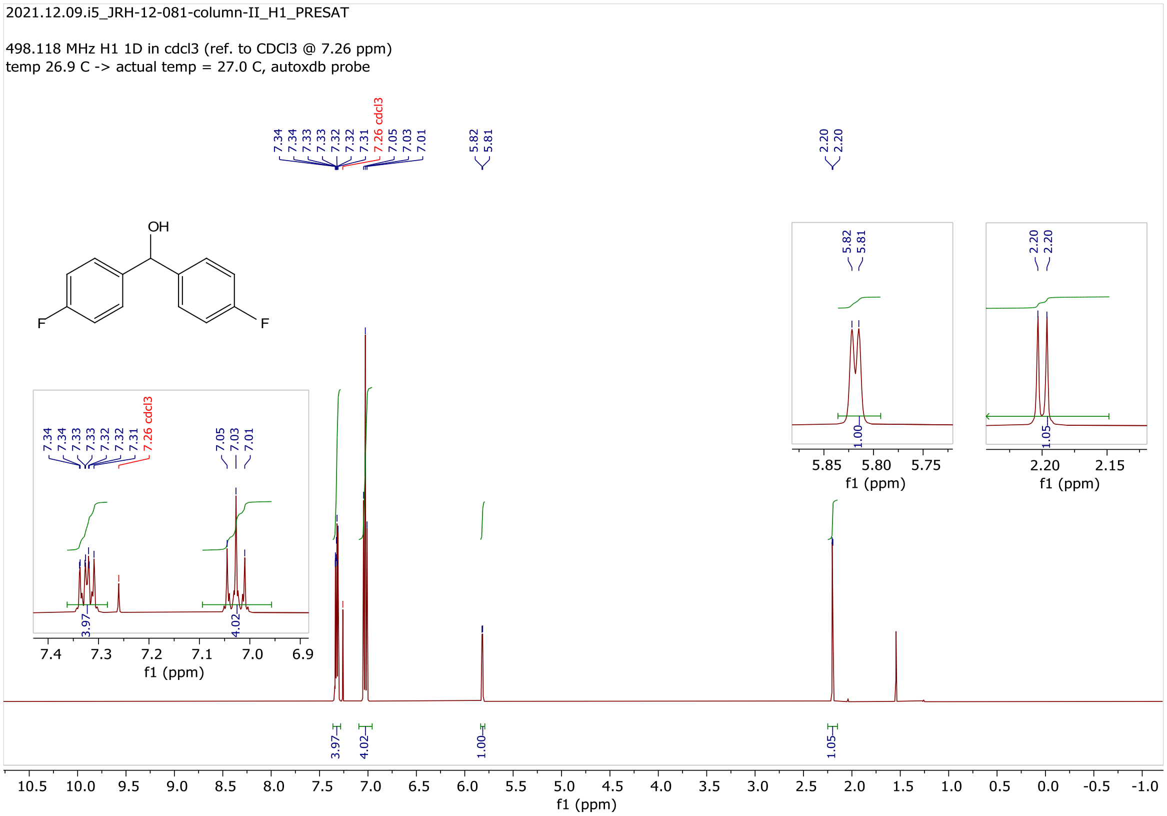

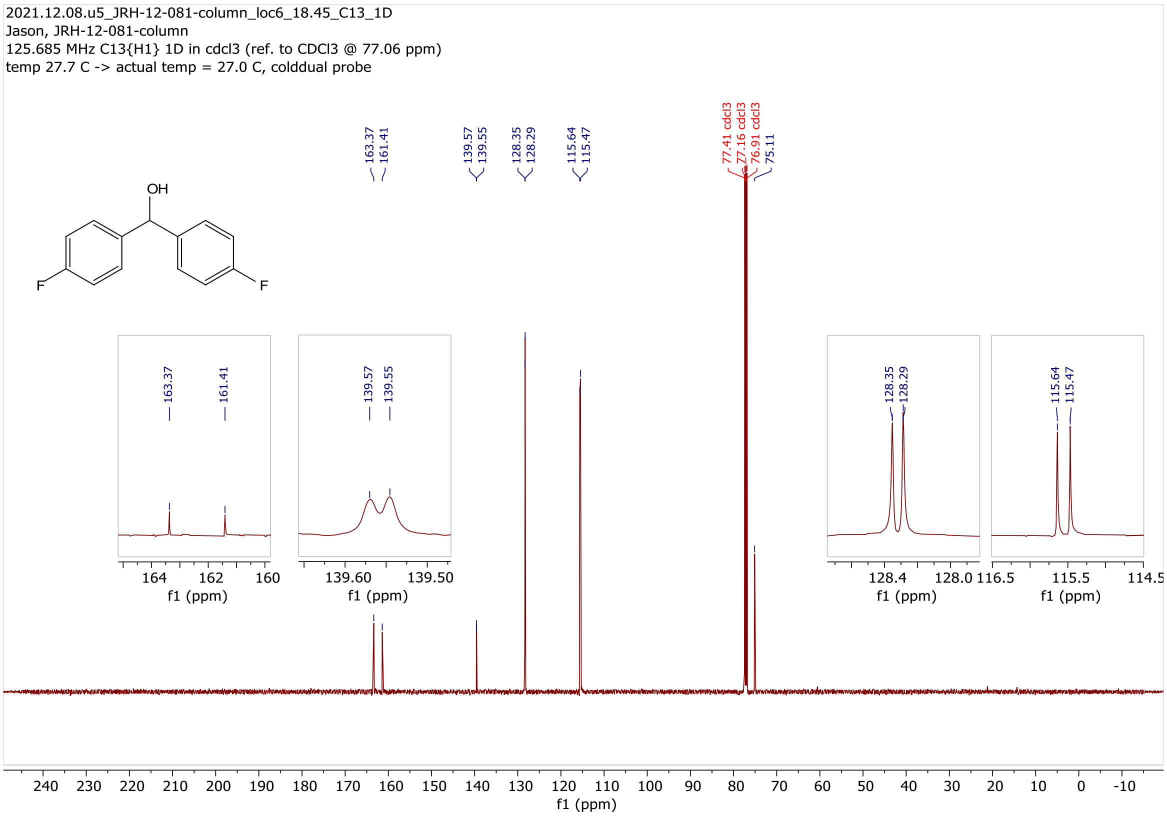
2021.12.08.u5_JRH-12-081-column_loc6_18.45_C13_1D
Jason, JRH-12-081-column
125.685 MHz C13{H1} 1D in cdcl3 (ref. to CDCl3 @ 77.06 ppm)
temp 27.7 C -> actual temp = 27.0 C, colddual probe
163.37
161.41
139.57
139.55
128.35
128.29
115.64
115.47
77.41 cdcl3
77.16 cdcl3
76.91 cdcl3
75.11
OH
F
F
f1 (ppm)

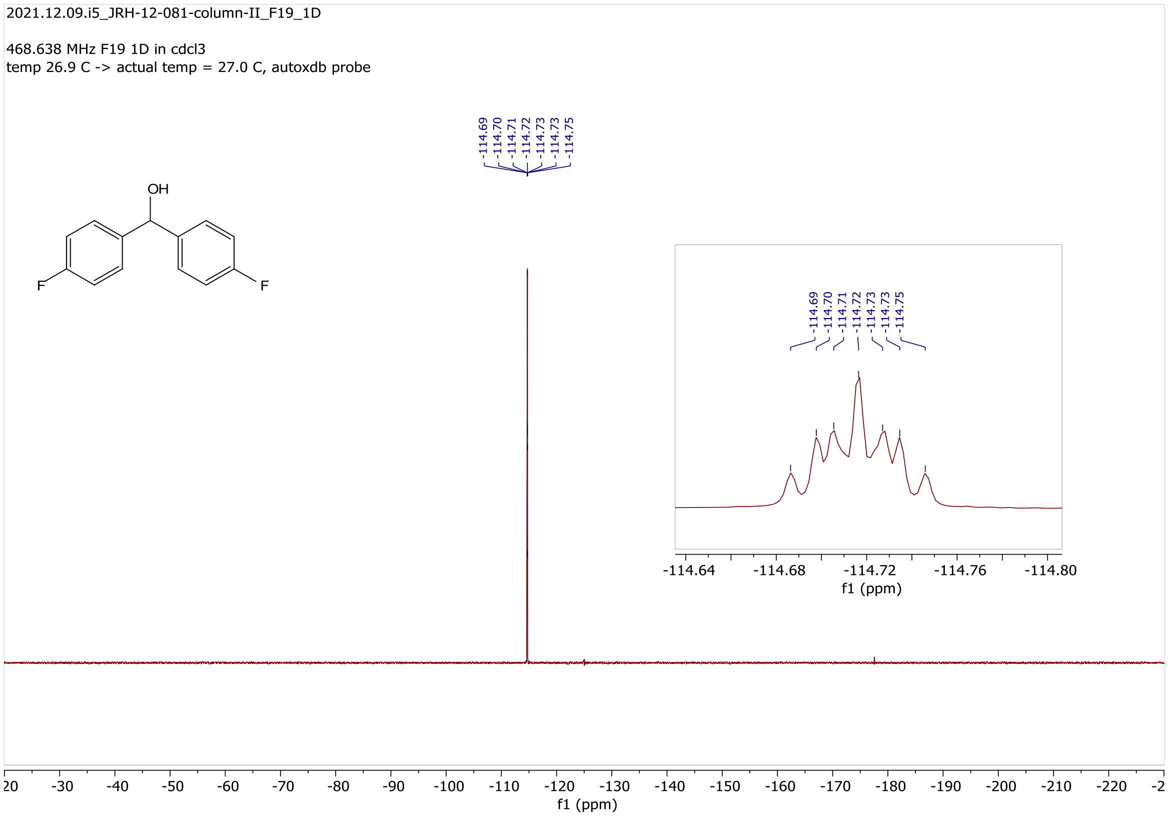
2021.12.09.i5_JRH-12-081-column-II_F19_1D
468.638 MHz F19 1D in cdcl3
temp 26.9 C -> actual temp = 27.0 C, autoxdb probe
-114.69
-114.70
-114.71
-114.72
-114.73
-114.73
-114.75
OH
F
F
f1 (ppm)

^{1}H (500 MHz) and ^{13}C (126 MHz) NMR of compound 6c ($CDCl_3$)

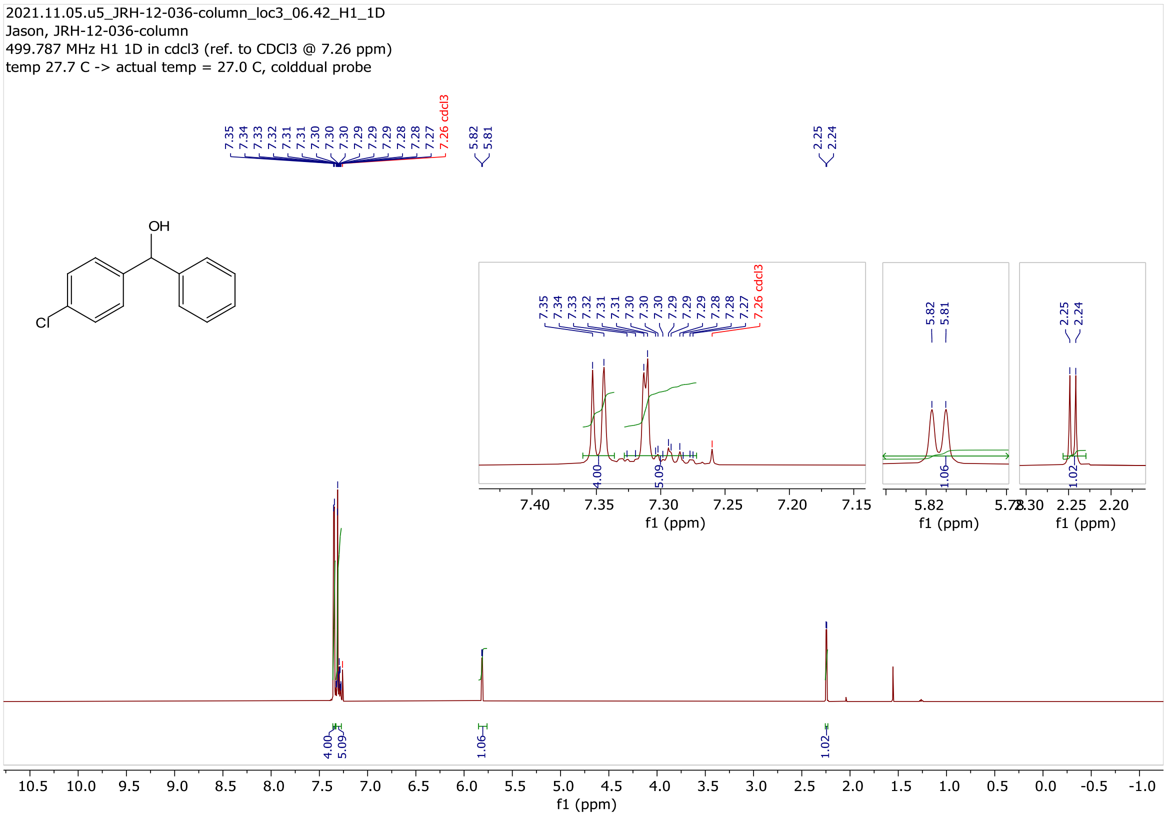

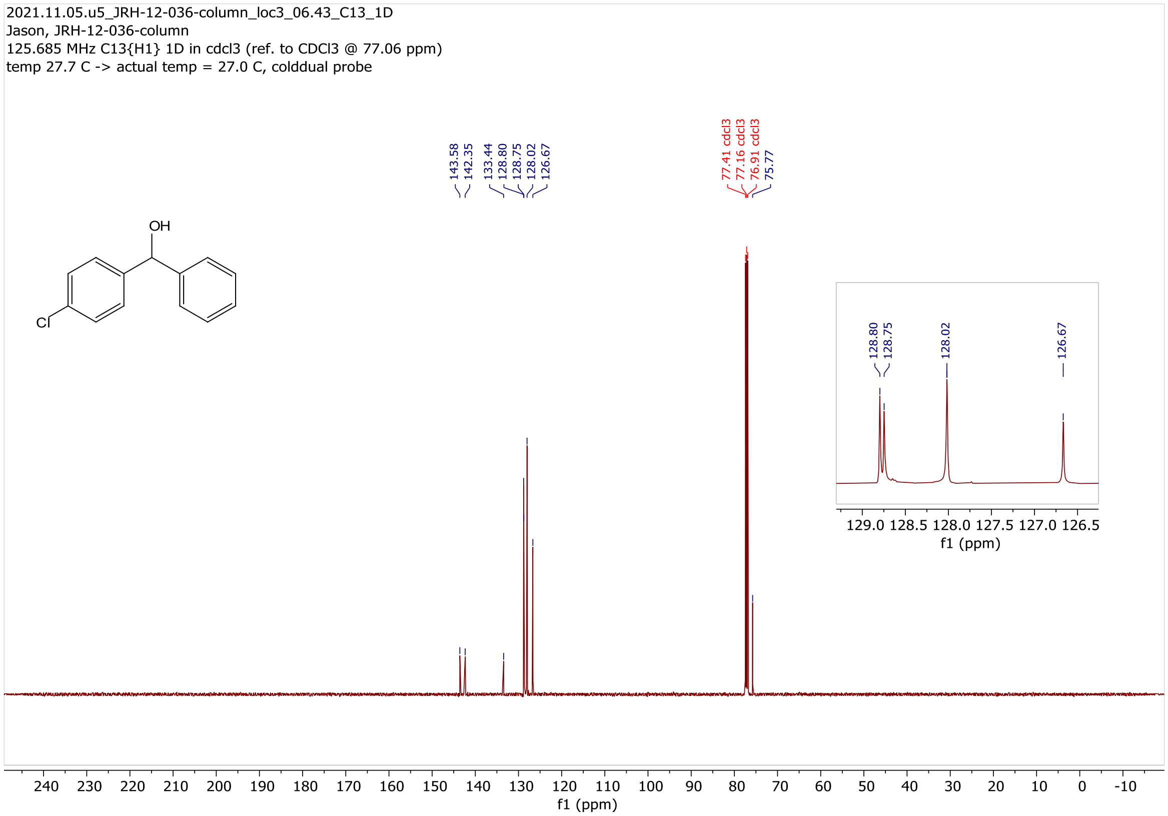

^{1}H (600 MHz) and ^{13}C (151 MHz) NMR of compound 6d ($CDCl_3$)

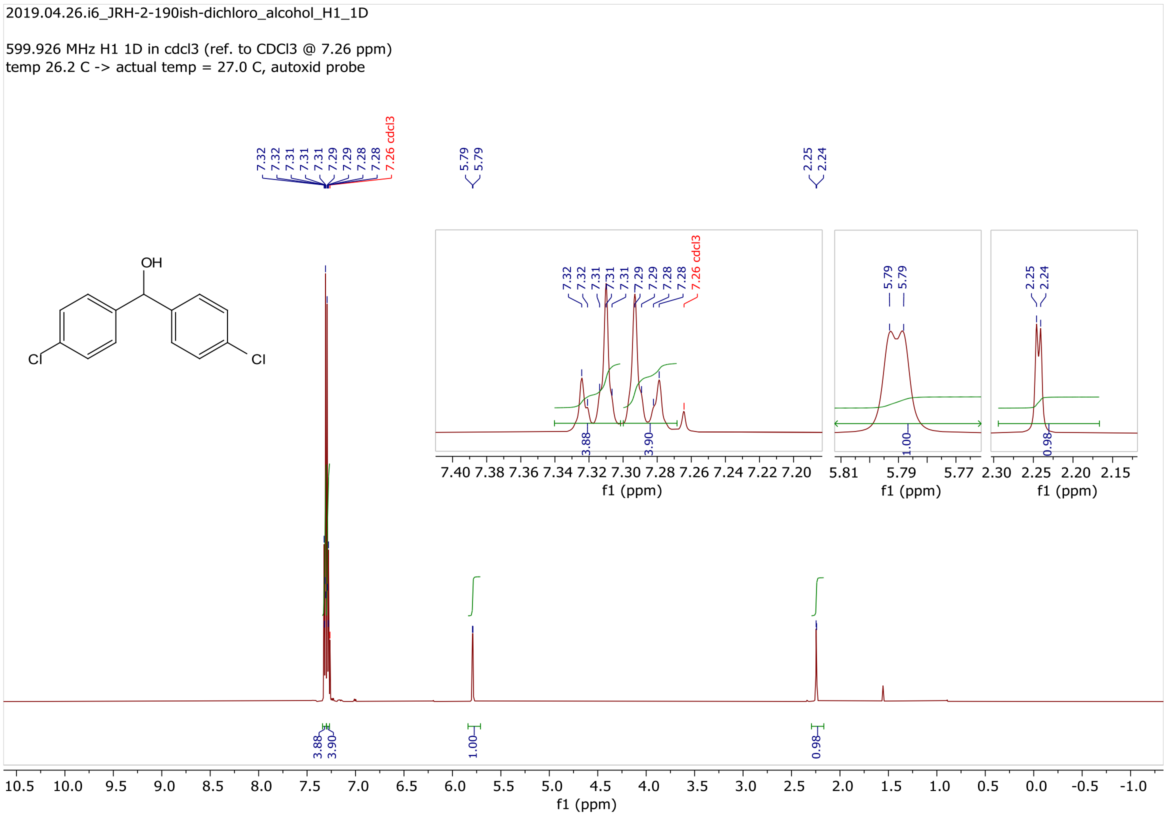

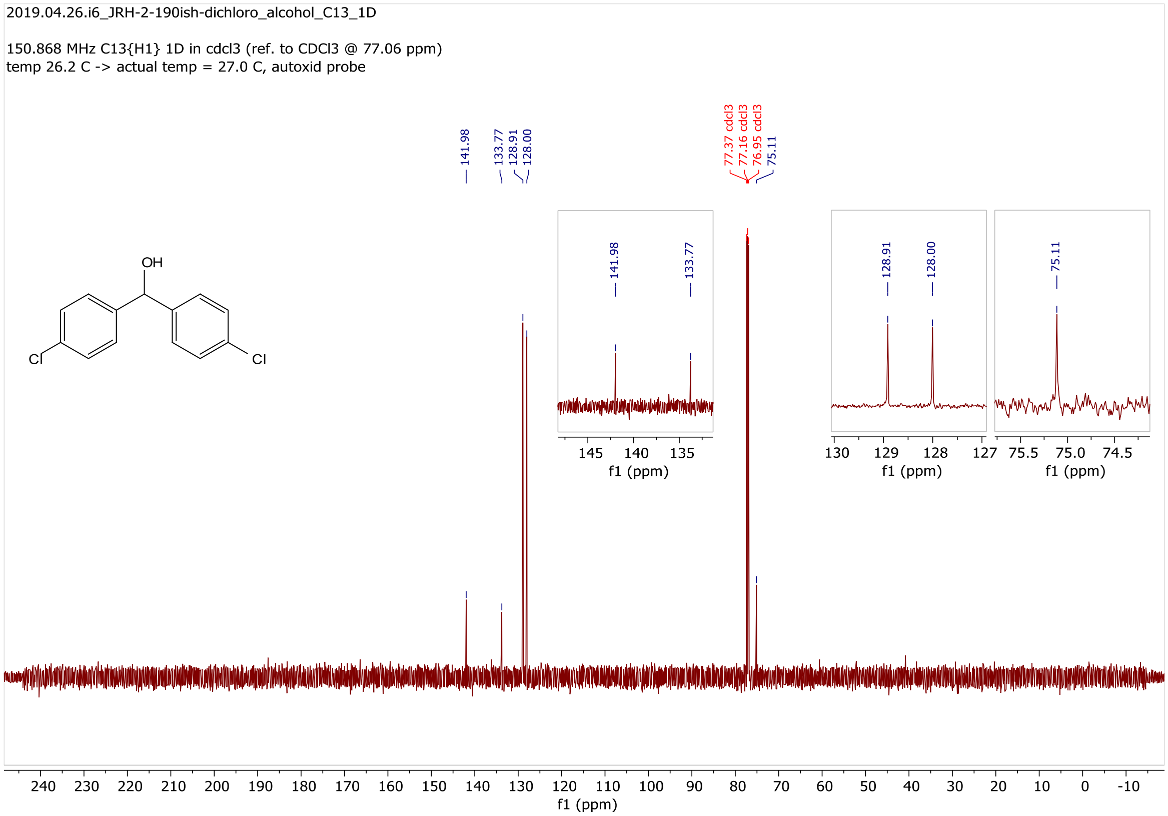

^{1}H (600 MHz) and ^{13}C (126 MHz) NMR of compound 6e ($CDCl_3$)

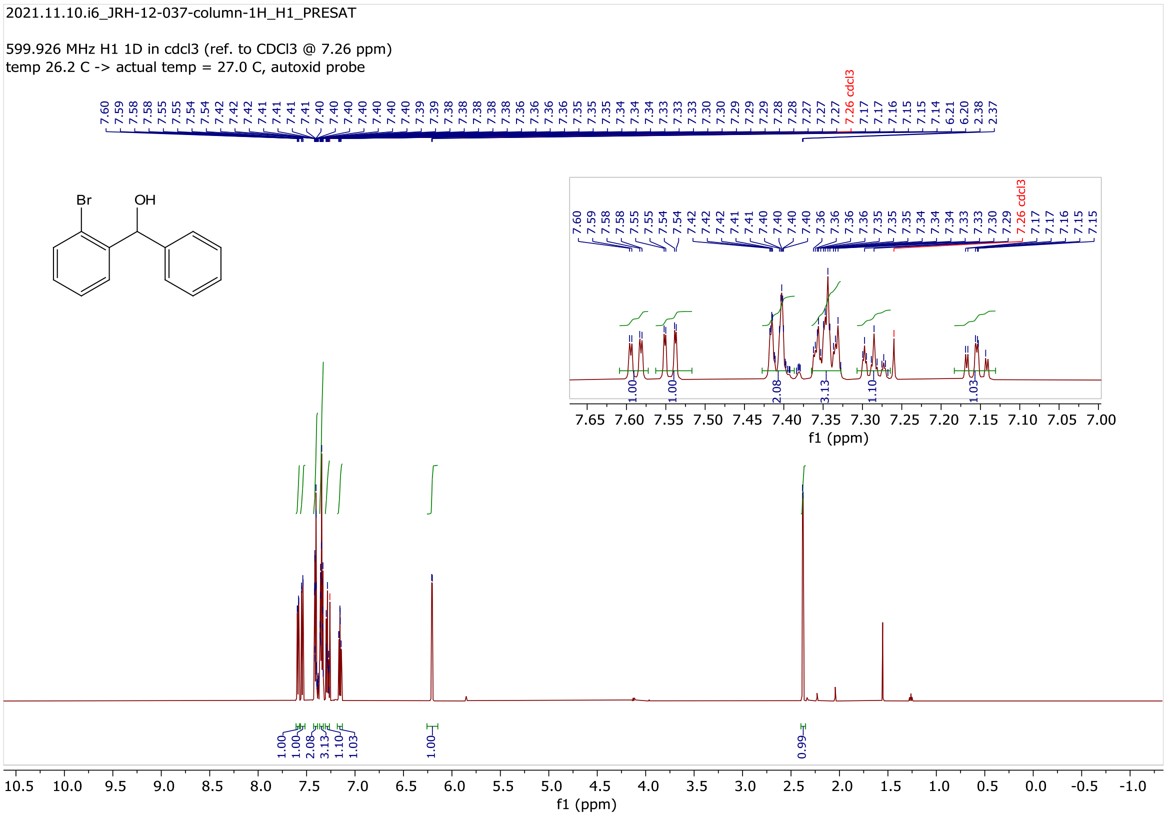

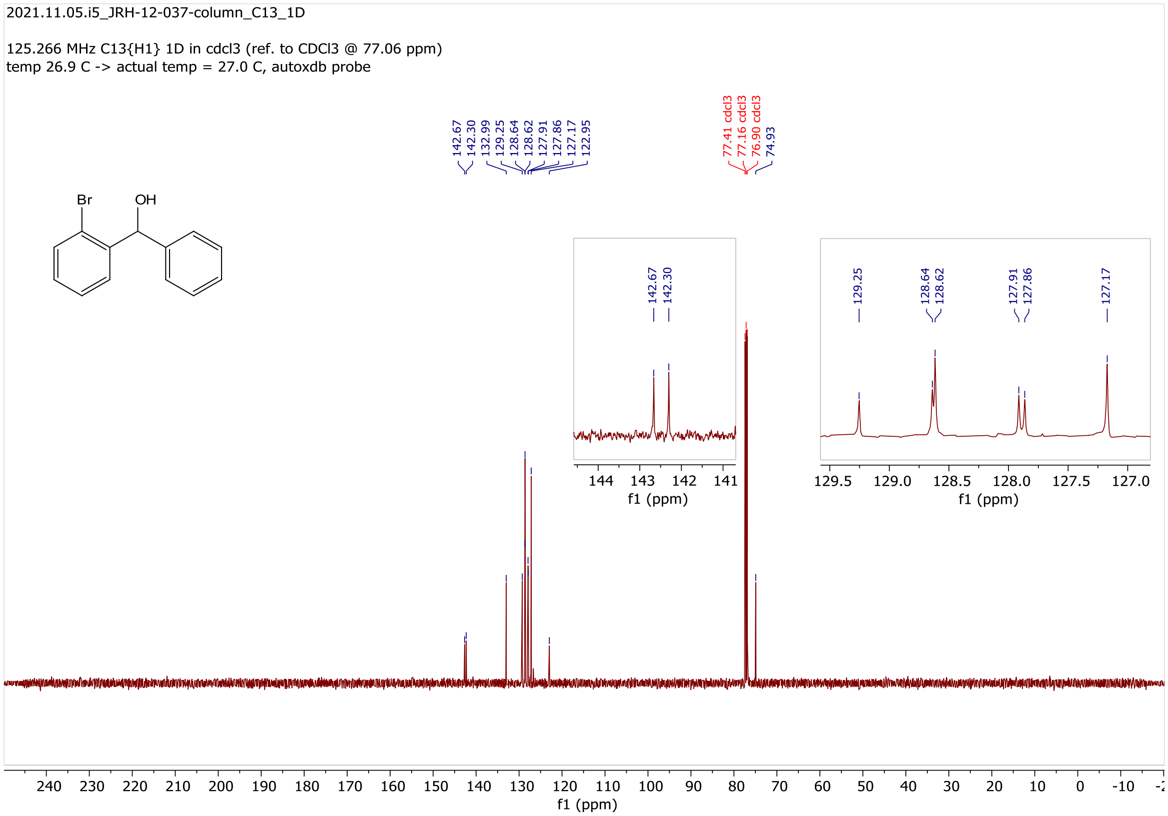

^{1}H (600 MHz) and ^{13}C (151 MHz) NMR of compound 6f ($CDCl_3$)

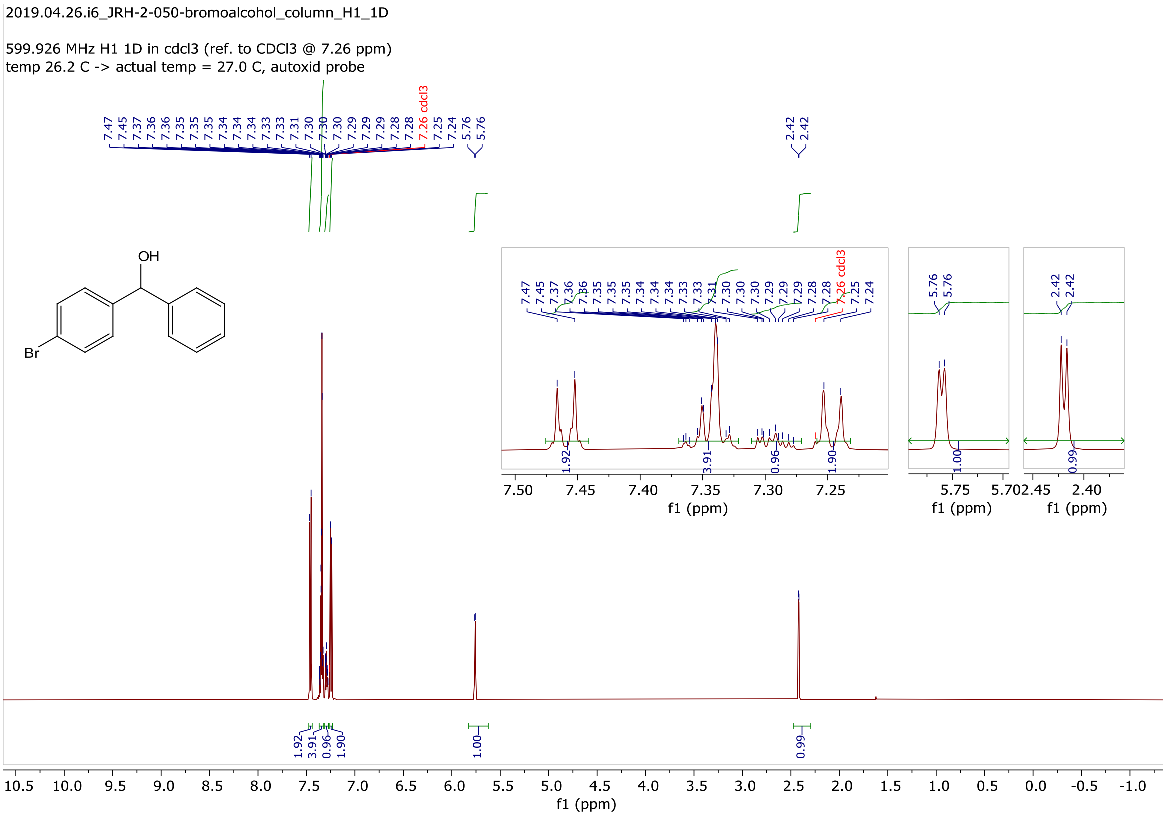

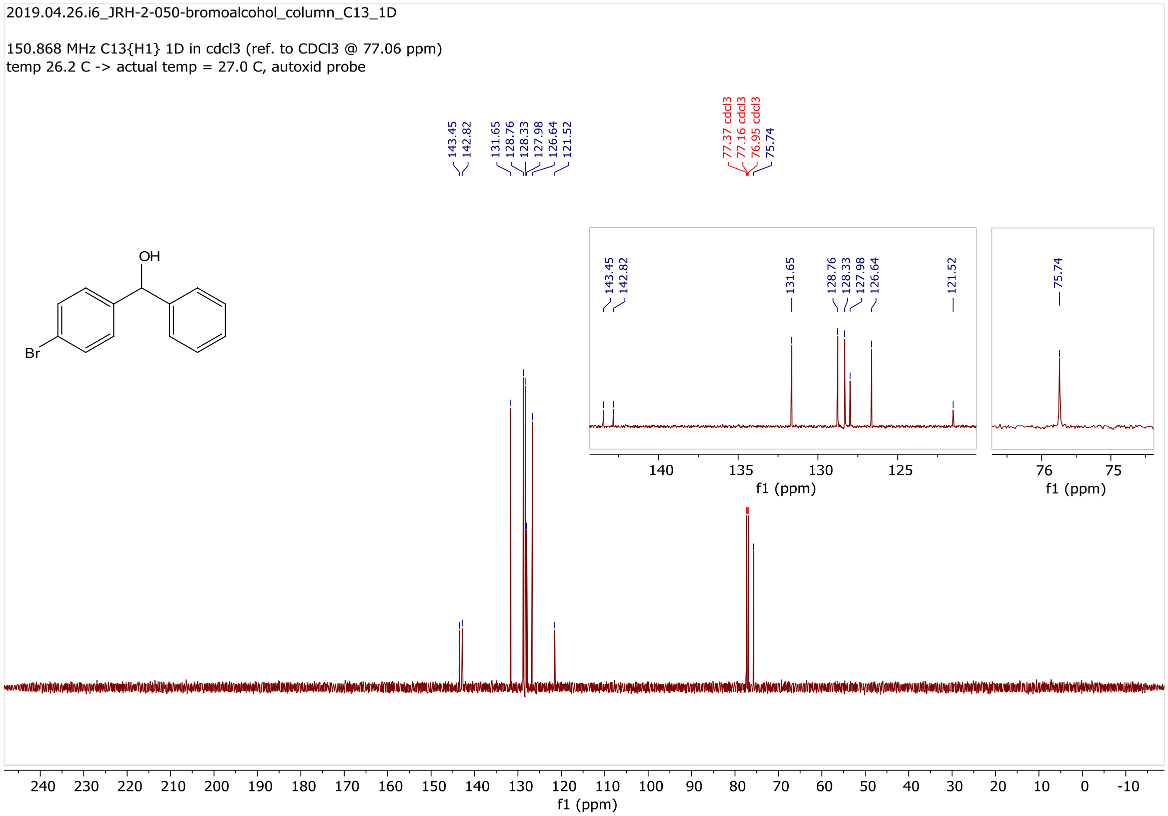

^1H (600 MHz) and ^{13}C (151 MHz) NMR of compound 6g ($CDCl_3$)

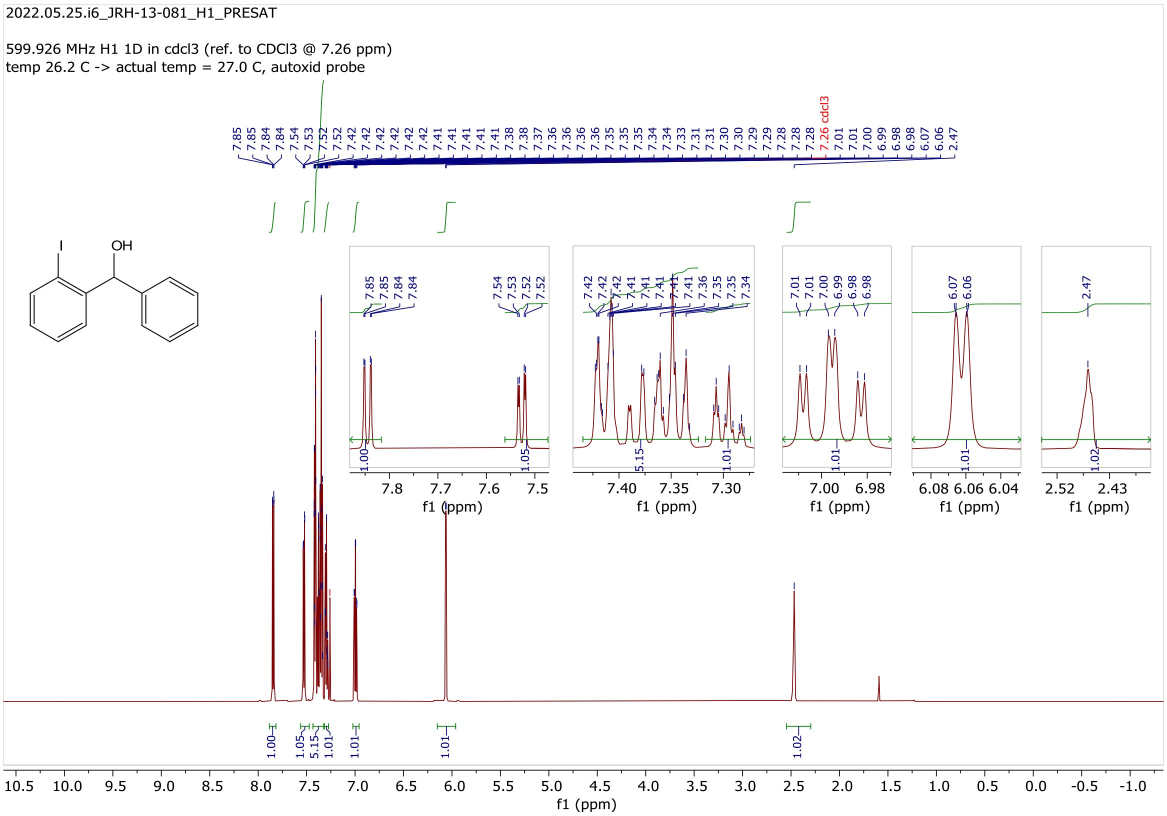

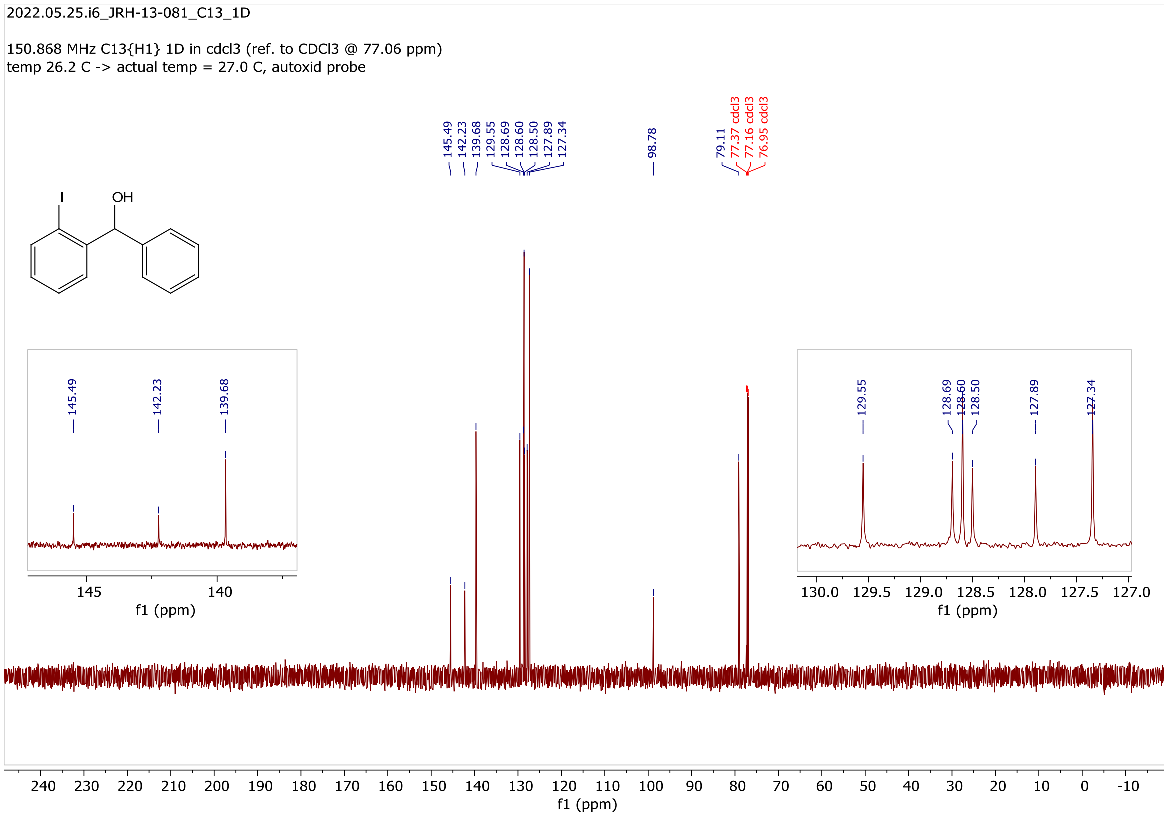

^{1}H (600 MHz) and ^{13}C (151 MHz) NMR of compound 6h

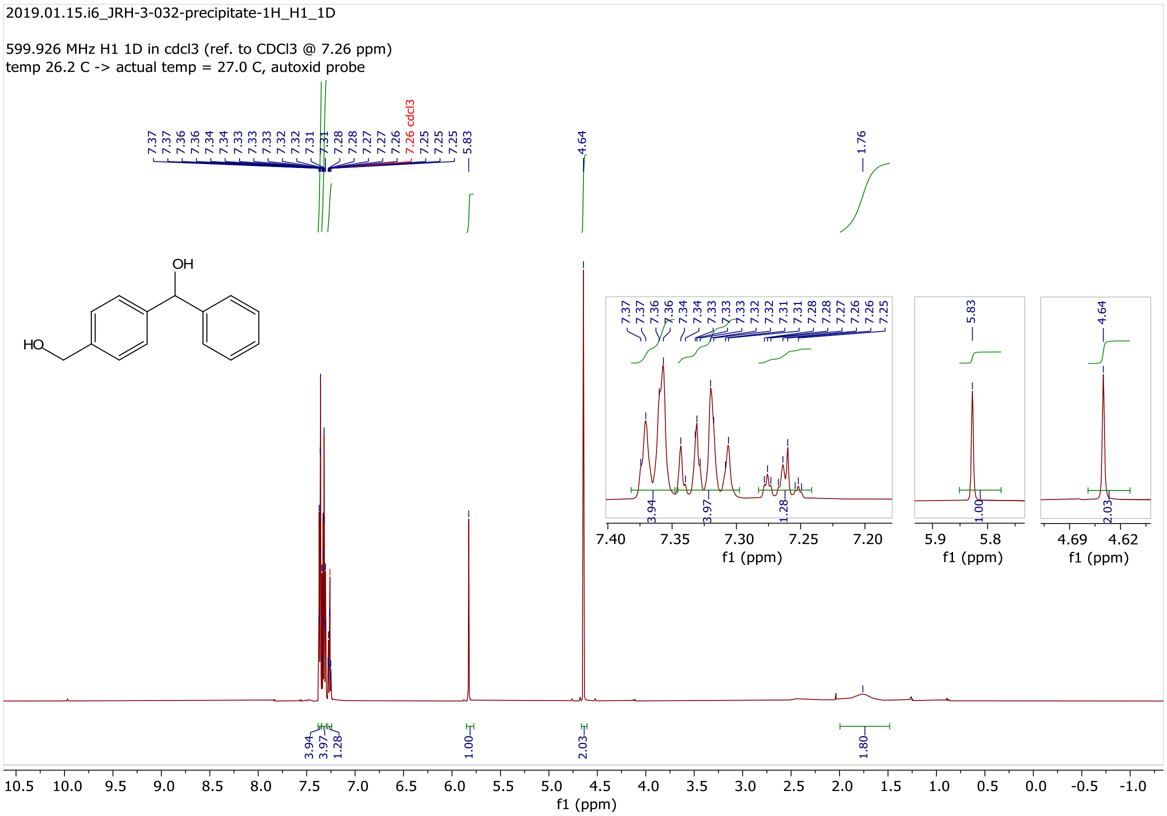

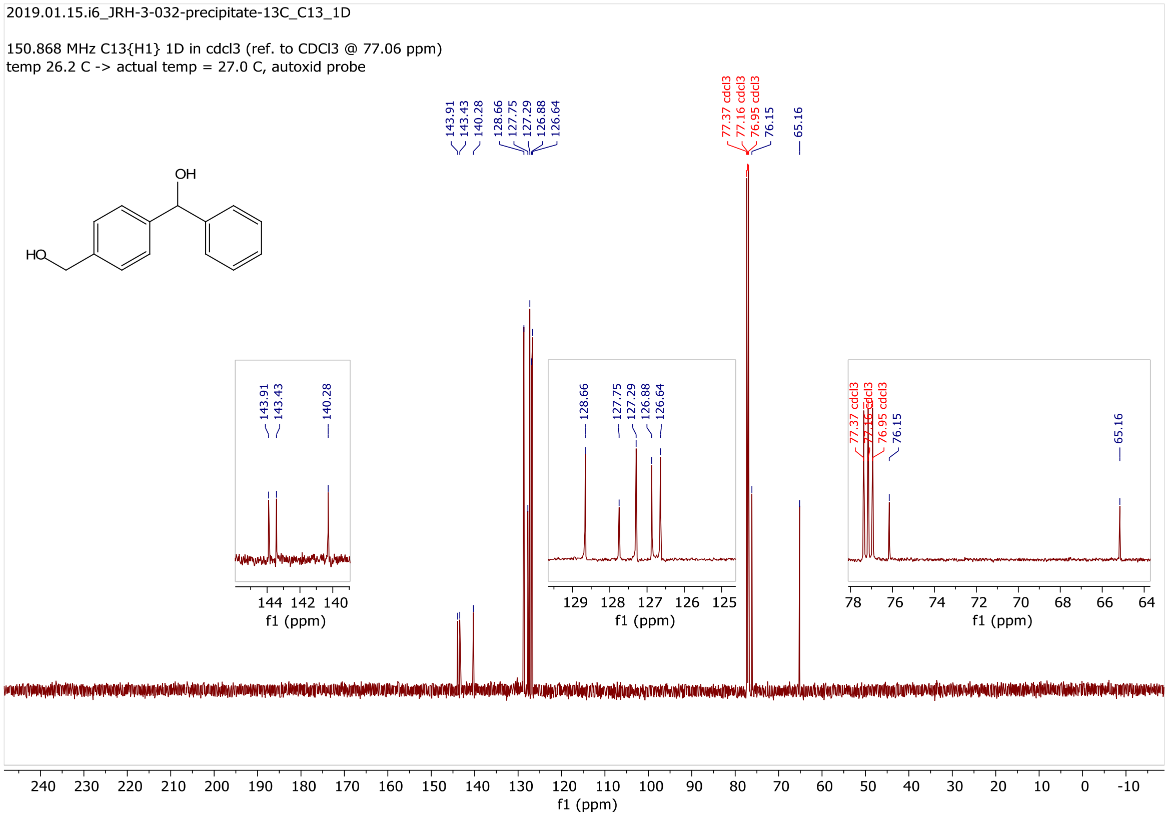

^{1}H (500 MHz) and ^{13}C (126 MHz) NMR of compound 6i ($CDCl_3$)

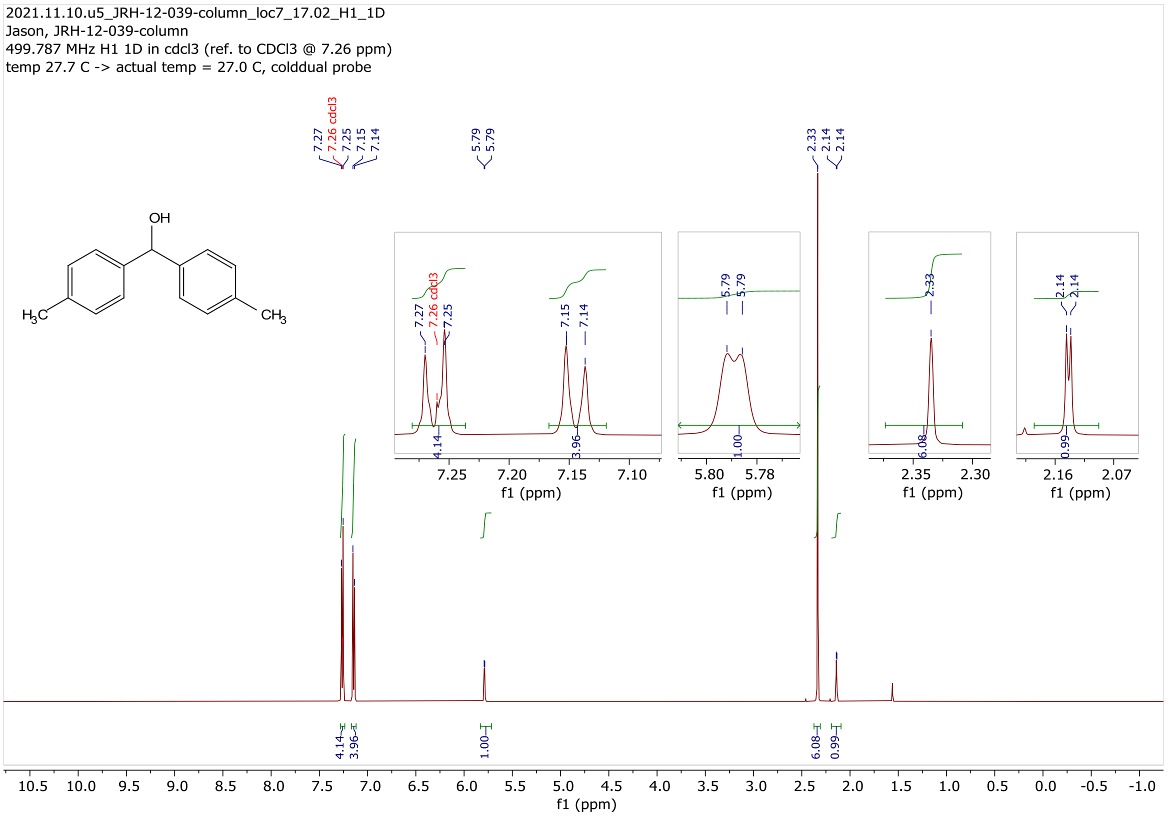

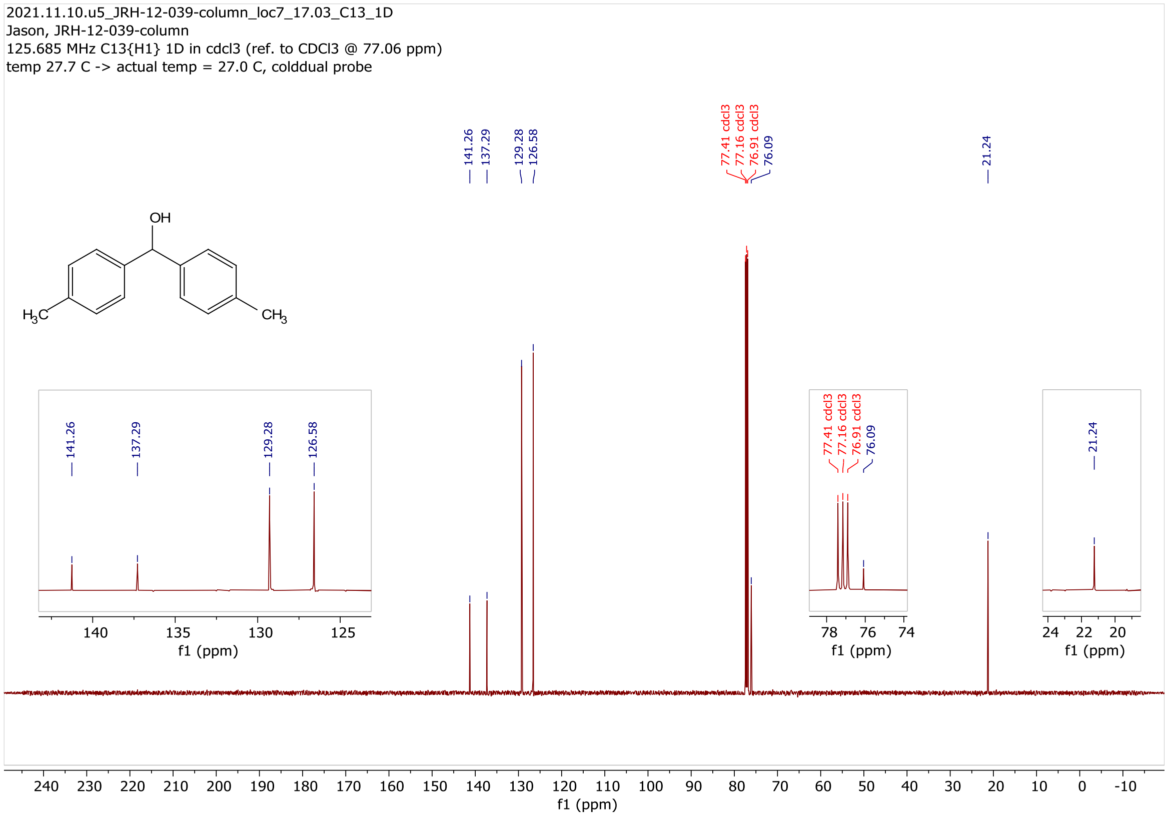

^{1}H (500 MHz) and ^{13}C (126 MHz) NMR of compound 6j ($CDCl_3$)

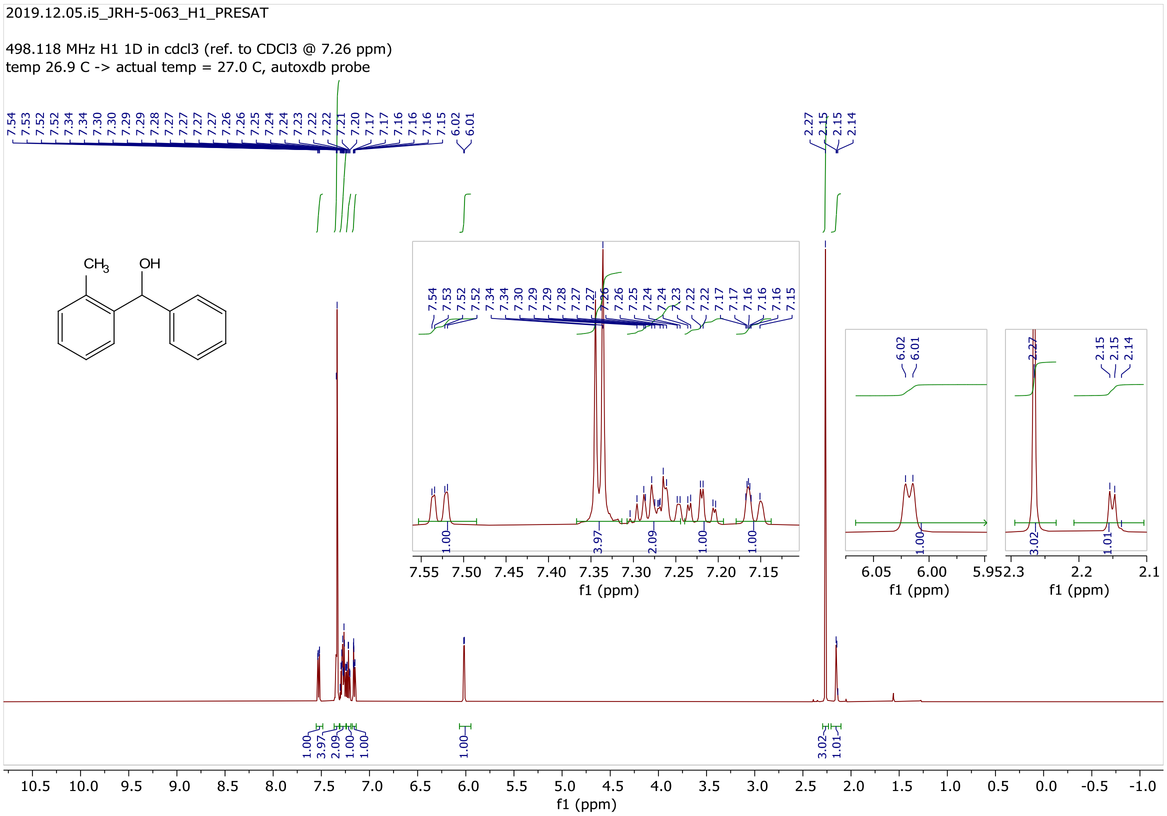

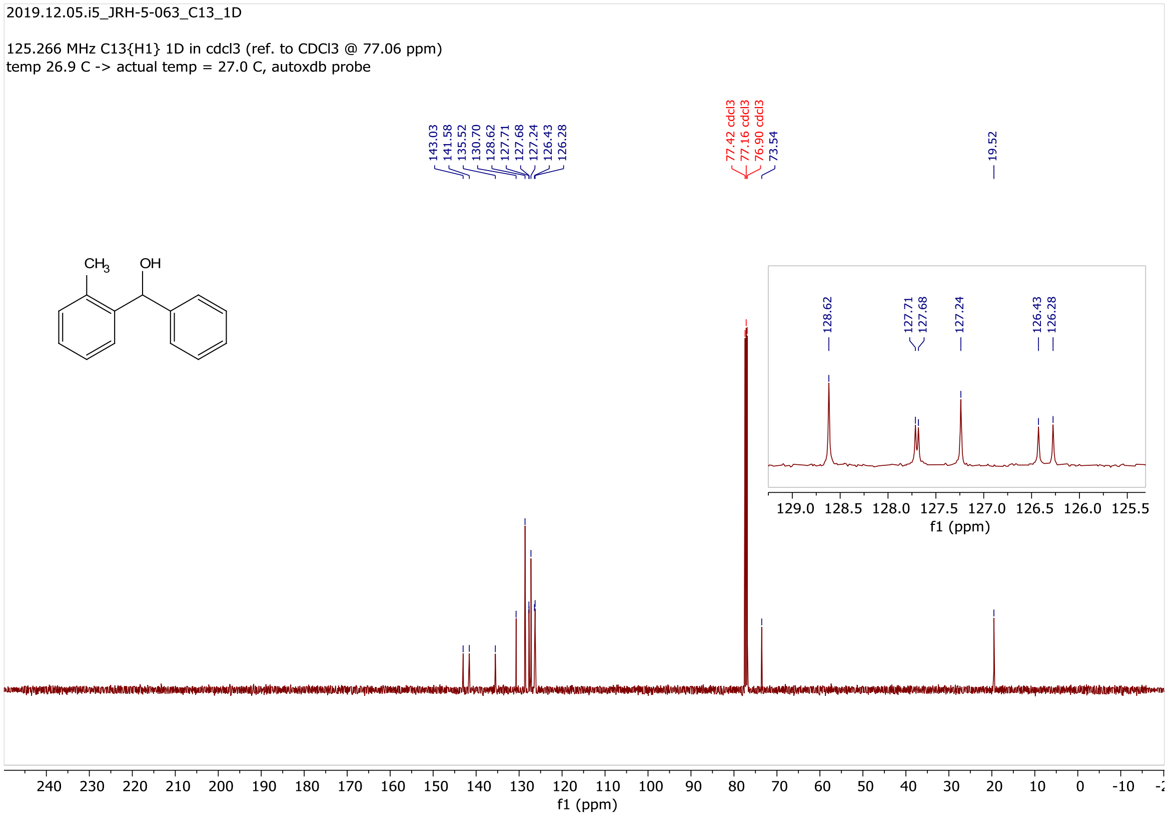

^{1}H (500 MHz) and ^{13}C (126 MHz) NMR of compound 6k ($CDCl_3$)

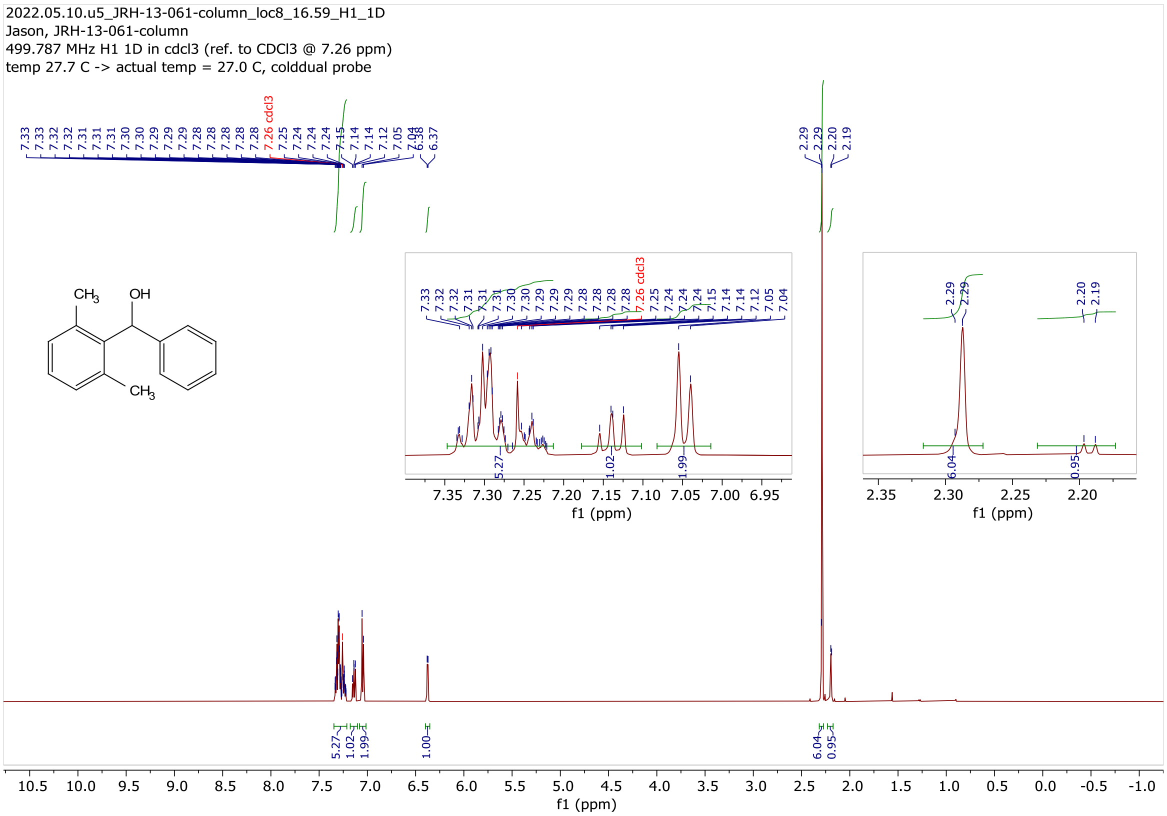

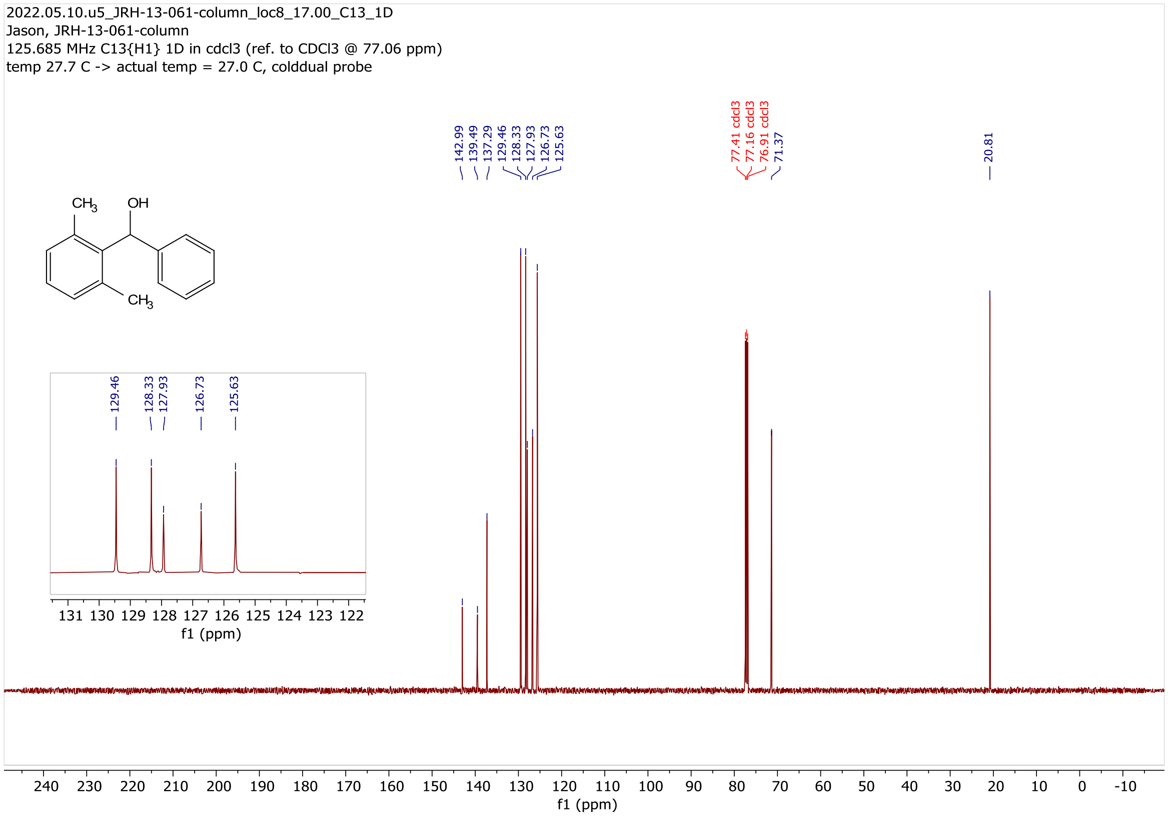

^{1}H (600 MHz) and ^{13}C (151 MHz) NMR of compound 6l ($CDCl_3$)

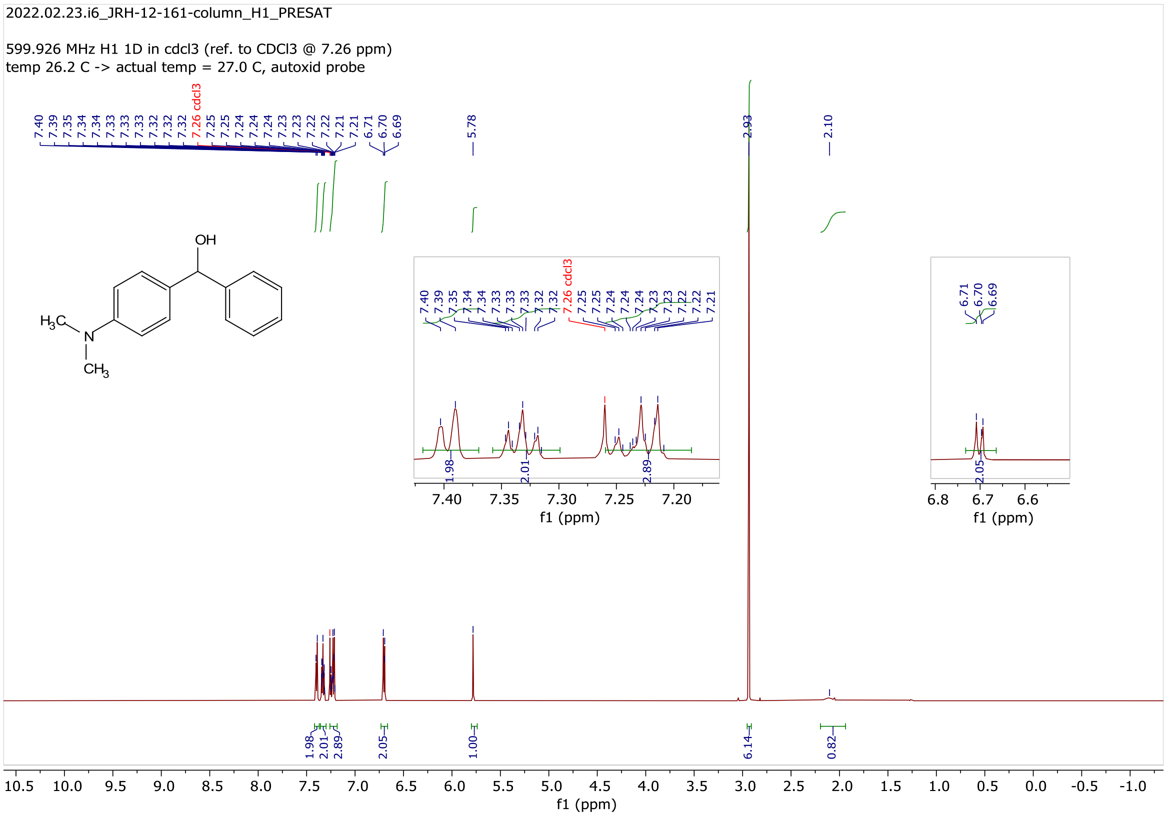

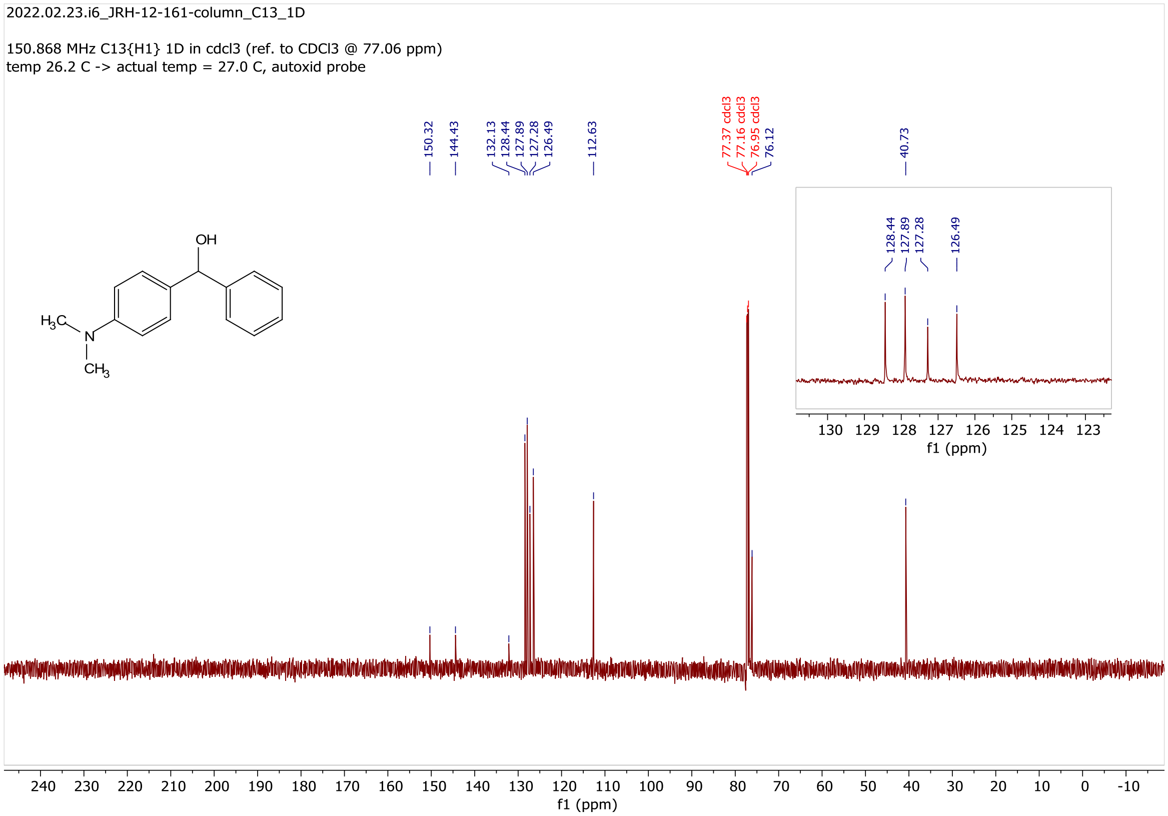

^{1}H (500 MHz) and ^{13}C (126 MHz) NMR of compound 6m ($CDCl_3$)

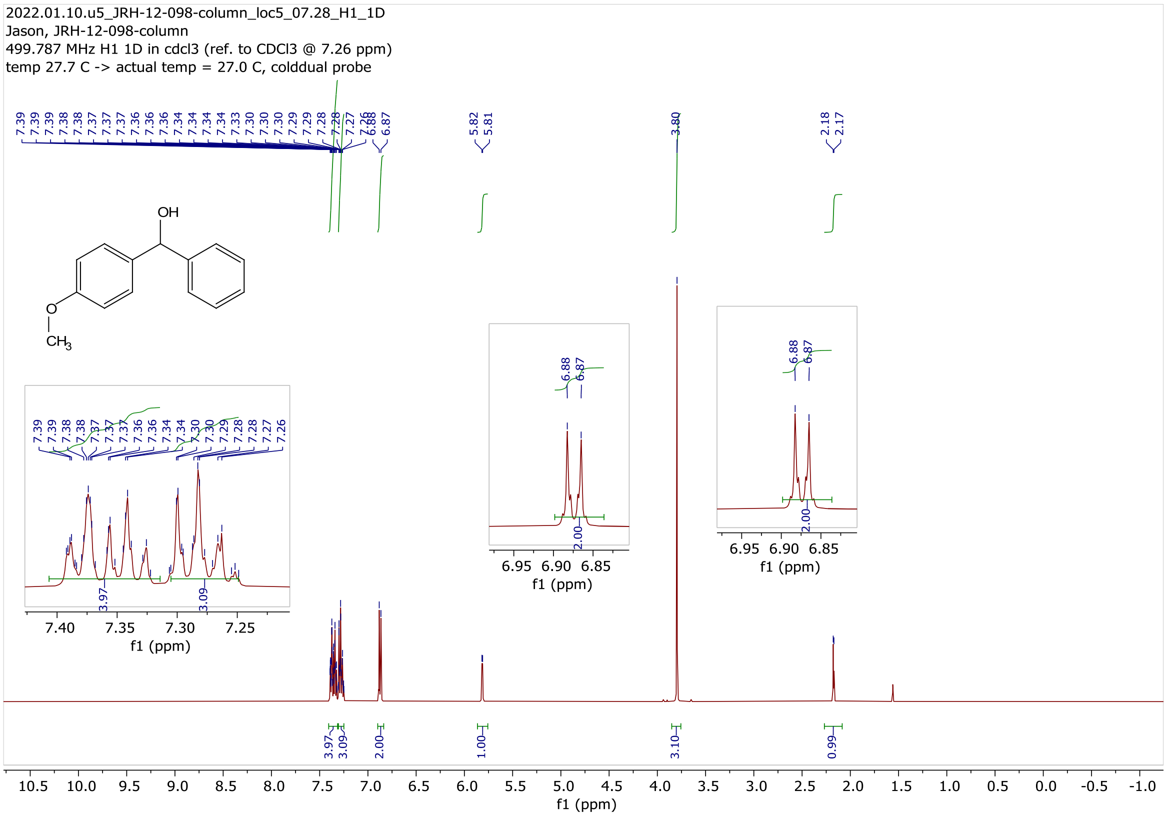

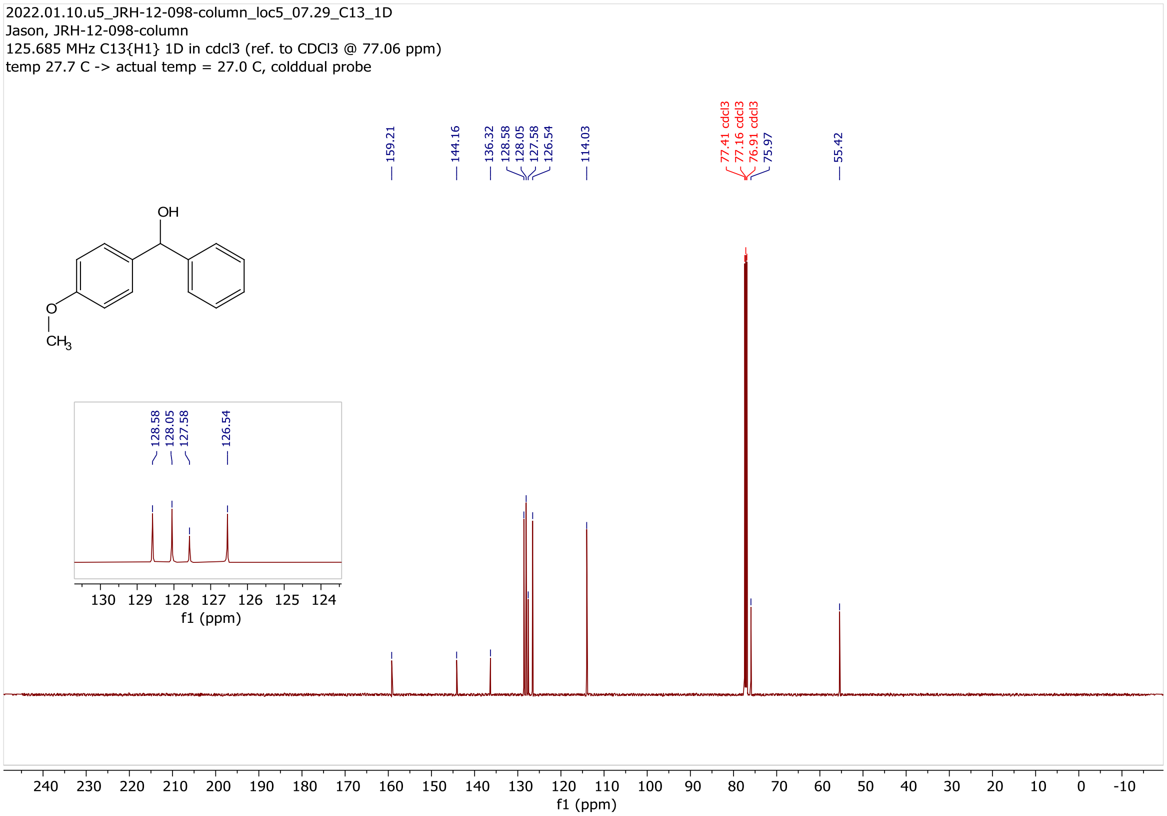

^{1}H (500 MHz) and ^{13}C (126 MHz) NMR of compound 6n ($CDCl_3$)

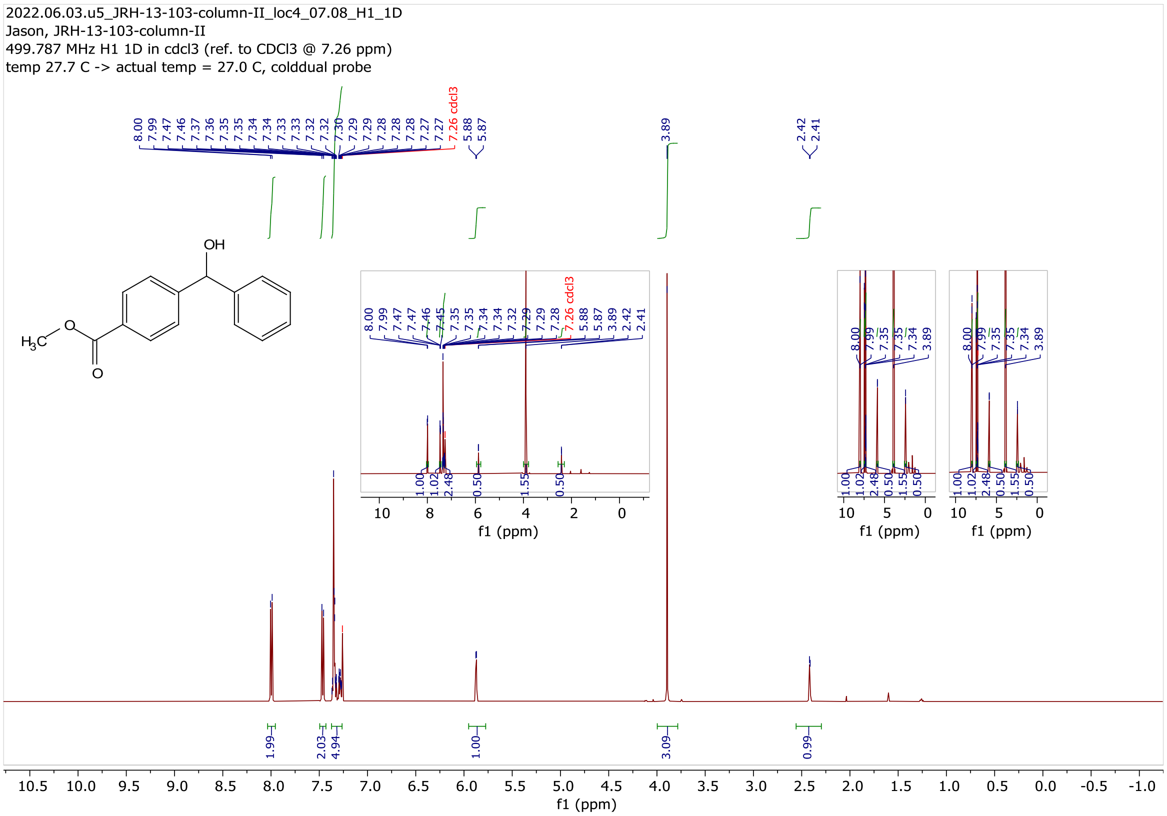

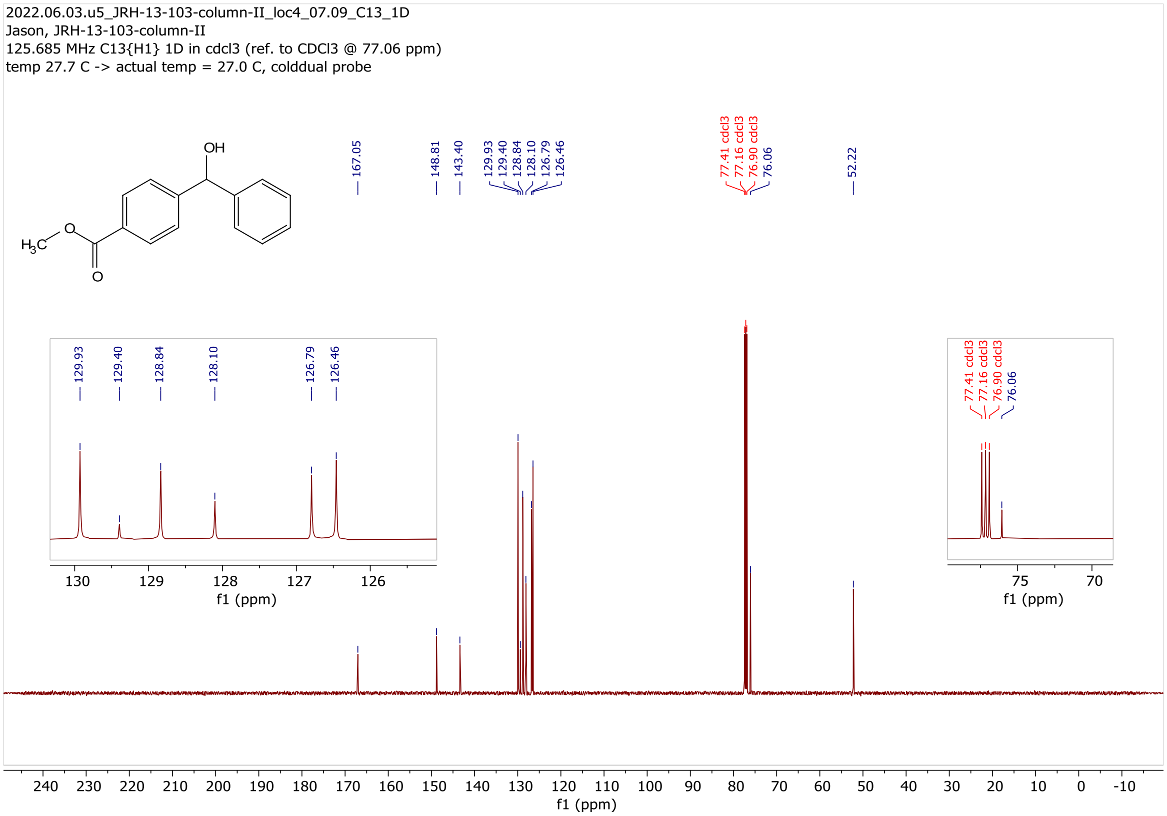

^{1}H (500 MHz) and ^{13}C (126 MHz) NMR of compound 6o ($CDCl_3$)

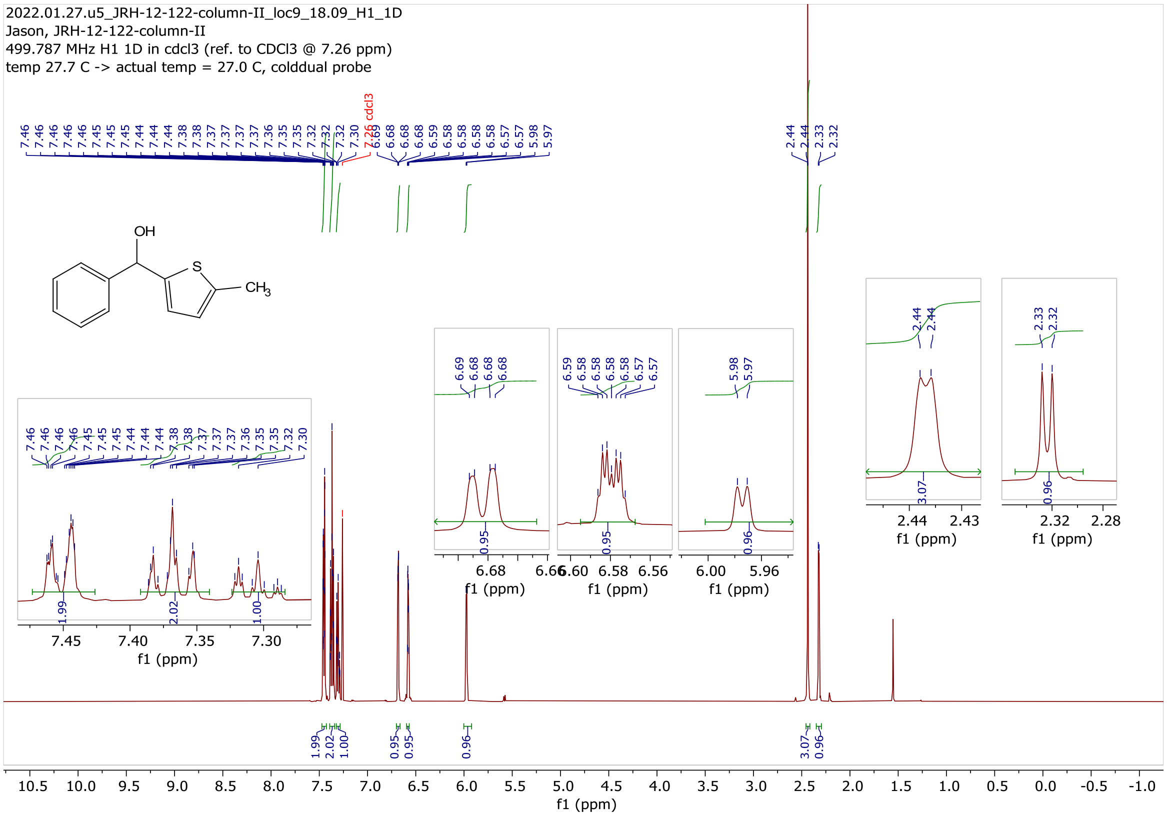

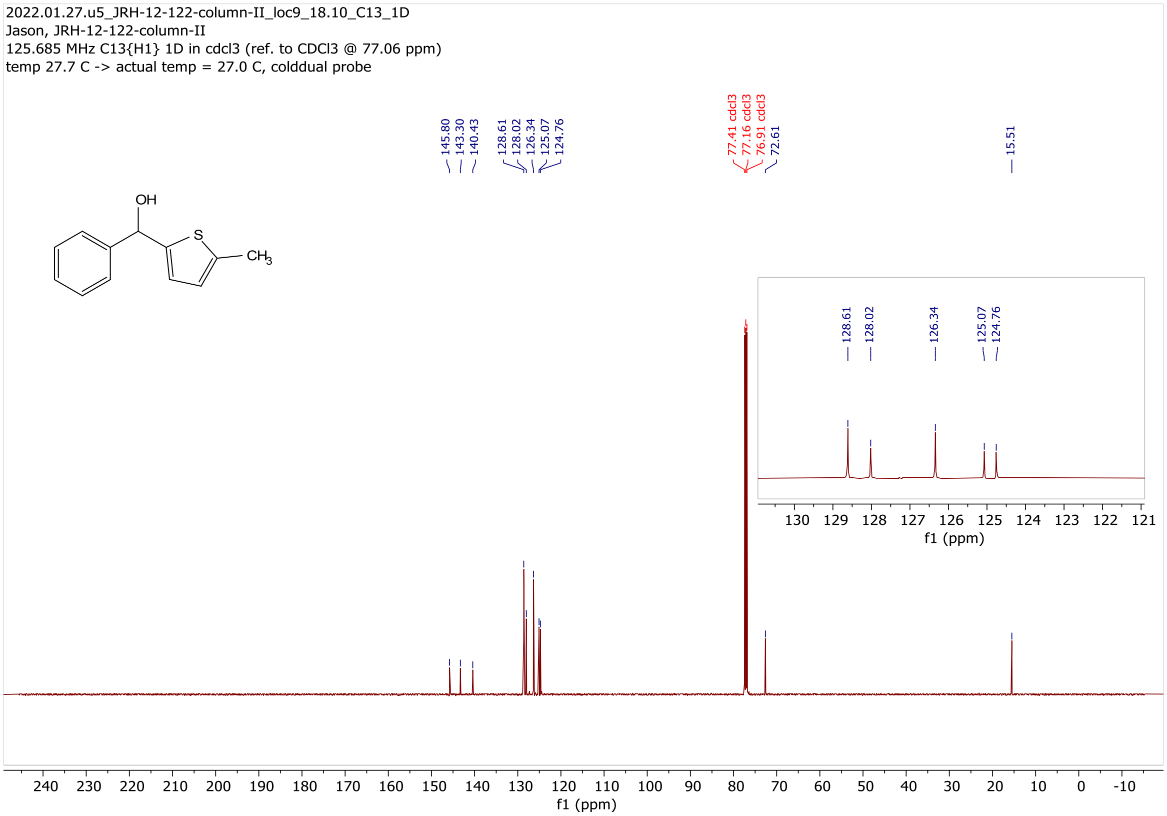

^{1}H (500 MHz) and ^{13}C (126 MHz) NMR of compound 6q ($CDCl_3$)

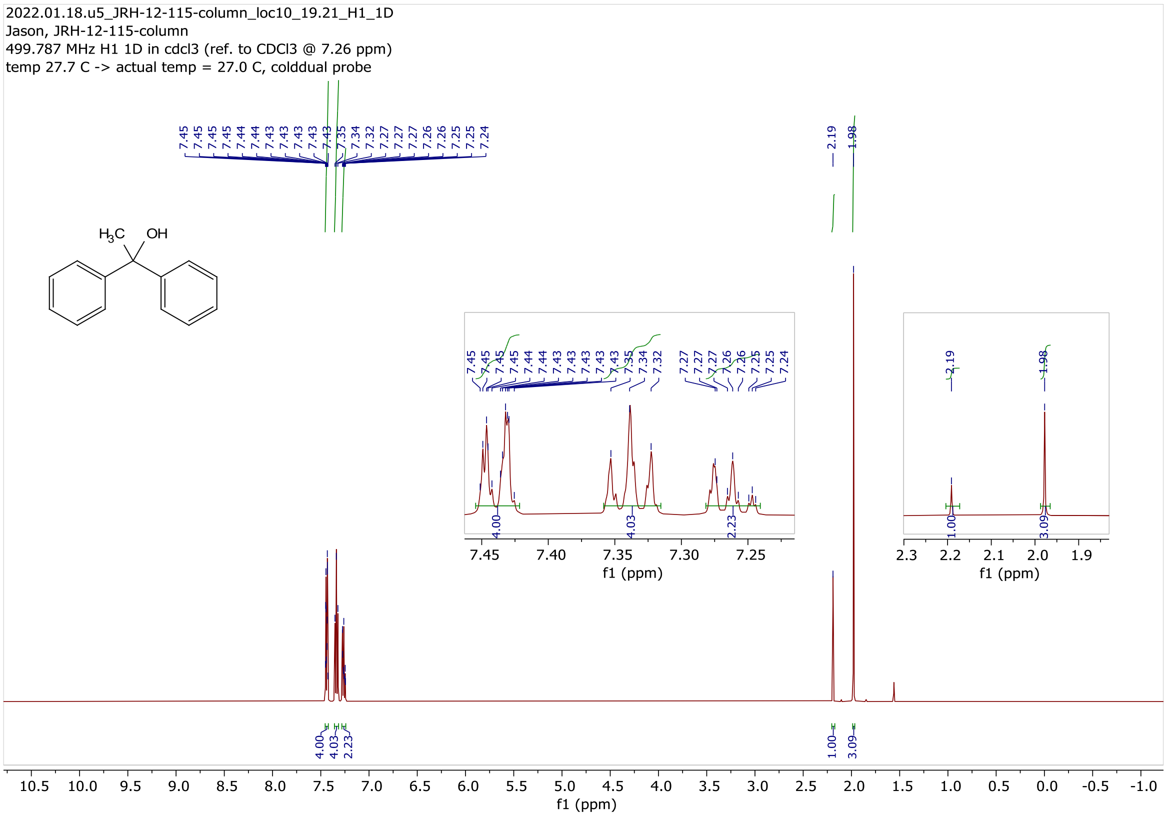

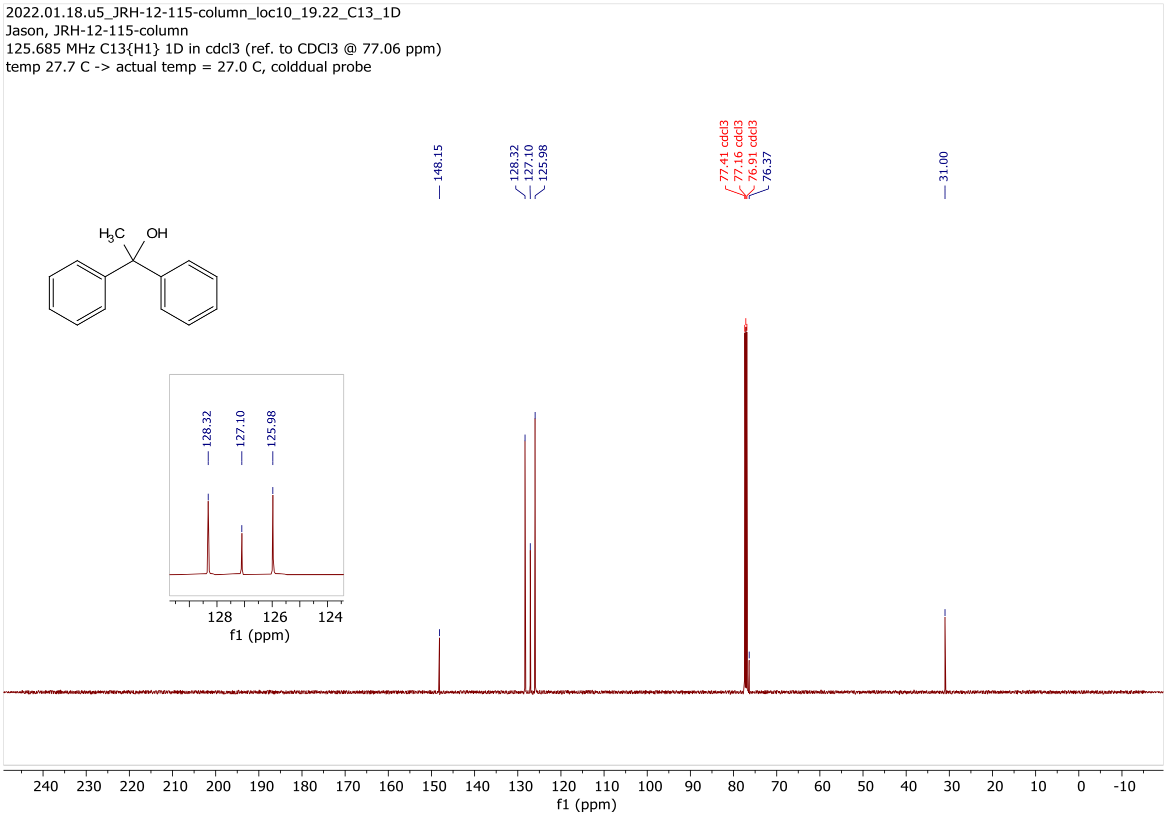

^{1}H (600 MHz) and ^{13}C (151 MHz) NMR of compound 6s ($CDCl_3$)

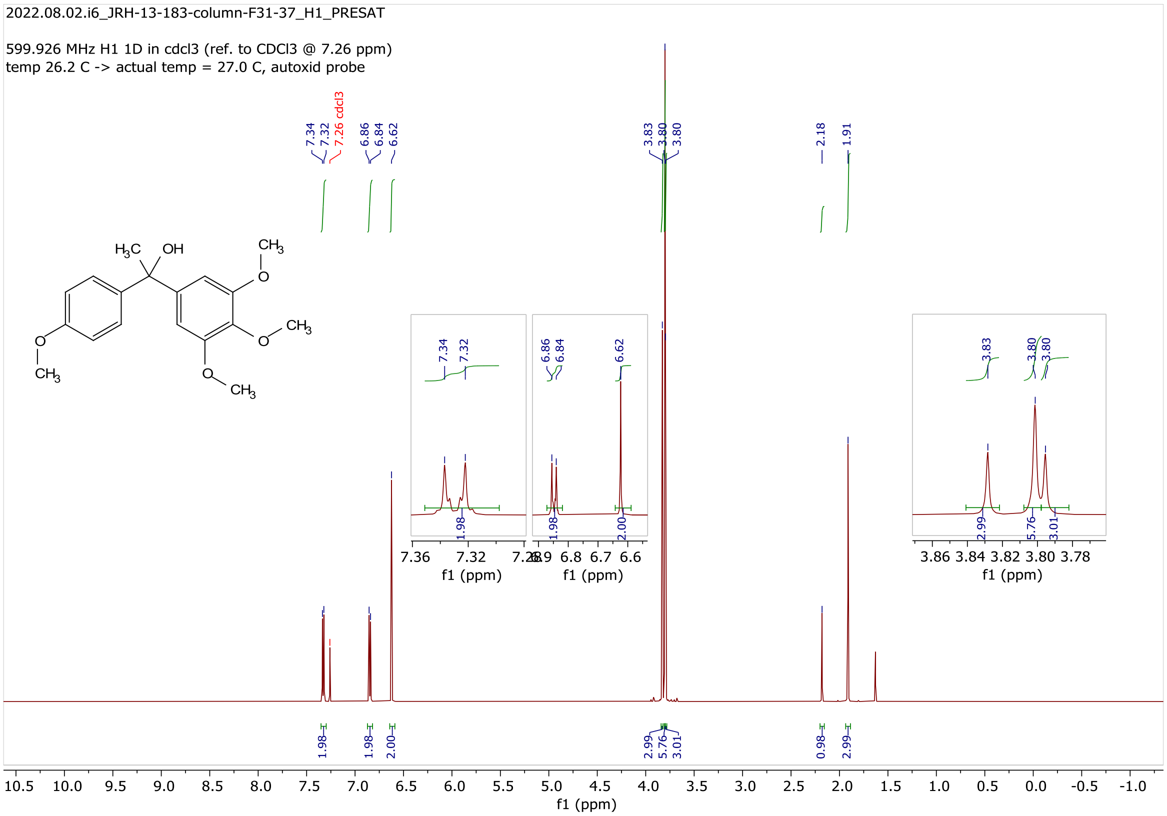

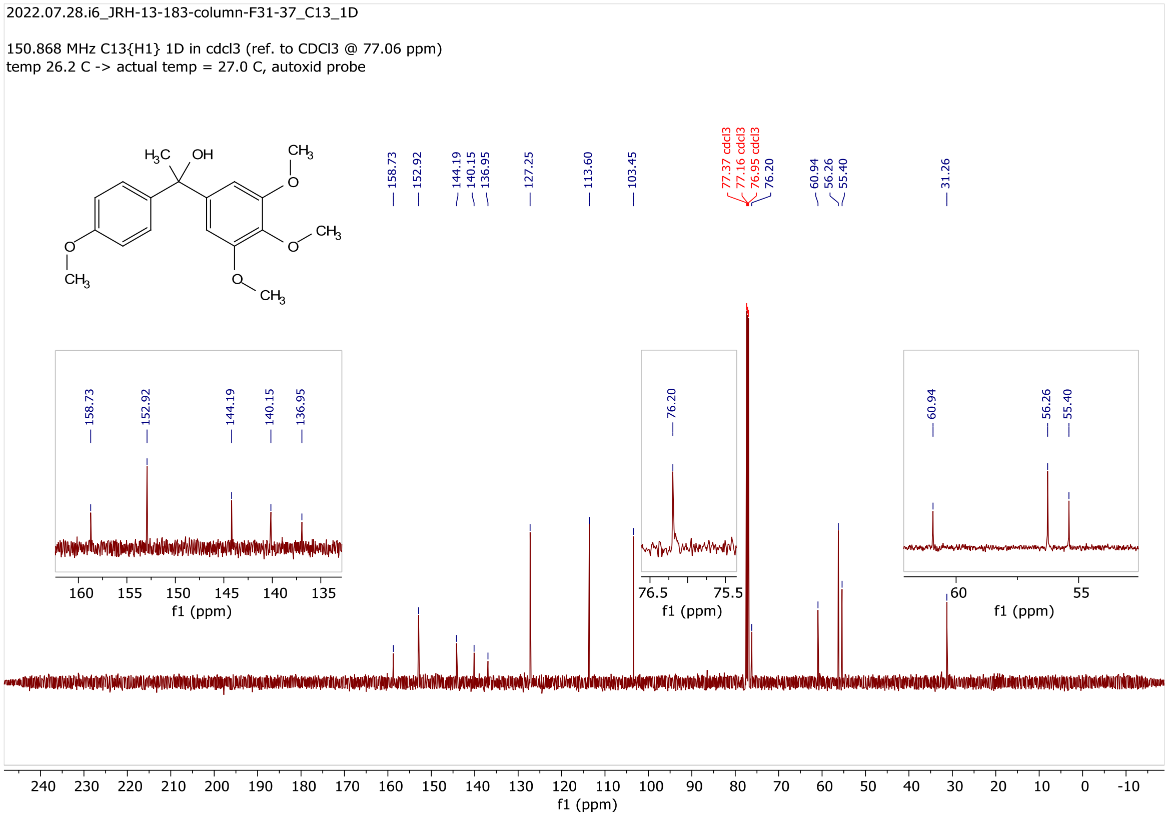

^{1}H (700 MHz) and ^{13}C (151 MHz) NMR of compound 6t ($CDCl_3$)

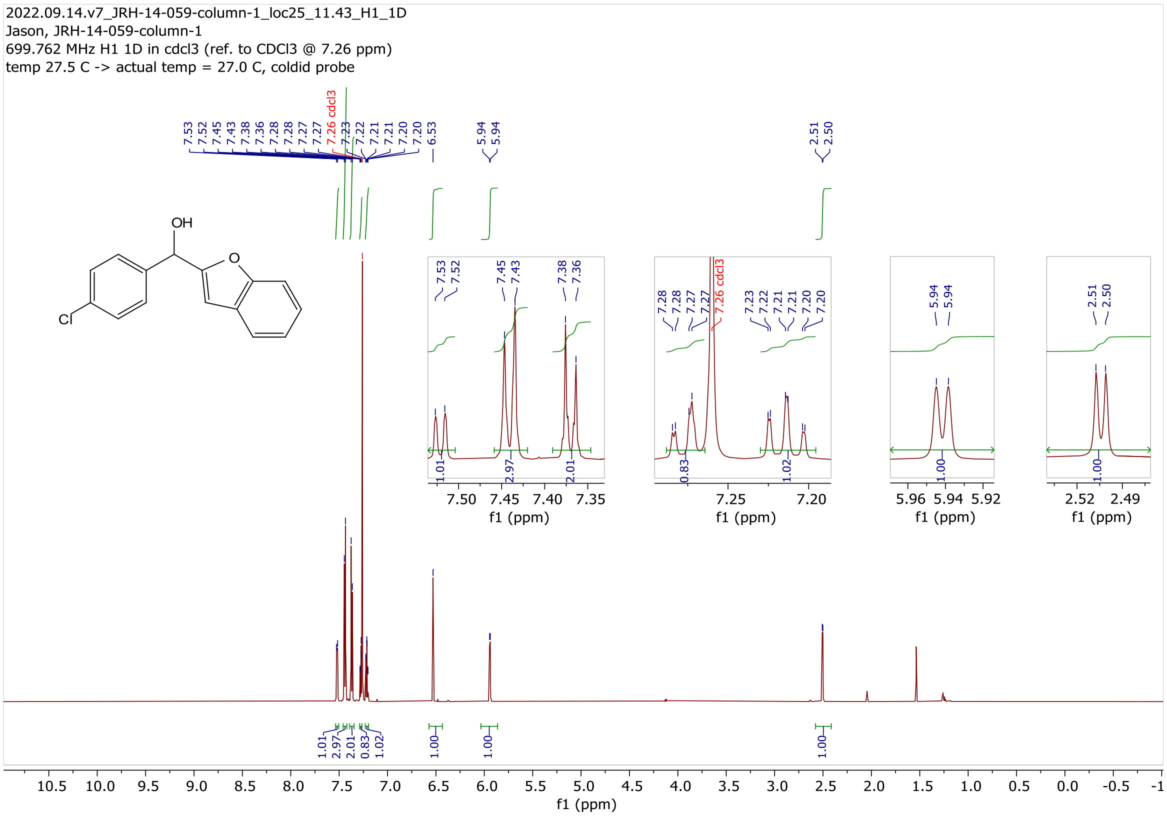

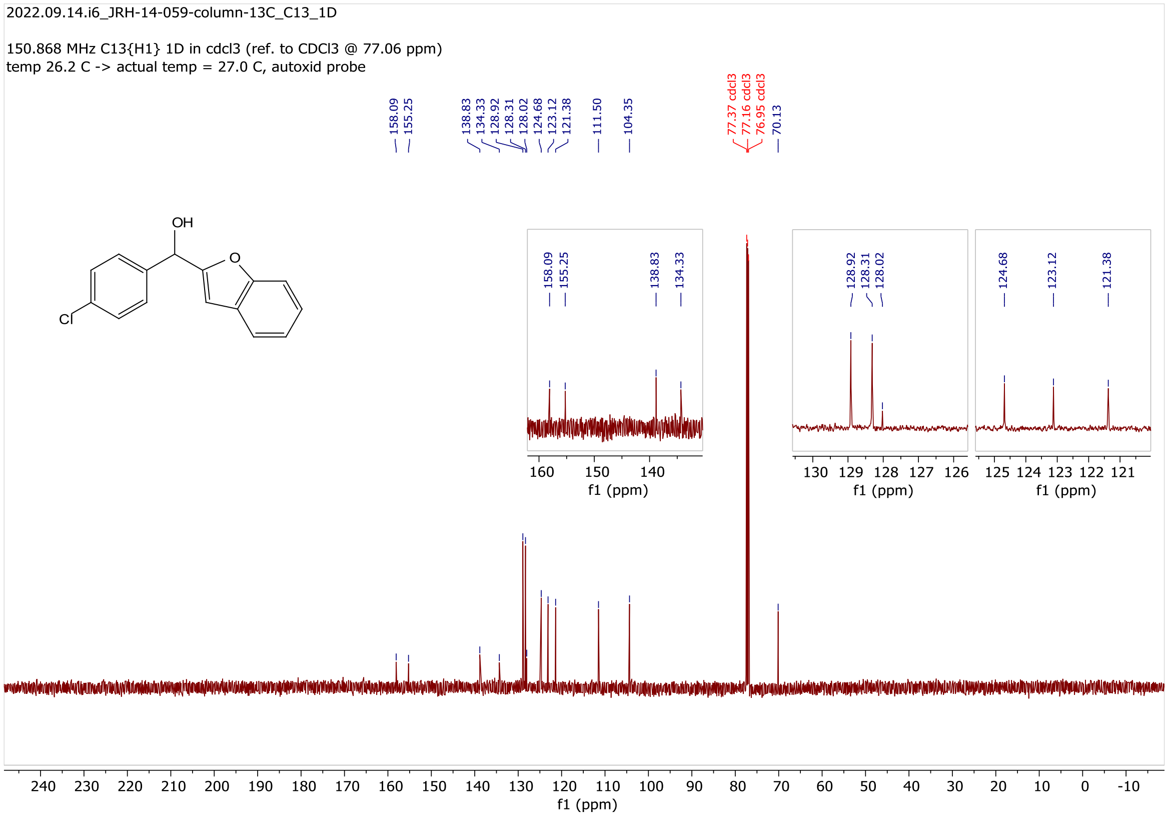

^{1}H (500 MHz) and ^{13}C (126 MHz) NMR of compound 6u ($CDCl_3$)

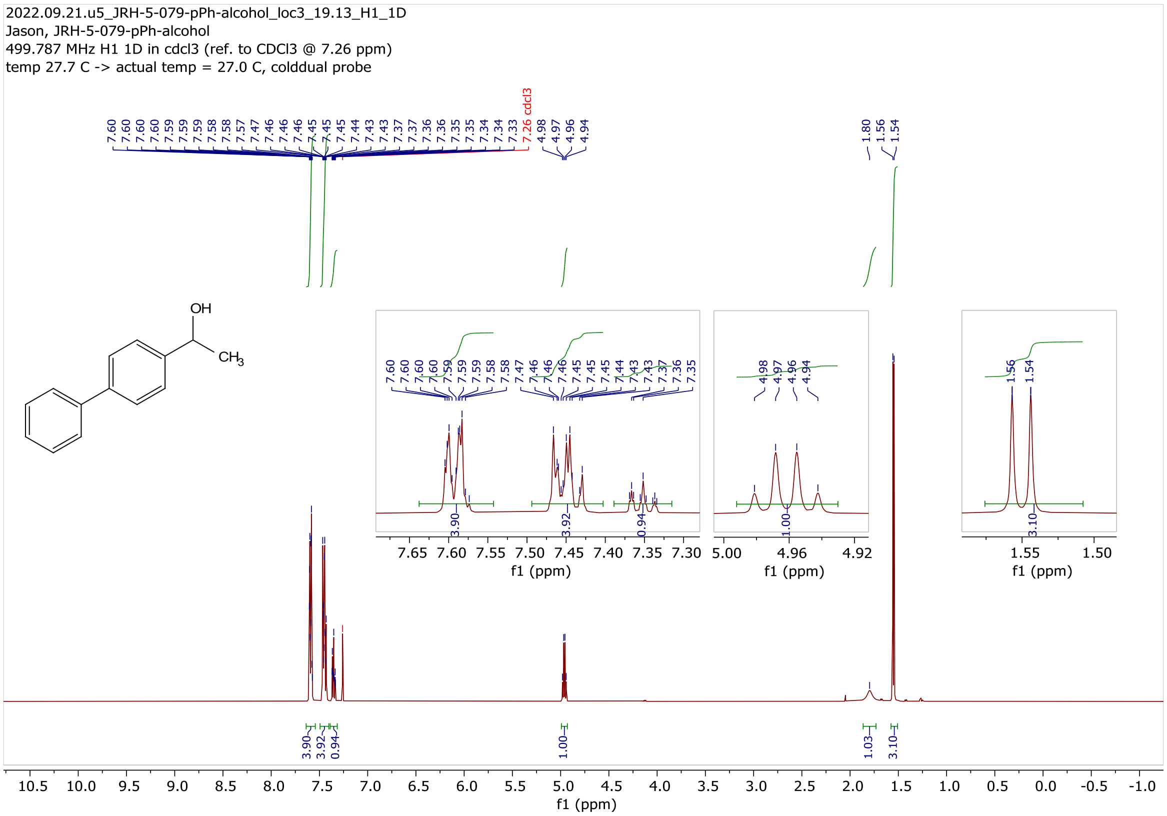

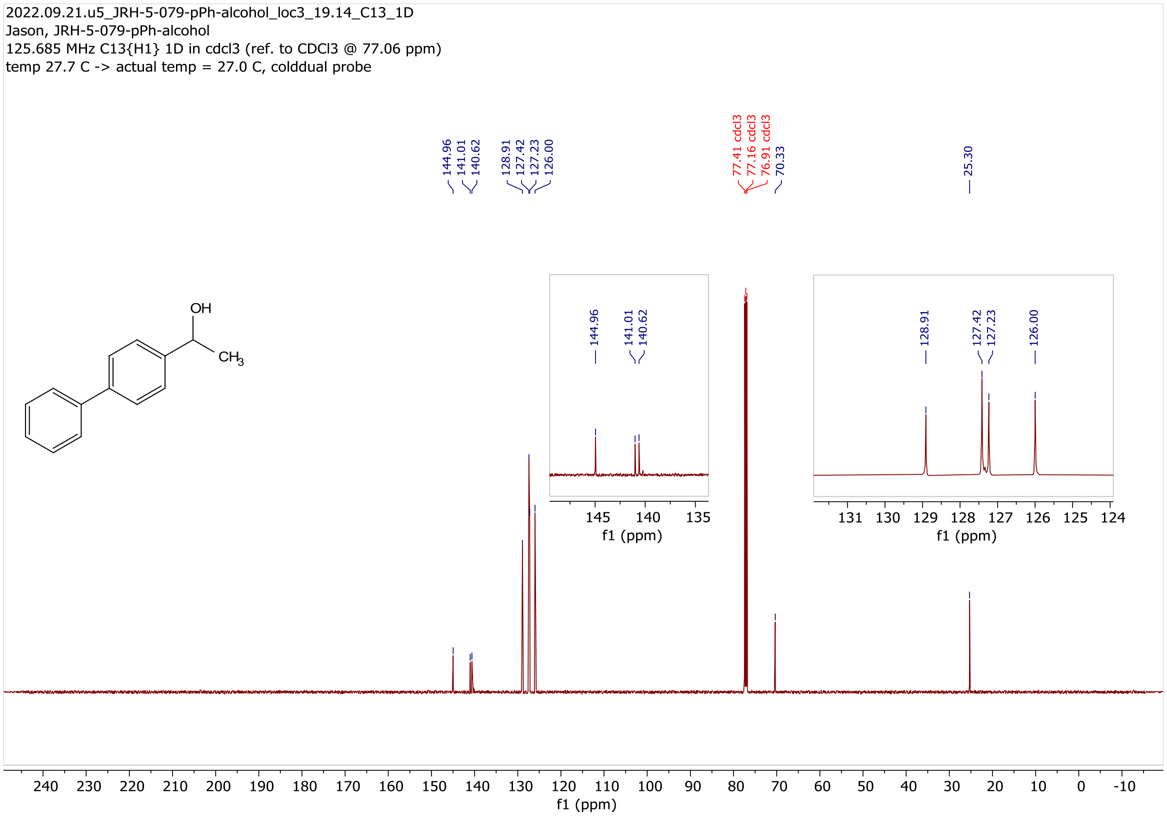

^{1}H (500 MHz) and ^{13}C (126 MHz) NMR of compound 6v ($CDCl_3$)

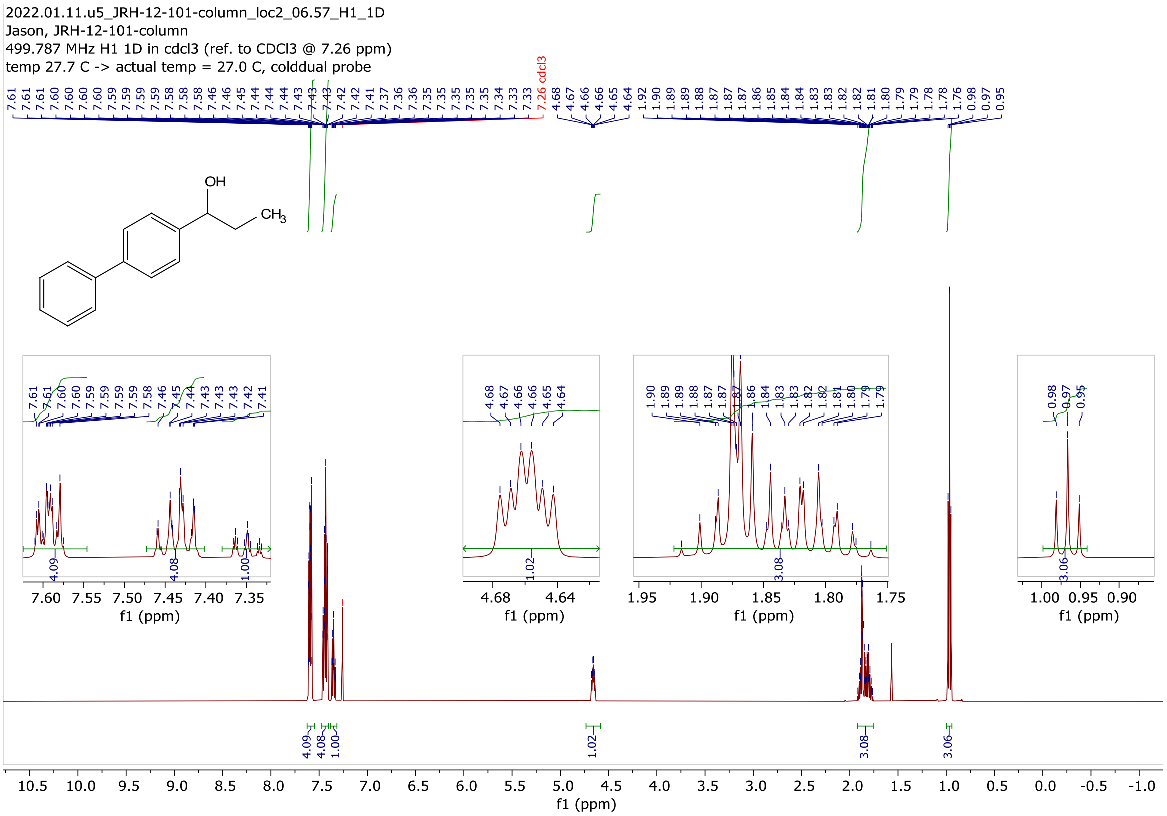

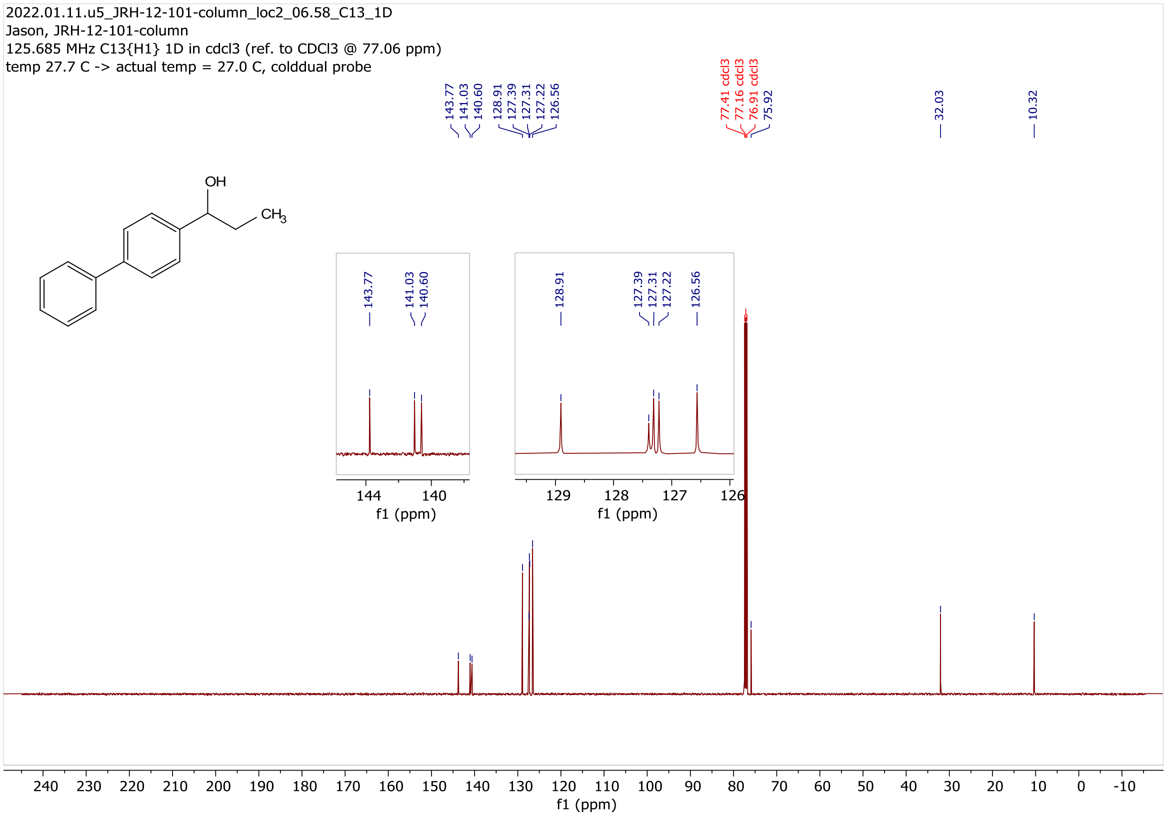

^{1}H (500 MHz) and ^{13}C (126 MHz) NMR of compound 6w ($CDCl_3$)

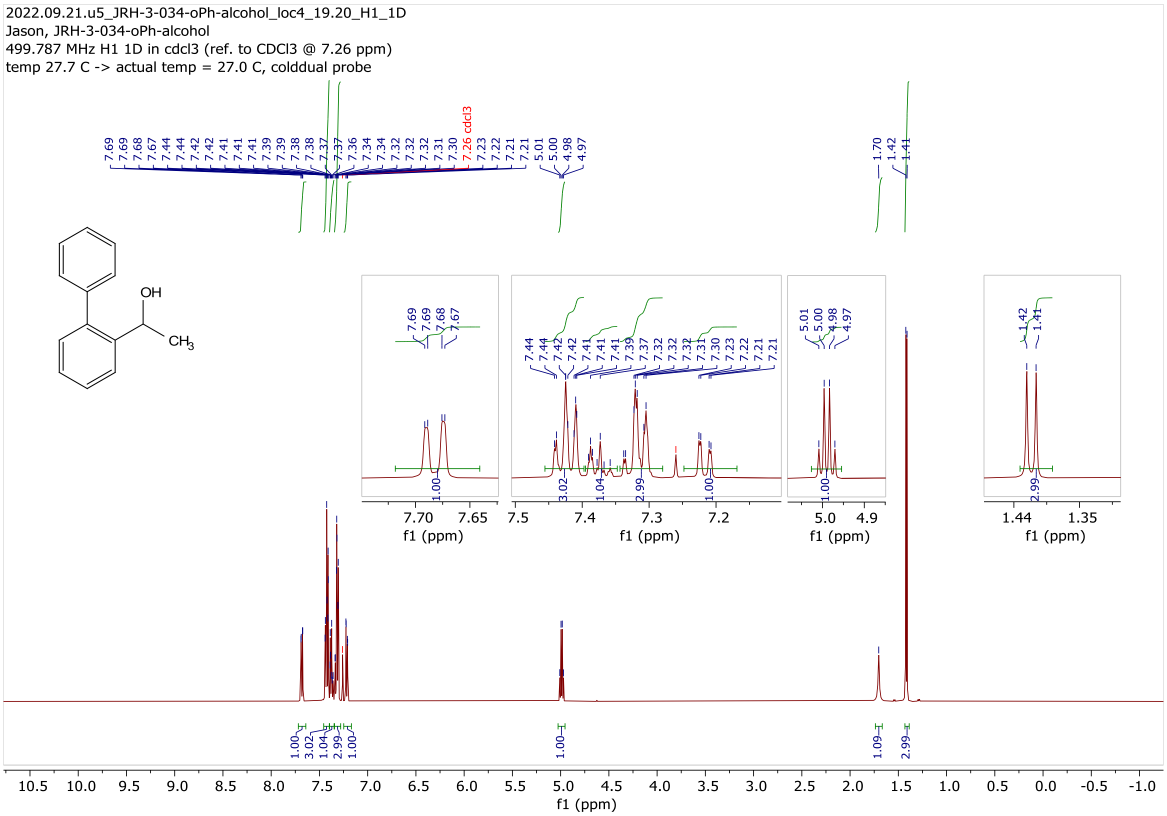

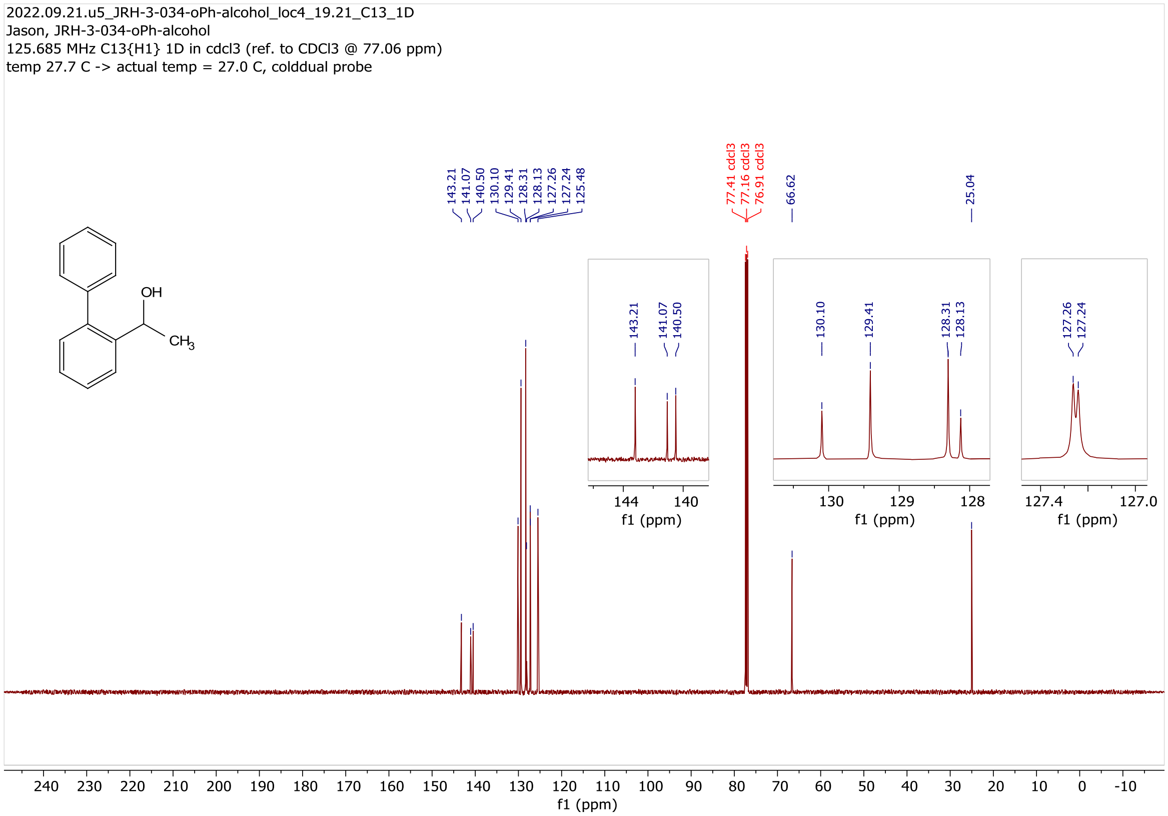

^{1}H (500 MHz) and ^{13}C (126 MHz) NMR of compound 6x ($CDCl_3$)

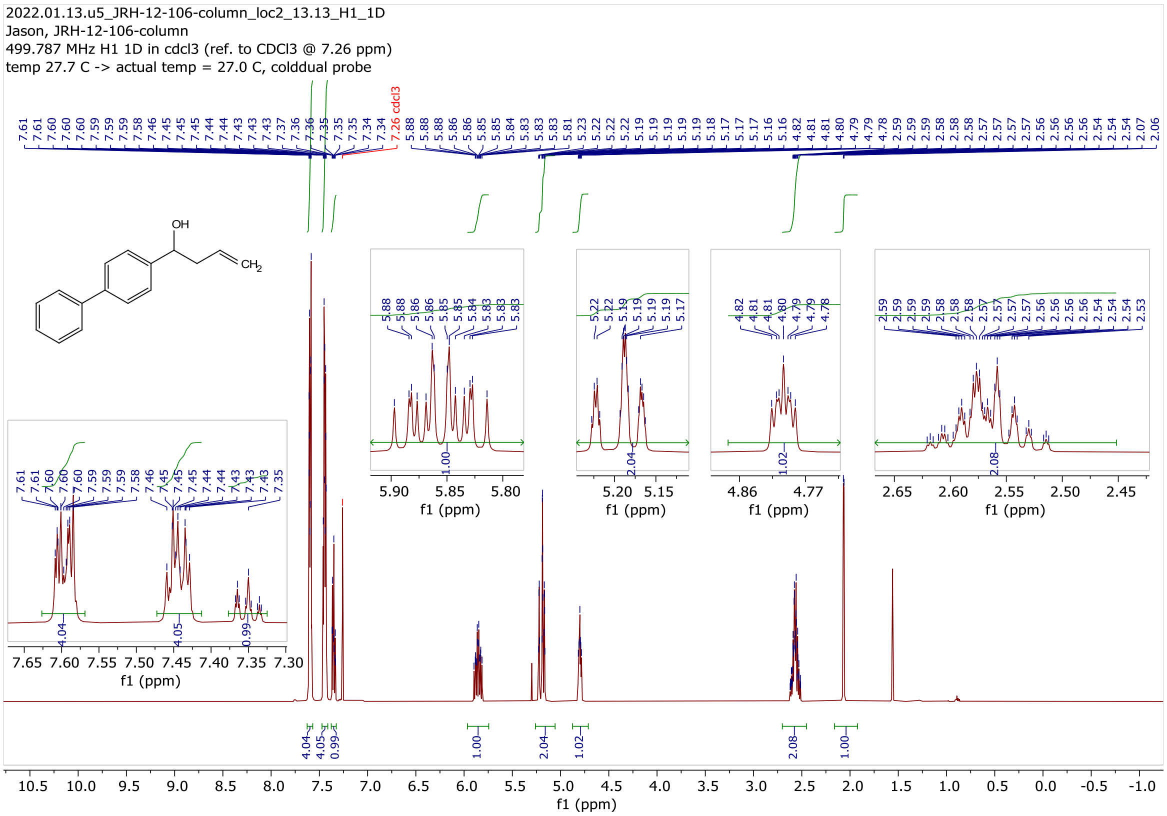

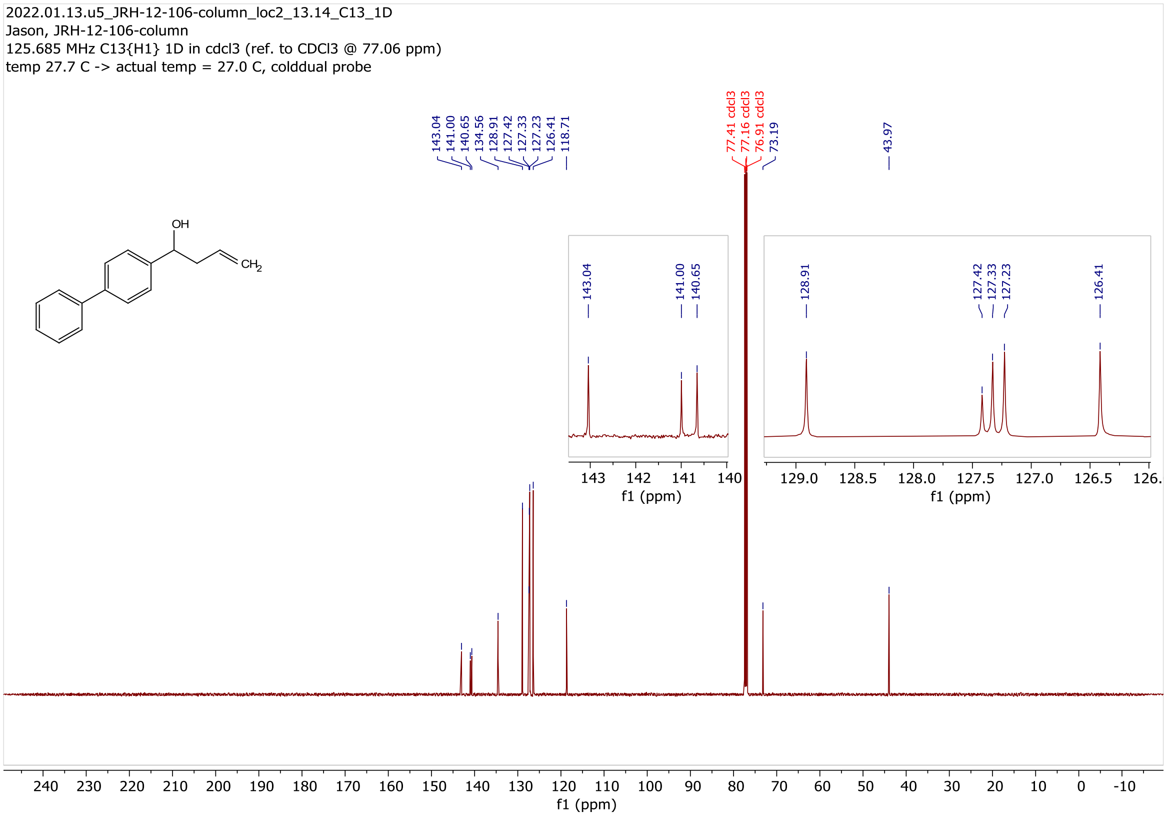

^{1}H (500 MHz) and ^{13}C (126 MHz) NMR of compound 6y ($CDCl_3$)

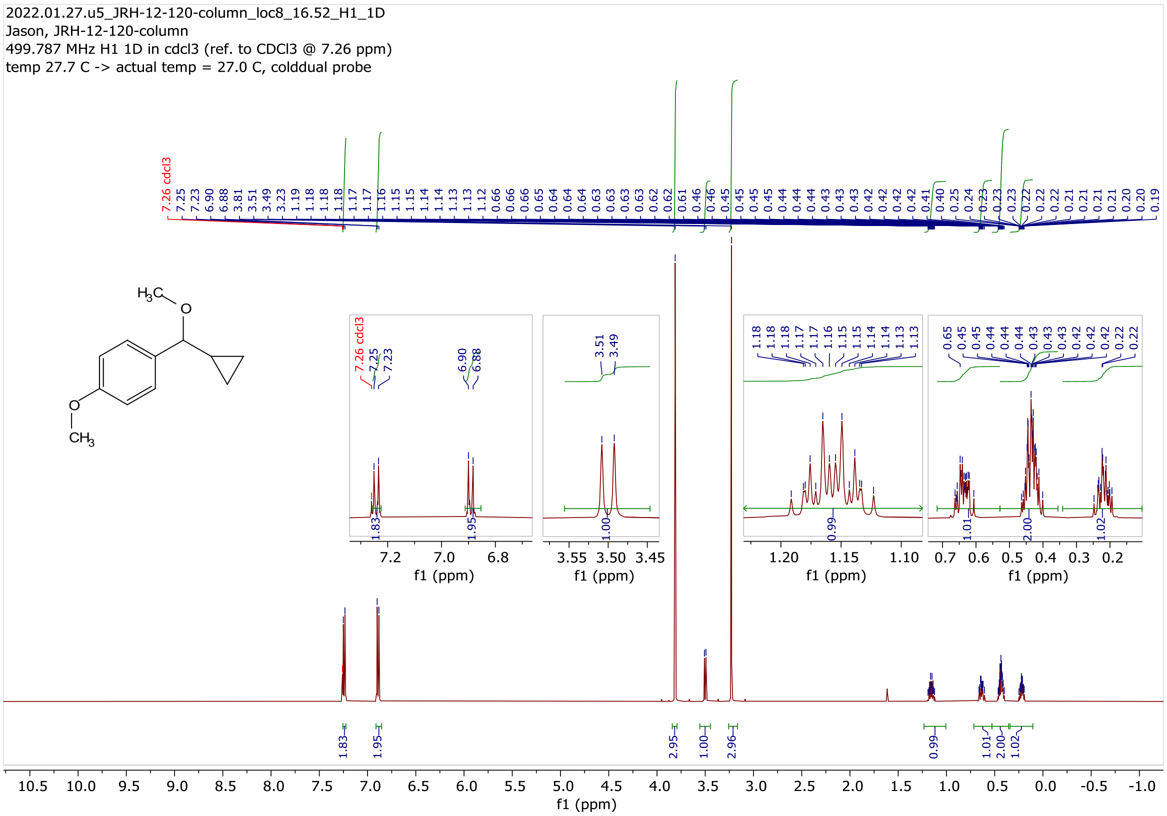

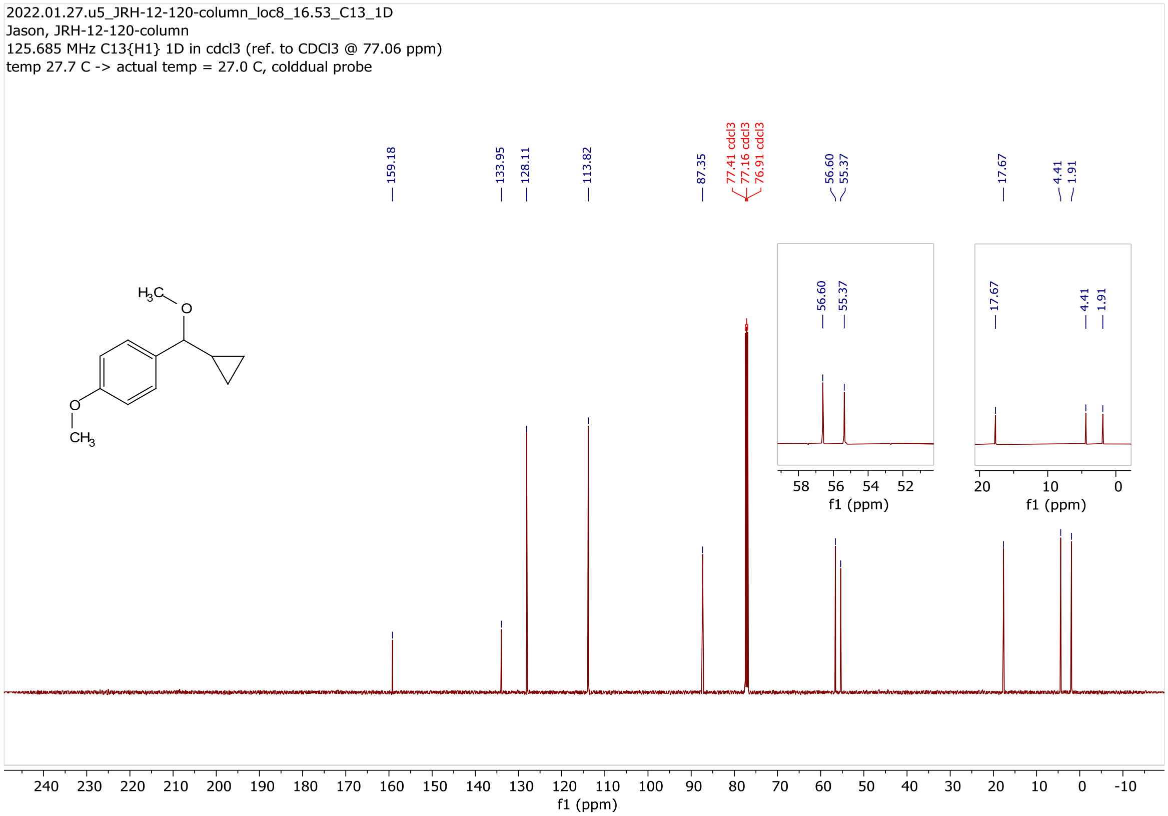

^{1}H (500 MHz) and ^{13}C (126 MHz) NMR of compound 6z ($CDCl_3$)

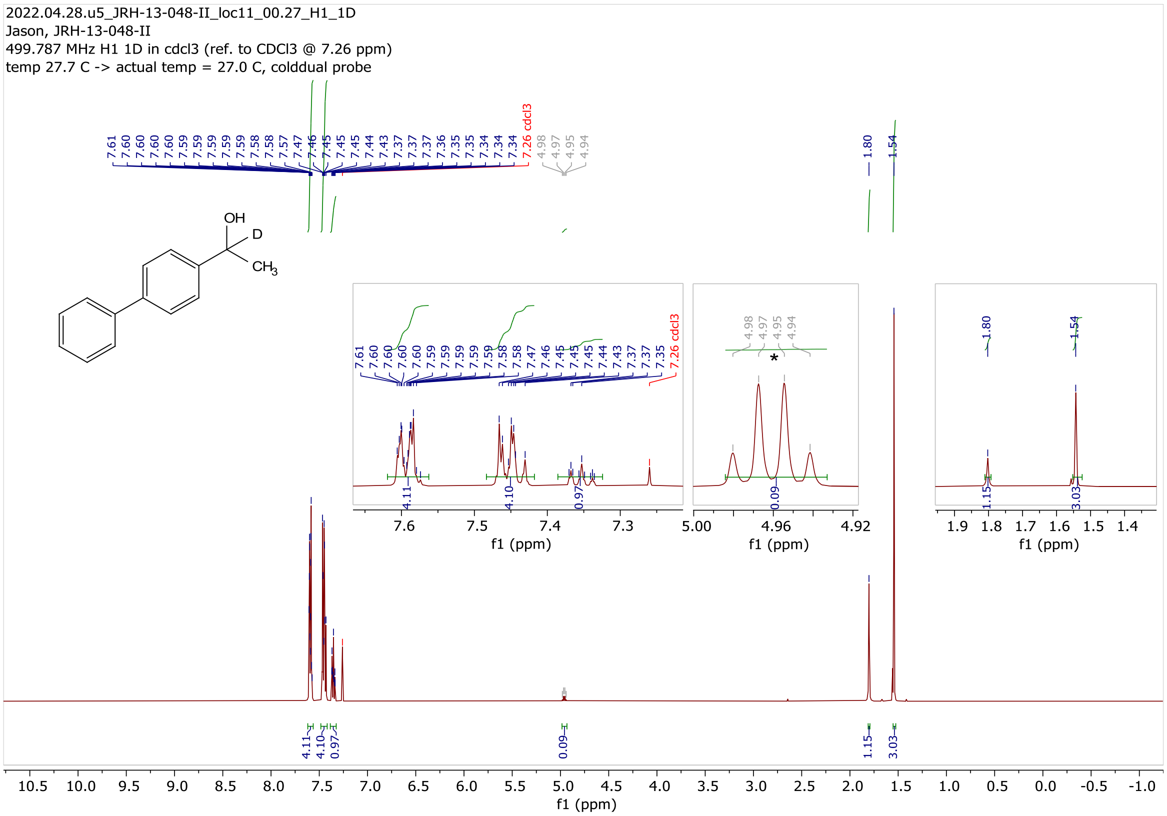

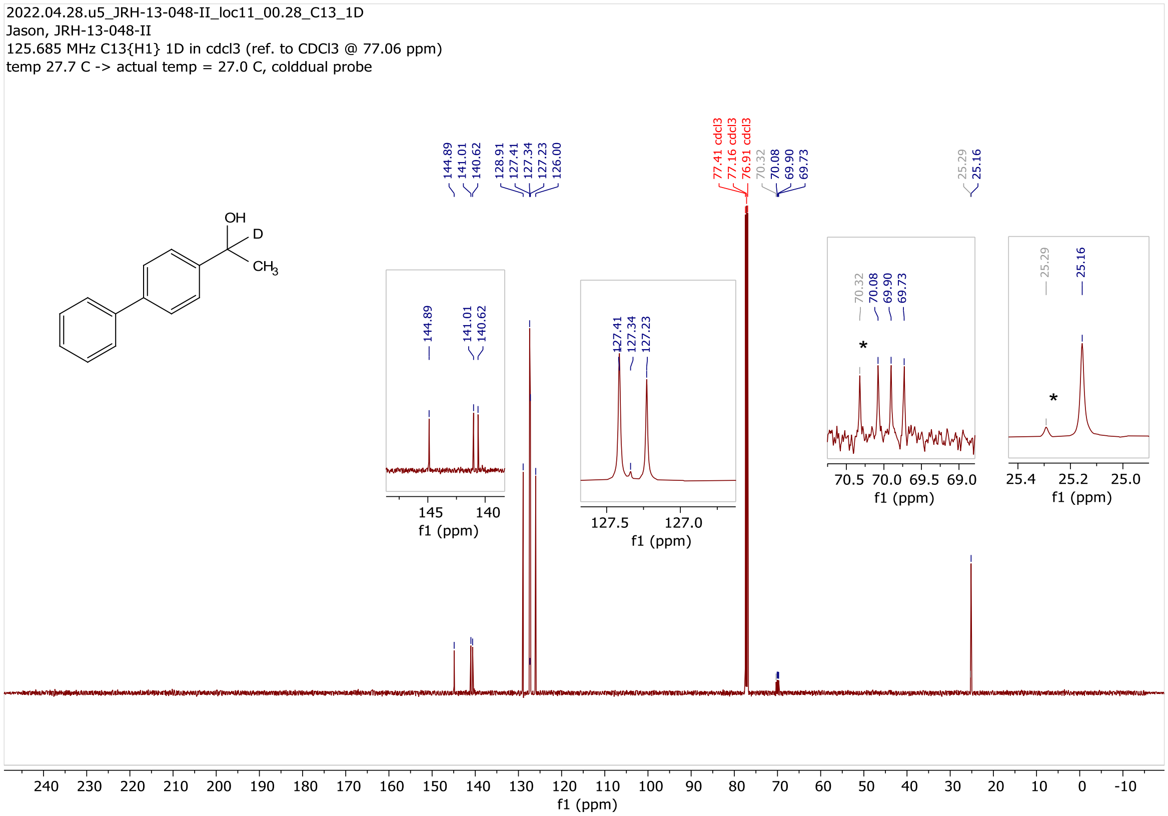

^{1}H (500 MHz) and ^{13}C (126 MHz) NMR of compound 6aa ($CDCl_3$)

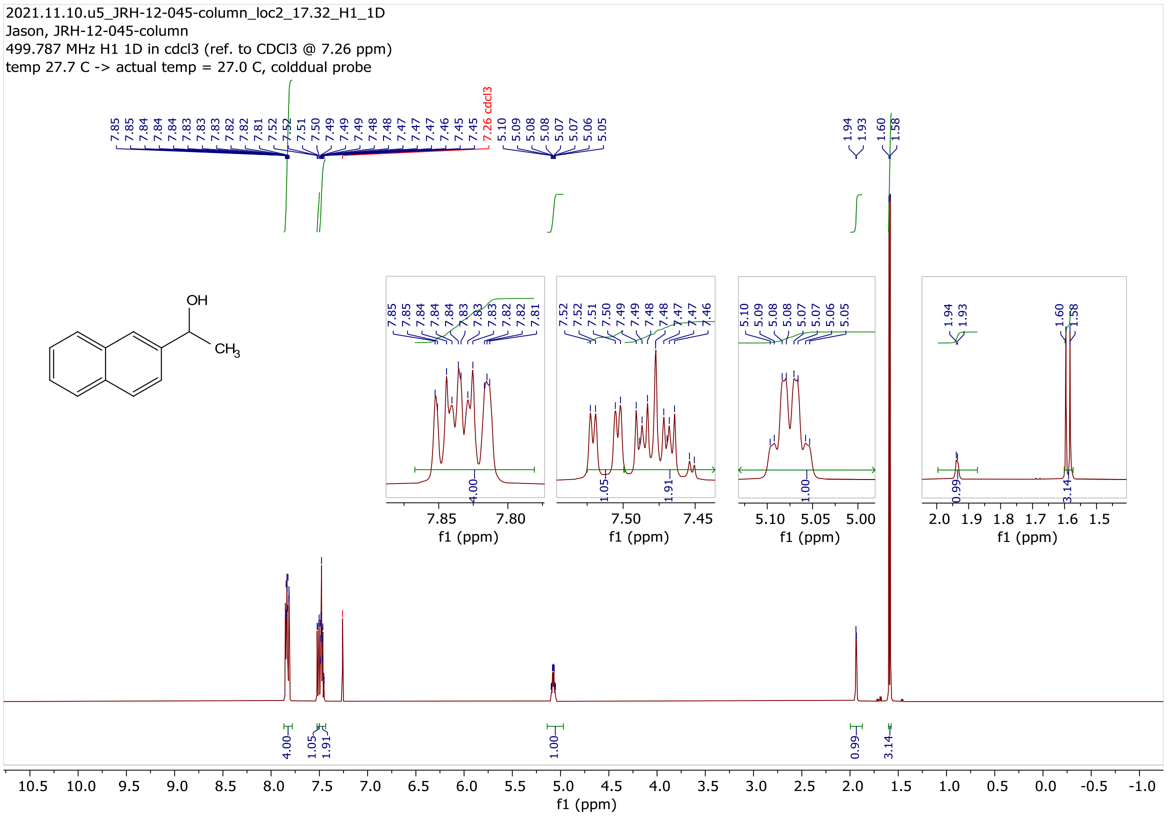

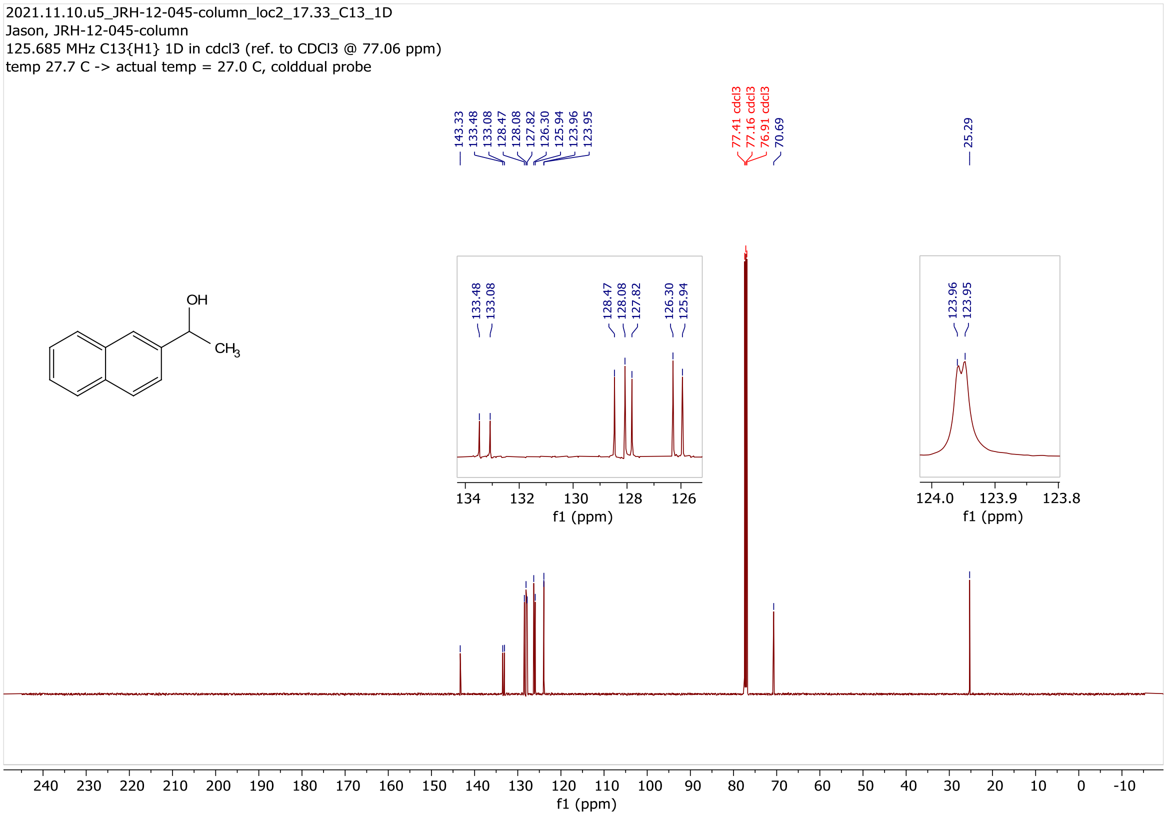

^{1}H (500 MHz) and ^{13}C (126 MHz) NMR of compound 8ac ($CDCl_3$)

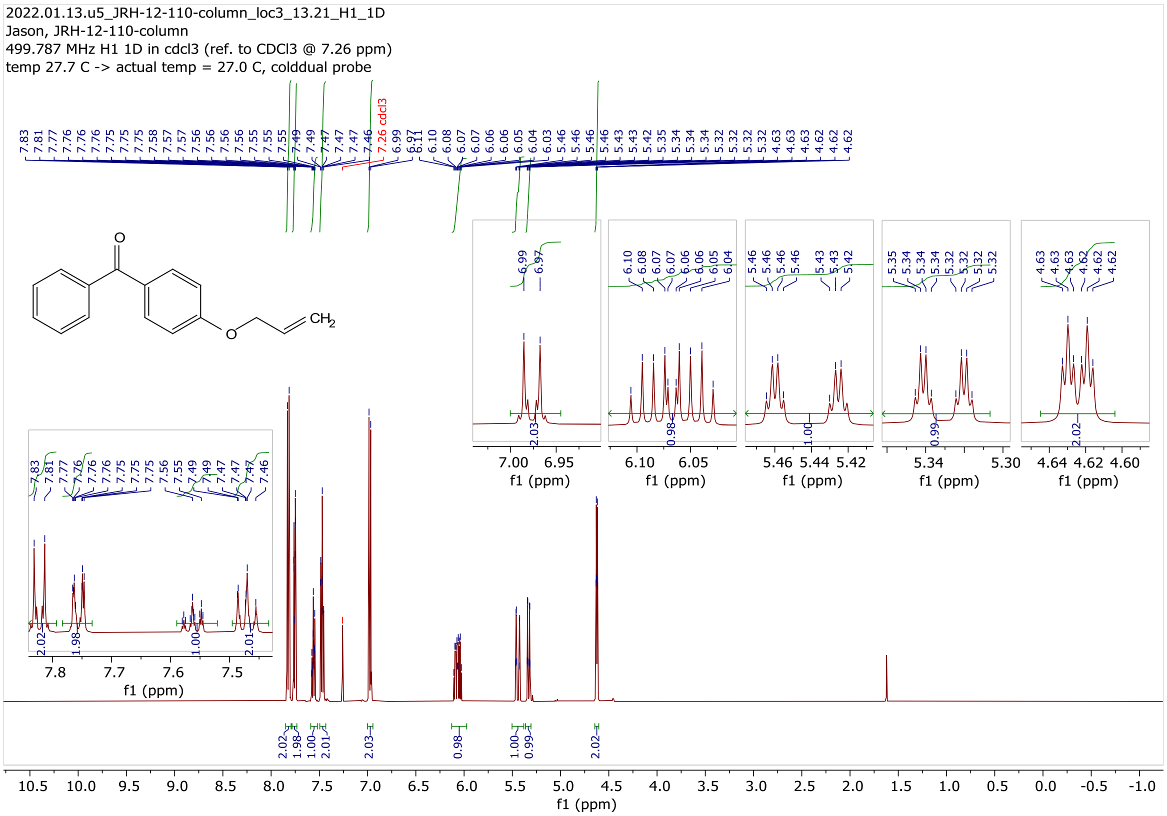

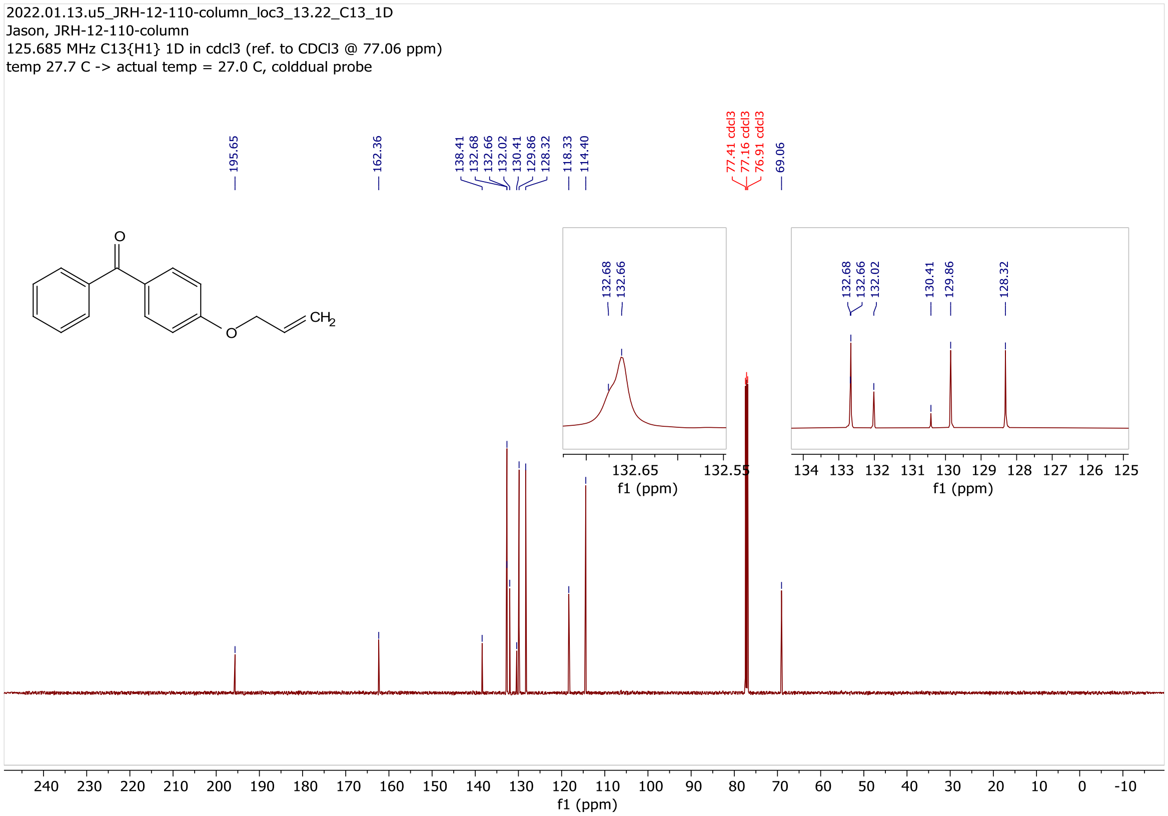

^{1}H (500 MHz) and ^{13}C (126 MHz) NMR of compound 8ae ($CDCl_3$)

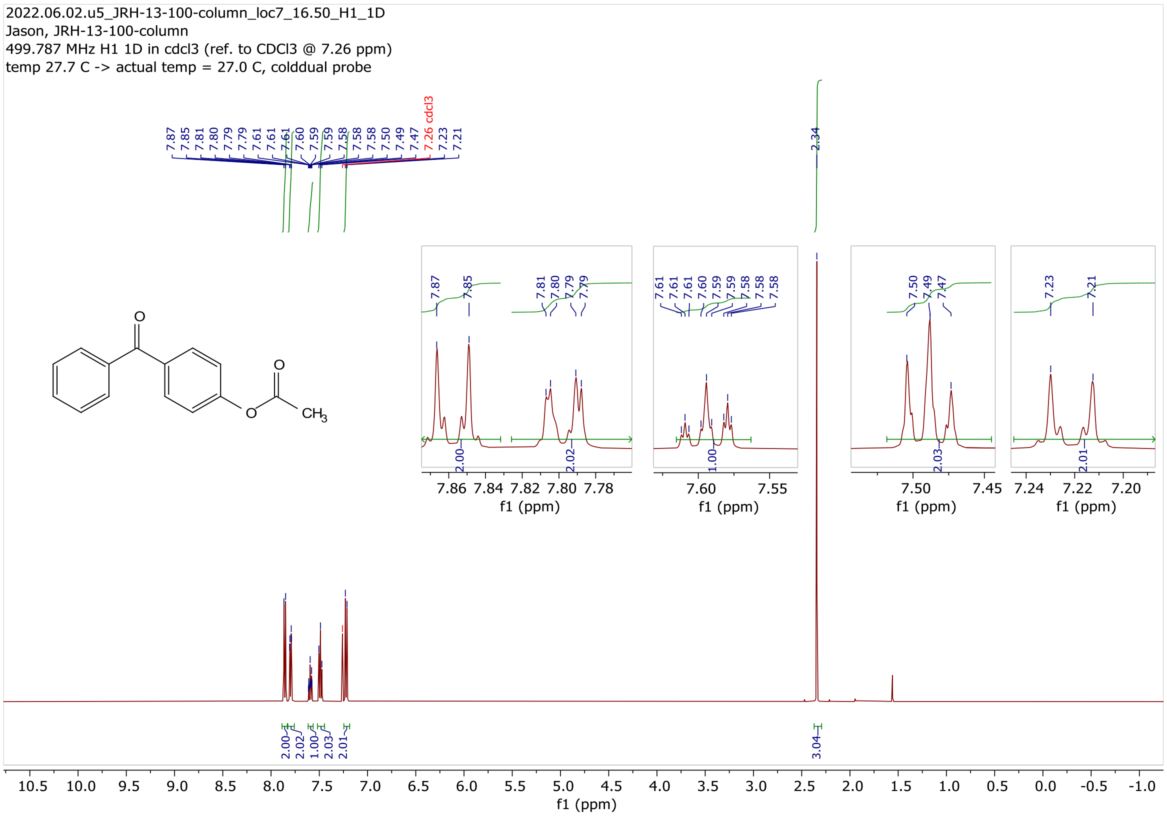

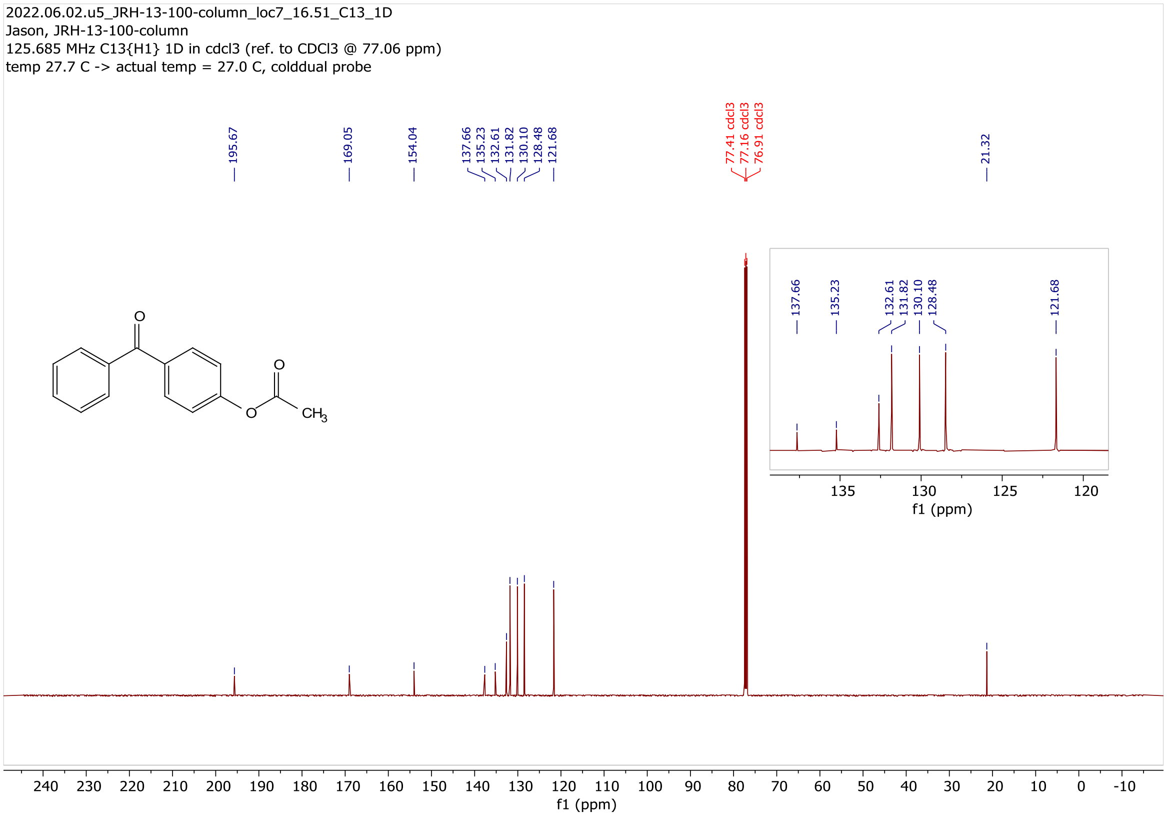

^{1}H (500 MHz) and ^{13}C (126 MHz) NMR of compound 8af ($CDCl_3$)

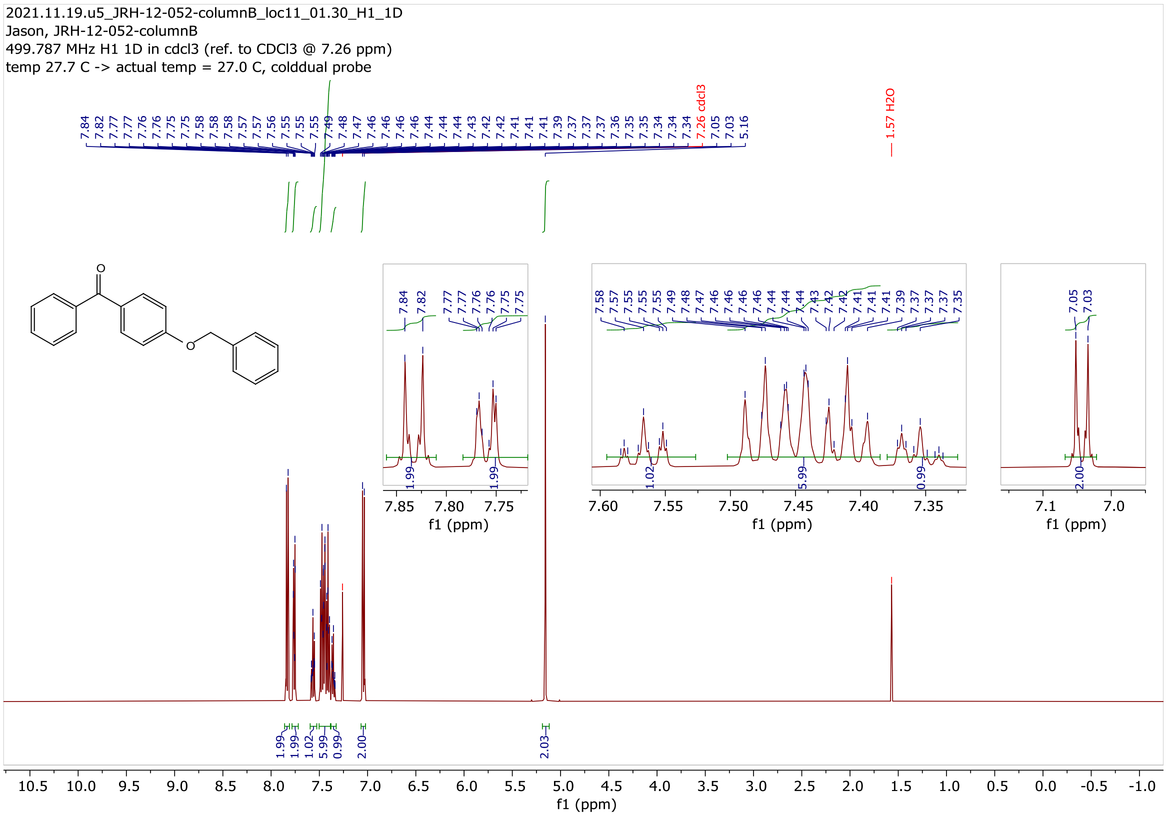

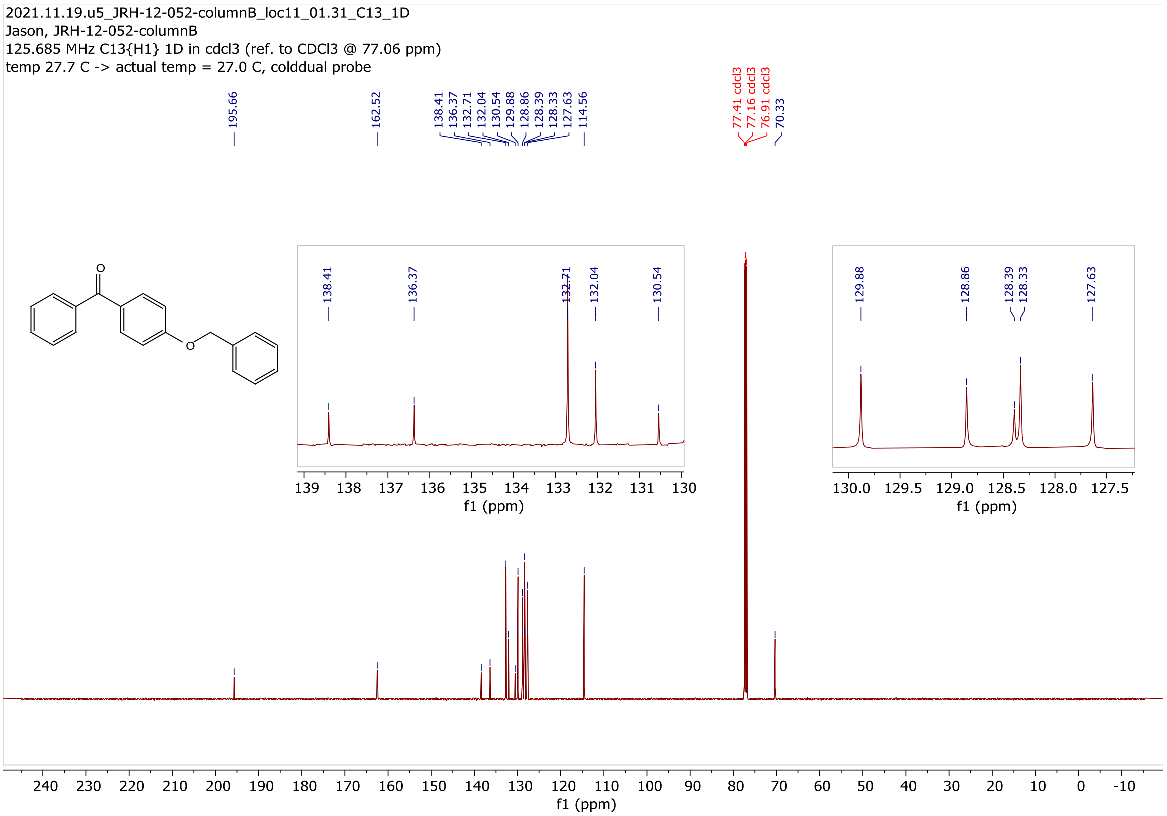

^{1}H (500 MHz) and ^{13}C (126 MHz) NMR of compound 8ai ($CDCl_3$)

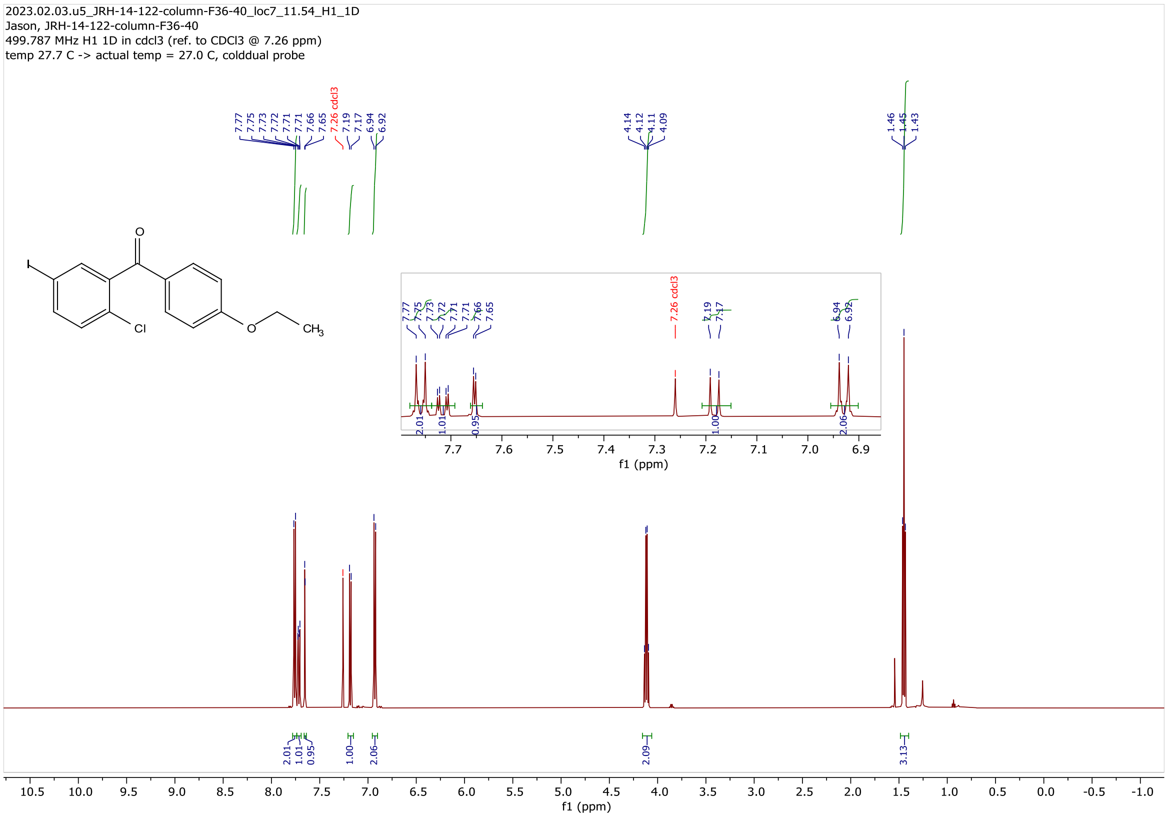

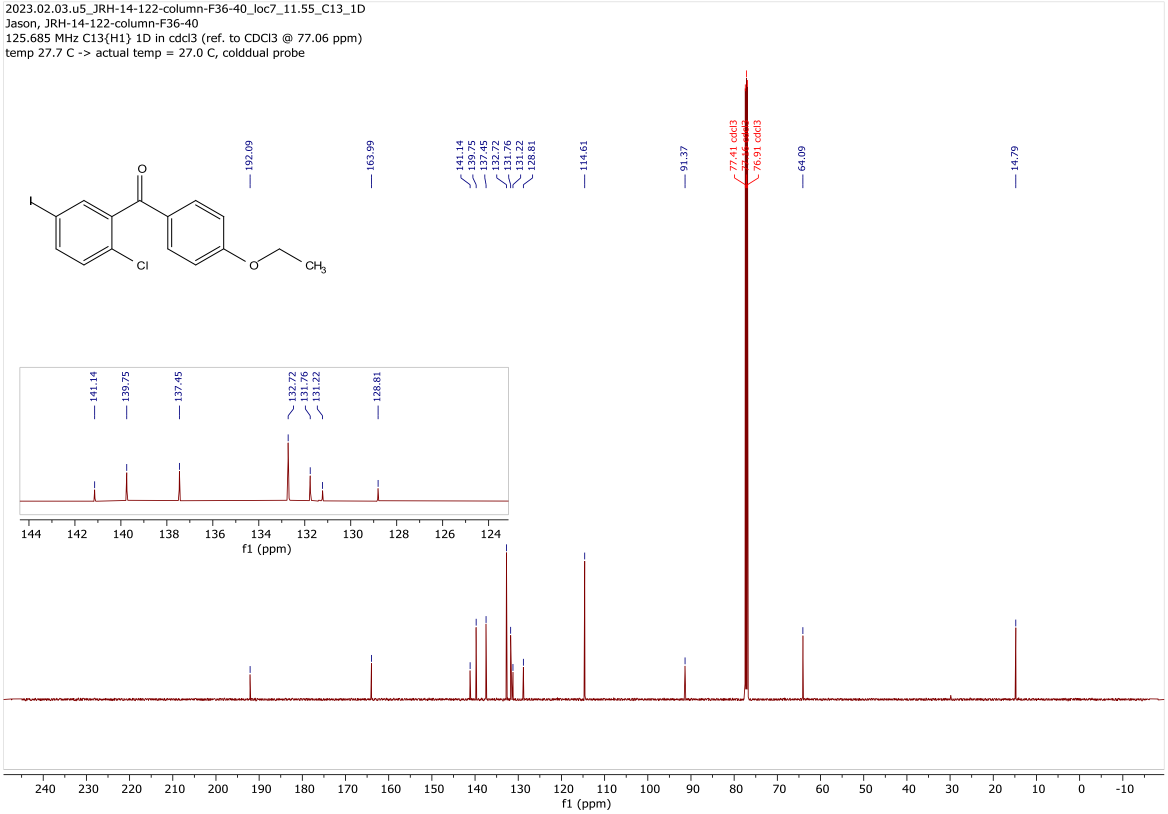

^{1}H (500 MHz) and ^{13}C (126 MHz) NMR of compound 7a ($CDCl_3$)

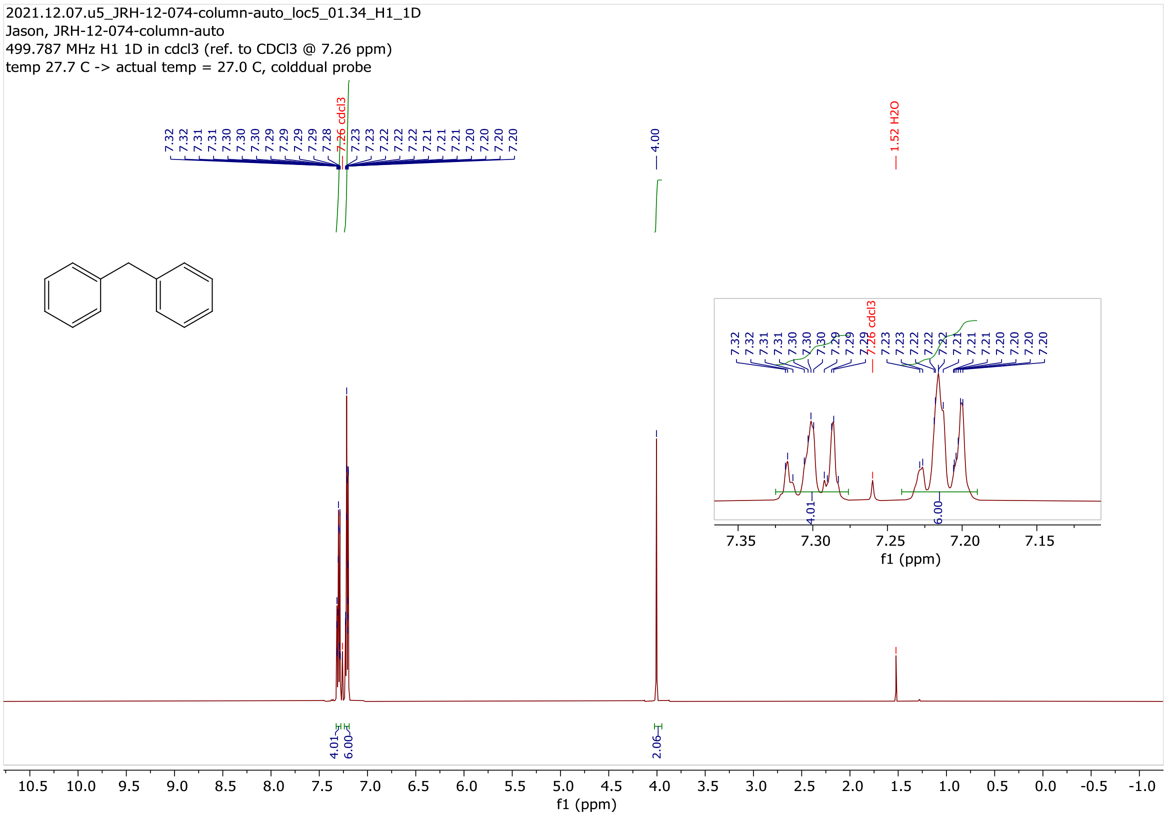

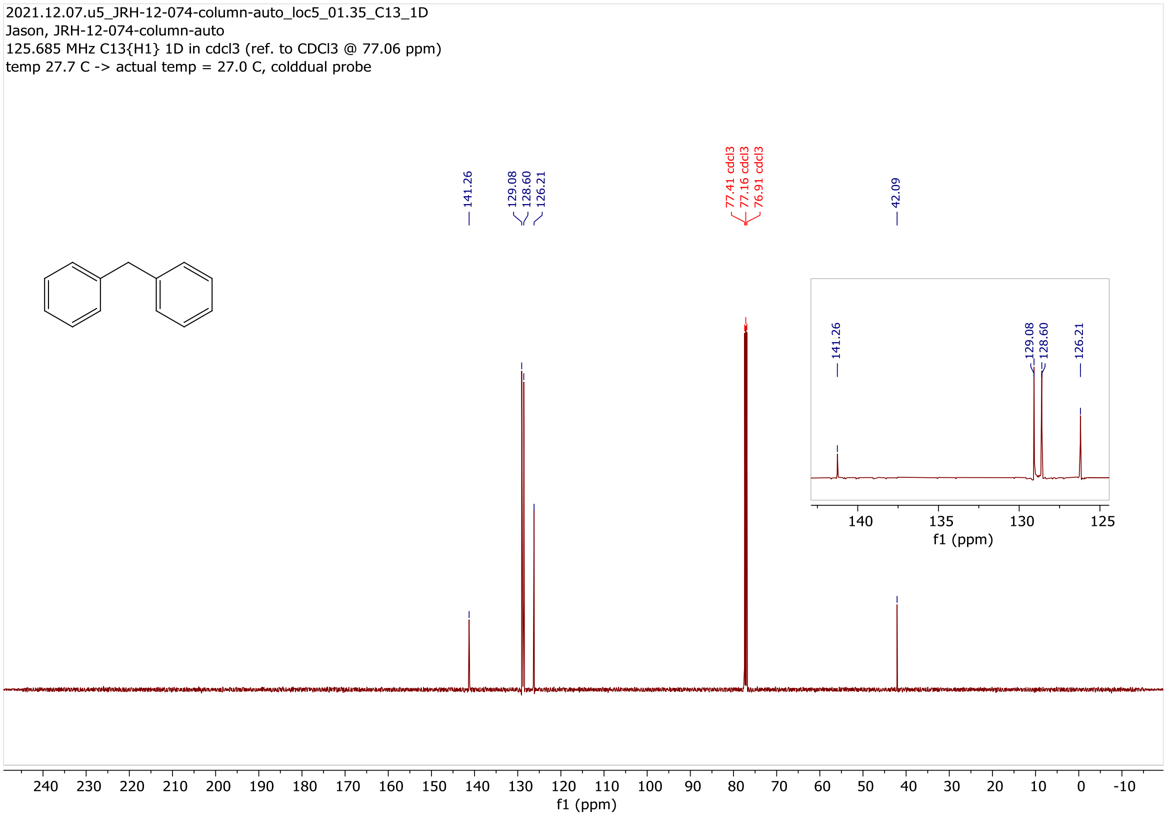

^{1}H (500 MHz), ^{13}C (126 MHz) and ^{19}F (376 MHz) NMR of compound 7b ($CDCl_3$)

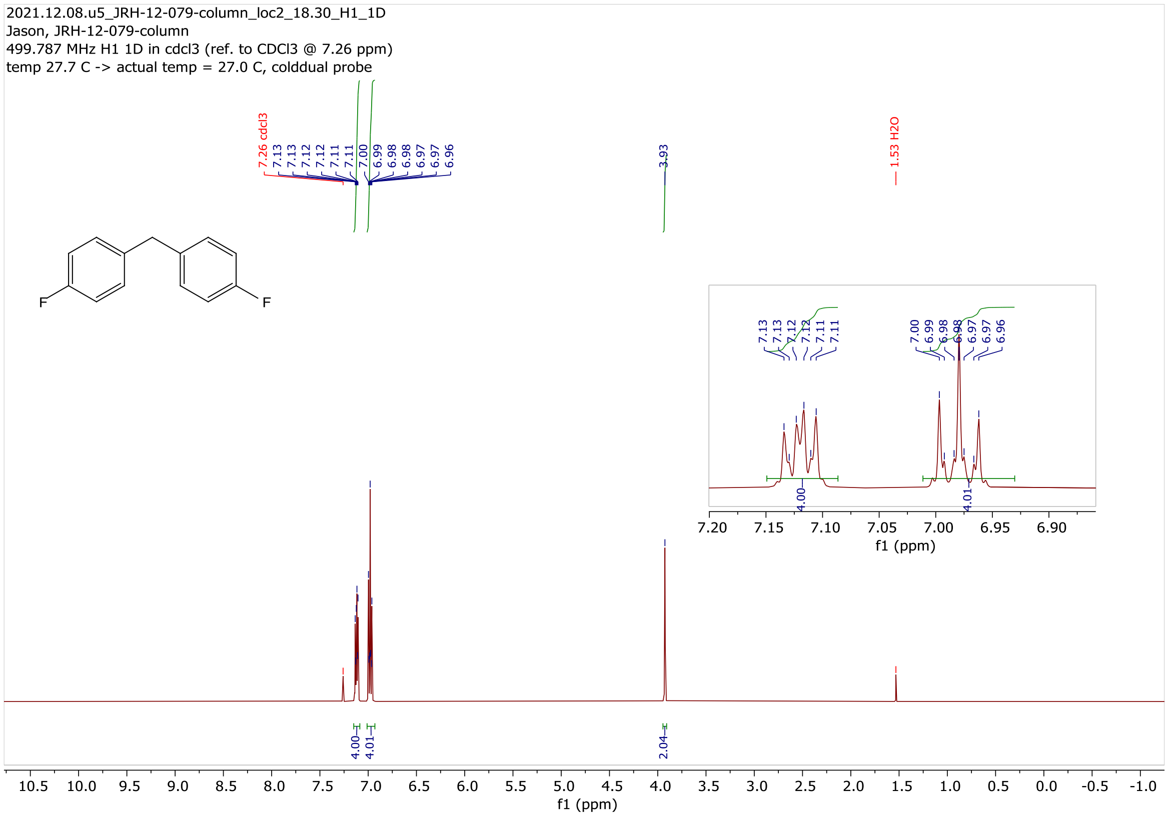

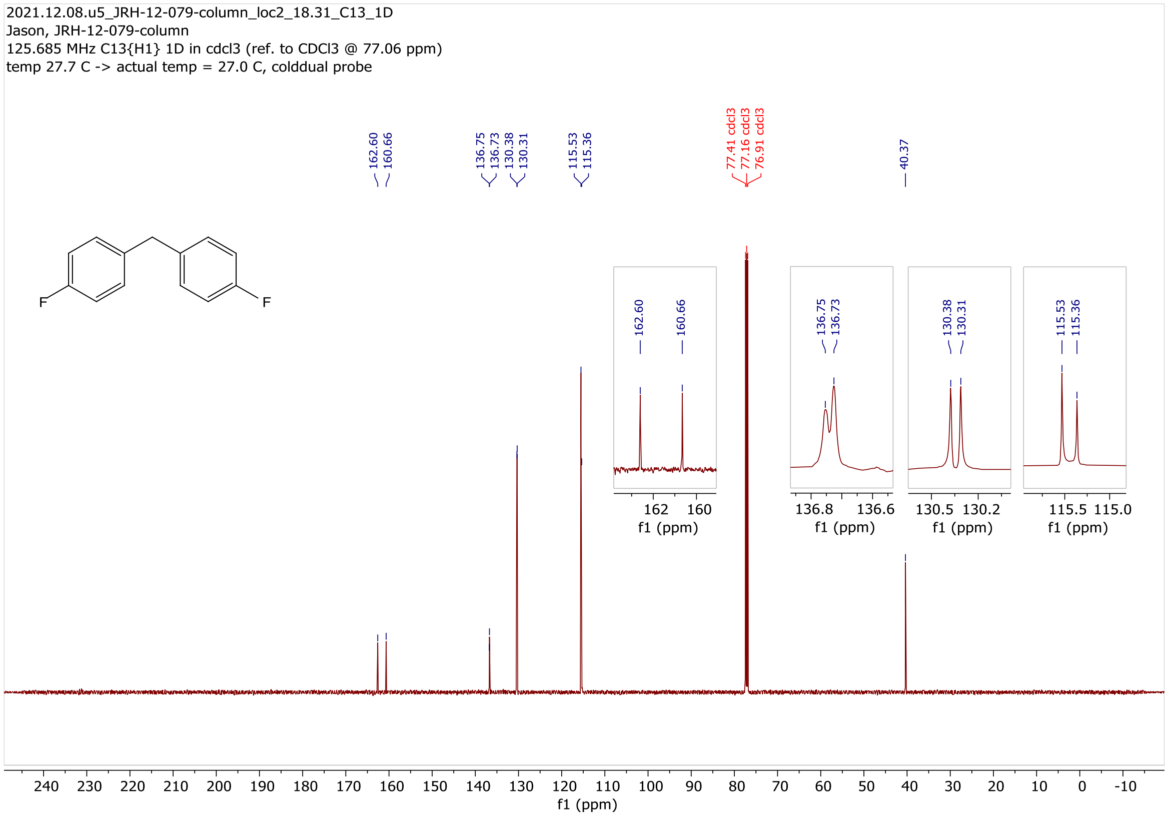

2021.12.13.mr4_JRH-12-079-column_F19_1D

376.306 MHz F19 1D in cdcl3
temp 25.9 C -> actual temp = 27.0 C, onenmr probe

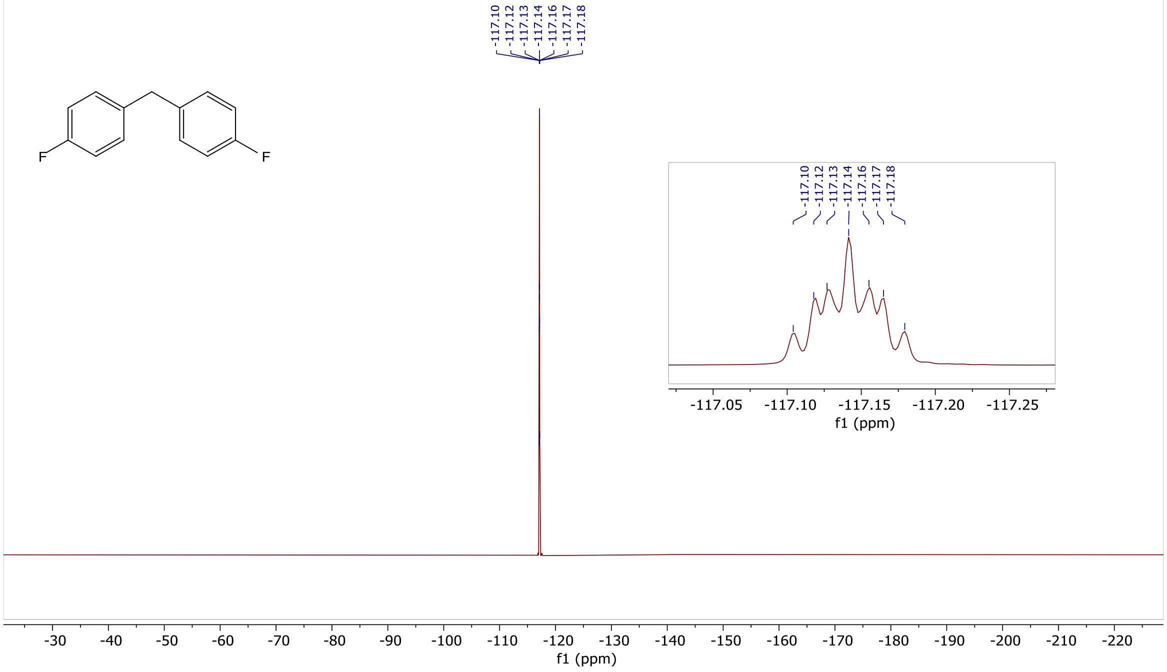

^{1}H (500 MHz) and ^{13}C (126 MHz) NMR of compound 7c ($CDCl_3$)

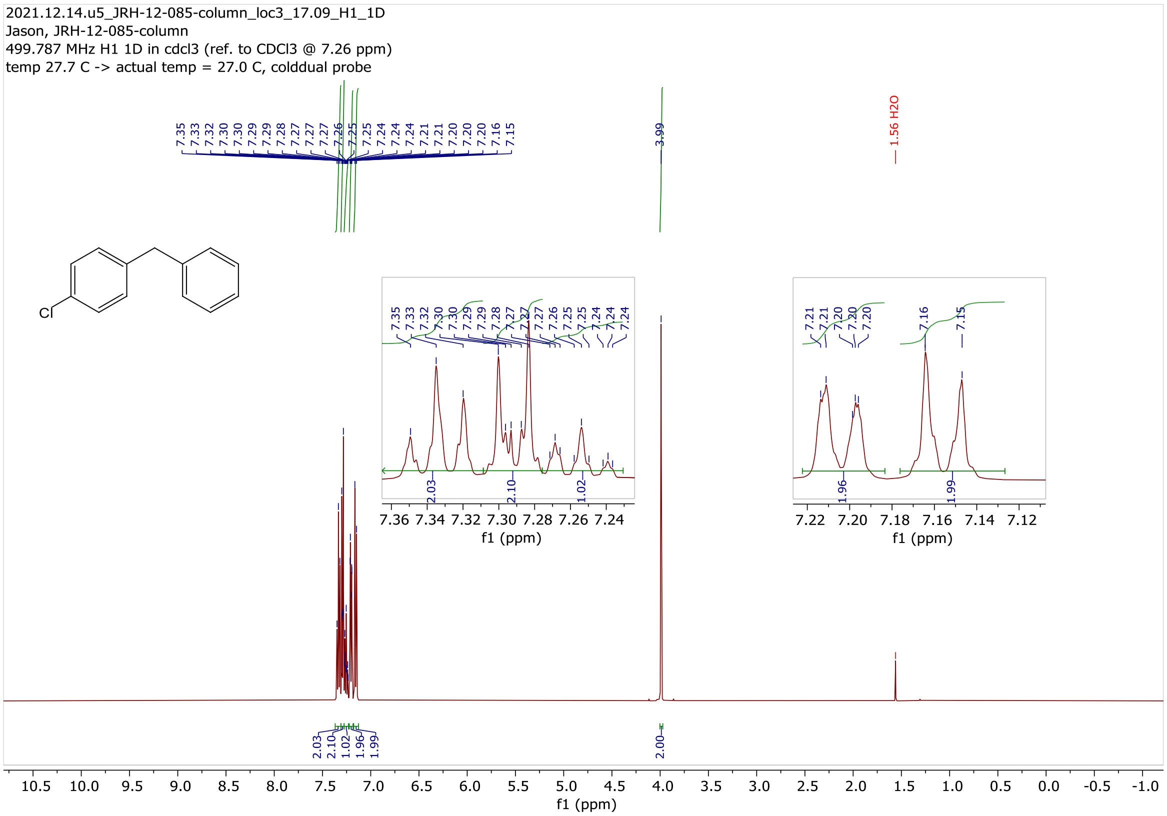

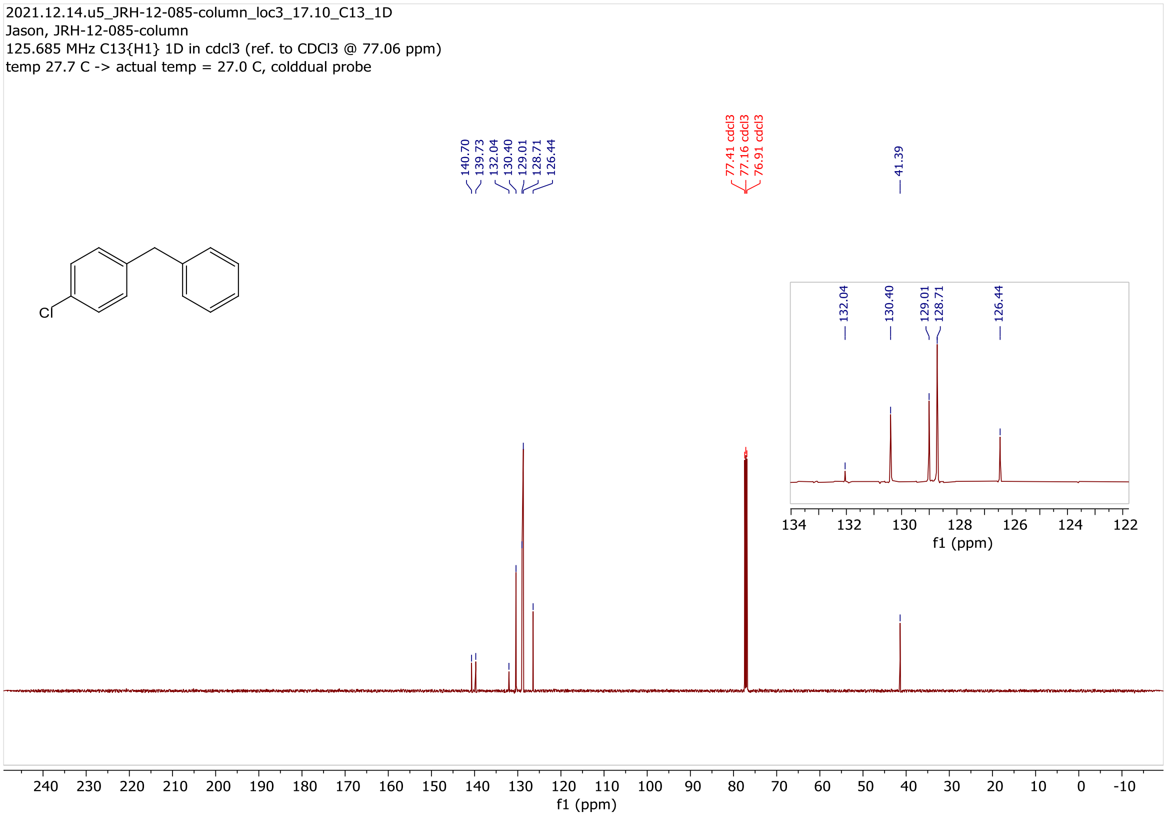

^{1}H (500 MHz) and ^{13}C (126 MHz) NMR of compound 7d ($CDCl_3$)

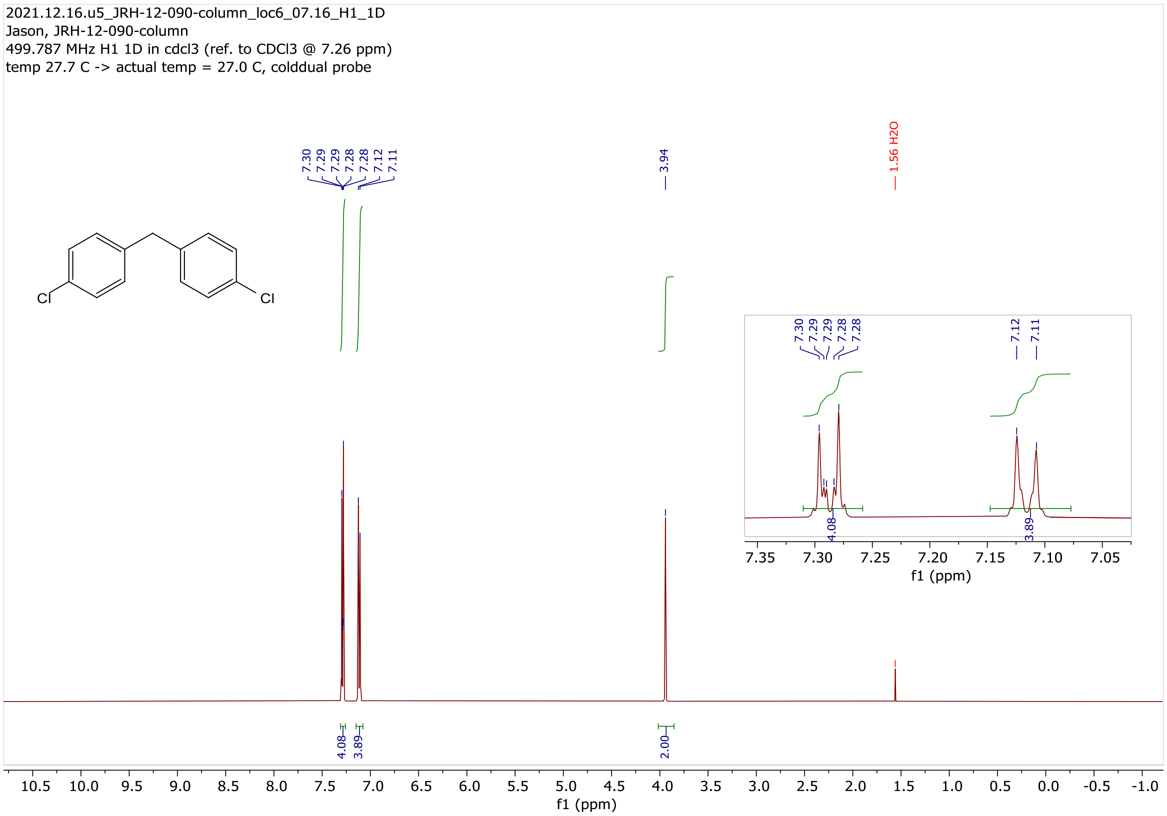

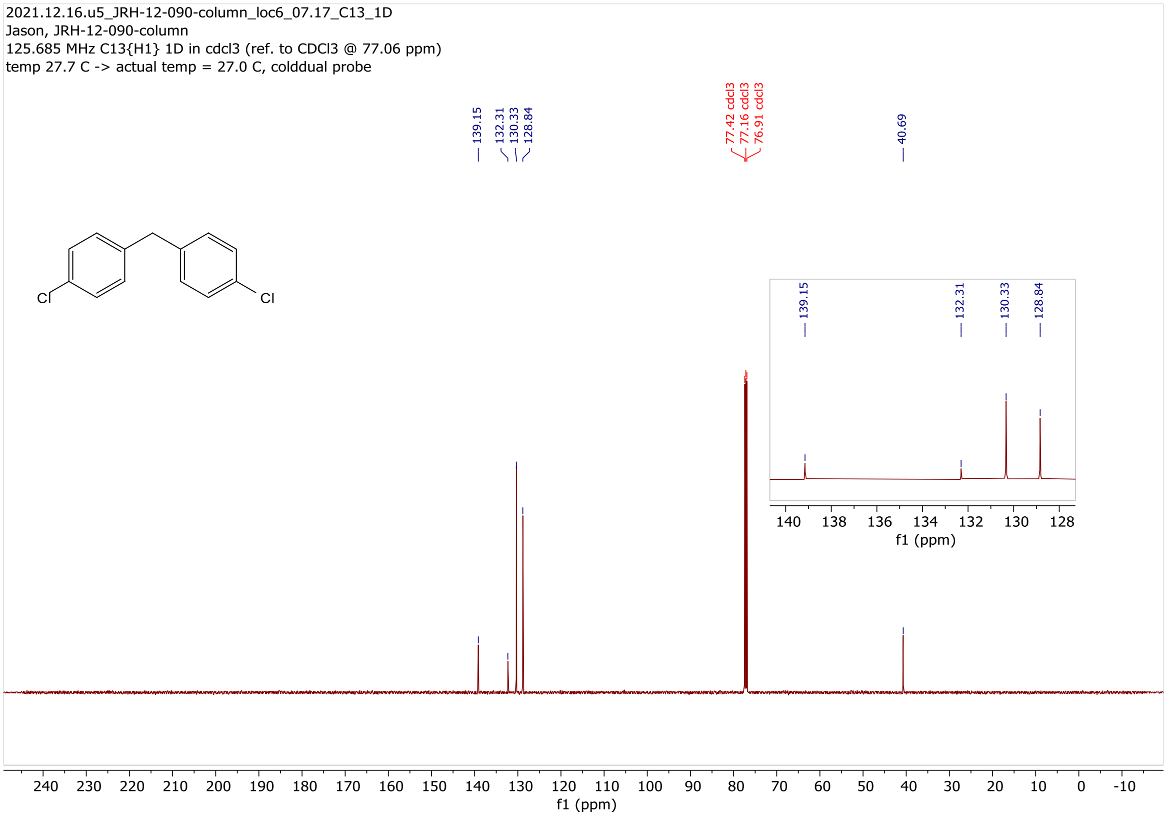

^{1}H (500 MHz) and ^{13}C (126 MHz) NMR of compound 7e ($CDCl_3$)

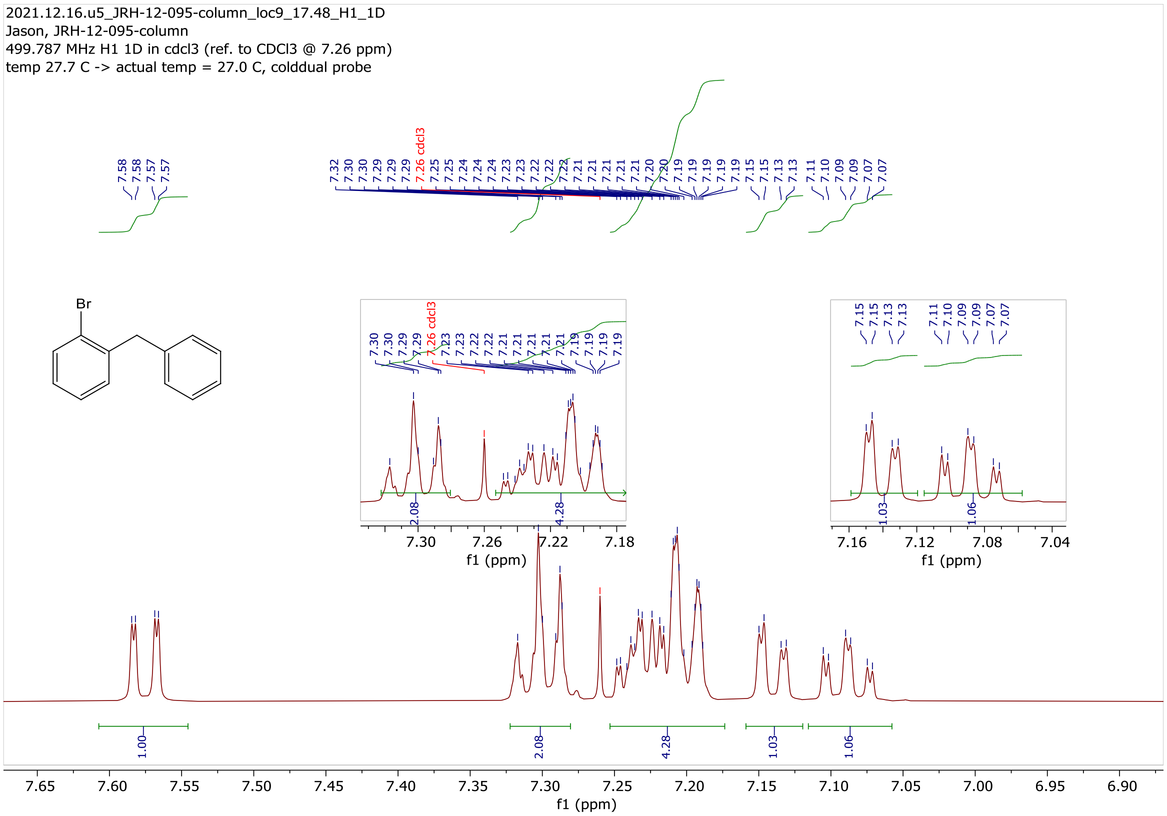

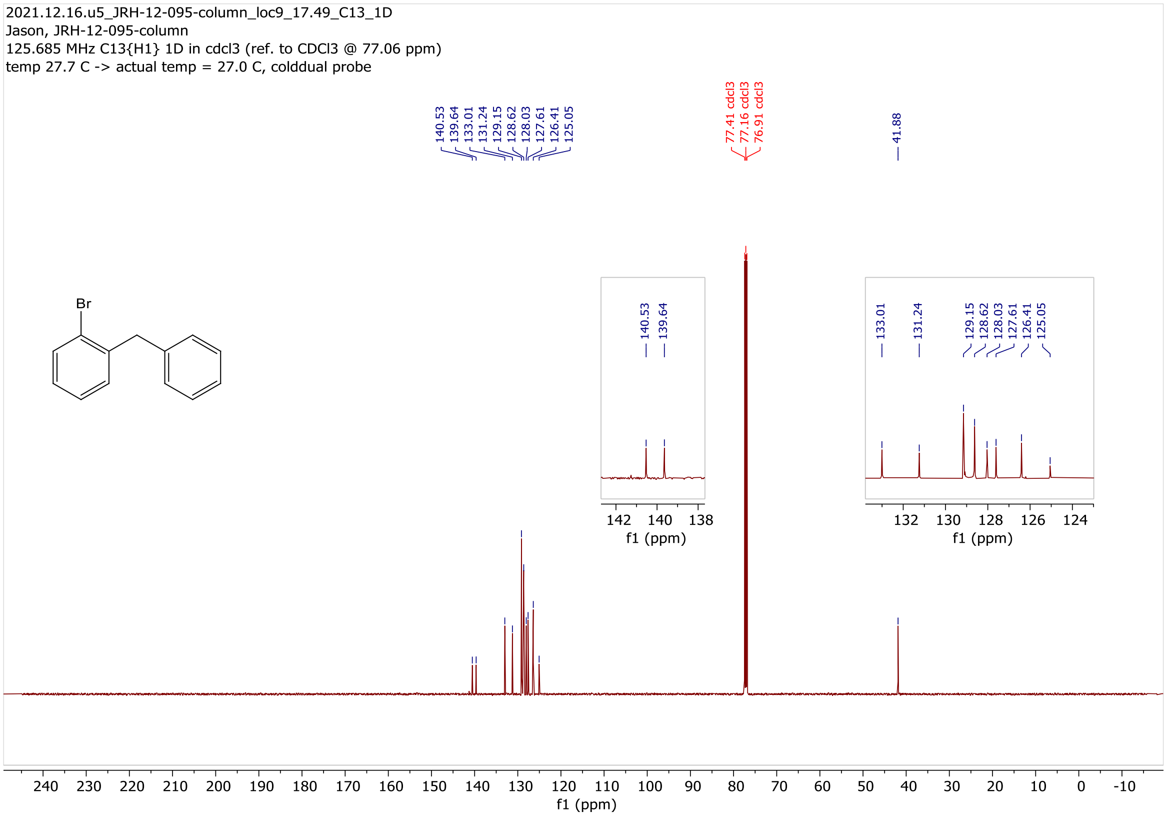

^{1}H (500 MHz) and ^{13}C (126 MHz) NMR of compound 7f ($CDCl_3$)

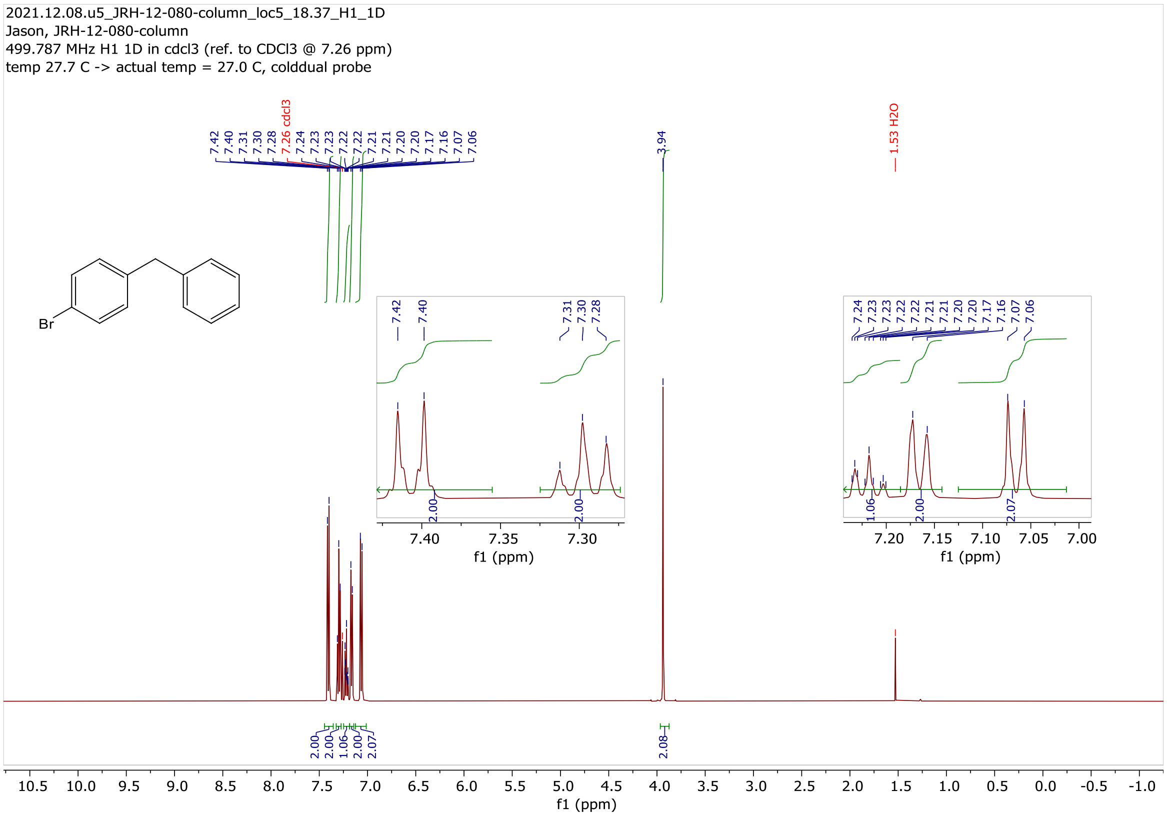

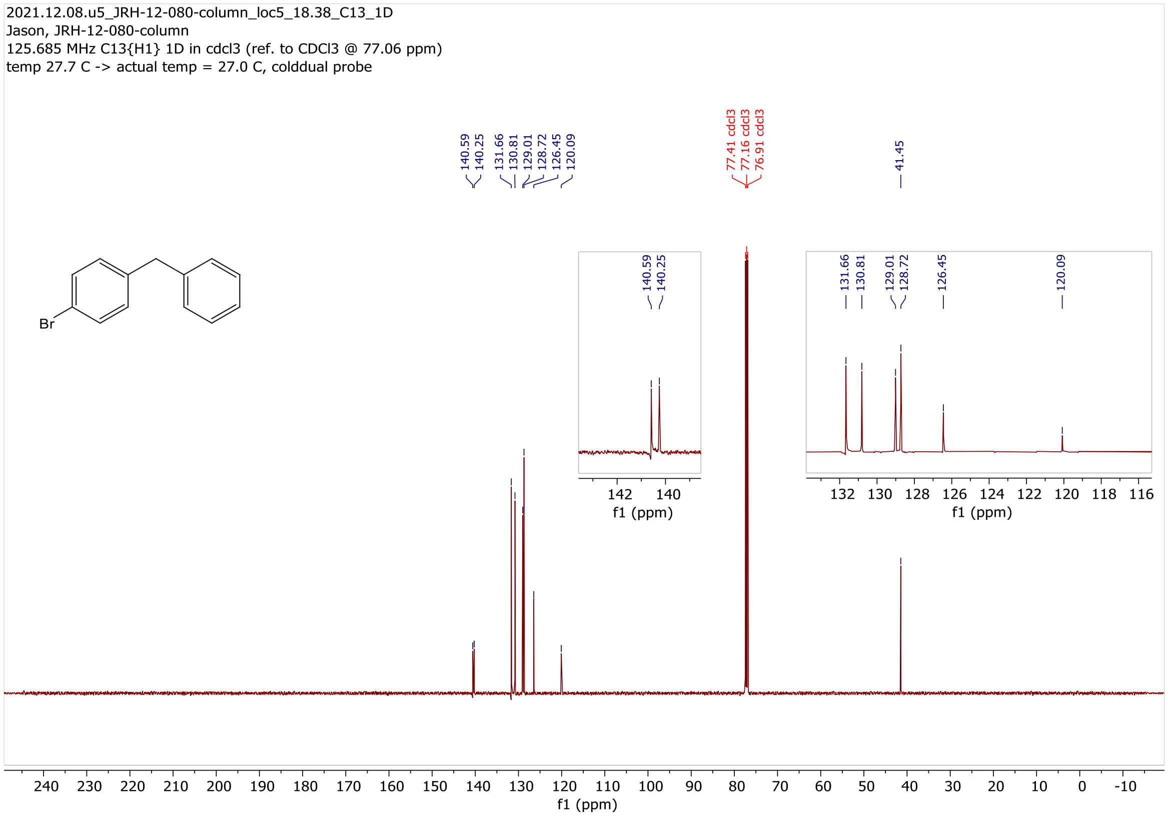

^{1}H (500 MHz) and ^{13}C (126 MHz) NMR of compound 7g

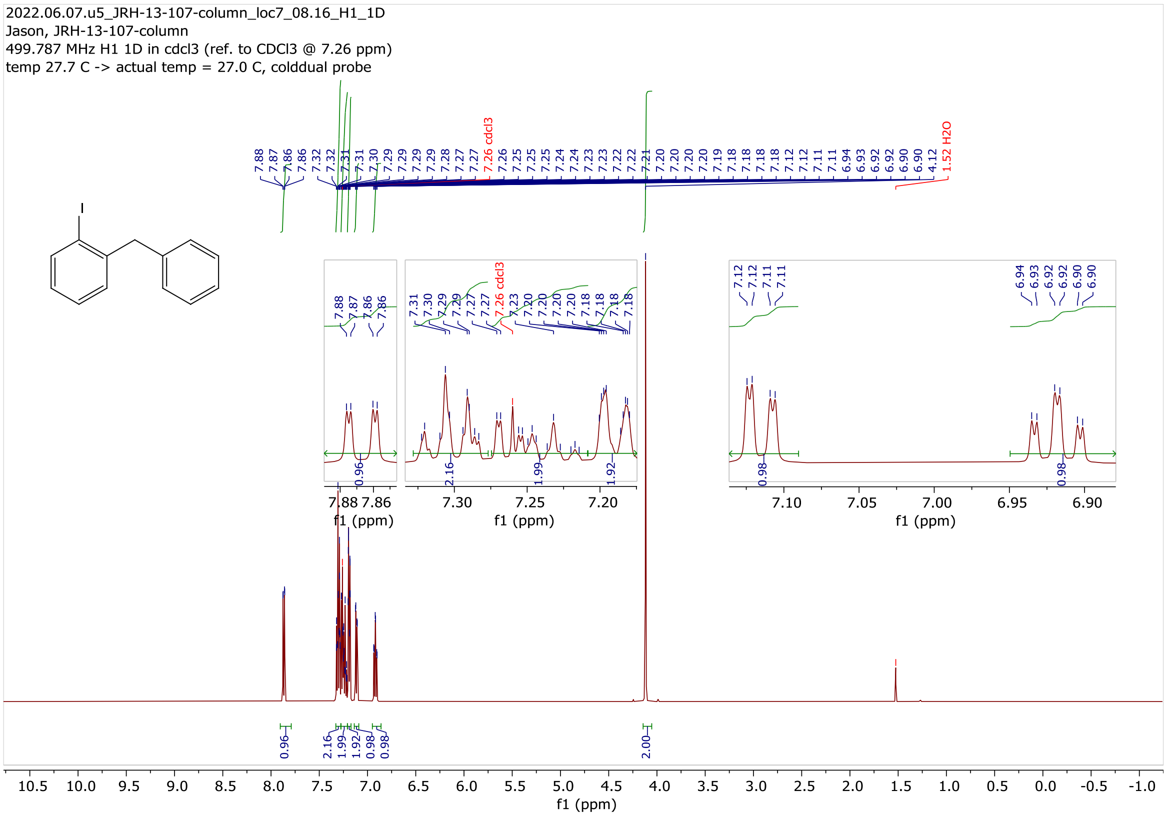

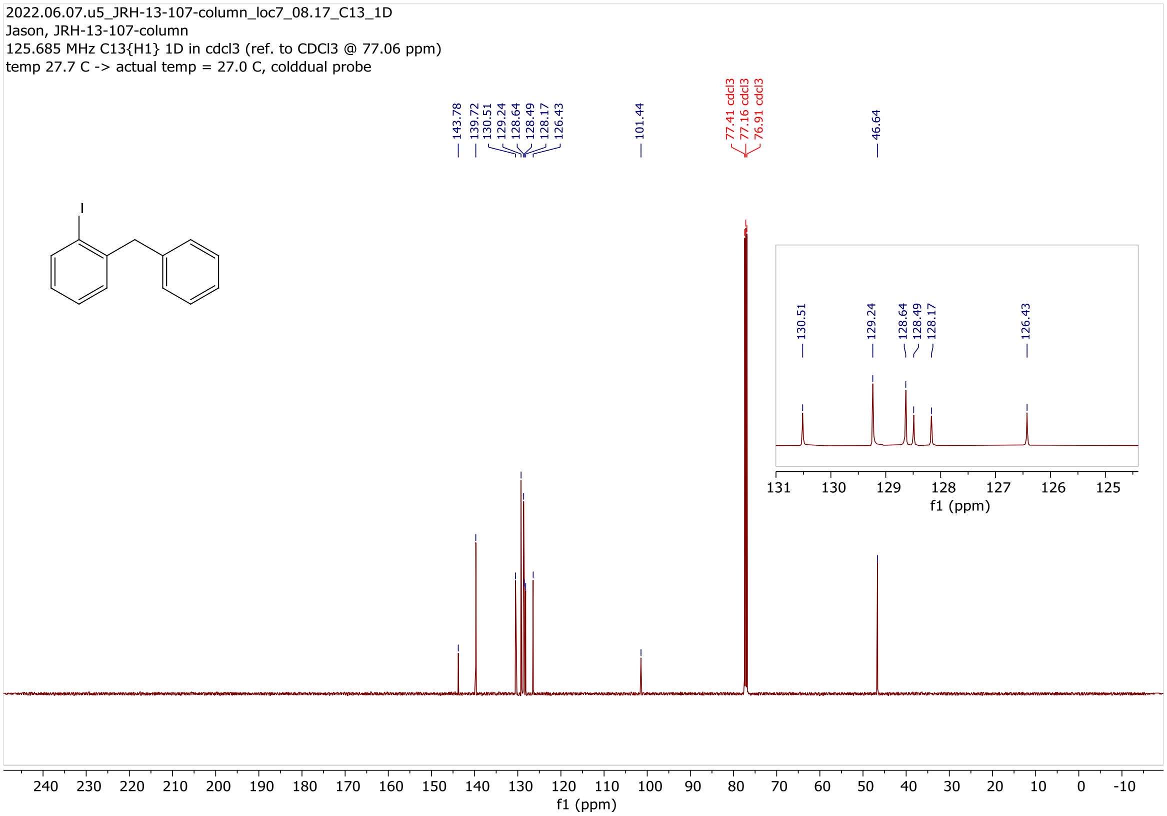

^{1}H (500 MHz) and ^{13}C (126 MHz) NMR of compound 7h ($CDCl_3$)

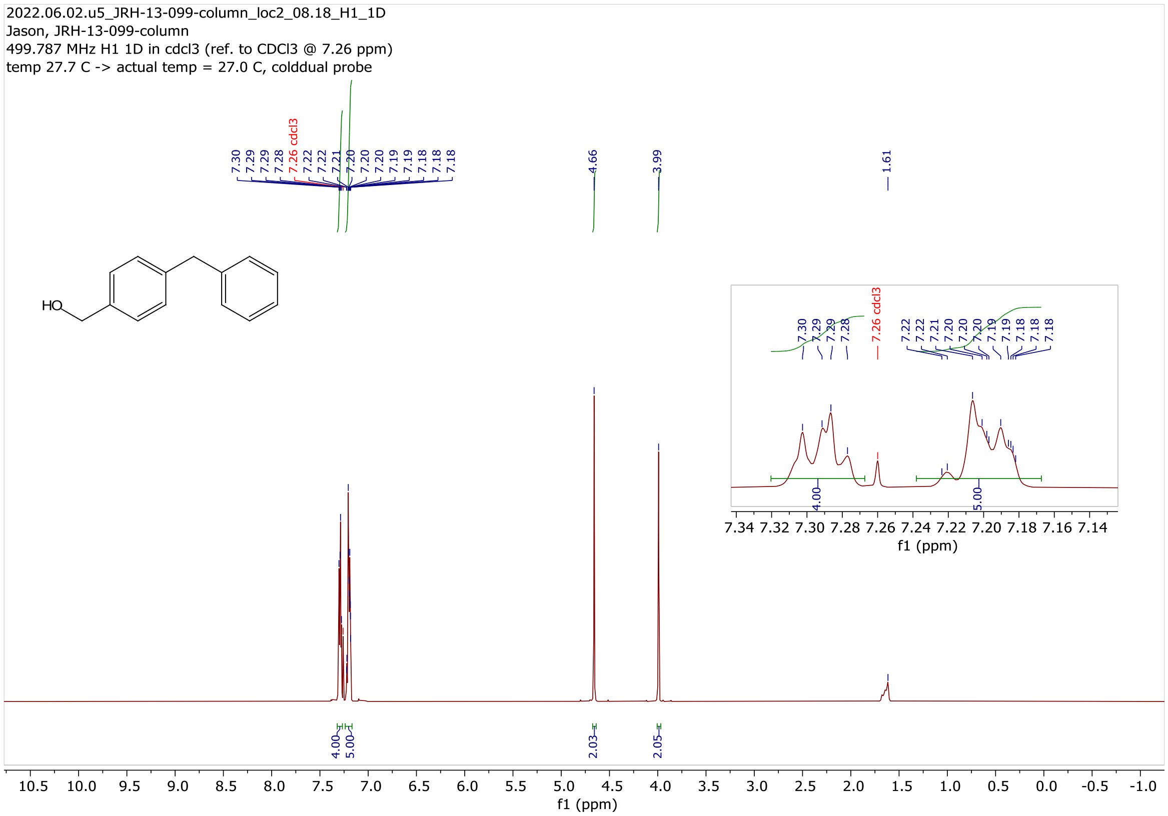

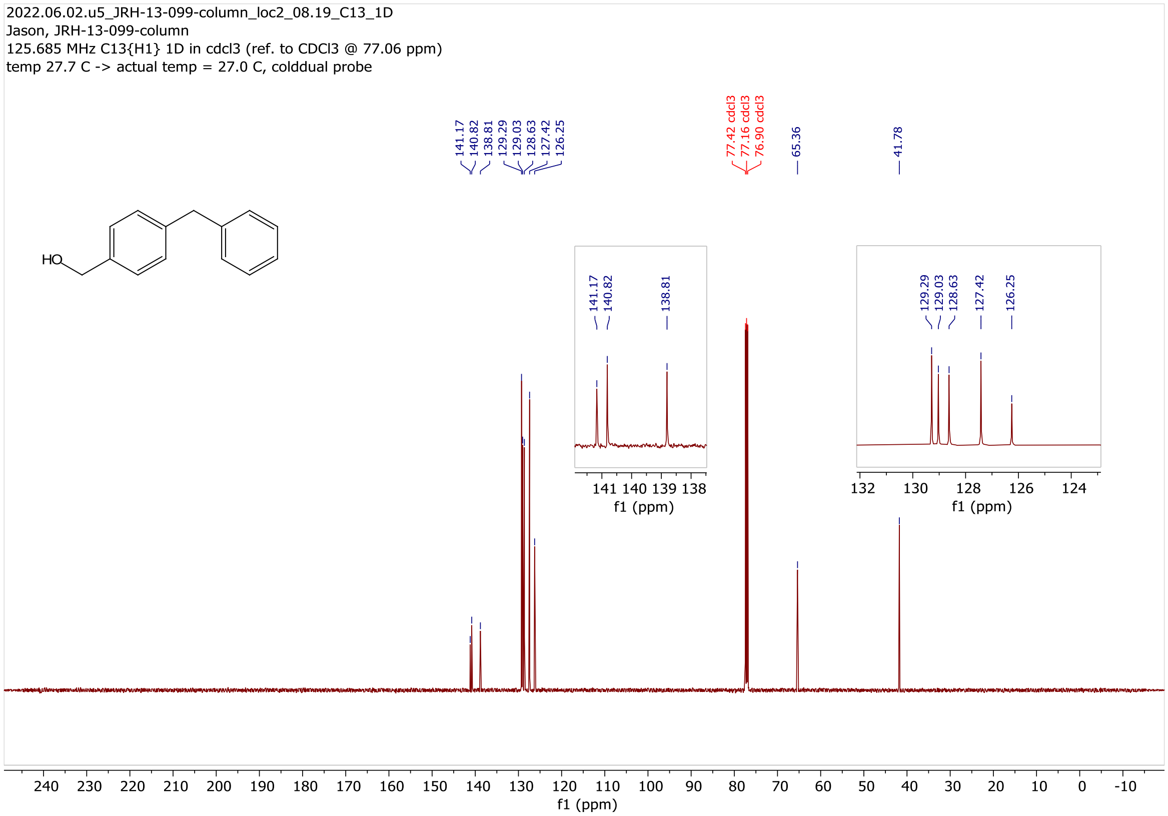

^{1}H (500 MHz) and ^{13}C (126 MHz) NMR of compound 7i ($CDCl_3$)

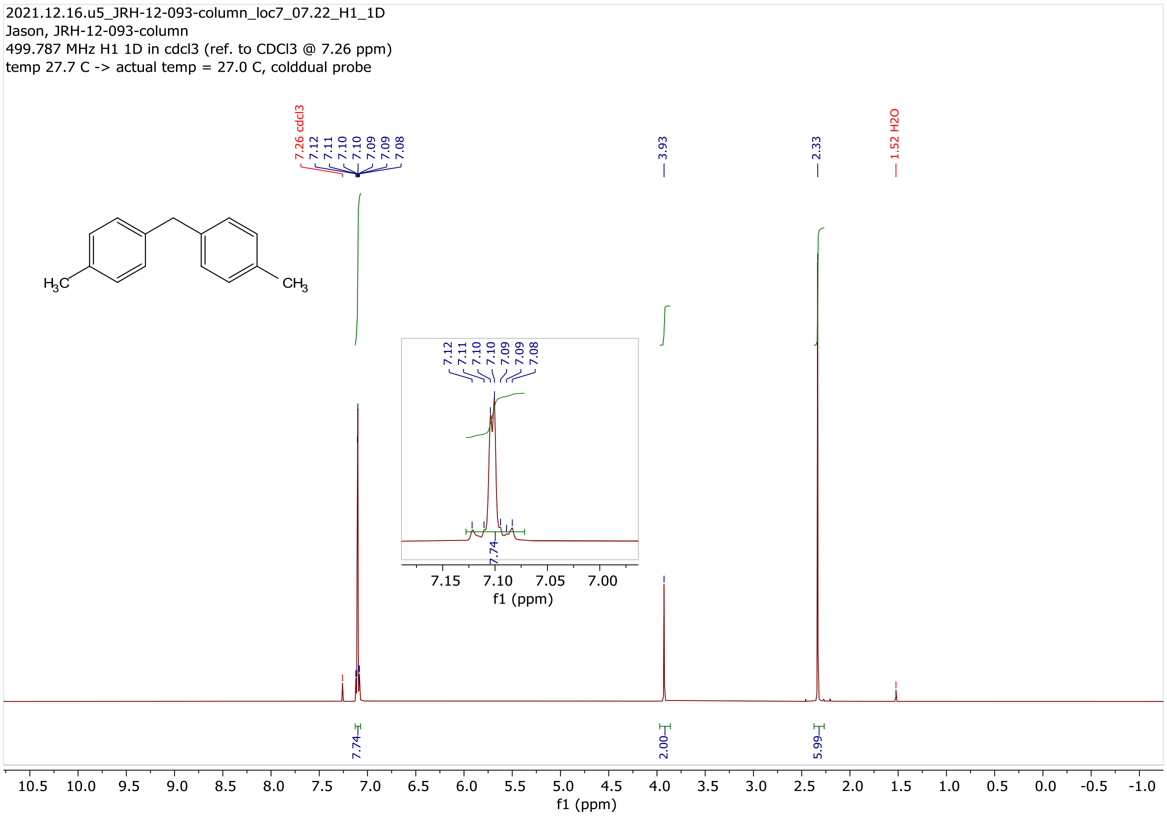

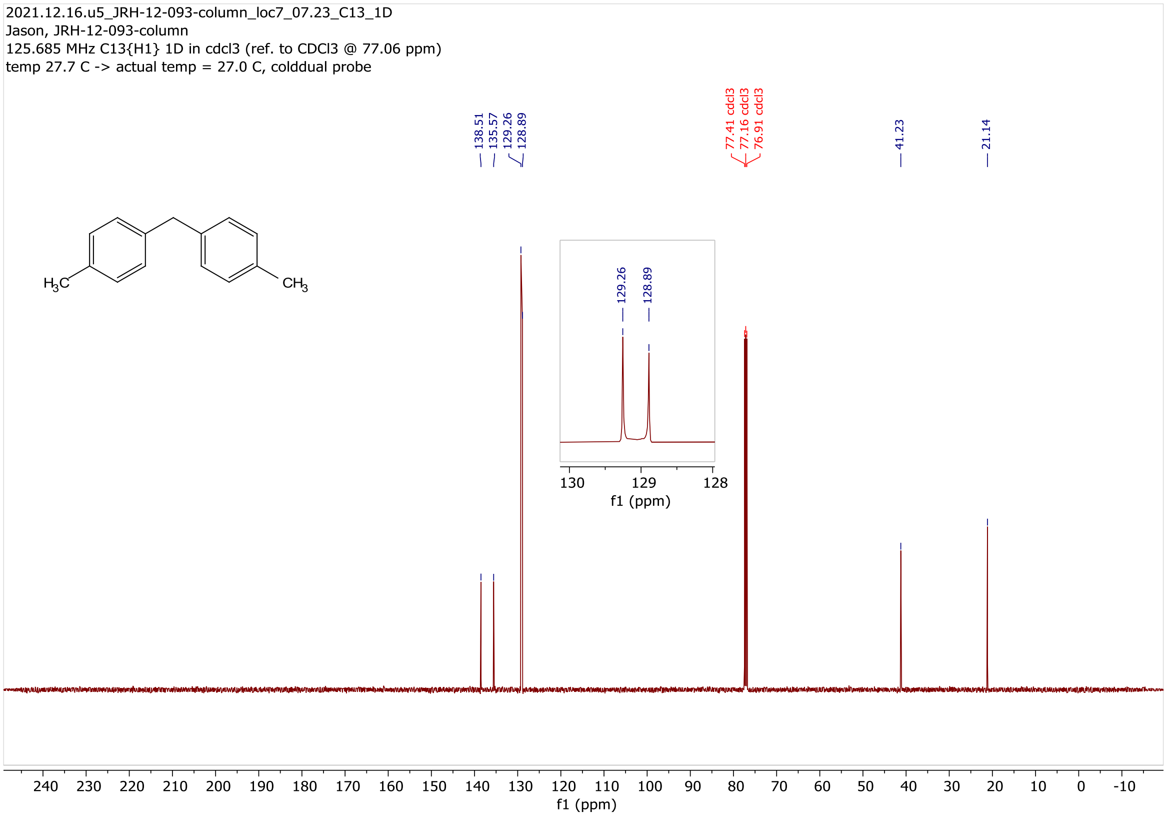

^{1}H (500 MHz) and ^{13}C (126 MHz) NMR of compound 7j ($CDCl_3$)

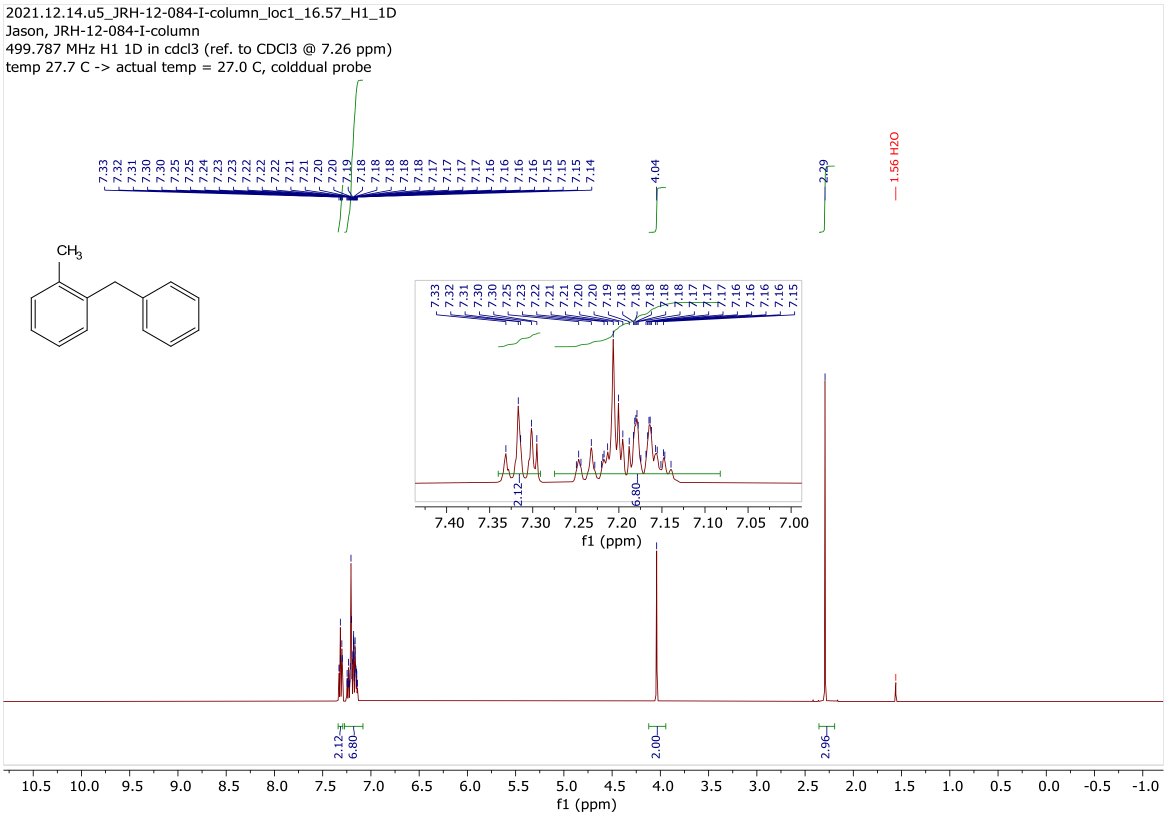

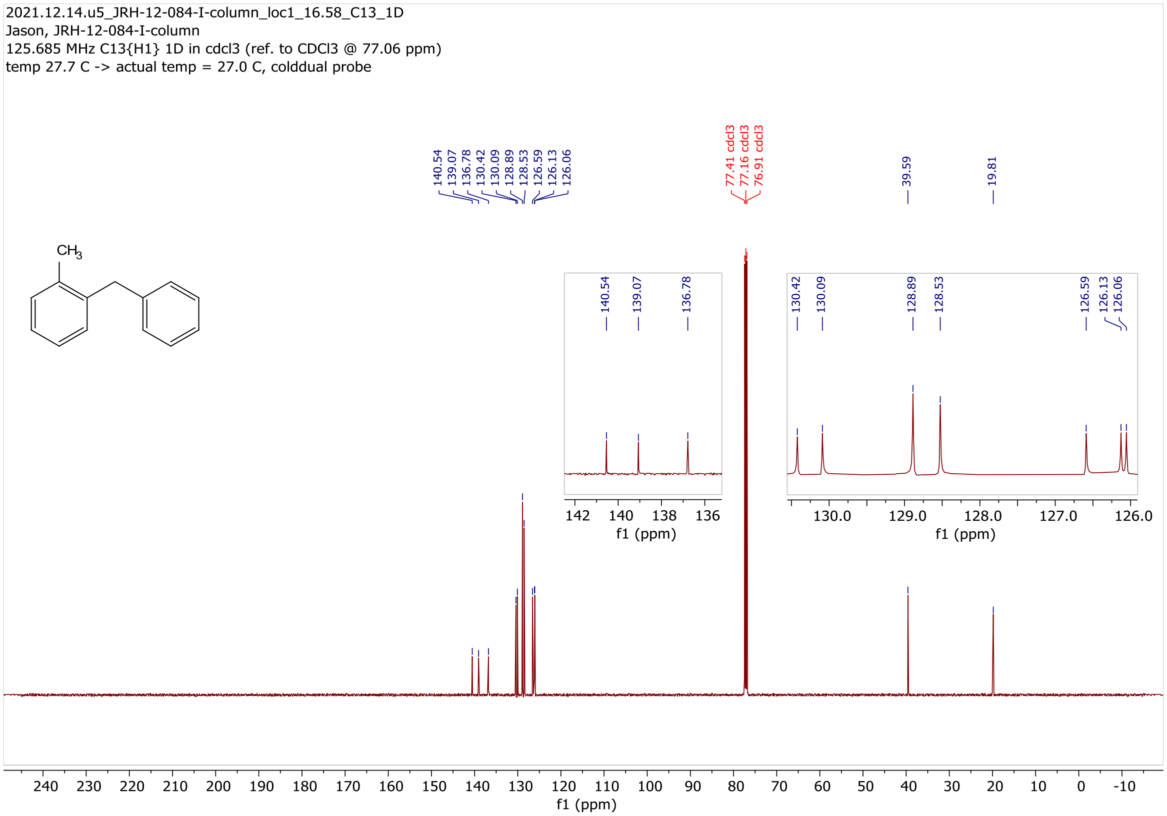

^{1}H (500 MHz) and ^{13}C (126 MHz) NMR of compound 7k ($CDCl_3$)

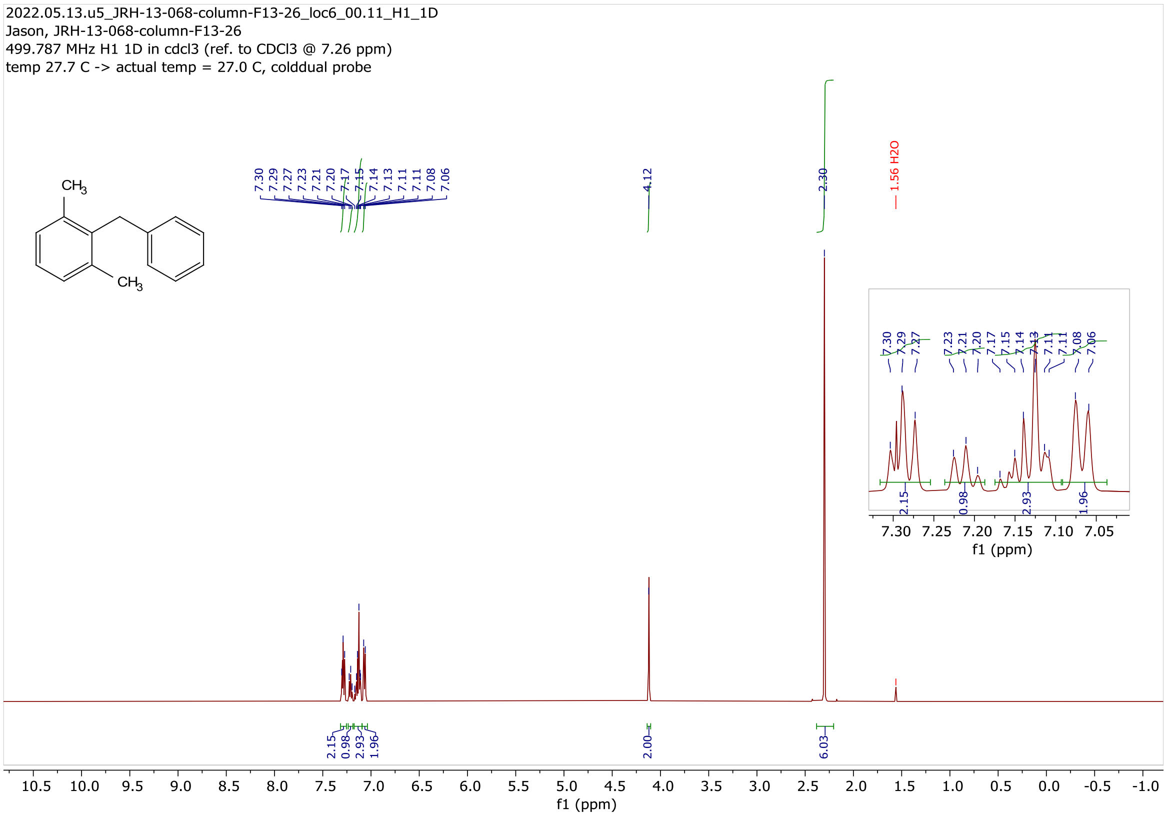

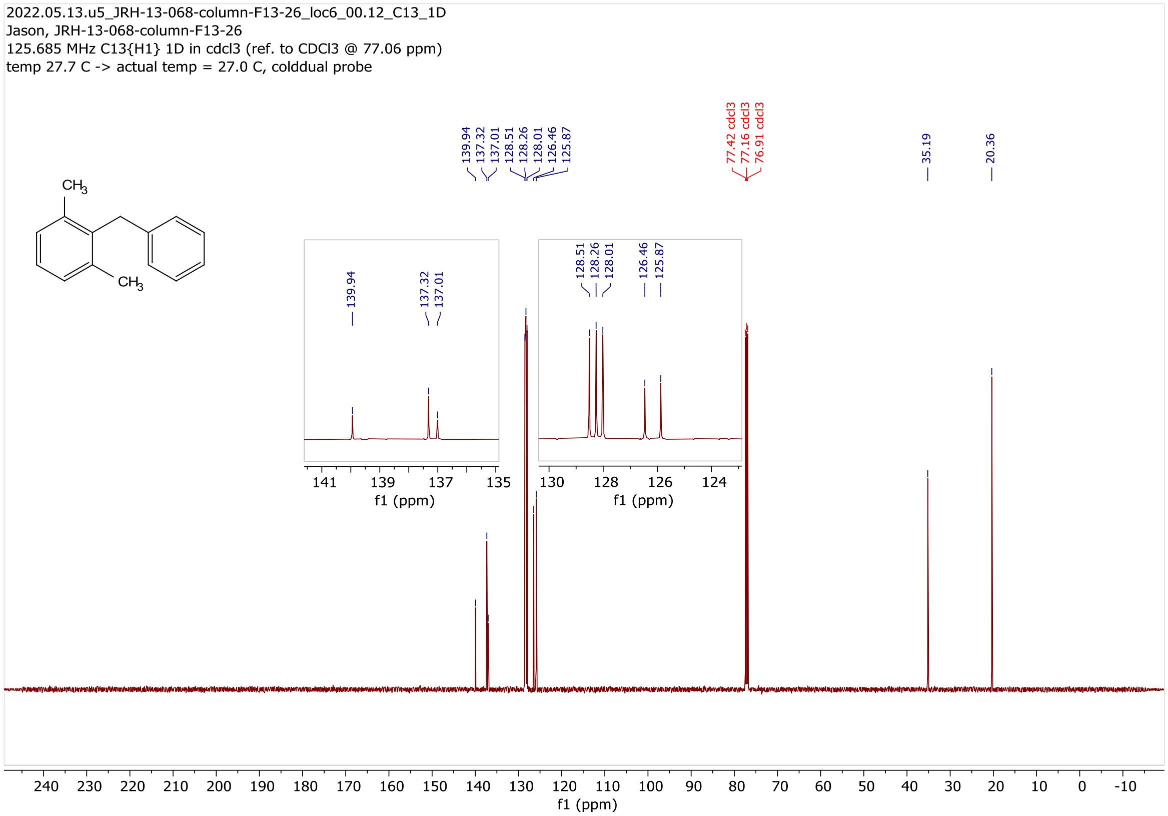

^{1}H (500 MHz) and ^{13}C (126 MHz) NMR of compound 7l ($CDCl_3$)

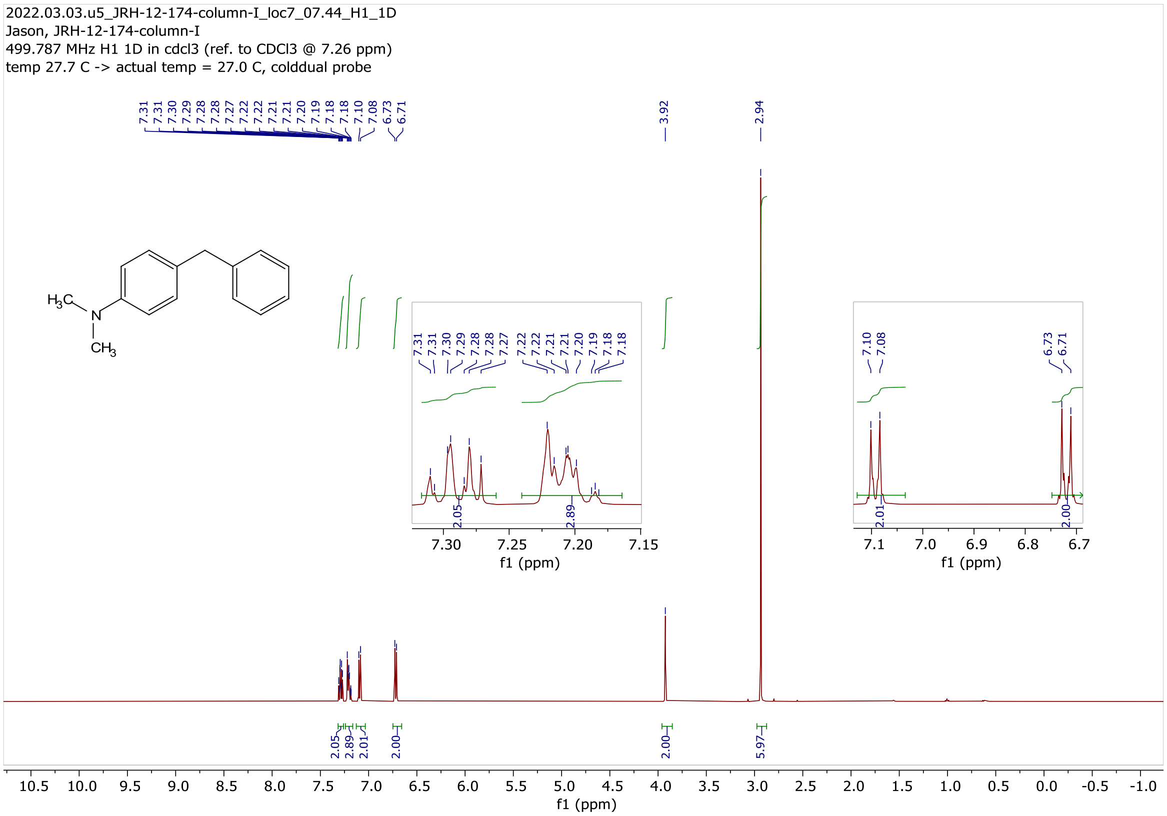

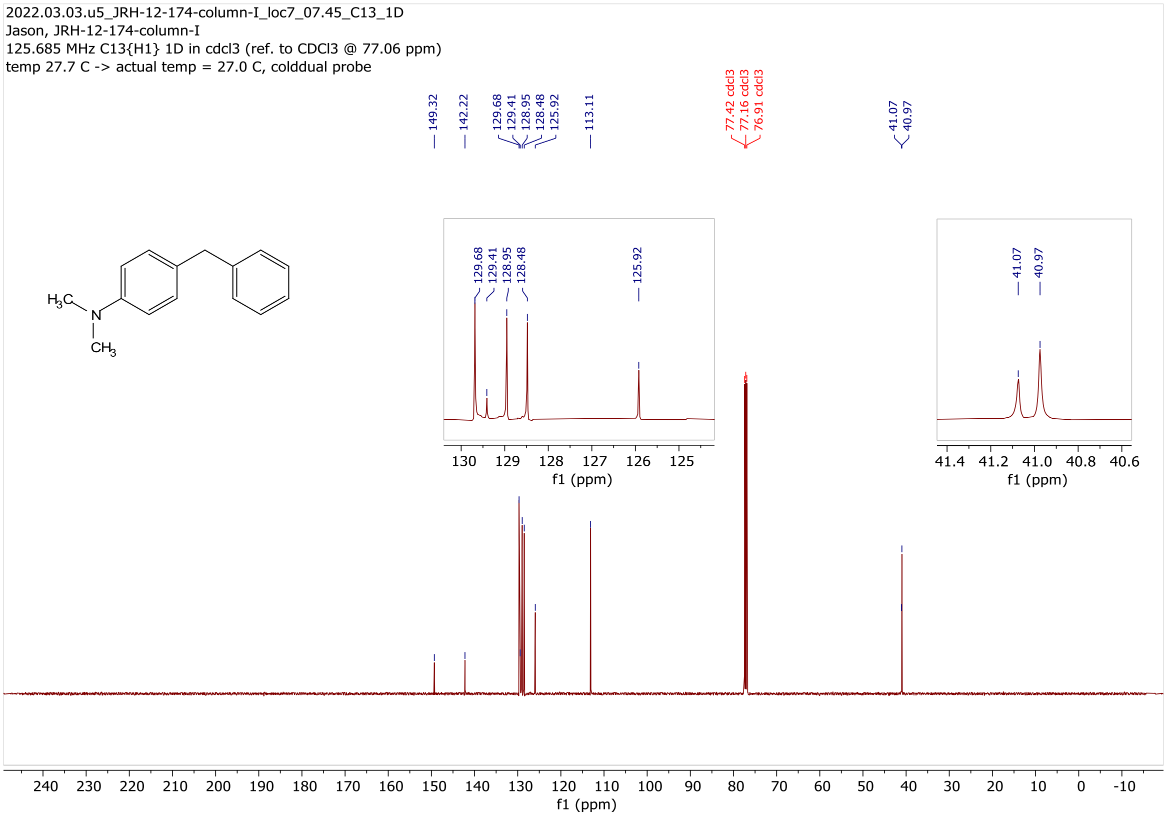

^1H (600 MHz) and ^{13}C (151 MHz) NMR of compound 7m ($CDCl_3$)

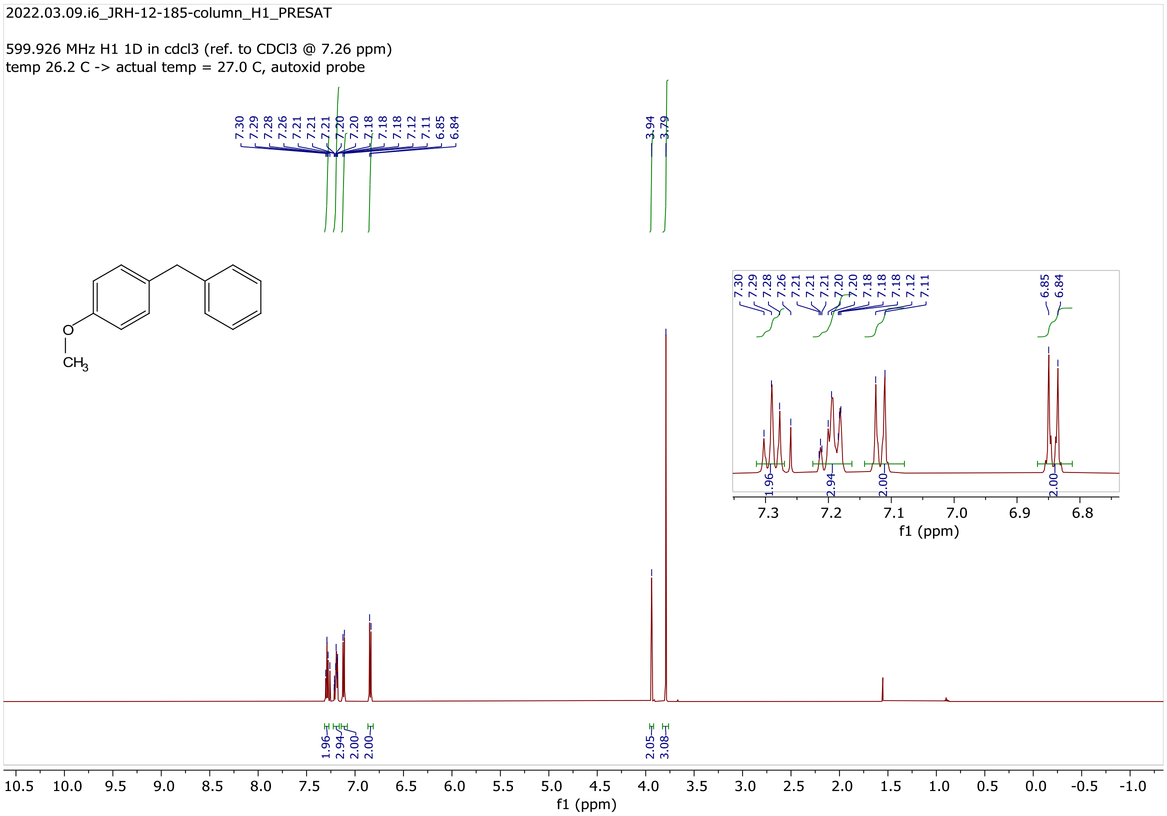

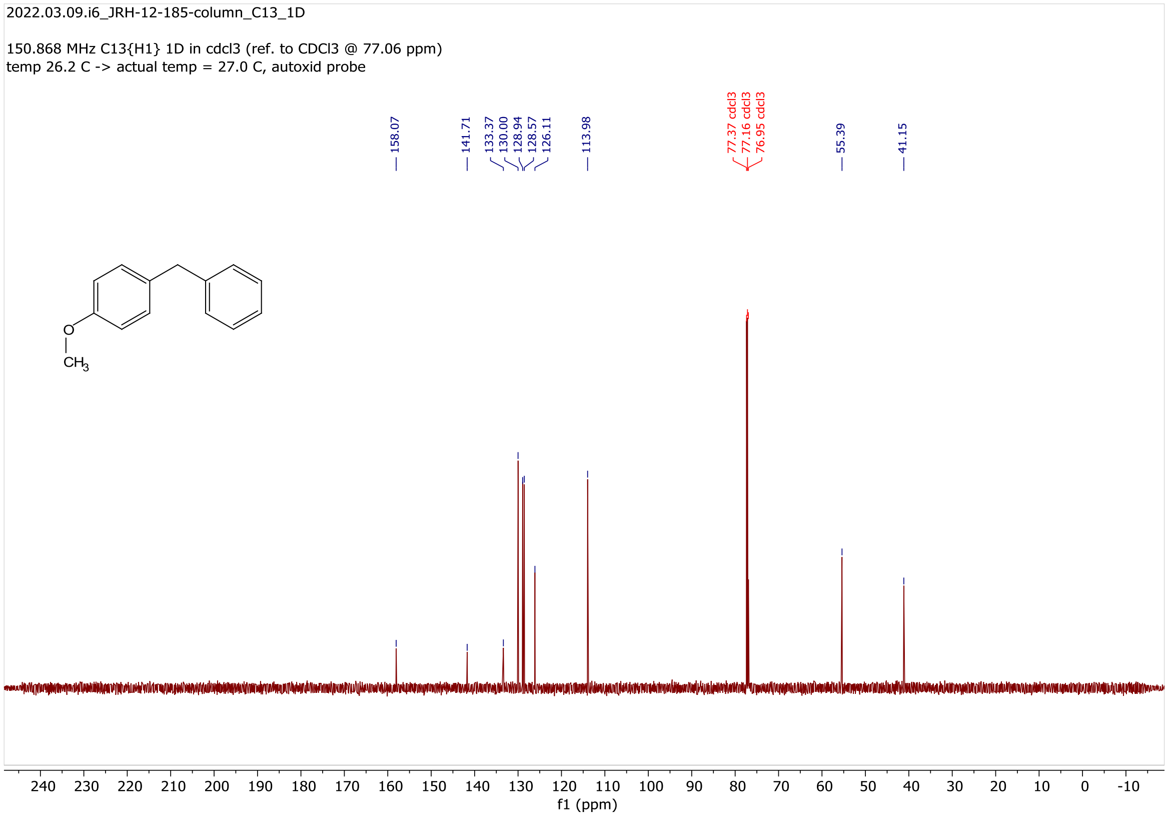

^{1}H (500 MHz) and ^{13}C (126 MHz) NMR of compound 7n ($CDCl_3$)

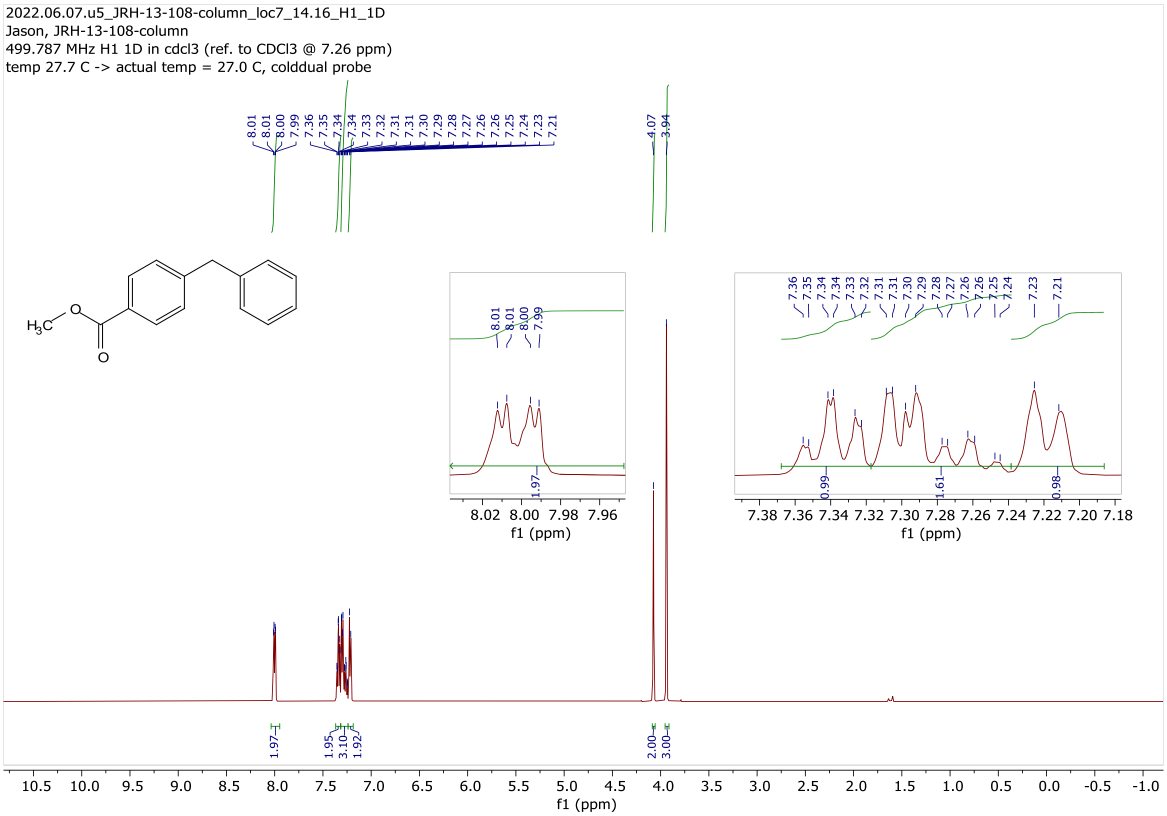

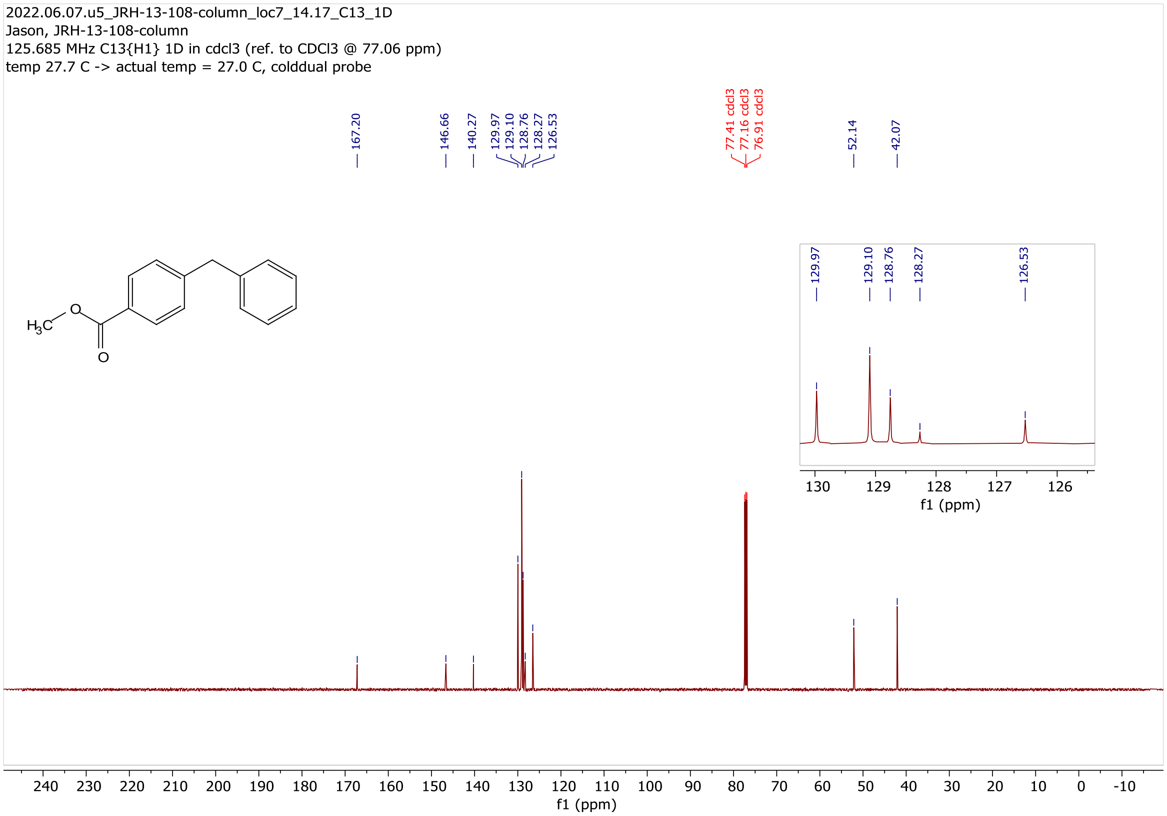

^{1}H (700 MHz) and ^{13}C (176 MHz) NMR of compound 7o ($CDCl_3$)

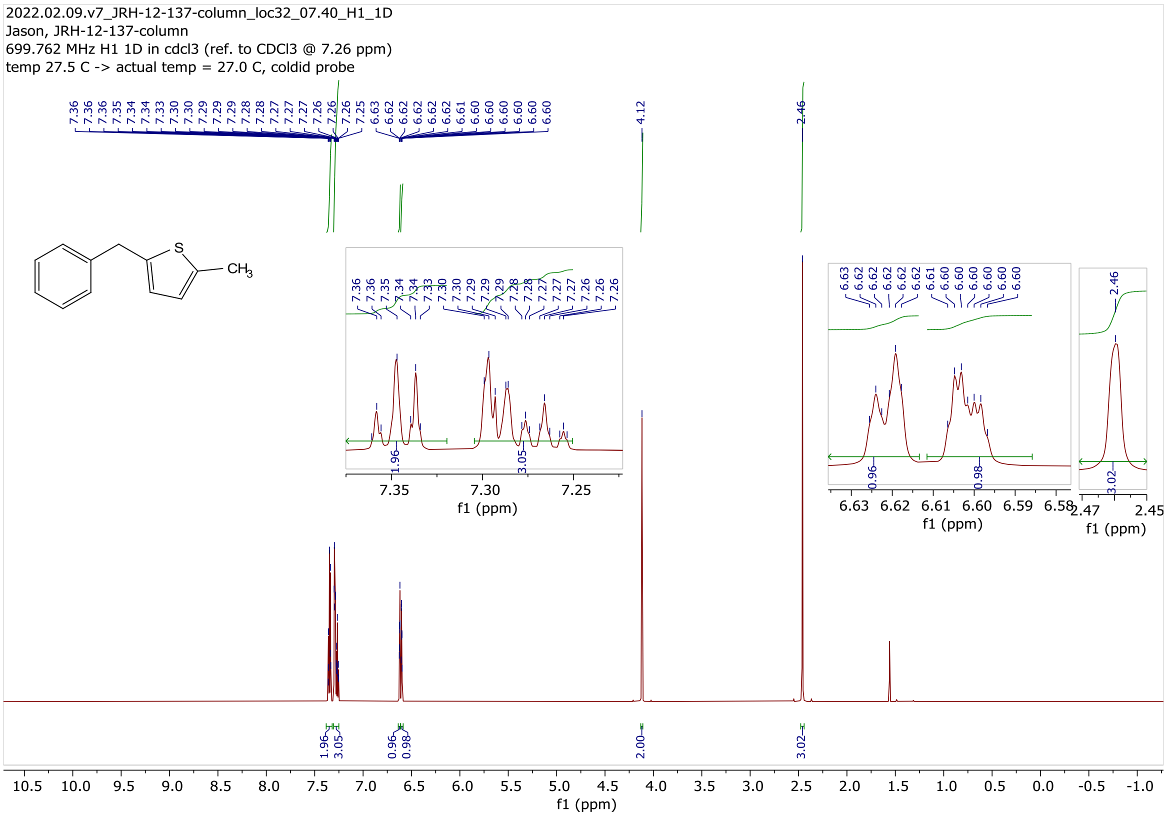

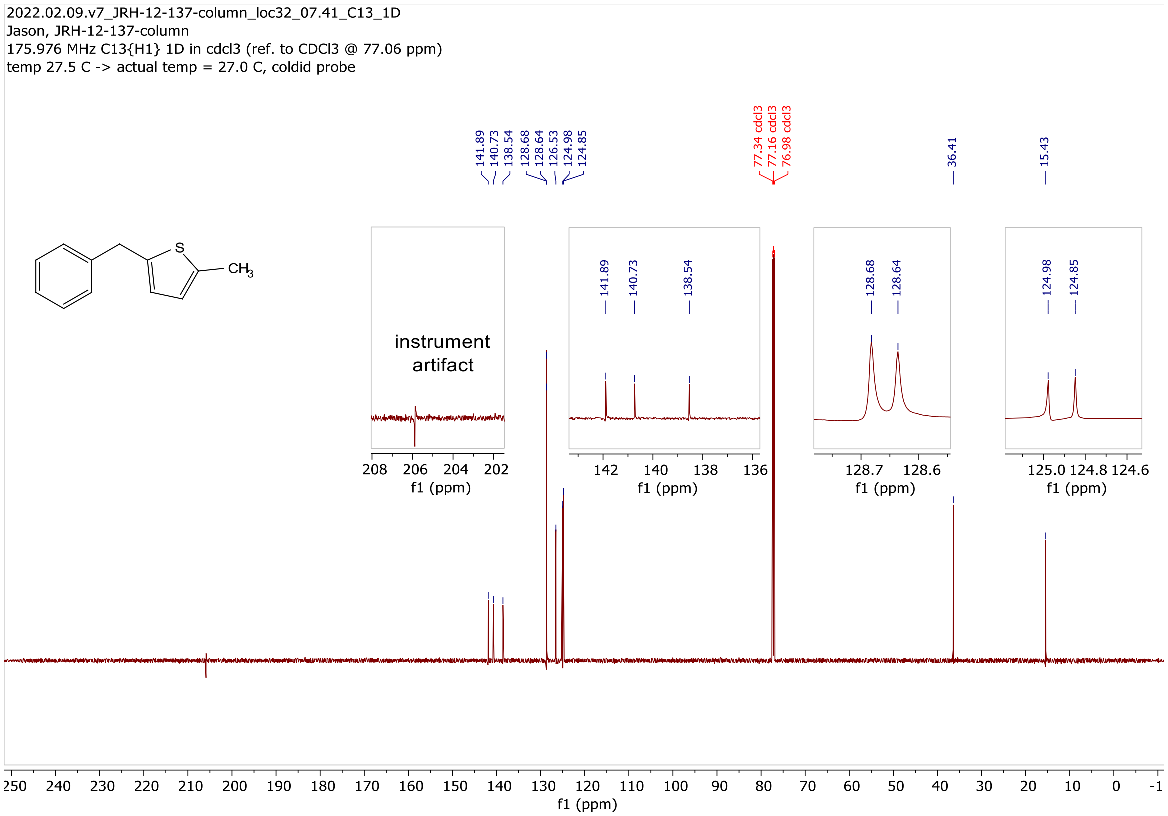

^{1}H (600 MHz) and ^{13}C (151 MHz) NMR of compound 7p ($CDCl_3$)

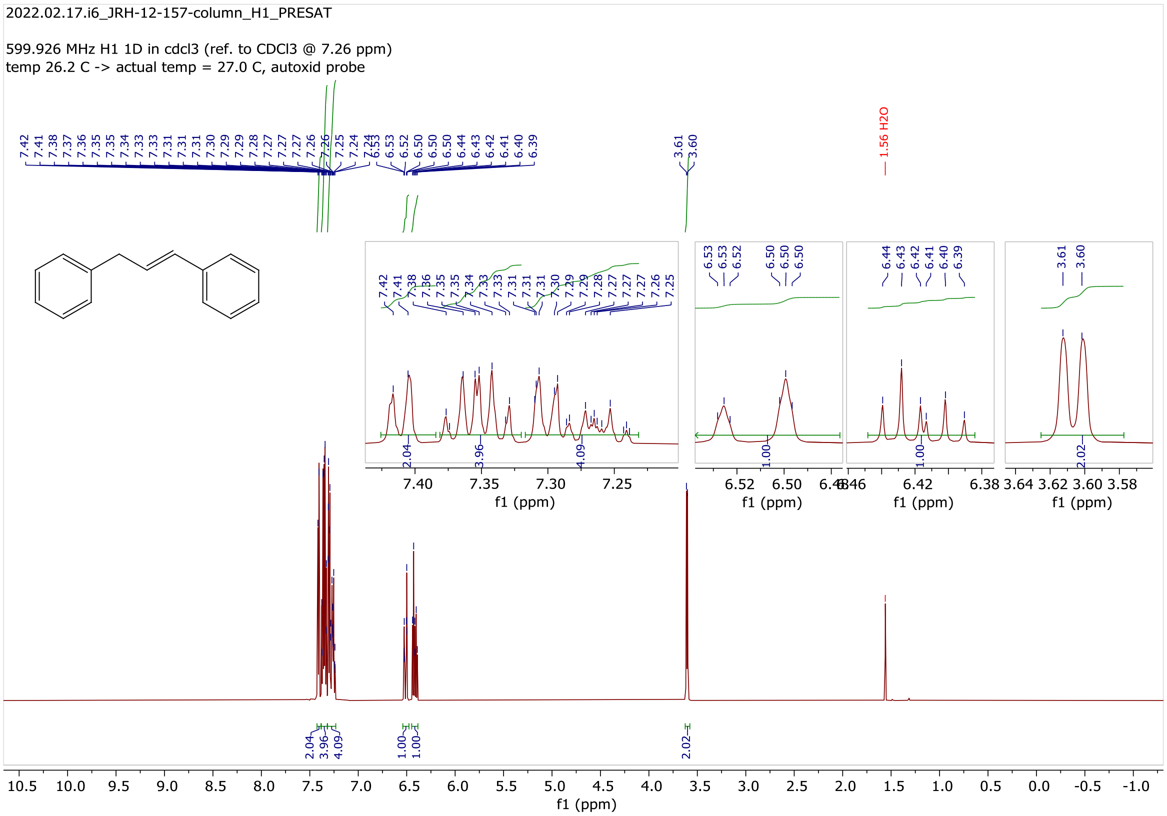

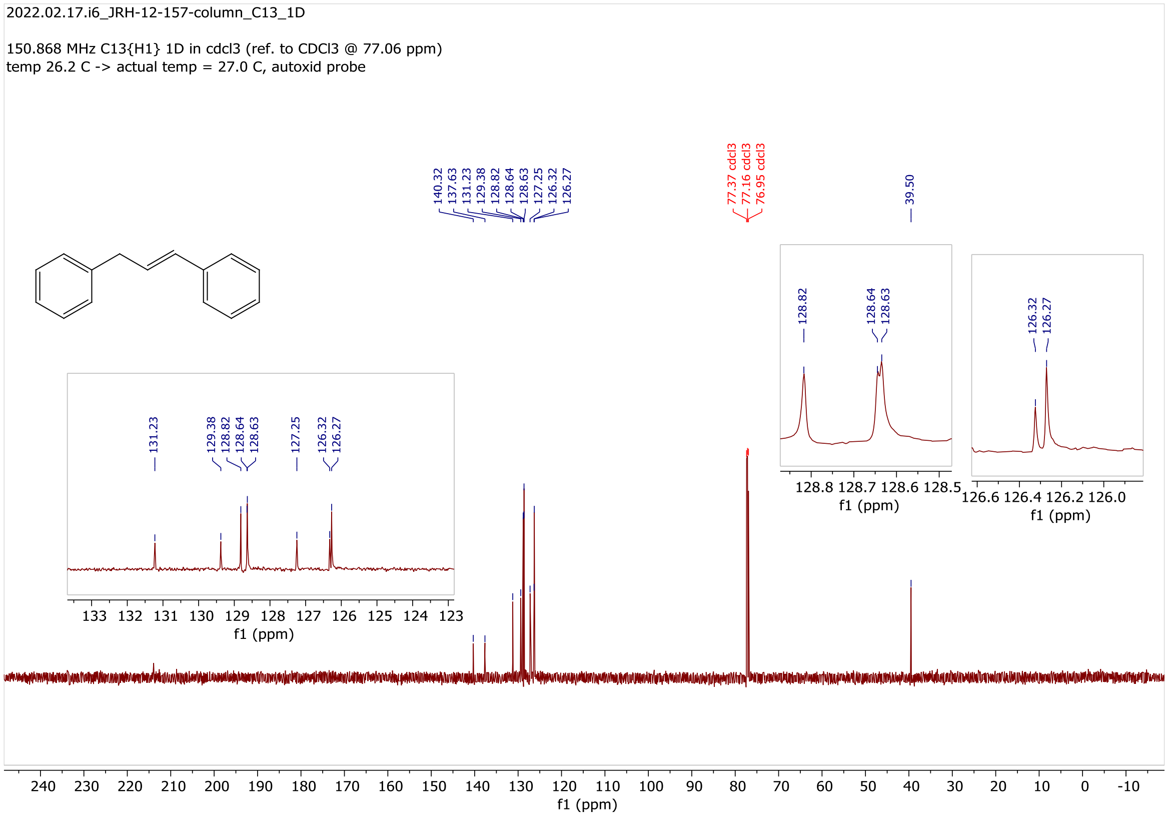

^{1}H (500 MHz) and ^{13}C (126 MHz) NMR of compound 7q ($CDCl_3$)

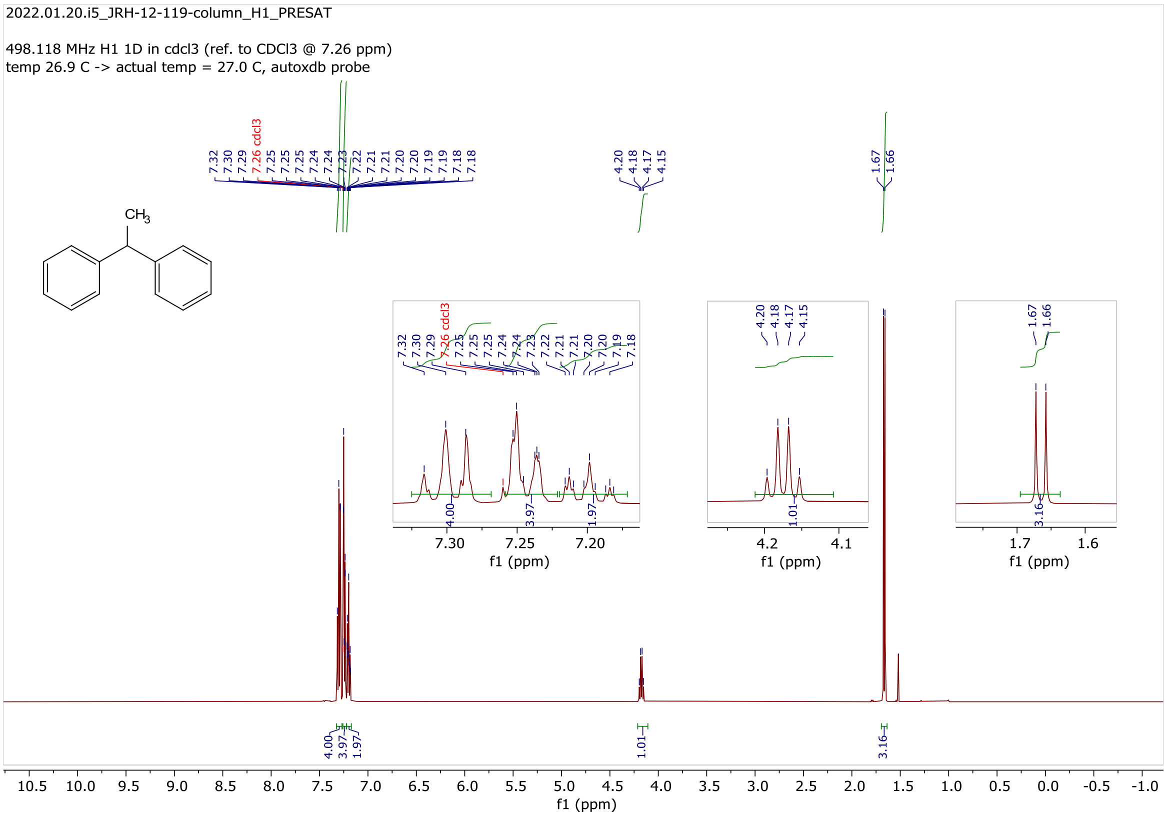

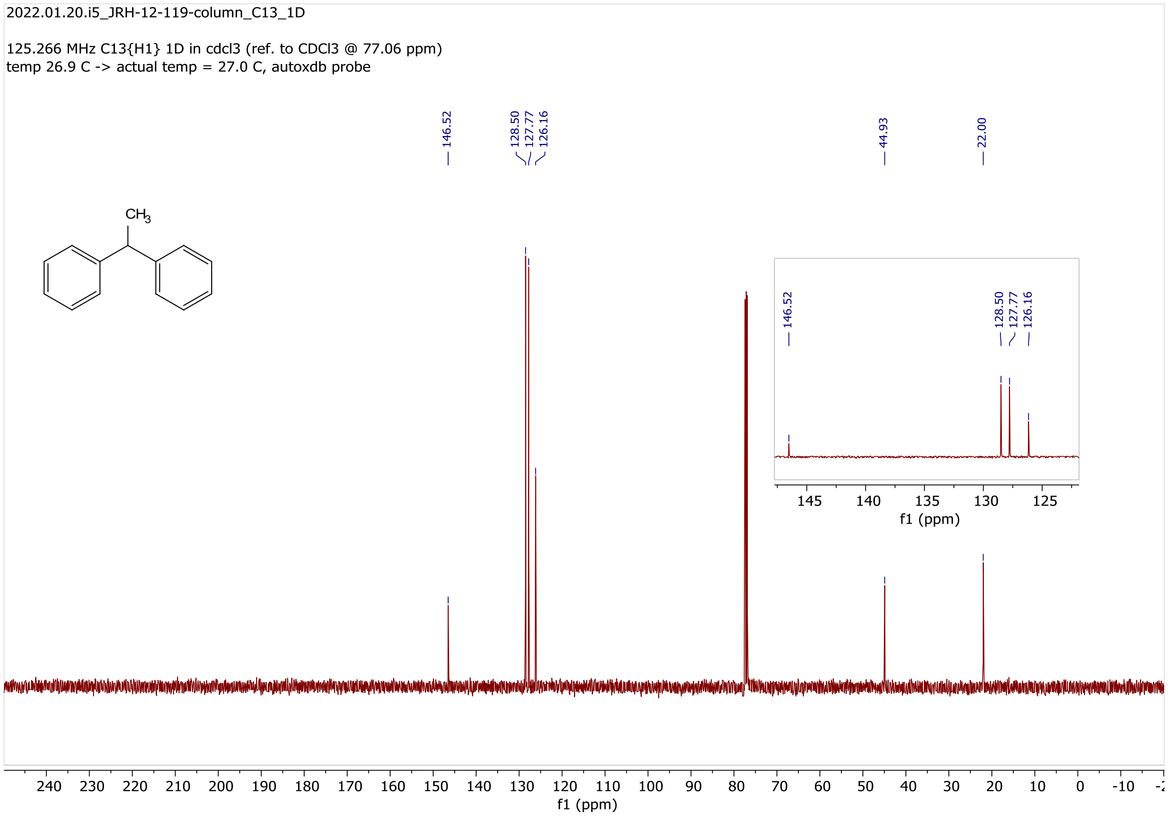

^{1}H (500 MHz) and ^{13}C (126 MHz) NMR of compound 7r ($CDCl_3$)

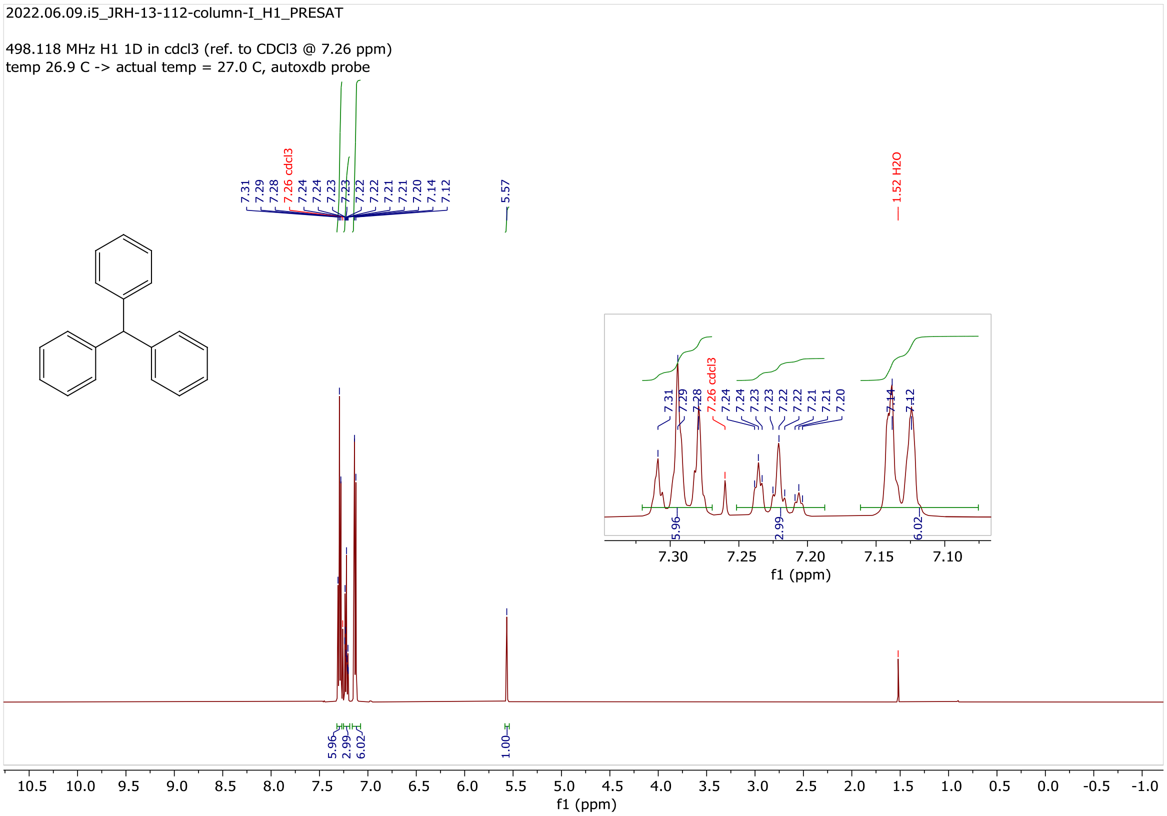

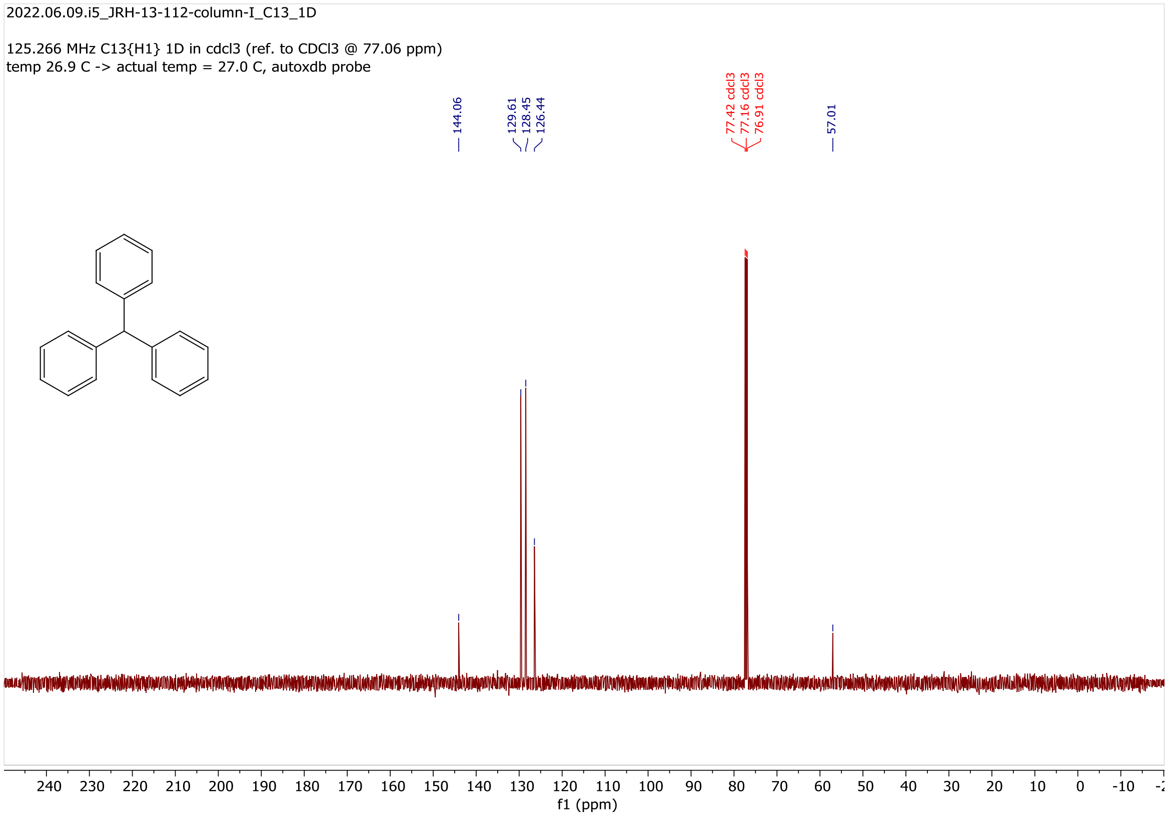

^{1}H (600 MHz) and ^{13}C (151 MHz) NMR of compound 7s ($CDCl_3$)

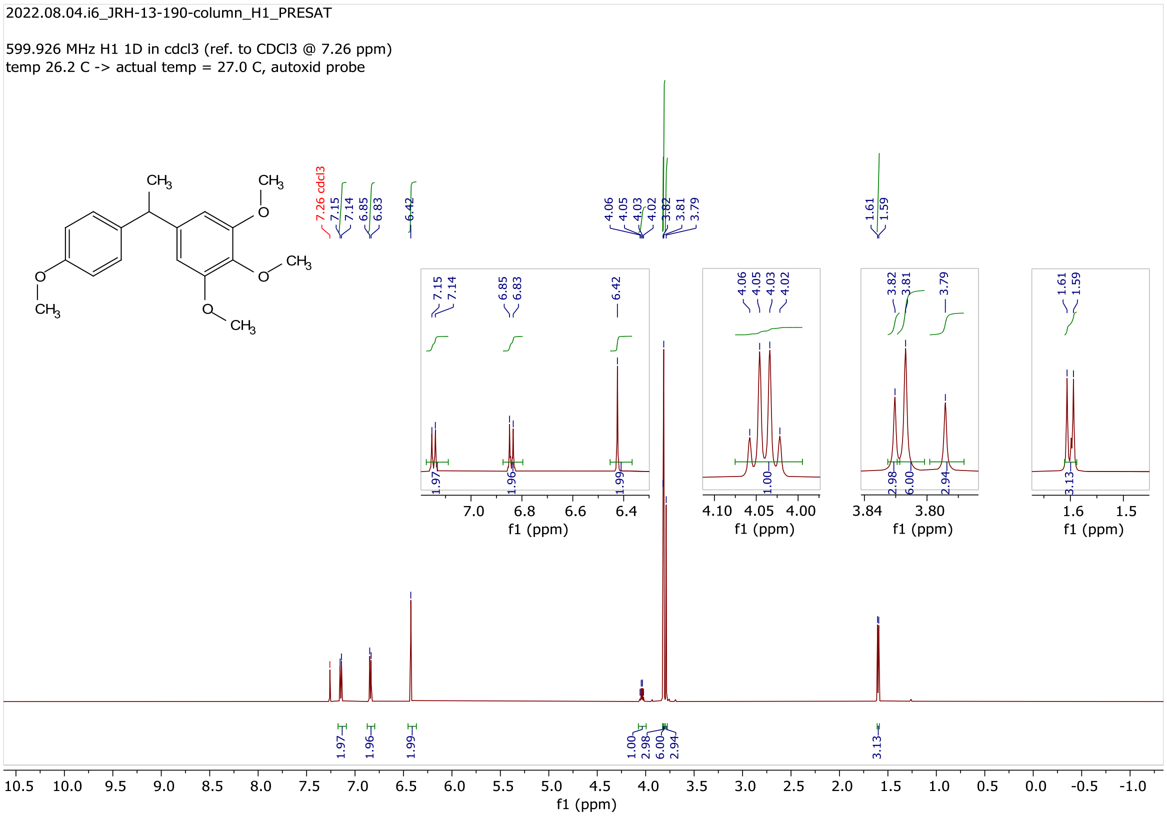

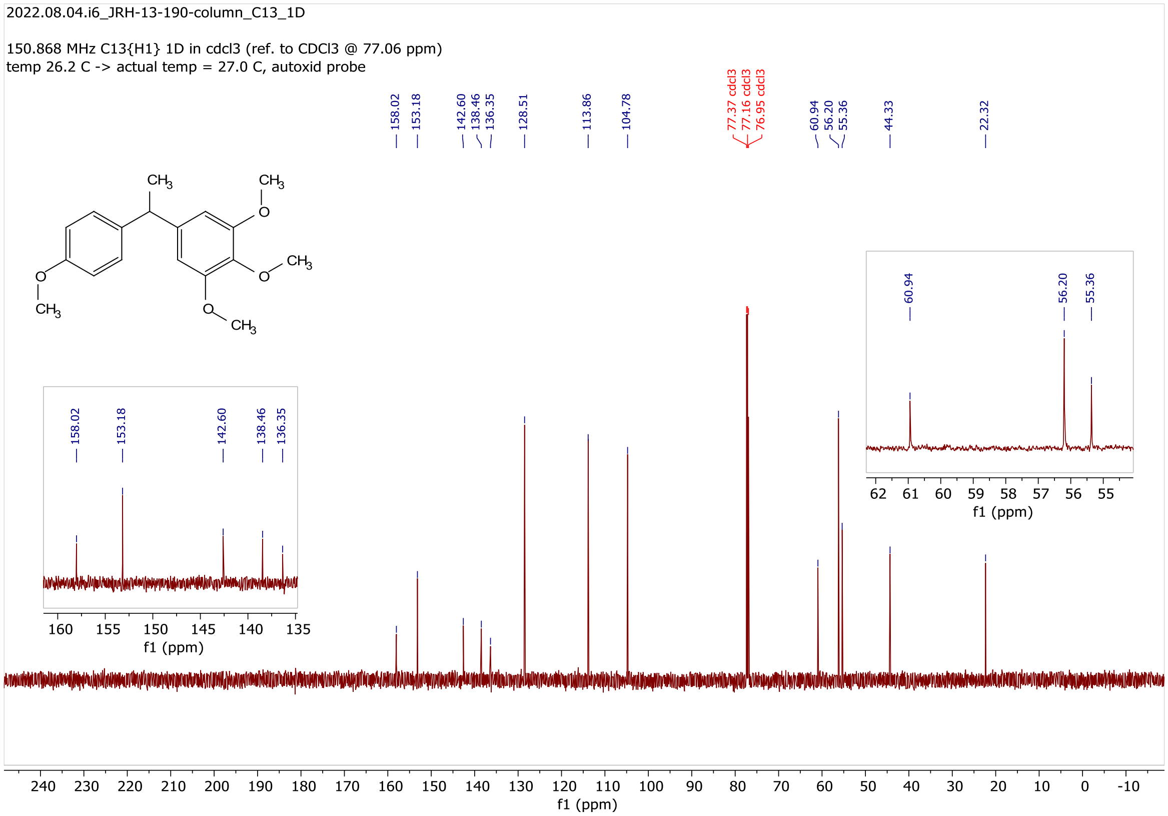

^{1}H (500 MHz) and ^{13}C (126 MHz) NMR of compound 7t ($CDCl_3$)

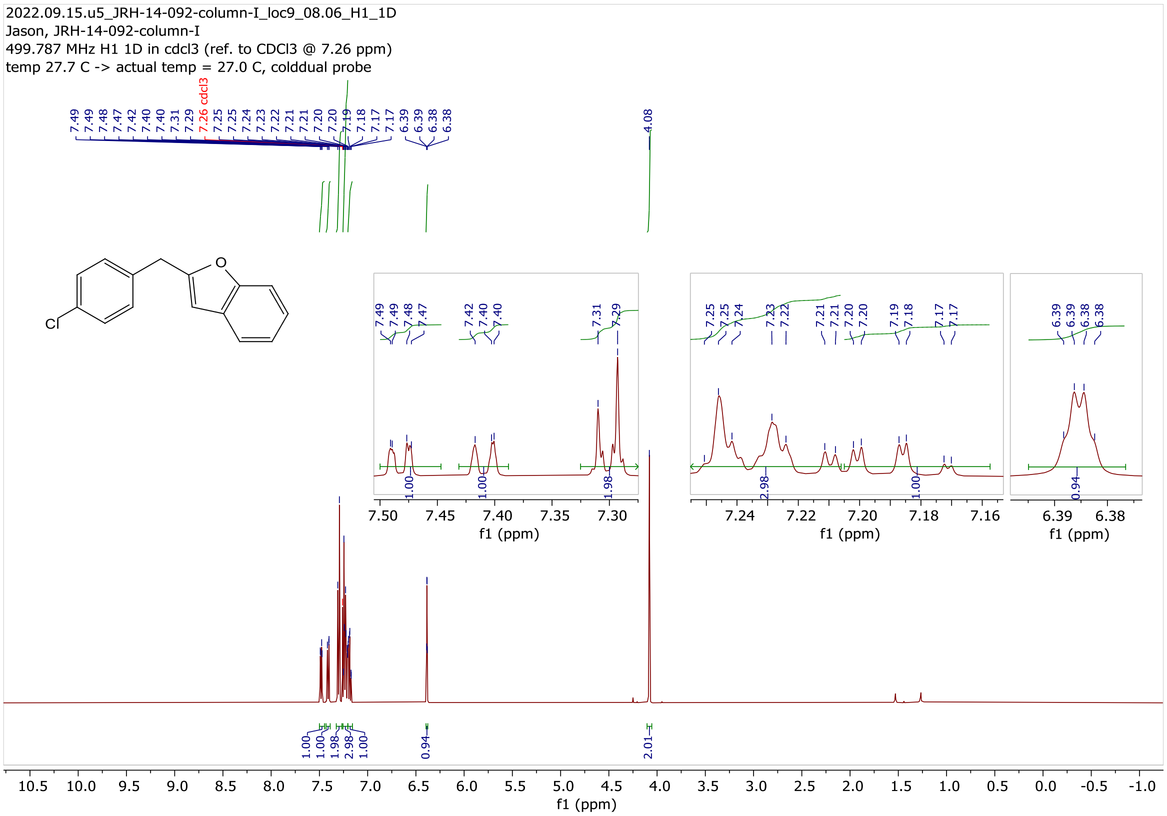

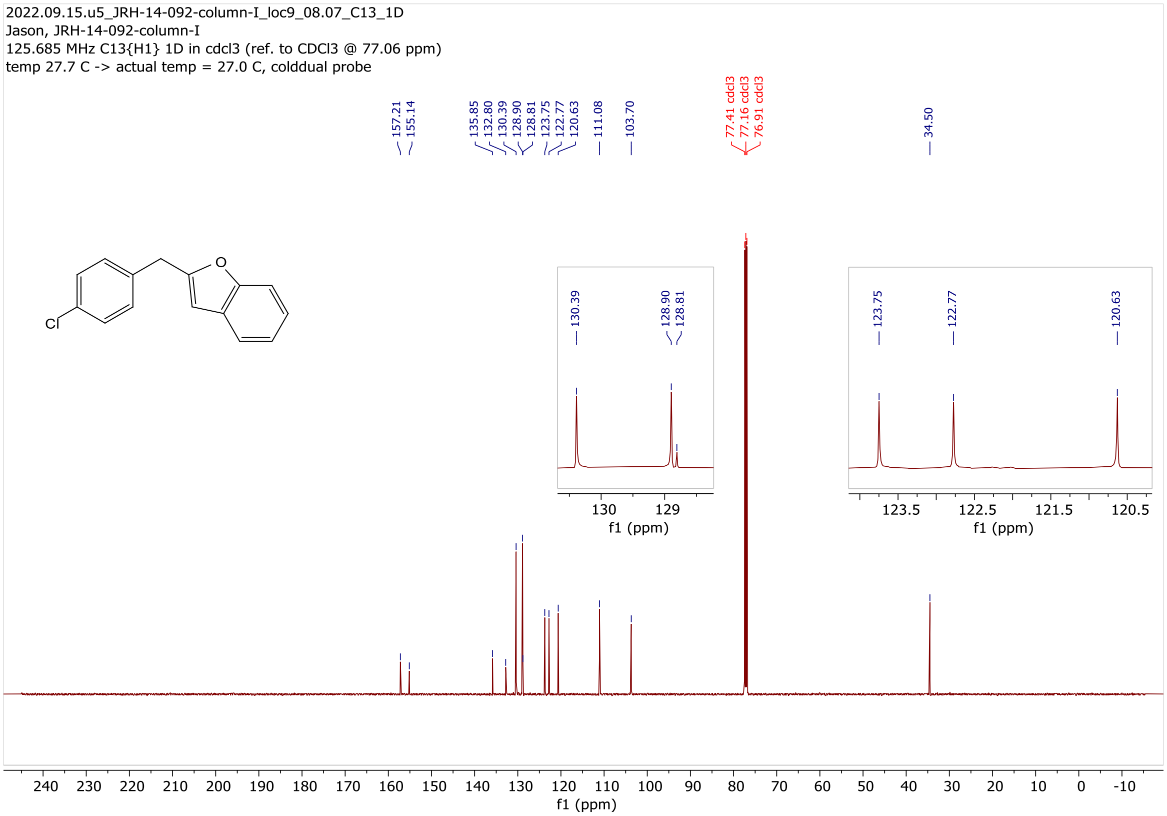

^{1}H (500 MHz) and ^{13}C (126 MHz) NMR of compound 7u ($CDCl_3$)

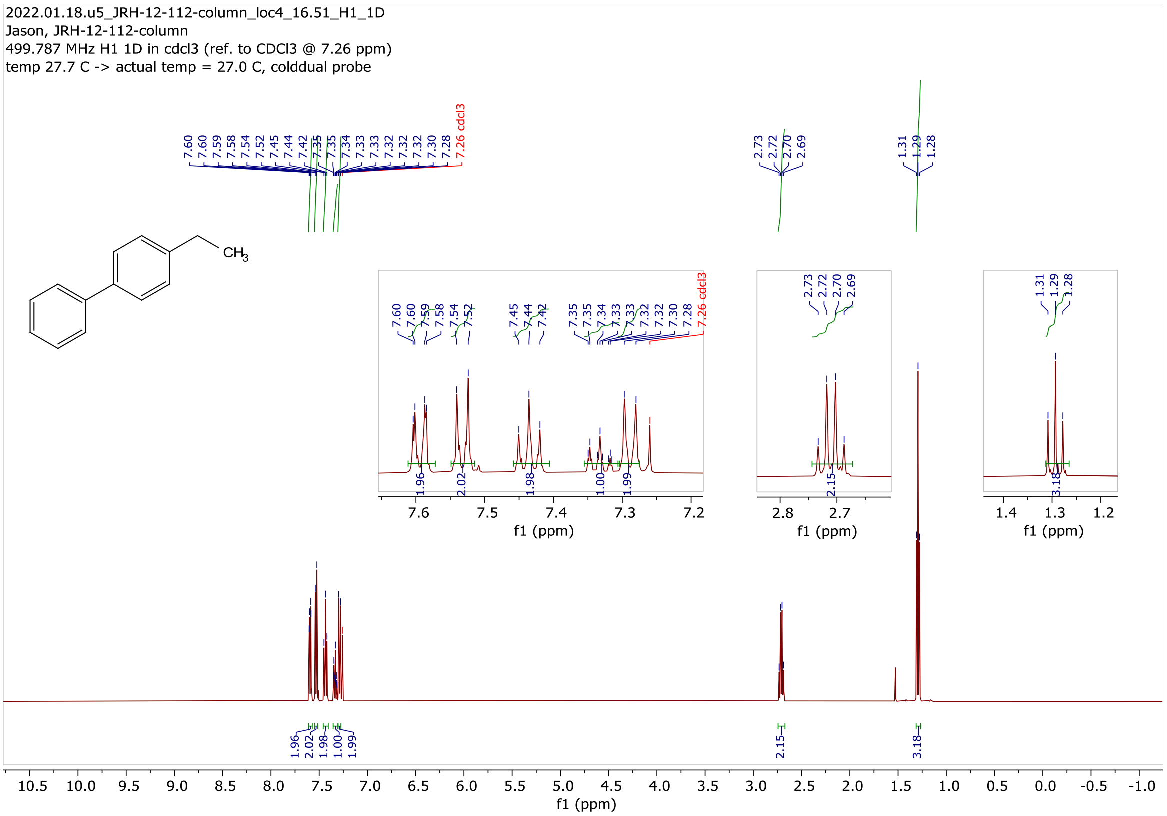

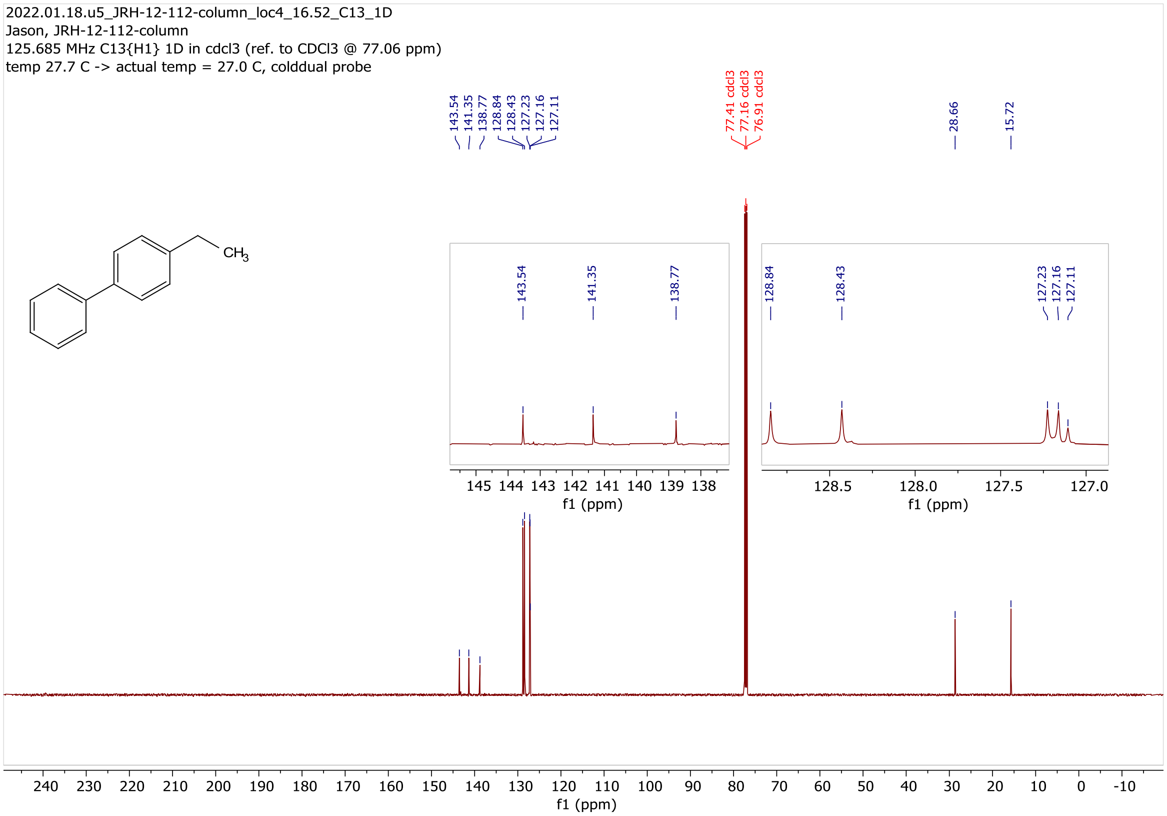

^{1}H (500 MHz) and ^{13}C (126 MHz) NMR of compound 7v ($CDCl_3$)

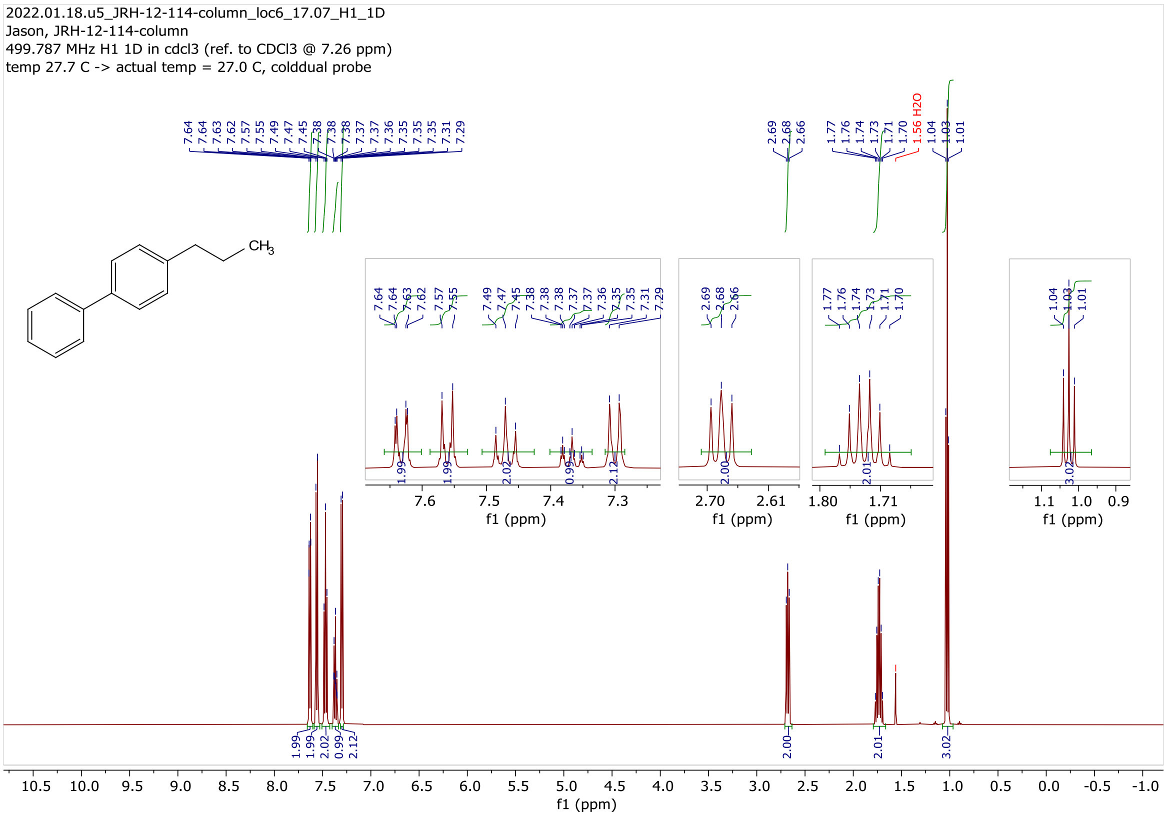

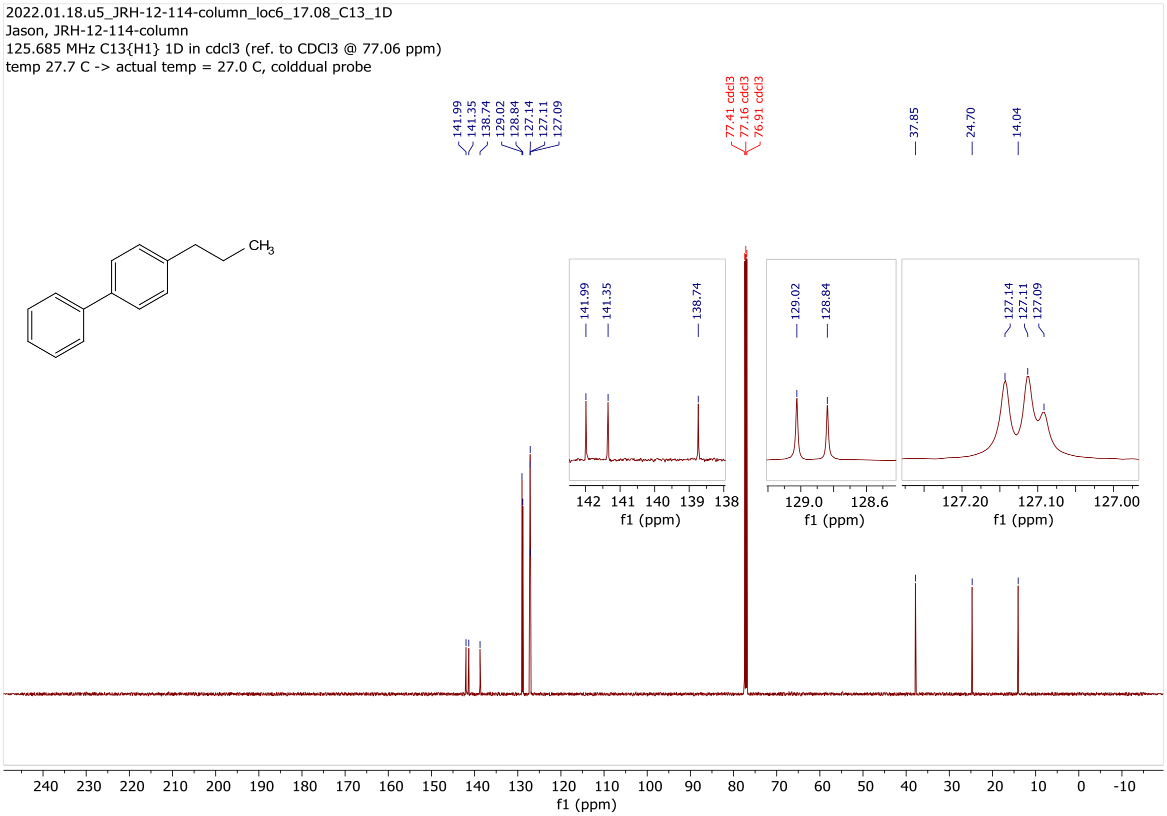

^{1}H (500 MHz) and ^{13}C (126 MHz) NMR of compound 7w ($CDCl_3$)

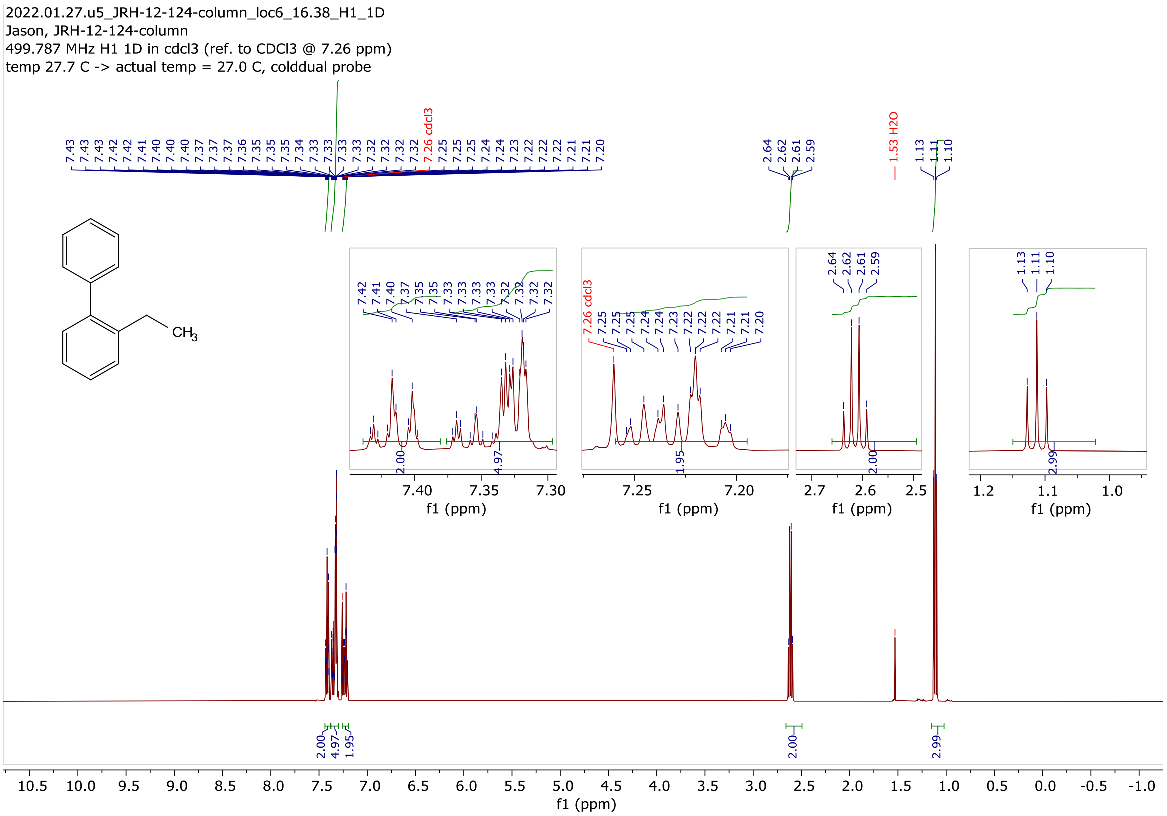

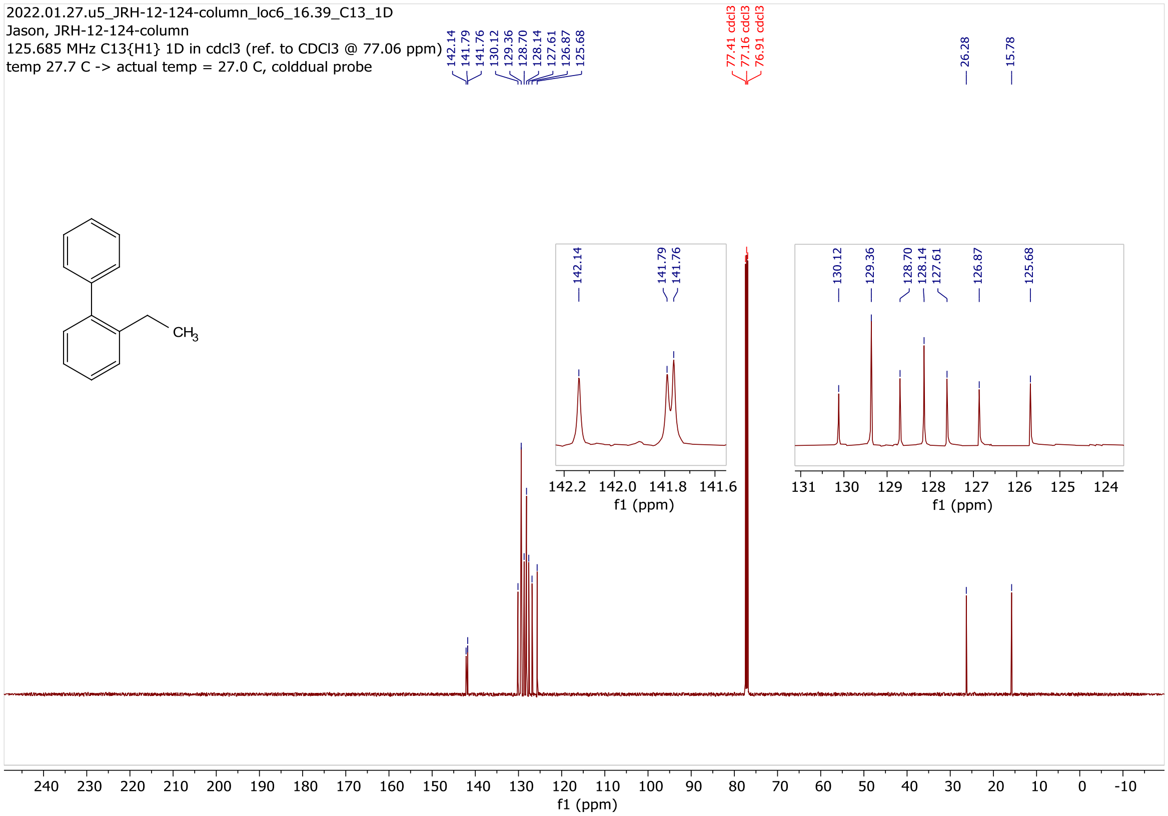

^{1}H (500 MHz) and ^{13}C (126 MHz) NMR of compound 7x ($CDCl_3$)

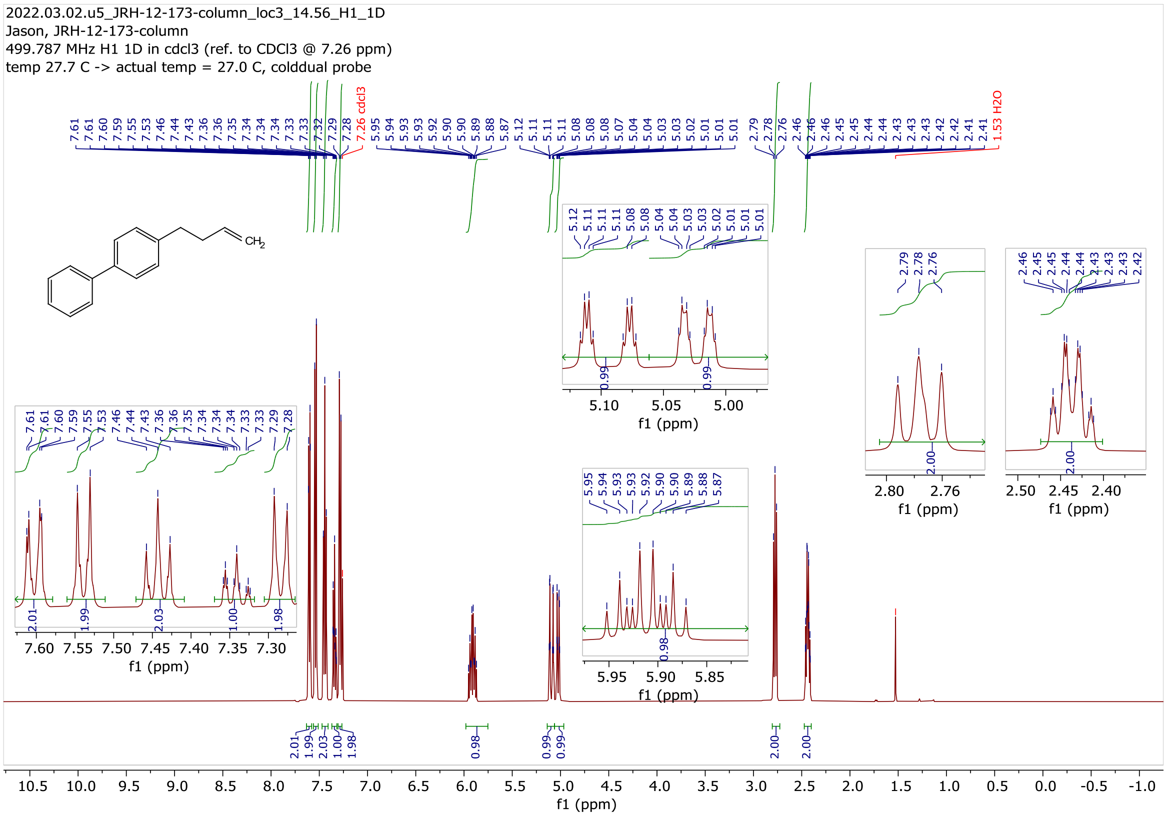

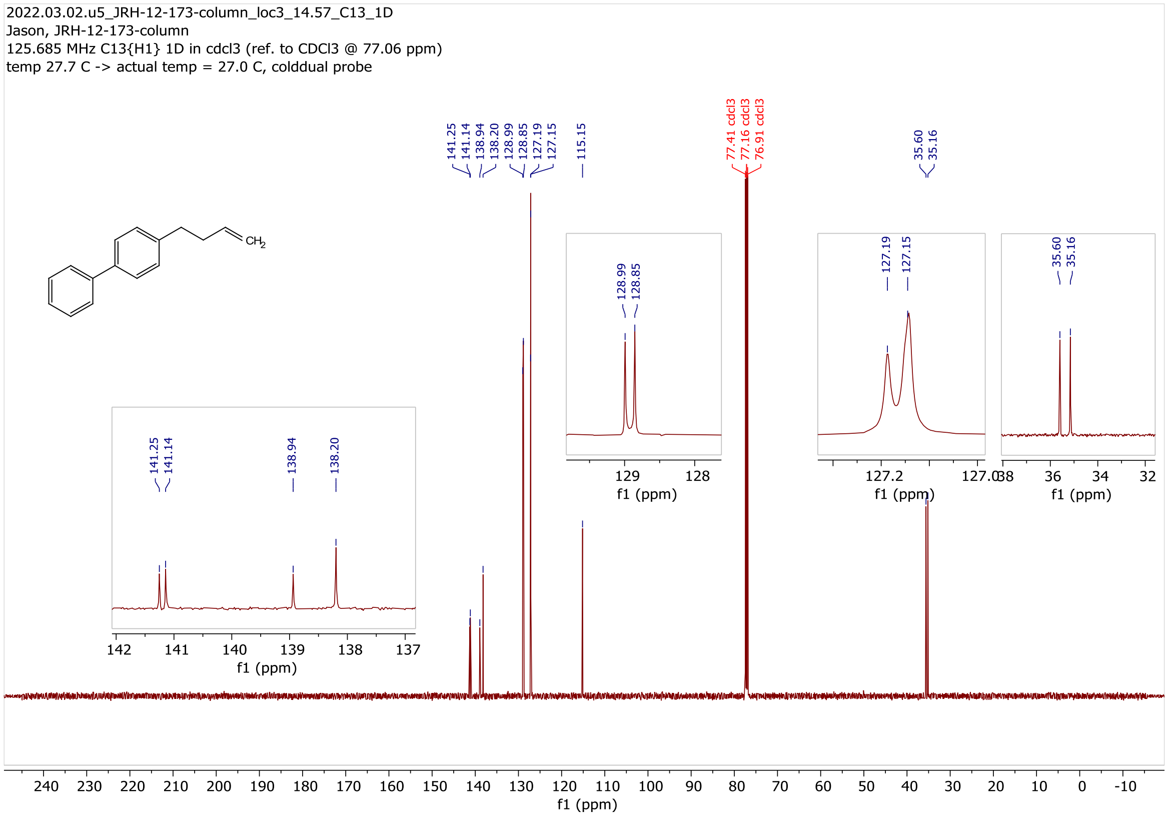

^{1}H (700 MHz) and ^{13}C (126 MHz) NMR of compound 7y ($CDCl_3$)

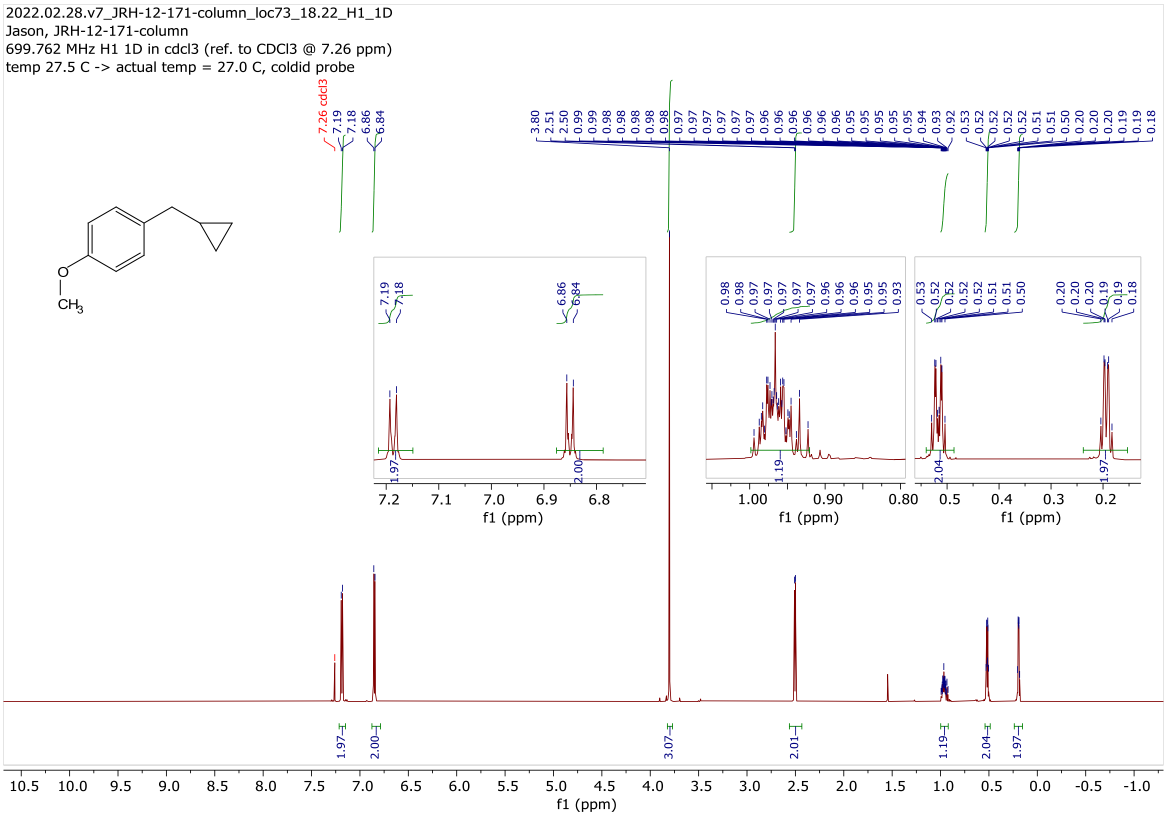

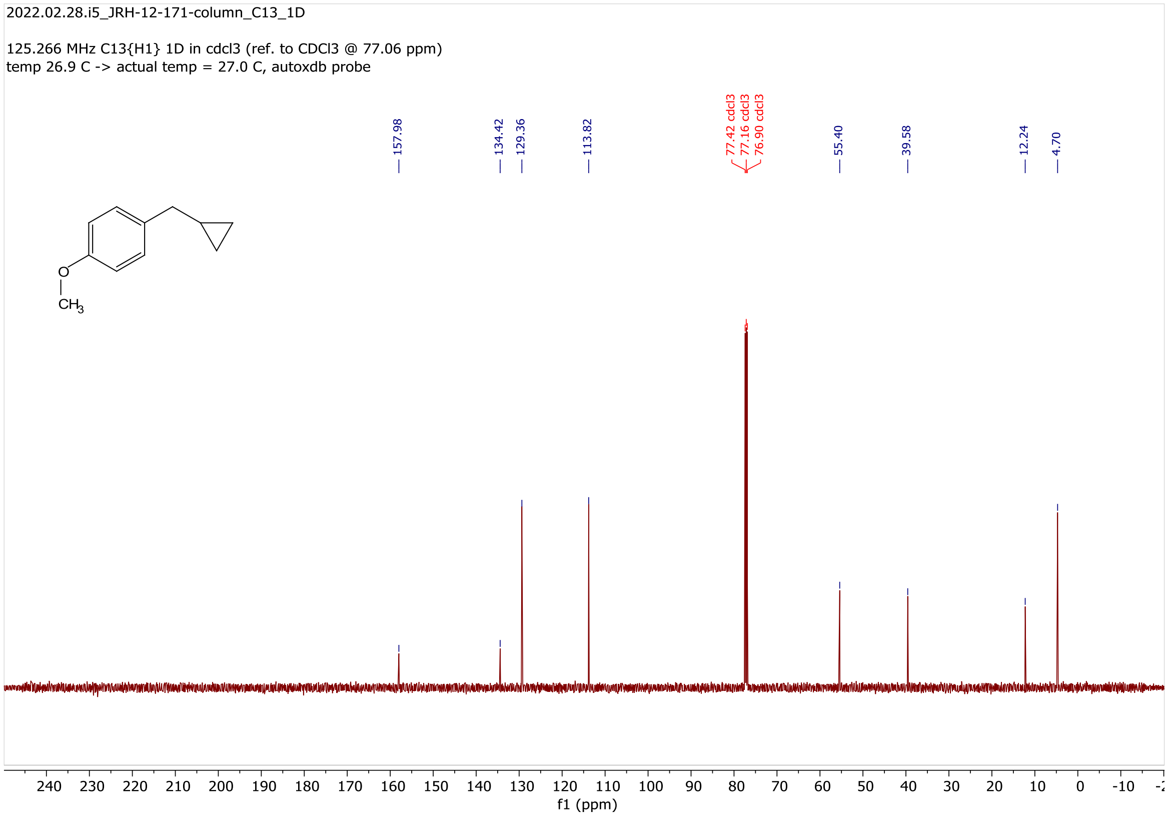

^{1}H (500 MHz) and ^{13}C (126 MHz) NMR of compound 7z ($CDCl_3$)

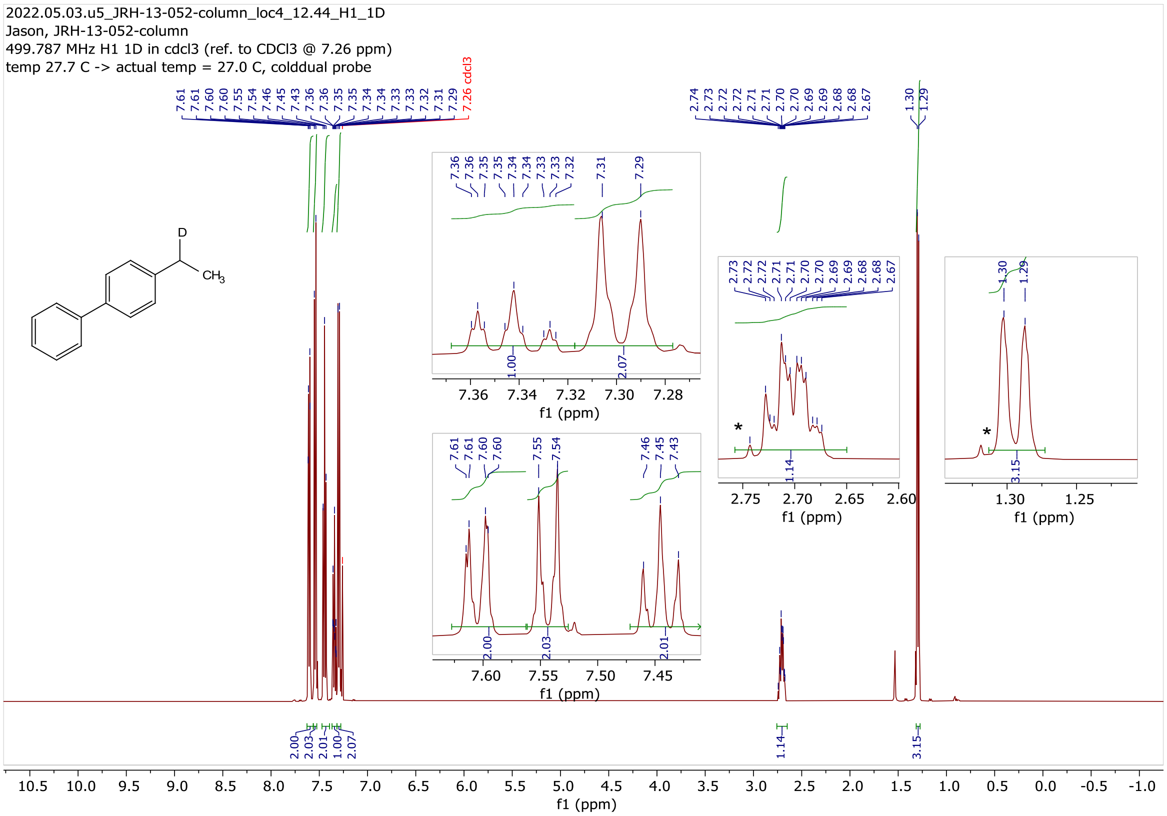

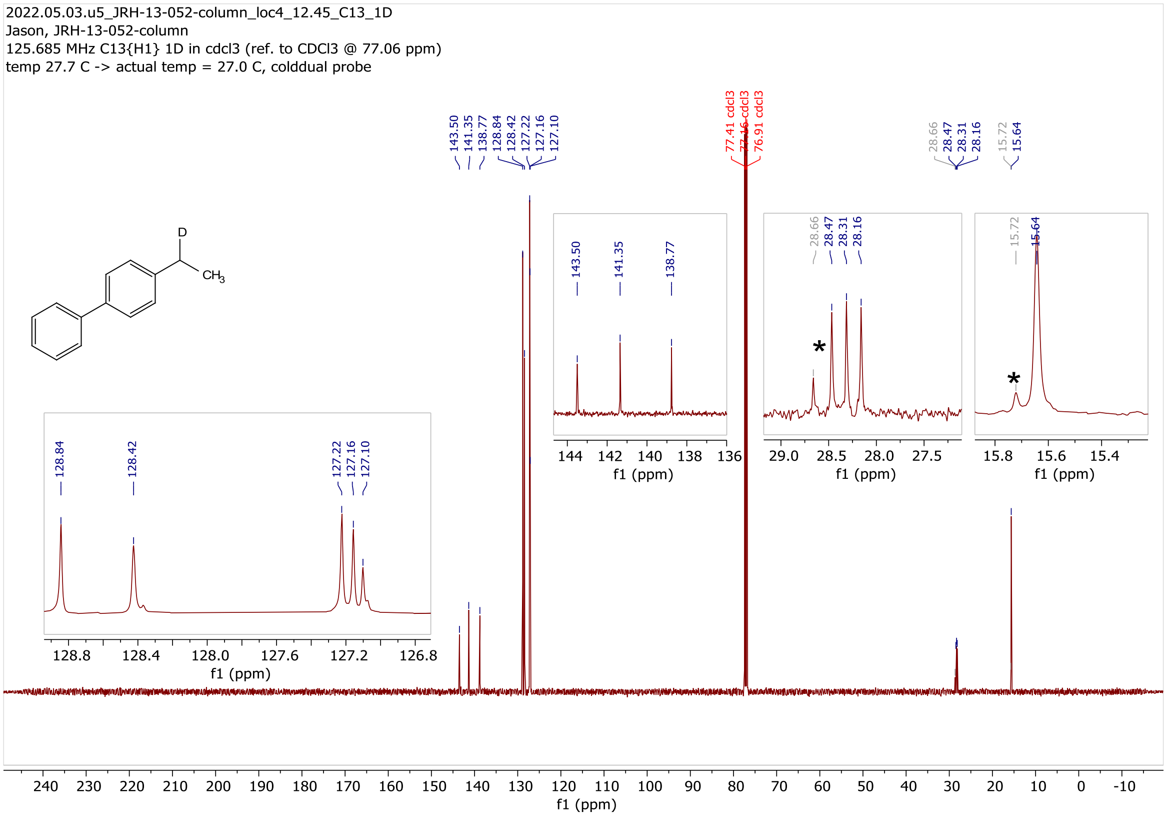

^{1}H (600 MHz) and ^{13}C (151 MHz) NMR of compound 7aa ($CDCl_3$)

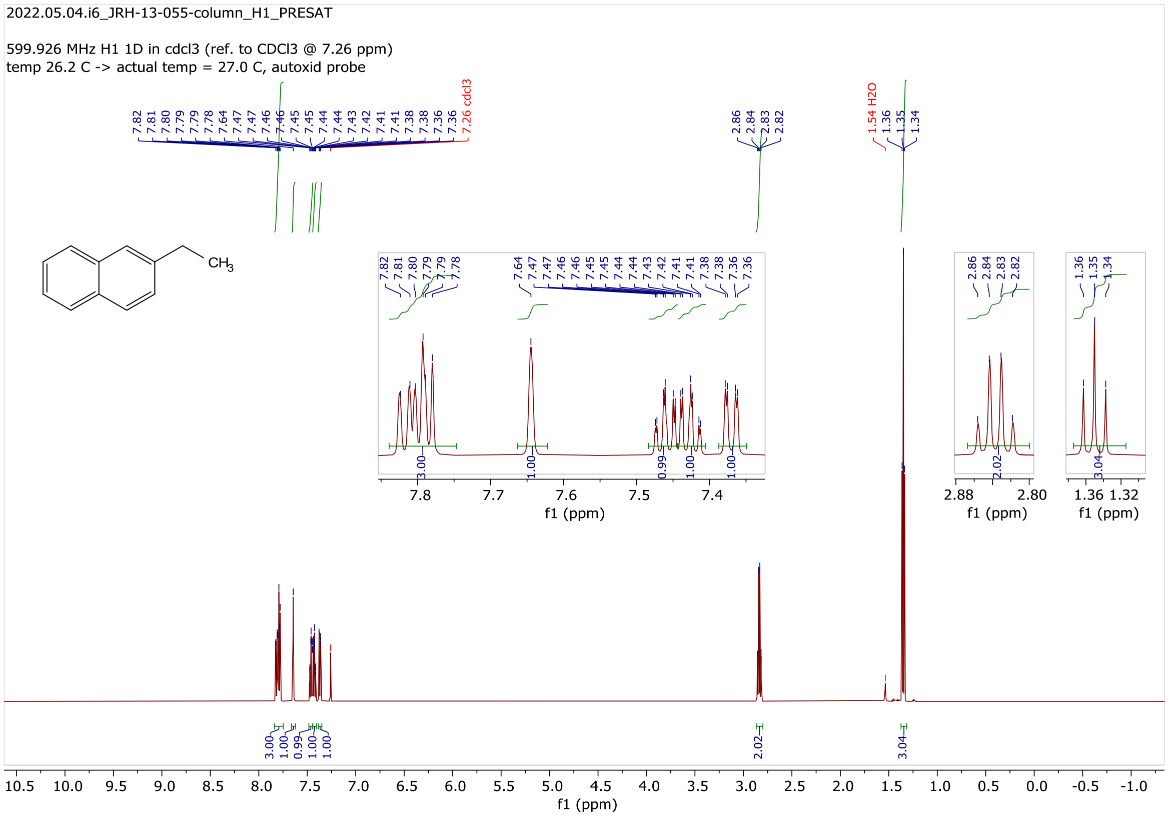

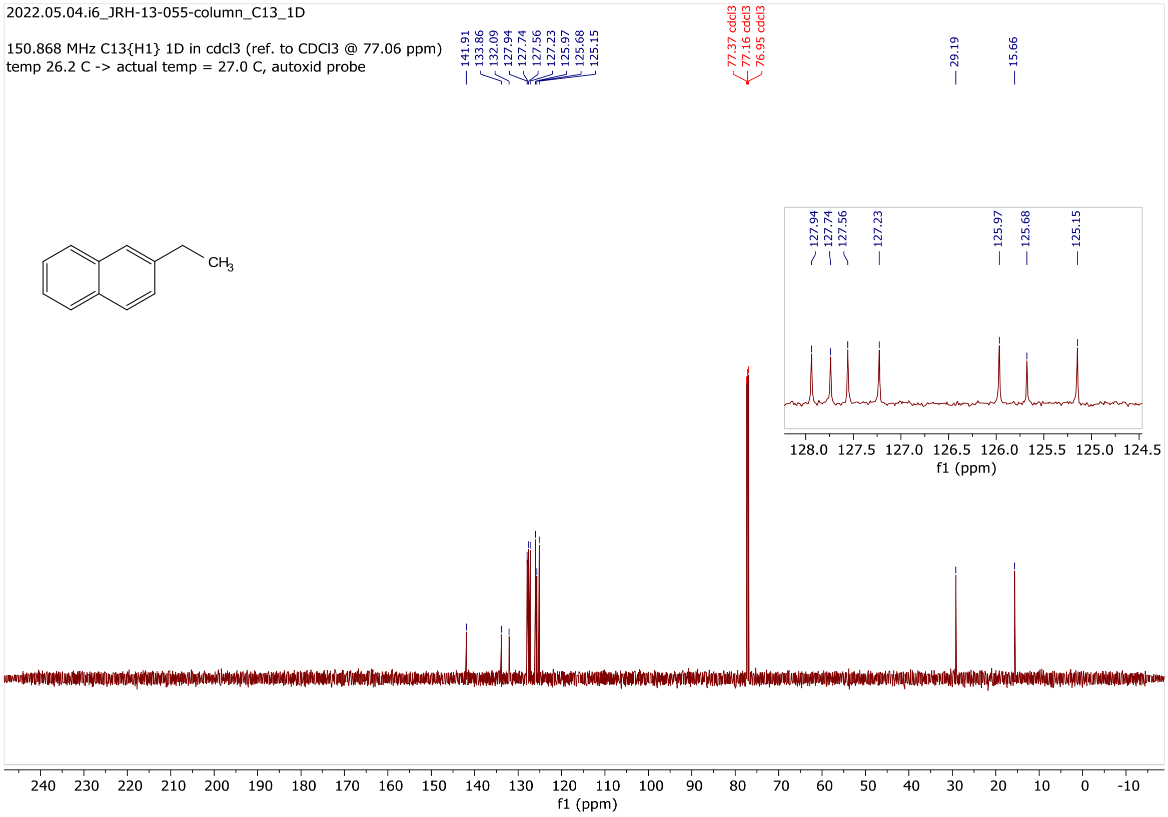

^{1}H (600 MHz) and ^{13}C (151 MHz) NMR of compound 7ab ($CDCl_3$)

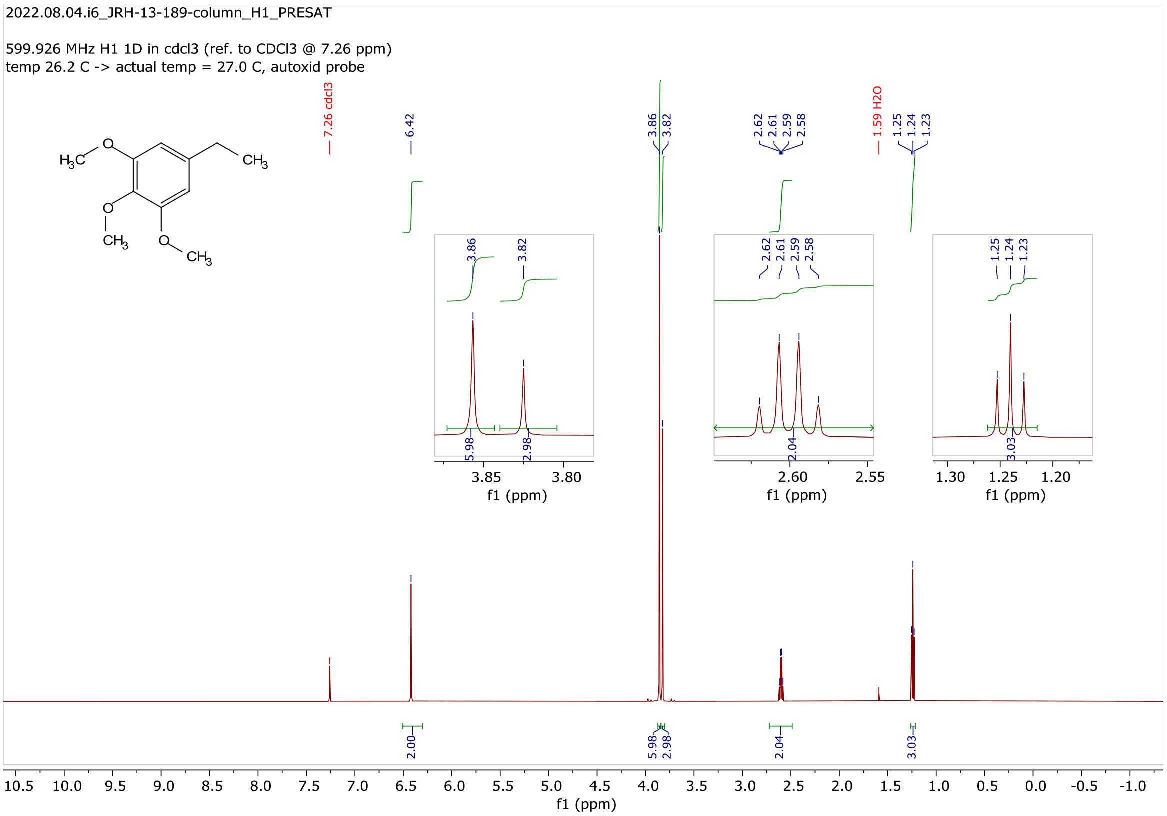

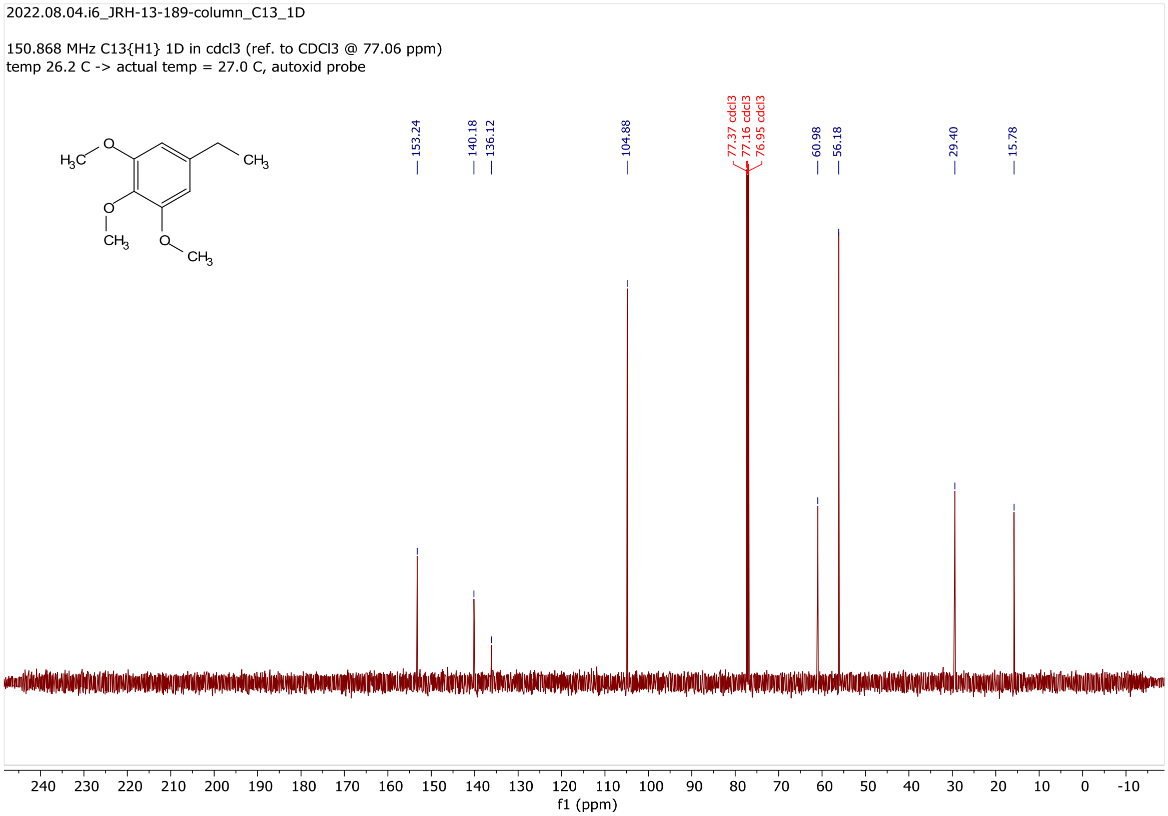

^{1}H (600 MHz) and ^{13}C (151 MHz) NMR of compound 7ac ($CDCl_3$)

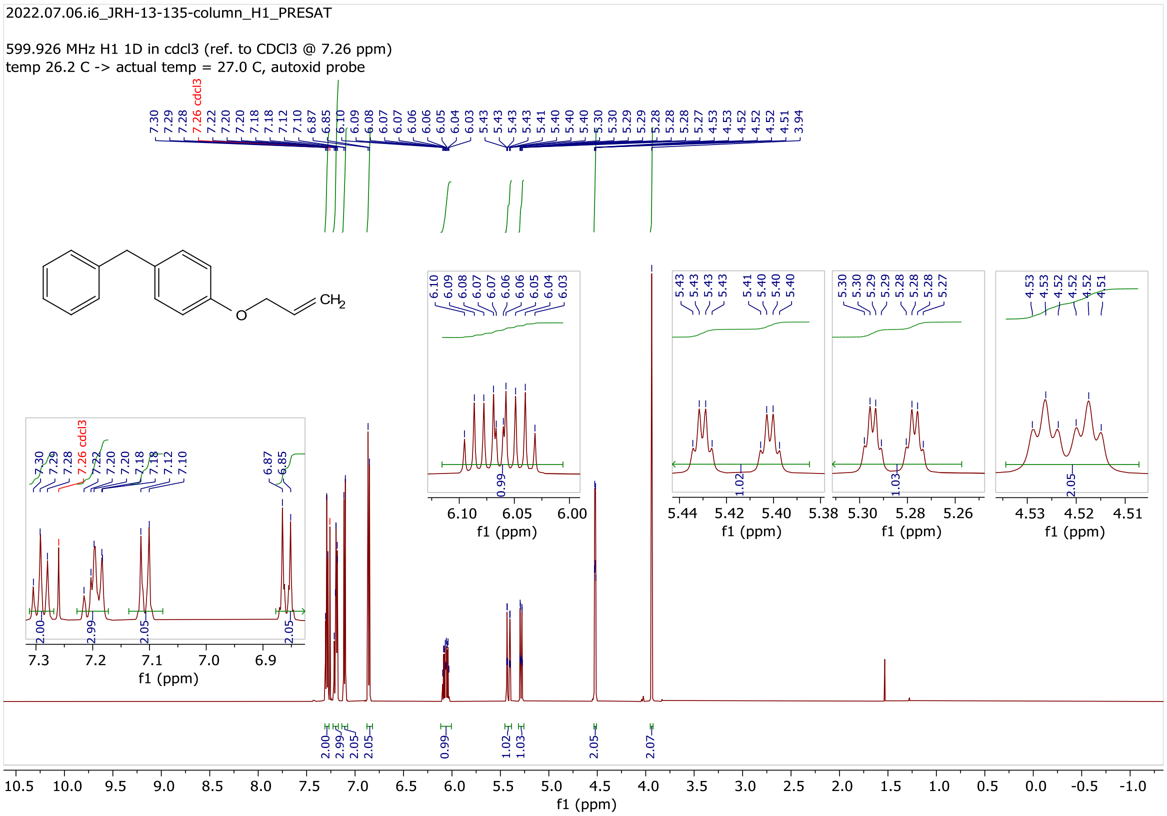

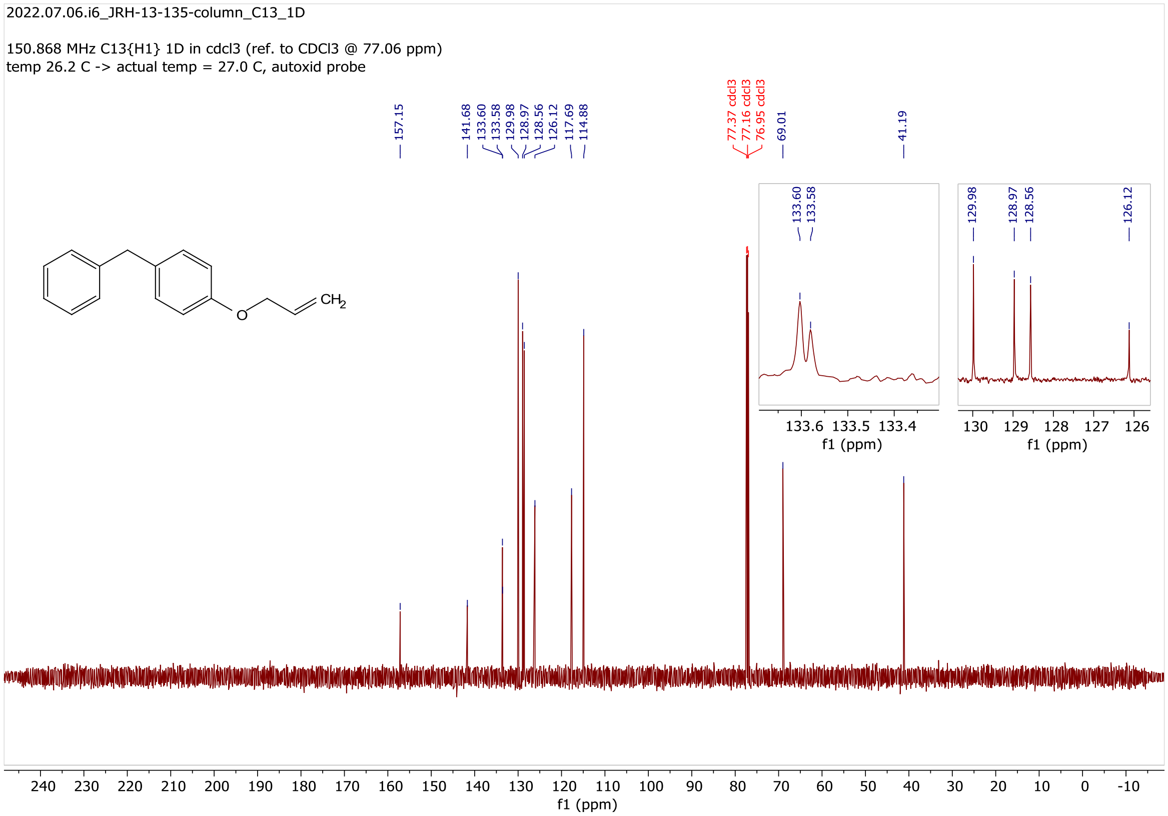

^{1}H (500 MHz) and ^{13}C (126 MHz) NMR of compound 7ad ($CDCl_3$)

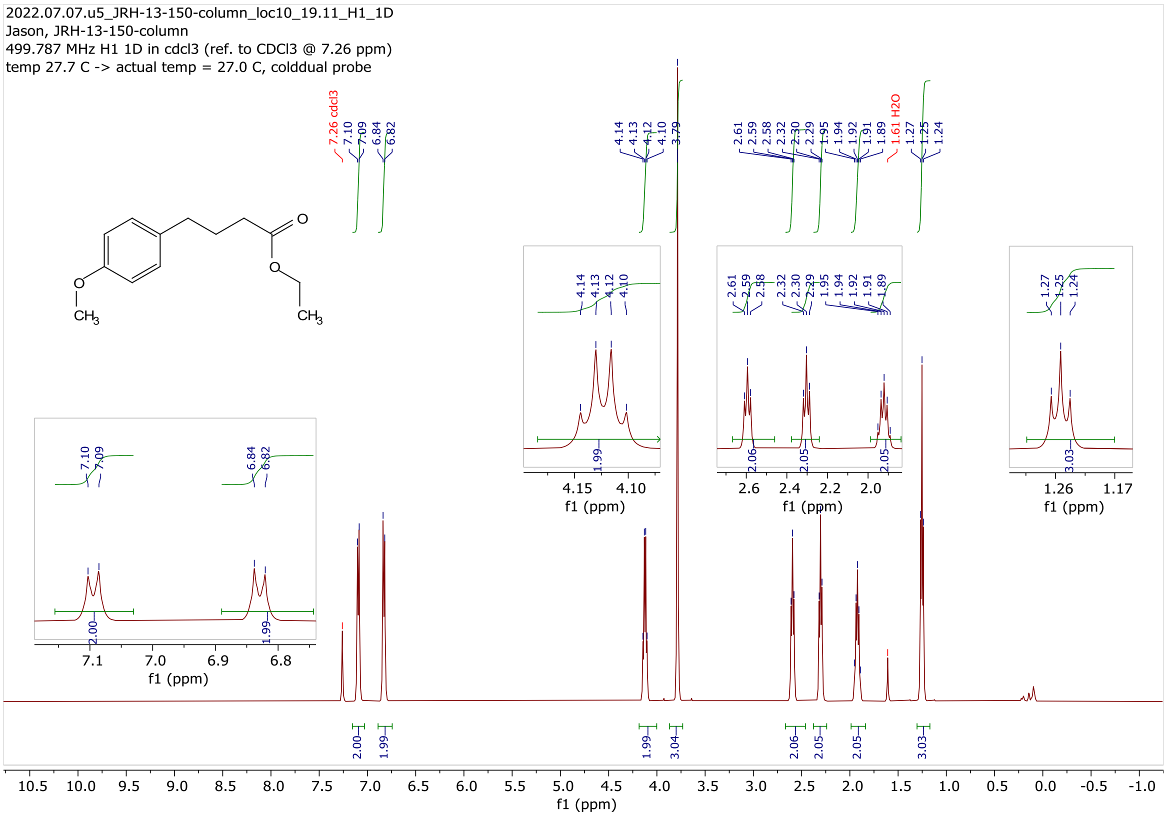

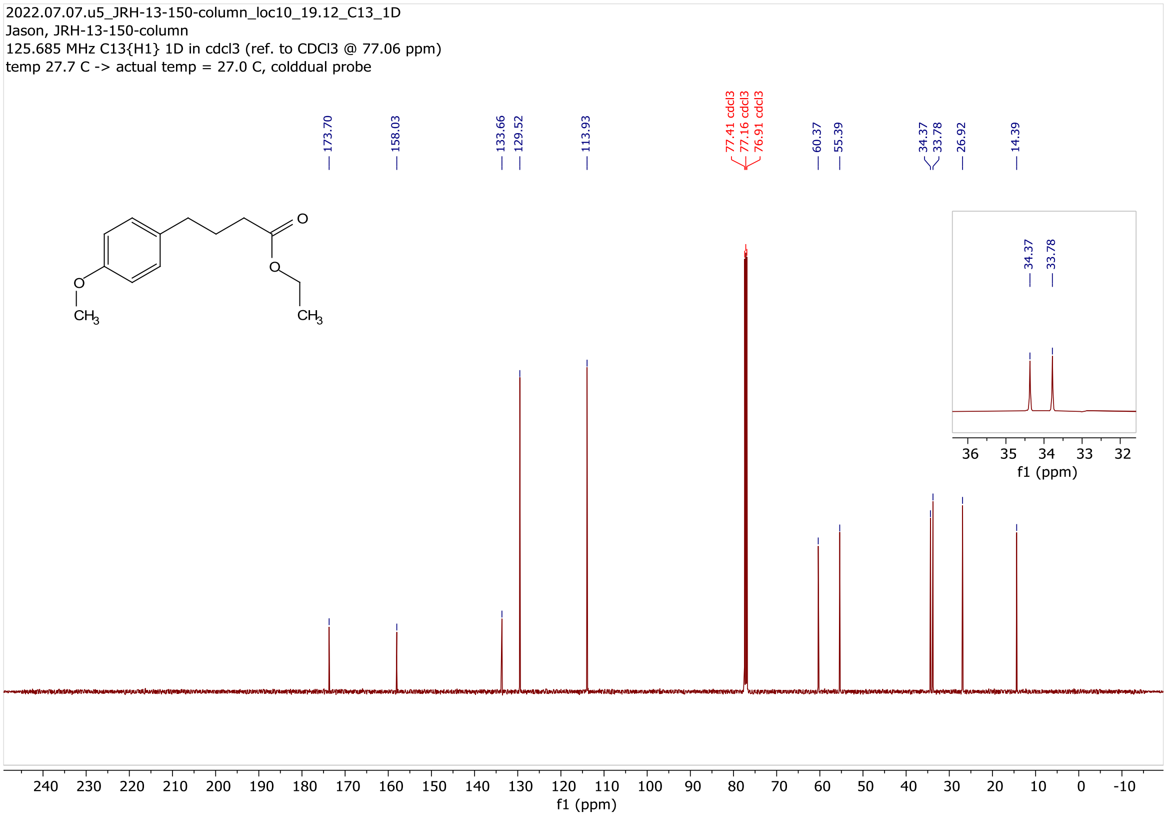

^{1}H (500 MHz) and ^{13}C (126 MHz) NMR of compound 7ae ($CDCl_3$)

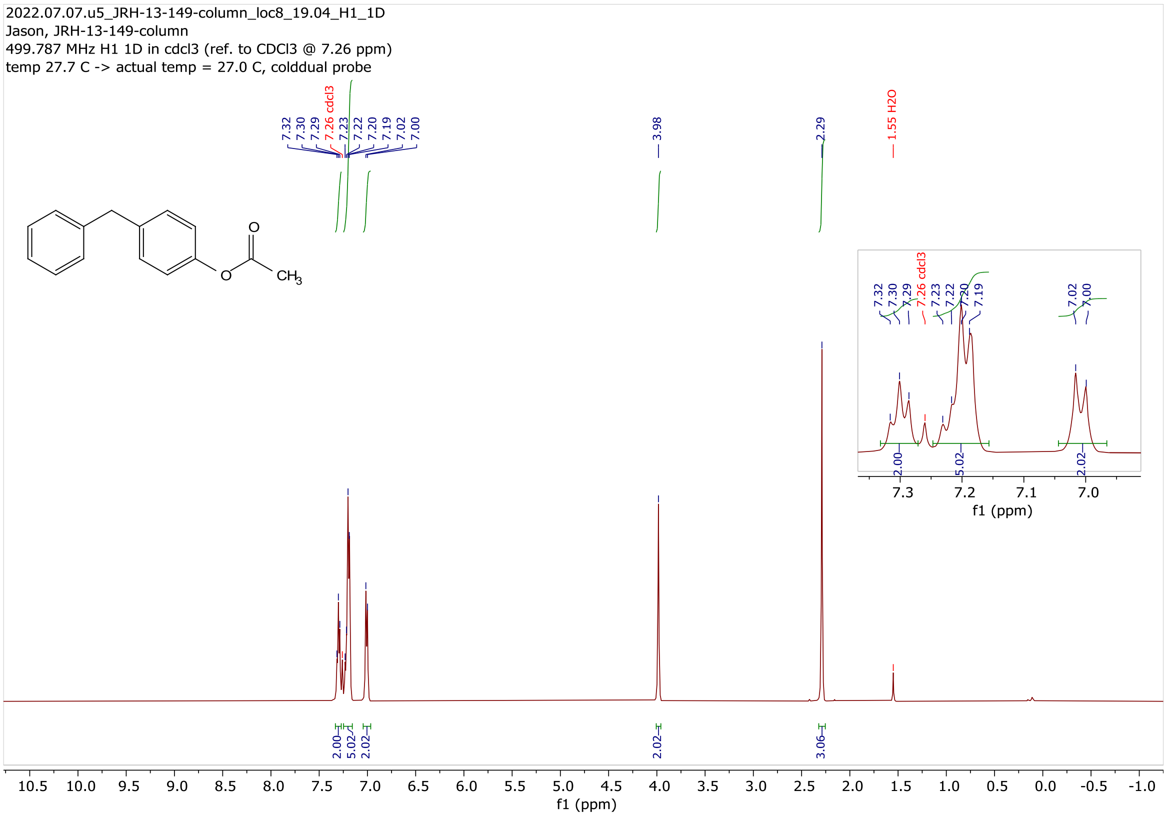

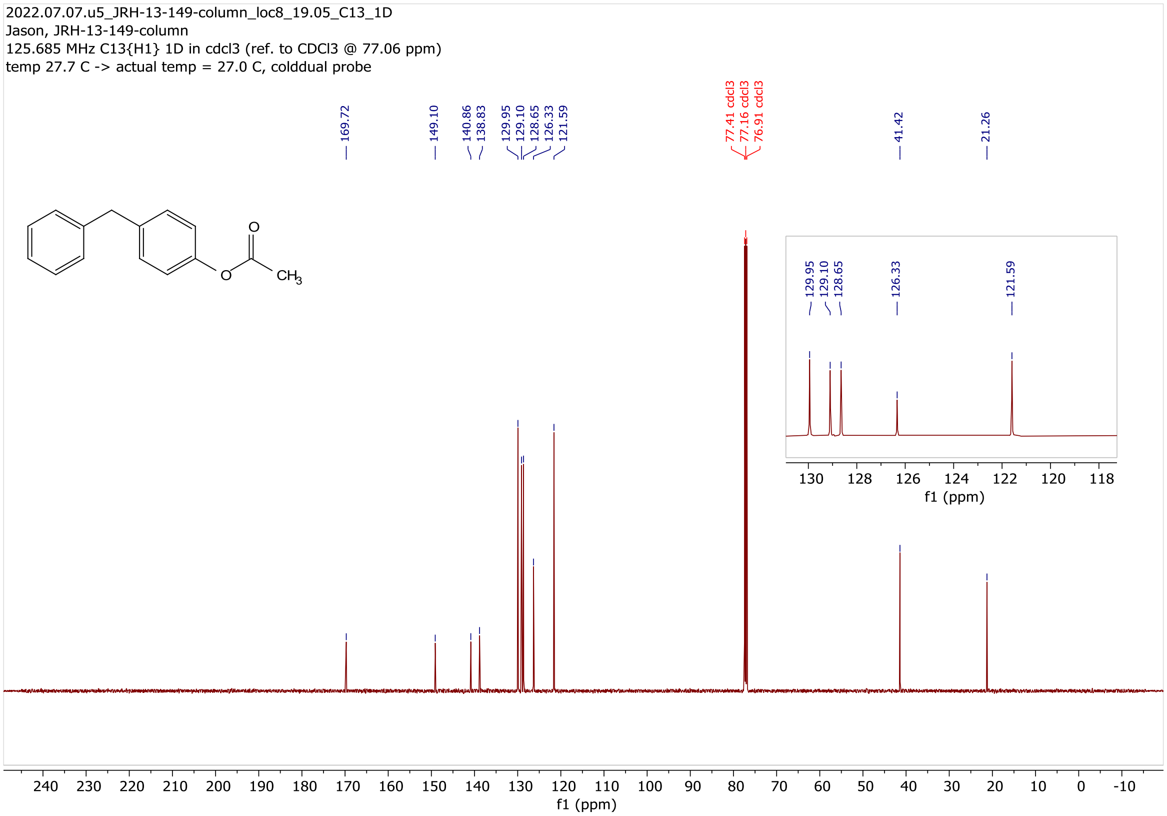

^{1}H (600 MHz) and ^{13}C (151 MHz) NMR of compound 7af ($CDCl_3$)

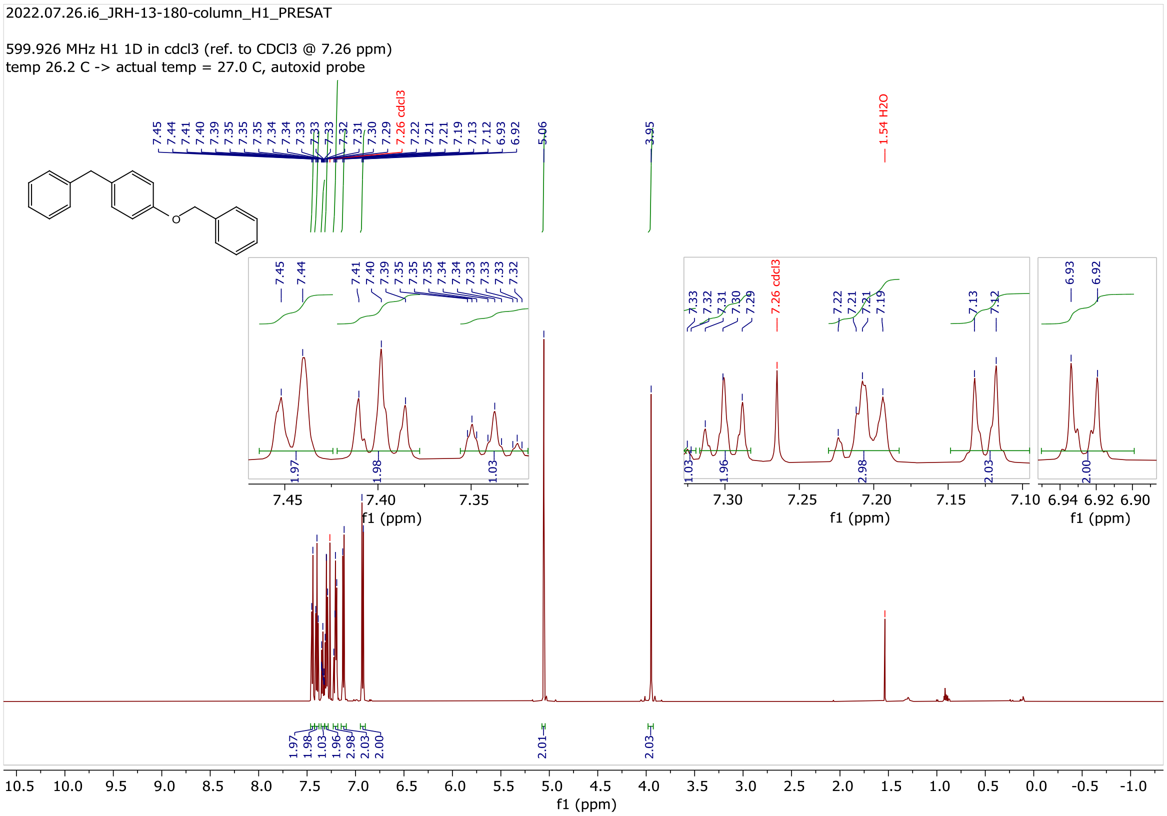

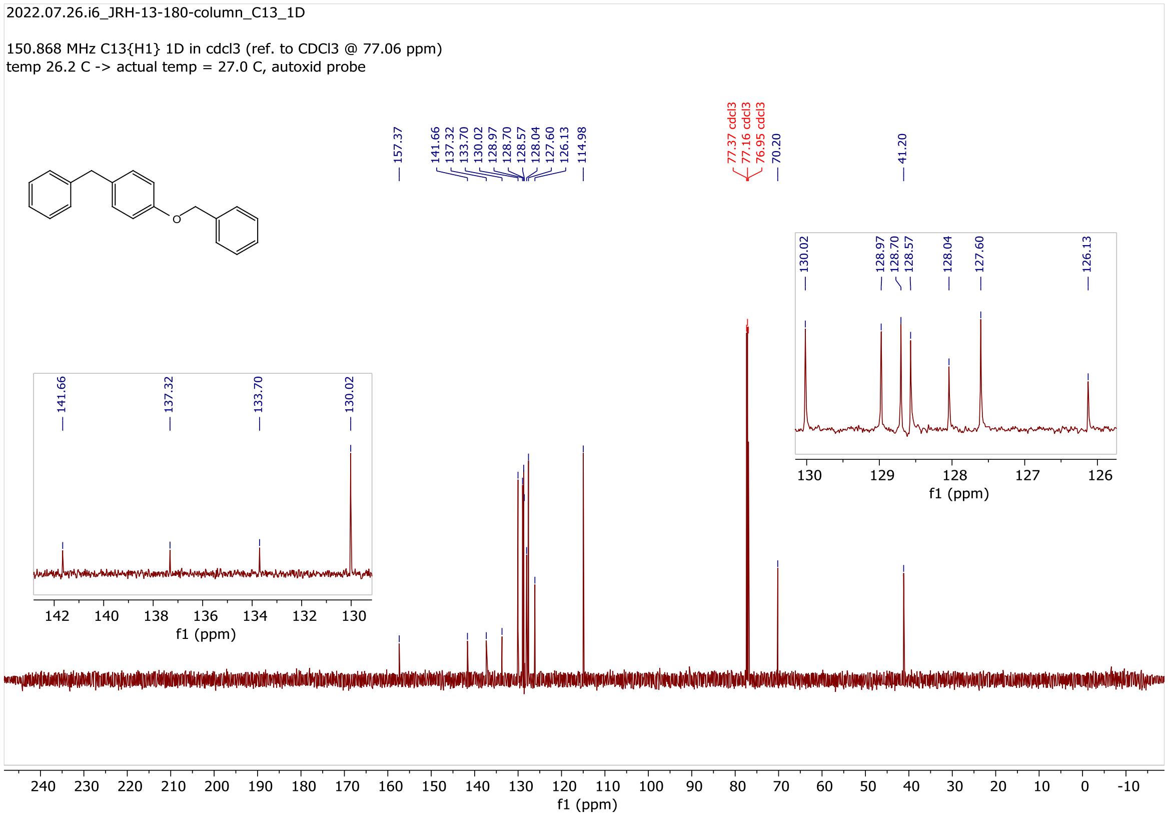

^{1}H (500 MHz) and ^{13}C (126 MHz) NMR of compound 7ag ($CDCl_3$)

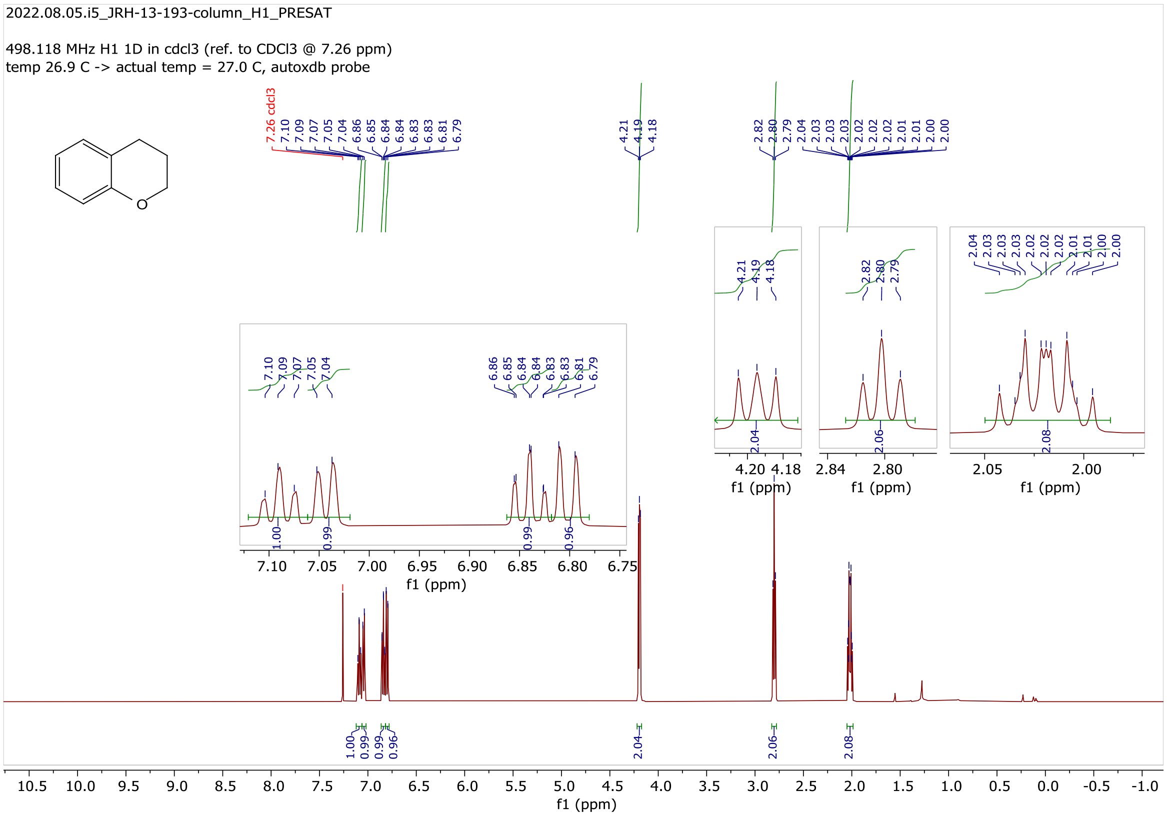

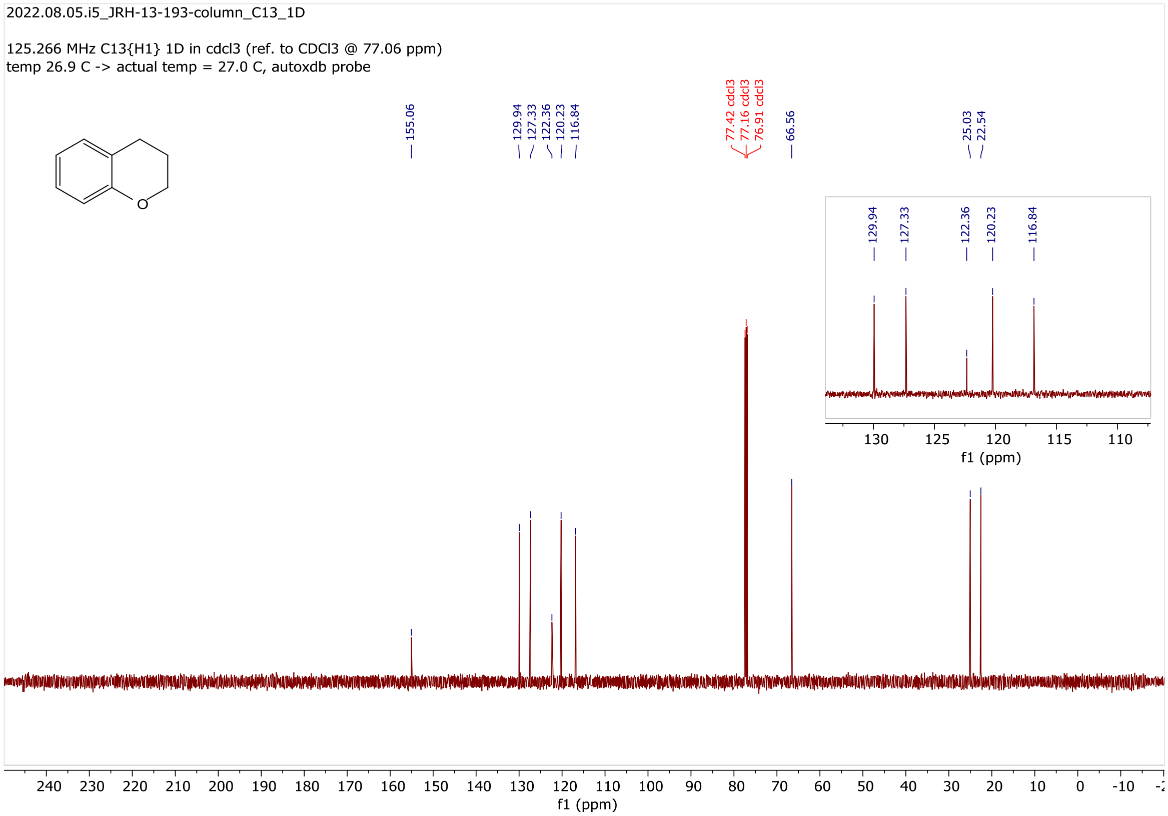

^{1}H (600 MHz) and ^{13}C (151 MHz) NMR of compound 7ah ($CDCl_3$)

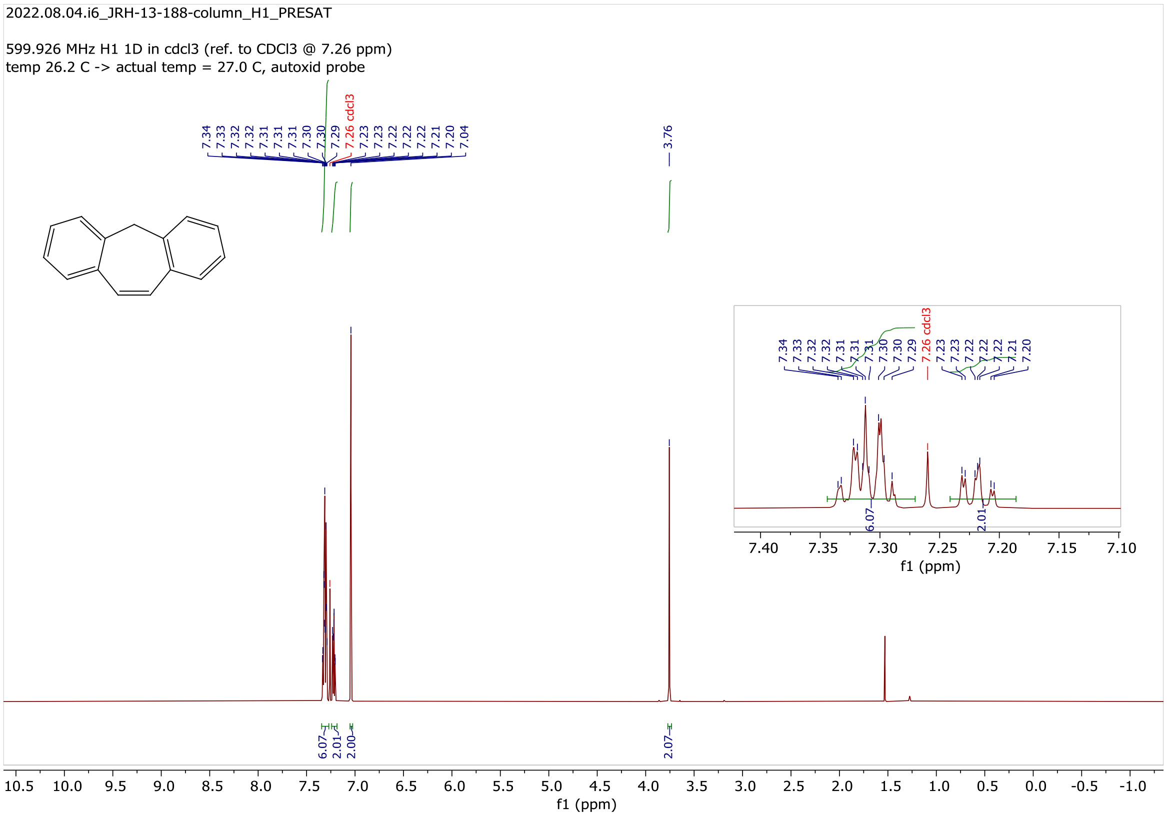

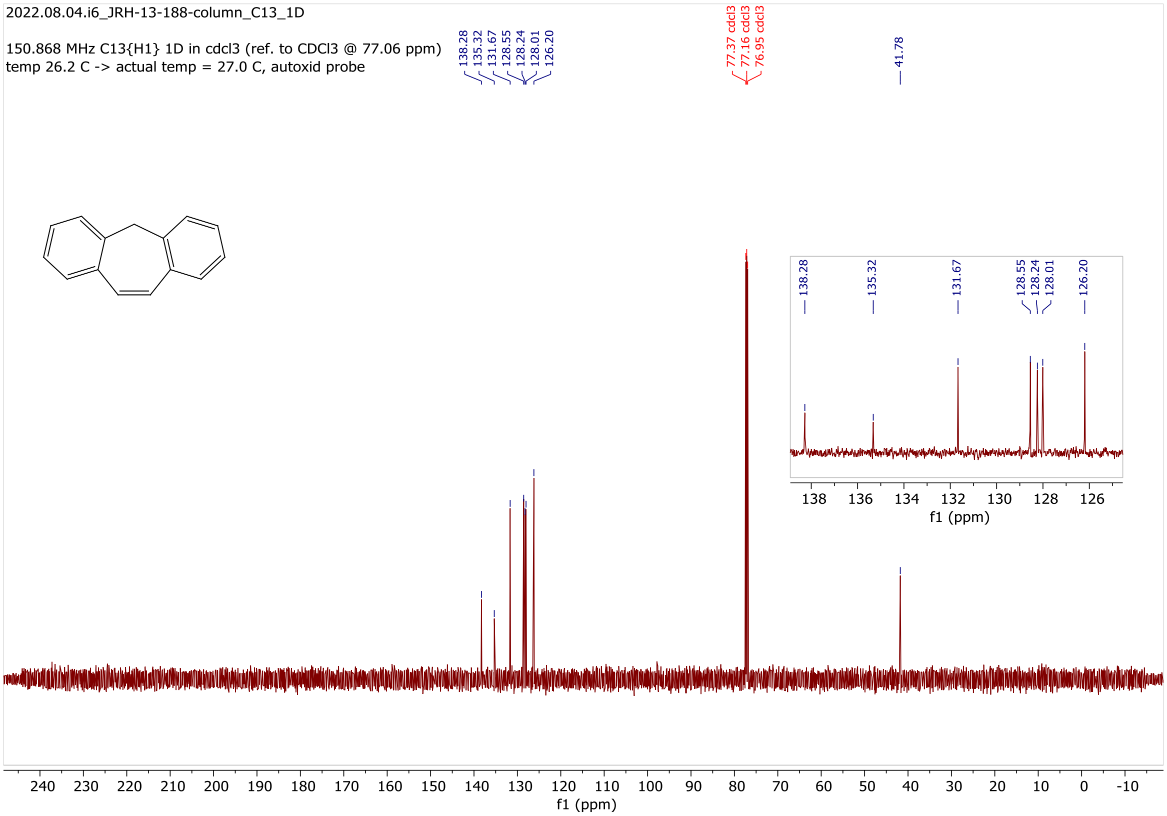

^1H (500 MHz) and ^{13}C (126 MHz) NMR of compound 7ai ($CDCl_3$)

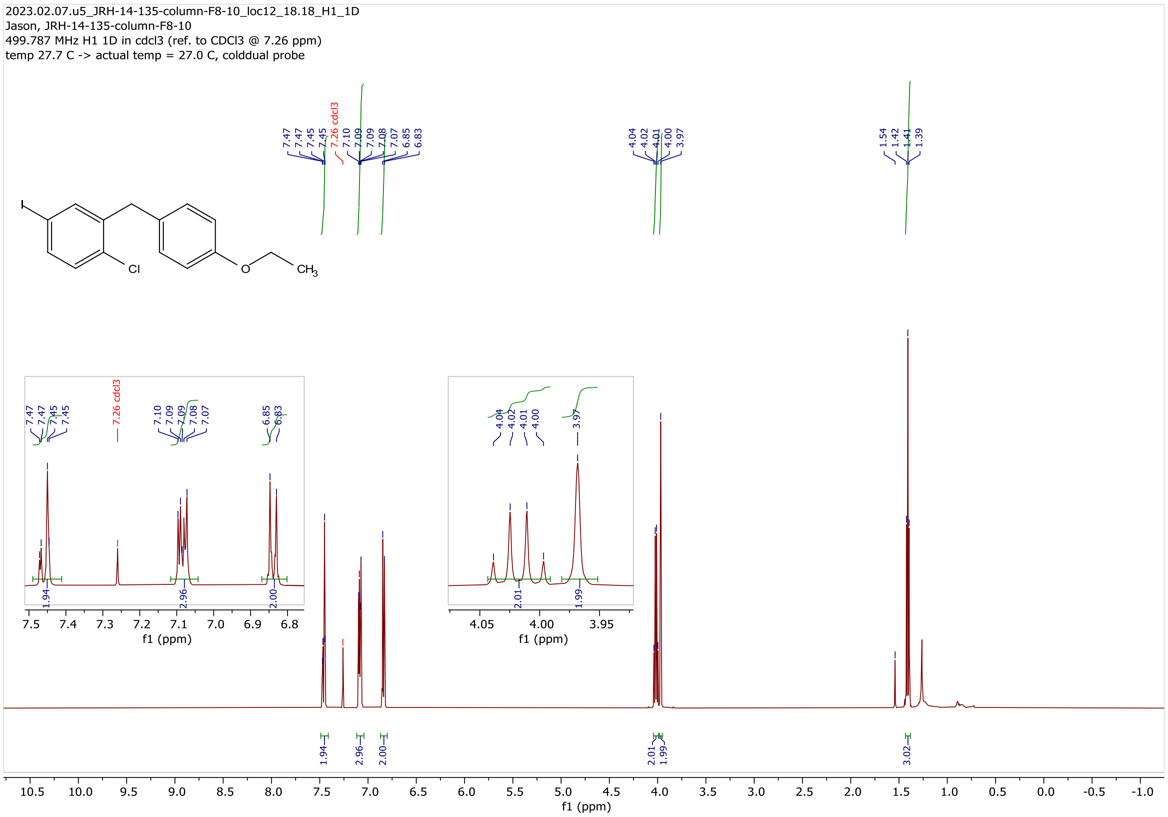

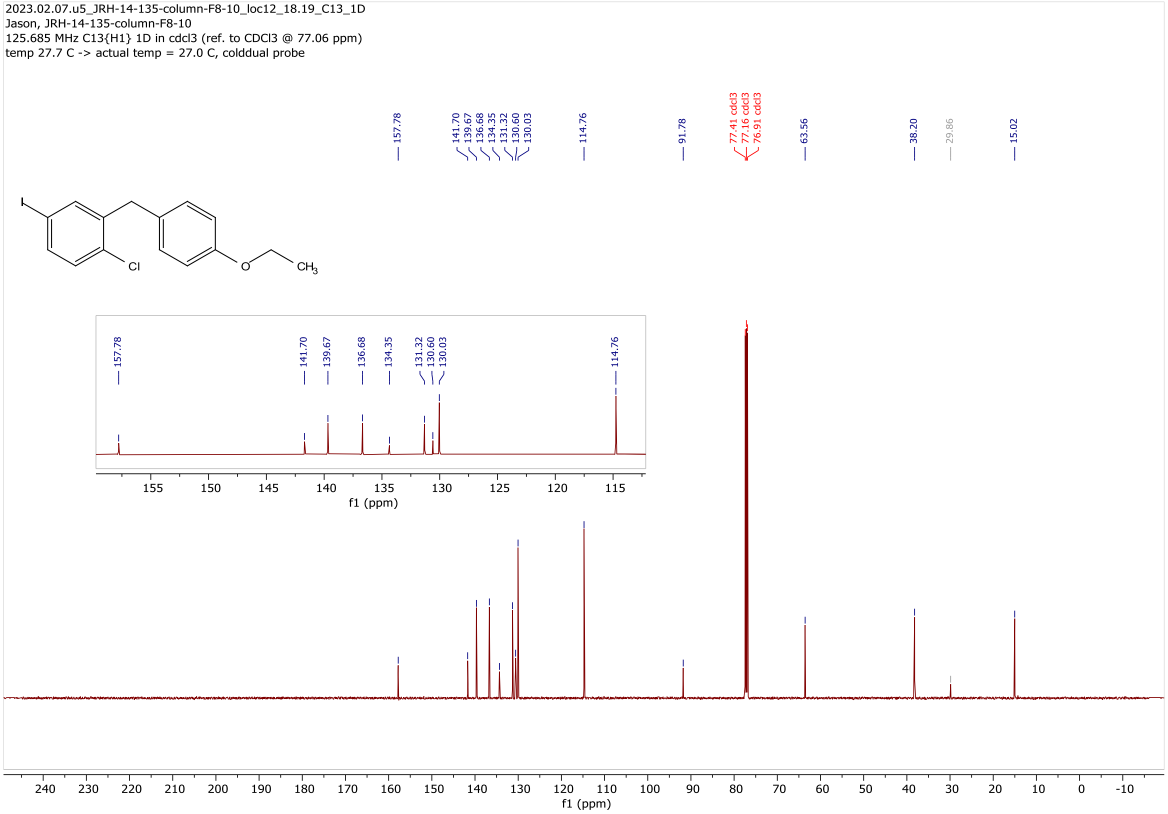

^{1}H (600 MHz) and ^{13}C (151 MHz) NMR of compound 7aj ($CDCl_3$)

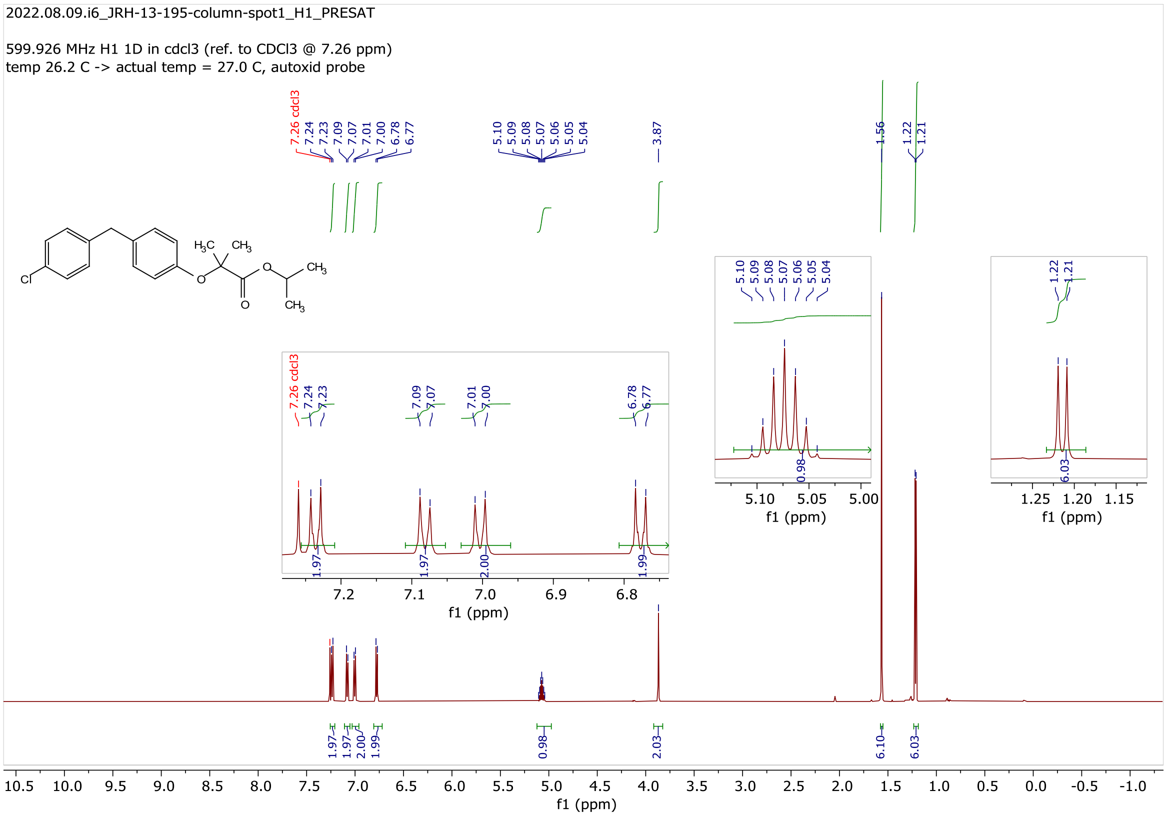

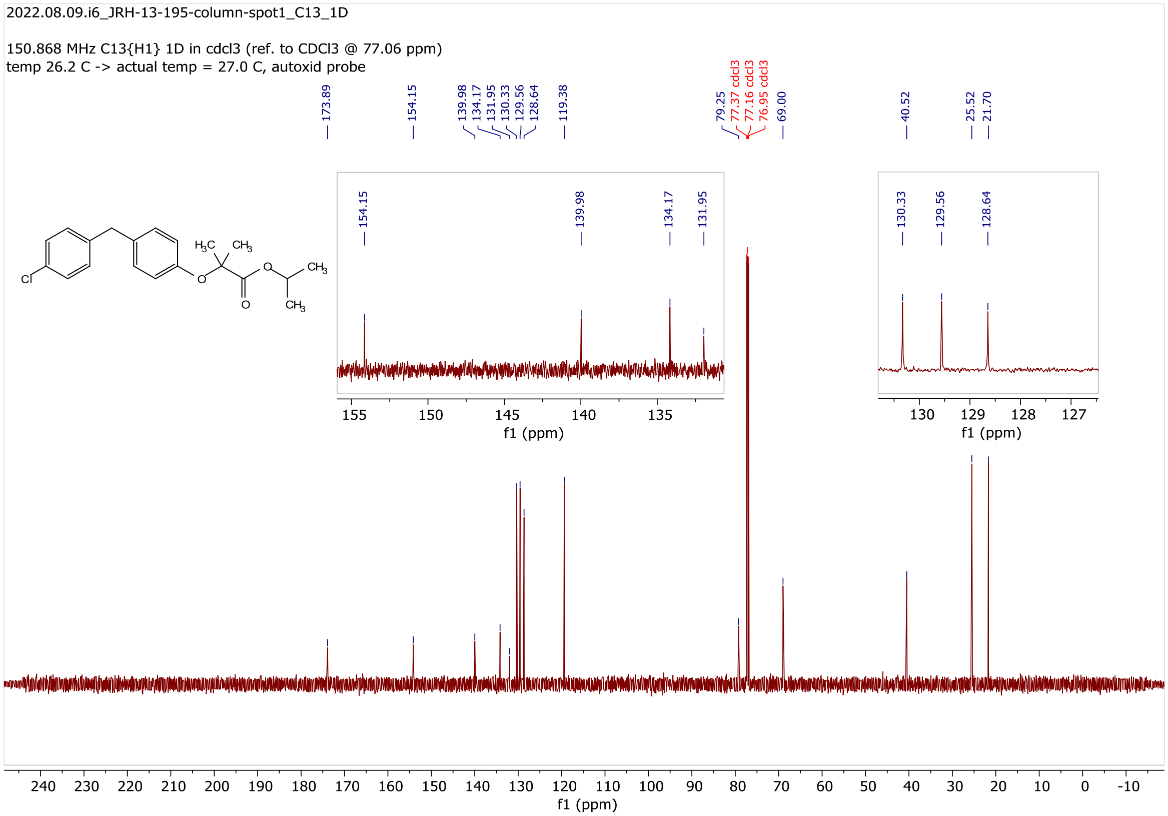

^{1}H (500 MHz) and ^{13}C (126 MHz) NMR of compound 7ak ($CDCl_3$)

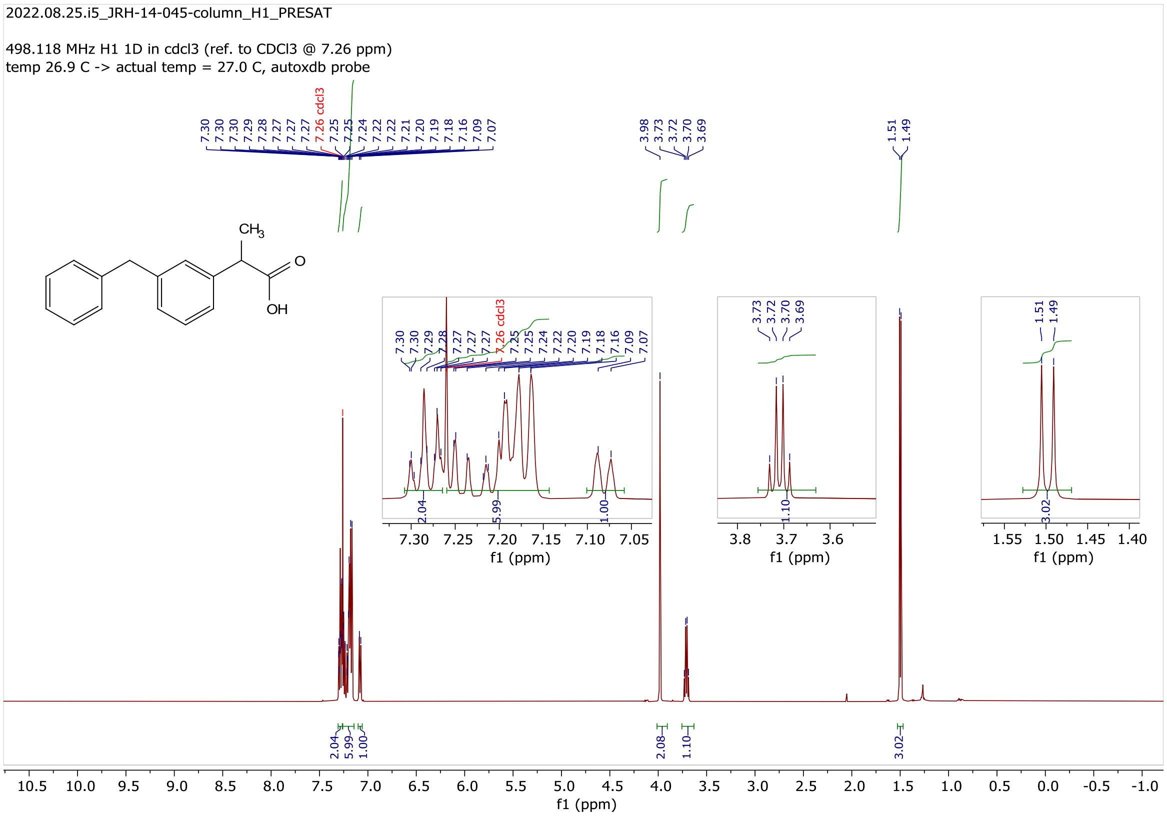

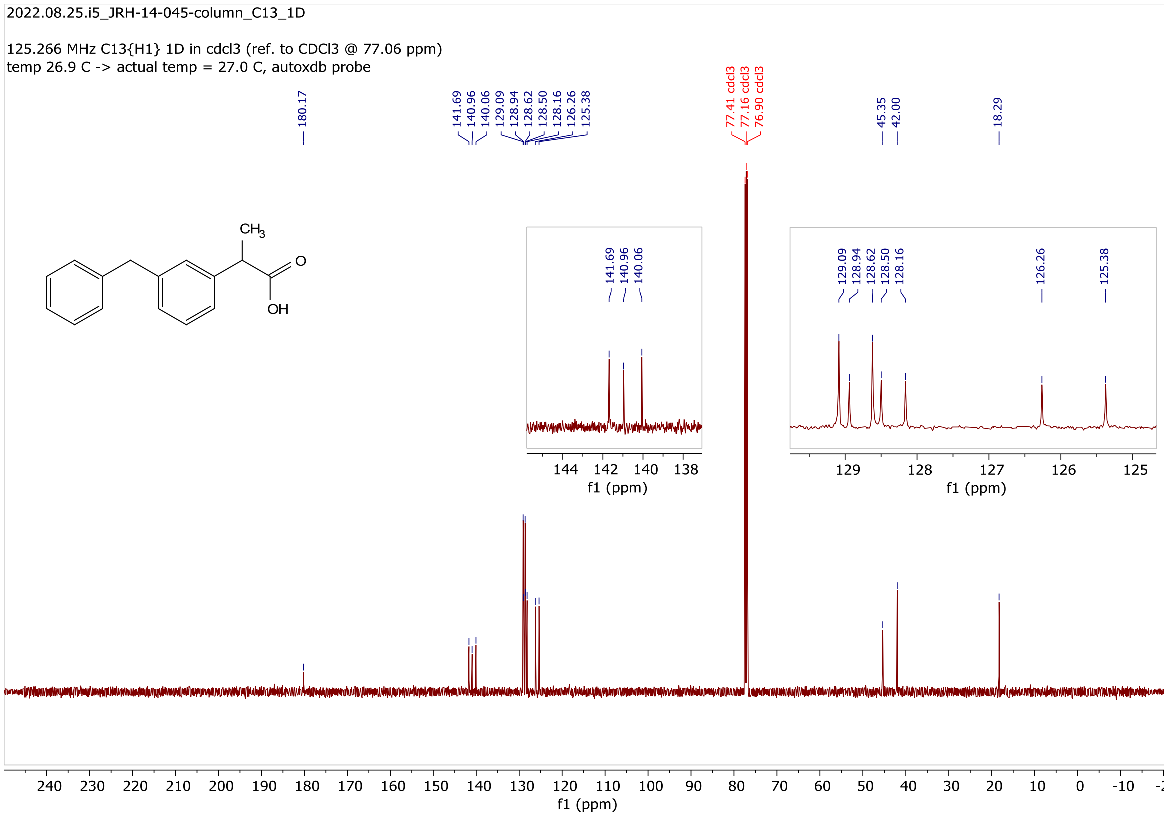

^{1}H (500 MHz) and ^{13}C (126 MHz) NMR of compound 10 ($CDCl_3$)

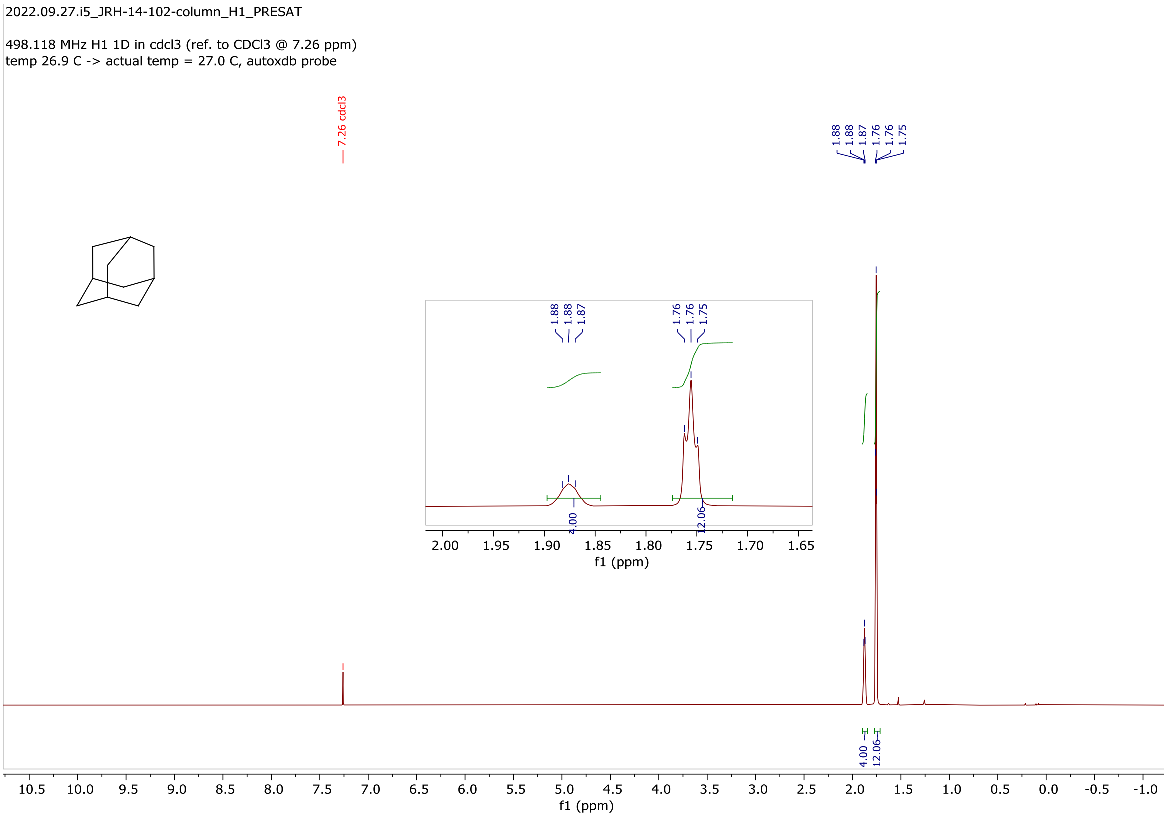

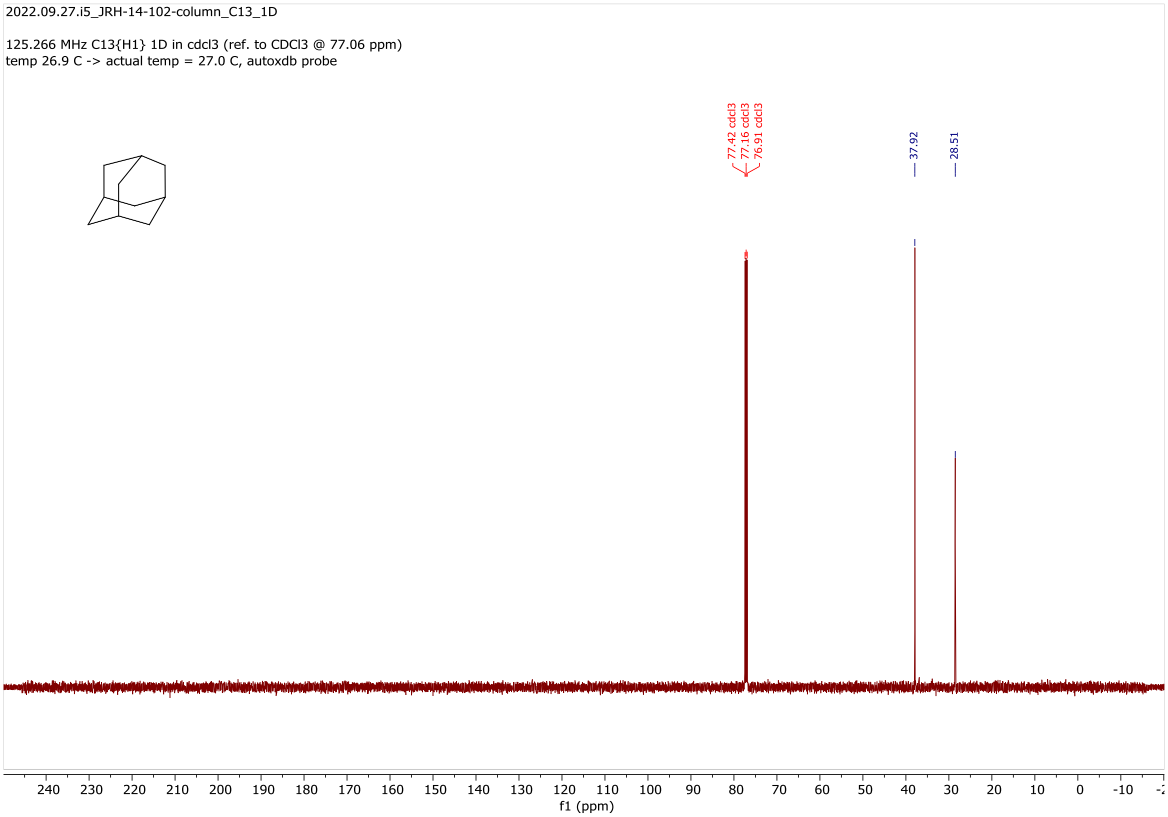

^{1}H (500 MHz) and ^{13}C (126 MHz) NMR of compound 12 ($CDCl_3$)

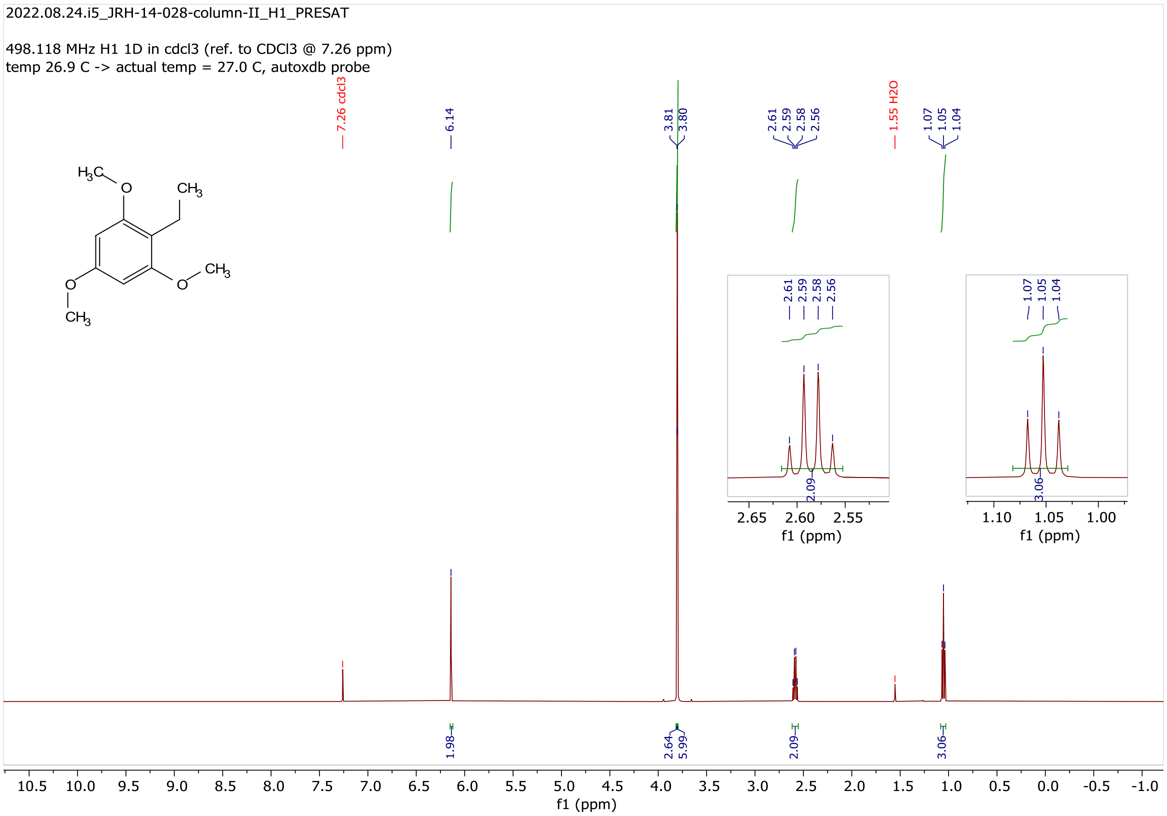

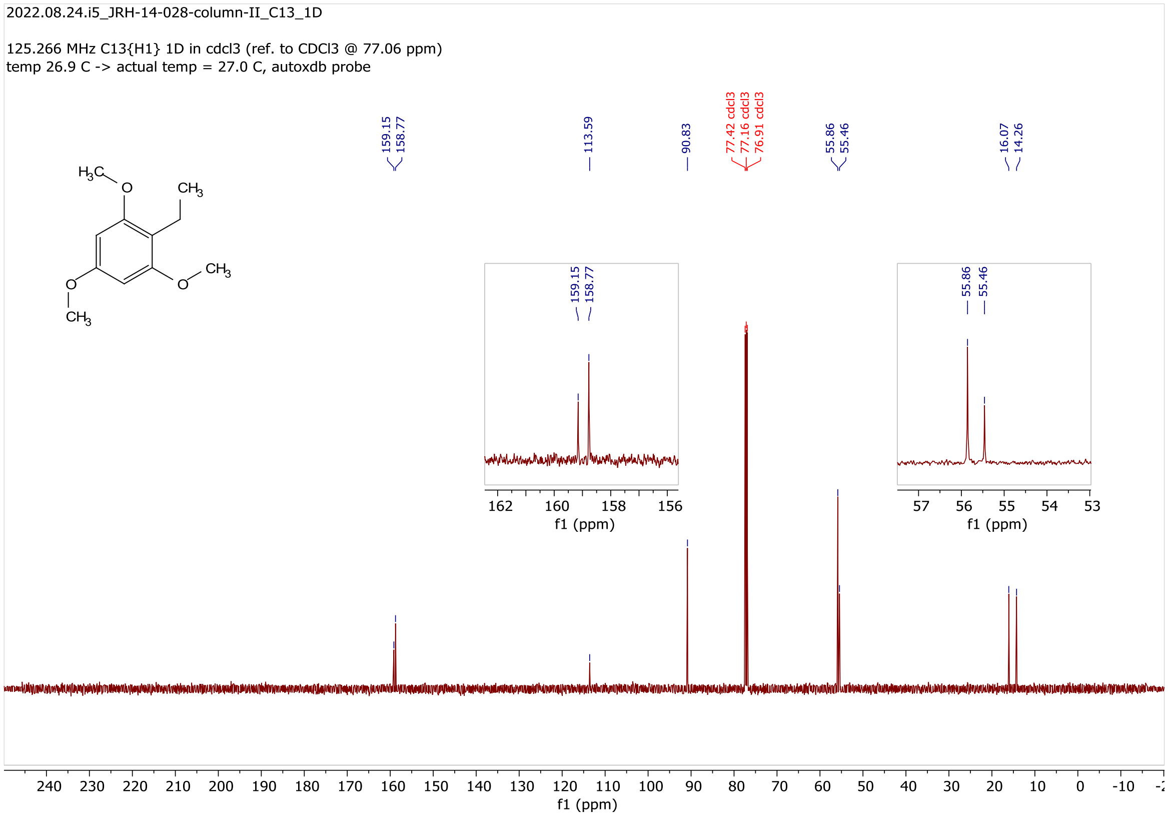

^{1}H (600 MHz) and ^{13}C (151 MHz) NMR of compound 14a ($CDCl_3$)

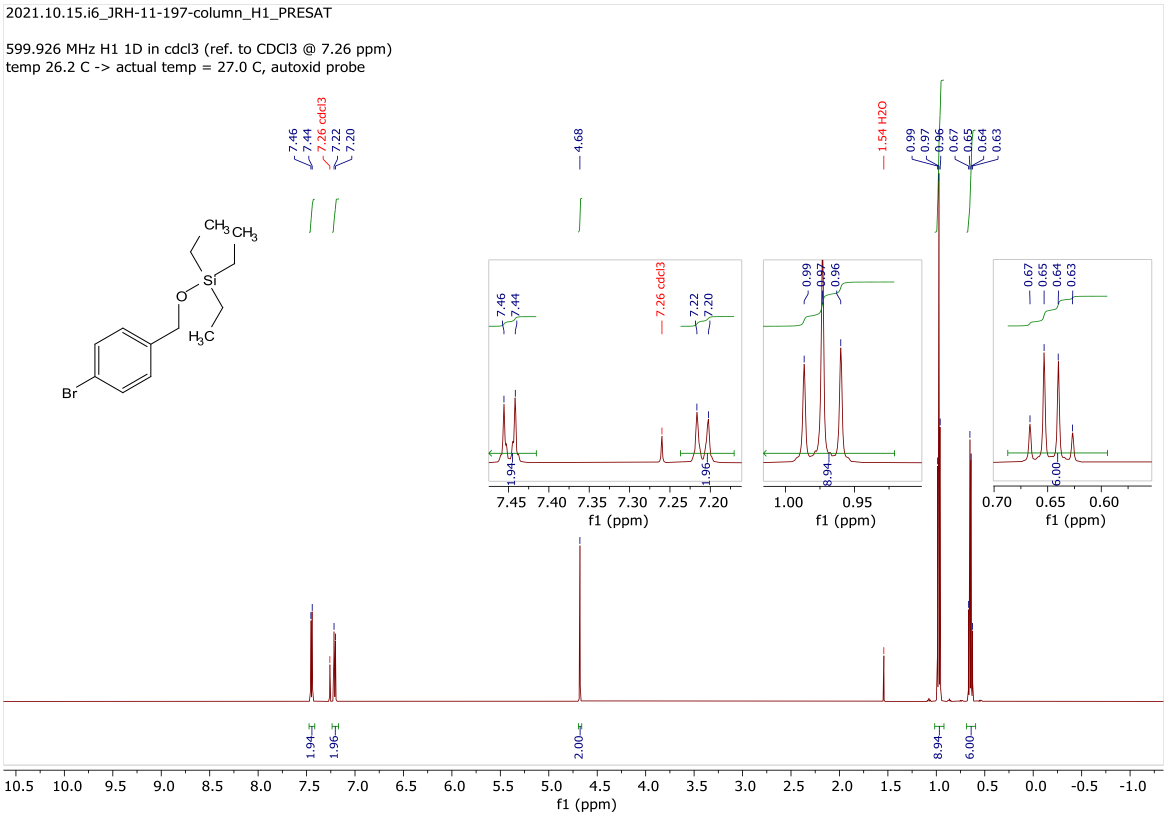

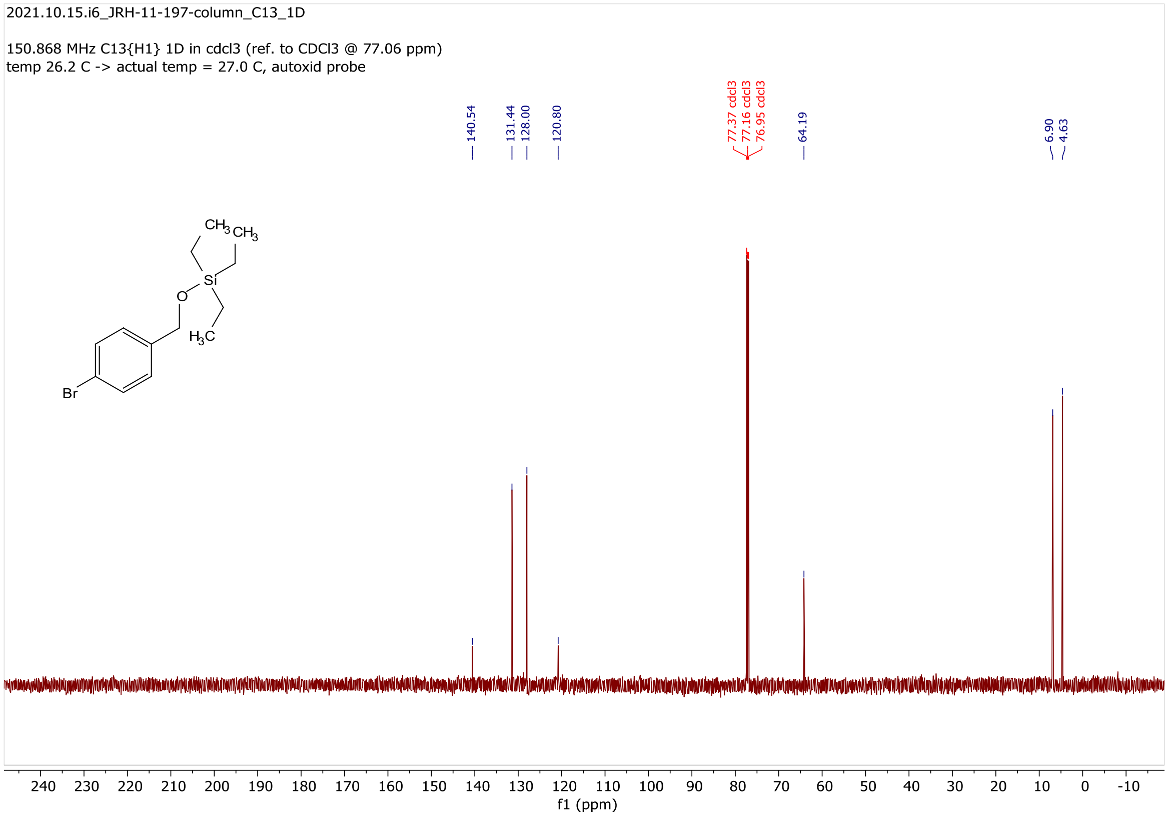

^{1}H (500 MHz) and ^{13}C (126 MHz) NMR of compound 15 ($CDCl_3$)

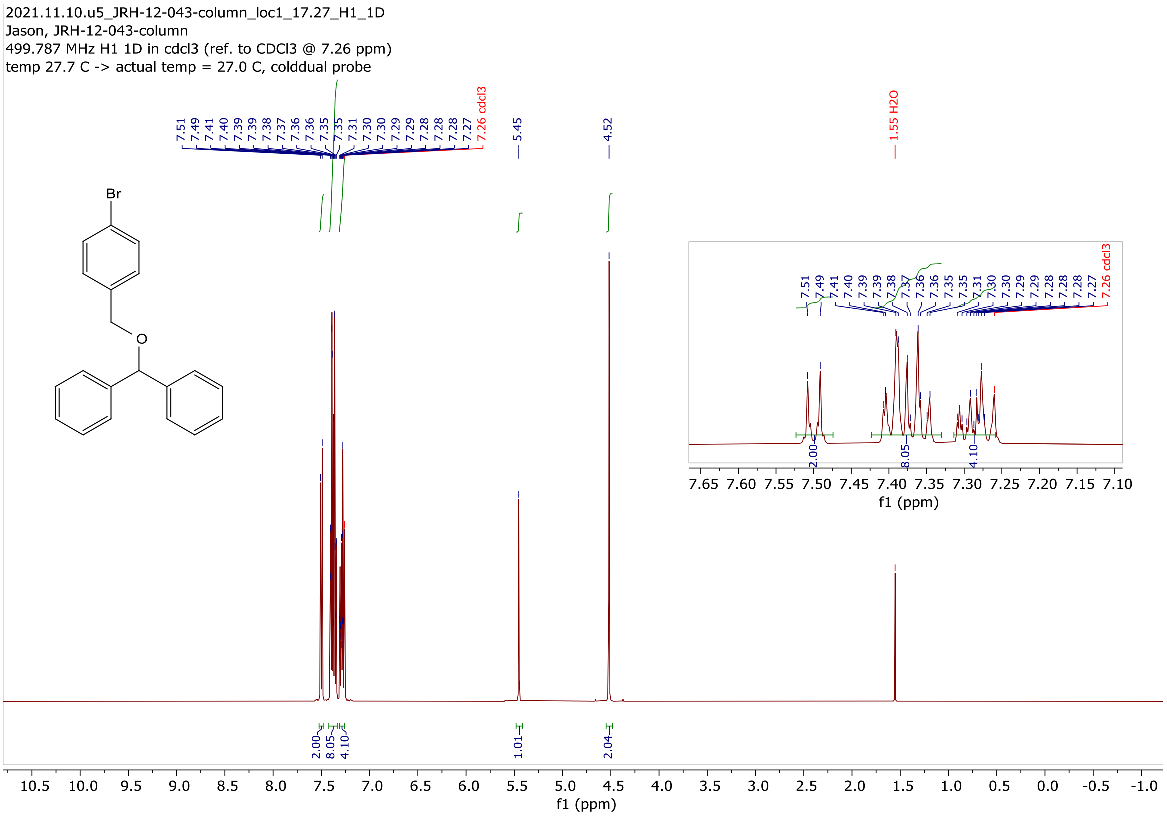

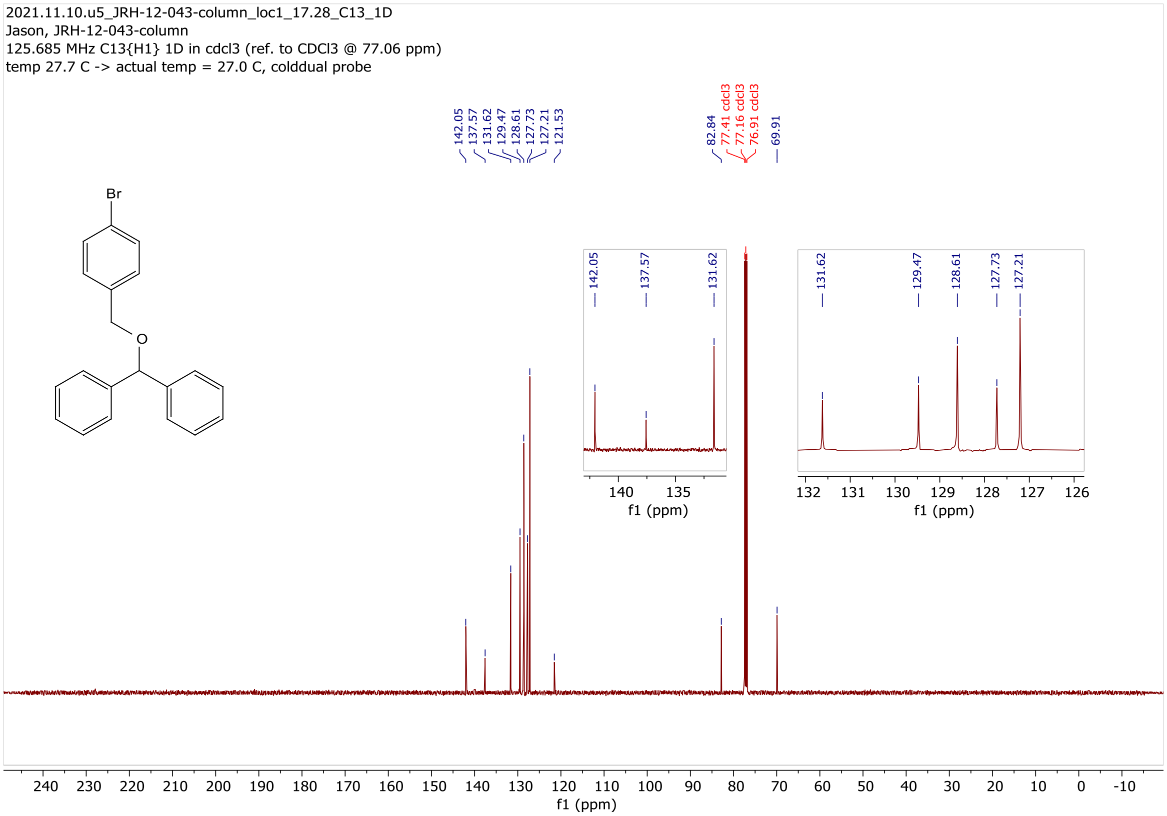

^{1}H (500 MHz), ^{13}C (126 MHz), ^{11}B (128 MHz) and ^{19}F (376 MHz) NMR of compound 3-II (CD_3CN)

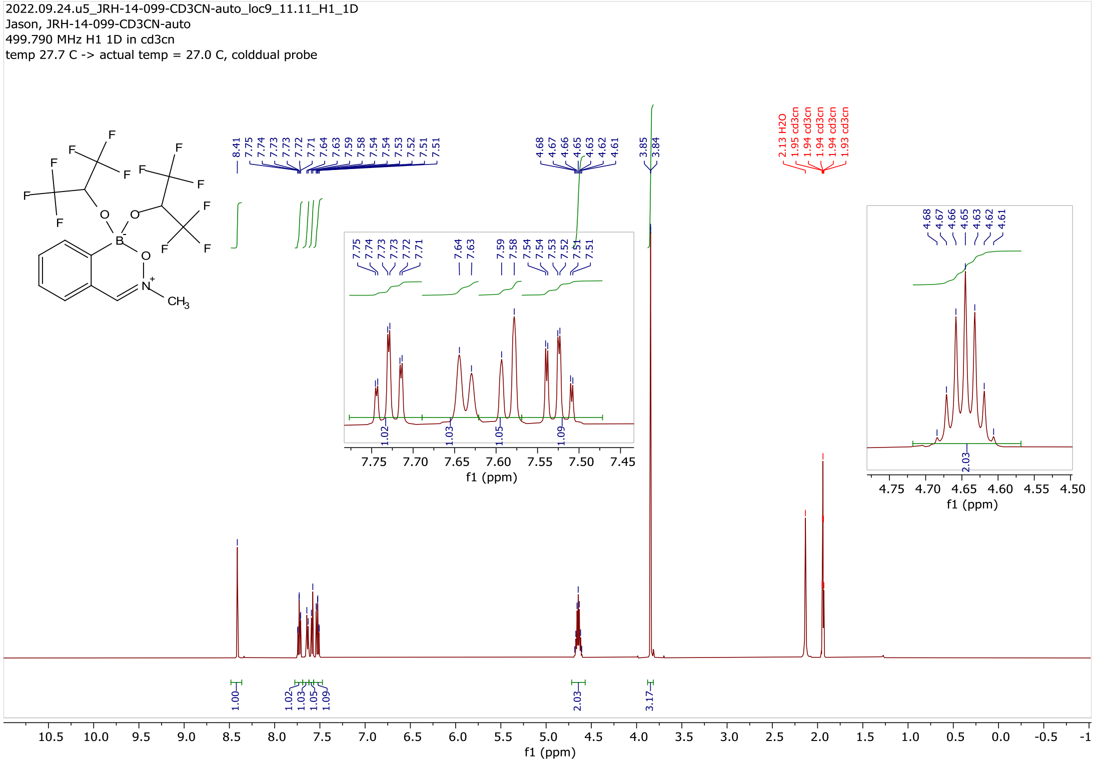

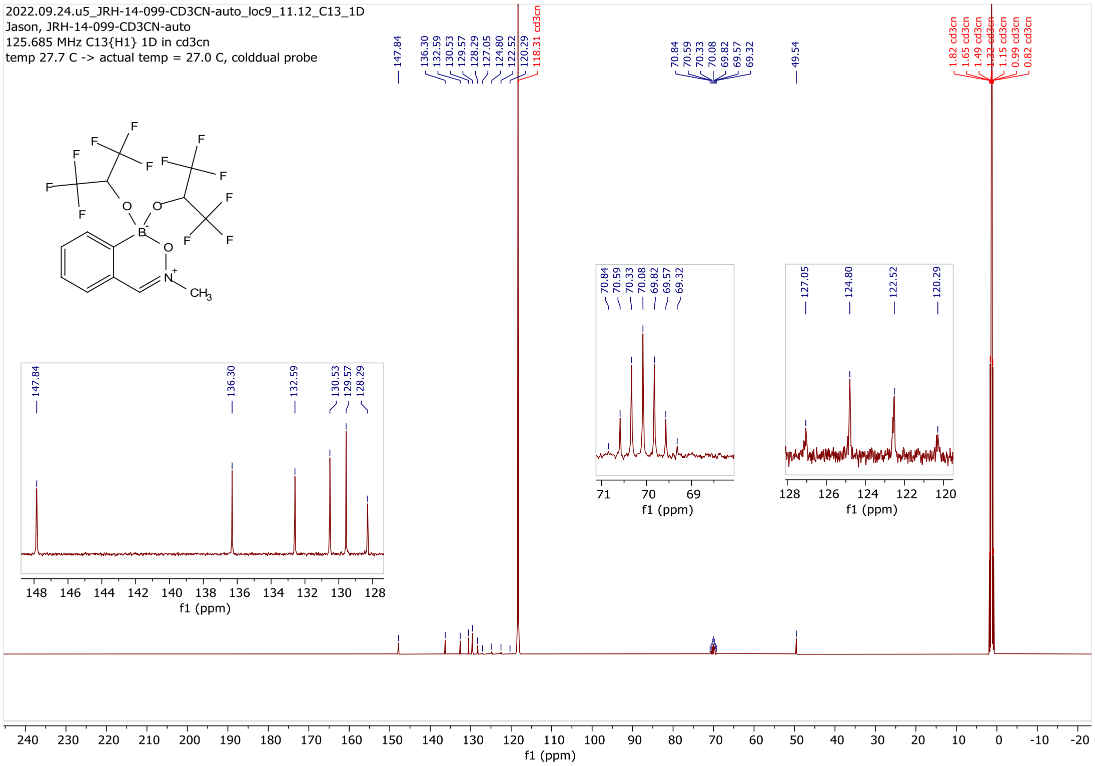

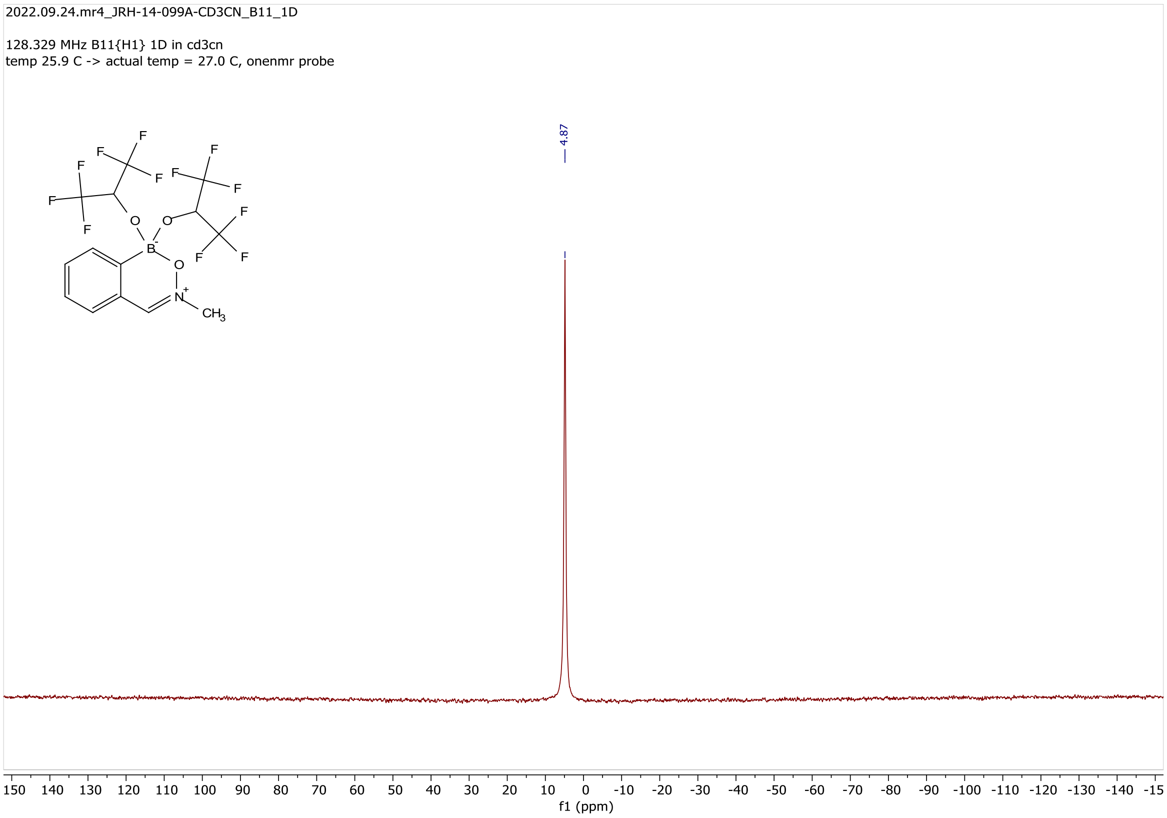
2022.09.24.mr4_JRH-14-099A-CD3CN_B11_1D
128.329 MHz B11{H1} 1D in cd3cn
temp 25.9 C -> actual temp = 27.0 C, onenmr probe
4.87
f1 (ppm)

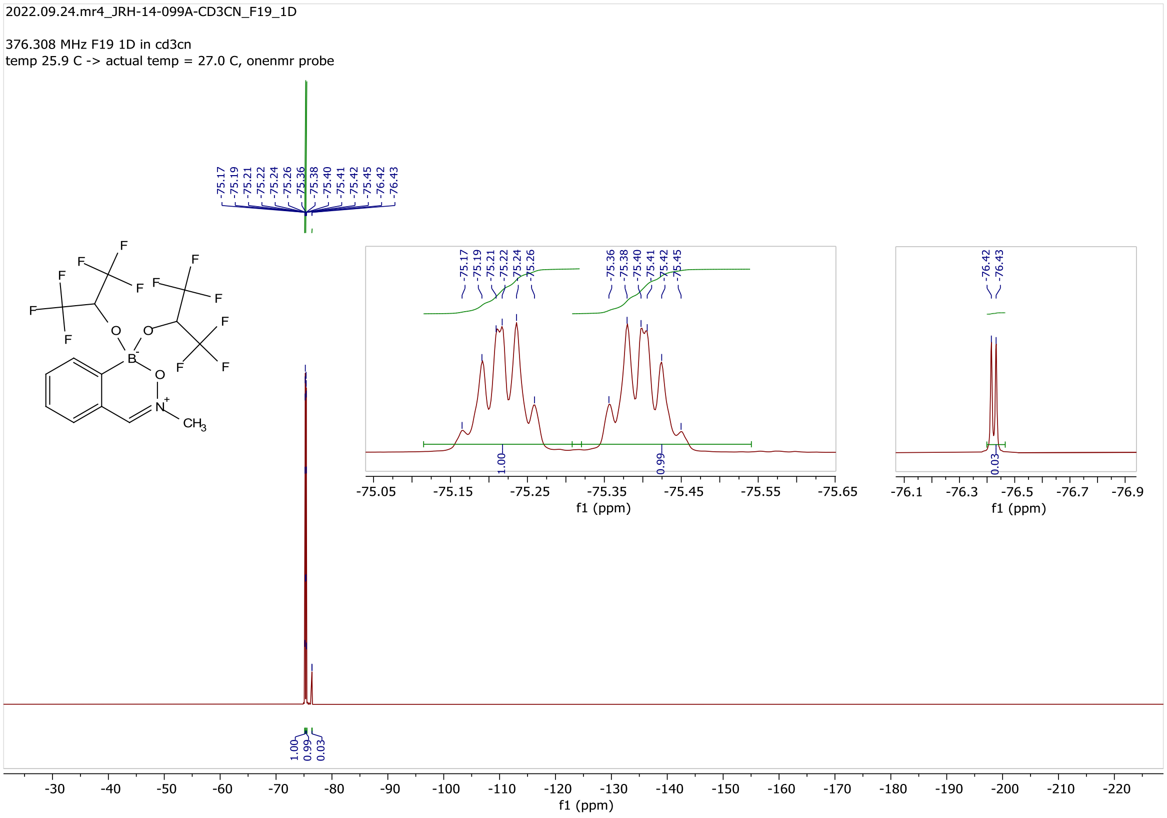
2022.09.24.mr4_JRH-14-099A-CD3CN_F19_1D
376.308 MHz F19 1D in cd3cn
temp 25.9 C -> actual temp = 27.0 C, onenmr probe
-75.17
-75.19
-75.21
-75.22
-75.24
-75.26
-75.36
-75.38
-75.40
-75.41
-75.42
-75.45
-76.42
-76.43
1.00
0.99
0.03
f1 (ppm)

12. Crystallographic Data

CCDC-2210073 (compound **3-II**) contains the supplementary crystallographic data for this paper. This data can be obtained free of charge from The Cambridge Crystallographic Data Center.

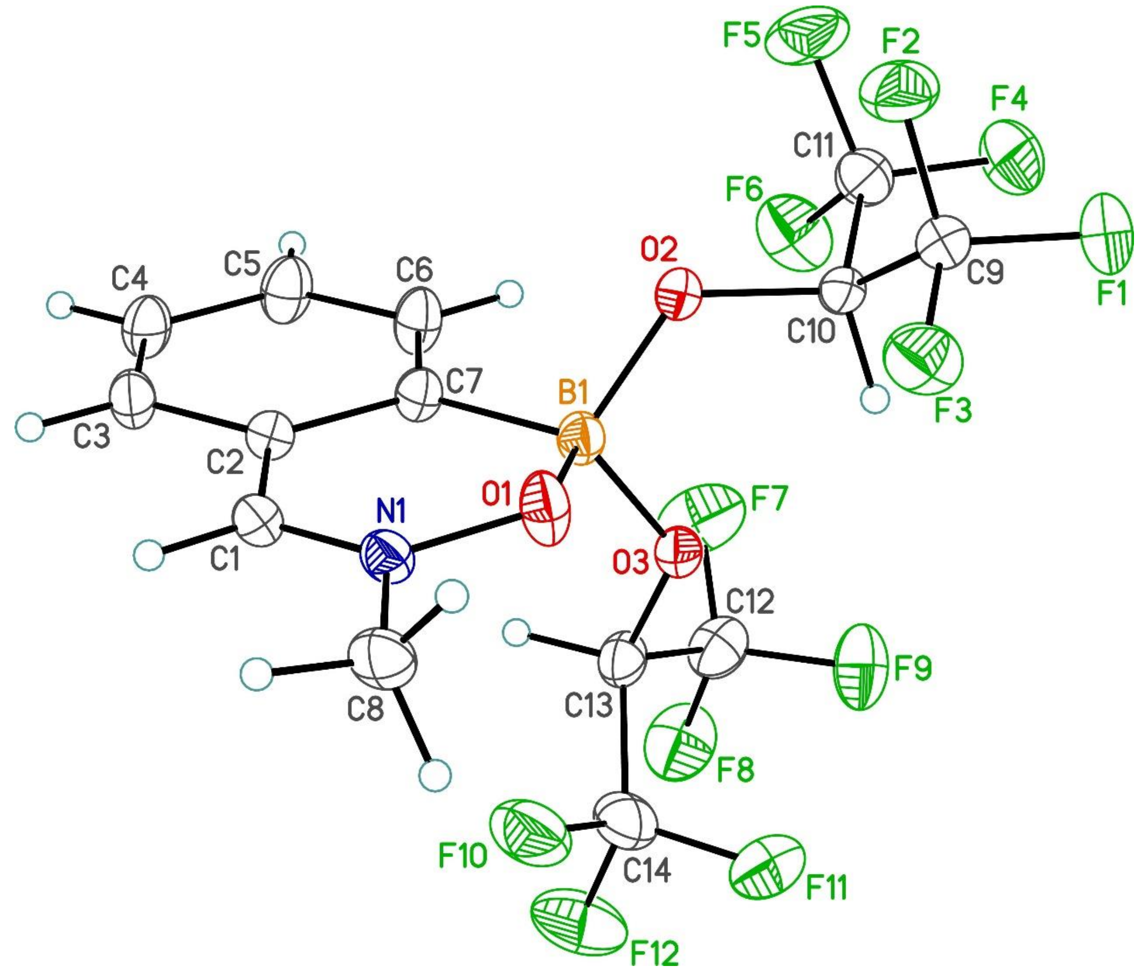

(ORTEP of compound **3-II**)

List of Tables

Table S1. Crystallographic Experimental Details

Table S2. Atomic Coordinates and Equivalent Isotropic Displacement Parameters

Table S3. Selected Interatomic Distances

Table S4. Selected Interatomic Angles

Table S5. Torsional Angles

Table S6. Anisotropic Displacement Parameters

Table S7. Derived Atomic Coordinates and Displacement Parameters for Hydrogen Atoms

Table S1. Crystallographic Experimental Details

A. Crystal Data	
formula	$C_{14}H_{10}BF_{12}NO_3$
formula weight	479.04
crystal colour and habit[a]	colourless block
crystal dimensions (mm)	0.31 × 0.14 × 0.10
crystal system	monoclinic
space group	$C2/c$ (No. 15)
unit cell parameters[b]	
a (Å)	24.821(6)
b (Å)	9.793(2)
c (Å)	17.286(4)
β (deg)	120.060(3)
V (Å^3)	3636.5(14)
Z	8
ρ_{calcd} (g cm^{-3})	1.750
μ (mm^{-1})	0.200
B. Data Collection and Refinement Conditions	
diffractometer	Bruker PLATFORM/APEX II CCD[c]
radiation (λ [Å])	graphite-monochromated Mo Kα (0.71073)
temperature (°C)	–80
scan type	ω scans (0.3°) (20 s exposures)
data collection 2θ limit (deg)	53.86
total data collected	29642 ($-31 \le h \le 31$, $-12 \le k \le 12$, $-22 \le l \le 21$)
independent reflections	3937 (R_{int} = 0.0538)
number of observed reflections (*NO*)	2982 [$F_o^2 \ge 2\sigma(F_o^2)$]
structure solution method	intrinsic phasing (*SHELXT-2014*[d])
refinement method	full-matrix least-squares on F^2 (*SHELXL–2018*[e])
absorption correction method	Gaussian integration (face-indexed)
range of transmission factors	1.0000–0.8976
data/restraints/parameters	3937 / 0 / 282
extinction coefficient (x)[f]	0.0011(2)
goodness-of-fit (S)[g] [all data]	1.058
final R indices[h]	
R_1 [$F_o^2 \ge 2\sigma(F_o^2)$]	0.0439
wR_2 [all data]	0.1245
largest difference peak and hole	0.309 and –0.323 e Å^{-3}

[a]Obtained by recrystallization from an HFIP and MeCN solution.

[b]Obtained from least-squares refinement of 5830 reflections with 4.82° < 2θ < 49.86°.

(continued)

Table S1. Crystallographic Experimental Details (continued)

[c]Programs for diffractometer operation, data collection, data reduction and absorption correction were those supplied by Bruker.

[d]Sheldrick, G. M. *Acta Crystallogr.* **2015**, *A71*, 3–8. (*SHELXT-2014*)

[e]Sheldrick, G. M. *Acta Crystallogr.* **2015**, *C71*, 3–8. (*SHELXL-2018/3*)

[f]$F_c^* = kF_c[1 + x\{0.001F_c^2\lambda^3/\sin(2\theta)\}]^{-1/4}$ where k is the overall scale factor.

[g]$S = [\Sigma w(F_o^2 - F_c^2)^2/(n - p)]^{1/2}$ (n = number of data; p = number of parameters varied; $w = [\sigma^2(F_o^2) + (0.0528P)^2 + 3.0515P]^{-1}$ where $P = [\mathrm{Max}(F_o^2, 0) + 2F_c^2]/3$).

[h]$R_1 = \Sigma||F_o| - |F_c||/\Sigma|F_o|$; $wR_2 = [\Sigma w(F_o^2 - F_c^2)^2/\Sigma w(F_o^4)]^{1/2}$.

Table S2. Atomic Coordinates and Equivalent Isotropic Displacement Parameters

Atom	*x*	*y*	*z*	U_{eq}, Å^2
F1	0.41633(7)	0.00343(14)	0.63637(10)	0.0634(4)*
F2	0.49186(6)	0.06753(15)	0.61843(9)	0.0616(4)*
F3	0.44614(6)	0.21231(13)	0.65831(8)	0.0579(4)*
F4	0.35573(7)	-0.08819(13)	0.47018(11)	0.0671(4)*
F5	0.42385(7)	-0.01764(15)	0.43899(11)	0.0730(5)*
F6	0.33003(8)	0.05629(14)	0.36430(9)	0.0703(4)*
F7	0.22774(7)	0.25910(19)	0.28681(11)	0.0825(5)*
F8	0.16422(6)	0.41041(18)	0.27977(10)	0.0750(5)*
F9	0.20655(7)	0.27653(16)	0.39212(9)	0.0692(4)*
F10	0.30857(8)	0.64044(14)	0.47285(13)	0.0777(5)*
F11	0.25134(8)	0.50887(16)	0.49826(11)	0.0703(4)*
F12	0.21001(8)	0.63027(18)	0.38054(13)	0.0939(6)*
O1	0.42364(7)	0.47574(13)	0.53111(9)	0.0436(3)*
O2	0.41519(6)	0.24613(12)	0.48293(8)	0.0340(3)*
O3	0.32299(6)	0.36885(13)	0.44214(8)	0.0358(3)*
N1	0.44324(7)	0.59833(14)	0.51952(10)	0.0329(3)*
C1	0.44089(8)	0.63665(18)	0.44650(12)	0.0341(4)*
C2	0.41445(8)	0.55541(17)	0.36664(12)	0.0321(4)*
C3	0.41733(9)	0.6033(2)	0.29231(13)	0.0391(4)*
C4	0.39233(10)	0.5266(2)	0.21568(14)	0.0444(5)*
C5	0.36482(10)	0.4020(2)	0.21282(14)	0.0479(5)*
C6	0.36234(10)	0.3542(2)	0.28669(13)	0.0440(5)*
C7	0.38693(8)	0.42962(18)	0.36544(12)	0.0337(4)*
C8	0.46869(10)	0.6811(2)	0.60086(13)	0.0417(4)*
C9	0.43628(9)	0.1048(2)	0.60585(13)	0.0402(4)*
C10	0.39033(8)	0.14146(17)	0.50972(12)	0.0328(4)*
C11	0.37524(10)	0.0216(2)	0.44585(15)	0.0464(5)*
C12	0.21708(10)	0.3470(3)	0.33570(14)	0.0515(5)*
C13	0.27190(9)	0.4450(2)	0.38312(13)	0.0399(4)*
C14	0.25994(10)	0.5561(2)	0.43356(17)	0.0524(5)*
B1	0.38412(9)	0.3781(2)	0.45110(13)	0.0318(4)*

Anisotropically-refined atoms are marked with an asterisk (*). The form of the anisotropic displacement parameter is: $\exp[-2\pi^2(h^2a^{*2}U_{11} + k^2b^{*2}U_{22} + l^2c^{*2}U_{33} + 2klb^*c^*U_{23} + 2hla^*c^*U_{13} + 2hka^*b^*U_{12})]$.

Table S3. Selected Interatomic Distances (Å)

Atom1	Atom2	Distance	Atom1	Atom2	Distance
F1	C9	1.331(2)	O3	C13	1.382(2)
F2	C9	1.335(2)	O3	B1	1.449(2)
F3	C9	1.330(2)	N1	C1	1.290(2)
F4	C11	1.331(2)	N1	C8	1.464(2)
F5	C11	1.327(2)	C1	C2	1.436(3)
F6	C11	1.333(3)	C2	C3	1.403(3)
F7	C12	1.323(3)	C2	C7	1.404(2)
F8	C12	1.331(3)	C3	C4	1.371(3)
F9	C12	1.323(3)	C4	C5	1.386(3)
F10	C14	1.334(3)	C5	C6	1.390(3)
F11	C14	1.323(3)	C6	C7	1.392(3)
F12	C14	1.328(2)	C7	B1	1.599(3)
O1	N1	1.3474(19)	C9	C10	1.514(3)
O1	B1	1.559(2)	C10	C11	1.524(3)
O2	C10	1.390(2)	C12	C13	1.525(3)
O2	B1	1.464(2)	C13	C14	1.514(3)

Table S4. Selected Interatomic Angles (deg)

Atom1	Atom2	Atom3	Angle	Atom1	Atom2	Atom3	Angle
N1	O1	B1	121.89(13)	F4	C11	F6	107.04(18)
C10	O2	B1	122.54(14)	F4	C11	C10	112.92(17)
C13	O3	B1	124.75(14)	F5	C11	F6	107.51(18)
O1	N1	C1	124.19(15)	F5	C11	C10	112.55(17)
O1	N1	C8	111.38(14)	F6	C11	C10	109.96(17)
C1	N1	C8	124.42(16)	F7	C12	F8	106.23(19)
N1	C1	C2	123.68(16)	F7	C12	F9	107.9(2)
C1	C2	C3	119.14(16)	F7	C12	C13	110.05(18)
C1	C2	C7	119.03(16)	F8	C12	F9	106.97(18)
C3	C2	C7	121.83(17)	F8	C12	C13	112.7(2)
C2	C3	C4	119.71(18)	F9	C12	C13	112.60(17)
C3	C4	C5	119.53(18)	O3	C13	C12	107.40(16)
C4	C5	C6	120.75(19)	O3	C13	C14	109.57(16)
C5	C6	C7	121.32(18)	C12	C13	C14	112.47(17)
C2	C7	C6	116.86(16)	F10	C14	F11	105.9(2)
C2	C7	B1	120.67(16)	F10	C14	F12	107.85(19)
C6	C7	B1	122.47(16)	F10	C14	C13	110.46(18)
F1	C9	F2	106.92(16)	F11	C14	F12	106.5(2)
F1	C9	F3	107.35(16)	F11	C14	C13	113.36(18)
F1	C9	C10	112.75(17)	F12	C14	C13	112.3(2)
F2	C9	F3	106.45(17)	O1	B1	O2	103.26(14)
F2	C9	C10	113.10(15)	O1	B1	O3	107.03(14)
F3	C9	C10	109.92(15)	O1	B1	C7	109.14(14)
O2	C10	C9	108.94(15)	O2	B1	O3	108.16(14)
O2	C10	C11	108.38(14)	O2	B1	C7	111.58(15)
C9	C10	C11	112.90(16)	O3	B1	C7	116.74(15)
F4	C11	F5	106.56(17)				

Table S5. Torsional Angles (deg)

Atom1	Atom2	Atom3	Atom4	Angle	Atom1	Atom2	Atom3	Atom4	Angle
B1	O1	N1	C1	-12.2(3)	C2	C7	B1	O1	-8.3(2)
B1	O1	N1	C8	169.00(16)	C2	C7	B1	O2	-121.74(18)
N1	O1	B1	O2	132.64(16)	C2	C7	B1	O3	113.20(19)
N1	O1	B1	O3	-113.34(17)	C6	C7	B1	O1	172.13(17)
N1	O1	B1	C7	13.8(2)	C6	C7	B1	O2	58.7(2)
B1	O2	C10	C9	-121.18(17)	C6	C7	B1	O3	-66.4(2)
B1	O2	C10	C11	115.62(18)	F1	C9	C10	O2	-179.04(14)
C10	O2	B1	O1	105.59(16)	F1	C9	C10	C11	-58.6(2)
C10	O2	B1	O3	-7.6(2)	F2	C9	C10	O2	-57.6(2)
C10	O2	B1	C7	-137.32(16)	F2	C9	C10	C11	62.9(2)
B1	O3	C13	C12	132.60(17)	F3	C9	C10	O2	61.24(19)
B1	O3	C13	C14	-105.0(2)	F3	C9	C10	C11	-178.31(15)
C13	O3	B1	O1	96.86(19)	O2	C10	C11	F4	174.94(16)
C13	O3	B1	O2	-152.48(16)	O2	C10	C11	F5	54.2(2)
C13	O3	B1	C7	-25.7(2)	O2	C10	C11	F6	-65.6(2)
O1	N1	C1	C2	2.8(3)	C9	C10	C11	F4	54.2(2)
C8	N1	C1	C2	-178.51(17)	C9	C10	C11	F5	-66.5(2)
N1	C1	C2	C3	-176.47(17)	C9	C10	C11	F6	173.64(16)
N1	C1	C2	C7	3.0(3)	F7	C12	C13	O3	-64.5(2)
C1	C2	C3	C4	179.90(18)	F7	C12	C13	C14	174.83(18)
C7	C2	C3	C4	0.5(3)	F8	C12	C13	O3	177.07(16)
C1	C2	C7	C6	-179.55(17)	F8	C12	C13	C14	56.4(2)
C1	C2	C7	B1	0.8(3)	F9	C12	C13	O3	55.9(2)
C3	C2	C7	C6	-0.1(3)	F9	C12	C13	C14	-64.7(2)
C3	C2	C7	B1	-179.76(17)	O3	C13	C14	F10	61.5(2)
C2	C3	C4	C5	-0.4(3)	O3	C13	C14	F11	-57.2(2)
C3	C4	C5	C6	0.0(3)	O3	C13	C14	F12	-178.02(18)
C4	C5	C6	C7	0.4(3)	C12	C13	C14	F10	-179.10(18)
C5	C6	C7	C2	-0.3(3)	C12	C13	C14	F11	62.2(2)
C5	C6	C7	B1	179.33(19)	C12	C13	C14	F12	-58.6(2)

Table S6. Anisotropic Displacement Parameters (U_{ij}, Å^2)

Atom	U_{11}	U_{22}	U_{33}	U_{23}	U_{13}	U_{12}
F1	0.0812(10)	0.0545(8)	0.0672(9)	0.0223(7)	0.0465(8)	0.0024(7)
F2	0.0495(7)	0.0765(10)	0.0626(8)	0.0155(7)	0.0308(7)	0.0246(7)
F3	0.0731(9)	0.0547(8)	0.0433(7)	-0.0048(6)	0.0271(6)	0.0076(6)
F4	0.0848(10)	0.0333(6)	0.0908(11)	-0.0067(7)	0.0497(9)	-0.0166(6)
F5	0.0838(10)	0.0594(9)	0.1044(12)	-0.0307(8)	0.0685(10)	-0.0034(7)
F6	0.0872(11)	0.0546(8)	0.0519(8)	-0.0131(6)	0.0220(8)	-0.0067(7)
F7	0.0593(9)	0.1042(13)	0.0817(11)	-0.0412(10)	0.0336(8)	-0.0182(8)
F8	0.0407(7)	0.1076(12)	0.0616(9)	0.0154(8)	0.0145(6)	0.0054(7)
F9	0.0645(9)	0.0756(10)	0.0643(9)	0.0094(7)	0.0300(7)	-0.0232(7)
F10	0.0766(10)	0.0416(7)	0.1227(14)	-0.0133(8)	0.0557(10)	-0.0045(7)
F11	0.0808(10)	0.0760(10)	0.0764(10)	-0.0066(8)	0.0559(9)	0.0053(8)
F12	0.0794(11)	0.0766(11)	0.1055(13)	0.0200(10)	0.0312(10)	0.0481(9)
O1	0.0633(9)	0.0340(7)	0.0355(7)	-0.0025(5)	0.0262(7)	-0.0152(6)
O2	0.0362(6)	0.0303(6)	0.0433(7)	0.0031(5)	0.0258(6)	-0.0011(5)
O3	0.0369(7)	0.0349(7)	0.0414(7)	0.0091(5)	0.0240(6)	0.0052(5)
N1	0.0355(8)	0.0254(7)	0.0381(8)	-0.0017(6)	0.0186(7)	-0.0012(6)
C1	0.0366(9)	0.0264(8)	0.0425(10)	0.0018(7)	0.0221(8)	0.0005(7)
C2	0.0331(9)	0.0300(9)	0.0366(9)	0.0021(7)	0.0201(7)	0.0011(7)
C3	0.0438(10)	0.0349(10)	0.0426(10)	0.0059(8)	0.0246(9)	-0.0033(8)
C4	0.0532(12)	0.0487(11)	0.0378(10)	0.0055(9)	0.0275(9)	-0.0039(9)
C5	0.0582(12)	0.0535(12)	0.0366(10)	-0.0064(9)	0.0271(9)	-0.0152(10)
C6	0.0526(11)	0.0452(11)	0.0381(10)	-0.0064(8)	0.0257(9)	-0.0178(9)
C7	0.0340(9)	0.0341(9)	0.0353(9)	0.0000(7)	0.0191(7)	-0.0043(7)
C8	0.0507(11)	0.0334(10)	0.0416(10)	-0.0093(8)	0.0235(9)	-0.0029(8)
C9	0.0483(11)	0.0361(10)	0.0470(11)	0.0054(8)	0.0320(9)	0.0065(8)
C10	0.0376(9)	0.0263(8)	0.0443(10)	0.0000(7)	0.0280(8)	-0.0008(7)
C11	0.0532(12)	0.0352(10)	0.0570(13)	-0.0045(9)	0.0321(11)	-0.0035(9)
C12	0.0417(11)	0.0680(15)	0.0439(12)	0.0035(10)	0.0207(10)	0.0014(10)
C13	0.0362(10)	0.0467(11)	0.0398(10)	0.0124(8)	0.0214(8)	0.0058(8)
C14	0.0457(12)	0.0433(12)	0.0670(14)	0.0099(10)	0.0273(11)	0.0115(9)
B1	0.0357(10)	0.0290(10)	0.0331(10)	-0.0007(8)	0.0191(8)	-0.0036(8)

The form of the anisotropic displacement parameter is:
$\exp[-2\pi^2(h^2a^{*2}U_{11} + k^2b^{*2}U_{22} + l^2c^{*2}U_{33} + 2klb^*c^*U_{23} + 2hla^*c^*U_{13} + 2hka^*b^*U_{12})]$

Table S7. Derived Atomic Coordinates and Displacement Parameters for Hydrogen Atoms

Atom	*x*	*y*	*z*	U_{eq}, Å^2
H1	0.457582	0.723488	0.445493	0.041
H3	0.436507	0.688483	0.295044	0.047
H4	0.393842	0.558520	0.164915	0.053
H5	0.347476	0.348754	0.159780	0.057
H6	0.343497	0.268305	0.283385	0.053
H8A	0.434732	0.711769	0.610068	0.063
H8B	0.490235	0.760639	0.594793	0.063
H8C	0.498136	0.626202	0.652187	0.063
H10	0.350928	0.174366	0.505904	0.039
H13	0.280527	0.487984	0.337957	0.048

Supplementary References

1. Kazmi, M. Z. H., Rygus, J. P. G., Ang, H. T., Paladino, M., Johnson, M. A., Ferguson, M. J., Hall, D. G. *J. Am. Chem. Soc.* **143**, 10143–10156 (2021).

2. Farre, A., Soares, K., Briggs, R. A., Balanta, A., Benoit, D. M., Bonet, A. *Chem. Eur. J.* **22**, 17552–17556 (2016).

3. Inada, H., Shibuya, M., Yamamoto, Y. *J. Org. Chem.* **85**, 11047–11059 (2020).

4. Cao, Y.-X., Zhu, G., Li, Y., Le Breton, N., Gourlaouen, G., Choua, S., Boixel, J., Jacquot de Rouville, H.-P., Soulé, J-F. *J. Am. Chem. Soc.* **144**, 5902–5909 (2002).

5. Zhang, Y., He, L., Shi, L. *Adv. Synth. Catal.* **360**, 1926–1931 (2018).

6. Li, J., He, L., Liu, X., Cheng, X., Li, G. *Angew. Chem. Int. Ed.* **58**, 1759–1763 (2019).

7. Garcia, K. J., Gilbert, M. M., Weix, D. J. *J. Am. Chem. Soc.* **141**, 1823–1827 (2019).

8. Shinohara, K., Tsurugi, H., Anwander, R., Mashima, K. *Chem. Eur. J.* **26**, 14130–14136 (2020).

9. Uchiyama, M., Kobayashi, Y., Furuyama, T., Nakamura, S., Kajihara, Y., Miyoshi, T., Sakamoto, T., Kondo, Y., Morokuma, K. *J. Am. Chem. Soc.* **130**, 472–480 (2008).

10. Lerebours, R., Wolf, C. *J. Am. Chem. Soc.* **128**, 13052–13053 (2006).

11. Caspers, L. D., Spils, J., Damrath, M., Lork, E., Nachtsheim, B. J. *J. Org. Chem.* **85**, 9161–9178 (2020).

12. Zhang, G., Scott, B. L., Hanson, S. K. *Angew. Chem. Int. Ed.* **51**, 12102–12106 (2012).

13. Yu, J-Y., Kuwano, R. *Org. Lett.* **10**, 973–976 (2008).

14. Balakrishnan, V., Murugesan, V., Chindan, B., Rasappan, R. *Org. Lett.* **23**, 1333–1338 (2021).

15. Zhang, X., Yang, C., Gao, H., Wang, L., Guo, L., Xia, W. *Org. Lett.* **23**, 3472–3476 (2021).

16. Dhiman, S., Ramasastry, S. S. V. *Org. Biomol. Chem.* **11**, 8030–8035 (2013).

17. Lei, C., Yip, Y. J., Zhou, J. S. *J. Am. Chem. Soc.* **139**, 6086–6089 (2017).

18. Isbrandt, E. S., Nasim, A., Zhao, K., Newman, S. G. *J. Am. Chem. Soc.* **143**, 14646–14656 (2021).

19. Xi, Z.-W., Yang, L., Wang, D.-Y., Feng, C.-W., Qin,Y., Shen, Y.-M., Pu, C., Peng, X. *J. Org. Chem.* **86**, 2474–2488 (2021).

20. Heijnen, D., Helbert, H., Luurtsema, G., Elsinga, P. H., Feringa, B. L. *Org. Lett.* **21**, 4087–4091 (2019).

21. Kourist, R., González-Sabin, J., Liz, R., Rebolledo, F. *Adv. Synth. Catal.* **347**, 695–702 (2005).

22. Potenti, S., Gualandi, A., Puggoli, A., Fermi, A., Bergamini, G., Cozzi, P. G. *Eur. J. Org. Chem.* **11**, 1624–1627 (2021).

23. Kirmse, W., Krzossa, B., Steenken, S. *J. Am. Chem. Soc.* **118**, 7473–7477 (1996).

24. Stridfeldt, E., Lindstedt, E., Reitti, M., Blid, J., Norrby, P.-O., Olofsson, B. *Chem. Eur. J.* **23**, 13249–13258 (2017).

25. Ai, J.-J., Liu, B.-B., Li, J., Wang, F., Huang, C-M., Rao, W., Wang, S.-Y. *Org. Lett.* **23**, 4705–4709 (2021).

26. Meng, M., Yang, L., Cheng, K., Qi, C. *J. Org. Chem.* **83**, 3275–3284 (2018).

27. Yu, C., Huang, R., Patureau, F. W. *Angew. Chem. Int. Ed.* **61**, e202201142 (2022).

28. Regier, J., Ghanty, S., Bolshan, Y. *J. Org. Chem.* **87**, 524–530 (2022).

29. Vasilopoulos, A., Zultanski, S. L., Stahl, S. S. *J. Am. Chem. Soc.* **139**, 7705–7708 (2017).

30. Wang, X., Liu, L.-H., Shi, J.-H., Peng, J., Tu, H.-Y., Zhang, A.-D. *Eur. J. Org. Chem.* **30**, 6870–6877 (2013).

31. Zhao, G., Yuan, L.-Z., Alami, M., Provot, O. *Adv. Synth. Catal.* **360**, 2522–2536 (2018).

32. Song, J., Li, Y., Sun, W., Yi, C., Wu, H., Wang, H., Ding, K., Xiao, K., Liu, C. *New. J. Chem.* **40**, 9030–9033 (2016).

33. Bernhardt, S., Shen, Z.-L., Knochel, P. *Chem. Eur. J.* **19**, 828–833 (2013).

34. Wang, X.-X., Xu, B.-B., Song, W.-T., Sun, K.-X., Lu, J.-M. *Org. Biomol. Chem.* **13**, 4925–4930 (2015).

35. Suga, T., Ukaji, Y. *Org. Lett.* **20**, 7846–7850 (2018).

36. Yoon, S., Hong, M. C., Rhee, H. *J. Org. Chem.* **79**, 4206–4211 (2014).

37. Yuguchi, M., Tokuda, M., Orito, K. *J. Org. Chem.* **69**, 908–914 (2004).

38. Aguila, M. J. B., Badiei, Y. M., Warren, T. H. *J. Am. Chem. Soc.* **135**, 9399–9406 (2013).

39. Sai, M. *Adv. Synth. Catal.* **360**, 4330–4335 (2018).

40. Udagawa, T., Kogawa, M., Tsuchi, Y., Watanabe, H., Yamamoto, M., Kawatsura, M. *Tetrahedron Lett.* **58**, 227–230 (2017).

41. Liu, H., Yin, B., Gao, Z., Li, Y., Jiang, H. *Chem. Commun.* **48**, 2033–2035 (2012).

42. Tsubouchi, A., Muramatsu, D., Takeda, T. *Angew. Chem. Int. Ed.* **52**, 12719–12722 (2013).

43. Kariofillis, S. K., Jiang, S., Żurański, A. M., Gandhi, S. S., Martinez Alvarado, J. I., Doyle, A. G. *J. Am. Chem. Soc.* **144**, 1045–1055 (2022).

44. Jana, S. K., Maiti, M., Dey, P., Maji, B. *Org. Lett.* **24**, 1298–1302 (2022).

45. Meng, Q.-Y., Schirmer, T. E., Berger, A. L., Donabauer, K., König, B. *J. Am. Chem. Soc.* **141**, 11393–11397 (2019).

46. Leow, D., Chen, Y.-H., Hung, T.-Z., Lin, Y.-Z. *Eur. J. Org. Chem.* **33**, 7347–7352 (2014).

47. Pincock, J. A., Wedge, P. J. *J. Org. Chem.* **59**, 5587–5595 (1994).

48. Sun, Y.-Y., Yi, J., Lu, X., Zhang, Z.-Q., Xiao, B., Fu, Y. *Chem. Commun.* **50**, 11060–11062 (2014).

49. Perkins, R. J., Pedro, D. J., Hansen, E. C. *Org. Lett.* **19**, 3755–3758 (2017).

50. Chen, C.-R., Zhou, S., Biradar, D. B., Gau, H.-M. *Adv. Synth. Catal.* **352**, 1718–1727 (2010).

51. Zhao, C., Zha, G.-F., Fang, W.-Y., Rakesh, K. P., Qin, H.-L. *Eur. J. Org. Chem.* **8**, 1801–1807 (2019).

52. Mikhael, M., Guo, W., Tantillo, D. J., Wengryniuk, S. E. *Adv. Synth. Catal.* **363**, 4867–4875 (2021).

53. Jereb, M., Vražič, D. *Org. Biomol. Chem.* **11**, 1978–1999 (2013).

54. Surya Prakash, G. K., Do, C., Mathew, T., Olah, G. A. *Catal. Lett.* **141**, 507–511 (2011).

55. Fukuyama, T., Fujita, Y., Miyoshi, H., Ryu, I., Kao, S.-C., Wu, Y.-K. *Chem. Commun.* **54**, 5582–5585 (2018).

56. Chan, Y.-C., Bai, Y., Chen, W.-C., Chen, H.-Y., Li, C.-Y., Wu, Y.-Y., Tseng, M.-C., Yap, G. A. P., Zhao, L., Chen, H.-Y., Ong, T.-G. *Angew. Chem. Int. Ed.* **60**, 19949–19956 (2021).

57. Xu, Q., Xie, H., Chen, P., Yu, L., Chen, J., Hu, X. *Green Chem.* **17**, 2774–2779 (2015).

58. Song, J., Li, Y., Sun, W., Yi, C., Wu, H., Wang, H., Ding, K., Xiao, K., Liu, C. *New. J. Chem.* **40**, 9030–9033 (2016).
